# Intermolecular Asymmetric Arylative Dearomatization of 1-Naphthols

**DOI:** 10.1021/jacs.4c14754

**Published:** 2024-12-04

**Authors:** Max Kadarauch, Thomas A. Moss, Robert J. Phipps

**Affiliations:** † Yusuf Hamied Department of Chemistry, University of Cambridge, Lensfield Road, Cambridge CB2 1EW, U.K.; ‡ Oncology Medicinal Chemistry, R&D AstraZeneca, The Discovery Centre (DISC), Trumpington, Cambridge CB2 0AA, U.K.

## Abstract

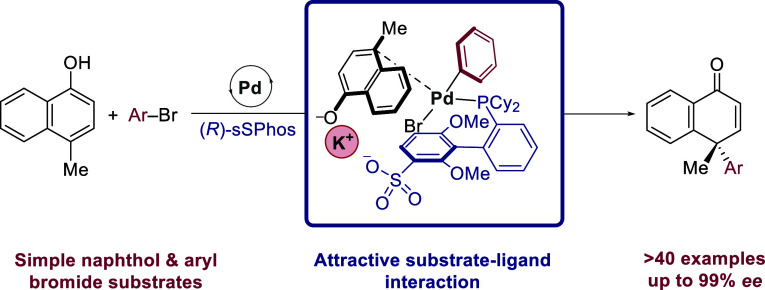

Arylative dearomatization forms quaternary stereocenters in cyclic systems with the concomitant introduction of an aromatic ring. Pd-catalyzed arylative dearomatization, which uses conditions analogous to cross-coupling, has emerged as a powerful method in an intramolecular context. But translating this from intramolecular cyclizations to an intermolecular process has proven extremely challenging: examples are scarce, and those that exist have not been rendered enantioselective, despite the potential for broad application in medicinal chemistry and natural product synthesis. We describe a strategy that utilizes attractive interactions between the ligand and substrate to overcome this challenge and promote intermolecular, highly enantioselective arylative dearomatization of naphthols using a broad range of aryl bromide electrophiles. Crucial to success is the use of the readily accessed sulfonated chiral phosphine sSPhos, which we believe engages in attractive electrostatic interactions with the substrate. Not only does sSPhos control enantioselectivity but it also drastically accelerates the reaction, most likely by facilitating the challenging palladation step that initiates dearomatization.

## 1 Introduction

**Figure 1:**
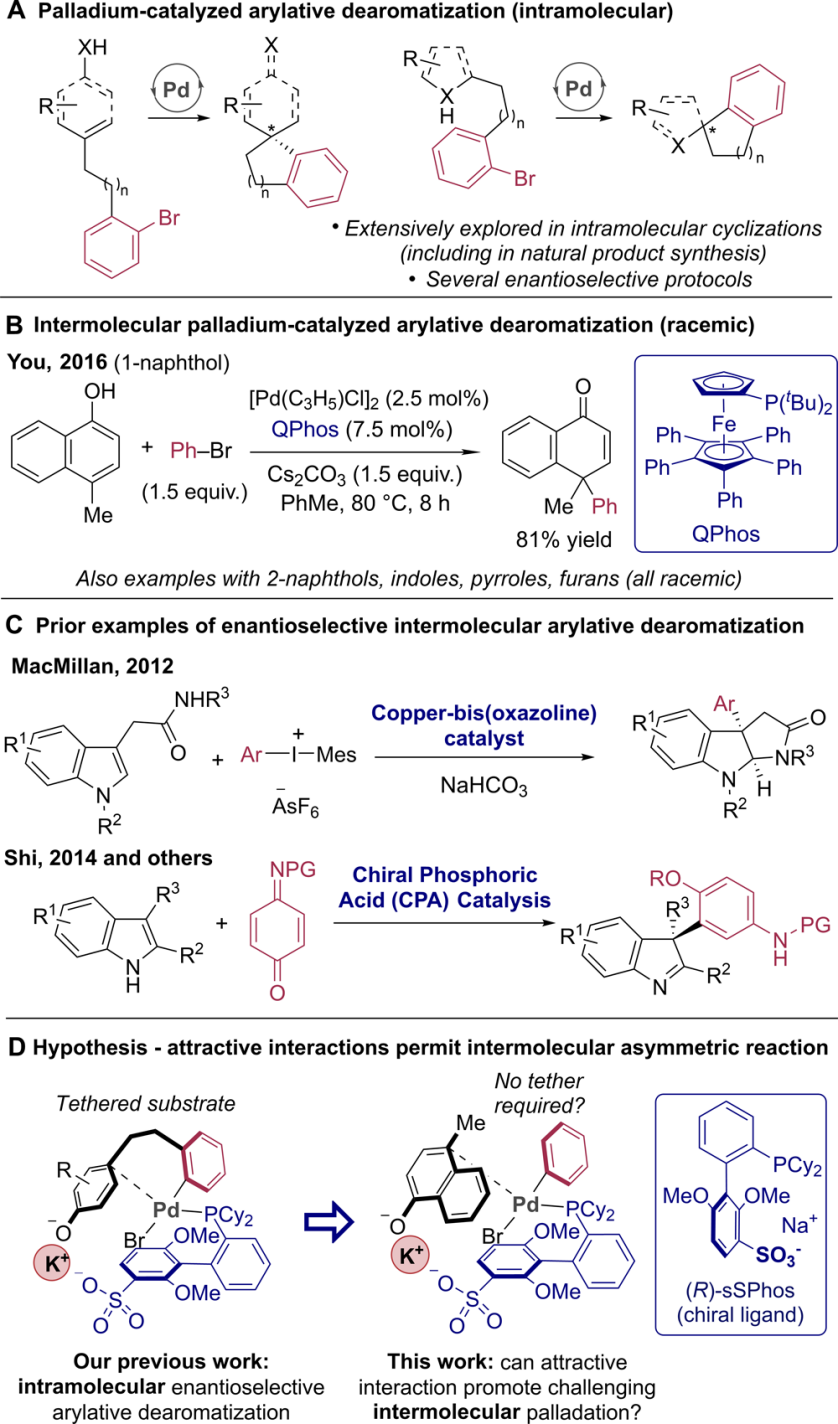
Context for enantioselective intermolecular arylative dearomatization and our hypothesis using sSPhos.

Arene or heteroarene dearomatization reactions are powerful complexity-increasing processes.^1^ Although the breaking of aromaticity requires significant energy input, versatile three-dimensional, stereocenter-containing products are typically formed, which are poised for further elaboration through the remaining unsaturation.^2^ Accordingly, much effort has been put into developing protocols that induce asymmetry during the dearomatizing event. These have been achieved on a variety of electron-rich arene or heteroarene substrates using various electrophiles; the use of highly reactive electrophiles naturally assists in overcoming the barrier to dearomatization, making the task of rendering the process enantioselective somewhat easier.^3^ In this context, arylative dearomatization is particularly challenging due to a paucity of highly electrophilic aryl surrogates. Yet since it results in an aryl group linked to a quaternary stereocenter, a structural feature that is ubiquitous in bioactive small molecules, it remains an important and challenging problem to address in an enantioselective manner.^4^ Methods for arylative dearomatization that utilize electrophilic lead,^5^ bismuth,^6^ and iodine^7^ arylating reagents have not translated into enantioselective versions because most do not operate under the control of a catalyst. The pioneering development by Buchwald and co-workers of palladium-catalyzed arylative dearomatization methods, using aryl bromides as the electrophilic partner, marked a significant step forward.^8^ Since then, the protocol has been applied in natural product synthesis and a number of asymmetric examples of this reaction have been reported using chiral ligands for palladium, making it arguably the leading strategy for enantioselective arylative dearomatization (Figure 1A).^9^ One aspect that stands out from surveying the asymmetric examples is that they are all intramolecular. There are very few examples of intermolecular arylative dearomatization using palladium catalysis, and none are enantiocontrolled. The most prominent are those of 1-naphthols and 2-naphthols, both from You and co-workers,^10^ as well as a handful of protocols reported on furans,^11^ indoles,^12^ and pyrroles (Figure 1B).^13^ In fact, there are very few enantioselective examples of intermolecular arylative dearomatization using any catalytic method. One notable example is Zhu and MacMillan’s 2012 report of indole dearomatization using iodonium salts and chiral copper catalysis, which was subsequently adapted for total synthesis by Reisman and co-workers,^14^ but has not since been further explored in other dearomatization reactions (Figure 1C, upper).^15^ In addition, there are examples of formal arylative dearomatization processes using quinone imides and related aryl-precursor electrophiles under chiral phosphoric acid catalysis, although these are, by their nature, limited to the introduction of very specific arene motifs (Figure 1C, lower).^16^ It is evident that the translation of arylative dearomatization from intramolecular cyclizations to intermolecular couplings is not a trivial matter and remains an outstanding challenge. The two pioneering racemic protocols reported by You using palladium catalysis were highly ligand-specific, requiring the use of achiral QPhos; no yield was obtained using the other ligands evaluated, a significant barrier to the development of asymmetric variants.^10^

We previously reported the use of sSPhos^17,18^ as a general chiral ligand for intramolecular arylative dearomatization and applied it to a range of different scaffolds.^9j^ We attribute its efficacy to a key attractive electrostatic interaction between the ligand sulfonate group, alkali metal cation, and phenolate at the transition state for arene palladation (Figure 1D, left). This should permit a high degree of organization in the defined chiral environment provided by the ligand.^19^ In considering the challenge of intermolecular arylative dearomatization, we speculated as to whether this network of interactions could constitute a key enabling feature, accelerating the challenging palladation step by emulating intramolecularity while simultaneously enforcing enantiofacial control (Figure 1D, right). In this article, we demonstrate that this is indeed feasible and highly effective. Additionally, we illustrate the versatility of the products in the synthesis of pharmaceutical analogues featuring quaternary stereocenters, as well as an application to natural product synthesis.

## 2 Results and Discussion

We commenced with 4-methylnaphthalen-1-ol (**1a**) as the substrate and based our initial conditions on those reported by You and co-workers, with [PdCl(allyl)]_2_ as the Pd source and Cs_2_CO_3_ as the base, replacing achiral ligand QPhos with (*R*)-sSPhos (Table 1, entry 1).^10a^ While excellent enantioselectivity (98% *ee*) was obtained, the yield was poor. An evaluation of palladium sources (entries 2 and 3) afforded the best results with Pd_2_dba_3_ (entry 3). Next, the base was varied (entries 3–7), with optimal yield being obtained with K_3_PO_4_ (61%) and excellent enantioselectivity maintained.^20^ A slight reduction in yield was obtained upon reduction of the reaction temperature to 70 °C (entry 8). While halving the ligand loading had a detrimental effect on reactivity (entry 9), reactivity was maintained when the loading of Pd_2_dba_3_ was halved in entry 10, which formed our final optimized conditions.^21^

**Table 1:**
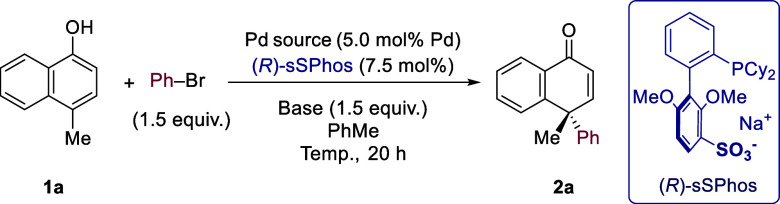
Reaction Optimization

entry	Pd source	base	temp/°C	yield/%^a^	ee/%^b^
1	[PdCl(allyl)]_2_	Cs_2_CO_3_	90	18	98
2	Pd(OAc)_2_	Cs_2_CO_3_	90	trace	N.D.
3	Pd_2_dba_3_	Cs_2_CO_3_	90	35	96
4	Pd_2_dba_3_	K_2_CO_3_	90	42	83
5	Pd_2_dba_3_	KOH	90	49	99
6	Pd_2_dba_3_	NaOH	90	54	99
7	Pd_2_dba_3_	K_3_PO_4_	90	61	95
8	Pd_2_dba_3_	K_3_PO_4_	70	55	96
9^c^	Pd_2_dba_3_	K_3_PO_4_	90	23	96
**10**^d^	**Pd_2_dba_3_**	**K**_**3**_**PO**_**4**_	**90**	**62 (63)**	**97 (97)**

a Yields determined by ^1^H NMR with reference to a dibromomethane internal standard. The value in parentheses refers to isolated yield.

b *ee* determined by SFC analysis of the crude reaction mixture. The value in parentheses refers to *ee* of isolated material.

c Reaction carried out with 3.75 mol % (*R*)-sSPhos.

d Reaction carried out with 2.5 mol % Pd.

**Scheme 1:**
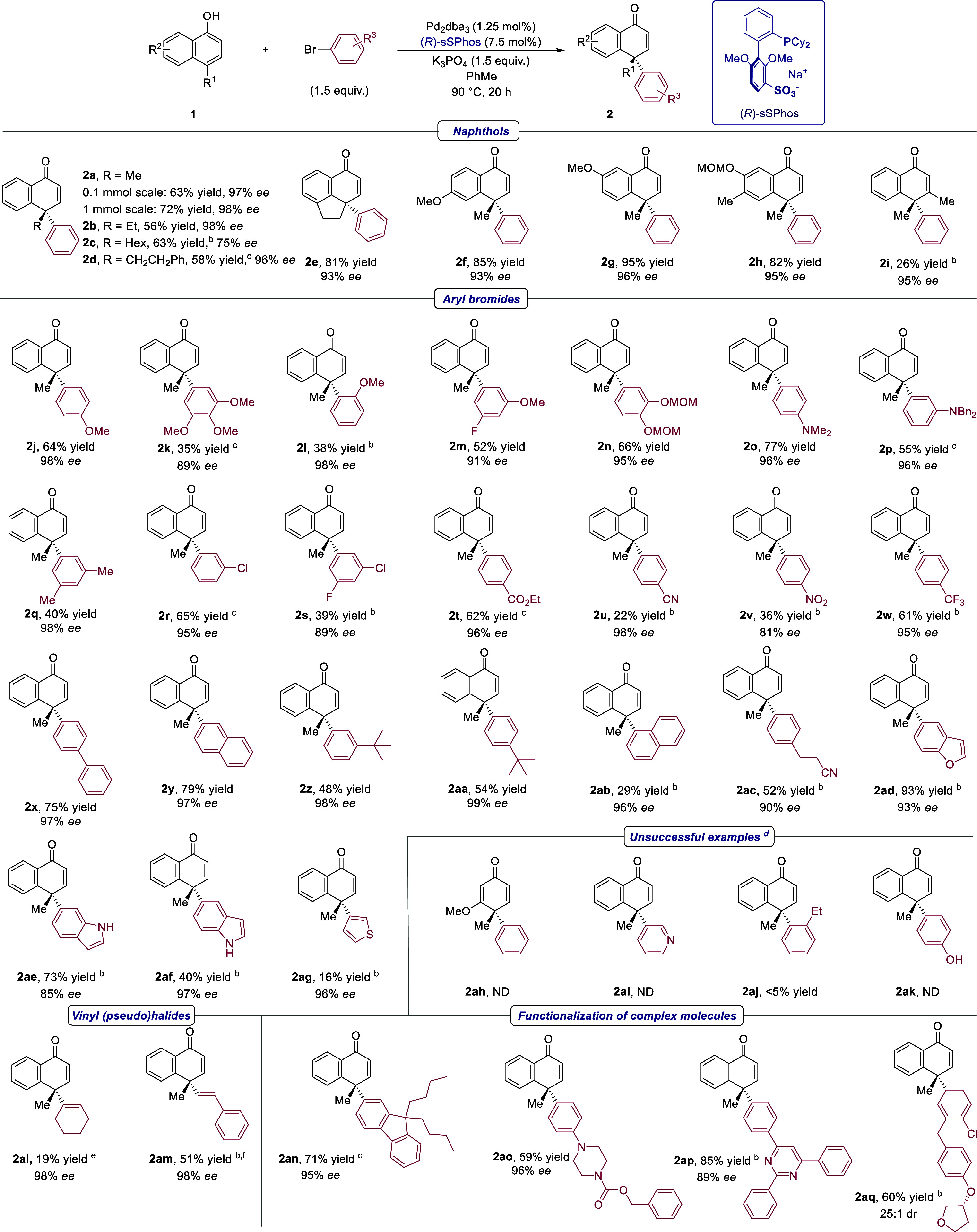
Scope of Intermolecular Arylative 1-Naphthol Dearomatization Yields are isolated, and *ee* values determined by SFC. 2.5 mol % Pd_2_dba_3_ and 15 mol % (*R*)-sSPhos for 48 h. 48 h reaction time. Unsuccessful examples were carried out using (*rac*)-sSPhos along with minor variations to the reaction conditions. For full details, see the Supporting Information. ND = Not detected. Vinyl triflate used as the electrophile instead of vinyl bromide. Product isolated with 11% of an unidentified inseparable impurity.

**Scheme 2:**
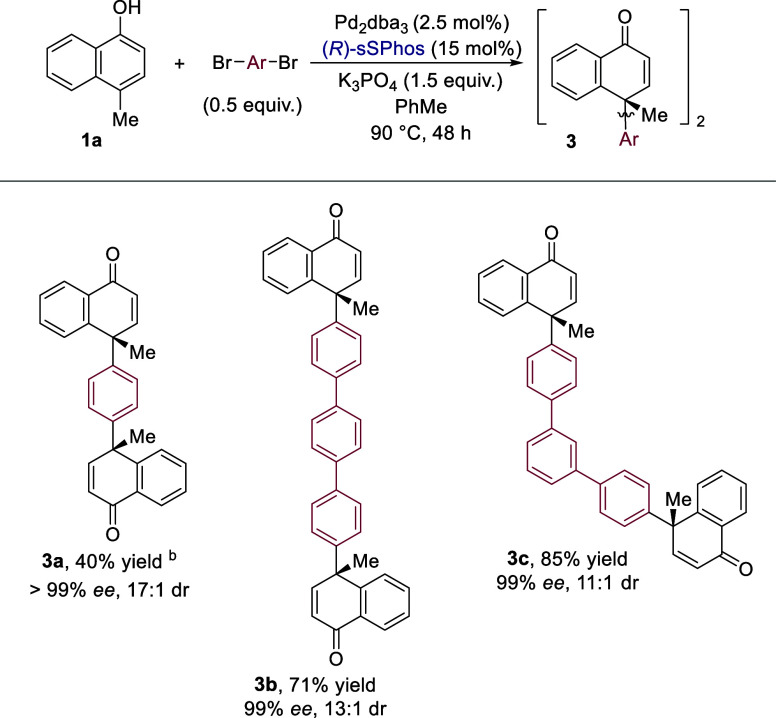
Evaluation of Bifunctional Electrophiles Yields are isolated. Products were isolated as a mixture of diastereomers. *ee* and dr values were determined by chiral SFC. Reaction conducted with 0.75 equiv. 1,4-dibromobenzene

With the optimized conditions in hand, we established that the reaction could be conducted on a 1 mmol scale, affording very similar results (Scheme 1, **2a**, 72% yield, 98% *ee*). Next, the 1-naphthol scope was evaluated. Several different alkyl substituents were tolerated at the C4 position in place of the methyl (**2a**–**2d**). Lower enantioselectivity was obtained with a hexyl substituent (**2c**) but remained high with a similarly large phenethyl substituent (**2d**). Tricyclic (**2e**) and methoxy-substituted naphthols (**2f** and **2g**) displayed excellent reactivity as well as enantioselectivity, and a substrate containing a MOM group was tolerated without any issue (**2h**). Methyl substitution at the C3 position (**2i)** impacted reactivity, presumably due to greater steric hindrance at the site of arylation, but product **2i** was still obtained in excellent enantioselectivity. The aryl bromide component tolerated a wide range of electron-donating substituents (**2j**–**2q**). Electron-withdrawing substituents also gave excellent selectivity outcomes, although slightly more forcing conditions were required to improve the conversion in some cases (**2r**–**2w**). Even in cases of extremely withdrawing substituents such as cyano (**2u**) or nitro (**2v**) which gave low yields, selectivity remained high. The absolute configuration of chlorine-substituted **2r** was determined by X-ray crystallography, with the stereochemistry of the remaining compounds assigned by analogy. Bulky substituents were tolerated in the *meta* and *para* positions of the aromatic ring (**2x–2aa**) but a naphthyl-based aryl bromide bearing bulk in the *ortho* position was less reactive (**2ab**). An aryl bromide containing a nitrile group separated from the arene by an aliphatic chain gave good results (**2ac**). We were very pleased to find that electron-rich heteroaryl bromides worked very well, including a benzofuran (**2ad**), two different isomers of an unprotected indole (**2ae** and **2af**), and even a thiophene (**2ag**), albeit in low yield. Such excellent tolerance was unexpected given the precedent for electron-rich heterocycles to undergo direct arylation reactions under similar Pd-catalyzed conditions.^22^ As for the limitations, intermolecular dearomatization of a phenol nucleophile, as opposed to that of a naphthol, appears too challenging (**2ah**). Regarding unsuccessful aryl bromides, no product was obtained using 3-bromopyridine (**2ai**), *ortho-*ethyl (**2aj**), or a *para*-phenolic (**2ak**) aryl bromide. We discovered that the reaction can extend beyond arylation: a cyclic vinyl triflate (**2al**) and an acyclic, styrenyl bromide (**2am**) were compatible, although further work is required to improve the yield. Finally, we applied the arylative dearomatization to more complex aryl bromides in **2an**–**2aq**, demonstrating the tolerance of this methodology to useful and medicinally relevant functionality.^23^ Surprisingly, O/C2-arylation were not generally observed as major byproducts when evaluating the reaction scope; the moderate yields of several scope examples were attributed to the gradual decomposition of 4-methylnaphthalen-1-ol (**1a**) under the reaction conditions (for details, see the Supporting Information).

We were curious to probe whether the catalyst could exert enantiocontrol on the formation of more than one stereocenter in a single reaction by carrying out the arylative dearomatization using bisbromoarenes (Scheme 2). Gratifyingly, these doubly functionalized products could be formed with good yields, enantioselectivities, and diastereoselectivities (**3a**–**3c**). Diastereomers were inseparable by column chromatography and indistinguishable by NMR: the presence of the minor *meso* diastereomer was detectable only by chiral SFC analysis.

**Scheme 3:**
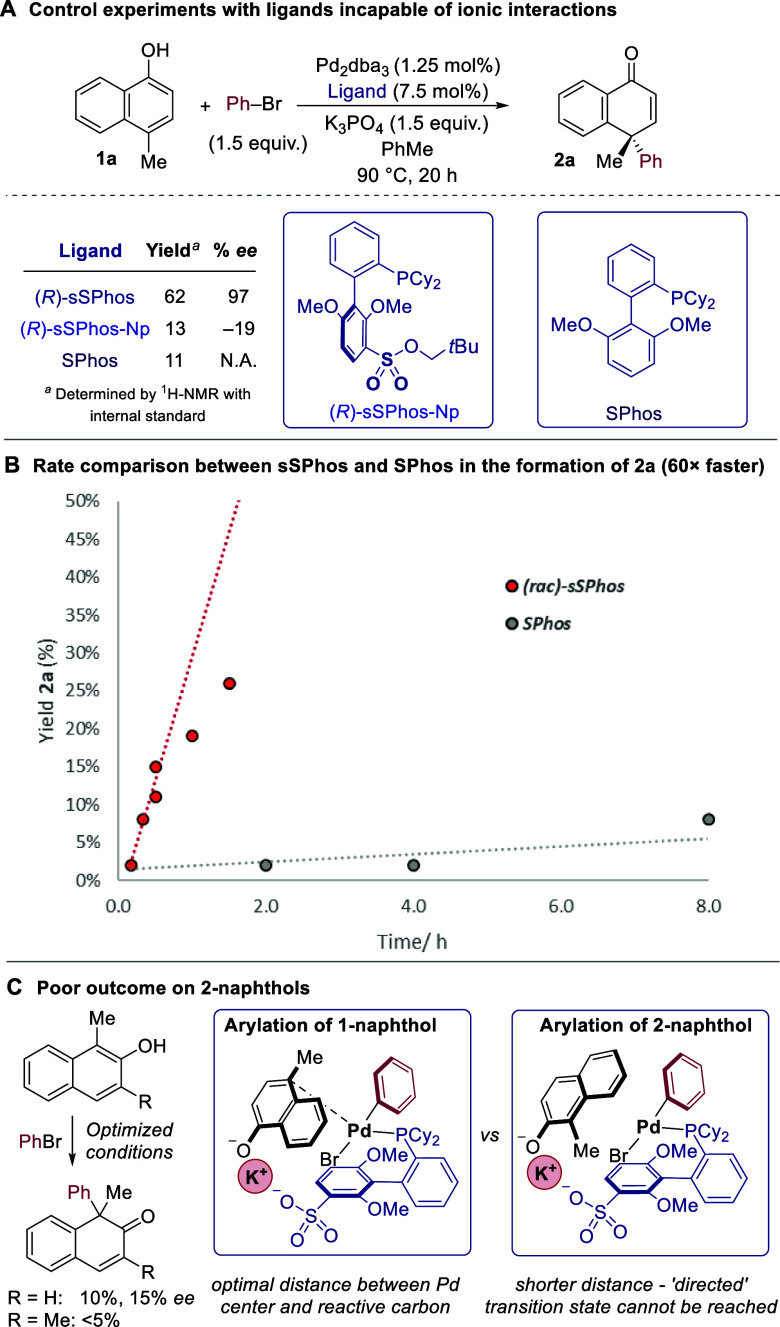
Experiments to Probe Electrostatic Substrate–Ligand Interaction

We next carried out control experiments to gain support for the proposed substrate–ligand ionic interaction (Figure 1D, right). The ligand (*R*)-sSPhos-Np, in which the anionic sulfonate group was converted to a neutral sulfonate ester, was evaluated under the optimized conditions, affording very poor enantioselectivity when compared to (*R*)-sSPhos (–19 vs 97% *ee*, Scheme 3A). Interestingly, the yield was also greatly impacted (13 vs 62%). To further probe this, standard SPhos was also tested, affording an almost identically poor yield. These results are consistent with our hypothesis that the ionic interaction is needed to emulate intramolecularity and promote the challenging carbopalladation step. We envisage that the resulting high degree of organization at the transition state permits the steric environment of the chiral ligand scaffold to exert maximum effect in delivering the excellent enantioselectivities observed in this work. We next compared the rates of the reaction with sSPhos and SPhos and discovered that with sSPhos, the reaction proceeds approximately 60 times faster (Scheme 3B). This provides further support that the nucleophilic C4 carbon of 1-naphthol is ideally situated to interact with the palladium when it is positioned by the key substrate–ligand interaction. Interestingly, we found two closely related 1-methyl-2-naphthols to be very poor substrates under the optimized conditions (Scheme 3C, left). We speculate that here, the reactive site for arylation is too close to the phenolate to fit properly with the ligand (Scheme 3C, right). Low enantioselectivity was also obtained in the cyclization of a suitable 1-substituted-2-naphthol substrate bearing a tethered aryl bromide, suggesting that this trend also applies to the substrates investigated in our earlier intramolecular work (for details, see the Supporting Information).^9j^

**Scheme 4:**
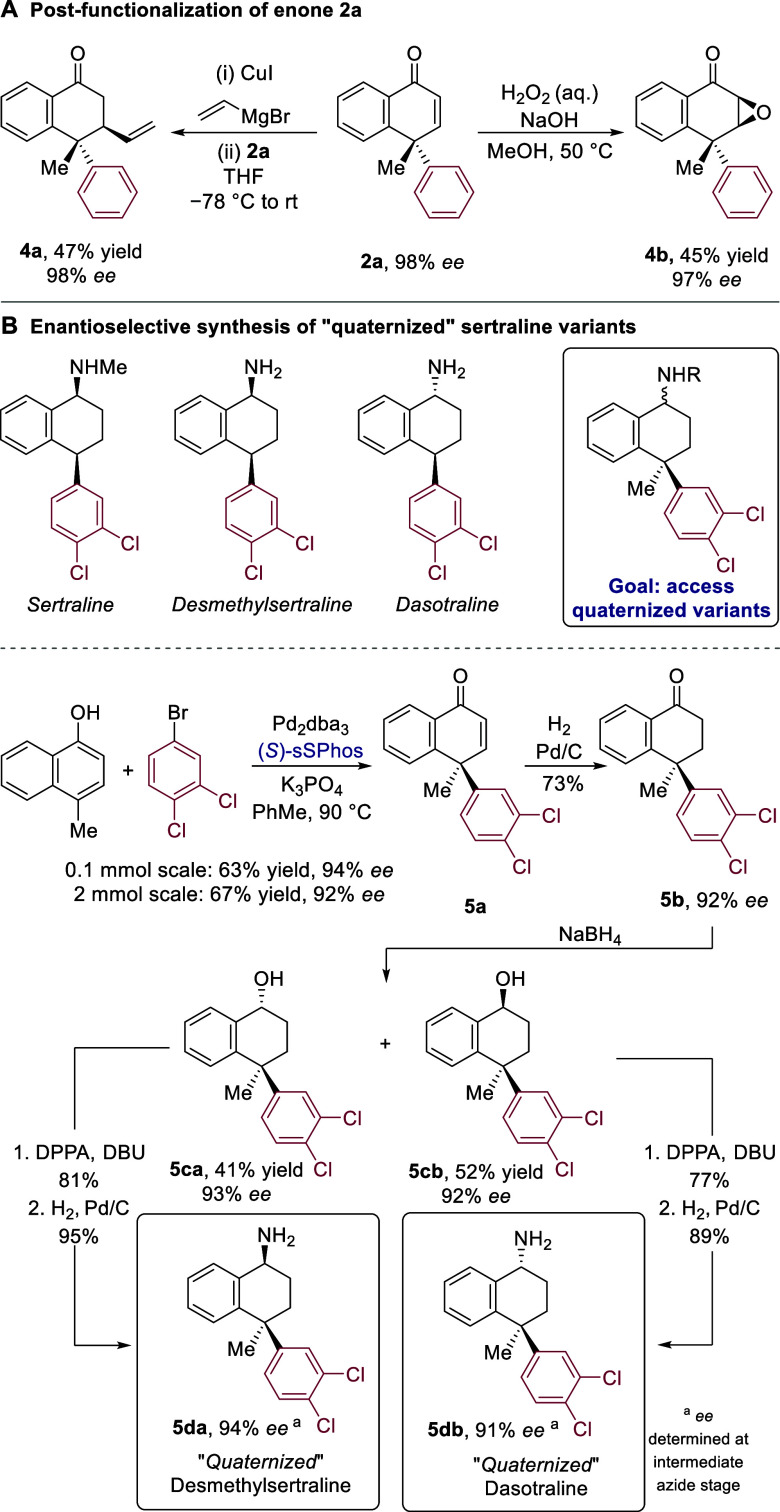
Post-functionalization and Synthesis of Quaternized Variants of Sertraline

The enone motif that remains following dearomatization can be further transformed in a multitude of ways. We demonstrate two: vinyl cuprate addition to enone **2a** gave a single isolated diastereomer of alkene **4a** (crude NMR indicated a 5:1 dr) and nucleophilic epoxidation using basic peroxide formed epoxide **4b** as a single diastereomer (Scheme 4A). We next sought to demonstrate the potential of our method for medicinal chemistry applications. Sertraline is a blockbuster single enantiomer antidepressant drug (Scheme 4B, upper).^24^ Desmethylsertraline, an active metabolite of sertraline, has also demonstrated in vivo activity as a monoamine uptake inhibitor.^25^ Dasotraline, an epimer of desmethylsertraline featuring the opposite configuration at the amine stereocenter, was investigated in clinical trials for the treatment of ADHD in 2015, demonstrating the contemporary relevance of these molecules in drug discovery long after the approval of sertraline by the FDA in 1991.^26^ Variants of these molecules containing a quaternary stereocenter arguably present more challenging synthetic targets, inspiring us to showcase our methodology by accessing “quaternized” analogues of these pharmaceuticals (Scheme 4B, upper right).^27^ The arylative dearomatization, using (*S*)-sSPhos to install the correctly configured stereocenter, delivered enone **5a** in 92% *ee* on a 2 mmol scale, which could be converted to tetralone **5b** following hydrogenation with Pd/C (Scheme 4B, lower). Since both amine configurations are relevant targets, we were pleased to find that the reduction with NaBH_4_ was unselective, delivering separable alcohol diastereomers **5ca** and **5cb**. These were converted to the corresponding free amines **5da** and **5db** through Mitsunobu inversion to form the corresponding azide (not shown), followed by hydrogenation.

**Scheme 5:**
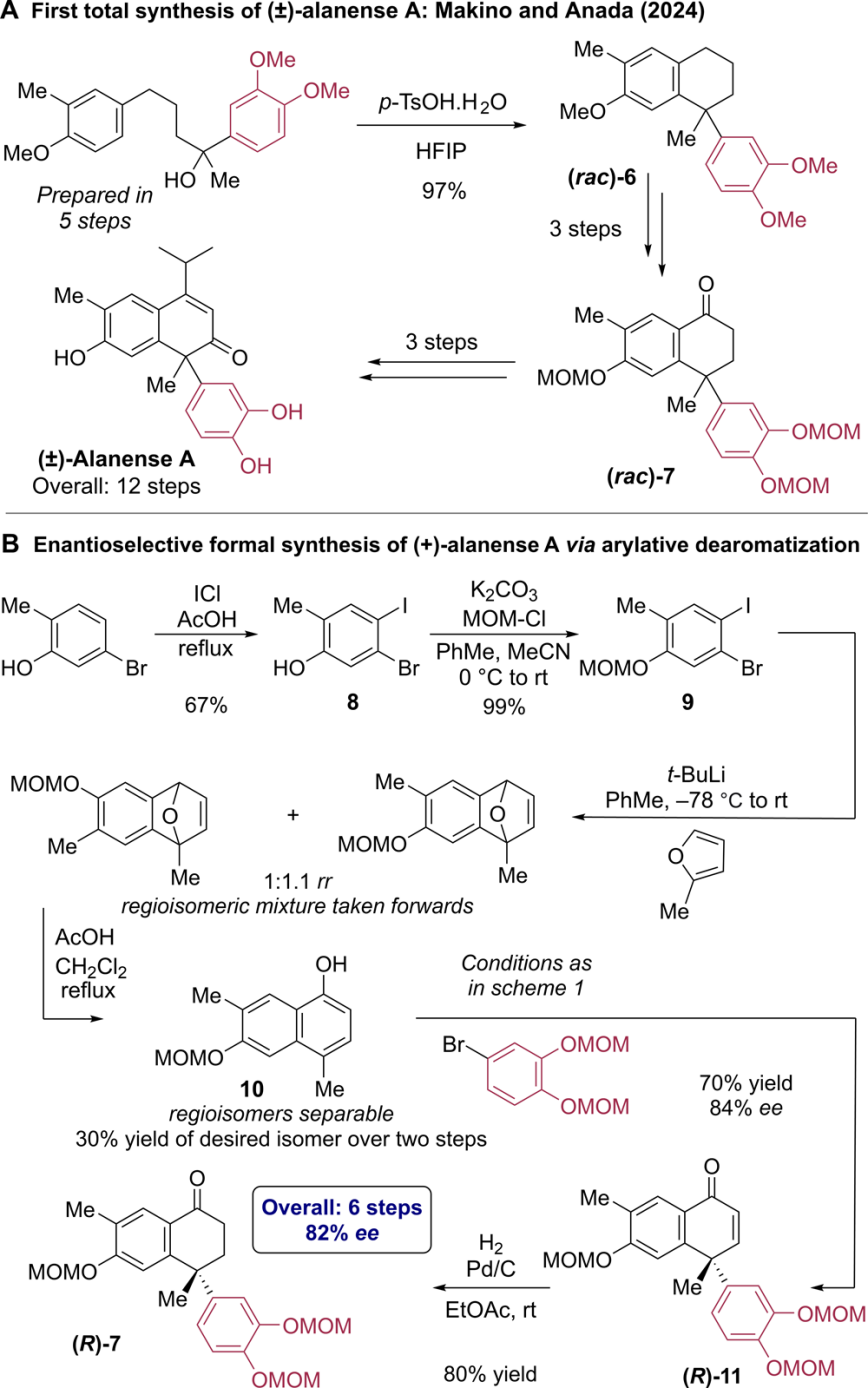
Application to Formal Enantioselective Synthesis of Alanense A

Alanense A is a cadinane sesquiterpenoid natural product first isolated as a racemate in 2022 by Cao and co-workers (Scheme 5A).^28^ In 2024, Makino, Anada, and co-workers reported the only total synthesis to date, forming the racemate in which it naturally occurs.^29^ Their key step formed the central ring via an intramolecular dehydrative Friedel–Crafts alkylation to give *(rac)*-**6**. Three further steps replaced three methoxy substituents for MOM-protected phenols while oxidizing the unsubstituted benzylic position to afford ketone (*rac*)-**7**. This was converted into (±)-alanense A in three further steps, constituting a 12-step racemic synthesis. We envisaged that a concise, enantioselective synthesis of intermediate **7** would be feasible using our methodology, which would thereby constitute an enantioselective formal synthesis of alanense A. Iodination of 5-bromo-2-methylphenol delivered phenol **8** which was MOM-protected to afford benzyne precursor **9** (Scheme 5B). Formation of the corresponding benzyne with *t*-BuLi preceded [4 + 2]-cycloaddition with 2-methylfuran to deliver a mixture of regioisomeric 7-oxabenzonorbornadienes. Typical methods to promote the ring opening of these structures rely on either a strong Brønsted acid (such as HCl) or a Lewis acid (such as Cu(OTf)_2_), both of which were found to be incompatible with the MOM protecting group (for full details, see the Supporting Information).^30^ Pleasingly, we found that refluxing with acetic acid in dichloromethane successfully promoted the desired isomerization while being sufficiently mild to leave the MOM-protected phenol intact. At this point, the desired naphthol isomer **10** was separable from its undesired regioisomer.^31^ The key enantioselective arylative dearomatization delivered enone **11** in 70% yield and 84% *ee*, and this was converted to the intermediate in the prior synthesis, **7**, by hydrogenation with Pd/C. This common intermediate was accessed in a highly enantioenriched form in only six steps, compared with the nine steps to form the racemate in the previous synthesis.

**Scheme 6:**
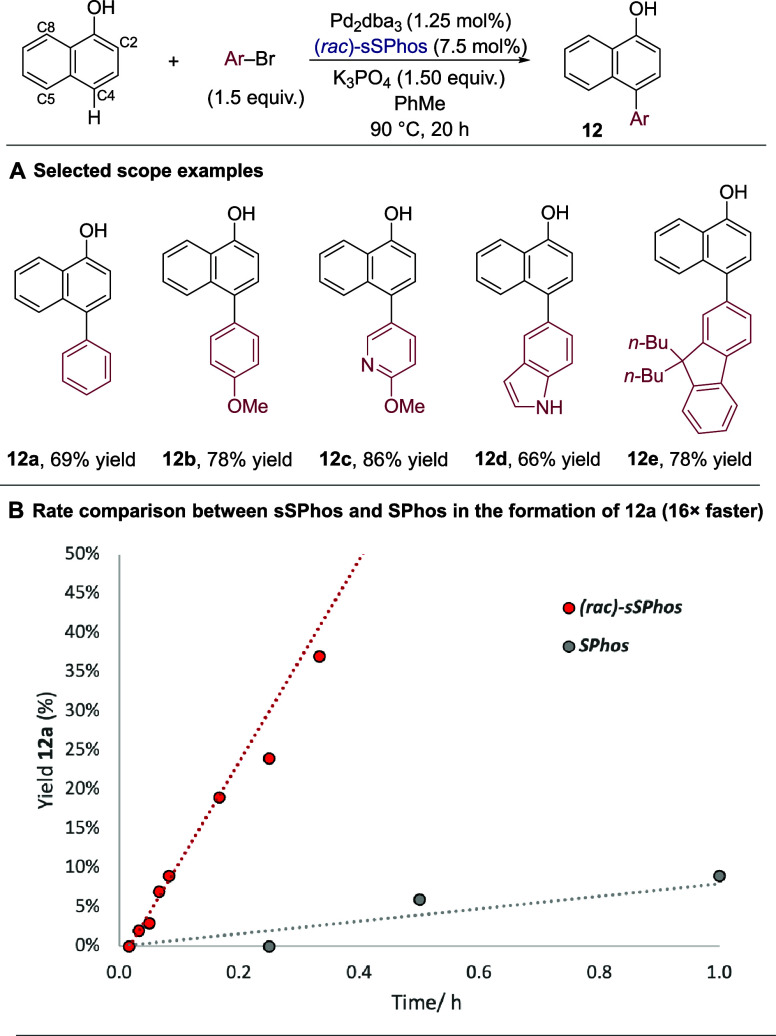
Direct Arylation of 1-Naphthols Bearing No *Para* Substituent

According to our mechanistic hypothesis, one might expect that if the alkyl substituent at the C4 position of the naphthol were removed, then rearomatization would occur, constituting a C4-selective C–H arylation of naphthalen-1-ol. To the best of our knowledge, such a transformation has generally been reported using specific electrophiles such as quinone monoacetals,^32^*ortho*-nitrobromoarenes,^33^ or highly electron-rich phenols via sulfoxide intermediates.^34^ More general aryl halides have been used as electrophiles for C–H arylation of 1-naphthols, but these give rise to the C2 product rather than C4, most likely due to proximity to the oxygen in those particular mechanisms.^35^ You and co-workers in their 2016 report showed that 2-methylnaphthalen-1-ol gave a 1:1 ratio of C4 arylation and diarylation resulting from reaction at C4 and C5 or C8.^10a^ We found C4-selective direct arylation to work very effectively with our system on simple naphthalen-1-ol and demonstrate this for several aryl bromides (Scheme 6A, **12a**–**12e**). This was a highly chemoselective transformation, with no competitive *O*-arylation visible, providing a very concise route to these compounds, which have previously been accessed through more lengthy cross-coupling sequences involving protecting group exchange.^36^ SPhos also gave similarly high selectivity outcomes but was a far less reactive ligand than sSPhos; we compared rates using both ligands for the reaction to form arylated product **12a** and observed a 16-fold rate enhancement for sSPhos when compared to SPhos (Scheme 6B). This provides further evidence for the attractive interaction between the ligand and substrate when sSPhos is used. Preliminary attempts to form very hindered biaryl bonds and therefore introduce axial chirality using this approach were unsuccessful (see the Supporting Information for details).

## 3 Conclusions

In summary, we have developed a highly enantioselective intermolecular arylative dearomatization of 1-naphthols. This constitutes a rare case of palladium-catalyzed arylative dearomatization being carried out asymmetrically in an intermolecular manner (i.e., not as part of a cyclization). We demonstrate applications of this method in the synthesis of medicinally relevant compounds and in the enantioselective formal synthesis of a natural product. Evidence is provided to support our hypothesis that the sulfonate group on the sSPhos ligand is playing a key role in assembling the reaction components through attractive noncovalent interactions. We anticipate that demonstration of this principle will lead to further enantioselective variants of this important reaction type.

## Supporting Information Available

Additional optimization, full experimental details, and characterization data for compounds (PDF)

## References

[ref1] aPouységu L.; Deffieux D.; Quideau S. Hypervalent iodine-mediated phenol dearomatization in natural product synthesis. Tetrahedron 2010, 66, 2235–2261. 10.1016/j.tet.2009.12.046.

[ref2] Zhang W.-W.; Feng Z.; You S.-L.; Zheng C. Electrophile–Arene Affinity: An Energy Scale for Evaluating the Thermodynamics of Electrophilic Dearomatization Reactions. J. Org. Chem. 2024, 89, 11487–11501. 10.1021/acs.joc.4c01168. 39077910

[ref3] aAn J.; Bandini M. Recent Advances in the Catalytic Dearomatization of Naphthols. Eur. J. Org. Chem. 2020, 2020, 4087–4097. 10.1002/ejoc.202000107.

[ref4] aDouglas C. J.; Overman L. E. Catalytic asymmetric synthesis of all-carbon quaternary stereocenters. Proc. Natl. Acad. Sci. U.S.A. 2004, 101, 5363–5367. 10.1073/pnas.0307113101. 14724294 PMC397386

[ref5] Morgan J.; Pinhey J. T. Mechanism of arylation of nucleophiles by aryllead triacetates. Part 1. Exclusion of a pathway involving aryl free radicals. J. Chem. Soc., Perkin Trans. 1993, 1, 1673–1676. 10.1039/p19930001673.

[ref6] aBarton D. H. R.; Blazejewski J.-C.; Charpiot B.; Lester D. J.; Motherwell W. B.; Papoula M. T. B. Comparative arylation reactions with pentaphenylbismuth and with triphenylbismuth carbonate. J. Chem. Soc., Chem. Commun. 1980, 827–829. 10.1039/c39800000827.

[ref7] Ozanne-Beaudenon A.; Quideau S. Regioselective Hypervalent-Iodine(III)-Mediated Dearomatizing Phenylation of Phenols through Direct Ligand Coupling. Angew. Chem., Int. Ed. 2005, 44, 7065–7069. 10.1002/anie.200501638. 16208727

[ref8] aGarcía-Fortanet J.; Kessler F.; Buchwald S. L. Palladium-Catalyzed Asymmetric Dearomatization of Naphthalene Derivatives. J. Am. Chem. Soc. 2009, 131, 6676–6677. 10.1021/ja9025193. 19388652 PMC2748791

[ref9] aXu R.-Q.; Gu Q.; Wu W.-T.; Zhao Z.-A.; You S.-L. Construction of Erythrinane Skeleton via Pd(0)-Catalyzed Intramolecular Dearomatization of para-Aminophenols. J. Am. Chem. Soc. 2014, 136, 15469–15472. 10.1021/ja508645j. 25308898

[ref10] aXu R. Q.; Yang P.; Tu H. F.; Wang S. G.; You S. L. Palladium(0)-Catalyzed Intermolecular Arylative Dearomatization of beta-Naphthols. Angew. Chem., Int. Ed. 2016, 55, 15137–15141. 10.1002/anie.201608724. 27791314

[ref11] Li J.; Peng H.; Wang F.; Wang X.; Jiang H.; Yin B. 2,5-Oxyarylation of Furans: Synthesis of Spiroacetals via Palladium-Catalyzed Aerobic Oxidative Coupling of Boronic Acids with α-Hydroxyalkylfurans. Org. Lett. 2016, 18, 3226–3229. 10.1021/acs.orglett.6b01472. 27310764

[ref12] Yamaguchi M.; Suzuki K.; Sato Y.; Manabe K. Palladium-Catalyzed Direct C3-Selective Arylation of N-Unsubstituted Indoles with Aryl Chlorides and Triflates. Org. Lett. 2017, 19, 5388–5391. 10.1021/acs.orglett.7b02669. 28898099

[ref13] aPolák P.; Tobrman T. Dearomatization Strategy for the Synthesis of Arylated 2H-Pyrroles and 2,3,5-Trisubstituted 1H-Pyrroles. Org. Lett. 2017, 19, 4608–4611. 10.1021/acs.orglett.7b02219. 28805398

[ref14] aKieffer M. E.; Chuang K. V.; Reisman S. E. A copper-catalyzed arylation of tryptamines for the direct synthesis of aryl pyrroloindolines. Chem. Sci. 2012, 3, 3170–3174. 10.1039/c2sc20914d. 23105962 PMC3480223

[ref15] Zhu S.; MacMillan D. W. Enantioselective copper-catalyzed construction of aryl pyrroloindolines via an arylation-cyclization cascade. J. Am. Chem. Soc. 2012, 134, 10815–10818. 10.1021/ja305100g. 22716914 PMC3392034

[ref16] aZhang Y.-C.; Zhao J.-J.; Jiang F.; Sun S.-B.; Shi F. Organocatalytic Asymmetric Arylative Dearomatization of 2,3-Disubstituted Indoles Enabled by Tandem Reactions. Angew. Chem., Int. Ed. 2014, 53, 13912–13915. 10.1002/anie.201408551. 25303741

[ref17] Anderson K. W.; Buchwald S. L. General Catalysts for the Suzuki–Miyaura and Sonogashira Coupling Reactions of Aryl Chlorides and for the Coupling of Challenging Substrate Combinations in Water. Angew. Chem., Int. Ed. 2005, 44, 6173–6177. 10.1002/anie.200502017. 16097019

[ref18] Pearce-Higgins R.; Hogenhout L. N.; Docherty P. J.; Whalley D. M.; Chuentragool P.; Lee N.; Lam N. Y. S.; McGuire T. M.; Valette D.; Phipps R. J. An Enantioselective Suzuki–Miyaura Coupling To Form Axially Chiral Biphenols. J. Am. Chem. Soc. 2022, 144, 15026–15032. 10.1021/jacs.2c06529. 35969692 PMC9434994

[ref19] aGolding W. A.; Pearce-Higgins R.; Phipps R. J. Site-Selective Cross-Coupling of Remote Chlorides Enabled by Electrostatically Directed Palladium Catalysis. J. Am. Chem. Soc. 2018, 140, 13570–13574. 10.1021/jacs.8b08686. 30295472

[ref20] The reason for reduced *ee* using K_2_CO_3_ is unclear but could be related to the poor solubility of this base in toluene.

[ref21] For complete optimization data, and an investigation of other aryl halides, see the Supporting Information.

[ref22] aSatoh T.; Miura M. Catalytic Direct Arylation of Heteroaromatic Compounds. Chem. Lett. 2007, 36, 200–205. 10.1246/cl.2007.200.

[ref23] For additional unsuccessful aryl bromides, see the Supporting Information.

[ref24] Khouzam H. R.; Emes R.; Gill T.; Raroque R. The antidepressant sertraline: A review of its uses in a range of psychiatric and medical conditions. Comprehensive Therapy 2003, 29, 47–53. 10.1007/s12019-003-0007-6. 12701343

[ref25] Fuller R. W.; Hemrick-Luecke S. K.; Littlefield E. S.; Audia J. E. Comparison of desmethylsertraline with sertraline as a monoamine uptake inhibitor in vivo. Prog. Neuro-Psychopharmacol. Biol. Psychiatry 1995, 19, 135–149. 10.1016/0278-5846(94)00110-4. 7535937

[ref26] Koblan K. S.; Hopkins S. C.; Sarma K.; Jin F.; Goldman R.; Kollins S. H.; Loebel A. Dasotraline for the Treatment of Attention-Deficit/Hyperactivity Disorder: A Randomized, Double-Blind, Placebo-Controlled, Proof-of-Concept Trial in Adults. Neuropsychopharmacology 2015, 40, 2745–2752. 10.1038/npp.2015.124. 25948101 PMC4864650

[ref27] aLautens M.; Rovis T. Selective functionalization of 1,2-dihydronaphthalenols leads to a concise, stereoselective synthesis of sertraline. Tetrahedron 1999, 55, 8967–8976. 10.1016/S0040-4020(99)00456-1.

[ref28] Zhang C.-L.; Liu J.; Xi C.-C.; Cao Y.-G.; He J.; Li S.-C.; Zhang F.; Naman C. B.; Cao Z.-Y. Cadinane Sesquiterpenoids and Their Glycosides from Alangium chinense That Inhibit Spontaneous Calcium Oscillations. J. Nat. Prod. 2022, 85, 599–606. 10.1021/acs.jnatprod.1c00978. 34957832

[ref29] Makino K.; Fukuda R.; Sueki S.; Anada M. Total Synthesis of Alanense A through an Intramolecular Friedel–Crafts Alkylation. J. Org. Chem. 2024, 89, 2050–2054. 10.1021/acs.joc.3c02481. 38241043

[ref30] aWolthuis E.; Bossenbroek B.; DeWall G.; Geels E.; Leegwater A. Reactions of Methyl-substituted 1,4-Epoxy-1,4-dihydronaphthalenes. J. Org. Chem. 1963, 28, 148–152. 10.1021/jo01036a034.

[ref31] The undesired regiosomer is included in the substrate scope to give product **2h**.

[ref32] Kamitanaka T.; Morimoto K.; Tsuboshima K.; Koseki D.; Takamuro H.; Dohi T.; Kita Y. Efficient Coupling Reaction of Quinone Monoacetal with Phenols Leading to Phenol Biaryls. Angew. Chem., Int. Ed. 2016, 55, 15535–15538. 10.1002/anie.201608013. 27860031

[ref33] Kumar A.; Yadav A.; Verma A.; Jana S.; Sattar M.; Kumar S.; Prasad C. D.; Kumar S. Chemoselective arylation of phenols with bromo-nitroarenes: synthesis of nitro-biaryl-ols and their conversion into benzofurans and carbazoles. Chem. Commun. 2014, 50, 9481–9484. 10.1039/C4CC03090G. 25007753

[ref34] He Z.; Perry G. J. P.; Procter D. J. Sulfoxide-mediated oxidative cross-coupling of phenols. Chem. Sci. 2020, 11, 2001–2005. 10.1039/C9SC05668H. 34123295 PMC8150100

[ref35] aBedford R. B.; Coles S. J.; Hursthouse M. B.; Limmert M. E. The Catalytic Intermolecular Orthoarylation of Phenols. Angew. Chem., Int. Ed. 2003, 42, 112–114. 10.1002/anie.200390037. 19757606

[ref36] aYang L.; Zheng H.; Luo L.; Nan J.; Liu J.; Wang Y.; Luan X. Palladium-Catalyzed Dynamic Kinetic Asymmetric Transformation of Racemic Biaryls: Axial-to-Central Chirality Transfer. J. Am. Chem. Soc. 2015, 137, 4876–4879. 10.1021/jacs.5b01285. 25851252

