# Supplementary material for: Intermolecular Asymmetric Arylative Dearomatization of 1-Naphthols
Source: J Am Chem Soc. 2024 Dec 4;146(50):34970–8. doi: 10.1021/jacs.4c14754 (PMC11664591; doi:10.1021/jacs.4c14754)

Supporting Information

Intermolecular Asymmetric Arylative Dearomatization of 1-Naphthols

Max Kadarauch,[a] Thomas A. Moss,[b] and Robert J. Phipps[a]*

[a] Yusuf Hamied Department of Chemistry, University of Cambridge, Lensfield Road, Cambridge, CB2 1EW, United Kingdom.

[b] Oncology R&D, AstraZeneca, Cambridge CB4 0WG, United Kingdom

Table of Contents

General Experimental 3

Extended Reaction Optimization Data 5
Investigation of Solvents with KOH as the Base 5
Investigation of Palladium Pre-catalysts with KOH as the Base 5
Investigation of Bases 5
Investigation of Reaction Time 6
Investigation of Concentration 7

Aryl (Pseudo)Halides Investigation 7

Unsuccessful Substrates 7
Unsuccessful Nucleophiles 8
Unsuccessful Electrophiles 10

Determination of Product Absolute Stereochemistry 11

Experiment to Determine Naphthol Starting Material Stability under Reaction Conditions 11

Time Course Investigation 12
Dearomatization with sSPhos 12
Dearomatization with SPhos 14
Direct Arylation with sSPhos 15
Direct Arylation with SPhos 17
Comparison of Initial Rates 19

Synthesis of Starting Materials in Scheme 1 19
General Procedure A: Reaction of 4-Bromo-1-naphthol with Alkyl Iodides 19

Characterization of Products in Scheme 1 28
General Procedure B: Palladium/(*R*)-sSPhos-Catalyzed Dearomatization of Naphthols with Aryl Bromides 28

Characterization of Products in Scheme 2 60

Characterization of Unsuccessful 2-Naphthol Substrate 63

Characterization of Post-functionalization Products 64
Post-functionalization of Enone 2a 64
Enantioselective Synthesis of Quaternized Sertraline Variants 67

Enantioselective Formal Synthesis of Alanense A 76

Direct Arylation of Naphthalen-1-ol 86

Unsuccessful Preliminary Investigations into Direct Arylation to form Axially Chiral Biaryls 90

Investigation of Intramolecular 2-Naphthol Substrate 90

Synthesis of Unsuccessful Starting Materials 92

References 96

General Experimental

Solvents: THF, MeOH, CH_2Cl_2, and PhMe were purified by distillation on site under inert atmosphere *via* the following processes. THF was pre-dried over sodium wire and then distilled from calcium hydride and lithium aluminum hydride. MeOH, CH_2Cl_2, and PhMe were distilled from calcium hydride.

Reagents: All reagents were used as supplied from commercial sources with no further purification. (*R*)-sSPhos and (*S*)-sSPhos was prepared by resolution of (*rac*)-sSPhos by preparative SFC, according to our previous publication.[1] Alternatively, (*R*)-sSPhos can also be obtained *via* recrystallization of diastereomeric quinidine salts, according to our previous publication.[1] (*R*)-sSPhos-Np was prepared according to our previous publication.[2] Pd_2dba_3 was purchased from commercial sources but repurified prior to use, according the method described by Zalesskiy and Ananikov.[3]

Chromatography: Flash column chromatography was performed using silica gel 60A (40-63 μM) from Fluorochem. Crude compounds were dried directly onto silica gel and then loaded onto a pre-equilibrated silica column eluting with the solvent system specified under a positive pressure of air. Thin layer chromatography (TLC) was performed using 0.25 mm E. Merck silica plates (60F-254). The plates were visualized using ultraviolet radiation (254 nm) or a potassium permanganate stain where appropriate.

Reactions: Optimization experiments were carried out on a 0.10 mmol scale in 4 mL 13 mm crimp ring vials. For the scope examples, the reactions were carried out in either 4 mL crimp vials or 2–5 mL Biotage microwave vials. 1 and 2 mmol scale up experiments were carried out in 10–20 mL Biotage microwave vials.

Data collection: ^{1}H NMR spectra were recorded on 400 MHz QNP cryoprobe, 400 MHz AVIII HD smart probe, 400 MHz Advance III HD, 400 MHz Neo Prodigy, 500 MHz DCH Cryoprobe, 600 MHz Advance BBI and 700 MHz TXO Cryoprobe spectrometers. The chemical shifts, reported in parts per million (δ ppm) were recorded relative to the residual undeuterated solvent ($CDCl_3$: 7.26 ppm, MeOD: 3.31 ppm, DMSO-d_6: 2.50 ppm). Multiplicity is recorded as follows: s = singlet, d = doublet, t = triplet, q = quartet, pent = pentet, sext = sextet, m = multiplet, br = broad with associated combinations. Coupling constant (*J*, Hz) and peak integrations (nH) are also reported. ^{13}C NMR spectra were recorded on the same machines with complete proton decoupling. The chemical shifts, reported in parts per million (δ ppm) were recorded relative to

the residual undeuterated solvent ($CDCl_3$: 77.16 ppm, MeOD: 49.00 ppm, DMSO-d_6: 39.52). ^{19}F NMR spectra were recorded on 400 MHz QNP cryoprobe, 400 MHz AVIII HD smart probe and 400 MHz Advance III HD spectrometers.

Chiral SFC Analysis: Performed on a Waters ACQUITY UPC2 System with DAICEL CHIRALPAK IA, IC, IE, IG, IH, IJ or IK columns (4.6 × 250 mm, 3.0 μm) in a mixed solvent system of supercritical CO_2 and MeOH. Some data was also obtained on a Waters ACQUITY UPC2 System with CHIRALPAK IA, IF, AD columns (3.0 × 100 mm, 3.0 μm) in either a mixed solvent system of supercritical CO_2 and 0.1% NH_3 in MeOH, or a mixed solvent system of supercritical CO_2 and 0.1% diethylamine (DEA) in isopropyl alcohol (IPA). A system backpressure of 138 bar was used for the 250 mm columns. A system backpressure of 120 bar was used for the 100 mm columns.

High Resolution Mass Spectrometry (HRMS): Recorded on a Waters Vion IMS QTOF or AGILENT 6230 LC/TOF at the Department of Chemistry at the University of Cambridge. The ionization method is noted as either positive or negative electrospray ionization (+/−ESI). Measured values are reported to 4 decimal places and are within ± 5 ppm of the calculated value. The calculated values are based on the most abundant isotope unless otherwise stated in the chemical formula.

Optical Rotations: measured in $CHCl_3$ on a Perkin Elmer 343 Polarimeter using a sodium lamp (λ = 589 nm, D-line). $[\alpha]_D$ values are reported at the stated temperature with concentration (c) in cg mL^{-1}.

X-Ray Crystallography: Data collection and analysis was performed by Dr Andrew Bond (University of Cambridge).

Racemic Reactions: Reactions to obtain racemic SFC traces were run with (*rac*)-sSPhos.

Extended Reaction Optimization Data

Investigation of Solvents with KOH as the Base

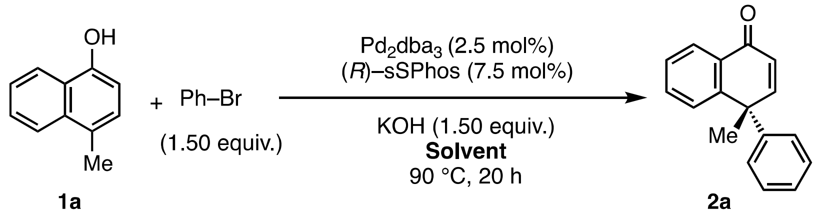

Entry	Solvent	Yield 2a/ %[a]	*ee*/ %[b]
**1**	**PhMe**	**49**	**99**
**2**	$PhCF_3$	51	98
**3**	1,4-Dioxane	6	N.D.

[a] Yields determined by ^{1}H NMR with reference to a dibromomethane internal standard. [b]*ee* determined by chiral SFC analysis of the crude reaction mixture.

Investigation of Palladium Pre-catalysts with KOH as the Base

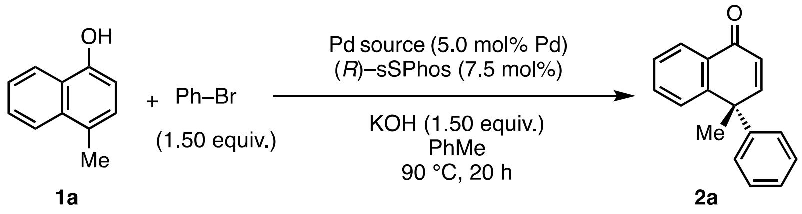

Entry	Pd source	Yield 2a/ %[a]	*ee*/ %[b]
**1**	**Pd_2dba_3**	**49**	**99**
**2**	$Pd(dba)_2$	39	99
**3**	$[PdCl(allyl)]_2$	19	N.D.
**4**	$Pd(OAc)_2$	11	N.D.
**5**	$PdCl_2(cod)$	9	N.D.
**6**	Pd/C (10% w/w)	trace	N.D.
**7**	$Pd(TFA)_2$	trace	N.D.
**8**	$PdCl_2$	trace	N.D.

[a] Yields determined by ^{1}H NMR with reference to a dibromomethane internal standard. [b]*ee* determined by chiral SFC analysis of the crude reaction mixture.

Investigation of Bases

Full data for the base screen in Table 1 (entries 3-7) are presented below.

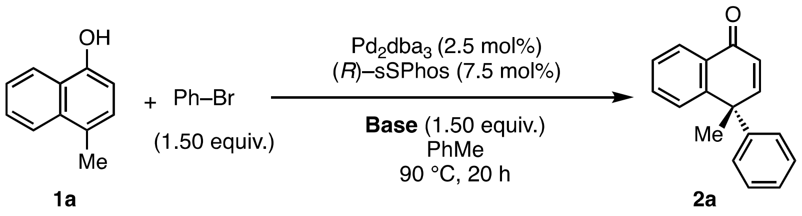

Entry	Base	Yield 2a/ %[a]	*ee*/ %[b]
**1**	K_2CO_3	42	83
**2**	Cs_2CO_3	35	96
**3**	Na_3PO_4	trace	N.D.
**4**	**K_3PO_4**	**61**	**95**
**5**	K_3PO_4 (3.00 equiv.)	53	98
**6**	LiOH	-	-
**7**	NaOH	54	99
**8**	KOH	49	99
**8**	CsOH.H_2O	58	95
**9**	NaOPh	40	99
**10**	^{t}AmONa	trace	N.D.

[a] Yields determined by ^{1}H NMR with reference to a dibromomethane internal standard. [b]*ee* determined by chiral SFC analysis of the crude reaction mixture.

Investigation of Reaction Time

A reaction time of 4 h resulted in near complete consumption of starting material **1a** (entry 2). However, to enable the full conversion of less reactive substrates, a reaction time of 20 h was judged to be optimal when investigating the scope of the reaction.

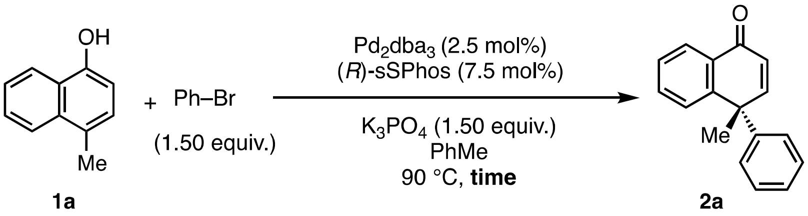

Entry	Time	Yield 2a/ %[a]	SM 1a/ %[a]	*ee*/ %[b]
**1**	20 h	61	-	95
**2**	4 h	60	7	97

[a] Yields determined by ^{1}H NMR with reference to a dibromomethane internal standard. [b]*ee* determined by chiral SFC analysis of the crude reaction mixture.

[N.B. The similar yields and enantioselectivities after 4 and 20 h was also taken as evidence that the product was stable under the reaction conditions (see **Experiment to Determine Naphthol**

Starting Material Stability under Reaction Conditions for the corresponding analysis of the naphthol substrate).]

[N.B. For more detailed analysis of *reactivity* over time, see **Time Course Investigation**].

Investigation of Concentration

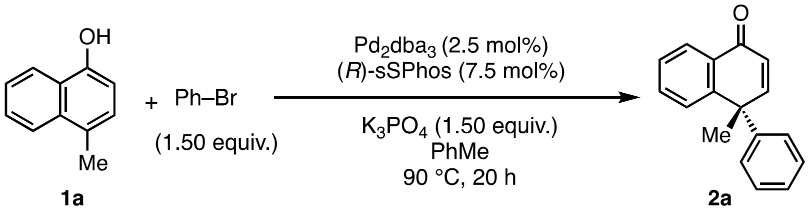

Entry	Concentration	Yield 2a/ %[a]	*ee*/ %[b]
**1**	0.1 M	55	95
**2**	**0.2 M**	**61**	**95**
**3**	0.4 M	49	95

[a] Yields determined by ^{1}H NMR with reference to a dibromomethane internal standard. [b] *ee* determined by chiral SFC analysis of the crude reaction mixture.

Aryl (Pseudo)Halides Investigation

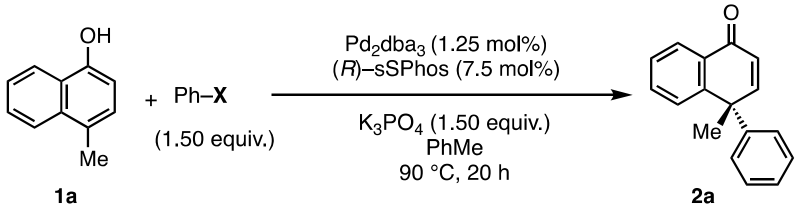

Entry	X	Yield 2a/ %[a]	*ee*/ %[b]
**1**	Cl	trace	N.D.
**2**	**Br**	**62 (63)**	**97 (97)**
**3**	I	32	98
**4**	OTf	15	98

[a] Yields determined by ^{1}H NMR with reference to a dibromomethane internal standard. [b]*ee* determined by chiral SFC analysis of the crude reaction mixture. Values in parentheses correspond to the isolated sample.

Unsuccessful Substrates

Unsuccessful Nucleophiles

Details of the attempted formation of enone **2ah** and formation of enone **S1**, described in the manuscript, are presented below. Additional unsuccessful examples are also displayed.

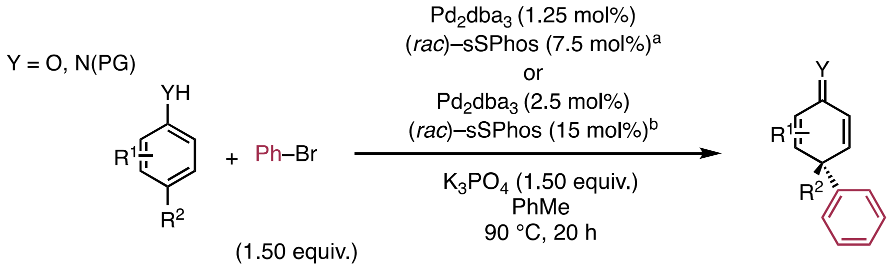

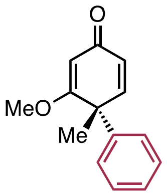

2ah,[b,c,d] no product detected
57% starting material detected

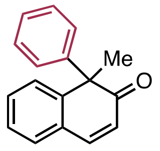

S1,[a] 10% yield[e] (16% NMR yield)
15% *ee*

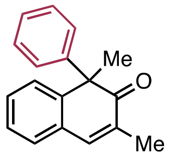

S2,[a] trace product detected
50% starting material detected

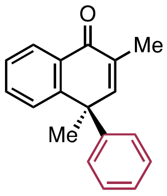

S3,[a] no product detected
47% starting material detected

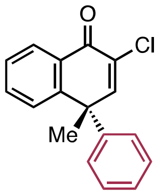

S4,[a] no product detected
no starting material detected

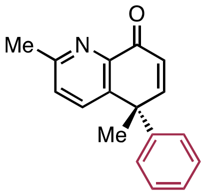

S5,[a] no product detected
62% starting material detected

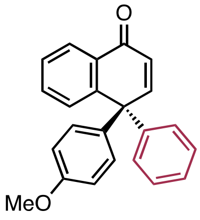

S6,[b,c,d] no product detected
57% starting material detected

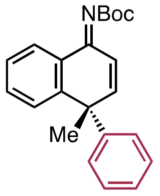

S7,[a] no product detected
19% starting material detected

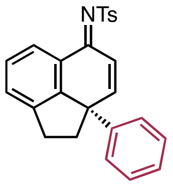

S8,[b] no product detected
no starting material detected

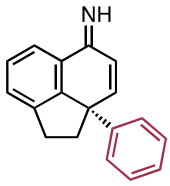

S9,[b] no product detected
52% starting material detected

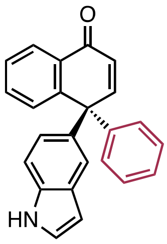

S10,[a] no product detected
25% starting material detected

Yields determined by ^{1}H NMR with reference to a dibromomethane internal standard. Detected starting material refers to phenol or naphthol substrate. [c] Reaction conducted at 110 °C. [d] 48 h reaction. [e] Isolated yield. Reaction conducted with (*R*)-sSPhos

[N.B. For characterization data for product **S1**, see **Characterization of Unsuccessful 2-Naphthol Substrate**]

Unsuccessful Electrophiles

Details of the attempted formation of enones **2ai-2ak**, described in the manuscript, are presented below. Additional unsuccessful examples are also displayed.

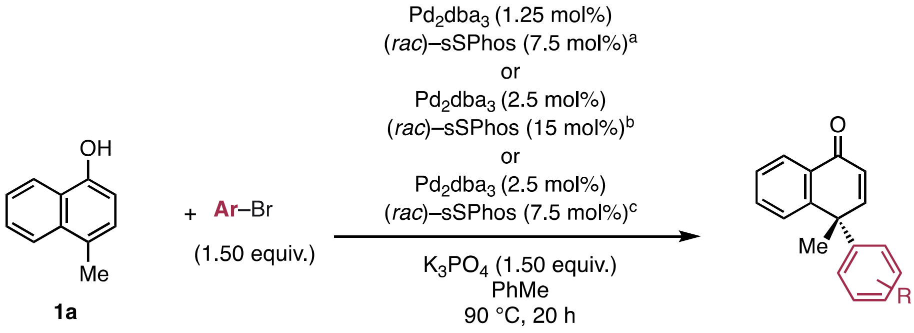

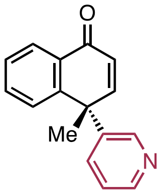

2ai,[c] no product detected
48% starting material detected

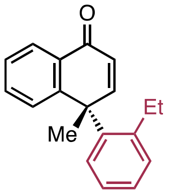

2aj,[a] trace product detected
46% starting material detected

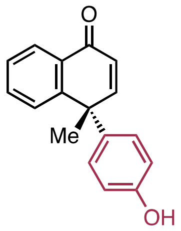

2ak,[a] no product detected
14% starting material detected

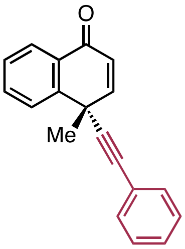

S11,[b] no product detected

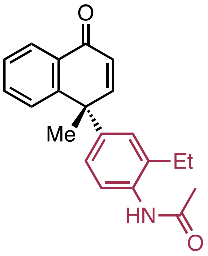

S12,[b] 22% NMR yield

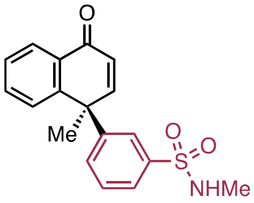

S13,[b,d] 11% NMR yield
50% starting material detected

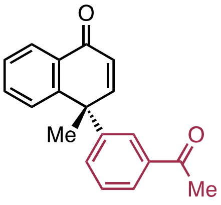

S14,[a] 10% NMR yield
45% starting material detected

Yields determined by ^1H NMR with reference to a dibromomethane internal standard. Recovered starting material refers to naphthol substrate.[d] 48 h reaction

Determination of Product Absolute Stereochemistry

The enantioenriched sample of enone **2r** was crystallized *via* vapor diffusion (40-60 °C petroleum ether/EtOAc).

Absolute stereochemistry of **2r** was determined as *R* by single crystal X-ray analysis, performed by Dr. Andrew Bond (deposited in the CCDC with deposition number 2389221). Absolute stereochemistry of the remaining products was assigned by analogy.

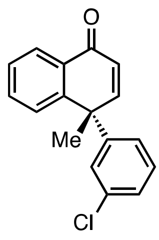

2r

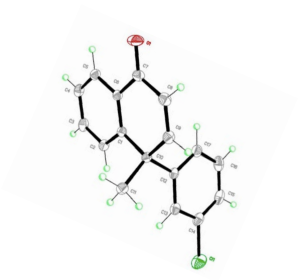

Following recrystallization, the enantioselectivity of the mother liquor was found to be 90% *ee*, lower than that of the original sample (95% *ee*), implying that the crystals had formed from the major enantiomer.

Experiment to Determine Naphthol Starting Material Stability under Reaction Conditions

For several examples in the substrate scope, including enone **2a**, it was observed that the naphthol starting material and product accounted for significantly less than 100% of the mass balance of the reaction. Additionally, no major side products were observed. In **Investigation of Reaction Time**, it was concluded that the product was stable under the reaction conditions. We therefore speculated whether decomposition of the naphthol starting material was responsible for the lost mass balance.

Naphthol starting material **1a** was therefore tested under the optimized conditions in the absence of an electrophile. 59% of the original starting material was observed, with no other significant byproducts. This indicates that naphthol **1a** is moderately unstable under the reaction conditions, explaining the lost mass balance of the reaction, at least to some degree.

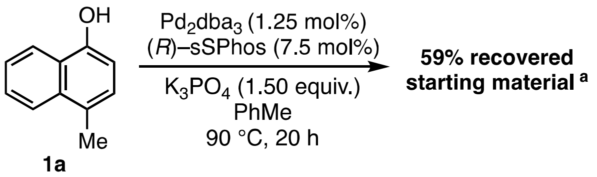

[a] Determined by ^{1}H NMR NMR of the crude reaction mixture with reference to a dibromomethane internal standard

The following procedure was followed:

A vial containing a magnetic stirrer bar was sequentially charged with the 4-methylnaphthalen-1-ol (15.8 mg, 0.100 mmol, 1.00 equiv.), (*R*)-sSPhos (3.8 mg, 0.0075 mmol, 7.5 mol%), K_3PO_4 (31.8 mg, 0.150 mmol, 1.50 equiv.), and Pd_2dba_3 (1.1 mg, 0.00125 mmol, 1.25 mol%). The vial was sealed, and evacuated and backfilled with N_2 (4×). PhMe (0.50 mL) was added under nitrogen. The reaction was stirred at 90 °C and 900 rpm for 20 h. Upon completion, the reaction mixture was filtered through celite, eluting with MeOH. The filtrate was concentrated under a stream of nitrogen. The crude reaction mixture was analyzed by ^{1}H NMR in MeOD with reference to a dibromomethane internal standard.

Time Course Investigation

[N.B. All time points correspond to individual runs.]

Dearomatization with sSPhos

A vial containing a magnetic stirrer bar was sequentially charged with 4-methylnaphthalen-1-ol (15.8 mg, 0.100 mmol, 1.00 equiv.), (*rac*)-sSPhos (3.8 mg, 0.0075 mmol, 7.5 mol%), K_3PO_4 (31.8 mg, 0.150 mmol, 1.50 equiv.), Pd_2dba_3 (1.1 mg, 0.00125 mmol, 1.25 mol%), and bromobenzene (23.6 mg, 0.150 mmol, 1.50 equiv.). The vial was sealed, and evacuated and backfilled with N_2 (4×). PhMe (0.50 mL) was added under nitrogen. The reaction was stirred at 90 °C and 900 rpm for the specified time. Upon completion, the reaction mixture was quickly cooled and filtered through celite, eluting with EtOAc. The filtrate was concentrated under a stream of air. The crude reaction mixture was analyzed by ^{1}H NMR in $CDCl_3$ with reference to a dibromomethane internal standard.

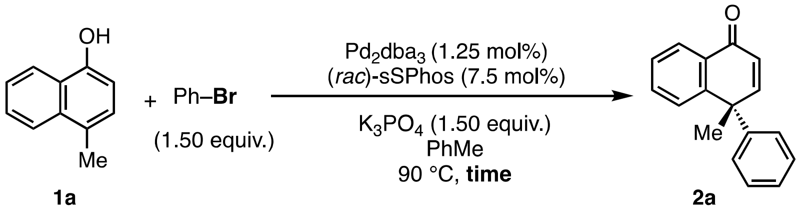

Time/ h	% Product 2a[a]	% SM 1a[a]
0.17	2	74
0.33	8	66
0.50	11	65
0.50	15	82
1.0	19	54
1.5	26	50
2.0	53	23
4.0	61	20
7.0	68	4
20	62	-

[a] Yields determined by ^{1}H NMR with reference to a dibromomethane internal standard.

Plot of % product 2a and % SM 1a over time

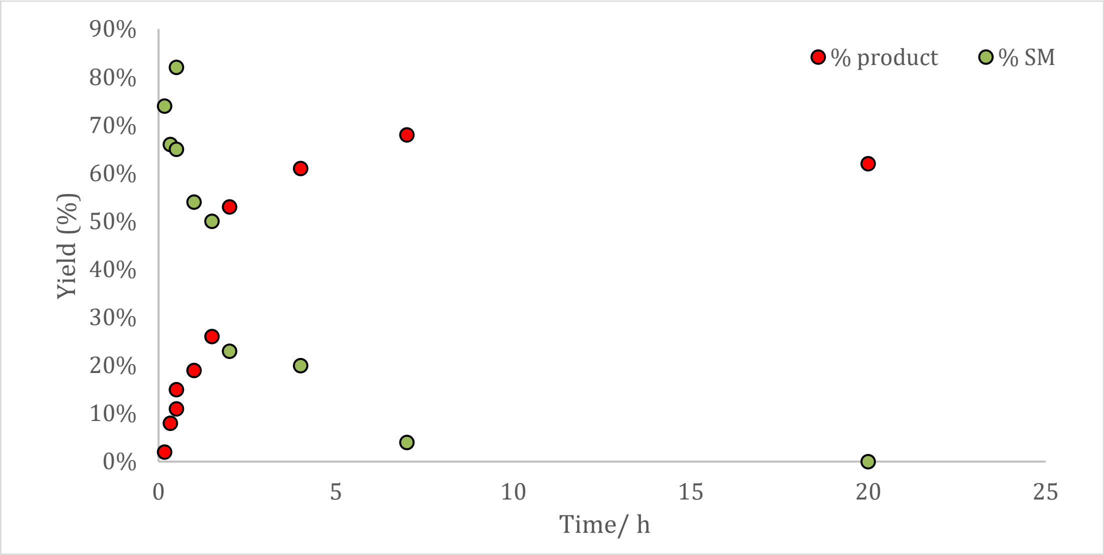

Plot of product 2a formation over time for calculation of initial rate

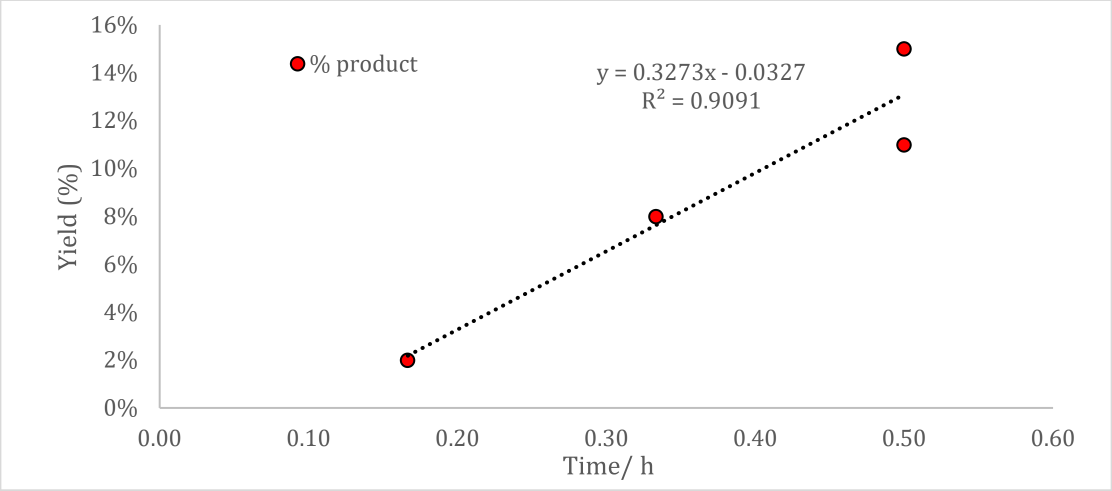

Dearomatization with SPhos

A vial containing a magnetic stirrer bar was sequentially charged with 4-methylnaphthalen-1-ol (15.8 mg, 0.100 mmol, 1.00 equiv.), SPhos (3.1 mg, 0.0075 mmol, 7.5 mol%), K_3PO_4 (31.8 mg, 0.150 mmol, 1.50 equiv.), Pd_2dba_3 (1.1 mg, 0.00125 mmol, 1.25 mol%), and bromobenzene (23.6 mg, 0.150 mmol, 1.50 equiv.). The vial was sealed, and evacuated and backfilled with N_2 (4×). PhMe (0.50 mL) was added under nitrogen. The reaction was stirred at 90 °C and 900 rpm for the specified time. Upon completion, the reaction mixture was quickly cooled and filtered through celite, eluting with EtOAc. The filtrate was concentrated under a stream of air. The crude reaction mixture was analyzed by ^1H NMR in $CDCl_3$ with reference to a dibromomethane internal standard.

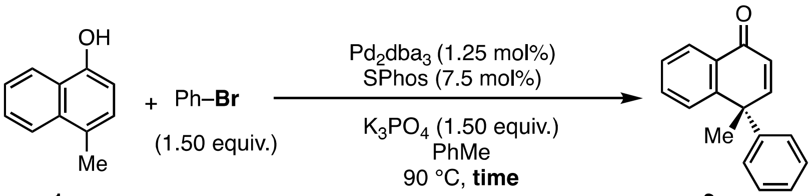

Time/ h	% Product 2a[a]	% SM 1a[a]
2.0	2	70
4.0	2	52
8.0	8	50
20	11	34
48	21	16

[a] Yields determined by ^1H NMR with reference to a dibromomethane internal standard.

Plot of % product 2a and % SM 1a over time

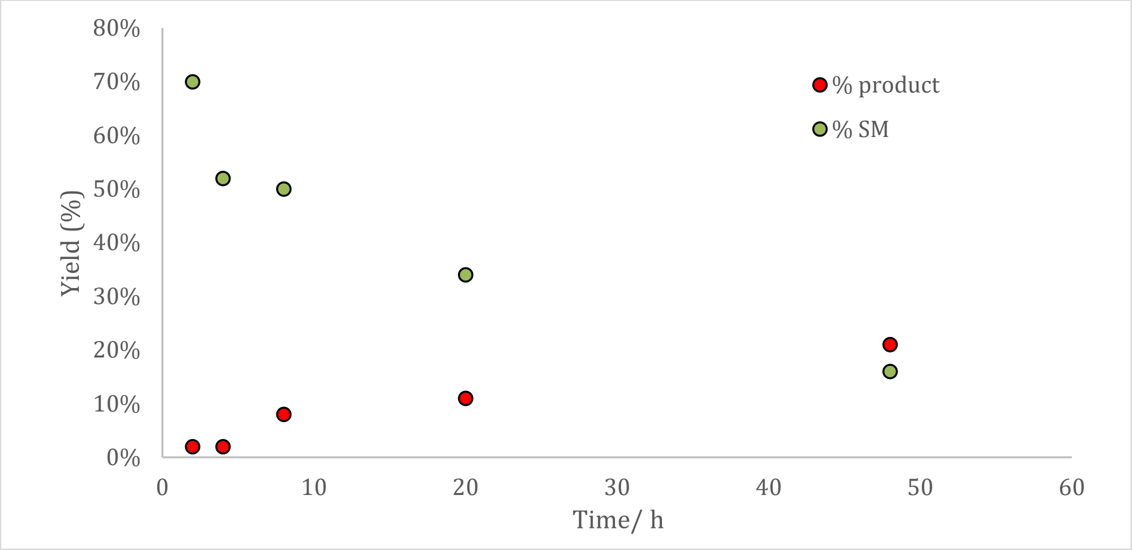

Plot of product 2a formation over time for calculation of initial rate

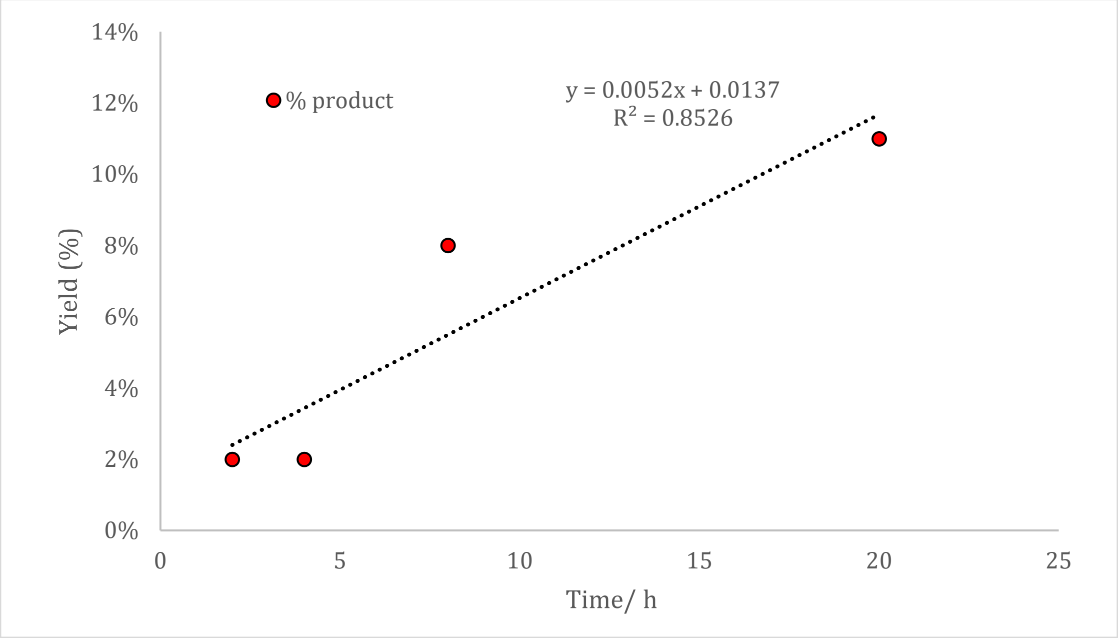

Direct Arylation with sSPhos

A vial containing a magnetic stirrer bar was sequentially charged with naphthalen-1-ol (14.4 mg, 0.100 mmol, 1.00 equiv.), (*rac*)-sSPhos (3.8 mg, 0.0075 mmol, 7.5 mol%), K_3PO_4 (31.8 mg, 0.150 mmol, 1.50 equiv.), Pd_2dba_3 (1.1 mg, 0.00125 mmol, 1.25 mol%), and bromobenzene (23.6 mg, 0.150 mmol, 1.50 equiv.). The vial was sealed, and evacuated and backfilled with N_2 (4×). PhMe (0.50 mL) was added under nitrogen. The reaction was stirred at 90 °C and 900 rpm for the specified time. Upon completion, the reaction mixture was quickly cooled and filtered through celite, eluting with EtOAc. The filtrate was concentrated under a stream of air. The crude reaction

mixture was analyzed by ^{1}H NMR in $CDCl_3$ with reference to a dibromomethane internal standard.

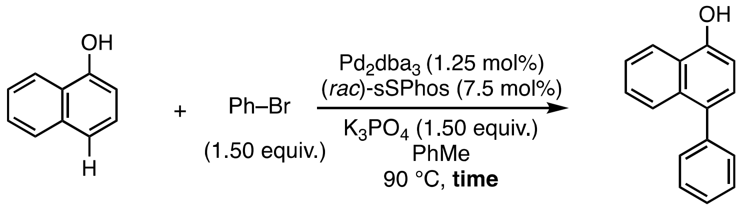

12a

Time/ h	% Product 12a[a]	% SM[a]
0.017	0	96
0.033	2	73
0.050	3	76
0.067	7	87
0.083	9	76
0.17	19	72
0.25	24	61
0.33	37	50
0.42	57	24
0.50	59	23
0.5	57	41
1.0	82	5
1.5	89	-
2.0	86	-
4.0	92	-

[a] Yields determined by ^{1}H NMR with reference to a dibromomethane internal standard.

Plot of % product 12a and % SM over time

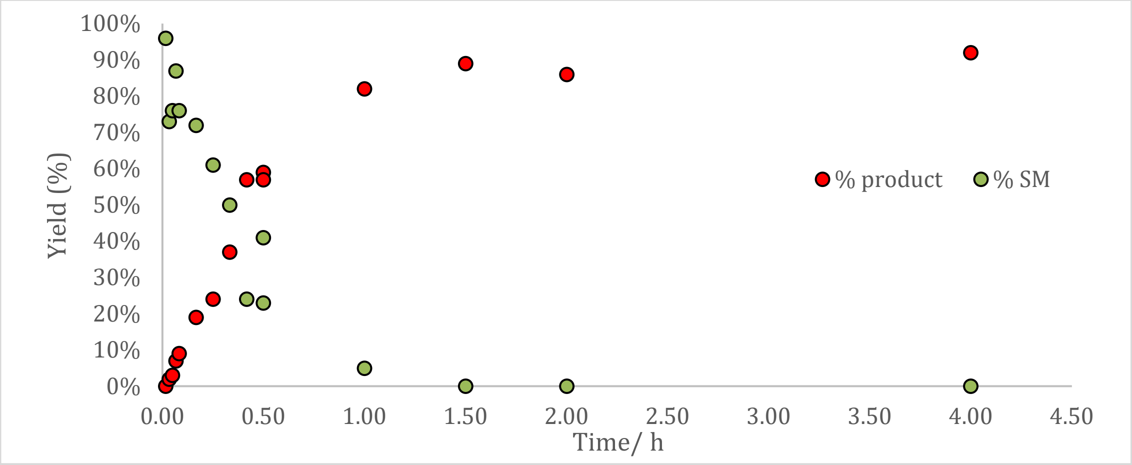

Plot of product 12a formation over time for calculation of initial rate

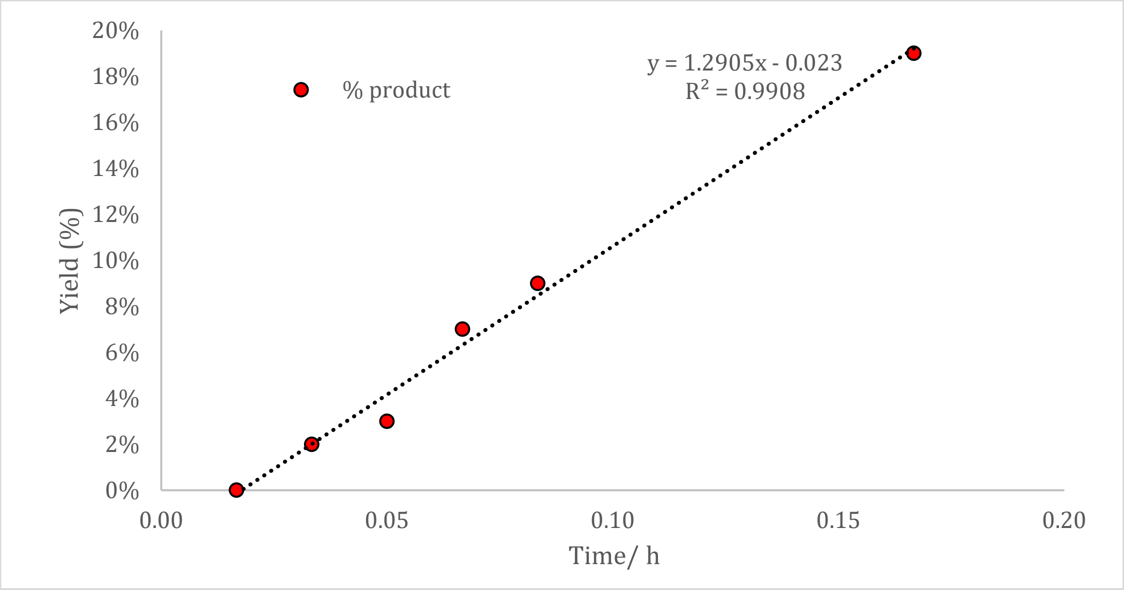

Direct Arylation with SPhos

A vial containing a magnetic stirrer bar was sequentially charged with naphthalen-1-ol (14.4 mg, 0.100 mmol, 1.00 equiv.), SPhos (3.1 mg, 0.0075 mmol, 7.5 mol%), K_3PO_4 (31.8 mg, 0.150 mmol, 1.50 equiv.), Pd_2dba_3 (1.1 mg, 0.00125 mmol, 1.25 mol%), and bromobenzene (23.6 mg, 0.150 mmol, 1.50 equiv.). The vial was sealed, and evacuated and backfilled with N_2 (4×). PhMe (0.50 mL) was added under nitrogen. The reaction was stirred at 90 °C and 900 rpm for the specified time. Upon completion, the reaction mixture was quickly cooled and filtered through celite, eluting with EtOAc. The filtrate was concentrated under a stream of air. The crude reaction mixture was analyzed by ^1H NMR in $CDCl_3$ with reference to a dibromomethane internal standard.

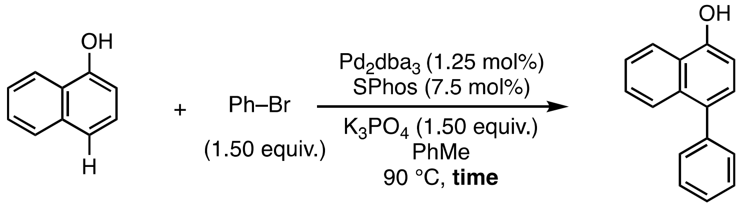

12a

Time/ h	% Product 12a[a]	% SM[a]
0.25	-	84%
0.50	6%	90%
1.0	9%	71%
1.5	11%	75%
2.0	20%	83%
4.0	47%	29%
20	43%	22%

[a] Yields determined by ^{1}H NMR with reference to a dibromomethane internal standard.

Plot of % product 12a and % SM over time

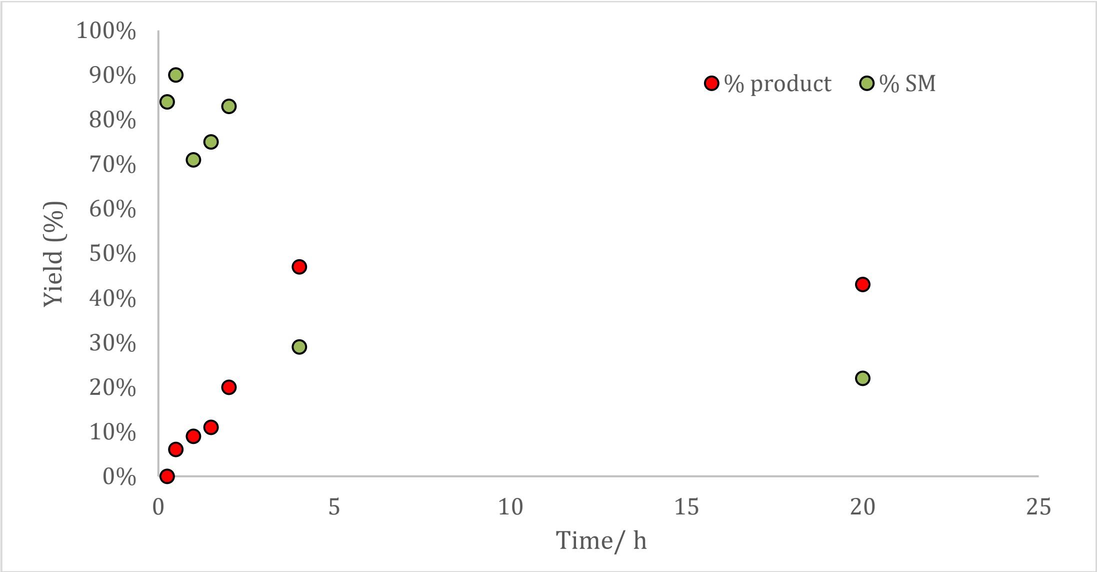

Plot of product 12a formation over time for calculation of initial rate

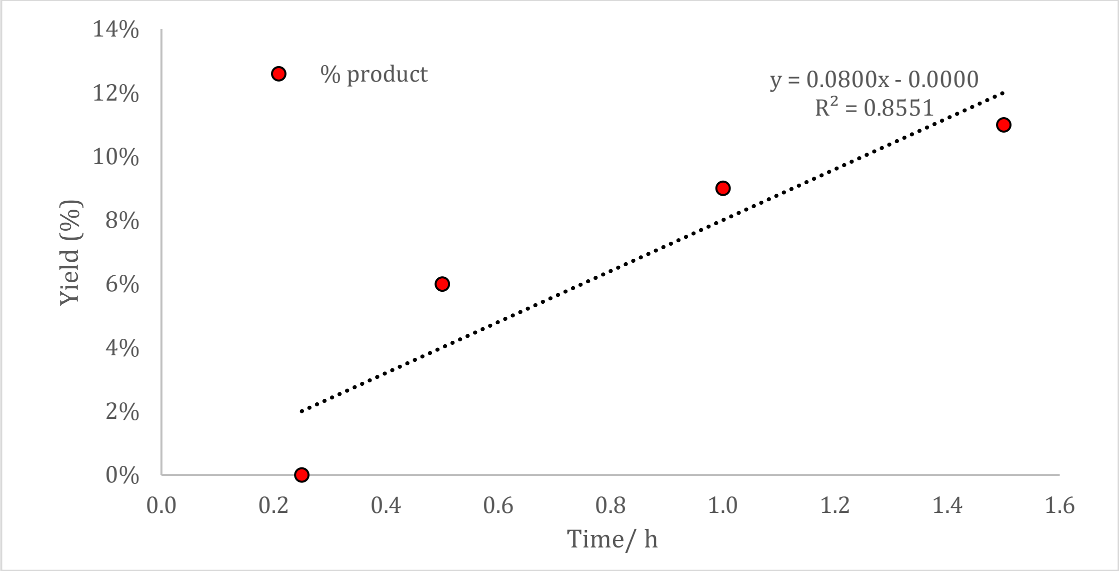

Comparison of Initial Rates

Reaction	Ligand	Initial rate (%/h)
Dearomatization	sSPhos	32.73
Dearomatization	SPhos	0.52
Direct Arylation	sSPhos	129.05
Direct Arylation	SPhos	8.00

Synthesis of Starting Materials in Scheme 1

General Procedure A: Reaction of 4-Bromo-1-naphthol with Alkyl Iodides

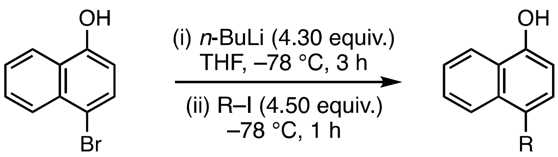

Adapted from the procedure reported by Kalek and co-workers.[4] Under vacuum, a heat gun-dried 2-necked round bottom flask containing a magnetic stirrer bar was charged with 4-bromo-1-naphthol (1.34 g, 6.00 mmol, 1.00 equiv.) and put under nitrogen. THF (50 mL) was added. The reaction was cooled to −78 °C, and *n*-BuLi (1.6 M, 16.3 mL, 26.0 mmol, 4.30 equiv.) was added dropwise. The reaction was stirred at −78 °C for 3 h. The appropriate alkyl iodide electrophile (27.0 mmol, 4.50 equiv.) was added dropwise, and the reaction was stirred for a further 1 h at

−78 °C. The reaction was quenched by the sequential addition of EtOH, H_2O, and 3 M HCl (aq.). The aqueous phase was extracted with CH_2Cl_2 (3×), dried over Na_2SO_4, concentrated under reduced pressure, and purified by flash column chromatography.

4-Ethylnaphthalen-1-ol (1b)

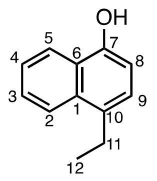

General Procedure A was performed with an ethyl iodide electrophile (4.21 g, 2.17 mL, 27.0 mmol, 4.50 equiv.). The crude product was purified by flash column chromatography (0-5% EtOAc in 40-60 °C petroleum ether, and 100% PhMe) to afford the title compound as a brown solid (283 mg, 1.64 mmol, 27% yield).

^{1}H NMR (400 MHz, $CDCl_3$) δ 8.24 (1H, d, *J*=8.1 Hz, H_5), 8.02 (1H, d, *J*=7.7 Hz, H_2), 7.61 – 7.43 (2H, m, H_3 and H_4), 7.17 (1H, d, *J*=7.6 Hz, H_9), 6.76 (1H, d, *J*=7.6 Hz, H_8), 5.17 (1H, br s, H_O), 3.05 (2H, q, *J*=7.6 Hz, H_{11}), 1.36 (3H, t, *J*=7.6 Hz, H_{12}).

^{13}C NMR (101 MHz, $CDCl_3$) δ 149.9, 133.0, 132.9, 126.4, 125.0, 124.8, 124.6, 124.0, 122.3, 108.4, 25.6, 15.3.

Data in agreement with the literature.[5]

4-Hexylnaphthalen-1-ol (1c)

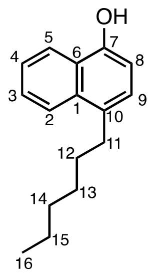

General Procedure A was performed with a hexyl iodide electrophile (5.73 g, 3.98 mL, 27.0 mmol, 4.50 equiv.). The crude product was purified by flash column chromatography (0-5% EtOAc in 40-60 °C petroleum ether) to afford the title compound as a brown solid (704 mg, 3.08 mmol, 51% yield).

^{1}H NMR (500 MHz, $CDCl_3$) δ 8.23 (1H, d, *J*=8.2 Hz, H_5), 8.01 (1H, d, *J*=8.3 Hz, H_2), 7.63 – 7.42 (2H, m, H_3 and H_4), 7.15 (1H, d, *J*=7.6 Hz, H_9), 6.74 (1H, d, *J*=7.6 Hz, H_8), 5.29 – 5.04 (1H, m, H_O), 2.99 (2H, t, *J*=7.9 Hz, H_{11}), 1.85 – 1.63 (2H, m, H_{12}), 1.50 – 1.39 (2H, m, H_{13}), 1.40 – 1.25 (4H, m, H_{14} and H_{15}), 0.95 – 0.84 (3H, m, H_{16}).

^{13}C NMR (126 MHz, $CDCl_3$) δ 149.9, 133.0, 131.7, 126.3, 125.6, 124.9, 124.9, 124.1, 122.3, 108.3, 32.8, 31.9, 31.1, 29.6, 22.8, 14.3.

Data in agreement with the literature.[4]

Synthesis of 4-Phenethylnaphthalen-1-ol (1d)

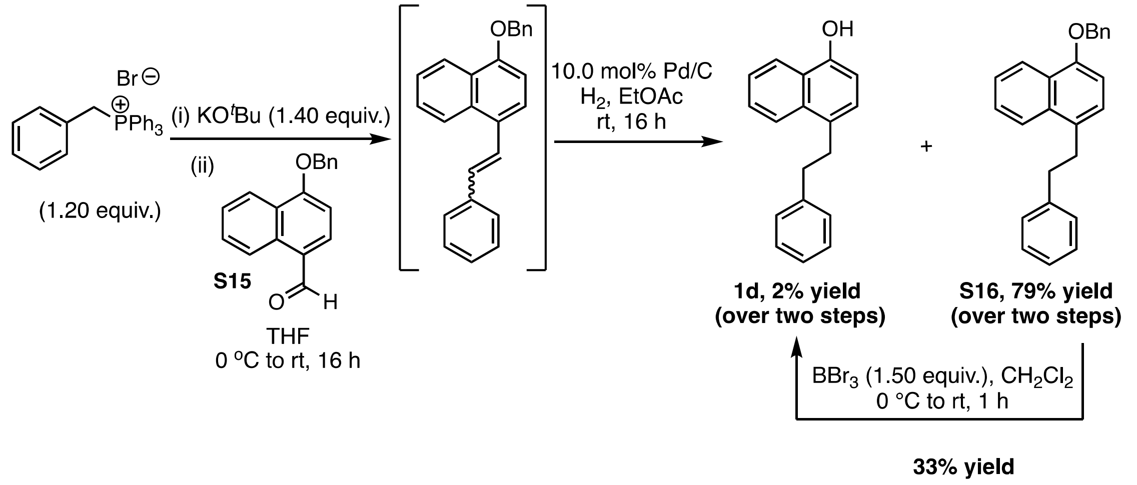

4-(benzyloxy)-1-Naphthaldehyde (**S15**) was prepared according to our previous publication.[2]

<u>Wittig Alkene Synthesis</u>: A solution of benzyltriphenylphosphonium bromide (2.60 g, 6.00 mmol, 1.20 equiv.) in THF (25 mL) was cooled to 0 °C. KO^{t}Bu (785 mg, 7.00 mmol, 1.40 equiv.) was added, and the reaction was stirred at 0 °C for 15 minutes. 4-(benzyloxy)-1-Naphthaldehyde (**S15**) (1.31 g, 5.00 mmol, 1.00 equiv.) was added, and the reaction was warmed to room temperature and stirred for 16 h. Upon completion, the reaction was quenched with water and the aqueous layer was extracted with EtOAc (3×). The combined organic extracts were washed with brine, dried over Na_2SO_4, and concentrated under reduced pressure. The crude alkene product was subjected to the next step without further purification.

<u>Alkene Hydrogenation</u>: To a solution of the crude alkene in EtOAc (50 mL) was added Pd/C (10 wt% loading Pd, 532 mg, 0.500 mmol, 10.0 mol% Pd). The flask was capped and evacuated and backfilled with hydrogen (3×). The reaction was stirred at room temperature under a hydrogen atmosphere for 16 h. Upon completion, the reaction was filtered through celite, eluting with

EtOAc, and concentrated under reduced pressure. The crude product was purified by flash column chromatography (0-20% EtOAc in heptane) to sequentially afford 1-(benzyloxy)-4-phenethylnaphthalene (**S16**) as a colorless oil (1.34 g, 3.96 mmol, 79% yield over two steps), and 4-phenethylnaphthalen-1-ol (**1d**) as an off-white solid (24.5 mg, 0.0987 mmol, 2% yield over two steps).

1-(benzyloxy)-4-Phenethylnaphthalene (**S16**) obtained following alkene hydrogenation could be converted to additional 4-phenethylnaphthalen-1-ol (**1d**) according to the following procedure:

A solution of 1-(benzyloxy)-4-phenethylnaphthalene (**S16**) (677 mg, 2.00 mmol, 1.00 equiv.) in CH_2Cl_2 (10 mL) was cooled to 0 °C under nitrogen. BBr_3 (1.00 M in CH_2Cl_2, 3.00 mL, 3.00 mmol, 1.50 equiv.) was added, and the reaction was stirred at 0 °C for 30 minutes. The reaction was warmed to room temperature and stirred for an additional 30 minutes. H_2O and CH_2Cl_2 were added, and the organic layer was separated. The aqueous layer was further extracted with CH_2Cl_2 (2×). The combined organic extracts were washed with sat. $NaHCO_3$ (aq.), dried over $MgSO_4$, filtered, concentrated under reduced pressure, and purified by flash column chromatography (0-30% EtOAc in heptane) to afford 4-phenethylnaphthalen-1-ol (**1d**) as a white solid (162 mg, 0.652 mmol, 33% yield).

1-(benzyloxy)-4-Phenethylnaphthalene (S16)

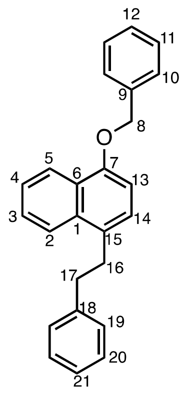

^{1}H NMR (500 MHz, $CDCl_3$) δ 8.45 (1H, dd, *J*=8.4, 1.4 Hz, H_5), 8.07 (1H, d, *J*=8.4 Hz, H_2), 7.61 – 7.50 (4H, m, H_3, H_4 and H_{10}), 7.47 – 7.41 (2H, m, H_{11}), 7.40 – 7.36 (1H, m, H_{12}), 7.35 – 7.31 (2H, m, H_{20}), 7.29 – 7.22 (3H, m, H_{19} and H_{21}), 7.19 (1H, d, *J*=7.8 Hz, H_{14}), 6.82 (1H, d, *J*=7.8 Hz, H_{13}), 5.26 (2H, s, H_8), 3.36 – 3.29 (2H, m, H_{16}), 3.09 – 3.02 (2H, m, H_{17}).

^{13}C NMR (126 MHz, $CDCl_3$) δ 153.4, 142.3, 137.4, 132.8, 130.2, 128.7, 128.6, 128.5, 128.0, 127.5, 126.6, 126.3, 126.1, 125.8, 125.0, 123.6, 123.0, 105.0, 70.2, 37.4, 34.8.

HRMS m/z: $[M+H]^+$ calculated for $[C_{25}H_{23}O]^+$ 339.1743, found 339.1747. Δ = +1.2 ppm.

4-Phenethylnaphthalen-1-ol (1d)

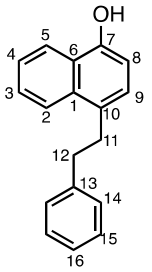

^{1}H NMR (700 MHz, DMSO-d_6) δ 9.90 (1H, s, H_O), 8.17 (1H, d, J=8.3 Hz, H_5), 8.03 (1H, d, J=8.5 Hz, H_2), 7.53 (1H, app t, J=8.0 Hz, H_3), 7.45 (1H, app t, J=7.6 Hz, H_4), 7.32 – 7.24 (4H, m, H_{14} and H_{15}), 7.19 (1H, td, J=6.5, 3.0 Hz, H_{16}), 7.13 (1H, d, J=7.6 Hz, H_9), 6.76 (1H, d, J=7.6 Hz, H_8), 3.20 (2H, dd, J=9.5, 7.1 Hz, H_{11}), 2.92 (2H, dd, J=9.5, 7.1 Hz, H_{12}).

^{13}C NMR (126 MHz, $CDCl_3$) δ 150.2, 142.2, 132.9, 130.4, 128.6, 128.5, 126.5, 126.1, 125.9, 125.0, 124.9, 123.8, 122.5, 108.3, 37.4, 34.8.

^{1}H NMR data in agreement with the literature.[6]

1,2-Dihydroacenaphthylen-5-ol (1e)

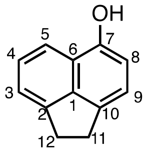

Prepared according to a modification of the procedure reported by Shinokubo and co-workers.[7] Under vacuum, a microwave vial containing a magnetic stirrer bar was dried with a heat gun and cooled to room temperature. The vial was charged with 5-bromo-1,2-dihydroacenaphthylene (233 mg, 1.00 mmol, 1.00 equiv.), Cu_2O (8.0 mg, 0.056 mmol, 5.6 mol%), and 4,7-dihydroxy-1,10-phenanthroline (21.2 mg, 0.100 mmol, 10.0 mol%). The vial was sealed, and evacuated and backfilled with N_2 (3×). ^{t}Bu$_4$NOH (aq.) (40% w/w, 2.48 g, 2.50 mL, 3.85 mmol, 3.85 equiv.) was added under nitrogen, and N_2 was bubbled through the reaction mixture for a further 10 minutes. The reaction was stirred at 110 °C for 16 h. Upon completion, the reaction was quenched with 1 M HCl (6 mL). The aqueous layer was extracted with EtOAc (3×). The combined organic extracts were washed with water and brine, dried over Na_2SO_4, concentrated under reduced pressure, and

purified by flash column chromatography (5% EtOAc in 40-60 °C petroleum ether) to afford the title compound as a white solid (102 mg, 0.599 mmol, 60% yield).

^{1}H NMR (400 MHz, $CDCl_3$) δ 7.73 (1H, dd, *J*=8.3, 1.0 Hz, H_5), 7.44 (1H, dd, *J*=8.3, 6.9 Hz, H_4), 7.29 (1H, d, *J*=6.9 Hz, H_3), 7.10 (1H, dt, *J*=7.3, 1.5 Hz, H_9), 6.81 (1H, d, *J*=7.3 Hz, H_8), 5.11 – 5.08 (1H, m, H_O), 3.45 – 3.37 (2H, m, H_{12}), 3.36 – 3.28 (2H, m, H_{11}).

^{13}C NMR (101 MHz, $CDCl_3$) δ 148.5, 146.0, 140.8, 138.2, 127.2, 122.5, 119.8, 118.9, 116.8, 110.8, 31.2, 29.6.

Data in agreement with the literature.[7]

Synthesis of 6-methoxy-4-methylnaphthalen-1-ol (1f) and 7-methoxy-4-methylnaphthalen-1-ol (1g)

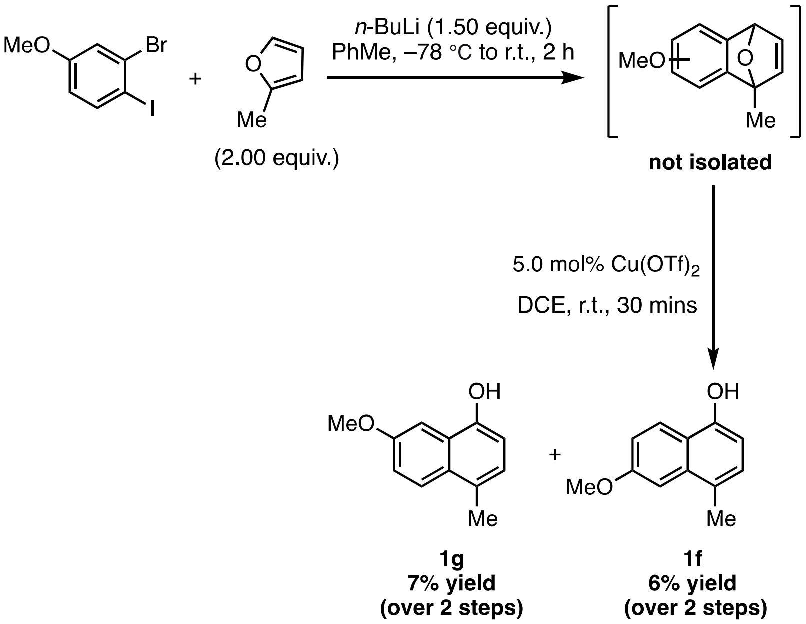

A heat gun-dried round bottom flask containing a magnetic stirrer bar was charged with 2-bromo-1-iodo-4-methoxybenzene (3.13 g, 1.52 ml, 10.0 mmol, 1.00 equiv.), 2-methylfuran (1.64 g, 1.77 mL, 20.0 mmol, 2.00 equiv.), and PhMe (40 mL) under nitrogen. The mixture was cooled to −78 °C, and *n*-BuLi (1.5 M in hexanes, 10.0 ml, 15.0 mmol, 1.50 equiv.) was added under nitrogen. The reaction was stirred at −78 °C for 45 minutes and at room temperature for 1 h. The reaction was quenched with water (30 mL) and extracted with EtOAc (3×). The combined organic extracts were dried over Na_2SO_4, concentrated under reduced pressure, and purified by flash column chromatography (0-10% EtOAc in 40-60 °C petroleum ether) to afford a semi-pure

mixture of regioisomers as a yellow oil (611 mg, R_F = 0.2 in 20% EtOAc in 40-60 °C petroleum ether), which was subjected to the next step without further purification.

Based on the procedure reported by Shao and co-workers.[8] A 2-necked round bottom flask containing a magnetic stirrer bar was charged with $Cu(OTf)_2$ (36 mg, 0.10 mmol, 5.0 mol%) and put under nitrogen. 1,2-Dichloroethane (10 mL) was added, followed by the semi-pure mixture of regioisomers (376 mg, assuming 2.00 mmol, 1.00 equiv.). The reaction was stirred for 30 minutes at room temperature, at which point complete consumption of the starting material was observed by TLC. The reaction was quenched with water (20 mL) and extracted with CH_2Cl_2 (2×). The combined organic extracts were dried over Na_2SO_4, concentrated under reduced pressure, and purified by flash column chromatography (0-10% EtOAc in 40-60 °C petroleum ether) to sequentially afford 6-methoxy-4-methylnaphthalen-1-ol (**1f**) (73 mg, 0.39 mmol, 6% corrected yield over two steps) as a brown solid, and 7-methoxy-4-methylnaphthalen-1-ol (**1g**) (79 mg, 0.42 mmol, 7% corrected yield over two steps) as a light brown solid.

6-Methoxy-4-methylnaphthalen-1-ol (1f)

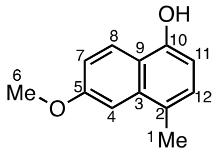

^{1}H NMR (400 MHz, $CDCl_3$) δ 8.14 (1H, dd, *J*=9.6, 1.3 Hz, H_7), 7.21 – 7.15 (2H, m, H_4 and H_8), 7.11 (1H, dd, *J*=7.5, 1.0 Hz, H_{12}), 6.58 (1H, d, *J*=7.5 Hz, H_{11}), 5.20 – 4.99 (1H, m, H_O), 3.96 (3H, s, H_6), 2.56 (3H, s, H_1).

^{13}C NMR (101 MHz, $CDCl_3$) δ 158.1, 150.2, 135.0, 127.0, 125.5, 124.1, 119.8, 117.0, 106.3, 103.3, 55.4, 19.2.

HRMS m/z: $[M]^+$ calculated for $[C_{12}H_{12}O_2]^+$ 188.0832, found 188.0825. Δ = −3.7 ppm.

7-Methoxy-4-methylnaphthalen-1-ol (1g)

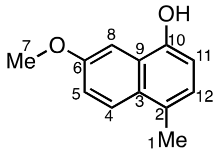

^{1}H NMR (400 MHz, $CDCl_3$) δ 7.86 (1H, d, *J*=9.2 Hz, H_4), 7.51 (1H, d, *J*=2.7 Hz, H_8), 7.21 (1H, dd, *J*=9.2, 2.7 Hz, H_5), 6.99 (1H, dd, *J*=7.5, 1.0 Hz, H_{12}), 6.70 (1H, d, *J*=7.5 Hz, H_{11}), 5.05 (1H, s, H_O), 3.96 (3H, s, H_7), 2.58 (3H, d, *J*=1.0 Hz, H_1).

^{13}C NMR (101 MHz, $CDCl_3$) δ 157.2, 149.1, 129.1, 126.9, 126.1, 125.7, 123.8, 118.9, 108.9, 100.7, 55.5, 19.0.

[N.B. The structure of this isomer was assigned based on an HMBC interaction between C_9 (δ = 125.7) and H_8 and H_4, but not H_5.]

HRMS m/z: $[M]^+$ calculated for $[C_{12}H_{12}O_2]^+$ 188.0832, found 188.0824. Δ = −4.3 ppm.

7-(methoxymethoxy)-4,6-Dimethylnaphthalen-1-ol (1h)

The synthesis of substrate 7-(methoxymethoxy)-4,6-dimethylnaphthalen-1-ol (**1h**) is shown in **Enantioselective Formal Synthesis of (*R*)-Alanense A**.

Synthesis of 3,4-Dimethylnaphthalen-1-ol (1i)

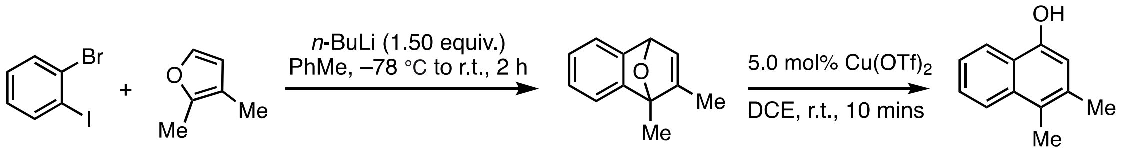

1,2-Dimethyl-1,4-dihydro-1,4-epoxynaphthalene (S17)

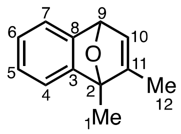

A heat gun-dried round bottom flask containing a magnetic stirrer bar was charged with 1-bromo-2-iodobenzene (2.83 g, 1.28 ml, 10.0 mmol, 1.00 equiv.), 2,3-dimethylfuran (1.00 g, 1.10 ml, 10.4 mmol, 1.04 equiv.), and PhMe (40 mL) under nitrogen. The mixture was cooled to −78 °C, and *n*-BuLi (1.5 M in hexanes, 10.0 ml, 15.0 mmol, 1.50 equiv.) was added under nitrogen. The reaction was stirred at −78 °C for 45 minutes and at room temperature for 1 h. The reaction was quenched with water (30 mL) and extracted with EtOAc (3×). The combined organic extracts were dried over Na_2SO_4, concentrated under reduced pressure, and purified by flash column chromatography (0-5% EtOAc in 40-60 °C petroleum ether) to afford the title compound as a

yellow oil (455 mg, 2.64 mmol, 26% yield). [N.B. The product contained a small impurity, which was removed in the next step.]

^{1}H NMR (500 MHz, $CDCl_3$) δ 7.20 – 7.15 (2H, m, H_4 and H_7), 6.99 – 6.96 (2H, m, H_5 and H_6), 6.51 – 6.47 (1H, m, H_{10}), 5.56 – 5.54 (1H, m, H_9), 1.81 (3H, s, H_1), 1.80 – 1.79 (3H, m, H_{12}).

^{13}C NMR (126 MHz, $CDCl_3$) δ 154.5, 151.3, 151.2, 136.4, 125.0, 124.7, 119.3, 118.5, 90.6, 80.9, 13.7, 12.9.

HRMS m/z: $[M+H]^+$ calculated for $[C_{12}H_{13}O]^+$ 173.0961, found 173.0957. Δ = −2.3 ppm.

3,4-Dimethylnaphthalen-1-ol (1i)

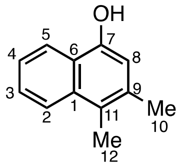

Based on the procedure reported by Shao and co-workers.[8] A 2-necked round bottom flask containing a magnetic stirrer bar was charged with $Cu(OTf)_2$ (36 mg, 0.10 mmol, 5.0 mol%) and put under nitrogen. 1,2-Dichloroethane (10 mL) was added, followed by 1,2-dimethyl-1,4-dihydro-1,4-epoxynaphthalene (**S17**) (344 mg, 2.00 mmol, 1.00 equiv.). The reaction was stirred for 10 minutes at room temperature, at which point complete consumption of the starting material was observed by TLC. The reaction was quenched with water (20 mL) and extracted with CH_2Cl_2 (2×). The combined organic phases were dried with Na_2SO_4, concentrated under reduced pressure, and purified by flash column chromatography (0-4% EtOAc in 40-60 °C petroleum ether) to afford 3,4-dimethylnaphthalen-1-ol (**1i**) (242 mg, 1.41 mmol, 70% yield) as a light brown solid.

^{1}H NMR (400 MHz, $CDCl_3$) δ 8.16 (1H, d, *J*=8.0 Hz, H_5), 7.99 (1H, d, *J*=8.0 Hz, H_2), 7.52 (1H, app t, *J*=8.0 Hz, H_3), 7.44 (1H, app t, *J*=8.0 Hz, H_4), 6.68 (1H, s, H_8), 5.18 – 5.04 (1H, m, H_O), 2.52 (3H, s, H_{12}), 2.43 (3H, s, H_{10}).

^{13}C NMR (101 MHz, $CDCl_3$) δ 149.2, 134.0, 133.2, 126.4, 124.0, 123.9, 123.7, 121.9, 121.9, 111.9, 20.9, 14.1.

Data in agreement with the literature.[9]

4-Bromo-1,2-bis(methoxymethoxy)benzene (S18)

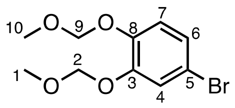

4-Bromobenzene-1,2-diol (378 mg, 2.00 mmol, 2.00 equiv.) was dissolved in DMF (20 mL) under nitrogen. Sodium hydride (240 mg, 10.0 mmol, 5.00 equiv.) was added, followed by chloro(methoxy)methane (483 mg, 0.456 mL, 6.00 mmol, 3.00 equiv.). The reaction was stirred at room temperature for 2 h. Upon completion, the reaction was quenched with sat. NH_4Cl (aq.) and extracted with EtOAc (3 × 10 mL). The combined organic extracts were washed with water and brine, dried over $MgSO_4$, filtered, concentrated under reduced pressure, and purified by flash column chromatography to afford the title compound as a colorless oil (272 mg, 0.982 mmol, 49% yield).

^{1}H NMR (500 MHz, $CDCl_3$) δ 7.30 (1H, d, *J*=2.2 Hz, H_4), 7.07 (1H, dd, *J*=8.7, 2.2 Hz, H_6), 7.03 (1H, d, *J*=8.7 Hz, H_7), 5.21 (2H, s, $H_{2/9}$), 5.20 (2H, s, $H_{2/9}$), 3.51 (3H, s, $H_{1/10}$), 3.50 (3H, s, $H_{1/10}$)

^{13}C NMR (126 MHz, $CDCl_3$) δ 148.2, 146.6, 125.4, 120.1, 118.2, 114.6, 95.7, 95.7, 56.5, 56.4.

Data in agreement with the literature.[10]

Characterization of Products in Scheme 1

General Procedure B: Palladium/(*R*)-sSPhos-Catalyzed Dearomatization of Naphthols with Aryl Bromides

A vial containing a magnetic stirrer bar was sequentially charged with the relevant naphthol (0.100 mmol, 1.00 equiv.), (*R*)-sSPhos (3.8 mg, 0.0075 mmol, 7.5 mol%), K_3PO_4 (31.8 mg, 0.150 mmol, 1.50 equiv.), Pd_2dba_3 (1.1 mg, 0.00125 mmol, 1.25 mol%), and aryl bromide (0.150 mmol, 1.50 equiv.). The vial was sealed, and evacuated and backfilled with N_2 (4×). PhMe (0.50 mL) was added under nitrogen. The reaction was stirred at 90 °C and 900 rpm for 20 h. Upon completion, the reaction mixture was filtered through celite, eluting with EtOAc. The filtrate was concentrated under a stream of air. The crude product was purified by flash column chromatography.

[N.B. Due to the moderate volatility of bromobenzene (b.p. 156 °C), the vial was not left under vacuum for prolonged periods when performing N_2/evacuation cycles with this substrate.]

(*R*)-4-Methyl-4-phenylnaphthalen-1(4*H*)-one (2a)

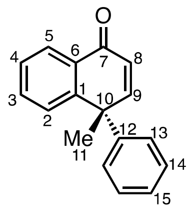

Prepared according to General Procedure B with 4-methylnaphthalen-1-ol (**1a**) (15.8 mg, 0.100 mmol) and bromobenzene (23.6 mg, 0.150 mmol) as the starting materials. Purification by flash column chromatography (0-5% EtOAc in 40-60 °C petroleum ether) afforded the title compound as a brown oil (14.8 mg, 0.063 mmol, 63% yield, 97% *ee*).

^{1}H NMR (500 MHz, $CDCl_3$) δ 8.22 (1H, dd, *J*=8.1, 1.5 Hz, H_5), 7.44 (1H, app td, *J*=7.6, 1.5 Hz, H_3), 7.36 (1H, ddd, *J*=8.1, 7.6, 1.2 Hz, H_4), 7.33 – 7.28 (2H, m, H_{14}), 7.27 – 7.22 (3H, m, H_{13} and H_{15}), 7.11 (1H, dd, *J*=7.6, 1.2 Hz, H_2), 6.93 (1H, d, *J*=10.1 Hz, H_9), 6.44 (1H, d, *J*=10.1 Hz, H_8), 1.89 (3H, s, H_{11}).

^{13}C NMR (126 MHz, $CDCl_3$) δ 185.3, 156.0, 150.0, 143.2, 132.9, 130.8, 128.9, 128.8, 127.2, 127.2, 126.9, 126.7, 125.8, 45.6, 27.2.

Chiral SFC Analysis: CHIRALPAK IE (CO_2:MeOH, 90:10, 2.5 mL min^{-1}, 40 °C, 250 nm) indicated 97% *ee*, t_R = 5.33 (minor), 5.68 (major) minutes.

$[\alpha]_D^{25}$ = + 37.5° (c 0.99, $CHCl_3$).

^{1}H NMR and ^{13}C NMR data in agreement with the literature.[11]

<u>1 mmol scale reaction:</u>

A vial containing a magnetic stirrer bar was sequentially charged with 4-methylnaphthalen-1-ol (**1a**) (158 mg, 1.00 mmol, 1.00 equiv.), (*R*)-sSPhos (38.4 mg, 0.0750 mmol, 7.50 mol%), K_3PO_4 (318 mg, 1.50 mmol, 1.50 equiv.), Pd_2dba_3 (11.4 mg, 0.0125 mmol, 1.25 mol%), and bromobenzene (236 mg, 1.50 mmol, 1.50 equiv.). The vial was sealed, and evacuated and backfilled with N_2 (4×). PhMe (5.0 mL) was added under nitrogen. The reaction was stirred at 90 °C and 900 rpm for 20 h. Upon completion, the reaction mixture was filtered through celite, eluting with EtOAc. The filtrate was concentrated under a stream of air. Purification by flash column chromatography (0-5% EtOAc in hexane) afforded the title compound as a brown oil (169 mg, 0.721 mmol, 72% yield, 98% *ee*).

Chiral SFC Analysis: CHIRALPAK IE (CO_2:MeOH, 90:10, 2.5 mL min^{-1}, 40 °C, 250 nm) indicated 98% *ee*, t_R = 5.22 (minor), 5.53 (major) minutes.

(*R*)-4-Ethyl-4-phenylnaphthalen-1(4*H*)-one (2b)

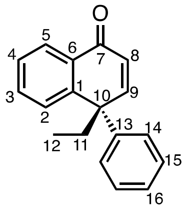

Prepared according to General Procedure B with 4-ethylnaphthalen-1-ol (**1b**) (17.2 mg, 0.100 mmol) and bromobenzene (23.6 mg, 0.150 mmol) as the starting materials. Purification by flash column chromatography (0-5% EtOAc in 40-60 °C petroleum ether) afforded the title compound as a pink solid (14.0 mg, 0.056 mmol, 56% yield, 98% *ee*).

^{1}H NMR (700 MHz, $CDCl_3$) δ 8.24 (1H, dd, *J*=7.6, 1.4 Hz, H_5), 7.46 (1H, app td, *J*=7.6, 1.4 Hz, H_3), 7.38 (1H, app td, *J*=7.6, 1.4 Hz, H_4), 7.34 – 7.30 (2H, m, H_{15}), 7.29 – 7.23 (3H, m, H_{14} and H_{16}), 7.09 (1H, dd, *J*=7.6, 1.4 Hz, H_2), 6.82 (1H, d, *J*=10.1 Hz, H_9), 6.58 (1H, d, *J*=10.1 Hz, H_8), 2.57 – 2.49 (1H, m, H_{11}), 2.45 – 2.38 (1H, m, $H_{11'}$), 0.67 (3H, app t, *J*=7.3 Hz, H_{12}).

^{13}C NMR (176 MHz, $CDCl_3$) δ 185.7, 154.7, 148.1, 143.5, 133.0, 132.5, 128.9, 128.4, 128.0, 127.5, 127.2, 126.9, 126.5, 50.2, 31.7, 8.8.

HRMS m/z: $[M+H]^+$ calculated for $[C_{18}H_{17}O]^+$ 249.1274, found 249.1265. Δ = −3.6 ppm.

Chiral SFC Analysis: CHIRALPAK IE (CO_2:MeOH, 90:10, 2.5 mL min^{-1}, 40 °C, 250 nm) indicated 98% *ee*, t_R = 5.51 (minor), 6.31 (major) minutes.

$[\alpha]_D^{25}$ = +97.9° (c 0.93, $CHCl_3$).

(*R*)-4-Hexyl-4-phenylnaphthalen-1(4*H*)-one (2c)

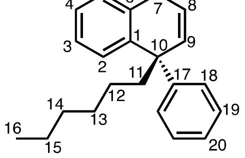

Prepared according to General Procedure B with 4-hexylnaphthalen-1-ol (**1c**) (24.2 mg, 0.100 mmol) and bromobenzene (23.6 mg, 0.150 mmol) as the starting materials, double the usual loadings of (*R*)-sSPhos (7.7 mg, 0.015 mmol, 15 mol%) and Pd_2dba_3 (2.3 mg, 0.0025 mmol, 2.5 mol%), and a reaction time of 48 h. Purification by flash column chromatography (0-5% EtOAc

in 40-60 °C petroleum ether) afforded the title compound as a brown oil (19.3 mg, 0.063 mmol, 63% yield, 75% *ee*).

^{1}H NMR (500 MHz, $CDCl_3$) δ 8.21 (1H, dd, *J*=8.1, 1.5 Hz, H_5), 7.44 (1H, ddd, *J*=8.0, 7.2, 1.5 Hz, H_3), 7.35 (1H, ddd, *J*=8.1, 7.2, 1.2 Hz, H_4), 7.32 – 7.21 (5H, m, H_{18}, H_{19}, and H_{20}), 7.08 (1H, dd, *J*=8.0, 1.2 Hz, H_2), 6.83 (1H, d, *J*=10.1 Hz, H_9), 6.53 (1H, d, *J*=10.1 Hz, H_8), 2.50 – 2.39 (1H, m, H_{11}), 2.37 – 2.26 (1H, m, $H_{11'}$), 1.32 – 1.09 (7H, m, $H_{12'}$, H_{13}, H_{14}, and H_{15}), 0.82 (3H, app t, *J*=7.0 Hz, H_{16}), 0.77 – 0.65 (1H, m, H_{12}).

^{13}C NMR (126 MHz, $CDCl_3$) δ 185.7, 155.1, 148.5, 143.5, 133.0, 132.2, 128.9, 128.4, 127.6, 127.4, 127.2, 126.8, 126.6, 49.8, 39.0, 31.6, 29.7, 24.2, 22.7, 14.1.

HRMS m/z: $[M+H]^+$ calculated for $[C_{22}H_{25}O]^+$ 305.1900, found 305.1898. Δ = −0.7 ppm.

Chiral SFC Analysis: CHIRALPAK IE (CO_2:MeOH, 90:10, 2.5 mL min^{-1}, 40 °C, 250 nm) indicated 75% *ee*, t_R = 5.24 (minor), 5.56 (major) minutes.

$[\alpha]_D^{25}$ = +35.0° (c 0.64, $CHCl_3$).

(*R*)-4-Phenethyl-4-phenylnaphthalen-1(4*H*)-one (2d)

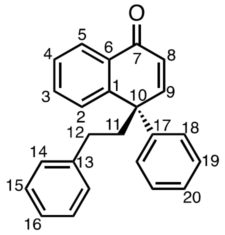

Prepared according to General Procedure B with 4-phenethylnaphthalen-1-ol (**1d**) (24.8 mg, 0.100 mmol) and bromobenzene (23.6 mg, 0.150 mmol) as the starting materials, and a longer reaction time of 48 h. Purification by flash column chromatography (0-15% EtOAc in heptane) afforded the title compound as a purple solid (18.8 mg, 0.058 mmol, 58% yield, 96% *ee*).

^{1}H NMR (500 MHz, $CDCl_3$) δ 8.26 (1H, dd, *J*=7.9, 1.6 Hz, H_5), 7.50 (1H, app td, *J*=7.6, 1.6 Hz, H_3), 7.41 (1H, app td, *J*=7.6, 1.2 Hz, H_4), 7.32 – 7.21 (7H, m, H_{15}, H_{18}, H_{19}, and $H_{16/20}$), 7.21 – 7.14 (2H, m, H_2 and $H_{16/20}$), 7.06 (2H, d, *J*=7.0 Hz, H_{14}), 6.92 (1H, d, *J*=10.1 Hz, H_9), 6.61 (1H, d, *J*=10.1 Hz, H_8), 2.78 (1H, app td, *J*=12.9, 4.7 Hz, H_{11}), 2.65 (1H, app td, *J*=12.9, 4.7 Hz, $H_{11'}$), 2.48 (1H, app td, *J*=12.9, 4.7 Hz, H_{12}), 2.05 (1H, app td, *J*=12.9, 4.7 Hz, $H_{12'}$).

^{13}C NMR (126 MHz, $CDCl_3$) δ 185.5, 154.5, 148.0, 143.1, 141.6, 133.2, 132.3, 129.0, 128.6, 128.4, 128.3, 128.0, 127.4, 127.4, 127.2, 126.8, 126.2, 49.7, 41.1, 30.8.

HRMS m/z: $[M+H]^+$ calculated for $[C_{24}H_{21}O]^+$ 325.1587, found 325.1583. Δ = −1.2 ppm.

Chiral SFC Analysis: CHIRALPAK IK (CO_2:MeOH, 90:10, 2.5 mL min^{-1}, 40 °C, 250 nm) indicated 96% *ee*, t_R = 8.40 (major), 9.41 (minor) minutes.

$[\alpha]_D^{25}$ = +47.6° (c 1.00, $CHCl_3$).

(*R*)-2a-Phenyl-2,2a-dihydroacenaphthylen-5(1*H*)-one (2e)

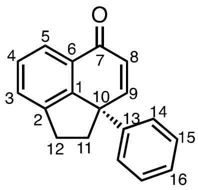

Prepared according to General Procedure B with 1,2-dihydroacenaphthylen-5-ol (**1e**) (17.0 mg, 0.100 mmol) and bromobenzene (23.6 mg, 0.150 mmol) as the starting materials. Purification by flash column chromatography (0-8% EtOAc in hexane) afforded the title compound as a yellow oil (19.9 mg, 0.081 mmol, 81% yield, 93% *ee*).

^{1}H NMR (700 MHz, $CDCl_3$) δ 7.86 (1H, d, *J*=7.6 Hz, H_5), 7.48 – 7.40 (3H, m, H_3, H_4 and H_9), 7.23 – 7.20 (2H, m, H_{15}), 7.19 – 7.15 (1H, m, H_{16}), 6.98 (2H, d, *J*=7.9 Hz, H_{14}), 6.21 (1H, dd, *J*=9.9, 1.2 Hz, H_8), 2.93 – 2.87 (1H, m, H_{12}), 2.85 – 2.79 (2H, m, H_{11} and $H_{12'}$), 2.40 (1H, app tdd, *J*=11.7, 7.4, 1.1 Hz, $H_{11'}$)

^{13}C NMR (176 MHz, $CDCl_3$) δ 186.0, 152.6, 152.4, 143.4, 143.1, 131.0, 128.8, 128.8, 128.5, 128.4, 127.1, 126.5, 123.1, 53.6, 40.5, 30.5.

HRMS m/z: $[M+H]^+$ calculated for $[C_{18}H_{15}O]^+$ 247.1117, found 247.1113. Δ = −1.6 ppm.

Chiral SFC Analysis: CHIRALPAK IJ (CO_2:MeOH, 90:10, 2.5 mL min^{-1}, 40 °C, 260 nm) indicated 93% ee, t_R = 4.22 (major), 5.19 (minor) minutes.

$[\alpha]_D^{25}$ = +240° (c 1.33, $CHCl_3$).

(*R*)-6-Methoxy-4-methyl-4-phenylnaphthalen-1(4*H*)-one (2f)

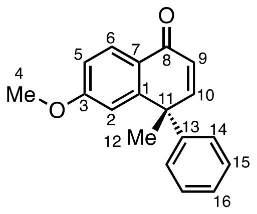

Prepared according to General Procedure B with 6-methoxy-4-methylnaphthalen-1-ol (**1f**) (18.8 mg, 0.100 mmol) and bromobenzene (23.6 mg, 0.150 mmol) as the starting materials. Purification by flash column chromatography (0-10% EtOAc in hexane) afforded the title compound as a yellow solid (22.5 mg, 0.085 mmol, 85% yield, 93% *ee*).

^{1}H NMR (700 MHz, $CDCl_3$) δ 8.19 (1H, d, *J*=8.8 Hz, H_6), 7.30 (2H, app t, *J*=7.7 Hz, H_{15}), 7.25 – 7.22 (3H, m, H_{14} and H_{16}), 6.89 (1H, dd, *J*=8.8, 2.5 Hz, H_5), 6.85 (1H, d, *J*=10.0 Hz, H_{10}), 6.53 (1H, d, *J*=2.5 Hz, H_2), 6.38 (1H, d, *J*=10.0 Hz, H_9), 3.74 (3H, s, H_4), 1.86 (3H, s, H_{12}).

^{13}C NMR (176 MHz, $CDCl_3$) δ 184.5, 163.1, 155.2, 152.3, 143.4, 129.2, 128.9, 127.2, 127.1, 125.8, 124.6, 113.3, 113.3, 55.5, 45.8, 27.4.

HRMS m/z: $[M+H]^+$ calculated for $[C_{18}H_{17}O_2]^+$ 265.1223, found 265.1221. Δ = −0.8 ppm.

Chiral SFC Analysis: CHIRALPAK IJ (CO_2:MeOH, 90:10, 2.5 mL min^{-1}, 40 °C, 250 nm) indicated 93% *ee*, t_R = 3.37 (major), 3.67 (minor) minutes.

$[\alpha]_D^{25}$ = +147° (c 1.50, $CHCl_3$).

(*R*)-7-Methoxy-4-methyl-4-phenylnaphthalen-1(4*H*)-one (2g)

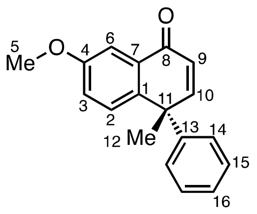

Prepared according to General Procedure B with 7-methoxy-4-methylnaphthalen-1-ol (**1g**) (18.8 mg, 0.100 mmol) and bromobenzene (23.6 mg, 0.150 mmol) as the starting materials. Purification by flash column chromatography (0-10% EtOAc in hexane) afforded the title compound as a yellow solid (25.2 mg, 0.095 mmol, 95% yield, 96% *ee*).

^{1}H NMR (700 MHz, $CDCl_3$) δ 7.71 – 7.63 (1H, m, H_6), 7.29 (2H, app t, *J*=7.5 Hz, H_{15}), 7.25 – 7.20 (3H, m, H_{14} and H_{16}), 7.04 – 7.00 (2H, m, H_2 and H_3), 6.92 (1H, d, *J*=10.0 Hz, H_{10}), 6.43 (1H, d, *J*=10.0 Hz, H_9), 3.87 (3H, s, H_5), 1.86 (3H, s, H_{12}).

^{13}C NMR (176 MHz, $CDCl_3$) δ 185.3, 158.4, 156.4, 143.3, 142.8, 131.8, 130.1, 128.9, 127.2, 127.1, 125.6, 121.8, 107.9, 55.6, 45.3, 27.1.

HRMS m/z: $[M+H]^+$ calculated for $[C_{18}H_{17}O_2]^+$ 265.1223, found 265.1218. Δ = −1.9 ppm.

Chiral SFC Analysis: CHIRALPAK IE (CO_2:MeOH, 90:10, 2.5 mL min^{-1}, 40 °C, 250 nm) indicated 96% *ee*, t_R = 5.81 (minor), 6.22 (major) minutes.

$[\alpha]_D^{25}$ = +70.6° (c 1.26, $CHCl_3$).

(*R*)-7-(methoxymethoxy)-4,6-Dimethyl-4-phenylnaphthalen-1(4*H*)-one (2h)

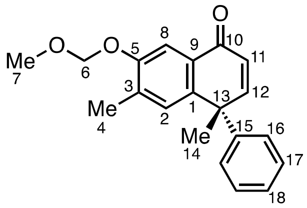

Prepared according to General Procedure B with 7-(methoxymethoxy)-4,6-dimethylnaphthalen-1-ol (**1h**) (23.2 mg, 0.100 mmol) and bromobenzene (23.6 mg, 0.150 mmol) as the starting materials. Purification by flash column chromatography (0-10% EtOAc in 40-60 °C petroleum ether) afforded the title compound as a yellow oil (25.4 mg, 0.082 mmol, 82% yield, 95% *ee*).

^{1}H NMR (400 MHz, $CDCl_3$) δ 7.77 (s, 1H, H_8), 7.33 – 7.27 (m, 2H, H_{17}), 7.26 – 7.21 (m, 3H, H_{16} and H_{18}), 6.90 – 6.83 (m, 2H, H_2 and H_{12}), 6.39 (d, *J* = 10.0 Hz, 1H, H_{11}), 5.32 – 5.24 (m, 2H, H_6), 3.49 (s, 3H, H_7), 2.19 (s, 3H, H_4), 1.85 (s, 3H, H_{14}).

^{13}C NMR (101 MHz, $CDCl_3$) δ 185.0, 155.9, 154.3, 143.5, 143.4, 134.0, 130.9, 130.0, 128.8, 127.2, 127.1, 125.6, 109.5, 94.5, 56.4, 45.2, 27.1, 17.0.

HRMS m/z: $[M+H]^+$ calculated for $[C_{20}H_{21}O_3]^+$ 309.1485, found 309.1484. Δ = −0.2 ppm.

Chiral SFC Analysis: CHIRALPAK IA (CO_2:MeOH, 95:05, 2.5 mL min^{-1}, 40 °C, 250 nm) indicated 95% *ee*, t_R = 6.00 (minor), 6.65 (major) minutes.

$[\alpha]_D^{25}$ = +125° (c 1.02, $CHCl_3$).

(*R*)-3,4-Dimethyl-4-phenylnaphthalen-1(4*H*)-one (2i)

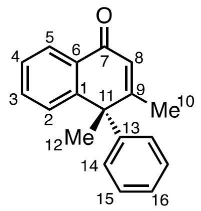

Prepared according to General Procedure B with 3,4-dimethylnaphthalen-1-ol (**1i**) (17.2 mg, 0.100 mmol) and bromobenzene (23.6 mg, 0.150 mmol) as the starting materials, double the usual loadings of (*R*)-sSPhos (7.7 mg, 0.015 mmol, 15 mol%) and Pd_2dba_3 (2.3 mg, 0.0025 mmol, 2.5 mol%), and a reaction time of 48 h. Purification by flash column chromatography (0-5% EtOAc in hexane) and preparatory TLC (10% EtOAc in PhMe) afforded the title compound as a colorless solid (6.5 mg, 0.026 mmol, 26% yield, 95% *ee*).

^{1}H NMR (700 MHz, $CDCl_3$) δ 8.19 (1H, d, *J*=7.9 Hz, H_5), 7.39 (1H, app t, *J*=7.7 Hz, H_3), 7.35 – 7.27 (3H, m, H_4 and H_{15}), 7.23 (1H, app t, *J*=7.3 Hz, H_{16}), 7.18 (2H, d, *J*=7.6 Hz, H_{14}), 7.01 (1H, d, *J*=7.7 Hz, H_2), 6.43 (1H, s, H_8), 1.86 (3H, s, H_{12}), 1.78 (3H, s, H_{10}).

^{13}C NMR (176 MHz, $CDCl_3$) δ 184.9, 164.7, 151.7, 143.6, 132.6, 130.1, 129.0, 128.8, 127.4, 127.1, 126.8, 126.6, 126.2, 49.1, 27.0, 20.8.

HRMS m/z: $[M+H]^+$ calculated for $[C_{18}H_{17}O]^+$ 249.1274, found 249.1271. Δ = −1.2 ppm.

Chiral SFC Analysis: CHIRALPAK IJ (CO_2:MeOH, 90:10, 2.5 mL min^{-1}, 40 °C, 260 nm) indicated 95% *ee,* t_R = 2.63 (major), 3.05 (minor) minutes.

$[\alpha]_D^{25}$ = +29.3° (c 0.43, $CHCl_3$).

(*R*)-4-(4-methoxyphenyl)-4-Methylnaphthalen-1(4*H*)-one (2j)

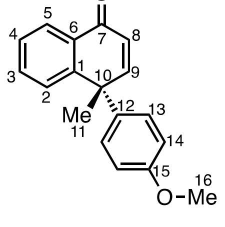

Prepared according to General Procedure B with 4-methylnaphthalen-1-ol (15.8 mg, 0.100 mmol) and 1-bromo-4-methoxybenzene (28.1 mg, 0.150 mmol) as the starting materials. Purification by

flash column chromatography (0-5% EtOAc in 40-60 °C petroleum ether) afforded the title compound as a yellow oil (16.9 mg, 0.064 mmol, 64% yield, 98% *ee*).

^{1}H NMR (500 MHz, $CDCl_3$) δ 8.20 (1H, ddd, *J*=7.8, 1.5, 0.5 Hz, H_5), 7.44 (1H, ddd, *J*=7.9, 7.2, 1.5 Hz, H_3), 7.35 (1H, ddd, *J*=7.8, 7.2, 1.2 Hz, H_4), 7.16 – 7.13 (2H, m, H_{13}), 7.11 (1H, ddd, *J*=7.9, 1.2, 0.5 Hz, H_2), 6.90 (1H, d, *J*=10.1 Hz, H_9), 6.85 – 6.80 (2H, m, H_{14}), 6.41 (1H, d, *J*=10.1 Hz, H_8), 3.78 (3H, s, H_{16}), 1.86 (3H, s, H_{11}).

^{13}C NMR (126 MHz, $CDCl_3$) δ 185.4, 158.7, 156.4, 150.3, 135.1, 132.9, 130.7, 128.8, 128.3, 126.8, 126.7, 125.5, 114.2, 55.4, 45.0, 27.4.

HRMS m/z: $[M+H]^+$ calculated for $[C_{18}H_{17}O_2]^+$ 265.1223, found 265.1221. Δ = −0.8 ppm.

Chiral SFC Analysis: CHIRALPAK IE (CO_2:MeOH, 90:10, 2.5 mL min^{-1}, 40 °C, 260 nm) indicated 98% ee, t_R = 7.89 (minor), 8.76 (major) minutes.

$[\alpha]_D^{25}$ = +147° (c 1.13, $CHCl_3$).

(*R*)-4-Methyl-4-(3,4,5-trimethoxyphenyl)naphthalen-1(4*H*)-one (2k)

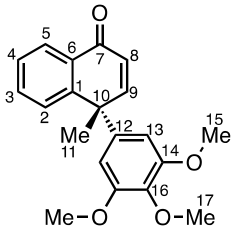

Prepared according to General Procedure B with 4-methylnaphthalen-1-ol (15.8 mg, 0.100 mmol) and 5-bromo-1,2,3-trimethoxybenzene (37.1 mg, 0.150 mmol) as the starting materials, and a longer reaction time of 48 h. Purification by flash column chromatography (0-20% EtOAc in 40-60 °C petroleum ether) afforded the title compound as a yellow solid (11.3 mg, 0.035 mmol, 35% yield, 89% *ee*).

^{1}H NMR (700 MHz, $CDCl_3$) δ 8.21 (1H, d, *J*=7.9 Hz, H_5), 7.47 (1H, app t, *J*=7.5 Hz, H_3), 7.37 (1H, app t, *J*=7.5 Hz, H_4), 7.16 (1H, d, *J*=7.9 Hz, H_2), 6.93 (1H, d, *J*=10.0 Hz, H_9), 6.44 (1H, d, *J*=10.0 Hz, H_8), 6.42 (2H, s, H_{13}), 3.82 (3H, s, H_{17}), 3.77 (6H, s, H_{15}), 1.85 (3H, s, H_{11}).

^{13}C NMR (176 MHz, $CDCl_3$) δ 185.3, 155.9, 153.4, 149.8, 138.7, 137.4, 133.0, 130.6, 128.6, 127.1, 126.7, 125.8, 104.7, 61.0, 56.4, 45.7, 27.6.

HRMS m/z: $[M+H]^+$ calculated for $[C_{20}H_{21}O_4]^+$ 325.1434, found 325.1429. Δ = −1.5 ppm.

Chiral SFC Analysis: CHIRALPAK IK (CO_2:MeOH, 80:20, 2.5 mL min^{-1}, 40 °C, 250 nm) indicated 89% *ee*, t_R = 4.69 (major), 5.52 (minor) minutes.

$[\alpha]_D^{25}$ = +90.0° (c 0.75, $CHCl_3$).

(*S*)-4-(2-methoxyphenyl)-4-Methylnaphthalen-1(4*H*)-one (2l)

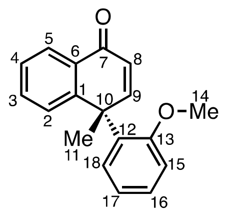

Prepared according to General Procedure B with 4-methylnaphthalen-1-ol (15.8 mg, 0.100 mmol) and 1-bromo-2-methoxybenzene (28.1 mg, 0.150 mmol) as the starting materials, double the usual loadings of (*R*)-sSPhos (7.7 mg, 0.015 mmol, 15 mol%) and Pd_2dba_3 (2.3 mg, 0.0025 mmol, 2.5 mol%), and a longer reaction time of 48 h. Purification by flash column chromatography (0-6% EtOAc in hexane) afforded the title compound as a yellow solid (10.0 mg, 0.038 mmol, 38% yield, 98% *ee*).

^{1}H NMR (700 MHz, $CDCl_3$) δ 8.20 (1H, dd, *J*=7.9, 1.4 Hz, H_5), 7.60 (1H, dd, *J*=7.7, 1.7 Hz, H_{18}), 7.36 (1H, app td, *J*=7.8, 1.4 Hz, H_3), 7.33 – 7.29 (2H, m, H_4 and H_{16}), 7.06 (1H, app td, *J*=7.7, 1.2 Hz, H_{17}), 6.97 (1H, dd, *J*=7.8, 1.3 Hz, H_2), 6.85 (1H, d, *J*=10.0 Hz, H_9), 6.75 (1H, dd, *J*=8.1, 1.2 Hz, H_{15}), 6.44 (1H, d, *J*=10.0 Hz, H_8), 3.28 (3H, s, H_{14}), 1.77 (3H, s, H_{11}).

^{13}C NMR (176 MHz, $CDCl_3$) δ 185.9, 158.0, 155.4, 151.0, 132.3, 131.8, 131.3, 129.3, 127.1, 126.6, 126.4, 126.3, 126.1, 120.8, 112.5, 55.3, 43.9, 29.2.

HRMS m/z: $[M+H]^+$ calculated for $[C_{18}H_{17}O_2]^+$ 265.1223, found 265.1220. Δ = −1.1 ppm.

Chiral SFC Analysis: CHIRALPAK IJ (CO_2:MeOH, 95:05, 2.5 mL min^{-1}, 40 °C, 250 nm) indicated 98% ee, t_R = 3.86 (major), 4.17 (minor) minutes.

$[\alpha]_D^{25}$ = +68.3° (c 0.67, $CHCl_3$).

(*R*)-4-(3-fluoro-5-methoxyphenyl)-4-Methylnaphthalen-1(4*H*)-one (2m)

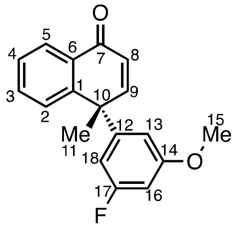

Prepared according to General Procedure B with 4-methylnaphthalen-1-ol (15.8 mg, 0.100 mmol) and 1-bromo-3-fluoro-5-methoxybenzene (30.8 mg, 0.150 mmol) as the starting materials. Purification by flash column chromatography (0-7% EtOAc in 40-60 °C petroleum ether) afforded the title compound as a white solid (14.7 mg, 0.052 mmol, 52% yield, 91% *ee*).

^{1}H NMR (500 MHz, $CDCl_3$) δ 8.20 (1H, dd, *J*=7.5, 1.5 Hz, H_5), 7.46 (1H, ddd, *J*=8.0, 7.5, 1.5 Hz, H_3), 7.37 (1H, app td, *J*=7.5, 1.2 Hz, H_4), 7.13 (1H, d, *J*=8.0 Hz, H_2), 6.88 (1H, d, *J*=10.1 Hz, H_9), 6.57 – 6.53 (2H, m, H_{13} and $H_{16/18}$), 6.49 (1H, app dt, *J*=10.4, 2.2 Hz, $H_{16/18}$), 6.44 (1H, d, *J*=10.1 Hz, H_8), 3.72 (3H, s, H_{15}), 1.84 (3H, s, H_{11}).

^{13}C NMR (126 MHz, $CDCl_3$) δ 185.0, 163.9 (d, *J*=245.7 Hz), 161.2 (d, *J*=11.2 Hz), 155.0, 149.1, 146.5 (d, *J*=8.8 Hz), 133.1, 130.7, 128.5, 127.2, 126.8, 126.2, 109.8 (d, *J*=2.7 Hz), 106.6 (d, *J*=22.9 Hz), 99.9 (d, *J*=25.3 Hz), 55.7, 45.5 (d, *J*=2.1 Hz), 27.3.

^{19}F NMR (376 MHz, $CDCl_3$) δ −110.5.

HRMS m/z: $[M+H]^+$ calculated for $[C_{18}H_{16}FO_2]^+$ 283.1129, found 283.1125. Δ = −1.4 ppm.

Chiral SFC Analysis: CHIRALPAK IK (CO_2:MeOH, 95:05, 2.5 mL min^{-1}, 40 °C, 250 nm) indicated 91% *ee*, t_R = 6.94 (minor), 7.33 (major) minutes.

$[\alpha]_D^{25}$ = +80.0° (c 0.98, $CHCl_3$).

(*R*)-4-(3,4-bis(methoxymethoxy)phenyl)-4-Methylnaphthalen-1(4*H*)-one (2n)

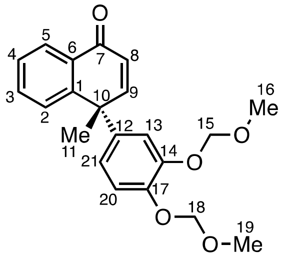

Prepared according to General Procedure B with 4-methylnaphthalen-1-ol (15.8 mg, 0.100 mmol) and 4-bromo-1,2-bis(methoxymethoxy)benzene (**S18**) (41.6 mg, 0.150 mmol) as the starting

materials. Purification by flash column chromatography (0-30% EtOAc in heptane) afforded the title compound as a yellow solid (23.3 mg, 0.066 mmol, 66% yield, 95% *ee*).

^{1}H NMR (500 MHz, $CDCl_3$) δ 8.19 (1H, ddd, *J*=7.8, 1.5, 0.6 Hz, H_5), 7.45 (1H, ddd, *J*=8.0, 7.2, 1.5 Hz, H_3), 7.35 (1H, ddd, *J*=7.8, 7.2, 1.2 Hz, H_4), 7.14 (1H, ddd, *J*=8.0, 1.2, 0.6 Hz, H_2), 7.06 (1H, d, *J*=8.5 Hz, H_{20}), 7.01 (1H, d, *J*=2.3 Hz, H_{13}), 6.91 (1H, d, *J*=10.1 Hz, H_9), 6.81 (1H, dd, *J*=8.5, 2.3 Hz, H_{21}), 6.41 (1H, d, *J*=10.1 Hz, H_8), 5.19 (2H, s, $H_{15/18}$), 5.13 (2H, s, $H_{15/18}$), 3.49 (3H, s, $H_{16/19}$), 3.46 (3H, s, $H_{16/19}$), 1.84 (3H, s, H_{11}).

^{13}C NMR (126 MHz, $CDCl_3$) δ 185.3, 156.0, 149.9, 147.3, 146.7, 137.5, 132.9, 130.7, 128.7, 126.9, 126.7, 125.7, 121.6, 116.8, 116.6, 96.0, 95.5, 56.4, 56.4, 45.2, 27.5.

HRMS m/z: $[M+H]^+$ calculated for $[C_{21}H_{23}O_5]^+$ 355.1540, found 355.1540. Δ = 0.0 ppm.

Chiral SFC Analysis: CHIRALPAK IF (CO_2 (A):0.1% NH_3 in MeOH (B), isocratic 5% B, 2.5 mL min^{-1}, 40 °C, 254 nm) indicated 95% *ee*, t_R = 2.04 (minor), 2.25 (major) minutes.

$[\alpha]_D^{25}$ = +108° (c 1.17, $CHCl_3$).

(*R*)-4-(4-(dimethylamino)phenyl)-4-Methylnaphthalen-1(4*H*)-one (2o)

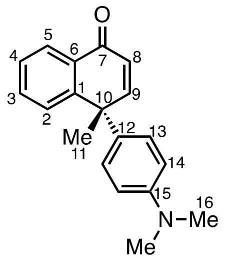

Prepared according to General Procedure B with 4-methylnaphthalen-1-ol (15.8 mg, 0.100 mmol) and 4-bromo-*N*,*N*-dimethylaniline (30.0 mg, 0.150 mmol) as the starting materials. Purification by flash column chromatography (0-20% EtOAc in heptane) afforded the title compound as a yellow solid (21.4 mg, 0.077 mmol, 77% yield, 96% *ee*).

^{1}H NMR (500 MHz, $CDCl_3$) δ 8.20 (1H, ddd, *J*=8.1, 1.6, 0.6 Hz, H_5), 7.43 (1H, ddd, *J*=7.9, 7.2, 1.6 Hz, H_3), 7.33 (1H, ddd, *J*=8.1, 7.2, 1.2 Hz, H_4), 7.16 (1H, ddd, *J*=7.9, 1.2, 0.6 Hz, H_2), 7.11 – 7.04 (2H, m, H_{13}), 6.91 (1H, d, *J*=10.1 Hz, H_9), 6.68 – 6.62 (2H, m, H_{14}), 6.39 (1H, d, *J*=10.1 Hz, H_8), 2.92 (6H, s, H_{16}), 1.85 (3H, s, H_{11}).

^{13}C NMR (126 MHz, $CDCl_3$) δ 185.6, 157.0, 150.7, 149.6, 132.8, 130.8, 130.3, 128.8, 127.9, 126.6, 126.6, 125.2, 112.6, 44.8, 40.6, 27.2.

HRMS m/z: $[M+H]^+$ calculated for $[C_{19}H_{20}NO]^+$ 278.1539, found 278.1536. Δ = −1.1 ppm.

Chiral SFC Analysis: CHIRALPAK AD (CO_2 (A):0.1% NH_3 in MeOH (B), 5% B – 50% B over two minutes, then isocratic 50% B for 2 minutes, 2.5 mL min^{-1}, 40 °C, 254 nm) indicated 96% *ee*, t_R = 1.20 (minor), 1.60 (major) minutes.

$[\alpha]_D^{25}$ = +230° (c 1.07, $CHCl_3$).

(*R*)-4-(3-(dibenzylamino)phenyl)-4-Methylnaphthalen-1(4*H*)-one (2p)

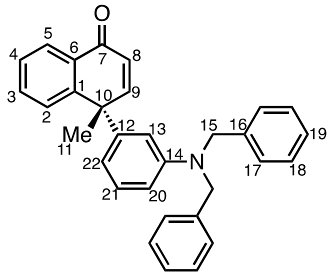

Prepared according to General Procedure B with 4-methylnaphthalen-1-ol (15.8 mg, 0.100 mmol) and *N*,*N*-dibenzyl-3-bromoaniline (52.8 mg, 0.150 mmol) as the starting materials, and a longer reaction time of 48 h. Purification by flash column chromatography (0-10% EtOAc in heptane) afforded the title compound along with a 4-methylnaphthalen-1-ol impurity. The impurity was removed according to the following procedure.

NaOH (aq.) (2 M, 0.5 mL) and $CDCl_3$ (0.5 mL) were added and the layers separated. The aqueous layer was further extracted with $CDCl_3$ (2×0.5 mL). The combined organic extracts were dried over Mg_2SO_4, filtered, and concentrated under reduced pressure to afford the title compound as a yellow solid (23.5 mg, 0.055 mmol, 55% yield, 96% *ee*).

^{1}H NMR (500 MHz, $CDCl_3$) δ 8.12 (1H, dd, *J*=7.7, 1.6 Hz, H_5), 7.35 (1H, app td, *J*=7.6, 1.6 Hz, H_3), 7.31 – 7.27 (5H, m, H_4 and H_{18}), 7.26 – 7.21 (2H, m, H_{19}), 7.20 – 7.15 (4H, m, H_{17}), 7.09 (1H, dd, *J*=8.3, 7.7 Hz, H_{21}), 6.99 (1H, dd, *J*=7.6, 1.3 Hz, H_2), 6.84 (1H, d, *J*=10.1 Hz, H_9), 6.63 (1H, ddd, *J*=8.3, 2.6, 0.8 Hz, H_{20}), 6.60 (1H, ddd, *J*=7.7, 1.7, 0.8 Hz, H_{22}), 6.48 (1H, dd, *J*=2.6, 1.7 Hz, H_{13}), 6.31 (1H, d, *J*=10.1 Hz, H_8), 4.59 (2H, d, *J*=17.1 Hz, H_{15}), 4.58 (2H, d, *J*=17.1 Hz, $H_{15'}$), 1.71 (3H, s, H_{11}).

^{13}C NMR (126 MHz, $CDCl_3$) δ 185.3, 156.3, 150.0, 149.1, 143.9, 138.6, 132.7, 130.6, 129.5, 128.8, 128.5, 127.1, 126.8, 126.7, 126.5, 125.6, 115.3, 112.3, 111.7, 55.2, 45.7, 27.0.

HRMS m/z: $[M+H]^+$ calculated for $[C_{31}H_{28}NO]^+$ 430.2165, found 430.2165. Δ = 0.0 ppm.

Chiral SFC Analysis: CHIRALPAK AD (CO_2 (A):0.1% DEA in IPA (B), 5% B – 50% B over two minutes, then isocratic 50% B for 2 minutes, 2.5 mL min^{-1}, 40 °C, 270 nm) indicated 96% *ee*, t_R = 1.53 (minor), 1.66 (major) minutes.

$[\alpha]_D^{25}$ = +55.4° (c 1.18, $CHCl_3$).

(*R*)-4-(3,5-dimethylphenyl)-4-Methylnaphthalen-1(4*H*)-one (2q)

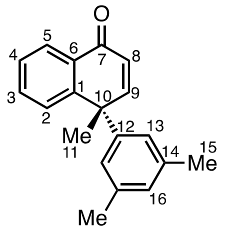

Prepared according to General Procedure B with 4-methylnaphthalen-1-ol (15.8 mg, 0.100 mmol) and 1-bromo-3,5-dimethylbenzene (27.8 mg, 0.150 mmol) as the starting materials. Purification by flash column chromatography (0-4% EtOAc in 40-60 °C petroleum ether) afforded the title compound as a yellow solid (10.5 mg, 0.040 mmol, 40% yield, 98% *ee*).

^{1}H NMR (500 MHz, $CDCl_3$) δ 8.21 (1H, dd, *J*=8.1, 1.6 Hz, H_5), 7.44 (1H, ddd, *J*=7.9, 7.2, 1.6 Hz, H_3), 7.35 (1H, ddd, *J*=8.1, 7.2, 1.2 Hz, H_4), 7.13 (1H, dd, *J*=7.9, 1.2 Hz, H_2), 6.91 (1H, d, *J*=10.1 Hz, H_9), 6.88 (1H, s, H_{16}), 6.83 (2H, s, H_{13}), 6.43 (1H, d, *J*=10.1 Hz, H_8), 2.26 (6H, s, H_{15}), 1.85 (3H, s, H_{11}).

^{13}C NMR (126 MHz, $CDCl_3$) δ 185.5, 156.4, 150.2, 143.0, 138.4, 132.9, 130.8, 128.9, 128.8, 126.8, 126.7, 125.7, 125.0, 45.5, 27.3, 21.6.

HRMS m/z: $[M+H]^+$ calculated for $[C_{19}H_{19}O]^+$ 263.1430, found 263.1431. Δ = +0.4 ppm.

Chiral SFC Analysis: CHIRALPAK IK (CO_2:MeOH, 95:05, 2.5 mL min^{-1}, 40 °C, 250 nm) indicated 98% *ee*, t_R = 6.82 (major), 7.34 (minor) minutes.

$[\alpha]_D^{25}$ = +79.7° (c 0.70, $CHCl_3$).

(*R*)-4-(3-chlorophenyl)-4-Methylnaphthalen-1(4*H*)-one (2r)

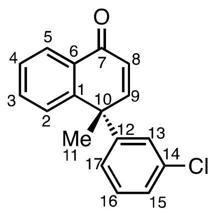

Prepared according to General Procedure B with 4-methylnaphthalen-1-ol (15.8 mg, 0.100 mmol) and 1-bromo-3-chlorobenzene (28.7 mg, 0.150 mmol) as the starting materials, and a longer reaction time of 48 h. Purification by flash column chromatography (0-5% EtOAc in 40-60 °C petroleum ether) afforded the title compound as a yellow solid (17.4 mg, 0.065 mmol, 65% yield, 95% *ee*).

^{1}H NMR (400 MHz, $CDCl_3$) δ 8.22 (1H, dd, *J*=7.6, 1.6 Hz, H_5), 7.46 (1H, app td, *J*=7.6, 1.6 Hz, H_3), 7.38 (1H, app td, *J*=7.6, 1.4 Hz, H_4), 7.29 – 7.19 (3H, m, 3×$H_{13/15/16/17}$), 7.11 – 7.06 (2H, m, H_2 and 1×$H_{13/15/16/17}$), 6.88 (1H, d, *J*=10.1 Hz, H_9), 6.45 (1H, d, *J*=10.1 Hz, H_8), 1.87 (3H, s, H_{11}).

^{13}C NMR (101 MHz, $CDCl_3$) δ 185.0, 155.0, 149.1, 145.5, 134.8, 133.1, 130.7, 130.1, 128.7, 127.5, 127.4, 127.2, 126.9, 126.2, 125.7, 45.5, 27.3.

HRMS m/z: $[M+H]^+$ calculated for $[C_{17}H_{14}ClO]^+$ 269.0728, found 269.0724. Δ = −1.5 ppm.

Chiral SFC Analysis: CHIRALPAK IE (CO_2:MeOH, 90:10, 2.5 mL min^{-1}, 40 °C, 250 nm) indicated 95% *ee*, t_R = 5.72 (minor), 6.15 (major) minutes.

$[\alpha]_D^{25}$ = +64.2° (c 1.16, $CHCl_3$).

(*R*)-4-(3-chloro-5-fluorophenyl)-4-Methylnaphthalen-1(4*H*)-one (2s)

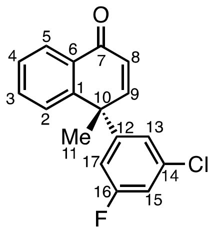

Prepared according to General Procedure B with 4-methylnaphthalen-1-ol (15.8 mg, 0.100 mmol) and 1-bromo-3-chloro-5-fluorobenzene (31.4 mg, 0.150 mmol) as the starting materials, double the usual loadings of (*R*)-sSPhos (7.7 mg, 0.015 mmol, 15 mol%) and Pd_2dba_3 (2.3 mg, 0.0025 mmol, 2.5 mol%), and a longer reaction time of 48 h. Purification by flash column

chromatography (0-5% EtOAc in hexane) afforded the title compound as a brown solid (11.3 mg, 0.039 mmol, 39%, 89% *ee*).

^{1}H NMR (700 MHz, $CDCl_3$) δ 8.22 (1H, dd, *J*=8.0, 1.6 Hz, H_5), 7.49 (1H, ddd, *J*=8.1, 7.2, 1.6 Hz, H_3), 7.40 (1H, ddd, *J*=8.0, 7.2, 1.1 Hz, H_4), 7.09 (1H, dd, *J*=8.1, 1.1 Hz, H_2), 7.02 (1H, app t, *J*=1.8 Hz, H_{13}), 6.98 (1H, app dt, *J*=8.1, 1.8 Hz, H_{15}), 6.86 – 6.82 (2H, m, H_9 and H_{17}), 6.47 (1H, d, *J*=10.1 Hz, H_8), 1.86 (3H, s, H_{11}).

^{13}C NMR (176 MHz, $CDCl_3$) δ 184.7, 162.9 (d, *J*=250.5 Hz), 154.1, 148.4, 147.4 (d, *J*=7.4 Hz), 135.6 (d, *J*=10.8 Hz), 133.3, 130.7, 128.5, 127.5, 127.0, 126.6, 123.5 (d, *J*=3.1 Hz), 115.3 (d, *J*=24.6 Hz), 113.2 (d, *J*=22.4 Hz), 45.4 (d, *J*=1.7 Hz), 27.3.

^{19}F NMR (471 MHz, $CDCl_3$) δ −109.7.

HRMS m/z: $[M+H]^+$ calculated for $[C_{17}H_{13}ClFO]^+$ 287.0633, found 287.0637. Δ = +1.4 ppm.

Chiral SFC Analysis: CHIRALPAK IG (CO_2:MeOH, 95:05, 2.5 mL min^{-1}, 40 °C, 250 nm) indicated 89% *ee*, t_R = 6.11 (minor), 7.20 (major) minutes.

$[\alpha]_D^{25}$ = +57.1° (c 0.75, $CHCl_3$).

Ethyl (*R*)-4-(1-methyl-4-oxo-1,4-dihydronaphthalen-1-yl)benzoate (2t)

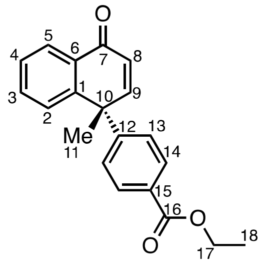

Prepared according to General Procedure B with 4-methylnaphthalen-1-ol (15.8 mg, 0.100 mmol) and ethyl 4-bromobenzoate (34.4 mg, 0.150 mmol) as the starting materials, and a longer reaction time of 48 h. Purification by flash column chromatography (0-7% EtOAc in 40-60 °C petroleum ether) afforded the title compound as a yellow oil (19.1 mg, 0.062 mmol, 62%, 96% *ee*).

^{1}H NMR (400 MHz, $CDCl_3$) δ 8.22 (1H, dd, *J*=7.8, 1.6 Hz, H_5), 7.97 (2H, d, *J*=8.5 Hz, H_{14}), 7.44 (1H, app td, *J*=7.5, 1.6 Hz, H_3), 7.37 (1H, app td, *J*=7.5, 1.4 Hz, H_4), 7.30 (2H, d, *J*=8.5 Hz, H_{13}), 7.05 (1H, dd, *J*=7.8, 1.4 Hz, H_2), 6.89 (1H, d, *J*=10.1 Hz, H_9), 6.47 (1H, d, *J*=10.1 Hz, H_8), 4.36 (2H, app q, *J*=7.1 Hz, H_{17}), 1.91 (3H, s, H_{11}), 1.37 (3H, app t, *J*=7.1 Hz, H_{18}).

^{13}C NMR (101 MHz, $CDCl_3$) δ 185.0, 166.3, 155.0, 149.2, 148.3, 133.1, 130.8, 130.1, 129.5, 128.7, 127.3 (2×), 126.9, 126.3, 61.2, 45.7, 27.2, 14.4.

HRMS m/z: $[M+H]^+$ calculated for $[C_{20}H_{19}O_3]^+$ 307.1329, found 307.1321. Δ = −2.6 ppm.

Chiral SFC Analysis: CHIRALPAK IE (CO_2:MeOH, 80:20, 2.5 mL min^{-1}, 40 °C, 250 nm) indicated 96% *ee*, t_R = 6.15 (minor), 7.14 (major) minutes.

$[\alpha]_D^{25}$ = +97.3° (c 1.27, $CHCl_3$).

(*R*)-4-(1-methyl-4-oxo-1,4-dihydronaphthalen-1-yl)Benzonitrile (2u)

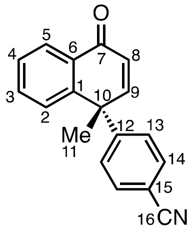

Prepared according to General Procedure B with 4-methylnaphthalen-1-ol (15.8 mg, 0.100 mmol) and 4-bromobenzonitrile (27.3 mg, 0.150 mmol) as the starting materials, double the usual loadings of (*R*)-sSPhos (7.7 mg, 0.015 mmol, 15 mol%) and Pd_2dba_3 (2.3 mg, 0.0025 mmol, 2.5 mol%), and a reaction time of 48 h. Purification by flash column chromatography (0-20% EtOAc in 40-60 °C petroleum ether) and preparatory TLC (40% EtOAc in 40-60 °C petroleum ether) afforded the title compound as a colorless solid (5.6 mg, 0.022 mmol, 22%, 90% *ee*).

^{1}H NMR (400 MHz, $CDCl_3$) δ 8.23 (1H, dd, *J*=7.7, 1.6 Hz, H_5), 7.60 (2H, d, *J*=8.5 Hz, H_{14}), 7.47 (1H, app td, *J*=7.7, 1.6 Hz, H_3), 7.40 (1H, app td, *J*=7.7, 1.4 Hz, H_4), 7.35 (2H, d, *J*=8.5 Hz, H_{13}), 7.02 (1H, dd, *J*=7.7, 1.4 Hz, H_2), 6.85 (1H, d, *J*=10.2 Hz, H_9), 6.48 (1H, d, *J*=10.2 Hz, H_8), 1.91 (3H, s, H_{11}).

^{13}C NMR (101 MHz, $CDCl_3$) δ 184.7, 154.1, 148.9, 148.5, 133.3, 132.7, 130.8, 128.6, 128.1, 127.6, 127.1, 126.7, 118.6, 111.4, 45.8, 27.1.

HRMS m/z: $[M+H]^+$ calculated for $[C_{18}H_{14}NO]^+$ 260.1070, found 260.1063. Δ = −2.7 ppm.

Chiral SFC Analysis: CHIRALPAK IE (CO_2:MeOH, 80:20, 2.5 mL min^{-1}, 40 °C, 250 nm) indicated 90% *ee*, t_R = 6.08 (minor), 6.52 (major) minutes.

$[\alpha]_D^{25}$ = +114° (c 0.37, $CHCl_3$).

(*R*)-4-Methyl-4-(4-nitrophenyl)naphthalen-1(4*H*)-one (2v)

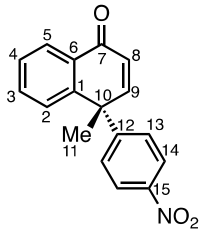

Prepared according to General Procedure B with 4-methylnaphthalen-1-ol (15.8 mg, 0.100 mmol) and 1-bromo-4-nitrobenzene (30.3 mg, 0.150 mmol) as the starting materials, double the usual loadings of (*R*)-sSPhos (7.7 mg, 0.015 mmol, 15 mol%) and Pd_2dba_3 (2.3 mg, 0.0025 mmol, 2.5 mol%), and a longer reaction time of 48 h. Purification by flash column chromatography (0-20% EtOAc in 40-60 °C petroleum ether) afforded the title compound as a yellow oil (10.1 mg, 0.036 mmol, 36%, 81% *ee*).

^{1}H NMR (700 MHz, $CDCl_3$) δ 8.24 (1H, dd, *J*=7.9, 1.5 Hz, H_5), 8.16 (2H, d, *J*=8.8 Hz, H_{14}), 7.48 (1H, app td, *J*=7.5, 1.5 Hz, H_3), 7.43 – 7.39 (3H, m, H_4 and H_{13}), 7.03 (1H, dd, *J*=8.0, 1.2 Hz, H_2), 6.87 (1H, d, *J*=10.0 Hz, H_9), 6.50 (1H, d, *J*=10.0 Hz, H_8), 1.95 (3H, s, H_{11}).

^{13}C NMR (176 MHz, $CDCl_3$) δ 184.7, 153.9, 150.9, 148.4, 147.1, 133.4, 130.7, 128.6, 128.3, 127.7, 127.1, 126.8, 124.1, 45.7, 27.3.

HRMS m/z: $[M+H]^+$ calculated for $[C_{17}H_{14}NO_3]^+$ 280.0968, found 280.0960. Δ = −2.9 ppm.

Chiral SFC Analysis: CHIRALPAK IE (CO_2:MeOH, 80:20, 2.5 mL min^{-1}, 40 °C, 260 nm) indicated 81% *ee*, t_R = 7.02 (minor), 7.58 (major) minutes.

$[\alpha]_D^{25}$ = +75.6° (c 0.67, $CHCl_3$).

(*R*)-4-Methyl-4-(4-(trifluoromethyl)phenyl)naphthalen-1(4*H*)-one (2w)

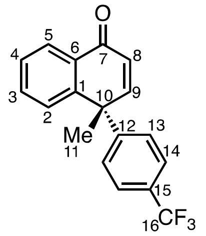

Prepared according to General Procedure B with 4-methylnaphthalen-1-ol (15.8 mg, 0.100 mmol) and 1-bromo-4-(trifluoromethyl)benzene (33.8 mg, 0.150 mmol) as the starting materials, double

the usual loadings of (*R*)-sSPhos (7.7 mg, 0.015 mmol, 15 mol%) and Pd_2dba_3 (2.3 mg, 0.0025 mmol, 2.5 mol%), and a longer reaction time of 48 h. Purification by flash column chromatography (0-6% EtOAc in hexane) afforded the title compound as a brown oil (18.3 mg, 0.061 mmol, 61% yield, 95% *ee*).

^{1}H NMR (500 MHz, $CDCl_3$) δ 8.23 (1H, dd, *J*=8.1, 1.5 Hz, H_5), 7.56 (2H, d, *J*=8.1 Hz, H_{14}), 7.47 (1H, ddd, *J*=7.8, 7.2, 1.5 Hz, H_3), 7.39 (1H, ddd, *J*=8.1, 7.2, 1.3 Hz, H_4), 7.36 (2H, d, *J*=8.1 Hz, H_{13}), 7.06 (1H, dd, *J*=7.8, 1.3 Hz, H_2), 6.88 (1H, d, *J*=10.2 Hz, H_9), 6.47 (1H, d, *J*=10.2 Hz, H_8), 1.92 (3H, s, H_{11}).

^{13}C NMR (176 MHz, $CDCl_3$) δ 184.9, 154.7, 149.0, 147.5 (q, *J*=1.3 Hz), 133.2, 130.8, 129.6 (q, *J*=32.6 Hz), 128.7, 127.7, 127.4, 126.9, 126.4, 125.9 (q, *J*=3.7 Hz), 124.1 (q, *J*=272.1 Hz), 45.6, 27.3.

^{19}F NMR (471 MHz, $CDCl_3$) δ −62.6.

HRMS m/z: $[M+H]^+$ calculated for $[C_{18}H_{14}F_3O]^+$ 303.0991, found 303.0994. Δ = +1.0 ppm.

Chiral SFC Analysis: CHIRALPAK IE (CO_2:MeOH, 98:02, 2.5 mL min^{-1}, 40 °C, 250 nm) indicated 95% *ee*, t_R = 11.54 (major), 12.70 (minor) minutes.

$[\alpha]_D^{25}$ = +58.9° (c 1.22, $CHCl_3$).

(*R*)-4-([1,1'-biphenyl]-4-yl)-4-Methylnaphthalen-1(4*H*)-one (2x)

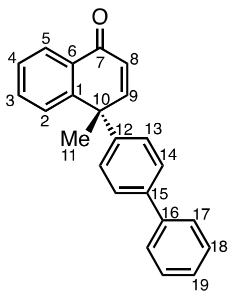

Prepared according to General Procedure B with 4-methylnaphthalen-1-ol (15.8 mg, 0.100 mmol) and 4-bromo-1,1'-biphenyl (35.0 mg, 0.150 mmol) as the starting materials. Purification by flash column chromatography (0-4% EtOAc in 40-60 °C petroleum ether) afforded the title compound as a colorless solid (23.2 mg, 0.075 mmol, 75%, 97% *ee*).

^{1}H NMR (400 MHz, $CDCl_3$) δ 8.24 (1H, dd, *J*=7.8, 1.5 Hz, H_5), 7.59 – 7.50 (4H, m, H_{14} and $H_{17/18}$), 7.50 – 7.28 (7H, m, H_3, H_4, H_{13}, $H_{17/18,}$ and H_{19}), 7.17 (1H, dd, *J*=7.9, 1.3 Hz, H_2), 6.96 (1H, d, *J*=10.1 Hz, H_9), 6.47 (1H, d, *J*=10.1 Hz, H_8), 1.93 (3H, s, H_{11}).

^{13}C NMR (101 MHz, $CDCl_3$) δ 185.3, 155.9, 149.9, 142.2, 140.5, 140.2, 133.0, 130.8, 128.9, 128.8, 127.6, 127.6 (2×), 127.1, 127.0, 126.8, 125.9, 45.4, 27.3.

HRMS m/z: $[M+H]^+$ calculated for $[C_{23}H_{19}O]^+$ 311.1430, found 311.1430. Δ = 0.0 ppm.

Chiral SFC Analysis: CHIRALPAK IK (CO_2:MeOH, 80:20, 2.5 mL min^{-1}, 40 °C, 250 nm) indicated 97% *ee*, t_R = 7.36 (minor), 8.68 (major) minutes.

$[\alpha]_D^{25}$ = +157° (c 1.55, $CHCl_3$).

(*R*)-1-Methyl-[1,2'-binaphthalen]-4(1*H*)-one (2y)

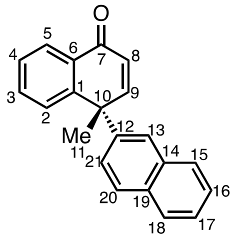

Prepared according to General Procedure B with 4-methylnaphthalen-1-ol (15.8 mg, 0.100 mmol) and 2-bromonaphthalene (31.1 mg, 0.150 mmol) as the starting materials. Purification by flash column chromatography (0-5% EtOAc in 40-60 °C petroleum ether) afforded the title compound as a yellow solid (22.4 mg, 0.079 mmol, 79% yield, 97% *ee*).

^{1}H NMR (400 MHz, $CDCl_3$) δ 8.26 (1H, d, *J*=7.7 Hz, H_5), 7.92 (1H, s, H_{13}), 7.86 (1H, d, *J*=7.8 Hz, $H_{15/18}$), 7.79 (1H, d, *J*=7.8 Hz, $H_{15/18}$), 7.69 (1H, d, *J*=8.6 Hz, H_{20}), 7.54 – 7.46 (2H, m, H_{16} and H_{17}), 7.39 (2H, m, H_3 and H_4), 7.12 (1H, d, *J*=7.4 Hz, H_2), 7.07 (1H, d, *J*=8.6 Hz, H_{21}), 6.97 (1H, d, *J*=10.0 Hz, H_9), 6.50 (1H, d, *J*=10.0 Hz, H_8), 2.01 (3H, s, H_{11}).

^{13}C NMR (101 MHz, $CDCl_3$) δ 185.3, 155.8, 149.8, 140.6, 133.5, 133.0, 132.4, 130.9, 128.9, 128.6, 128.1, 127.7, 127.1, 126.8, 126.6, 126.4, 126.2 (2×), 124.9, 45.8, 27.3.

HRMS m/z: $[M+H]^+$ calculated for $[C_{21}H_{17}O]^+$ 285.1274, found 285.1274. Δ = 0.0 ppm.

Chiral SFC Analysis: CHIRALPAK IE (CO_2:MeOH, 90:10, 2.5 mL min^{-1}, 40 °C, 250 nm) indicated 97% *ee*, t_R = 12.28 (minor), 14.33 (major) minutes.

[α]D[25] = +91.3° (c 1.49, $CHCl_3$).

(*R*)-4-(3-(*tert*-butyl)phenyl)-4-Methylnaphthalen-1(4*H*)-one (2z)

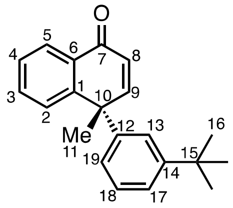

Prepared according to General Procedure B with 4-methylnaphthalen-1-ol (15.8 mg, 0.100 mmol) and 1-bromo-3-(*tert*-butyl)benzene (32.0 mg, 0.150 mmol) as the starting materials. Purification by flash column chromatography (0-3% EtOAc in 40-60 °C petroleum ether) afforded the title compound as a yellow oil (14.0 mg, 0.048 mmol, 48% yield, 98% *ee*).

^{1}H NMR (500 MHz, $CDCl_3$) δ 8.22 (1H, ddd, *J*=7.8, 1.6, 0.6 Hz, H_5), 7.44 (1H, ddd, *J*=7.9, 7.2, 1.6 Hz, H_3), 7.36 (1H, ddd, *J*=7.8, 7.2, 1.2 Hz, H_4), 7.30 – 7.20 (3H, m, H_{13}, H_{17}, and H_{18}), 7.12 (1H, ddd, *J*=7.9, 1.2, 0.6 Hz, H_2), 7.04 (1H, ddd, *J*=7.6, 1.9, 1.2 Hz, H_{19}), 6.95 (1H, d, *J*=10.0 Hz, H_9), 6.44 (1H, d, *J*=10.0 Hz, H_8), 1.89 (3H, s, H_{11}), 1.26 (9H, s, H_{16}).

^{13}C NMR (126 MHz, $CDCl_3$) δ 185.4, 156.4, 151.8, 150.2, 142.9, 132.9, 130.8, 128.8, 128.5, 126.9, 126.7, 125.7, 124.4, 124.2, 124.0, 45.9, 35.0, 31.4, 27.4.

HRMS m/z: $[M+H]^+$ calculated for $[C_{21}H_{23}O]^+$ 291.1743, found 291.1747. Δ = +1.4 ppm.

Chiral SFC Analysis: CHIRALPAK IC (CO_2:MeOH, 95:05, 2.5 mL min^{-1}, 40 °C, 250 nm) indicated 98% *ee*, t_R = 6.44 (major), 6.78 (minor) minutes.

[α]D[25] = +102° (c 0.93, $CHCl_3$).

(*R*)-4-(4-(*tert*-butyl)phenyl)-4-Methylnaphthalen-1(4*H*)-one (2aa)

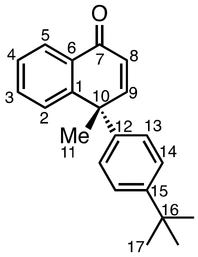

Prepared according to General Procedure B with 4-methylnaphthalen-1-ol (15.8 mg, 0.100 mmol) and 1-bromo-4-(*tert*-butyl)benzene (32.0 mg, 0.150 mmol) as the starting materials. Purification by flash column chromatography (0-3% EtOAc in 40-60 °C petroleum ether) afforded the title compound as a yellow oil (15.7 mg, 0.054 mmol, 54% yield, 99% *ee*).

^{1}H NMR (500 MHz, $CDCl_3$) δ 8.21 (1H, ddd, *J*=8.1, 1.6, 0.5 Hz, H_5), 7.44 (1H, ddd, *J*=7.9, 7.2, 1.6 Hz, H_3), 7.35 (1H, ddd, *J*=8.1, 7.2, 1.2 Hz, H_4), 7.33 – 7.28 (2H, m, H_{14}), 7.17 – 7.13 (3H, m, H_2 and H_{13}), 6.93 (1H, d, *J*=10.1 Hz, H_9), 6.42 (1H, d, *J*=10.1 Hz, H_8), 1.88 (3H, s, H_{11}), 1.29 (9H, s, H_{17}).

^{13}C NMR (126 MHz, $CDCl_3$) δ 185.4, 156.4, 150.1 (2×), 140.1, 132.9, 130.8, 128.9, 126.9, 126.8, 126.7, 125.8, 125.6, 45.3, 34.5, 31.4, 27.3.

HRMS m/z: $[M+H]^+$ calculated for $[C_{21}H_{23}O]^+$ 291.1743, found 291.1744. Δ = +0.3 ppm.

Chiral SFC Analysis: CHIRALPAK IE (CO_2:MeOH, 95:05, 2.5 mL min^{-1}, 40 °C, 250 nm) indicated 99% *ee*, t_R = 10.40 (minor), 11.41 (major) minutes.

$[\alpha]_D^{25}$ = +130° (c 1.05, $CHCl_3$).

(*R*)-1-Methyl-[1,1'-binaphthalen]-4(1*H*)-one (2ab)

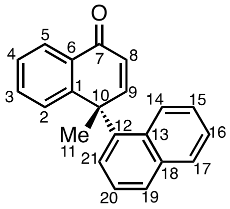

Prepared according to General Procedure B with 4-methylnaphthalen-1-ol (15.8 mg, 0.100 mmol) and 1-bromonaphthalene (31.1 mg, 0.150 mmol) as the starting materials, double the usual loadings of (*R*)-sSPhos (7.7 mg, 0.015 mmol, 15 mol%) and Pd_2dba_3 (2.3 mg, 0.0025 mmol, 2.5 mol%), and a longer reaction time of 48 h. Purification by flash column chromatography (0-4% EtOAc in 40-60 °C petroleum ether) afforded the title compound as a yellow solid (8.3 mg, 0.029 mmol, 29% yield, 96% *ee*).

^{1}H NMR (500 MHz, $CDCl_3$) δ 8.32 (1H, dd, *J*=7.9, 1.6 Hz, H_5), 7.90 – 7.84 (2H, m, H_{19} and H_{21}), 7.81 (1H, d, *J*=8.5 Hz, H_{17}), 7.59 (1H, app t, *J*=7.8 Hz, H_{20}), 7.38 – 7.27 (4H, m, H_4, H_3, H_{14}, and H_{16}), 7.17 – 7.08 (2H, m, H_9 and H_{15}), 6.88 (1H, d, *J*=7.8 Hz, H_2), 6.58 (1H, d, *J*=10.1 Hz, H_8), 1.94 (3H, s, H_{11}).

^{13}C NMR (126 MHz, $CDCl_3$) δ 185.0, 158.1, 151.6, 138.4, 134.8, 133.3, 131.6, 130.8, 129.5, 129.0, 127.4, 127.2, 127.1, 127.0, 126.1, 125.7, 125.4, 125.2, 125.1, 46.5, 33.2.

HRMS m/z: $[M+H]^+$ calculated for $[C_{21}H_{17}O]^+$ 285.1274, found 285.1271. Δ = −1.1 ppm.

Chiral SFC Analysis: CHIRALPAK IE (CO_2:MeOH, 90:10, 2.5 mL min^{-1}, 40 °C, 260 nm) indicated 96% *ee*, t_R = 9.31 (minor), 9.77 (major) minutes.

$[\alpha]_D^{25}$ = +42.3° (c 0.55, $CHCl_3$).

(*R*)-3-(4-(1-methyl-4-oxo-1,4-dihydronaphthalen-1-yl)phenyl)Propanenitrile (2ac)

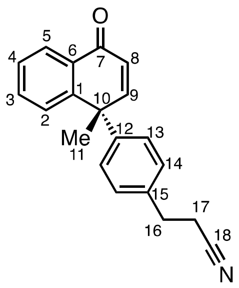

Prepared according to General Procedure B with 4-methylnaphthalen-1-ol (15.8 mg, 0.100 mmol) and 3-(4-bromophenyl)propanenitrile (31.5 mg, 0.150 mmol) as the starting materials, double the usual loadings of (*R*)-sSPhos (7.7 mg, 0.015 mmol, 15 mol%) and Pd_2dba_3 (2.3 mg, 0.0025 mmol, 2.5 mol%), and a longer reaction time of 48 h. Purification by flash column chromatography (0-30% EtOAc in heptane) afforded the title compound as an orange solid (14.9 mg, 0.052 mmol, 52% yield, 90% *ee*).

^{1}H NMR (500 MHz, $CDCl_3$) δ 8.21 (1H, ddd, *J*=7.8, 1.5, 0.6 Hz, H_5), 7.45 (1H, ddd, *J*=7.9, 7.2, 1.5 Hz, H_3), 7.36 (1H, ddd, *J*=7.8, 7.2, 1.2 Hz, H_4), 7.23 – 7.15 (4H, m, H_{13} and H_{14}), 7.10 (1H, ddd, *J*=7.9, 1.2, 0.6 Hz, H_2), 6.90 (1H, d, *J*=10.0 Hz, H_9), 6.43 (1H, d, *J*=10.0 Hz, H_8), 2.92 (2H, app t, *J*=7.3 Hz, H_{16}), 2.60 (2H, app t, *J*=7.3 Hz, H_{17}), 1.88 (3H, s, H_{11}).

^{13}C NMR (126 MHz, $CDCl_3$) δ 185.2, 155.8, 149.8, 142.4, 137.0, 133.0, 130.8, 128.9, 128.8, 127.7, 127.0, 126.8, 125.9, 119.1, 45.4, 31.1, 27.3, 19.4.

HRMS m/z: $[M+H]^+$ calculated for $[C_{20}H_{18}NO]^+$ 288.1383, found 288.1378. Δ = −1.7 ppm.

Chiral SFC Analysis: CHIRALPAK AD (CO_2 (A):0.1% NH_3 in MeOH (B), 5% B – 50% B over two minutes, then isocratic 50% B for 2 minutes, 2.5 mL min^{-1}, 40 °C, 220 nm) indicated 90% *ee*, t_R = 1.24 (minor), 1.38 (major) minutes.

[α]$_D^{25}$ = +105° (c 0.75, $CHCl_3$).

(*R*)-4-(benzofuran-5-yl)-4-Methylnaphthalen-1(4*H*)-one (2ad)

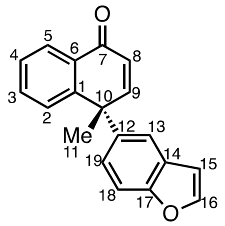

Prepared according to General Procedure B with 4-methylnaphthalen-1-ol (15.8 mg, 0.100 mmol) and 5-bromobenzofuran (29.6 mg, 0.150 mmol) as the starting materials, double the usual loadings of (*R*)-sSPhos (7.7 mg, 0.015 mmol, 15 mol%) and Pd_2dba_3 (2.3 mg, 0.0025 mmol, 2.5 mol%), and a longer reaction time of 48 h. Purification by flash column chromatography (0-20% EtOAc in heptane) afforded the title compound as a yellow solid (25.4 mg, 0.093 mmol, 93% yield, 93% *ee*).

^{1}H NMR (500 MHz, $CDCl_3$) δ 8.23 (1H, ddd, *J*=8.1, 1.6, 0.6 Hz, H_5), 7.62 (1H, d, *J*=2.2 Hz, H_{16}), 7.57 (1H, d, *J*=1.9 Hz, H_{13}), 7.42 (1H, ddd, *J*=7.9, 7.2, 1.6 Hz, H_3), 7.39 (1H, app dt, *J*=8.7, 0.9 Hz, H_{18}), 7.35 (1H, ddd, *J*=8.1, 7.2, 1.3 Hz, H_4), 7.11 (1H, ddd, *J*=7.9, 1.3, 0.6 Hz, H_2), 7.07 (1H, dd, *J*=8.7, 1.9 Hz, H_{19}), 6.95 (1H, d, *J*=10.0 Hz, H_9), 6.74 (1H, dd, *J*=2.2, 0.9 Hz, H_{15}), 6.44 (1H, d, *J*=10.0 Hz, H_8), 1.95 (3H, s, H_{11}).

^{13}C NMR (126 MHz, $CDCl_3$) δ 185.3, 156.4, 154.0, 150.4, 145.9, 137.8, 132.9, 130.7, 128.9, 127.8, 126.9, 126.7, 125.5, 124.1, 119.4, 111.7, 106.8, 45.6, 27.7.

HRMS m/z: $[M+H]^+$ calculated for $[C_{19}H_{15}O_2]^+$ 275.1067, found 275.1063. Δ = −1.5 ppm.

Chiral SFC Analysis: CHIRALPAK AD (CO_2 (A):0.1% NH_3 in MeOH (B), 5% B – 50% B over two minutes, then isocratic 50% B for 2 minutes, 2.5 mL min^{-1}, 40 °C, 250 nm) indicated 93% *ee*, t_R = 1.06 (minor), 1.23 (major) minutes.

[α]$_D^{25}$ = +97.9° (c 1.27, $CHCl_3$).

(*R*)-4-(1*H*-indol-6-yl)-4-Methylnaphthalen-1(4*H*)-one (2ae)

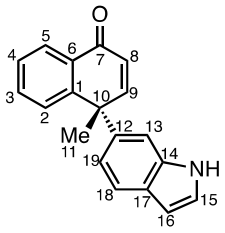

Prepared according to General Procedure B with 4-methylnaphthalen-1-ol (15.8 mg, 0.100 mmol) and 6-bromo-1*H*-indole (29.4 mg, 0.150 mmol) as the starting materials, double the usual loadings of (*R*)-sSPhos (7.7 mg, 0.015 mmol, 15 mol%) and Pd_2dba_3 (2.3 mg, 0.0025 mmol, 2.5 mol%), and a longer reaction time of 48 h. Purification by flash column chromatography (0-30% EtOAc in heptane) afforded the title compound as a brown solid (20.0 mg, 0.073 mmol, 73% yield, 85% *ee*).

^{1}H NMR (500 MHz, $CDCl_3$) δ 8.26 (1H, br s, H_N), 8.22 (1H, dd, *J*=8.2, 1.5 Hz, H_5), 7.54 (1H, d, *J*=8.3 Hz, H_{18}), 7.40 (1H, ddd, *J*=7.8, 7.2, 1.5 Hz, H_3), 7.34 (1H, ddd, *J*=8.2, 7.2, 1.3 Hz, H_4), 7.32 – 7.29 (1H, m, H_{13}), 7.21 (1H, dd, *J*=3.1, 2.4 Hz, H_{15}), 7.15 (1H, dd, *J*=7.8, 1.3 Hz, H_2), 6.98 (1H, d, *J*=10.0 Hz, H_9), 6.95 (1H, dd, *J*=8.3, 1.7 Hz, H_{19}), 6.51 (1H, ddd, *J*=3.1, 2.0, 0.9 Hz, H_{16}), 6.42 (1H, d, *J*=10.0 Hz, H_8), 1.94 (3H, s, H_{11}).

^{13}C NMR (126 MHz, $CDCl_3$) δ 185.7, 156.9, 150.8, 136.9, 136.1, 132.9, 130.7, 129.0, 126.9, 126.8, 126.6, 125.2, 125.1, 121.0, 119.5, 109.7, 102.5, 45.7, 27.5.

HRMS m/z: $[M+H]^+$ calculated for $[C_{19}H_{16}NO]^+$ 274.1226, found 274.1219. Δ = −2.6 ppm.

Chiral SFC Analysis: CHIRALPAK AD (CO_2 (A):0.1% NH_3 in MeOH (B), 5% B – 50% B over two minutes, then isocratic 50% B for 2 minutes, 2.5 mL min^{-1}, 40 °C, 230 nm) indicated 85% *ee*, t_R = 1.46 (minor), 1.56 (major) minutes.

$[\alpha]_D^{25}$ = +169° (c 1.00, $CHCl_3$).

(*R*)-4-(1*H*-indol-5-yl)-4-Methylnaphthalen-1(4*H*)-one (2af)

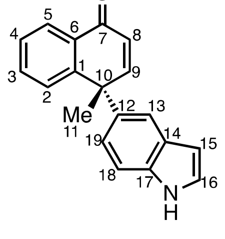

Prepared according to General Procedure B with 4-methylnaphthalen-1-ol (15.8 mg, 0.100 mmol) and 5-bromo-1*H*-indole (29.4 mg, 0.150 mmol) as the starting materials, double the usual loadings of (*R*)-sSPhos (7.7 mg, 0.015 mmol, 15 mol%) and Pd_2dba_3 (2.3 mg, 0.0025 mmol, 2.5 mol%), and a longer reaction time of 48 h. Purification by flash column chromatography (0-20% EtOAc in hexane) afforded the title compound as a yellow solid (11.0 mg, 0.040 mmol, 40% yield, 97% *ee*).

^{1}H NMR (700 MHz, $CDCl_3$) δ 8.26 – 8.19 (2H, m, H_5 and H_N), 7.69 (1H, s, H_{13}), 7.40 (1H, app t, *J*=7.7 Hz, H_3), 7.33 (1H, app t, *J*=7.7 Hz, H_4), 7.27 – 7.25 (1H, m, H_{18}), 7.24 – 7.21 (1H, m, H_{16}), 7.15 (1H, d, *J*=7.7 Hz, H_2), 6.99 (1H, d, *J*=10.0 Hz, H_9), 6.87 (1H, d, *J*=8.6 Hz, H_{19}), 6.56 – 6.54 (1H, m, H_{15}), 6.43 (1H, d, *J*=10.0 Hz, H_8), 1.95 (3H, s, H_{11}).

^{13}C NMR (176 MHz, $CDCl_3$) δ 185.7, 157.2, 151.0, 134.8, 134.4, 132.8, 130.7, 129.1, 128.1, 126.7, 126.6, 125.2, 125.0, 122.3, 118.5, 111.5, 103.0, 45.7, 27.6.

HRMS m/z: $[M+H]^+$ calculated for $[C_{19}H_{16}NO]^+$ 274.1226, found 274.1222. Δ = −1.5 ppm.

Chiral SFC Analysis: CHIRALPAK IE (CO_2:MeOH, 80:20, 2.5 mL min^{-1}, 40 °C, 270 nm) indicated 97% *ee*, t_R = 5.79 (minor), 9.52 (major) minutes.

$[\alpha]_D^{25}$ = +98.9° (c 0.73, $CHCl_3$).

(*S*)-4-Methyl-4-(thiophen-3-yl)naphthalen-1(4*H*)-one (2ag)

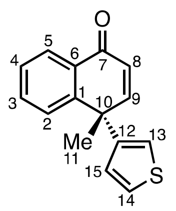

Prepared according to General Procedure B with 4-methylnaphthalen-1-ol (15.8 mg, 0.100 mmol) and 3-bromothiophene (24.5 mg, 0.150 mmol) as the starting materials, double the usual loadings of (*R*)-sSPhos (7.7 mg, 0.015 mmol, 15 mol%) and Pd_2dba_3 (2.3 mg, 0.0025 mmol, 2.5 mol%), and a reaction time of 48 h. Purification by preparatory TLC (10% acetone in toluene and 25% EtOAc in hexane) afforded the title compound as a yellow solid (3.9 mg, 0.016 mmol, 16% yield, 96% *ee*).

^{1}H NMR (700 MHz, $CDCl_3$) δ 8.20 (1H, d, *J*=7.5 Hz, H_5), 7.48 (1H, app t, *J*=7.5 Hz, H_3), 7.38 (1H, app t, *J*=7.5 Hz, H_4), 7.24 – 7.20 (3H, m, H_2, $H_{13,}$ and H_{15}), 6.92 (1H, d, *J*=10.0 Hz, H_9), 6.66 (1H, d, *J*=4.9 Hz, H_{14}), 6.42 (1H, d, *J*=10.0 Hz, H_8), 1.88 (3H, s, H_{11}).

^{13}C NMR (176 MHz, $CDCl_3$) δ 185.1, 155.2, 148.8, 144.7, 132.9, 130.5, 128.4, 127.4, 127.2, 126.8, 126.6, 126.0, 121.0, 43.4, 28.2.

HRMS m/z: $[M+H]^+$ calculated for $[C_{15}H_{13}OS]^+$ 241.0682, found 241.0679. Δ = −1.2 ppm.

Chiral SFC Analysis: CHIRALPAK IE (CO_2:MeOH, 90:10, 2.5 mL min^{-1}, 40 °C, 250 nm) indicated 96% *ee*, t_R = 6.06 (minor), 6.47 (major) minutes.

$[\alpha]_D^{25}$ = +114° (c 0.26, $CHCl_3$).

(*R*)-4-(cyclohex-1-en-1-yl)-4-Methylnaphthalen-1(4*H*)-one (2al)

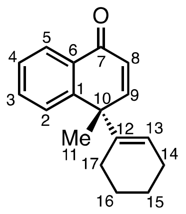

Prepared according to General Procedure B with 4-methylnaphthalen-1-ol (15.8 mg, 0.100 mmol) and cyclohex-1-en-1-yl trifluoromethanesulfonate (34.5 mg, 0.150 mmol) as the starting materials. Purification by flash column chromatography (0-10% EtOAc in heptane) afforded the title compound as a yellow oil (4.6 mg, 0.019 mmol, 19% yield, 98% *ee*).

^{1}H NMR (500 MHz, $CDCl_3$) δ 8.16 (1H, ddd, *J*=7.8, 1.5 Hz, H_5), 7.51 (1H, ddd, *J*=7.8, 7.2, 1.5 Hz, H_3), 7.36 (1H, ddd, *J*=7.8, 7.2, 1.2 Hz, H_4), 7.32 (1H, dd, *J*=7.8, 1.2 Hz, H_2), 6.74 (1H, d, *J*=10.1 Hz, H_9), 6.45 (1H, d, *J*=10.1 Hz, H_8), 6.00 (1H, app tt, *J*=4.0, 1.6 Hz, H_{13}), 2.25 – 2.07 (2H, m, H_{14}), 1.69 – 1.22 (9H, m, H_{11}, H_{15}, H_{16}, and H_{17}).

^{13}C NMR (126 MHz, $CDCl_3$) δ 185.4, 156.5, 148.8, 139.2, 132.7, 131.8, 127.9, 127.3, 126.9, 126.7, 123.2, 47.1, 26.5, 25.9, 25.8, 23.1, 22.3.

HRMS m/z: $[M+H]^+$ calculated for $[C_{17}H_{19}O]^+$ 239.1430, found 239.1426. Δ = −1.7 ppm.

Chiral SFC Analysis: CHIRALPAK AD (CO_2 (A):0.1% NH_3 in MeOH (B), 5% B – 50% B over two minutes, then isocratic 50% B for 2 minutes, 2.5 mL min^{-1}, 40 °C, 250 nm) indicated 98% *ee*, t_R = 0.82 (minor), 0.95 (major) minutes.

[α]$_D^{25}$ = +94.2° (c 0.42, $CHCl_3$).

(*S*,*E*)-4-Methyl-4-styrylnaphthalen-1(4*H*)-one (2am)

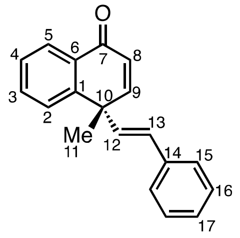

[N.B. The ^{1}H NMR spectrum for the isolated sample revealed a small inseparable unidentified impurity (see spectrum). Signals corresponding to the impurity integrated to 0.12 compared to 1 for the product, implying a mol/mol impurity of 11%.]

Prepared according to General Procedure B with 4-methylnaphthalen-1-ol (15.8 mg, 0.100 mmol) and (*E*)-(2-bromovinyl)benzene (27.5 mg, 0.150 mmol) as the starting materials, double the usual loadings of (*R*)-sSPhos (7.7 mg, 0.015 mmol, 15 mol%) and Pd_2dba_3 (2.3 mg, 0.0025 mmol, 2.5 mol%), and a longer reaction time of 48 h. Purification by flash column chromatography (0-5% EtOAc in hexane) afforded the title compound as a yellow oil (13.2 mg, 0.051 mmol [not accounting for the impurity], 51% yield [not accounting for the impurity], 98% *ee*).

^{1}H NMR (500 MHz, $CDCl_3$) δ 8.06 (1H, app dt, *J*=7.9, 1.1 Hz, H_5), 7.44 – 7.39 (2H, m, H_2 and H_3), 7.31 – 7.26 (1H, m, H_4), 7.05 – 6.99 (1H, m, H_{17}), 6.98 – 6.93 (2H, m, H_{16}), 6.81 (1H, d, *J*=10.1 Hz, H_9), 6.68 – 6.61 (3H, m, H_{13} and H_{15}), 6.13 (1H, d, *J*=10.1 Hz, H_8), 6.08 (1H, d, *J*=11.9 Hz, H_{12}), 1.56 (3H, s, H_{11}).

^{13}C NMR (126 MHz, $CDCl_3$) δ 185.0, 154.8, 149.7, 136.2, 135.6, 132.8, 132.6, 130.6, 128.4, 127.7, 127.5, 127.2, 126.8, 126.7, 126.0, 42.8, 33.4.

HRMS m/z: $[M+H]^+$ calculated for $[C_{19}H_{17}O]^+$ 261.1274, found 261.1273. Δ = −0.4 ppm.

Chiral SFC Analysis: CHIRALPAK IJ (CO_2:MeOH, 97:03, 2.5 mL min^{-1}, 40 °C, 250 nm) indicated 98% *ee*, t_R = 4.64 (major), 5.02 (minor) minutes.

[α]$_D^{25}$ = +285° (c 0.50, $CHCl_3$).

(*R*)-4-(9,9-dibutyl-9*H*-fluoren-2-yl)-4-Methylnaphthalen-1(4*H*)-one (2an)

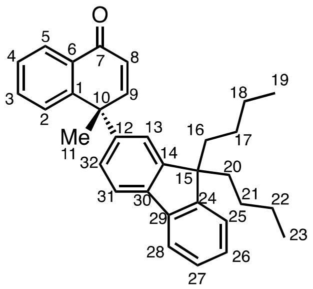

Prepared according to General Procedure B with 4-methylnaphthalen-1-ol (15.8 mg, 0.100 mmol) and 2-bromo-9,9-dibutyl-9*H*-fluorene (53.6 mg, 0.150 mmol) as the starting materials, and a longer reaction time of 48 h. Purification by flash column chromatography (0-10% EtOAc in heptane) afforded the title compound as a yellow oil (30.9 mg, 0.071 mmol, 71%, 95% *ee*).

^{1}H NMR (500 MHz, $CDCl_3$) δ 8.23 (1H, dd, *J*=8.1, 1.5 Hz, H_5), 7.68 – 7.64 (1H, m, H_{28}), 7.62 (1H, d, *J*=7.9 Hz, H_{31}), 7.44 – 7.37 (1H, m, H_3), 7.35 (1H, ddd, *J*=8.1, 7.2, 1.2 Hz, H_4), 7.33 – 7.28 (3H, m, H_{25}, H_{26}, and H_{27}), 7.23 (1H, dd, *J*=7.9, 1.5 Hz, H_{32}), 7.13 (1H, d, *J*=1.5 Hz, H_{13}), 7.09 (1H, dd, *J*=8.0, 1.2 Hz, H_2), 7.01 (1H, d, *J*=10.0 Hz, H_9), 6.48 (1H, d, *J*=10.0 Hz, H_8), 2.01 – 1.73 (7H, m, H_{11}, H_{16} and H_{20}), 1.14 – 1.03 (2H, m, $H_{18/22}$), 1.01 – 0.85 (2H, m, $H_{18/22}$), 0.69 (3H, app t, *J*=7.4 Hz, $H_{19/23}$), 0.65 – 0.46 (6H, m, $H_{19/23}$ and 3×$H_{17/21}$), 0.45 – 0.34 (1H, m, $H_{17/21}$).

^{13}C NMR (126 MHz, $CDCl_3$) δ 185.5, 156.3, 151.4, 151.0, 150.5, 142.0, 140.5, 140.4, 132.8, 130.8, 128.8, 127.3, 126.9, 126.9, 126.7, 125.7, 125.7, 123.0, 121.9, 119.9, 119.8, 55.1, 45.9, 40.0, 39.9, 27.6, 26.1, 26.0, 23.1, 22.9, 14.0, 13.8.

HRMS m/z: $[M+H]^+$ calculated for $[C_{32}H_{35}O]^+$ 435.2682, found 435.2679. Δ = −0.7 ppm.

Chiral SFC Analysis: CHIRALPAK IA (CO_2 (A):0.1% DEA in IPA (B), 5% B – 50% B over two minutes, then isocratic 50% B for 2 minutes, 2.5 mL min^{-1}, 40 °C, 250 nm) indicated 95% *ee*, t_R = 1.13 (minor), 1.20 (major) minutes.

$[\alpha]_D^{25}$ = +108° (c 1.03, $CHCl_3$).

Benzyl (*R*)-4-(4-(1-methyl-4-oxo-1,4-dihydronaphthalen-1-yl)phenyl)Piperazine-1-carboxylate (2ao)

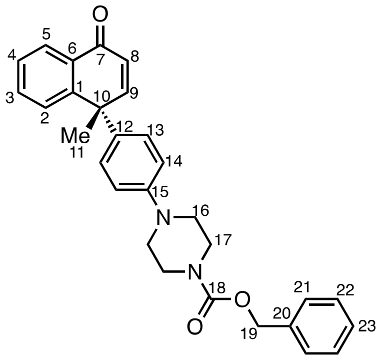

Prepared according to General Procedure B with 4-methylnaphthalen-1-ol (15.8 mg, 0.100 mmol) and benzyl 4-(4-bromophenyl)piperazine-1-carboxylate (56.3 mg, 0.150 mmol) as the starting materials. Purification by flash column chromatography (0-30% EtOAc in heptane) afforded the title compound as a yellow solid (26.5 mg, 0.059 mmol, 59% yield, 96% *ee*).

^{1}H NMR (700 MHz, $CDCl_3$) δ 8.19 (dd, *J* = 7.9, 1.5 Hz, 1H, H_5), 7.43 (ddd, *J* = 8.0, 7.2, 1.5 Hz, 1H, H_3), 7.39 – 7.29 (m, 6H, H_4, H_{21}, H_{22} and H_{23}), 7.14 – 7.09 (m, 3H, H_2 and H_{13}), 6.89 (d, *J* = 10.0 Hz, 1H, H_9), 6.83 (d, *J* = 8.9 Hz, 1H, H_{14}), 6.40 (d, *J* = 10.0 Hz, 1H, H_8), 5.15 (s, 2H, H_{19}), 3.66 – 3.62 (m, 4H, H_{17}), 3.12 (app br s, 4H, H_{16}), 1.85 (s, 3H, H_{11}).

^{13}C NMR (176 MHz, $CDCl_3$) δ 185.4, 156.4, 155.3, 150.3, 150.1, 136.7, 134.5, 132.8, 130.8, 128.8, 128.7, 128.3, 128.1, 128.0, 126.8, 126.7, 125.5, 116.6, 67.4, 49.1, 45.0, 43.8, 27.3.

HRMS m/z: $[M+H]^+$ calculated for $[C_{29}H_{29}N_2O_3]^+$ 453.2173, found 453.2169. Δ = −0.9 ppm.

Chiral SFC Analysis: CHIRALPAK IA (CO_2 (A):0.1% DEA in IPA (B), 5% B – 50% B over two minutes, then isocratic 50% B for 2 minutes, 2.5 mL min^{-1}, 40 °C, 250 nm) indicated 96% *ee*, t_R = 2.14 (minor), 2.29 (major) minutes.

$[\alpha]_D^{25}$ = +105° (c 1.35, $CHCl_3$).

(*R*)-4-(4-(2,6-diphenylpyrimidin-4-yl)phenyl)-4-Methylnaphthalen-1(4*H*)-one (2ap)

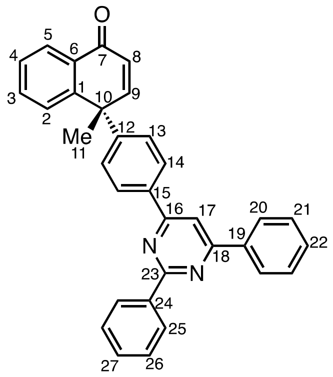

Prepared according to General Procedure B with 4-methylnaphthalen-1-ol (15.8 mg, 0.100 mmol) and 4-(4-bromophenyl)-2,6-diphenylpyrimidine (51.8 mg, 0.150 mmol) as the starting materials, double the usual loadings of (*R*)-sSPhos (7.7 mg, 0.015 mmol, 15 mol%) and Pd_2dba_3 (2.3 mg, 0.0025 mmol, 2.5 mol%), and a longer reaction time of 48 h. Purification by flash column chromatography (0-30% EtOAc in heptane) afforded the title compound as a brown solid (39.6 mg, 0.085 mmol, 85% yield, 89% *ee*).

^{1}H NMR (500 MHz, $CDCl_3$) δ 8.73 – 8.69 (2H, m, H_{25}), 8.29 – 8.24 (3H, m, H_{13} and $H_{22/27}$), 8.23 – 8.20 (2H, m, H_5 and $H_{22/27}$), 7.96 (1H, s, H_{17}), 7.59 – 7.50 (6H, m, H_{20}, H_{21} and H_{26}), 7.47 (1H, app td, *J*=7.7, 1.6 Hz, H_3), 7.44 – 7.37 (3H, m, H_4 and H_{14}), 7.15 (1H, dd, *J*=7.7, 1.2 Hz, H_2), 6.97 (1H, d, *J*=10.0 Hz, H_9), 6.50 (1H, d, *J*=10.0 Hz, H_8), 1.97 (3H, s, H_{11}).

^{13}C NMR (126 MHz, $CDCl_3$) δ 185.1, 165.0, 164.7, 164.2, 155.3, 149.5, 146.1, 138.2, 137.5, 136.7, 133.1, 131.0, 130.8 (2×), 129.1, 128.8, 128.6, 128.6, 127.9, 127.8, 127.4, 127.2, 126.9, 126.2, 110.3, 45.7, 27.3.

HRMS m/z: $[M+H]^+$ calculated for $[C_{33}H_{25}N_2O]^+$ 465.1961, found 465.1961. Δ = 0.0 ppm.

Chiral SFC Analysis: CHIRALPAK IA (CO_2 (A):0.1% NH_3 in MeOH (B), isocratic 50% B, 2.5 mL min^{-1}, 40 °C, 254 nm) indicated 89% *ee*, t_R = 2.05 (minor), 2.61 (major) minutes.

$[\alpha]_D^{25}$ = +88.8° (c 1.28, $CHCl_3$).

(*R*)-4-(4-chloro-3-(4-(((*R*)-tetrahydrofuran-3-yl)oxy)benzyl)phenyl)-4-Methylnaphthalen-1(4*H*)-one (2aq)

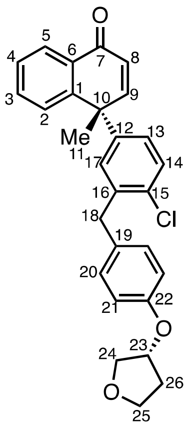

Prepared according to General Procedure B with 4-methylnaphthalen-1-ol (15.8 mg, 0.100 mmol) and (*R*)-3-(4-(5-bromo-2-chlorobenzyl)phenoxy)tetrahydrofuran (55.2 mg, 0.150 mmol) as the starting materials, double the usual loadings of (*R*)-sSPhos (7.7 mg, 0.015 mmol, 15 mol%) and Pd_2dba_3 (2.3 mg, 0.0025 mmol, 2.5 mol%), and a longer reaction time of 48 h. Purification by flash column chromatography (0-20% EtOAc in heptane) afforded the title compound as a yellow solid (26.5 mg, 0.060 mmol, 60% yield, 25:1 dr).

[N.B. The product was isolated as an inseparable mixture of diastereomers. Diastereomers displayed identical signals by ^1H and ^{13}C NMR. dr was determined by chiral SFC from the isolated sample.]

^{1}H NMR (500 MHz, $CDCl_3$) δ 8.19 (1H, dd, *J*=8.1, 1.5 Hz, H_5), 7.44 (1H, ddd, *J*=7.8, 7.2, 1.5 Hz, H_3), 7.36 (1H, ddd, *J*=8.1, 7.2, 1.2 Hz, H_4), 7.28 (1H, dd, *J*=7.8, 0.6 Hz, H_{14}), 7.05 (1H, dd, *J*=7.8, 1.2 Hz, H_2), 7.03 – 6.98 (4H, m, H_{13}, H_{17} and H_{20}), 6.86 (1H, d, *J*=10.1 Hz, H_9), 6.75 (2H, d, *J*=8.7 Hz, H_{21}), 6.41 (1H, d, *J*=10.1 Hz, H_8), 4.89 (1H, app ddt, *J*=6.4, 4.4, 2.2 Hz, H_{23}), 4.01 – 3.93 (5H, m, 2×H_{18}, 2×H_{24}, and 1×H_{25}), 3.90 (1H, app td, *J*=8.2, 4.4 Hz, $H_{25'}$), 2.24 – 2.09 (2H, m, 2×H_{26}), 1.81 (3H, s, H_{11}).

^{13}C NMR (126 MHz, $CDCl_3$) δ 185.0, 156.0, 155.4, 149.4, 142.2, 139.4, 133.2, 133.0, 131.6, 130.7, 129.9, 129.9, 129.8, 128.6, 127.1, 126.8, 126.6, 126.0, 115.5, 77.4, 73.2, 67.3, 45.2, 38.6, 33.1, 27.3.

HRMS m/z: $[M+H]^+$ calculated for $[C_{28}H_{26}ClO_3]^+$ 445.1565, found 445.1568. Δ = +0.7 ppm.

Chiral SFC Analysis: CHIRALPAK IK (CO_2:MeOH, 70:30, 2.5 mL min^{-1}, 40 °C, 270 nm) indicated 25:1 dr, t_R = 8.15 (major), 8.99 (minor) minutes.

$[\alpha]_D^{25}$ = +81.9° (c 1.33, $CHCl_3$).

Characterization of Products in Scheme 2

(4*R*,4'*R*)-4,4'-(1,4-phenylene)Bis(4-methylnaphthalen-1(4*H*)-one) (3a)

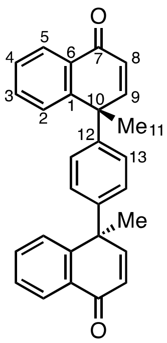

Prepared according to General Procedure B with 4-methylnaphthalen-1-ol (15.8 mg, 0.100 mmol) and 1,4-dibromobenzene (17.7 mg, 0.0750 mmol, 0.750 equiv.) as the starting materials, double the usual loadings of (*R*)-sSPhos (7.7 mg, 0.015 mmol, 15 mol%) and Pd_2dba_3 (2.3 mg, 0.0025 mmol, 2.5 mol%), and a longer reaction time of 48 h. Purification by flash column chromatography (0-20% EtOAc in hexane) afforded the title compound as a yellow solid (7.8 mg, 0.020 mmol [N.B. theoretical maximum: 0.050 mmol], 40% yield, > 99% *ee*, 17:1 dr).

[N.B. Product was isolated as an inseparable mixture of diastereomers. Diastereomers were indistinguishable by ^{1}H and ^{13}C NMR. dr was therefore determined by chiral SFC analysis of the isolated sample.]

^{1}H NMR (500 MHz, $CDCl_3$) δ 8.20 (2H, dd, *J*=7.7, 1.6 Hz, H_5), 7.45 (2H, app td, *J*=7.7, 1.6 Hz, H_3), 7.36 (2H, app td, *J*=7.7, 1.2 Hz, H_4), 7.15 (4H, s, H_{13}), 7.09 (2H, dd, *J*=7.7, 1.2 Hz, H_2), 6.87 (2H, d, *J*=10.1 Hz, H_9), 6.40 (2H, d, *J*=10.1 Hz, H_8), 1.85 (6H, s, H_{11}).

^{13}C NMR (126 MHz, $CDCl_3$) δ 185.2, 155.7, 149.6, 142.2, 132.9, 130.8, 128.8, 127.6, 127.1, 126.8, 125.9, 45.3, 27.2.

HRMS m/z: $[M+H]^+$ calculated for $[C_{28}H_{23}O_2]^+$ 391.1693, found 391.1677. Δ = −4.1 ppm.

Chiral SFC Analysis: CHIRALPAK IJ (CO_2:MeOH, 85:15, 2.5 mL min^{-1}, 40 °C, 250 nm) indicated > 99% *ee* and 17:1 dr, t_R = 6.66 (major enantiomer), 7.46 (minor diastereomer [meso compound]), 7.77 (minor enantiomer) minutes.

$[\alpha]_D^{25}$ = +195° (c 0.52, $CHCl_3$).

(4*R*,4'*R*)-4,4'-([1,1':4',1''-terphenyl]-4,4''-diyl)Bis(4-methylnaphthalen-1(4*H*)-one) (3b)

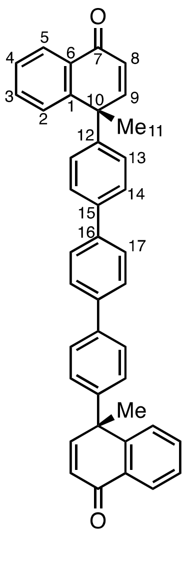

[N.B. Reaction performed with 0.50 equiv. aryl bromide to discourage formation of mono-coupled product. A significant amount of mono-coupled product was not observed.]

[N.B. The product was isolated as an inseparable mixture of diastereomers. Diastereomers displayed identical signals by ^{1}H and ^{13}C NMR. dr was determined by chiral SFC from the isolated sample.]

Prepared according to General Procedure B with 4-methylnaphthalen-1-ol (15.8 mg, 0.100 mmol) and 4,4''-dibromo-1,1':4',1''-terphenyl (19.4 mg, 0.050 mmol, 0.50 equiv.) as the starting materials, double the usual loadings of (*R*)-sSPhos (7.7 mg, 0.015 mmol, 15 mol%) and Pd_2dba_3 (2.3 mg, 0.0025 mmol, 2.5 mol%), and a longer reaction time of 48 h. Purification by flash column chromatography (0-40% EtOAc in heptane) afforded the title compound as a brown solid (19.4 mg, 0.036 mmol [N.B. theoretical maximum: 0.050 mmol], 71% yield, 99% *ee*, 13:1 dr).

^{1}H NMR (500 MHz, $CDCl_3$) δ 8.24 (2H, dd, *J*=8.0, 1.5 Hz, H_5), 7.62 (4H, s, H_{17}), 7.56 (4H, d, *J*=8.4 Hz, H_{14}), 7.47 (2H, app td, *J*=7.6, 1.5 Hz, H_3), 7.38 (2H, ddd, *J*=8.0, 7.6, 1.2 Hz, H_4), 7.32 (4H, d, *J*=8.4 Hz, H_{13}), 7.17 (2H, dd, *J*=7.6, 1.2 Hz, H_2), 6.96 (2H, d, *J*=10.0 Hz, H_9), 6.47 (2H, d, *J*=10.0 Hz, H_8), 1.93 (6H, s, H_{11}).

^{13}C NMR (126 MHz, $CDCl_3$) δ 185.3, 155.8, 149.8, 142.4, 139.5, 139.5, 133.0, 130.9, 128.8, 127.7, 127.5, 127.4, 127.0, 126.8, 125.9, 45.5, 27.3.

HRMS m/z: $[M+H]^+$ calculated for $[C_{40}H_{31}O_2]^+$ 543.2319, found 543.2315. $\Delta = -0.7$ ppm.

Chiral SFC Analysis: CHIRALPAK AD (CO_2 (A):0.1% NH_3 in MeOH (B), isocratic 50% B, 2.5 mL min^{-1}, 40 °C, 290 nm) indicated 99% *ee* and 13:1 dr, t_R = 2.76 (minor enantiomer), 3.31 (minor diastereomer [meso compound]), 3.70 (major enantiomer) minutes.

$[\alpha]_D^{25}$ = +162° (c 0.97, $CHCl_3$).

(4*R*,4'*R*)-4,4'-([1,1':3',1''-terphenyl]-4,4''-diyl)Bis(4-methylnaphthalen-1(4*H*)-one) (3c)

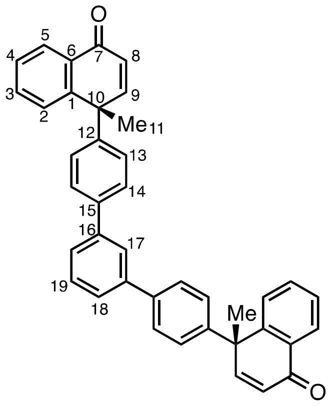

[N.B. Reaction performed with 0.50 equiv. aryl bromide to discourage formation of mono-coupled product. A significant amount of mono-coupled product was not observed]

[N.B. The product was isolated as an inseparable mixture of diastereomers. Diastereomers displayed identical signals by ^1H and ^{13}C NMR. dr was determined by chiral SFC from the isolated sample.]

Prepared according to General Procedure B with 4-methylnaphthalen-1-ol (15.8 mg, 0.100 mmol) and 4,4''-dibromo-1,1':3',1''-terphenyl (19.4 mg, 0.050 mmol, 0.50 equiv.) as the starting materials, double the usual loadings of (*R*)-sSPhos (7.7 mg, 0.015 mmol, 15 mol%) and Pd_2dba_3 (2.3 mg, 0.0025 mmol, 2.5 mol%), and a reaction time of 48 h. Purification by flash column chromatography (0-40% EtOAc in heptane) afforded the title compound as a brown solid (23.0 mg, 0.042 mmol [N.B. theoretical maximum: 0.050 mmol], 85% yield, 99% *ee*, 11:1 dr).

^{1}H NMR (500 MHz, $CDCl_3$) δ 8.24 (2H, dd, *J*=7.7, 1.5 Hz, H_5), 7.72 (1H, t, *J*=1.8 Hz, H_{17}), 7.58 – 7.51 (6H, m, H_{14} and H_{18}), 7.50 – 7.44 (3H, m, H_3 and H_{19}), 7.38 (2H, app td, *J*=7.7, 1.2 Hz, H_4), 7.32 (4H, d, *J*=8.4 Hz, H_{13}), 7.17 (2H, dd, *J*=7.8, 1.2 Hz, H_2), 6.96 (2H, d, *J*=10.1 Hz, H_9), 6.47 (2H, d, *J*=10.1 Hz, H_8), 1.93 (6H, s, H_{11}).

^{13}C NMR (126 MHz, $CDCl_3$) δ 185.3, 155.8, 149.8, 142.5, 141.1, 140.0, 133.0, 130.9, 129.4, 128.8, 127.7, 127.6, 127.1, 126.8, 126.3, 126.0, 125.9, 45.4, 27.3.

HRMS m/z: $[M+H]^+$ calculated for $[C_{40}H_{31}O_2]^+$ 543.2319, found 543.2305. Δ = −2.6 ppm.

Chiral SFC Analysis: CHIRALPAK IF (CO_2 (A):0.1% DEA in IPA (B), isocratic 50% B, 2.5 mL min^{-1}, 40 °C, 254 nm) indicated 99% *ee* and 11:1 dr, t_R = 1.64 (minor enantiomer), 1.85 (minor diastereomer [meso compound]), 2.07 (major enantiomer) minutes.

$[\alpha]_D^{25}$ = +137° (c 0.87, $CHCl_3$).

Characterization of Unsuccessful 2-Naphthol Substrate

1-Methyl-1-phenylnaphthalen-2(1*H*)-one (S1)

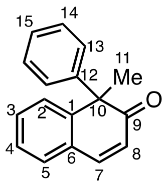

Prepared according to General Procedure B with 1-methylnaphthalen-2-ol (15.8 mg, 0.100 mmol) and bromobenzene (23.6 mg, 0.150 mmol) as the starting materials. ^{1}H NMR analysis of the crude reaction mixture with a dibromomethane internal standard revealed an NMR yield of 16%, with unreacted 1-methylnaphthalen-2-ol accounting for 62% of the mass balance. Due to the challenging separation of the title compound from 1-methylnaphthalen-2-ol, the impurity was removed according to the following procedure.

To the crude reaction product were added NaOH (aq.) (2 M, 0.5 mL) and $CDCl_3$ (0.8 mL), and the layers separated. The aqueous layer was further extracted with $CDCl_3$ (1×0.5 mL). The combined organic extracts were dried over Mg_2SO_4, filtered, and concentrated under reduced pressure. Purification by preparatory TLC (10% EtOAc in 40-60 °C petroleum ether) afforded the title compound as a colorless solid (2.4 mg, 0.010 mmol, 10%, 15% *ee*).

^{1}H NMR (700 MHz, $CDCl_3$) δ 7.51 (d, *J* = 9.8 Hz, 1H, H_7), 7.39 (dd, *J* = 7.3, 1.7 Hz, 1H, H_5), 7.36 – 7.28 (m, 2H, H_3 and H_4), 7.27 – 7.23 (m, 2H, H_{13}), 7.23 – 7.18 (m, 1H, H_{15}), 7.12 – 7.07 (m, 3H, H_2 and H_{14}), 6.17 (d, *J* = 9.8 Hz, 1H, H_8), 1.87 (s, 3H, H_{11}).

^{13}C NMR (176 MHz, $CDCl_3$) δ 202.6, 147.6, 145.1, 143.6, 130.3, 129.4, 129.4, 129.1, 128.6, 127.6, 127.2, 127.1, 124.6, 56.1, 26.4.

Chiral SFC Analysis: CHIRALPAK IK (CO_2:MeOH, 95:05, 2.5 mL min^{-1}, 40 °C, 300 nm) indicated 15% *ee*, t_R = 9.19 (major), 13.80 (minor) minutes.

[α]$_D^{25}$ = −20.4° (c 0.24, $CHCl_3$).

^{1}H NMR and ^{13}C NMR data in agreement with the literature.[11]

Characterization of Post-functionalization Products

Post-functionalization of Enone 2a

(3*S*,4*R*)-4-Methyl-4-phenyl-3-vinyl-3,4-dihydronaphthalen-1(2*H*)-one (4a)

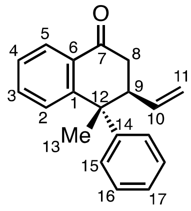

Based on the procedure reported by Breinbauer and co-workers.[12] An oven-dried vial was charged with CuI (22.9 mg, 0.120 mmol, 1.20 equiv.). The vial was sealed and evacuated and backfilled with N_2 (3×). THF (0.3 mL) was added under nitrogen, and the reaction mixture cooled to −78 °C. Vinylmagnesium bromide (1.0 M in THF, 0.24 mL, 0.24 mmol, 2.4 equiv.) was added under nitrogen, and the reaction was stirred at −78 °C for 15 minutes. In a separate vial, (*R*)-4-methyl-4-phenylnaphthalen-1(4*H*)-one (**2a**) (23.4 mg, 0.100 mmol, 1.00 equiv., 98% *ee*) was dissolved in THF (0.7 mL) and added dropwise to the reaction mixture under nitrogen. The reaction was stirred at −78 °C for 30 minutes, then stirred for 4 hours as the dry ice/acetone bath slowly warmed to room temperature. The reaction was quenched with sat. NH_4Cl (aq.) and stirred for a further 1 hour at room temperature. The quenched reaction was then diluted with Et_2O (2 mL), washed with a 9:1 mixture of sat. NH_4Cl (aq.): 25% NH_4OH (aq.) (3×1 mL) and brine (1×), dried over Mg_2SO_4, filtered, and concentrated under a stream of air. Analysis of the crude mixture by ^{1}H NMR analysis indicated a dr of 5:1. The crude product was purified by preparatory TLC (20% EtOAc in in 40-60 °C petroleum ether) to afford the title compound as a yellow oil (12.4 mg, 0.047 mmol, 47% yield, 98% *ee*).

^{1}H NMR (500 MHz, $CDCl_3$) δ 8.11 (ddd, *J* = 7.8, 1.5, 0.5 Hz, 1H, H_5), 7.45 (ddd, *J* = 7.9, 7.2, 1.5 Hz, 1H, H_3), 7.34 (ddd, *J* = 7.8, 7.2, 1.2 Hz, 1H, H_4), 7.32 – 7.27 (m, 2H, H_{16}), 7.25 – 7.21 (m, 1H, H_{17}), 7.14 (dd, *J* = 8.4, 1.3 Hz, 2H, H_{15}), 6.98 (d, *J* = 7.9 Hz, 1H, H_2), 5.60 (ddd, *J* = 17.2, 10.5, 6.9 Hz, 1H, H_{10}), 5.03 (app dt, *J* = 10.5, 1.3 Hz, 1H, $H_{11,trans}$), 4.97 (app dt, *J* = 17.2, 1.3 Hz, 1H, $H_{11,cis}$), 3.30 – 3.21 (m, 1H, H_9), 2.75 – 2.72 (m, 2H, H_8), 1.66 (s, 3H, H_{13}).

^{13}C NMR (126 MHz, $CDCl_3$) δ 197.7, 147.8, 140.5, 137.1, 134.0, 132.1, 129.4, 128.3, 127.8, 127.0, 126.9, 126.6, 117.0, 49.9, 46.5, 40.2, 23.8.

[N.B. The structure of this diastereomer was assigned based on a 2D NOESY correlation between H_{10} and H_{13}]

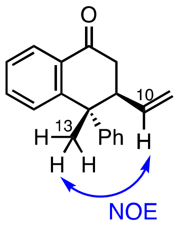

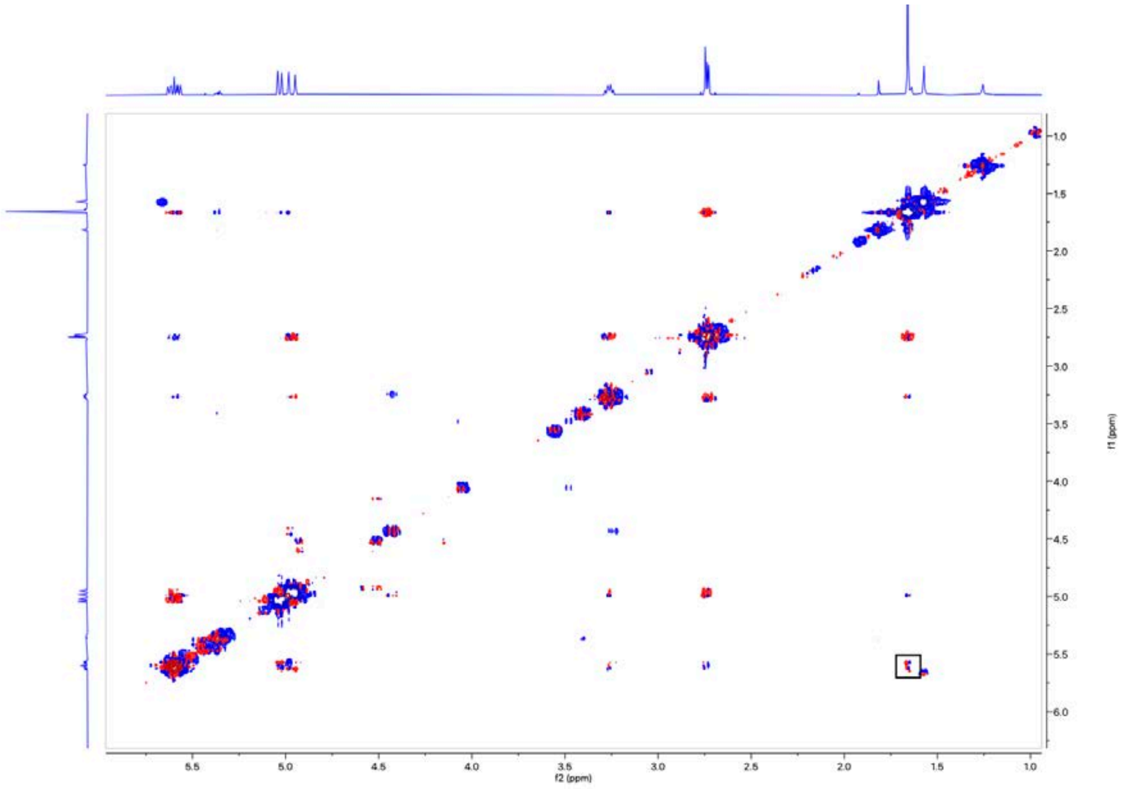

HRMS m/z: $[M+H]^+$ calculated for $[C_{19}H_{19}O]^+$ 263.1430, found 263.1434. Δ = +1.5 ppm.

Chiral SFC Analysis: CHIRALPAK IE (CO_2:MeOH, 90:10, 2.5 mL min^{-1}, 40 °C, 250 nm) indicated 98% *ee*, t_R = 3.72 (minor), 4.00 (major) minutes.

$[\alpha]_D^{25}$ = −22.8° (c 0.33, $CHCl_3$).

(1a*S*,7*R*,7a*S*)-7-Methyl-7-phenyl-7,7a-dihydronaphtho[2,3-*b*]oxiren-2(1a*H*)-one (4b)

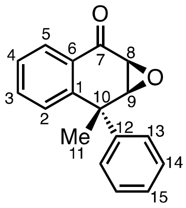

A vial containing a magnetic stirrer bar was charged with (*R*)-4-methyl-4-phenylnaphthalen-1(4*H*)-one (**2a**) (23.4 mg, 0.100 mmol, 1.00 equiv., 98% *ee*). A separate 10 mL stock solution of was made up from NaOH (8.0 mg), H_2O_2 (30% w/v in H_2O, 0.23 mL) and methanol, of which 1.0 mL was added to the reaction vial. [N.B. This should contain NaOH (0.80 mg, 0.020 mmol, 20 mol%) and H_2O_2 (6.8 mg, 0.20 mmol, 2.0 equiv.).] The reaction was heated to 50 °C and stirred for 48 h. H_2O (1 mL) and CH_2Cl_2 (1 mL) were added, and the layers were separated. The aqueous layer was further extracted with CH_2Cl_2 (2×). The combined organic layers were dried over $MgSO_4$, filtered, and the solvent removed under a stream of nitrogen. The crude product was purified by flash column chromatography (0-50% PhMe in 40-60 °C petroleum ether) to afford the title compound as a colorless solid (11.3 mg, 0.045 mmol, 45%, 97% *ee*).

[N.B. Only one diastereomer of epoxide **4b** was observed.]

^{1}H NMR (500 MHz, $CDCl_3$) δ 8.00 (1H, dd, *J*=7.8, 1.5 Hz, H_5), 7.55 (1H, ddd, *J*=8.0, 7.2, 1.5 Hz, H_3), 7.38 (1H, app td, *J*=7.5, 1.1 Hz, H_4), 7.31 – 7.26 (3H, m, H_2 and H_{14}), 7.24 – 7.19 (1H, m, H_{15}), 7.12 – 7.06 (2H, m, H_{13}), 3.77 (1H, d, *J*=4.0 Hz, H_8), 3.73 (1H, d, *J*=4.0 Hz, H_9), 2.11 (3H, s, H_{11}).

^{13}C NMR (126 MHz, $CDCl_3$) δ 194.7, 145.5, 143.9, 134.5, 129.7, 128.9, 128.0, 127.5, 127.4, 127.3, 126.6, 63.2, 55.9, 43.4, 24.1.

HRMS m/z: $[M+H]^+$ calculated for $[C_{17}H_{15}O_2]^+$ 251.1067, found 251.1057. Δ = −4.0 ppm.

Chiral SFC Analysis: CHIRALPAK IE (CO_2:MeOH, 94:06, 2.5 mL min^{-1}, 40 °C, 220 nm) indicated 97% *ee*, t_R = 6.52 (major), 7.06 (minor) minutes.

$[\alpha]_D^{25}$ = −310° (c 0.75, $CHCl_3$).

It was expected that the depicted diastereomer would form preferentially, resulting from attack of the nucleophilic hydroperoxide anion from the less hindered methyl-substituted face of enone **2a**. The 2D NOESY spectrum was inconclusive: since protons H_4 and H_5 lie not far off the plane of the ketone ring, correlations could be seen with both H_{11} and H_{13}.

Therefore, to further verify our assignment, the two possible diastereomers of epoxide **4b** were predicted and compared with the experimental spectra using the DP4+ probabilistic tool.[13] The predicted NMR data for epoxide **4b** showed much closer agreement with the experimental data. We thank Dr. Peter Gierth for his kind assistance with this analysis.

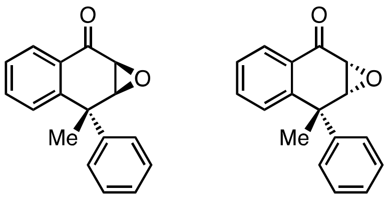

4b (good match) **(poor match)**

Enantioselective Synthesis of Quaternized Sertraline Variants

(*S*)-4-(3,4-dichlorophenyl)-4-Methylnaphthalen-1(4*H*)-one (5a)

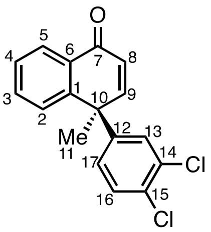

[N.B. Reaction performed with (*S*)-sSPhos.]

<u>0.1 mmol scale:</u>

Prepared according to General Procedure B with 4-methylnaphthalen-1-ol (15.8 mg, 0.100 mmol) and 4-bromo-1,2-dichlorobenzene (33.9 mg, 0.150 mmol) as the starting materials, double the usual loadings of (*S*)-sSPhos (7.7 mg, 0.015 mmol, 15 mol%) and Pd_2dba_3 (2.3 mg, 0.0025 mmol, 2.5 mol%), and a longer reaction time of 48 h. Purification by flash column chromatography (0-5% EtOAc in hexane) afforded the title compound as a yellow solid (19.1 mg, 0.063 mmol, 63% yield, 94% *ee*).

^{1}H NMR (700 MHz, $CDCl_3$) δ 8.21 (1H, dd, *J*=7.9, 1.5 Hz, H_5), 7.50 – 7.45 (1H, m, H_3), 7.41 – 7.37 (1H, m, H_4), 7.36 – 7.34 (2H, m, H_{13} and H_{16}), 7.07 (1H, dd, *J*=8.3, 1.1 Hz, H_2), 7.02 (1H, dd, *J*=8.5, 2.3 Hz, H_{17}), 6.85 (1H, d, *J*=10.0 Hz, H_9), 6.46 (1H, d, *J*=10.0 Hz, H_8), 1.86 (3H, s, H_{11}).

^{13}**C NMR** (176 MHz, $CDCl_3$) δ 184.8, 154.4, 148.7, 143.8, 133.2, 133.0, 131.6, 130.8, 130.7, 129.2, 128.6, 127.4, 127.0, 126.9, 126.5, 45.1, 27.3.

HRMS m/z: $[M+H]^+$ calculated for $[C_{17}H_{13}Cl_2O]^+$ 303.0338, found 303.0328. Δ = −3.3 ppm.

Chiral SFC Analysis: CHIRALPAK IE (CO_2:MeOH, 90:10, 2.5 mL min^{-1}, 40 °C, 250 nm) indicated 94% *ee*, t_R = 7.17 (major), 7.68 (minor) minutes.

$[\alpha]_D^{25}$ = −132° (c 0.42, $CHCl_3$).

2 mmol scale reaction:

[N.B. Reaction performed with (*S*)-sSPhos.]

A vial containing a magnetic stirrer bar was sequentially charged with 4-methylnaphthalen-1-ol (316 mg, 2.00 mmol, 1.00 equiv.), (*S*)-sSPhos (154 mg, 0.300 mmol, 15.0 mol%), K_3PO_4 (637 mg, 3.00 mmol, 1.50 equiv.), Pd_2dba_3 (45.8 mg, 0.0500 mmol, 2.50 mol%), and 4-bromo-1,2-dichlorobenzene (678 mg, 3.00 mmol, 1.50 equiv.). The vial was sealed, and evacuated and backfilled with N_2 (4×). PhMe (10 mL) was added under nitrogen. The reaction was stirred at 90 °C and 900 rpm for 48 h. Upon completion, the reaction mixture was filtered through celite, eluting with EtOAc. The filtrate was concentrated under a stream of air. Purification by flash column chromatography (0-5% EtOAc in hexane) afforded the title compound as a yellow solid (408 mg, 1.35 mmol, 67% yield, 92% *ee*).

Chiral SFC Analysis: CHIRALPAK IE (CO_2:MeOH, 90:10, 2.5 mL min^{-1}, 40 °C, 250 nm) indicated 92% *ee*, t_R = 7.29 (major), 7.80 (minor) minutes.

(*S*)-4-(3,4-dichlorophenyl)-4-Methyl-3,4-dihydronaphthalen-1(2*H*)-one (5b)

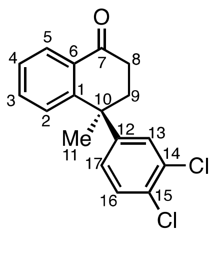

To a solution of (*S*)-4-(3,4-dichlorophenyl)-4-methylnaphthalen-1(4*H*)-one (**5a**) (121 mg, 0.400 mmol, 1.00 equiv., 92% *ee*) in EtOAc (4 mL) was added Pd/C (10 wt % loading Pd, 21.3 mg, 0.0200 mmol, 5.00 mol%). The vial was capped with a septum and evacuated and backfilled with hydrogen (3×). The reaction was stirred at room temperature under a hydrogen atmosphere for

45 minutes, at which point it was judged complete by TLC. The reaction was filtered through celite, eluting with EtOAc and MeOH, and concentrated under reduced pressure. The crude product was purified by flash column chromatography (0-5% EtOAc in 40-60 °C petroleum ether) to afford the title compound as a yellow solid (88.9 mg, 0.291 mmol, 73% yield, 92% *ee*).

^{1}H NMR (700 MHz, $CDCl_3$) δ 8.12 (1H, ddd, *J*=7.8, 1.6, 0.5 Hz, H_5), 7.56 (1H, ddd, *J*=7.9, 7.3, 1.6 Hz, H_3), 7.41 (1H, ddd, *J*=7.8, 7.3, 1.2 Hz, H_4), 7.32 (1H, d, *J*=8.5 Hz, H_{16}), 7.21 – 7.17 (2H, m, H_2 and H_{13}), 6.87 (1H, dd, *J*=8.5, 2.3 Hz, H_{17}), 2.66 (1H, ddd, *J*=17.4, 6.8, 4.4 Hz, H_8), 2.46 (1H, ddd, *J*=17.4, 10.5, 4.6 Hz, $H_{8'}$), 2.40 (1H, ddd, *J*=13.7, 6.8, 4.6 Hz, H_9), 2.29 (1H, ddd, *J*=13.7, 10.5, 4.4 Hz, $H_{9'}$), 1.80 (3H, s, H_{11}).

^{13}C NMR (176 MHz, $CDCl_3$) δ 197.6, 149.2, 148.4, 134.2, 132.7, 132.4, 130.7, 130.3, 129.3, 128.2, 127.7, 127.5, 126.9, 42.8, 38.8, 35.4, 29.1.

HRMS m/z: $[M+H]^+$ calculated for $[C_{17}H_{15}Cl_2O]^+$ 305.0494, found 305.0488. Δ = −2.0 ppm.

Chiral SFC Analysis: CHIRALPAK IE (CO_2:MeOH, 90:10, 2.5 mL min^{-1}, 40 °C, 220 nm) indicated 92% ee, t_R = 5.94 (major), 6.41 (minor) minutes.

$[\alpha]_D^{25}$ = + 9.9° (c 0.61, $CHCl_3$).

Synthesis of (1*S*,4*S*)-4-(3,4-dichlorophenyl)-4-methyl-1,2,3,4-tetrahydronaphthalen-1-ol (5cb) and (1*R*,4*S*)-4-(3,4-dichlorophenyl)-4-methyl-1,2,3,4-tetrahydronaphthalen-1-ol (5ca)

A vial containing a magnetic stirrer bar was charged with (*S*)-4-(3,4-dichlorophenyl)-4-methyl-3,4-dihydronaphthalen-1(2*H*)-one (**5b**) (46.2 mg, 0.151 mmol, 1.00 equiv., 92% *ee*), and methanol (1 mL) was added. The solution was cooled to 0 °C, and $NaBH_4$ (11.4 mg, 0.302 mmol, 2.00 equiv.) was added. The reaction was stirred at 0 °C for 5 minutes, and room temperature for a further 5 minutes, at which point it was judged complete by TLC. The reaction was quenched with sat. NH_4Cl (aq.) (0.5 mL), and H_2O (1 mL) was added. The aqueous phase was extracted with $CDCl_3$ (3×1 mL). The combined organic extracts were dried over $MgSO_4$, filtered, concentrated under a stream of air, and purified by flash column chromatography (0-15% EtOAc in 40-60 °C petroleum ether) to sequentially afford (1*S*,4*S*)-4-(3,4-dichlorophenyl)-4-methyl-1,2,3,4-tetrahydronaphthalen-1-ol (**5cb**) as a yellow solid (24.2 mg, 0.0788 mmol, 52% yield, 92% *ee*) and (1*R*,4*S*)-4-(3,4-dichlorophenyl)-4-methyl-1,2,3,4-tetrahydronaphthalen-1-ol (**5ca**) (19.0 mg, 0.0618 mmol, 41% yield, 93% *ee*).

(1*S*,4*S*)-4-(3,4-dichlorophenyl)-4-Methyl-1,2,3,4-tetrahydronaphthalen-1-ol (5cb)

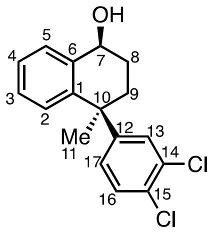

^{1}H NMR (500 MHz, $CDCl_3$) δ 7.48 (d, J = 7.6, 1.6 Hz, 1H, H_5), 7.31 – 7.28 (m, 2H, H_{13} and H_{16}), 7.26 (app td, J = 7.6, 1.4 Hz, 1H, H_4), 7.21 (app td, J = 7.6, 1.6 Hz, 1H, H_3), 6.97 (dd, J = 8.5, 2.3 Hz, 1H, H_{17}), 6.89 (dd, J = 7.6, 1.4 Hz, 1H, H_2), 4.84 (app t, J = 5.1 Hz, 1H, H_7), 2.33 – 2.21 (m, 1H, H_9), 2.13 – 2.02 (m, 1H, H_8), 1.84 – 1.76 (m, 2H, $H_{8'}$ and $H_{9'}$), 1.65 (s, 3H, H_{11}). [N.B. The O-H peak was not observed].

^{13}C NMR (126 MHz, $CDCl_3$) δ 151.7, 143.5, 138.6, 132.1, 129.9, 129.9, 129.5, 129.3, 128.7, 128.5, 127.5, 127.0, 68.5, 43.0, 36.7, 29.3, 28.7.

[N.B. The structure of this diastereomer was assigned based on a 2D NOESY correlation between H_7 and H_{11}.]

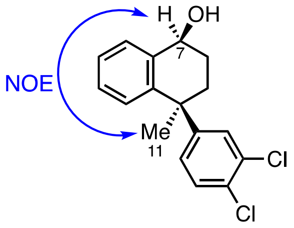

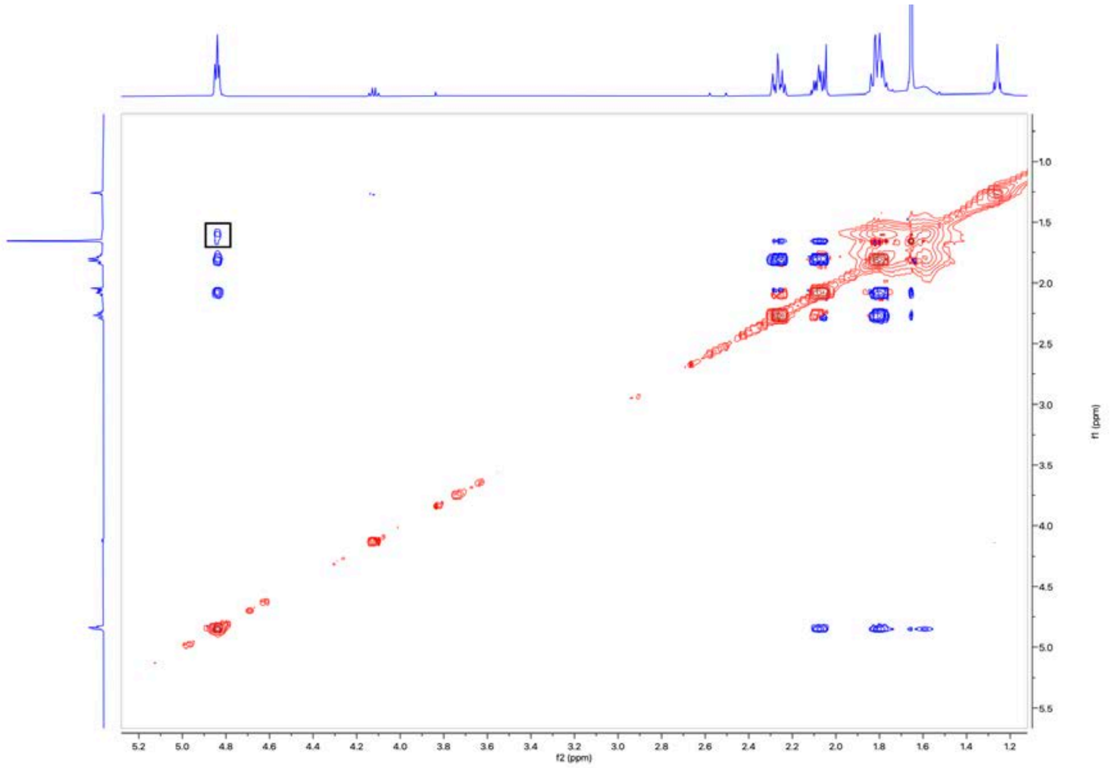

HRMS m/z: $[M+Na]^+$ calculated for $[C_{17}H_{16}Cl_2NaO]^+$ 329.0470, found 329.0462. Δ = −2.4 ppm.

Chiral SFC Analysis: CHIRALPAK IE (CO_2:MeOH, 90:10, 2.5 mL min^{-1}, 40 °C, 220 nm) indicated 92% *ee*, t_R = 5.46 (major), 6.93 (minor) minutes.

$[\alpha]_D^{21}$ = +31.9° (c 1.21, $CHCl_3$).

(1*R*,4*S*)-4-(3,4-dichlorophenyl)-4-Methyl-1,2,3,4-tetrahydronaphthalen-1-ol (5ca)

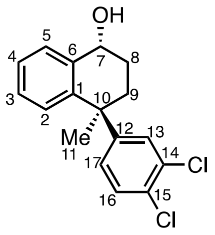

^{1}H NMR (700 MHz, $CDCl_3$) δ 7.51 (dd, *J* = 7.6, 1.7 Hz, 1H, H_5), 7.29 (app td, *J* = 7.6, 1.4 Hz, 1H, H_4), 7.27 (d, *J* = 8.5 Hz, 1H, H_{16}), 7.27 – 7.22 (m, 1H, H_3), 7.13 (d, *J* = 2.3 Hz, 1H, H_{13}), 7.02 (dd, *J* = 7.9, 1.4 Hz, 1H, H_2), 6.82 (dd, *J* = 8.5, 2.3 Hz, 1H, H_{17}), 4.81 (app t, *J* = 5.1 Hz, 1H, H_7), 2.16 (ddd, *J* = 13.6, 9.1, 4.3 Hz, 1H, H_9), 1.93 (ddd, *J* = 13.6, 7.0, 3.6 Hz, 1H, $H_{9'}$), 1.88 – 1.84 (m, 2H, H_8), 1.74 (s, 3H, H_{11}). [N.B. The O-H peak was not observed].

^{13}C NMR (176 MHz, $CDCl_3$) δ 151.1, 142.9, 138.8, 132.1, 129.9, 129.9, 129.4, 128.9, 128.7, 128.5, 127.2, 127.1, 68.3, 42.8, 36.4, 29.9, 28.8.

HRMS m/z: $[M+HCO_2]^-$ calculated for $[C_{18}H_{17}{}^{35}Cl^{37}ClO_3]^-$ 353.0531, found 353.0541. Δ = +2.8 ppm.

Chiral SFC Analysis: CHIRALPAK IE (CO_2:MeOH, 90:10, 2.5 mL min^{-1}, 40 °C, 220 nm) indicated 93% *ee*, t_R = 5.62 (major), 6.07 (minor) minutes.

$[\alpha]_D^{21}$ = −8.0° (c 0.95, $CHCl_3$).

(1*S*,4*R*)-4-Azido-1-(3,4-dichlorophenyl)-1-methyl-1,2,3,4-tetrahydronaphthalene (S19b)

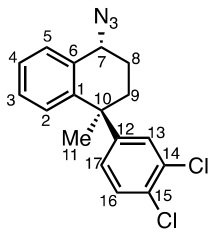

Based on the procedure reported by Lautens and Rovis.[14] A vial containing a magnetic stirrer bar was charged with (1*S*,4*S*)-4-(3,4-dichlorophenyl)-4-methyl-1,2,3,4-tetrahydronaphthalen-1-ol (**5cb**) (12.9 mg, 0.0420 mmol, 1.00 equiv., 92% *ee*). The vial was sealed, and evacuated and backfilled with N_2 (3×). THF (0.5 mL) was added under nitrogen. The reaction was set stirring, and diphenylphosphoryl azide (34.7 mg, 27.1 µL, 0.126 mmol, 3.00 equiv.) was added under nitrogen. The reaction was stirred for 5 minutes, before cooling to 0 °C in an ice bath. DBU (19.2 mg, 18.8 µL, 0.126 mmol, 3.00 equiv.) was added under nitrogen, and the reaction was slowly warmed to room temperature as the ice bath melted. The reaction was stirred at room temperature for 20 h, at which point it was judged complete by TLC. Et_2O and H_2O were added, the layers were separated, and the aqueous layer was further extracted with Et_2O (3×). The combined organic extracts were dried over $MgSO_4$, filtered, concentrated under a stream of air, and purified by flash column chromatography (0-5% Et_2O in 40-60 °C petroleum ether) to afford the title compound as a colorless solid (10.7 mg, 0.0322 mmol, 77% yield, 91% *ee*).

^{1}H NMR (500 MHz, $CDCl_3$) δ 7.40 – 7.35 (m, 1H, H_5), 7.35 – 7.30 (m, 2H, H_4 and H_3), 7.27 (d, *J* = 8.5 Hz, 1H, H_{16}), 7.11 – 7.07 (m, 2H, H_2 and H_{13}), 6.79 (dd, *J* = 8.5, 2.4 Hz, 1H, H_{17}), 4.62 (app t, *J* = 4.4 Hz, 1H, H_7), 2.21 – 2.12 (m, 1H, H_9), 1.96 – 1.81 (m, 3H, 2×H_8 and $H_{9'}$), 1.75 (s, 3H, H_{11}).

^{13}C NMR (126 MHz, $CDCl_3$) δ 150.8, 143.0, 133.9, 132.2, 130.1, 129.9, 129.5, 129.4, 129.3, 129.1, 127.2, 127.0, 59.8, 42.5, 36.5, 30.2, 25.5.

HRMS m/z: $[M{-}N_2{+}H]^+$ calculated for $[C_{17}H_{16}Cl^{37}ClN]^+$ 306.0625, found 306.0627. Δ = +0.7 ppm.

Chiral SFC Analysis: CHIRALPAK IJ (CO_2:MeOH, 95:05, 2.5 mL min^{-1}, 40 °C, 220 nm) indicated 91% *ee*, t_R = 5.09 (major), 6.25 (minor) minutes.

$[\alpha]_D^{20}$ = +44.5° (c 0.54, $CHCl_3$).

(1*S*,4*S*)-4-Azido-1-(3,4-dichlorophenyl)-1-methyl-1,2,3,4-tetrahydronaphthalene (S19a)

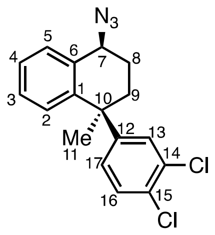

Based on the procedure reported by Lautens and Rovis.[14] A vial containing a magnetic stirrer bar was charged with (1*R*,4*S*)-4-(3,4-dichlorophenyl)-4-methyl-1,2,3,4-tetrahydronaphthalen-1-ol (**5ca**) (17.0 mg, 0.0554 mmol, 1.00 equiv., 93% *ee*). The vial was sealed, and evacuated and backfilled with N_2 (3×). THF (0.5 mL) was added under nitrogen. The reaction was set stirring, and diphenylphosphoryl azide (45.6 mg, 35.6 µL, 0.166 mmol, 3.00 equiv.) was added under nitrogen. The reaction was stirred for 5 minutes, before cooling to 0 °C in an ice bath. DBU (25.3 mg, 24.8 µL, 0.166 mmol, 3.00 equiv.) was added under nitrogen, and the reaction was slowly warmed to room temperature as the ice bath melted. The reaction was stirred at room temperature for 20 h, at which point it was judged complete by TLC. Et_2O and H_2O were added, the layers were separated, and the aqueous layer was further extracted with Et_2O (3×). The combined organic extracts were dried over $MgSO_4$, filtered, concentrated under a stream of air, and purified by flash column chromatography (0-5% Et_2O in 40-60 °C petroleum ether) to afford the title compound as a colorless solid (14.9 mg, 0.0448 mmol, 81% yield, 94% *ee*).

^{1}H NMR (500 MHz, $CDCl_3$) δ 7.36 – 7.33 (m, 2H, H_5 and H_{13}), 7.32 (d, *J* = 8.5 Hz, 1H, H_{16}), 7.29 – 7.21 (m, 2H, H_3 and H_4), 6.95 (dd, *J* = 8.5, 2.3 Hz, 1H, H_{17}), 6.89 (dd, *J* = 7.6, 1.6 Hz, 1H, H_2), 4.64 (app t, *J* = 4.8 Hz, 1H, H_7), 2.25 (ddd, *J* = 13.7, 11.4, 2.8 Hz, 1H, H_9), 2.18 – 2.09 (m, 1H, H_8), 1.92 (dddd, *J* = 13.9, 6.5, 4.8, 2.8 Hz, 1H, $H_{8'}$), 1.81 (dddd, *J* = 13.7, 6.5, 2.8, 0.8 Hz, 1H, $H_{9'}$), 1.66 (s, 3H, H_{11}).

^{13}C NMR (126 MHz, $CDCl_3$) δ 151.3, 144.2, 133.5, 132.2, 130.2, 130.1, 129.8, 129.4, 129.3, 129.1, 127.5, 126.9, 59.9, 42.9, 36.8, 29.1, 25.9.

HRMS m/z: $[M{-}N_2{+}H]^+$ calculated for $[C_{17}H_{16}Cl^{37}ClN]^+$ 306.0625, found 306.0633. Δ = +2.6 ppm.

Chiral SFC Analysis: CHIRALPAK IK (CO_2:MeOH, 90:10, 1.25 mL min^{-1}, 40 °C, 230 nm) indicated 94% *ee*, t_R = 8.90 (major), 9.21 (minor) minutes.

$[\alpha]_D^{20}$ = −8.1° (c 0.75, $CHCl_3$).

(1*R*,4*S*)-4-(3,4-dichlorophenyl)-4-Methyl-1,2,3,4-tetrahydronaphthalen-1-amine [“Dasotraline variant] (5db)

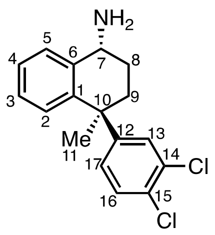

To a solution of (1*S*,4*R*)-4-azido-1-(3,4-dichlorophenyl)-1-methyl-1,2,3,4-tetrahydronaphthalene (**S19b**) (6.0 mg, 0.018 mmol, 1.00 equiv., 91% *ee*) in EtOH (1 mL) was added Pd/C (10 wt % loading Pd, 1.9 mg, 0.0018 mmol, 10 mol%). The vial was capped with a septum and evacuated and backfilled with hydrogen (3×). The reaction was stirred at room temperature under a hydrogen atmosphere for 2 hours, at which point it was judged complete by TLC. The reaction was filtered through celite, eluting with MeOH, and concentrated under a stream of air. A small unidentified impurity in the crude sample was removed according to the following procedure.

To the crude sample were added 10% NaOH (aq.) (0.5 mL) and CH_2Cl_2 (0.5 mL) and the layers separated. The aqueous layer was further extracted with CH_2Cl_2 (2×0.5 mL). The combined organic extracts were dried over Mg_2SO_4, filtered, and concentrated under a stream of air. This process was then repeated three further times to afford the title compound as a colorless solid (4.9 mg, 0.016 mmol, 89% yield).

[N.B. To avoid protonation of the amine caused by small amounts of acid present in $CDCl_3$, it was filtered through a plug of oven-dried K_2CO_3 and $MgSO_4$ before use.]

^{1}H NMR (700 MHz, $CDCl_3$) δ 7.47 (d, *J* = 7.7 Hz, 1H, H_5), 7.28 – 7.24 (m, 2H, H_4 and H_{16}), 7.20 – 7.16 (m, 2H, H_3 and H_{13}), 6.97 (dd, *J* = 7.9, 1.4 Hz, 1H, H_2), 6.84 (dd, *J* = 8.5, 2.2 Hz, 1H, H_{17}), 4.03 (app t, *J* = 5.4 Hz, 1H, H_7), 2.09 (ddd, *J* = 13.7, 9.6, 3.0 Hz, 1H, H_9), 1.95 (ddd, *J* = 13.7, 8.3, 3.0 Hz, 1H, $H_{9'}$), 1.89 (dddd, *J* = 13.0, 9.6, 5.4, 3.0 Hz, 1H, H_8), 1.73 (s, 3H, H_{11}), 1.70 – 1.66 (m, 1H, $H_{8'}$). [N.B. The NH_2 peak was not observed].

^{13}C NMR (176 MHz, $CDCl_3$) δ 151.6, 142.7, 140.9, 132.1, 129.9, 129.8, 129.4, 128.9, 128.2, 127.4, 127.2, 127.0, 49.6, 43.0, 37.2, 30.0, 29.8.

HRMS m/z: $[M+H]^+$ calculated for $[C_{17}H_{18}Cl^{37}ClN]^+$ 308.0781, found 308.0779. Δ = −0.6 ppm.

$[\alpha]_D^{25}$ = +4.9° (c 0.45, MeOH).

(1*S*,4*S*)-4-(3,4-dichlorophenyl)-4-Methyl-1,2,3,4-tetrahydronaphthalen-1-amine ["Desmethylsertraline variant"] (5da)

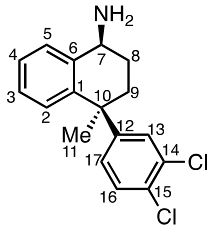

To a solution of (1*S*,4*S*)-4-azido-1-(3,4-dichlorophenyl)-1-methyl-1,2,3,4-tetrahydronaphthalene (**S19a**) (8.0 mg, 0.024 mmol, 1.00 equiv., 94% *ee*) in EtOH (1 mL) was added Pd/C (10 wt % loading Pd, 2.6 mg, 0.0024 mmol, 10 mol%). The vial was capped with a septum and evacuated and backfilled with hydrogen (3×). The reaction was stirred at room temperature under a hydrogen atmosphere for 1 hour, at which point it was judged complete by TLC. The reaction was filtered through celite, eluting with MeOH, and concentrated under a stream of air. A small unidentified impurity in the crude sample was removed according to the following procedure.

To the crude sample were added 10% NaOH (aq.) (0.5 mL) and CH_2Cl_2 (0.5 mL) and the layers separated. The aqueous layer was further extracted with CH_2Cl_2 (2×0.5 mL). The combined organic extracts were dried over Mg_2SO_4, filtered, and concentrated under a stream of air. This process was then repeated once more to afford the title compound as a colorless solid (7.0 mg, 0.023 mmol, 95% yield).

[N.B. To avoid protonation of the amine caused by small amounts of acid present in $CDCl_3$, it was filtered through a plug of oven-dried K_2CO_3 and $MgSO_4$ before use.]

^{1}H NMR (500 MHz, $CDCl_3$) δ 7.46 (ddd, *J* = 7.6, 1.5, 0.7 Hz, 1H, H_5), 7.30 – 7.27 (m, 2H, H_{13} and H_{16}), 7.24 (app td, *J* = 7.6, 1.4 Hz, 1H, H_4), 7.16 (app td, *J* = 7.6, 1.5 Hz, 1H, H_3), 6.94 (dd, *J* = 8.4, 2.3 Hz, 1H, H_{17}), 6.91 (dd, *J* = 7.6, 1.4 Hz, 1H, H_2), 4.06 (dd, J = 6.5, 5.2 Hz, 1H, H_7), 2.19 (ddd, *J* = 13.6, 9.5, 2.7 Hz, 1H, H_9), 2.04 (dddd, *J* = 13.5, 9.5, 5.2, 2.7 Hz, 1H, H_8), 1.83 (ddd, *J* = 13.6, 8.8, 2.7 Hz, 1H, $H_{9'}$), 1.66 (s, 3H, H_{11}), 1.60 – 1.51 (m, 1H, $H_{8'}$). [N.B. The NH_2 peak was not observed].

^{13}C NMR (126 MHz, $CDCl_3$) δ 151.9, 142.9, 140.8, 132.1, 129.9, 129.8, 129.4, 129.2, 128.4, 127.5, 127.4, 126.9, 49.9, 43.1, 37.5, 29.8, 29.7.

HRMS m/z: $[M+Na]^+$ calculated for $[C_{17}H_{17}Cl_2NNa]^+$ 328.0630, found 328.0644. Δ = +4.3 ppm.

$[\alpha]_D^{25}$ = +21.7° (c 0.64, MeOH).

Enantioselective Formal Synthesis of Alanense A

5-Bromo-4-iodo-2-methylphenol (8)

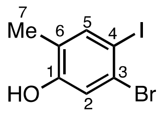

To a solution of ICl (7.39 g, 2.38 mL, 45.5 mmol, 1.30 equiv.) in glacial acetic acid (30 mL) was added 5-bromo-2-methylphenol (6.55 g, 35.0 mmol, 1.00 equiv.). The mixture was heated under reflux at 130 °C for 3 hours. The reaction was cooled to room temperature and 2 M NaOH (100 mL) was added. The mixture was extracted with CH_2Cl_2 (3×), and the combined organic extracts were washed with sat. $Na_2S_2O_3$ (aq.), dried over Na_2SO_4, and concentrated under reduced pressure. The crude product was recrystallized from hot hexane to afford the title compound as a brown solid (7.35 g, 23.5 mmol, 67% yield).

^{1}H NMR (400 MHz, $CDCl_3$) δ 7.57 (d, *J* = 0.9 Hz, 1H, H_5), 7.07 (s, 1H, H_2), 4.90 (br s, 1H, H_O), 2.16 (d, *J* = 0.9 Hz, 3H, H_7).

^{13}C NMR (126 MHz, $CDCl_3$) δ 154.8, 141.6, 126.7, 125.8, 119.3, 89.5, 15.1.

Data in agreement with the literature.[15]

1-Bromo-2-iodo-5-(methoxymethoxy)-4-methylbenzene (9)

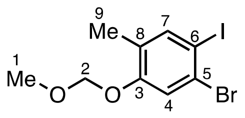

[N.B. MOM-Cl (2.1 M in PhMe) was prepared by Jiří Doležel according to the literature, for which we are very grateful.][16]

A solution of 5-bromo-4-iodo-2-methylphenol (**8**) (5.19 g, 16.6 mmol, 1.00 equiv.) in MeCN (160 mL) was cooled to 0 °C. K_2CO_3 (13.8 g, 99.6 mmol, 6.00 equiv.) was added and the reaction was stirred for 30 minutes at 0 °C. MOM-Cl (2.1 M in PhMe, 15.8 mL, 33.2 mmol, 2.00 equiv.) was added, and the reaction was warmed to room temperature and stirred for 1 h. MeOH (80 mL) was added, and the mixture was concentrated under reduced pressure. H_2O (400 mL) and EtOAc (400 mL) were added and the layers separated. The aqueous layer was further extracted with EtOAc (2×). The combined organic extracts were dried over Na_2SO_4 and concentrated under reduced pressure to afford the title compound as an orange oil (5.84 g, 16.4 mmol, 99% yield).

^{1}H NMR (400 MHz, $CDCl_3$) δ 7.59 (d, J = 0.8 Hz, 1H, H_7), 7.32 (s, 1H, H_4), 5.16 (s, 2H, H_2), 3.47 (s, 3H, H_1), 2.14 (d, J = 0.8 Hz, 3H, H_9).

^{13}C NMR (101 MHz, $CDCl_3$) δ 156.1, 141.2, 128.9, 126.7, 118.3, 94.6, 90.9, 56.3, 15.7.

Data in agreement with the literature.[15]

Synthesis of 7-(methoxymethoxy)-1,6-dimethyl-1,4-dihydro-1,4-epoxynaphthalene (S20) and 6-(methoxymethoxy)-1,7-dimethyl-1,4-dihydro-1,4-epoxynaphthalene (S21)

No product formation was detected when the benzyne cycloaddition was attempted with *n*-BuLi, which was used in the synthesis of related substrates, such as 6-methoxy-4-methylnaphthalen-1-ol (**1f**) and 7-methoxy-4-methylnaphthalen-1-ol (**1g**) (*vide supra*).

Better results were obtained upon switching to *t*-BuLi (Scheme). Due to the challenging separation of epoxide intermediates **S20** and **S21** on silica, no clean sample of either product was obtained. Eluting fractions were divided into three bands (**A**, **B**, and **C**) of varying regioisomeric ratios. This enabled the characterization of both epoxide intermediates **S20** and **S21** as the major components of bands **A** and **C** respectively, while also assisting with the less challenging but still difficult separation of regioisomeric naphthols **10** and **1h** following the subsequent isomerization.

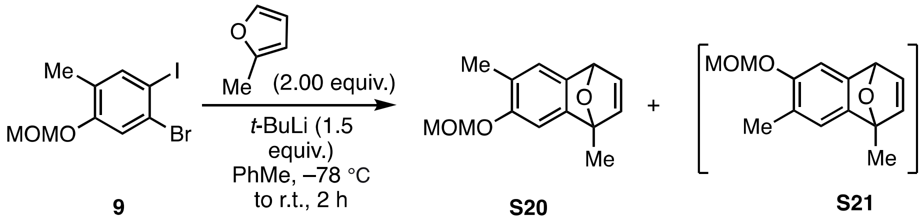

Overall: 1.1:1 *rr*, 88% yield
Band A: 33% yield (3.5:1)
Band B: 44% yield (1:1.2)
Band C: 11% yield (1:5.7)

A heat gun-dried round bottom flask containing a magnetic stirrer bar was charged with 1-bromo-2-iodo-5-(methoxymethoxy)-4-methylbenzene (**9**) (3.75 g, 10.5 mmol, 1.00 equiv.), 2-methylfuran (1.72 g, 1.89 ml, 21.0 mmol, 2.00 equiv.), and PhMe (45 mL) under nitrogen. The mixture was cooled to −78 °C, and *t*-BuLi (1.7 M in pentane, 6.18 ml, 10.5 mmol, 1.00 equiv.) was added under nitrogen. The reaction was stirred at −78 °C for 45 minutes and at room temperature for 1 h. The reaction was quenched with water (30 mL) and extracted with EtOAc (3×). The combined organic extracts were dried over Na_2SO_4, concentrated under reduced pressure, and purified by flash column chromatography (the silica was basified with several column volumes of 10% NEt_3 in EtOAc, then dried; eluent: 0-10% EtOAc in 40-60 °C petroleum ether). Product regioisomers were partially separable on silica, although no clean fractions of either regioisomer were obtained. Fractions containing the desired products were therefore divided into three bands of varying regioisomeric ratios. Band **A** eluted first, band **C** eluted last.

Band A: yellow oil (795 mg, 3.42 mmol, 33% yield, 3.5:1 **S20**:**S21**).

Band B: yellow oil (1.08 g, 4.65 mmol, 44% yield, 1:1.2 **S20**:**S21**).

Band C: yellow oil (273 mg, 1.18 mmol, 11% yield, 1:5.7 **S20**:**S21**).

Data for the major components of bands **A** and **C** respectively are presented below.

(*rac*)-7-(methoxymethoxy)-1,6-Dimethyl-1,4-dihydro-1,4-epoxynaphthalene (S20)

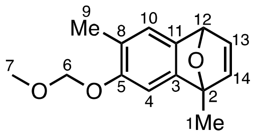

Major regioisomer of band **A**.

^{1}**H NMR** (400 MHz, $CDCl_3$) δ 7.03 – 6.99 (m, 2H, H_{10} and H_{13}), 6.98 (s, 1H, H_4), 6.76 (d, *J* = 5.5 Hz, 1H, H_{14}), 5.59 – 5.53 (m, 1H, H_{12}), 5.18 (d, *J* = 6.7 Hz, 1H, H_6), 5.14 (d, *J* = 6.7 Hz, 1H, $H_{6'}$), 3.49 (s, 3H, H_7), 2.18 (s, 3H, H_9), 1.90 (s, 3H, H_1).

^{13}**C NMR** (101 MHz, $CDCl_3$) δ 152.7, 150.8, 145.6, 144.7, 143.5, 122.7, 122.5, 107.8, 95.1, 89.7, 81.7, 56.1, 16.4, 15.4.

HRMS m/z: Not obtained due to presence of other regioisomer of identical mass.

(*rac*)-6-(methoxymethoxy)-1,7-Dimethyl-1,4-dihydro-1,4-epoxynaphthalene (S21)

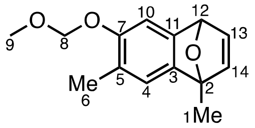

Major regioisomer of band **C**.

^{1}**H NMR** (400 MHz, $CDCl_3$) δ 7.06 (1H, s, H_4), 7.01 (1H, dd, *J*=5.4, 1.9 Hz, H_{13}), 6.96 (1H, s, H_{10}), 6.76 (1H, d, *J*=5.4 Hz, H_{14}), 5.58 – 5.54 (1H, m, H_{12}), 5.15 – 5.13 (2H, m, H_8), 3.48 (3H, s, H_9), 2.19 (3H, s, H_6), 1.90 (3H, s, H_1).

^{13}**C NMR** (101 MHz, $CDCl_3$) δ 152.4, 150.0, 146.0, 144.3 (2×), 122.6, 121.6, 108.9, 95.1, 89.4, 81.9, 56.0, 16.5, 15.4.

HRMS m/z: Not obtained due to presence of other regioisomer of identical mass.

Synthesis of 6-(methoxymethoxy)-4,7-dimethylnaphthalen-1-ol (10) and 7-(methoxymethoxy)-4,6-dimethylnaphthalen-1-ol (1h)

The isomerization reaction was initially conducted with band **A**, which contains the highest proportion of the desired intermediate **S20** (entry 1). To improve the overall corrected yield, band **B** was also subjected to the reaction conditions (entry 2).

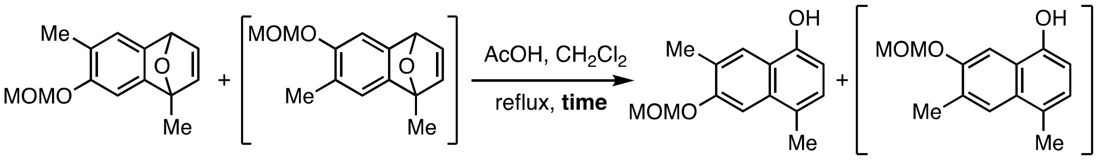

S20 **S21** **10** **1h**

Entry	Substrate	S20:S21	Time/days	Yield 10/%	Yield 1h/%
**1**	**Band A**	3.5:1	6	54	15
**2**	**Band B**	1:1.2	2	27	31

N.B. The desired intermediate **S20** was observed to react more slowly than the undesired intermediate **S21**. The reaction time required for the reaction of band **B** to go to completion was therefore shorter (2 h vs 6 h). We speculate that this is caused by more efficient resonance stabilization of the carbocation intermediate by the electron-donating OMOM group in the reaction of the undesired intermediate **S21** (scheme).

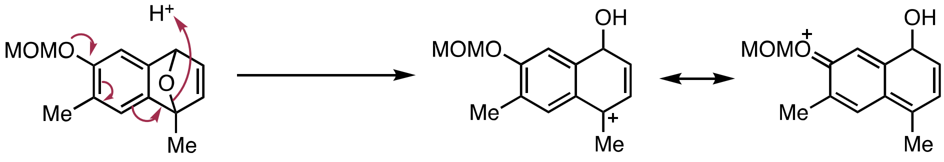

S21

Procedure for entry 1: A round bottom flask fitted with a reflux condenser was charged with band **A** (465 mg, 2.00 mmol). CH_2Cl_2 (20 mL) and AcOH (2 mL) were added. The reaction was heated under reflux and monitored by TLC; full consumption of the starting material was observed after 6 days. The solvent was removed under reduced pressure. The crude product was purified by flash column chromatography (0-10% EtOAc in 40-60 °C petroleum ether) to sequentially afford 6-(methoxymethoxy)-4,7-dimethylnaphthalen-1-ol (**10**) as a light brown solid (250 mg, 1.08 mmol, 54% yield) and 7-(methoxymethoxy)-4,6-dimethylnaphthalen-1-ol (**1h**) as a light brown solid (69.3 mg, 0.298 mmol, 15% yield).

Procedure for entry 2: An analogous procedure was performed for entry 2 using band **B** on a 0.100 mmol scale (23.2 mg band **B**), with a reaction time of 2 days. The crude product was purified by preparative TLC (30% EtOAc in 40-60 °C petroleum ether) to sequentially afford 6-(methoxymethoxy)-4,7-dimethylnaphthalen-1-ol (**10**) as a light brown solid (6.3 mg, 0.027 mmol, 27% yield) and 7-(methoxymethoxy)-4,6-dimethylnaphthalen-1-ol (**1h**) as a light brown solid (7.2 mg, 0.031 mmol, 31% yield).

Calculation of corrected yield of naphthol 10 over two steps:

via band **A**: 33% × 54% = 18% yield (corrected over two steps)

via band **B**: 44% × 27% = 12% yield (corrected over two steps)

Overall: 18% + 12% yield = 30% yield (corrected over two steps).

6-(methoxymethoxy)-4,7-Dimethylnaphthalen-1-ol (10)

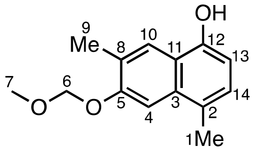

[N.B. A COSY correlation was observed between H_9 and H_{10}. A strong HMBC correlation was observed between H_{10} and C_{12} (δ = 149.5). These observations are consistent only with this structure.]

^{1}H NMR (700 MHz, $CDCl_3$) δ 7.97 (1H, s, H_{10}), 7.39 (1H, s, H_4), 7.03 (1H, d, *J*=7.4 Hz, H_{14}), 6.58 (1H, dd, *J*=7.4, 2.1 Hz, H_{13}), 5.36 (2H, s, H_6), 5.08 (1H, br s, H_O), 3.54 (3H, s, H_7), 2.54 (3H, s, H_1), 2.43 (3H, s, H_9).

^{13}C NMR (176 MHz, $CDCl_3$) δ 154.8, 149.5, 133.7, 127.6, 125.9, 125.6, 123.4, 120.2, 106.6, 105.5, 94.6, 56.3, 19.2, 17.1.

HRMS m/z: $[M{-}H]^-$ calculated for $[C_{14}H_{15}O_3]^-$ 231.1027, found 231.1023. $\Delta = -1.7$ ppm.

7-(methoxymethoxy)-4,6-Dimethylnaphthalen-1-ol (1h)

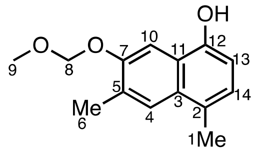

[N.B. A COSY correlation, as well as long range *J*-coupling, was observed between H_6 and H_4. A strong HMBC correlation was observed between H_{10} and C_{12} (δ = 149.1). These observations are consistent only with this structure.]

^{1}H NMR (700 MHz, $CDCl_3$) δ 7.69 (1H, q, *J*=1.1 Hz, H_4), 7.68 (1H, s, H_{10}), 6.98 (1H, dd, *J*=7.5, 1.0 Hz, H_{14}), 6.64 (1H, d, *J*=7.5 Hz, H_{13}), 5.38 (2H, s, H_8), 3.55 (3H, s, H_9), 2.56 (3H, d, *J*=1.0 Hz, H_1), 2.45 (3H, d, *J*=1.1 Hz, H_6).

^{13}C NMR (176 MHz, $CDCl_3$) δ 153.5, 149.1, 129.4, 128.7, 125.7, 125.7, 124.2, 124.1, 107.9, 103.0, 94.4, 56.2, 18.9, 17.3.

HRMS m/z: [M−H]$^-$ calculated for $[C_{14}H_{15}O_3]^-$ 231.1027, found 231.1023. Δ = −1.7 ppm.

Unsuccessful Attempt Towards the Synthesis of 6-(methoxymethoxy)-4,7-dimethylnaphthalen-1-ol (10) and 7-(methoxymethoxy)-4,6-dimethylnaphthalen-1-ol (1h)

The ring opening of other epoxide intermediates to the corresponding naphthol was conducted with a $Cu(OTf)_2$ Lewis acid (see **Synthesis of Starting Materials in Scheme 1**). However, the treatment of the epoxide mixture **S20** and **S21** with $Cu(OTf)_2$ appeared to be incompatible with the OMOM group, resulting in rapid decomposition of the substrate. We note that other Lewis acids such as BF_3 are typical reagents for MOM-deprotection; it seems likely that MOM deprotection is being catalyzed by $Cu(OTf)_2$ in this case, followed by decomposition.

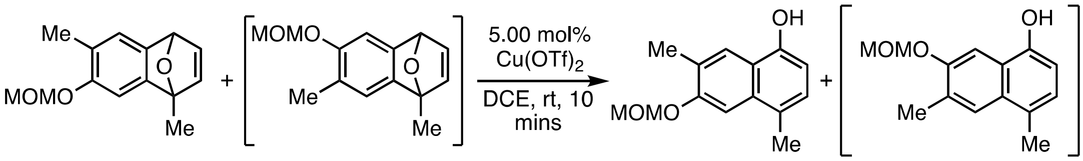

S20 **S21** **10** **1h**

1.1:1

not observed, SM decomposed

Early reports into the isomerization of variously substituted l,4-epoxy-l,4-dihydronaphthalenes used hydrochloric acid.[17] Due to the acid sensitive MOM protecting group, these conditions were also considered unsuitable for our purposes.[18]

Good results were eventually achieved by heating the epoxide mixture **S20** and **S21** with a weak Brønsted acid (AcOH), which could catalyze the epoxide isomerization, while leaving the MOM protecting group intact (*vide supra*).

4-Bromo-1,2-bis(methoxymethoxy)benzene (S18)

The synthesis of substrate 4-bromo-1,2-bis(methoxymethoxy)benzene (**S18**) is shown in **Synthesis of Starting Materials in Scheme 1**.

(*R*)-4-(3,4-bis(methoxymethoxy)phenyl)-6-(methoxymethoxy)-4,7-Dimethylnaphthalen-1(4*H*)-one (11)

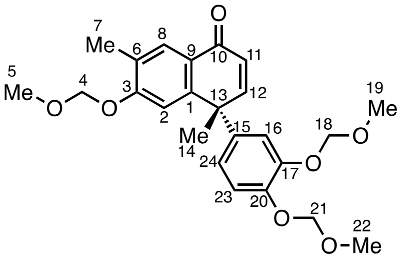

Prepared according to General Procedure B with 6-(methoxymethoxy)-4,7-dimethylnaphthalen-1-ol (**10**) (23.2 mg, 0.100 mmol) and 4-bromo-1,2-bis(methoxymethoxy)benzene (**S18**) (41.6 mg, 0.150 mmol) as the starting materials. Purification by flash column chromatography (0-30% EtOAc in 40-60 °C petroleum ether) afforded the title compound as an orange solid (30.1 mg, 0.070 mmol, 70% yield, 84% *ee*).

^{1}H NMR (400 MHz, $CDCl_3$) δ 7.98 (s, 1H, H_8), 7.08 – 6.99 (m, 2H, H_{16} and H_{23}), 6.88 – 6.83 (m, 1H, H_{12}), 6.82 – 6.77 (m, 1H, H_{24}), 6.71 (s, 1H, H_2), 6.39 – 6.31 (m, 1H, H_{11}), 5.20 – 5.17 (m, 2H, $H_{4/18/21}$), 5.15 – 5.11 (m, 3H, 3×$H_{4/18/21}$), 5.09 – 5.03 (m, 1H, $H_{4'/18'/21'}$), 3.49 – 3.47 (m, 3H, $H_{5/19/22}$), 3.47 – 3.46 (m, 3H, $H_{5/19/22}$), 3.41 – 3.39 (m, 3H, $H_{5/19/22}$), 2.25 (s, 3H, H_7), 1.81 (s, 3H, H_{14}).

^{13}C NMR (101 MHz, $CDCl_3$) δ 184.7, 159.2, 155.4, 149.8, 147.2, 146.6, 137.8, 129.0, 126.9, 125.7, 124.7, 121.5, 116.8, 116.4, 112.0, 96.0, 95.5, 94.2, 56.4, 56.3 (2×), 45.2, 27.5, 16.1.

HRMS m/z: $[M+H]^+$ calculated for $[C_{24}H_{29}O_7]^+$ 429.1908, found 429.1912. Δ = +0.9 ppm.

Chiral SFC Analysis: CHIRALPAK IE (CO_2:MeOH, 90:10, 2.5 mL min^{-1}, 40 °C, 230 nm) indicated 84% *ee*, t_R = 10.95 (major), 11.90 (minor) minutes.

$[\alpha]_D^{25}$ = +195° (c 1.00, $CHCl_3$).

(*R*)-4-(3,4-bis(methoxymethoxy)phenyl)-6-(methoxymethoxy)-4,7-Dimethyl-3,4-dihydronaphthalen-1(2*H*)-one (7)

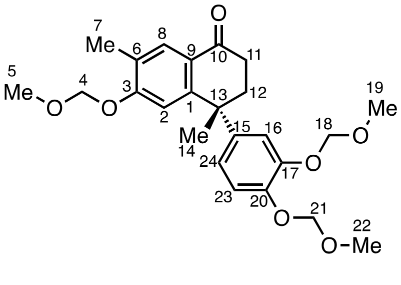

To a solution of (*R*)-4-(3,4-bis(methoxymethoxy)phenyl)-6-(methoxymethoxy)-4,7-dimethylnaphthalen-1(4*H*)-one (**11**) (13.0 mg, 0.0303 mmol, 1.00 equiv., 84% *ee*) in EtOAc (1 mL) was added Pd/C (10 wt % loading Pd, 3.2 mg, 0.0030 mmol, 10 mol%). The vial was capped with a septum and evacuated and backfilled with hydrogen (3×). The reaction was stirred at room temperature under a hydrogen atmosphere for 2 hours, at which point it was judged complete by LCMS. The reaction was filtered through celite, eluting with EtOAc and MeOH, and concentrated under a stream of air. The crude product was purified by preparative TLC (50% EtOAc in 40-60 °C petroleum ether) to afford the title compound as a colorless oil (10.4 mg, 0.024 mmol, 80% yield, 82% *ee*).

^{1}H NMR (700 MHz, $CDCl_3$) δ 7.92 (s, 1H, H_8), 6.99 (d, *J* = 8.5 Hz, 1H, H_{23}), 6.93 (d, *J* = 2.3 Hz, 1H, H_{16}), 6.86 (s, 1H, H_2), 6.56 (dd, *J* = 8.5, 2.3 Hz, 1H, H_{24}), 5.21 – 5.10 (m, 6H, H_4, H_{18} and H_{21}), 3.49 (s, 3H, $H_{5/19/22}$), 3.45 (s, 3H, $H_{5/19/22}$), 3.44 (s, 3H, $H_{5/19/22}$), 2.53 (app dt, *J* = 17.2, 4.8 Hz, 1H, H_{11}), 2.43 – 2.31 (m, 2H, $H_{11'}$ and H_{12}), 2.27 (s, 3H, H_7), 2.24 (ddd, *J* = 13.4, 11.5, 4.2 Hz, 1H, $H_{12'}$), 1.76 (s, 3H, H_{14}).

^{13}C NMR (176 MHz, $CDCl_3$) δ 197.4, 159.9, 150.2, 146.8, 145.8, 141.9, 129.9, 126.5, 126.4, 121.6, 116.5, 116.0, 111.9, 95.8, 95.4, 94.2, 56.2, 56.2, 56.2, 42.5, 39.1, 35.2, 29.4, 15.9.

Chiral SFC Analysis: CHIRALPAK IE (CO_2:MeOH, 90:10, 2.5 mL min^{-1}, 40 °C, 270 nm) indicated 82% *ee*, t_R = 8.97 (major), 10.03 (minor) minutes.

$[\alpha]_D^{25}$ = +67.1° (c 0.69, $CHCl_3$).

^{1}H NMR and ^{13}C data in agreement with the literature (see below).[19]

Comparison of ^{1}H NMR data of 7 with the literature

H	Our data/ppm	Literature data/ ppm[19]
**8**	7.92	7.92
**23**	6.99	6.99
**16**	6.93	6.93
**2**	6.86	6.86
**24**	6.56	6.56
**4, 18, 21**	5.21 – 5.10	5.20-5.13
**5/19/22**	3.49	3.50
**5/19/22**	3.45	3.46
**5/19/22**	3.44	3.45

**11**	2.53	2.56-2.50
**11' and 12**	2.43 – 2.31	2.42-2.32
**7**	2.27	2.27
**12'**	2.24	2.26-2.21
**14**	1.76	1.76

Comparison of ^{13}C NMR data of 7 with the literature

**Our data/ppm**	**Literature data/ ppm**[19]
197.4	197.5
159.9	159.9
150.2	150.2
146.8	146.8
145.8	145.8
141.9	141.9
129.9	129.9
126.5	126.5
126.4	126.4
121.6	121.6
116.5	116.5
116.0	116.0
111.9	111.8
95.8	95.8
95.4	95.4
94.2	94.1
56.2	56.3
56.2	56.2
56.2	56.2
42.5	42.5
39.1	39.1
35.2	35.2
29.4	29.4
15.9	15.9

Direct Arylation of Naphthalen-1-ol

4-Phenylnaphthalen-1-ol (12a)

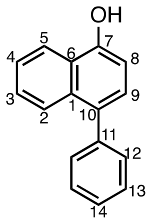

A vial containing a magnetic stirrer bar was sequentially charged with naphthalen-1-ol (14.4 mg, 0.100 mmol, 1.00 equiv.), (*rac*)-sSPhos (3.8 mg, 0.0075 mmol, 7.50 mol%), K_3PO_4 (31.8 mg, 0.150 mmol, 1.50 equiv.), Pd_2dba_3 (1.1 mg, 0.00125 mmol, 1.25 mol%), and bromobenzene (23.6 mg, 0.150 mmol, 1.50 equiv.). The vial was sealed, and evacuated and backfilled with N_2 (4×). PhMe (0.50 mL) was added under nitrogen. The reaction was stirred at 90 °C and 900 rpm for 20 h. Upon completion, the reaction mixture was filtered through celite, eluting with EtOAc. The filtrate was concentrated under a stream of air. Purification by flash column chromatography (0-5% EtOAc in 40-60 °C petroleum ether) afforded the title compound as a yellow solid (15.1 mg, 0.069 mmol, 69%).

[N.B. Due to the moderate volatility of bromobenzene (b.p. 156 °C), the vial was not left under vacuum for prolonged periods when performing N_2/evacuation cycles with this substrate.]

[N.B. No doubly arylated dearomatized product was observed.]

^{1}H NMR (400 MHz, $CDCl_3$) δ 8.28 (1H, d, *J*=8.3 Hz, H_5), 7.89 (1H, d, *J*=8.4 Hz, H_2), 7.57 – 7.36 (7H, m, H_3, H_4, H_{12}, H_{13}, H_{14}), 7.27 (1H, d, *J*=7.9 Hz, H_9), 6.87 (1H, d, *J*=7.9 Hz, H_8), 5.36 (1H, br s, H_O).

^{13}C NMR (101 MHz, $CDCl_3$) δ 151.0, 140.9, 133.4, 132.8, 130.4, 128.4, 127.1, 127.0, 126.7, 126.1, 125.3, 124.6, 122.0, 108.3.

Data in agreement with the literature.[20]

4-(4-methoxyphenyl)Naphthalen-1-ol (12b)

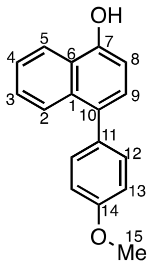

N.B. Reaction performed on a 1 mmol scale.

A vial containing a magnetic stirrer bar was sequentially charged with naphthalen-1-ol (144 mg, 1.00 mmol, 1.00 equiv.), (*rac*)-sSPhos (38.4 mg, 0.0750 mmol, 7.50 mol%), K_3PO_4 (318 mg, 1.50 mmol, 1.50 equiv.), Pd_2dba_3 (11.4 mg, 0.0125 mmol, 1.25 mol%), and 1-bromo-4-methoxybenzene (281 mg, 1.50 mmol, 1.50 equiv.). The vial was sealed, and evacuated and backfilled with N_2 (4×). PhMe (5.0 mL) was added under nitrogen. The reaction was stirred at 90 °C and 900 rpm for 20 h. Upon completion, the reaction mixture was filtered through celite, eluting with EtOAc. The filtrate was concentrated under a stream of air. Purification by flash column chromatography (0-10% EtOAc in hexane) afforded the title compound as an off-white solid (195 mg, 0.779 mmol, 78%).

[N.B. No doubly arylated dearomatized product was observed.]

^{1}H NMR (500 MHz, $CDCl_3$) δ 8.27 (1H, d, *J*=8.2 Hz, H_5), 7.89 (1H, d, *J*=8.5 Hz, H_2), 7.51 (1H, ddd, *J*=8.2, 6.7, 1.3 Hz, H_4), 7.46 (1H, ddd, *J*=8.5, 6.7, 1.4 Hz, H_3), 7.40 (2H, d, *J*=8.1 Hz, H_{12}), 7.24 (1H, d, *J*=7.7 Hz, H_9), 7.03 (2H, d, *J*=8.1 Hz, H_{13}), 6.86 (1H, d, *J*=7.7 Hz, H_8), 5.39 (1H, br s, H_O), 3.90 (3H, s, H_{15}).

^{13}C NMR (126 MHz, $CDCl_3$) δ 158.8, 150.8, 133.3, 133.0, 133.0, 131.4, 126.9, 126.6, 126.2, 125.2, 124.6, 121.9, 113.8, 108.3, 55.5.

Data in agreement with the literature.[21]

4-(6-methoxypyridin-3-yl)Naphthalen-1-ol (12c)

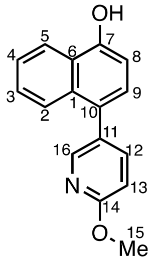

A vial containing a magnetic stirrer bar was sequentially charged with naphthalen-1-ol (14.4 mg, 0.100 mmol, 1.00 equiv.), (*rac*)-sSPhos (3.8 mg, 0.0075 mmol, 7.50 mol%), K_3PO_4 (31.8 mg, 0.150 mmol, 1.50 equiv.), Pd_2dba_3 (1.1 mg, 0.00125 mmol, 1.25 mol%), and 5-bromo-2-methoxypyridine (28.2 mg, 0.150 mmol, 1.50 equiv.). The vial was sealed, and evacuated and backfilled with N_2 (4×). PhMe (0.50 mL) was added under nitrogen. The reaction was stirred at 90 °C and 900 rpm for 20 h. Upon completion, the reaction mixture was filtered through celite, eluting with EtOAc. The filtrate was concentrated under a stream of air. Purification by flash column chromatography (0-30% EtOAc in heptane) afforded the title compound as a colorless solid (21.5 mg, 0.086 mmol, 86%).

^{1}H NMR (500 MHz, $CDCl_3$) δ 8.32 (1H, dd, *J*=8.3, 1.4 Hz, H_5), 8.27 (1H, dd, *J*=2.4, 0.8 Hz, H_{16}), 7.78 (1H, d, *J*=8.5, H_2), 7.72 (1H, dd, *J*=8.5, 2.4 Hz, H_{12}), 7.51 (1H, dd, *J*=8.3, 6.8 Hz, H_4), 7.46 (1H, ddd, *J*=8.5, 6.8, 1.4 Hz, H_3), 7.20 (1H, d, *J*=7.6 Hz, H_9), 6.91 – 6.88 (2H, m, H_8 and H_{13}), 4.05 (3H, s, H_{15}). [N.B. The O-H peak was not observed].

^{13}C NMR (126 MHz, $CDCl_3$) δ 163.4, 152.1, 147.3, 141.1, 133.0, 130.0, 128.7, 127.5, 126.9, 125.4, 125.3, 124.9, 122.4, 110.4, 108.2, 53.9.

HRMS m/z: $[M+H]^+$ calculated for $[C_{16}H_{14}NO_2]^+$ 252.1019, found 252.1015. Δ = −1.6 ppm.

4-(1*H*-indol-5-yl)Naphthalen-1-ol (12d)

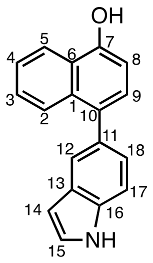

N.B. Reaction performed on a 1 mmol scale.

A vial containing a magnetic stirrer bar was sequentially charged with naphthalen-1-ol (144 mg, 1.00 mmol, 1.00 equiv.), (*rac*)-sSPhos (38.4 mg, 0.0750 mmol, 7.50 mol%), K_3PO_4 (318 mg, 1.50 mmol, 1.50 equiv.), Pd_2dba_3 (11.4 mg, 0.0125 mmol, 1.25 mol%), and 5-bromo-1*H*-indole (294 mg, 1.50 mmol, 1.50 equiv.). The vial was sealed, and evacuated and backfilled with N_2 (4×). PhMe (5.0 mL) was added under nitrogen. The reaction was stirred at 90 °C and 900 rpm for 20 h. Upon completion, the reaction mixture was filtered through celite, eluting with EtOAc. The filtrate was concentrated under a stream of air. Purification by flash column chromatography

(0-20% EtOAc in 40-60 °C petroleum ether) afforded the title compound as a yellow solid (172 mg, 0.663 mmol, 66%).

[N.B. No doubly arylated dearomatized product was observed.]

^{1}H NMR (700 MHz, $CDCl_3$) δ 8.26 (ddd, *J* = 8.4, 1.4, 0.7 Hz, 1H, H_5), 8.24 (br s, 1H, H_N), 7.95 (app dt, *J* = 8.3, 1.0 Hz, 1H, H_2), 7.73 – 7.70 (m, 1H, H_{12}), 7.53 – 7.47 (m, 2H, H_4 and H_{17}), 7.42 (ddd, *J* = 8.3, 6.7, 1.3 Hz, 1H, H_3), 7.32 (d, *J* = 7.6 Hz, 1H, H_9), 7.30 (dd, *J* = 8.3, 1.7 Hz, 1H, H_{18}), 7.29 (dd, *J* = 3.1, 2.5 Hz, 1H, H_{15}), 6.89 (d, *J* = 7.6 Hz, 1H, H_8), 6.61 (ddd, *J* = 3.1, 2.0, 1.0 Hz, 1H, H_{14}), 5.22 (s, 1H, H_O).

^{13}C NMR (176 MHz, $CDCl_3$) δ 150.6, 135.1, 134.6, 133.4, 132.7, 128.1, 127.2, 126.7, 126.4, 125.1, 125.0, 124.8, 124.5, 122.3, 121.8, 110.7, 108.3, 103.0.

HRMS m/z: $[M{-}H]^-$ calculated for $[C_{18}H_{12}NO]^+$ 258.0924, found 258.0933. Δ = +3.5 ppm.

4-(9,9-dibutyl-9*H*-fluoren-2-yl)Naphthalen-1-ol (12e)

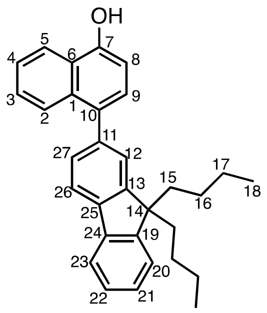

A vial containing a magnetic stirrer bar was sequentially charged with naphthalen-1-ol (14.4 mg, 0.100 mmol, 1.00 equiv.), (*rac*)-sSPhos (3.8 mg, 0.0075 mmol, 7.50 mol%), K_3PO_4 (31.8 mg, 0.150 mmol, 1.50 equiv.), Pd_2dba_3 (1.1 mg, 0.00125 mmol, 1.25 mol%), and 2-bromo-9,9-dibutyl-9*H*-fluorene (53.6 mg, 0.150 mmol, 1.50 equiv.). The vial was sealed, and evacuated and backfilled with N_2 (4×). PhMe (0.50 mL) was added under nitrogen. The reaction was stirred at 90 °C and 900 rpm for 20 h. Upon completion, the reaction mixture was filtered through celite, eluting with EtOAc. The filtrate was concentrated under a stream of air. Purification by flash column chromatography (0-20% EtOAc in heptane) afforded the title compound as a brown solid (33.0 mg, 0.078 mmol, 78%).

^{1}H NMR (500 MHz, $CDCl_3$) δ 8.30 (1H, dd, *J*=8.4, 2.3 Hz, H_5), 7.95 (1H, dd, *J*=8.7, 3.1 Hz, H_2), 7.81 (1H, d, *J*=8.4 Hz, H_{Ar}), 7.77 (1H, d, *J*=7.3 Hz, H_{Ar}), 7.53 (1H, ddd, *J*=8.4, 6.9, 1.7 Hz, H_4), 7.49 – 7.43 (3H, m, H_3, 2H_{Ar}), 7.41 – 7.31 (4H, m, H_9 and 3H_{Ar}), 6.92 (1H, d, *J*=7.6 Hz, H_8), 5.46

(1H, br s, H_O), 2.05 – 1.94 (4H, m, H_{15}), 1.19 – 1.05 (4H, m, H_{17}), 0.81 – 0.67 (10H, m, H_{16} and H_{18}).

^{13}C NMR (126 MHz, $CDCl_3$) δ 151.1, 151.0, 150.8, 141.1, 140.1, 139.6, 133.9, 133.0, 129.0, 127.1, 126.9 (2×), 126.6, 126.2, 125.3, 125.1, 124.7, 123.0, 122.0, 119.8, 119.6, 108.3, 55.2, 40.2, 26.2, 23.2, 14.0.

HRMS m/z: $[M]^+$ calculated for $[C_{31}H_{32}O]^+$ 420.2448, found 420.2432. Δ = −3.8 ppm.

Unsuccessful Preliminary Investigations into Direct Arylation to form Axially Chiral Biaryls

In the direct arylation, increasing steric bulk on both reaction components should lead to products containing a stereogenic axis. We were curious as to whether any catalyst-controlled atroposelectivity could be obtained. Attempted formation of biaryls **S22** and **S23** from 3-methoxynaphthalen-1-ol afforded no desired substrate, with likely decomposition of the electron-rich starting material under the reaction conditions.

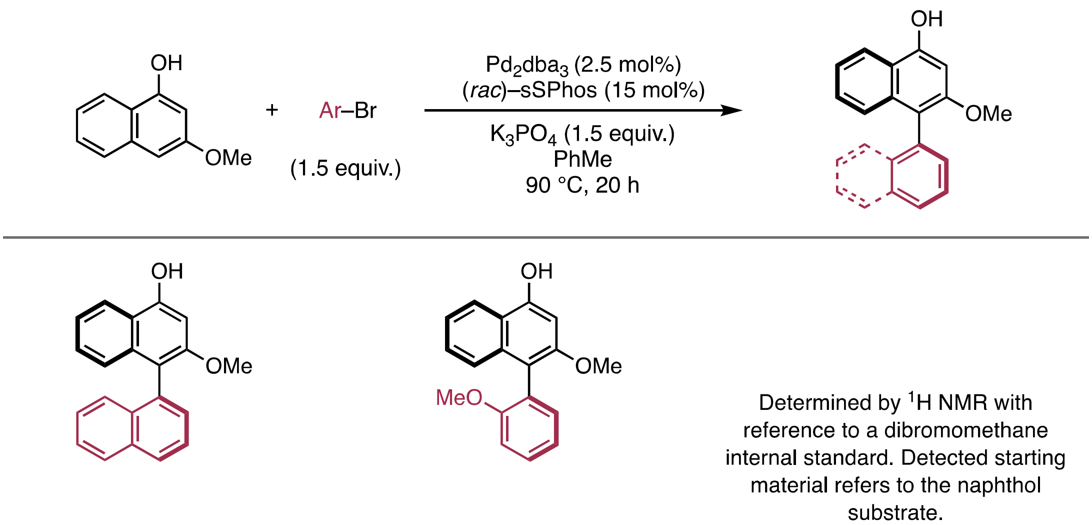

S22, no product detected **S23, no product detected**

Investigation of Intramolecular 2-Naphthol Substrate

Considering the poor enantioselectivity obtained when 1-methylnaphthalen-2-ol was used as the substrate for dearomatization (Scheme 3c), we were curious to probe whether similarly low enantioselectivity would be obtained in an intramolecular variant of this reaction. A similarly low 12% *ee* was obtained, consistent with our hypothesis that the directed transition state cannot be reached in the dearomatization of 2-naphthol substrates.

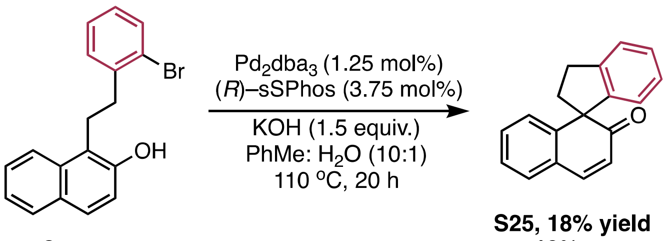

S24 **S25, 18% yield** **12% *ee***

N.B. In this transformation, we elected to use optimized conditions for the intramolecular reaction, as reported in our previous publication.[2] The catalytic system is the same for both sets of optimized conditions.

1-(2-bromophenethyl)Naphthalen-2-ol (**S24**)

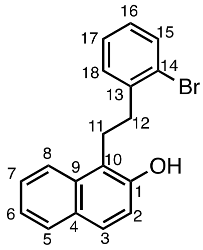

1-(2-bromophenethyl)naphthalen-2-ol (**S24**) was prepared according to the literature.[22]

^{1}H NMR (400 MHz, $CDCl_3$) δ 8.06 (dd, *J* = 8.5, 1.0 Hz, 1H, H_5), 7.80 (d, *J* = 8.0 Hz, 1H, H_8), 7.67 (d, *J* = 8.7 Hz, 1H, H, H_3), 7.59 (d, *J* = 7.5 Hz, 1H, $H_{15/18}$), 7.52 (ddd, *J* = 8.5, 6.7, 1.4 Hz, 1H, H_6), 7.36 (ddd, *J* = 8.0, 6.7, 1.0 Hz, 1H, H_7), 7.27 – 7.21 (m, 2H, $H_{15/18}$ and $H_{16/17}$), 7.13 – 7.06 (m, 2H, H_2 and $H_{16/17}$), 4.99 (s, 1H, H_O), 3.37 – 3.28 (m, 2H, H_{11}), 3.12 – 3.03 (m, 2H, H_{12}).

^{13}C NMR (101 MHz, $CDCl_3$) δ 151.1, 141.2, 133.3, 133.0, 130.8, 129.6, 128.8, 128.3, 128.1, 127.8, 126.7, 124.4, 123.3, 122.9, 118.7, 117.9, 36.4, 25.9.

Data in agreement with the literature.[22]

2,3-Dihydro-2'*H*-spiro[indene-1,1'-naphthalen]-2'-one (S25)

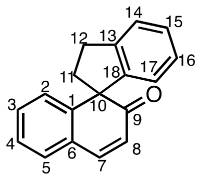

A vial containing a magnetic stirrer bar was charged with 1-(2-bromophenethyl)naphthalen-2-ol (**S24**) (32.7 mg, 0.100 mmol, 1.00 equiv.), crushed KOH (8.4 mg, 0.15 mmol, 1.5 equiv.), (*R*)–

sSPhos (1.9 mg, 0.00375 mmol, 3.75 mol%), and Pd_2dba_3 (1.1 mg, 0.00125 mmol, 1.25 mol%). The vial was sealed, and evacuated and backfilled with N_2 (3×). PhMe (0.50 mL) and H_2O (50 µL) were added under nitrogen. The reaction was stirred at 110 °C and 900 rpm for 20 h. Upon completion, the vial was opened, and the solvent was removed under a stream of air. Sat. NH_4Cl (aq.) (0.5 mL) was added, and the aqueous layer was extracted with chloroform (3 × 0.5 mL). The combined organic extracts were dried over $MgSO_4$, filtered, and concentrated under reduced pressure. The crude product was purified by preparatory TLC (10% EtOAc in 40-60 °C petroleum ether) to afford the title compound as a yellow oil (4.5 mg, 0.018 mmol, 18% yield, 12% *ee*).

^{1}H NMR (500 MHz, $CDCl_3$) δ 7.53 (d, *J* = 9.9 Hz, 1H, H_7), 7.40 – 7.32 (m, 2H, H_5 and H_{14}), 7.32 – 7.20 (m, 3H, H_3, H_4, and H_{15}), 7.10 (app t, *J* = 7.5 Hz, 1H, H_{16}), 7.05 – 6.96 (m, 1H, H_2), 6.77 (d, *J* = 7.7 Hz, 1H, H_{17}), 6.19 (d, *J* = 9.9 Hz, 1H, H_8), 3.34 (app dt, *J* = 15.9, 8.0 Hz, 1H, H_{12}), 3.15 (ddd, *J* = 15.9, 8.9, 4.3 Hz, 1H, $H_{12'}$), 2.81 (ddd, *J* = 13.0, 8.7, 4.3 Hz, 1H, H_{11}), 2.38 (ddd, *J* = 13.0, 8.9, 7.4 Hz, 1H, $H_{11'}$).

^{13}C NMR (126 MHz, $CDCl_3$) δ 202.5, 146.8, 145.8, 145.4, 145.3, 130.3, 129.6, 129.3, 128.2, 127.9, 127.1, 127.0, 125.0, 124.7, 124.7, 65.4, 41.9, 31.7.

Chiral SFC Analysis: CHIRALPAK IK (CO_2:MeOH, 85:15, 2.5 mL min^{-1}, 40 °C, 300 nm) indicated 12% *ee*, t_R = 5.23 (major), 6.24 (minor) minutes.

^{1}H NMR and ^{13}C NMR data in agreement with the literature.[22]

Synthesis of Unsuccessful Starting Materials

N.B. 4-(4-methoxyphenyl)naphthalen-1-ol (**12b**), described in **Direct Arylation of Naphthalen-1-ol**, was also used as a substrate in **Unsuccessful Nucleophiles** (*vide supra*).

1,3-Dimethylnaphthalen-2-ol (S26)

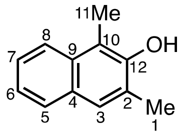

1,3-Dimethylnaphthalen-2-ol (**S26**) was prepared according to the literature.[11]

^{1}H NMR (700 MHz, $CDCl_3$) δ 7.87 (dd, J = 8.4, 1.1 Hz, 1H, H_5), 7.71 (d, J = 8.0 Hz, 1H, H_8), 7.50 (s, 1H, H_3), 7.44 (ddd, J = 8.4, 6.8, 1.4 Hz, 1H, H_6), 7.32 (ddd, J = 8.0, 6.8, 1.1 Hz, 1H, H_7), 4.89 (s, 1H, H_O), 2.55 (s, 3H, H_{12}), 2.44 (d, J = 1.1 Hz, 3H, H_{11}).

^{13}C NMR (176 MHz, $CDCl_3$) δ 150.2, 132.7, 129.2, 127.8, 127.2, 125.6, 125.5, 123.3, 123.1, 114.5, 17.1, 10.8.

Data in agreement with the literature.[11]

3-Methoxy-4-methylphenol (S27)

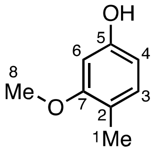

To a solution of 4-hydroxy-2-methoxybenzaldehyde (761 mg, 5.00 mmol, 1.00 equiv.) in EtOH (20 mL) and AcOH (3 mL) was added Pd/C (10 wt % loading Pd, 202 mg, 0.190 mmol, 3.80 mol%). The vial was capped with a septum and evacuated and backfilled with hydrogen (3×). The reaction was stirred at room temperature under a hydrogen atmosphere for 20 h. The reaction was filtered through celite, eluting with EtOAc, and concentrated under reduced pressure. The crude product was purified by flash column chromatography (10% EtOAc in 40-60 °C petroleum ether) to afford the title compound as a yellow oil (295 mg, 2.14 mmol, 43% yield).

^{1}H NMR (400 MHz, $CDCl_3$) δ 6.96 (1H, d, J=8.0 Hz, H_3), 6.39 (1H, d, J=2.0 Hz, H_6), 6.37 – 6.32 (1H, m, H_4), 5.72 – 5.00 (1H, m, H_O), 3.94 – 3.59 (3H, m, H_8), 2.15 (3H, d, J=2.2 Hz, H_1).

^{13}C NMR (101 MHz, $CDCl_3$) δ 158.7, 154.7, 130.9, 118.8, 106.6, 98.9, 55.4, 15.5.

Data in agreement with the literature.[23]

Synthesis of 2,4-Dimethylnaphthalen-1-ol (S28)

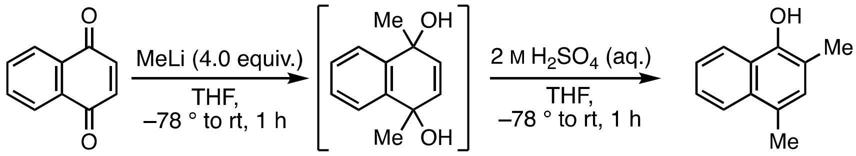

S28, 17% yield

Based on the procedure reported by Urbano and co-workers.[24] A heat gun-dried flask containing a magnetic stirrer bar was charged with 1,4-naphthoquinone (501 mg, 3.17 mmol, 1.00 equiv.) and put under nitrogen. THF (64 mL) was added, the solution was cooled to −78 °C, and MeLi (1.6 M in Et_2O, 7.9 mL, 13 mmol, 4.0 equiv.) was added dropwise. The reaction was warmed to room temperature, stirred for 1 h, and cooled back down to −78 °C. H_2SO_4 (aq.) (2 M, 75 ml) was added, and the reaction was warmed to room temperature and stirred for an additional 1 h. The THF was removed under a stream of air, and the aqueous residue extracted with CH_2Cl_2 (3×), dried over Na_2SO_4, concentrated under reduced pressure, and purified by flash column chromatography (0-4% EtOAc in 40-60 °C petroleum ether) to afford the title compound as an off-white solid (93 mg, 0.54 mmol, 17% yield).

2,4-Dimethylnaphthalen-1-ol (S28)

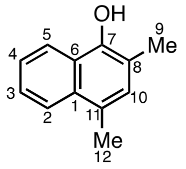

^{1}H NMR (400 MHz, $CDCl_3$) δ 7.96 – 7.89 (2H, m, H_2 and H_5), 7.51 (1H, ddd, *J*=8.6, 6.7, 1.3 Hz, H_4), 7.38 (1H, ddd, *J*=8.0, 6.7, 1.3 Hz, H_3), 6.93 (1H, s, H_{10}), 4.80 (1H, s, H_O), 2.64 (3H, s, H_{12}), 2.51 (3H, s, H_9).

^{13}C NMR (101 MHz, $CDCl_3$) δ 150.1, 134.2, 134.0, 128.6, 126.2, 124.7, 123.8, 123.1, 118.6, 113.1, 19.4, 10.5.

Data in agreement with the literature.[24]

2-Chloro-4-methylnaphthalen-1-ol (S29)

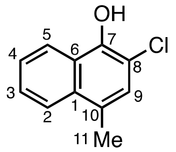

Based on the procedure reported by Ishihara and co-workers.[25] A flask was charged with 4-methylnaphthalen-1-ol (158 mg, 1.00 mmol, 1.00 equiv.), NaCl (58.4 mg, 1.00 mmol, 1.00 equiv.), EtOAc (10 mL) and H_2O (10 mL). Oxone (615 mg, 1.00 mmol, 1.00 equiv.) was added, and the reaction was stirred for 16 h at room temperature. An additional portion of NaCl (58.4 mg, 1.00 mmol, 1.00 equiv.) was added, and the reaction was stirred for a further 1 h at room

temperature. Upon completion, the reaction mixture was poured onto sat. $Na_2S_2O_3$ (aq.) and extracted with EtOAc (2×). The combined organic extracts were washed with brine, dried over Na_2SO_4, concentrated under reduced pressure, and purified by flash column chromatography (0-5% EtOAc in 40-60 °C petroleum ether) to afford the title compound as a brown solid (58.3 mg, 0.303 mmol, 30% yield).

^{1}H NMR (400 MHz, $CDCl_3$) δ 8.29 – 8.21 (1H, m, H_5), 7.96 – 7.84 (1H, m, H_2), 7.58 – 7.49 (2H, m, H_3 and H_4), 7.21 (1H, s, H_9), 5.85 (1H, br s, H_O), 2.59 (3H, s, H_{11}).

^{13}C NMR (101 MHz, $CDCl_3$) δ 145.6, 132.3, 127.6, 126.6, 126.1, 125.9, 124.8, 124.3, 122.7, 112.9, 18.7.

Data in agreement with the literature.[25]

***tert*-Butyl (4-methylnaphthalen-1-yl)carbamate (S30)**

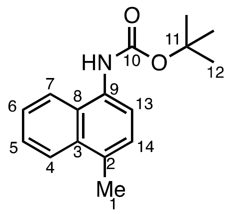

Based on the procedure reported by Augustine and co-workers.[26] A flask was charged with propylphosphonic anhydride solution (50% w/w in EtOAc, 7.00 g, 6.54 mL, 11.0 mmol, 1.10 equiv.), 4-methyl-1-naphthoic acid (1.86 g, 10.0 mmol, 1.00 equiv.), trimethylsilyl azide (1.27 g, 1.45 mL, 11.0 mmol, 1.10 equiv), and Et_3N (1.52 g, 2.09 mL, 15.0 mmol, 1.50 equiv.). THF (10 mL) and ^{t}BuOH (889 mg, 1.14 ml, 12.0 mmol, 1.20 equiv.) were added, and the reaction was stirred under reflux for 1.5 h under nitrogen, at which point it was judged complete by TLC. The mixture was concentrated, diluted with H_2O (25 mL), and extracted with EtOAc (2×). The combined organic extracts were washed with sat. $NaHCO_3$ (aq.) and brine, dried over Na_2SO_4, and concentrated under reduced pressure. The residue was filtered through a plug of silica (eluent: 5% EtOAc in hexane) and concentrated under reduced pressure, to afford the title compound as a yellow solid (1.52 g, 5.91 mmol, 59% yield).

^{1}H NMR (400 MHz, DMSO-d_6) δ 9.10 (1H, s, H_N), 8.07 – 7.94 (2H, m, 2×$H_{4/5/6/7}$), 7.60 – 7.48 (2H, m, 2×$H_{4/5/6/7}$), 7.40 (1H, d, J=7.6 Hz, H_{13}), 7.31 (1H, d, J=7.6 Hz, H_{14}), 2.61 (3H, s, H_1), 1.48 (9H, s, H_{12}).

^{13}C NMR (101 MHz, DMSO-d_6) δ 154.2, 132.5, 132.4, 130.8, 128.5, 126.1, 125.8, 125.4, 124.3, 123.4, 121.4, 78.7, 28.2, 18.9.

Data in agreement with the literature.[26]

***N*-(1,2-dihydroacenaphthylen-5-yl)-4-Methylbenzenesulfonamide (S31)**

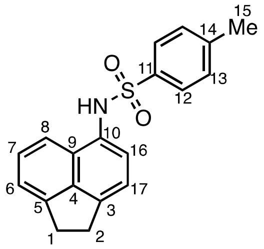

A flask was charged with 1,2-dihydroacenaphthylen-5-amine (169 mg, 1.00 mmol, 1.00 equiv.) and pyridine (5 mL), and cooled to 0 °C. 4-Methylbenzenesulfonyl chloride (248 mg, 1.30 mmol, 1.30 equiv.) was added, and the reaction was stirred at room temperature for 48 h. The reaction mixture was concentrated under reduced pressure, diluted with water, and extracted with CH_2Cl_2 (2×). The combined organic extracts were dried over $MgSO_4$, filtered, concentrated under reduced pressure, and purified by flash column chromatography (10-30% EtOAc in heptane) to afford the title compound as a brown solid (90.0 mg, 0.278 mmol, 28% yield).

^{1}H NMR (500 MHz, DMSO-d_6) δ 7.62 (2H, d, J=8.4 Hz, H_{12}), 7.43 – 7.39 (1H, m, H_8), 7.36 (1H, dd, J=8.4, 6.7 Hz, H_7), 7.30 (1H, d, J=7.4 Hz, H_{16}), 7.24 (1H, dd, J=6.7, 1.1 Hz, H_6), 7.18 – 7.12 (3H, m, H_{13} and H_{17}), 6.66 (1H, br s, H_N), 3.41 – 3.36 (2H, m, H_1), 3.36 – 3.30 (2H, m, H_2), 2.34 (3H, s, H_{15}).

^{13}C NMR (126 MHz, $CDCl_3$) δ 146.4, 145.3, 143.7, 140.1, 136.7, 129.6, 128.5, 127.8, 127.5, 127.5, 124.7, 119.9, 119.1, 117.1, 30.8, 30.1, 21.6.

HRMS m/z: $[M+Na]^+$ calculated for $[C_{19}H_{17}NNaO_2S]^+$ 346.0872, found 346.0882. Δ = +2.9 ppm.

References

1. Pearce-Higgins, R.; Hogenhout, L. N.; Docherty, P. J.; Whalley, D. M.; Chuentragool, P.; Lee, N.; Lam, N. Y. S.; McGuire, T. M.; Valette, D.; Phipps, R. J., An Enantioselective Suzuki–Miyaura Coupling To Form Axially Chiral Biphenols. *J. Am. Chem. Soc.* **2022,** *144*, 15026-15032.

2. Kadarauch, M.; Whalley, D. M.; Phipps, R. J., sSPhos: A General Ligand for Enantioselective Arylative Phenol Dearomatization via Electrostatically-Directed Palladium Catalysis. *J. Am. Chem. Soc.* **2023,** *145*, 25553-25558.

3. Zalesskiy, S. S.; Ananikov, V. P., $Pd_2(dba)_3$ as a Precursor of Soluble Metal Complexes and Nanoparticles: Determination of Palladium Active Species for Catalysis and Synthesis. *Organometallics* **2012,** *31*, 2302-2309.

4. Kraszewski, K.; Tomczyk, I.; Kalek, M., Intermolecular enantioselective dearomatizing *para*-methoxylation of phenols using 2-iodoresorcinol/lactamide catalysts. *Tetrahedron Lett.* **2022,** *108*.

5. Hill, J.; Tam, W., Palladium/Lewis Acid Cocatalyzed Ring-Opening Reactions of Unsymmetrical Oxabenzonobornadienes with Oximes. *J. Org. Chem.* **2019,** *84*, 8309-8314.

6. Maksimovic, I.; Finkin-Groner, E.; Fukase, Y.; Zheng, Q.; Sun, S.; Michino, M.; Huggins, D. J.; Myers, R. W.; David, Y., Deglycase-activity oriented screening to identify DJ-1 inhibitors. *RSC Med. Chem.* **2021,** *12*, 1232-1238.

7. Odajima, M.; Fukui, N.; Shinokubo, H., Dinaphthooxepine Bisimide Undergoes Oxygen Extrusion Reaction upon Electron Injection at Room Temperature. *Org. Lett.* **2023,** *25*, 282-287.

8. Shao, Z.; Peng, F.; Fan, B.; Pu, X.; Li, P.; Zhang, H., $Cu(OTf)_2$-Catalyzed Isomerization of 7-Oxabicyclic Alkenes: A Practical Route to the Synthesis of 1-Naphthol Derivatives. *Synthesis* **2008,** *2008*, 3043-3046.

9. Greenland, H.; Pinhey, J. T.; Sternhell, S., Synthesis and Autoxidation of 2,3,4-Trimethylnaphthalen-1-ol and Related Naphthalen-1-ols. *Aust. J. Chem.* **1987,** *40*, 325-331.

10. Pilkington, L. I.; Barker, D., Total Synthesis of (–)-Isoamericanin A and (+)-Isoamericanol A. *Eur. J. Org. Chem.* **2014,** *2014*, 1037-1046.

11. Xu, R.-Q.; Yang, P.; Tu, H.-F.; Wang, S.-G.; You, S.-L., Palladium(0)-Catalyzed Intermolecular Arylative Dearomatization of β-Naphthols. *Angew. Chem. Int. Ed.* **2016,** *55*, 15137-15141.

12. Friess, M.; Sahrawat, A. S.; Kerschbaumer, B.; Wallner, S.; Torvisco, A.; Fischer, R.; Gruber, K.; Macheroux, P.; Breinbauer, R., Asymmetric Synthesis of Chiral 2-Cyclohexenones with Quaternary Stereocenters via Ene-Reductase Catalyzed Desymmetrization of 2,5-Cyclohexadienones. *ACS Catal.* **2024,** *14*, 7256-7266.

13. Howarth, A.; Ermanis, K.; Goodman, J. M., DP4-AI automated NMR data analysis: straight from spectrometer to structure. *Chem. Sci.* **2020,** *11*, 4351-4359.

14. Lautens, M.; Rovis, T., Selective functionalization of 1,2-dihydronaphthalenols leads to a concise, stereoselective synthesis of sertraline. *Tetrahedron* **1999,** *55*, 8967-8976.

15. Felber, B.; Diederich, F., Synthesis of Dendritic Metalloporphyrins with Distal H-Bond Donors as Model Systems for Hemoglobin. *Helv. Chim. Acta* **2005,** *88*, 120-153.

16. Berliner, M.; Belecki, K., Synthesis of Alpha-Halo Ethers from Symmetric Acetals and in Situ Methoxymethylation of an Alcohol. *Org. Synth.* **2007,** *84*, 102-110.

17. Wolthuis, E.; Bossenbroek, B.; DeWall, G.; Geels, E.; Leegwater, A., Reactions of Methyl-substituted 1,4-Epoxy-1,4-dihydronaphthalenes. *J. Org. Chem.* **1963,** *28*, 148-152.

18. Schelhaas, M.; Waldmann, H., Protecting Group Strategies in Organic Synthesis. *Angew. Chem. Int. Ed.* **1996,** *35*, 2056-2083.

19. Makino, K.; Fukuda, R.; Sueki, S.; Anada, M., Total Synthesis of Alanense A through an Intramolecular Friedel–Crafts Alkylation. *J. Org. Chem.* **2024,** *89*, 2050-2054.

20. Chumillas, S.; Loharch, S.; Beltrán, M.; Szewczyk, M. P.; Bernal, S.; Puertas, M. C.; Martinez-Picado, J.; Alcamí, J.; Bedoya, L. M.; Marchán, V.; Gallego, J., Exploring the HIV-1 Rev Recognition Element (RRE)–Rev Inhibitory Capacity and Antiretroviral Action of Benfluron Analogs. *Molecules* **2023,** *28*, 7031.

21. Zhang, M. Y.; Barrow, R. A., Accessing Polyoxygenated Dibenzofurans via the Union of Phenols and o-Benzoquinones: Rapid Syntheses of Metabolites Isolated from Ribes takare. *Org. Lett.* **2017,** *19*, 2302-2305.

22. Xu, R.-Q.; Yang, P.; You, S.-L., Pd(0)-Catalyzed intramolecular arylative dearomatization of β-naphthols. *Chem. Commun.* **2017,** *53*, 7553-7556.

23. Wang, B.; Gao, R.; Zhang, D.; Zeng, Y.; Zhang, F.; Yan, X.; Li, Y.; Chen, L., Ceria-promoted Co@NC catalyst for biofuel upgrade: synergy between ceria and cobalt species. *J. Mater. Chem. A* **2021,** *9*, 8541-8553.

24. Cabrera-Afonso, M. J.; Carreño, M. C.; Urbano, A., Site-selective Oxidative Dearomatization of Phenols and Naphthols into *ortho*-Quinols or Epoxy *ortho*-Quinols using Oxone as the Source of Dimethyldioxirane. *Adv. Synth. Catal.* **2019,** *361*, 4468-4473.

25. Uyanik, M.; Sahara, N.; Ishihara, K., Regioselective Oxidative Chlorination of Arenols Using NaCl and Oxone. *Eur. J. Org. Chem.* **2019,** *2019*, 27-31.

26. Augustine, J.; Bombrun, A.; Mandal, A.; Alagarsamy, P.; Atta, R.; Selvam, P., Propylphosphonic Anhydride (T3P®)-Mediated One-Pot Rearrangement of Carboxylic Acids to Carbamates. *Synthesis* **2011,** *2011*, 1477-1483.

^{1}H NMR ($CDCl_3$): 4-Ethylnaphthalen-1-ol (**1b**)

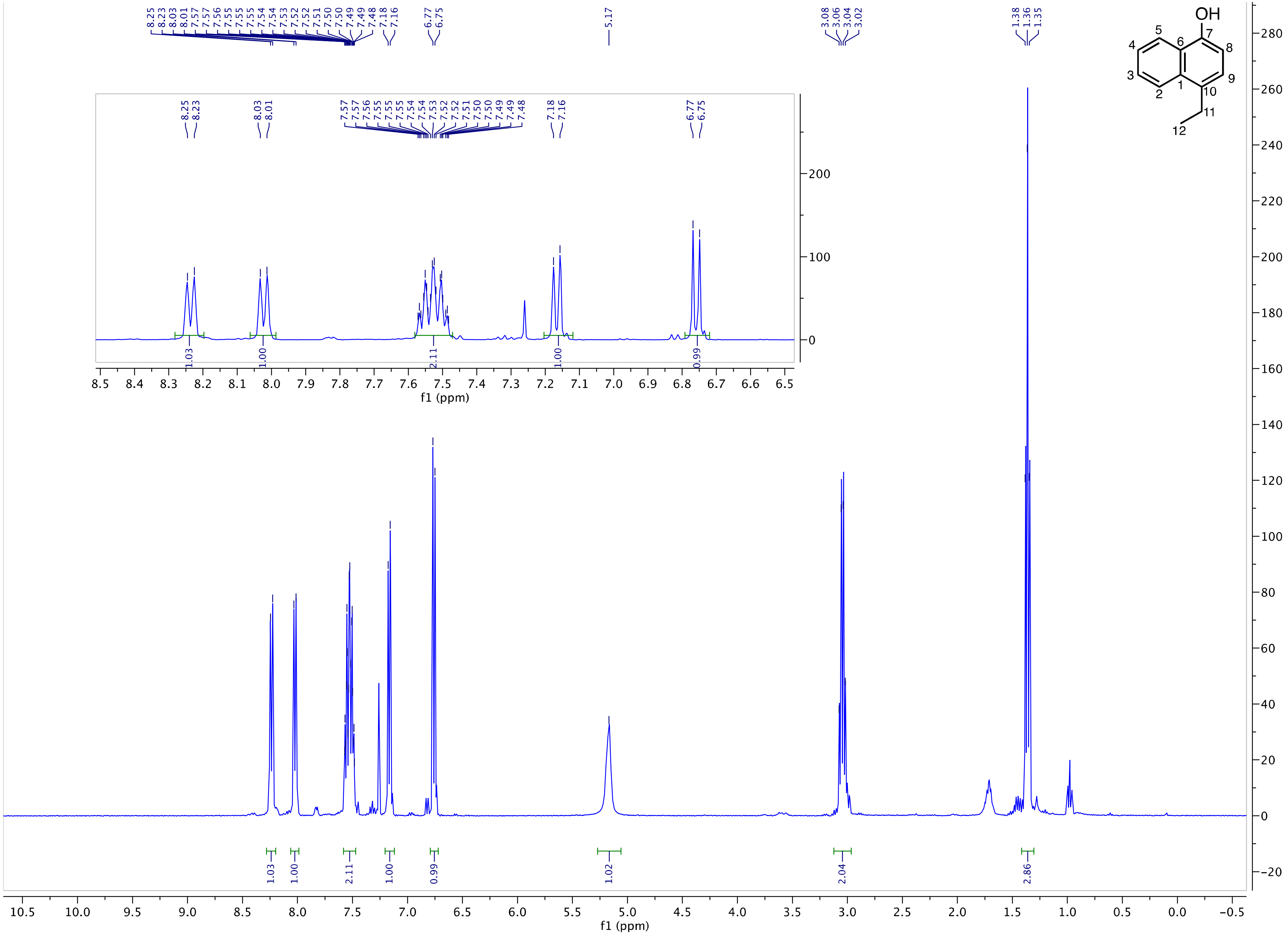

^{13}C NMR ($CDCl_3$): 4-Ethylnaphthalen-1-ol (**1b**)

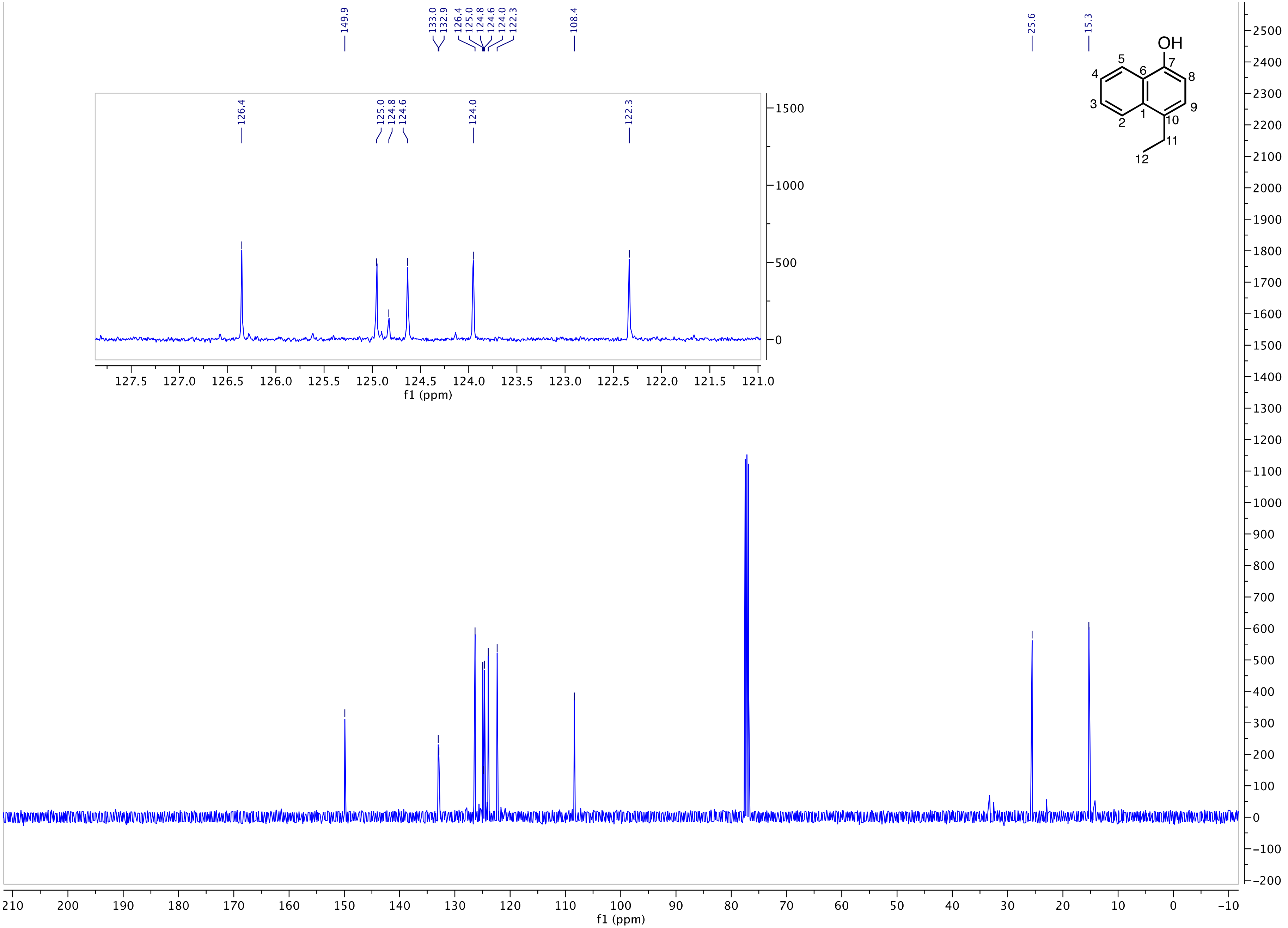

^{1}H NMR ($CDCl_3$): 4-Hexylnaphthalen-1-ol (1c)

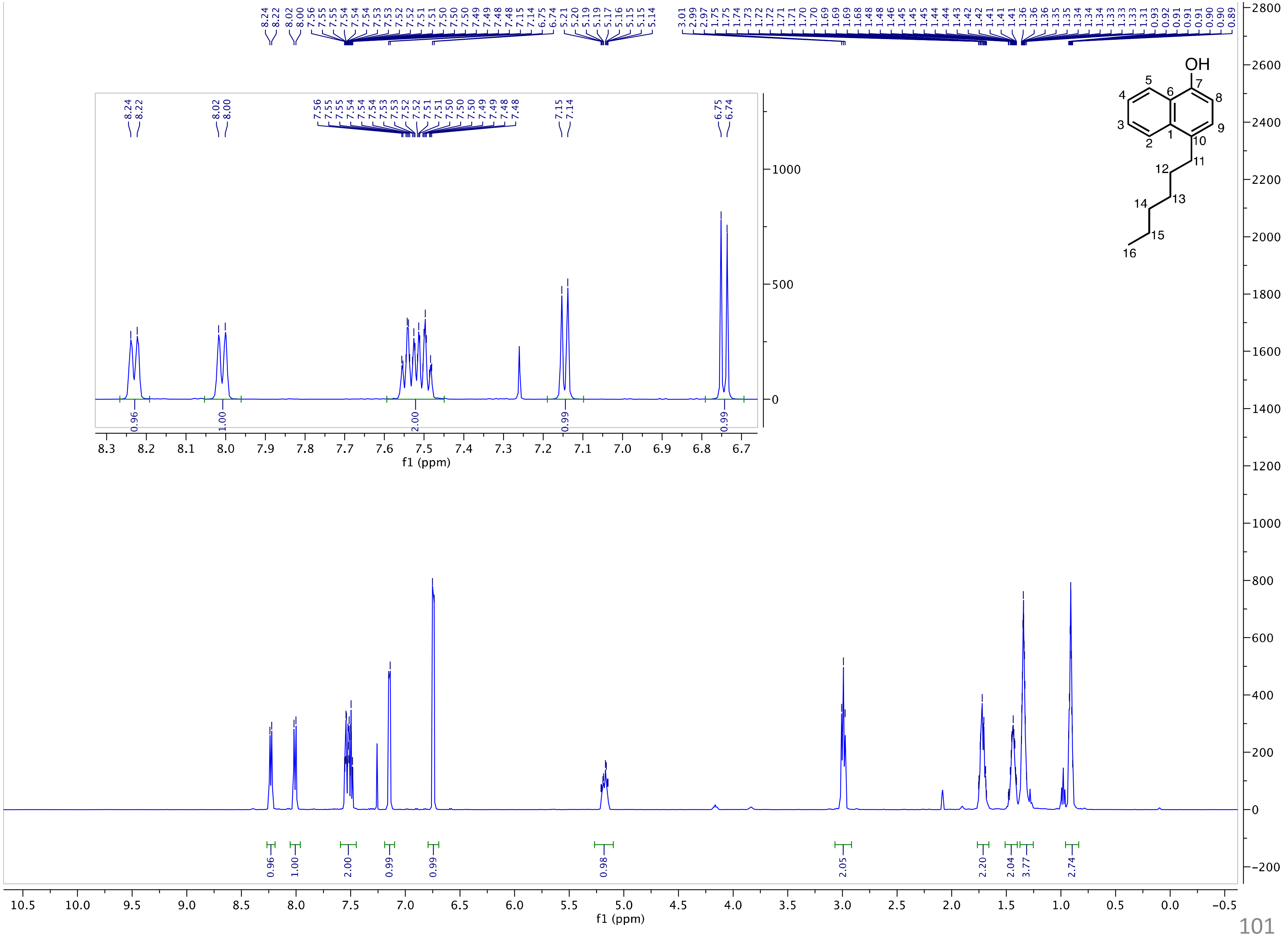

^{13}C NMR ($CDCl_3$): 4-Hexylnaphthalen-1-ol (**1c**)

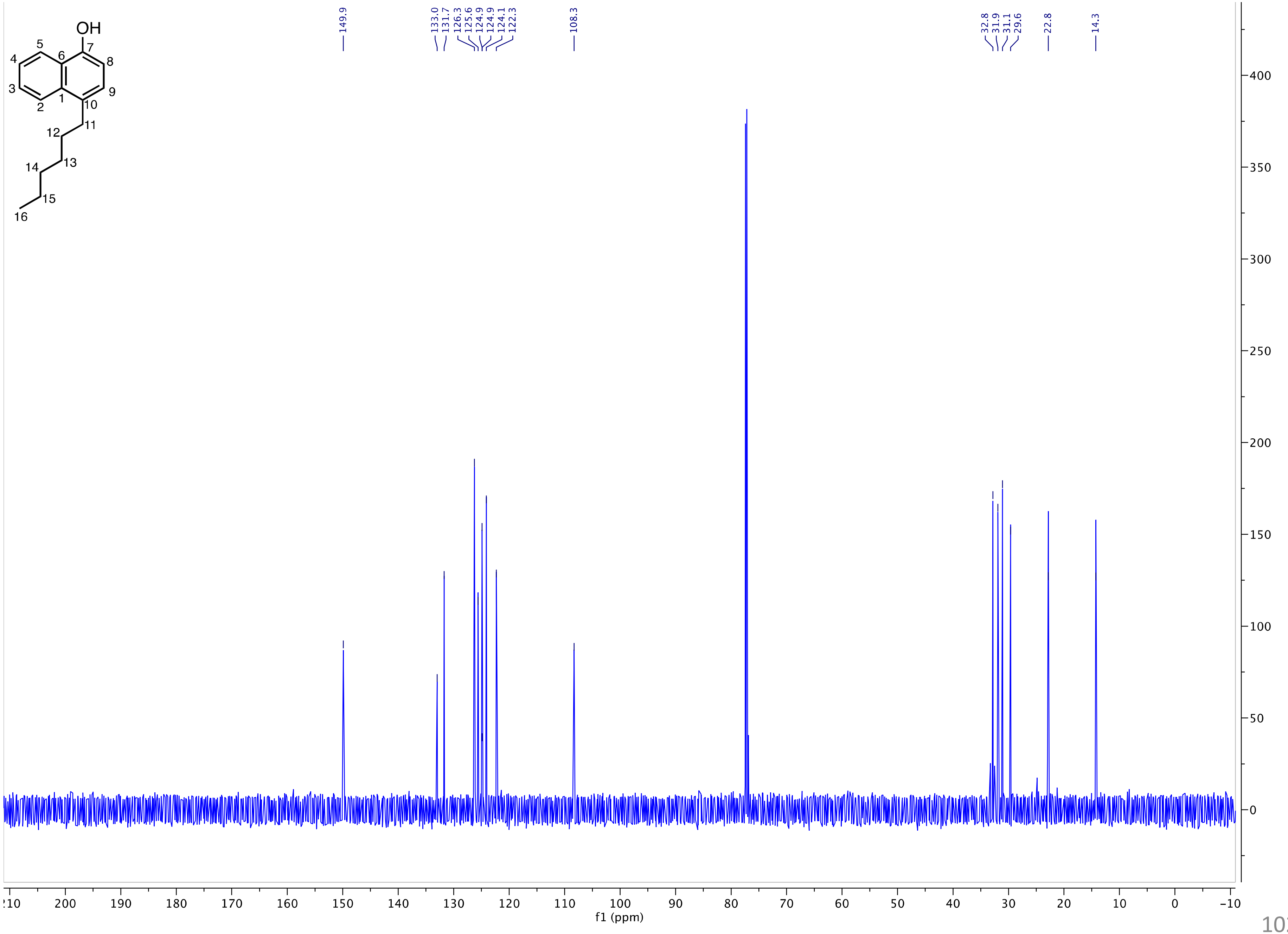

^{1}H NMR ($CDCl_3$): 1-(benzyloxy)-4-Phenethylnaphthalene (S16)

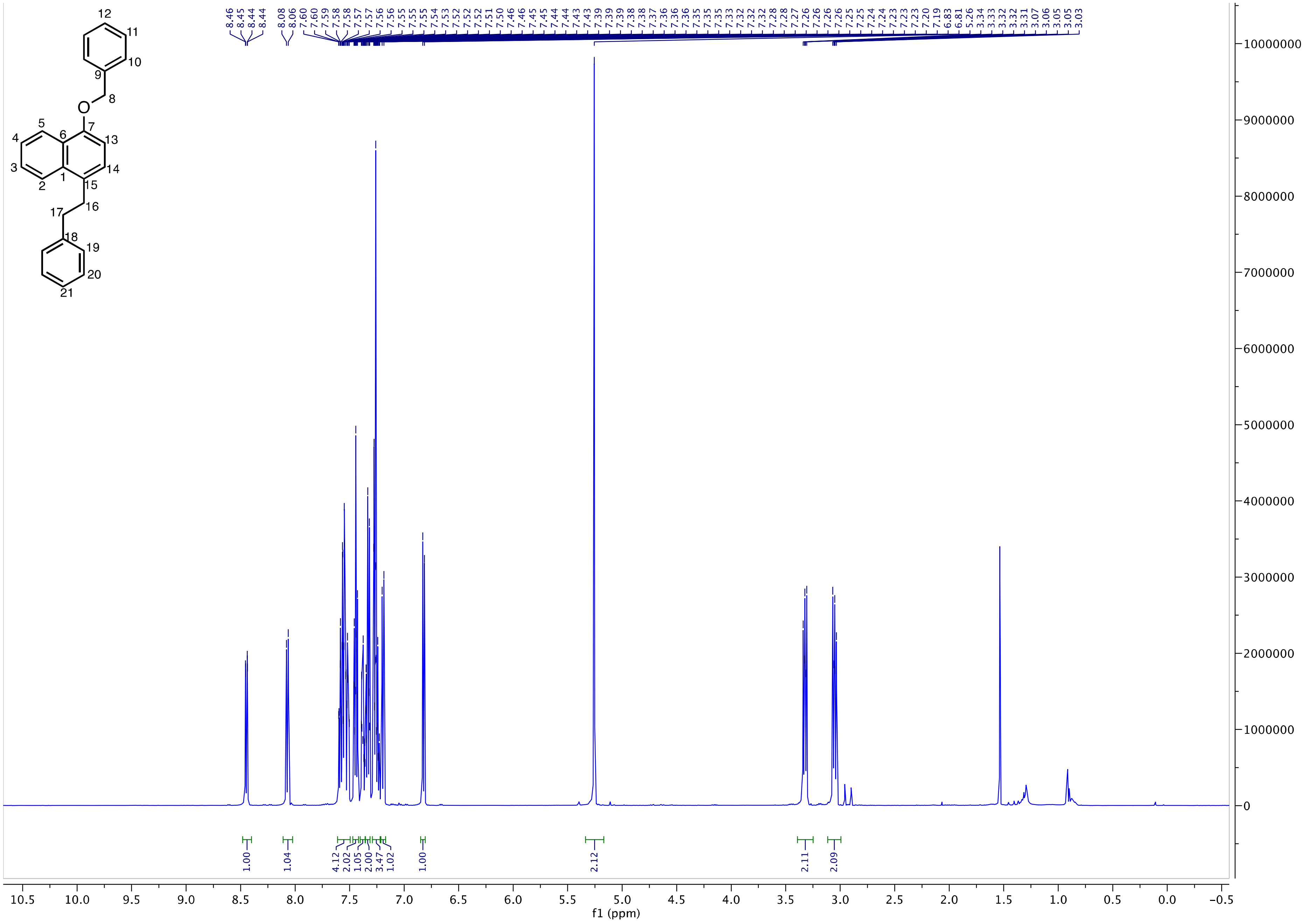

^{13}C NMR ($CDCl_3$): 1-(benzyloxy)-4-Phenethylnaphthalene (S16)

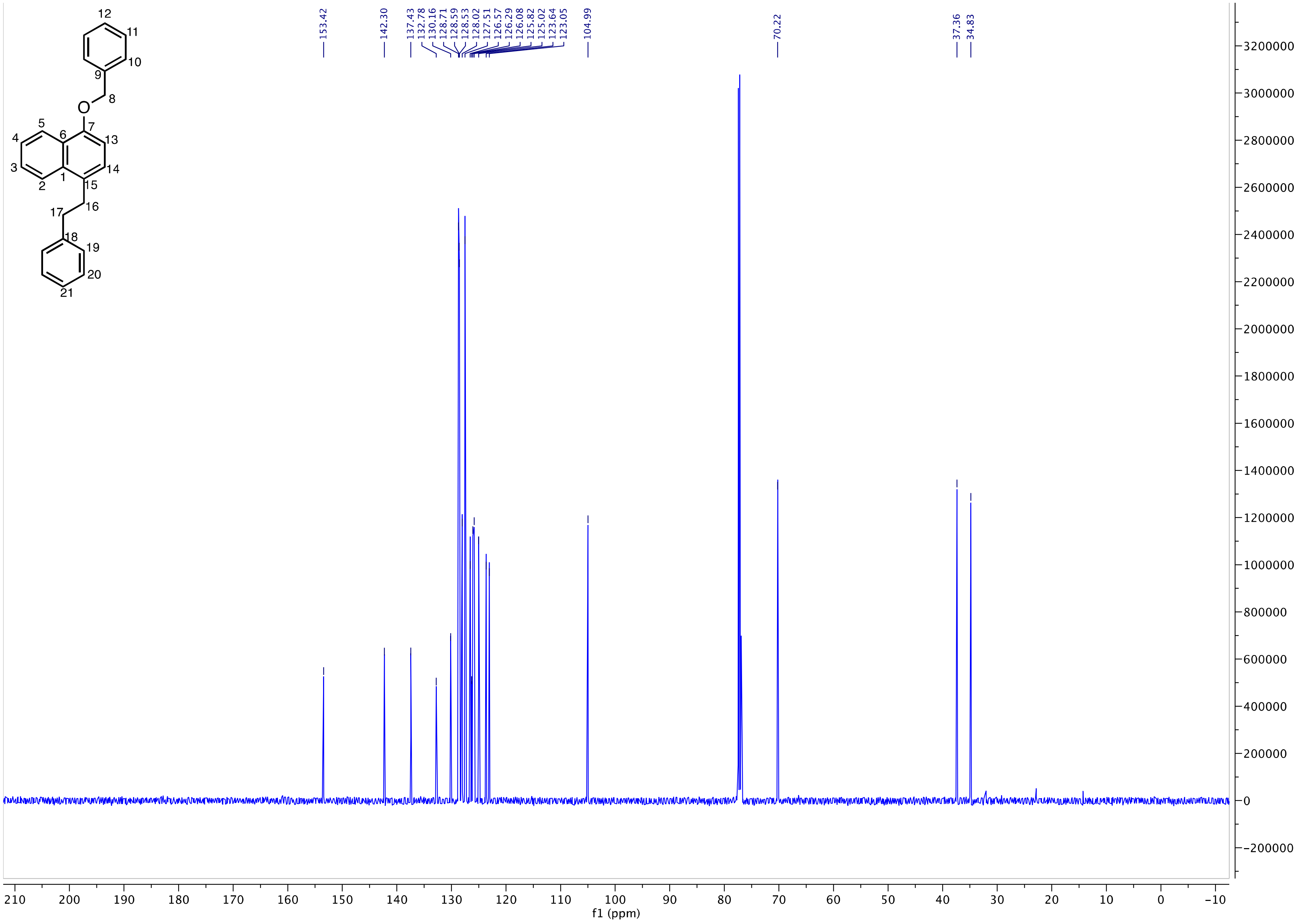

^{1}H NMR (DMSO-d_6): 4-Phenethylnaphthalen-1-ol (1d)

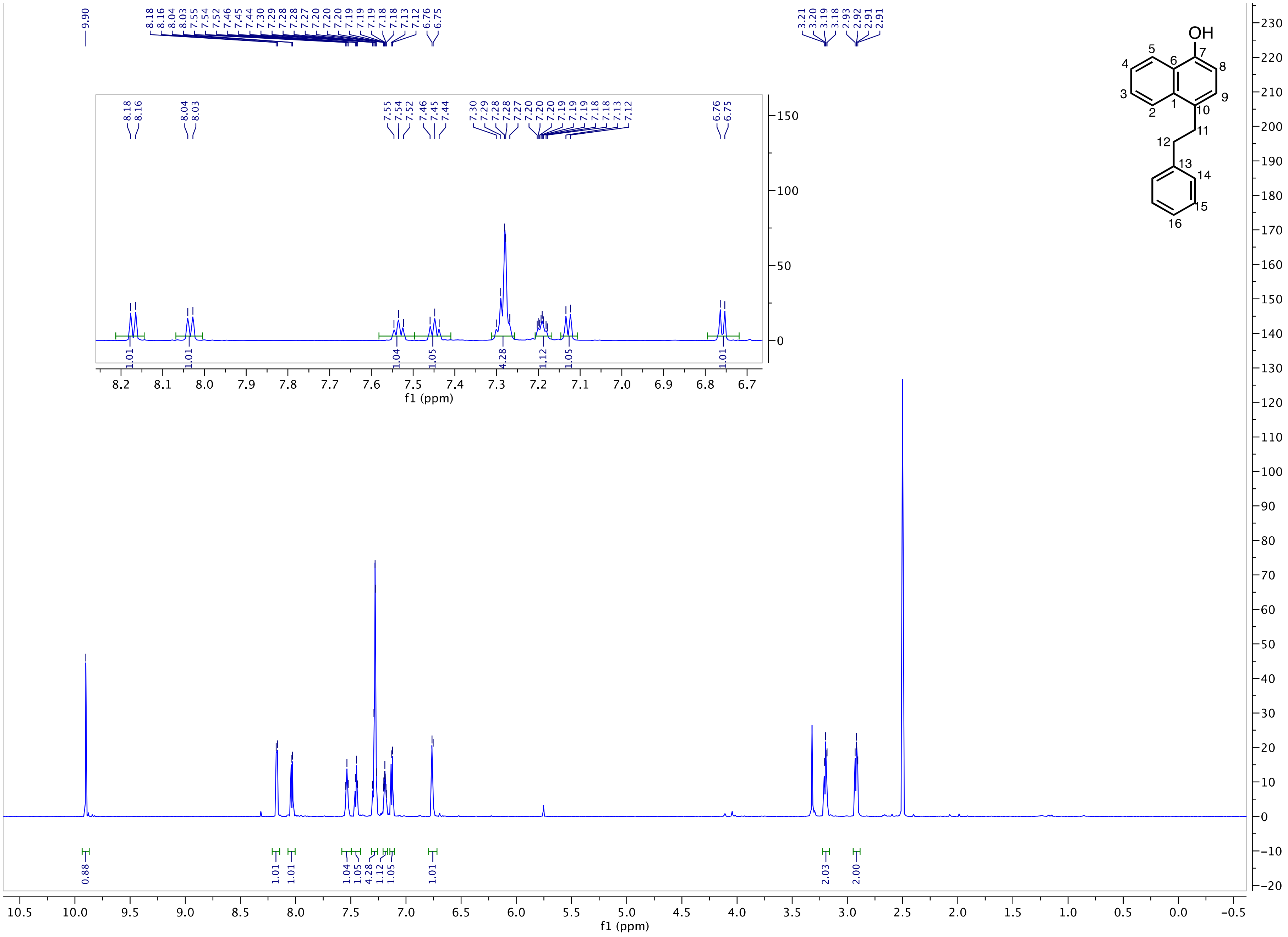

^{13}C NMR ($CDCl_3$): 4-Phenethylnaphthalen-1-ol (**1d**)

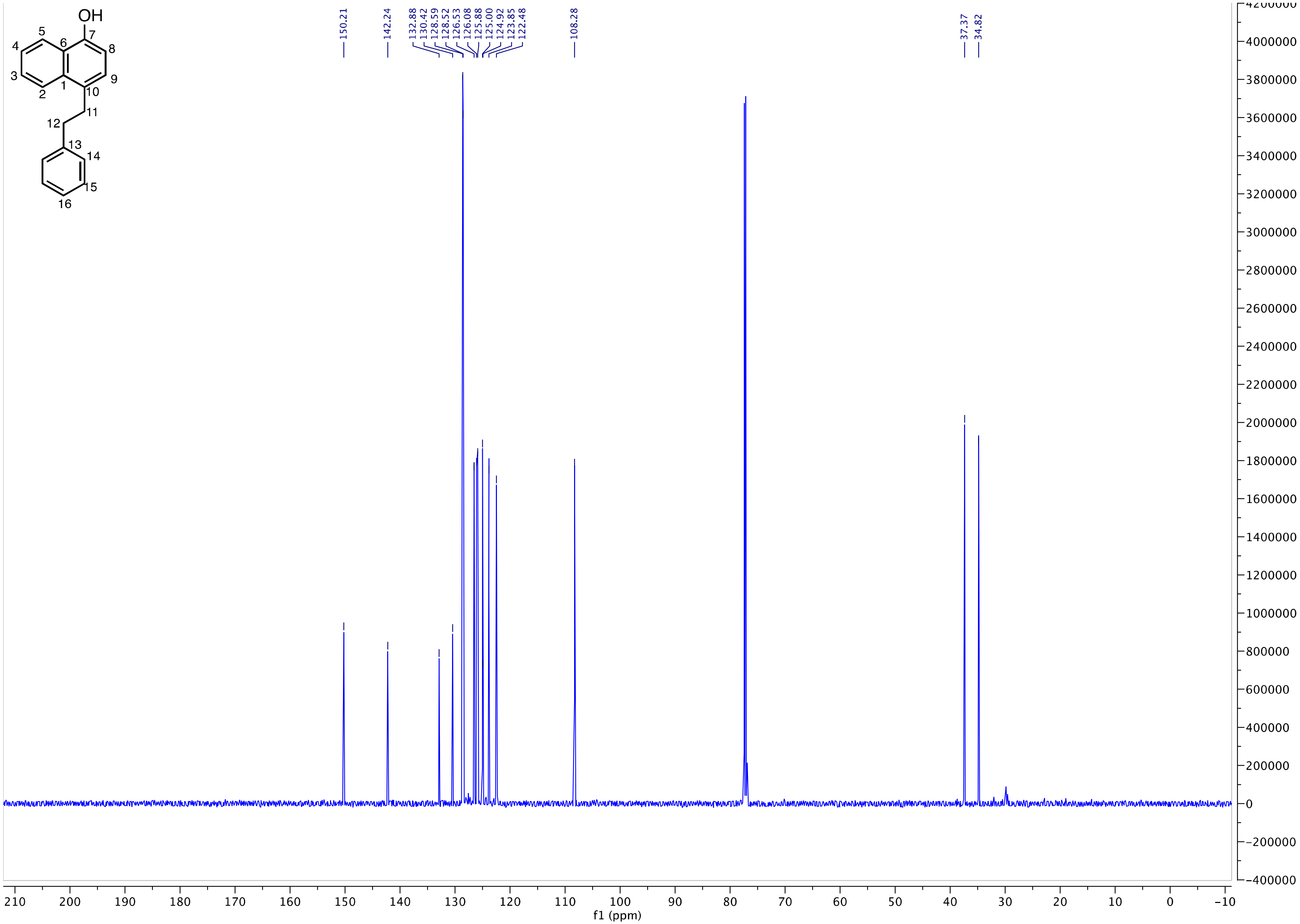

^{1}H NMR ($CDCl_3$): 1,2-Dihydroacenaphthylen-5-ol (1e)

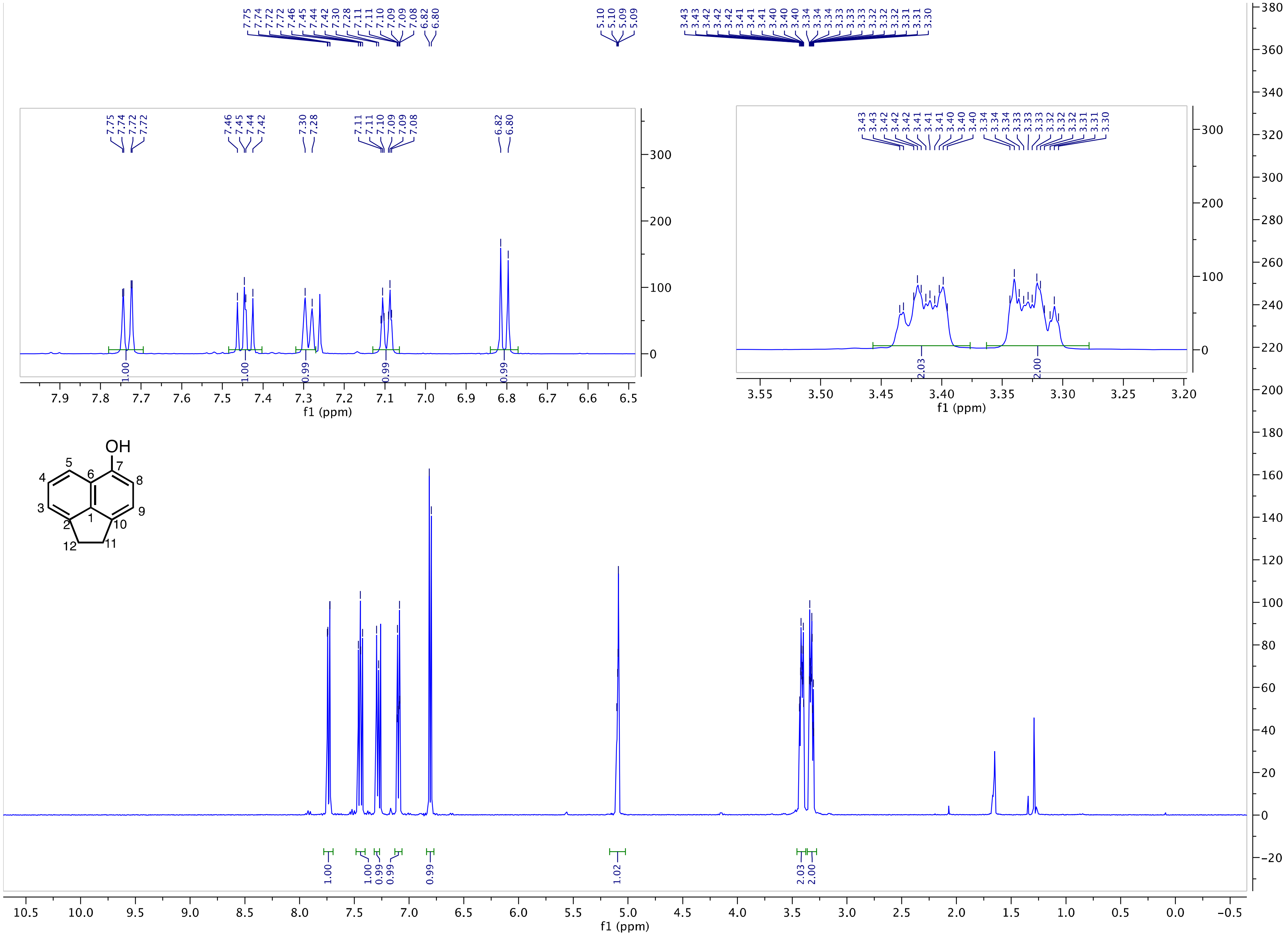

^{13}C NMR ($CDCl_3$): 1,2-Dihydroacenaphthylen-5-ol (**1e**)

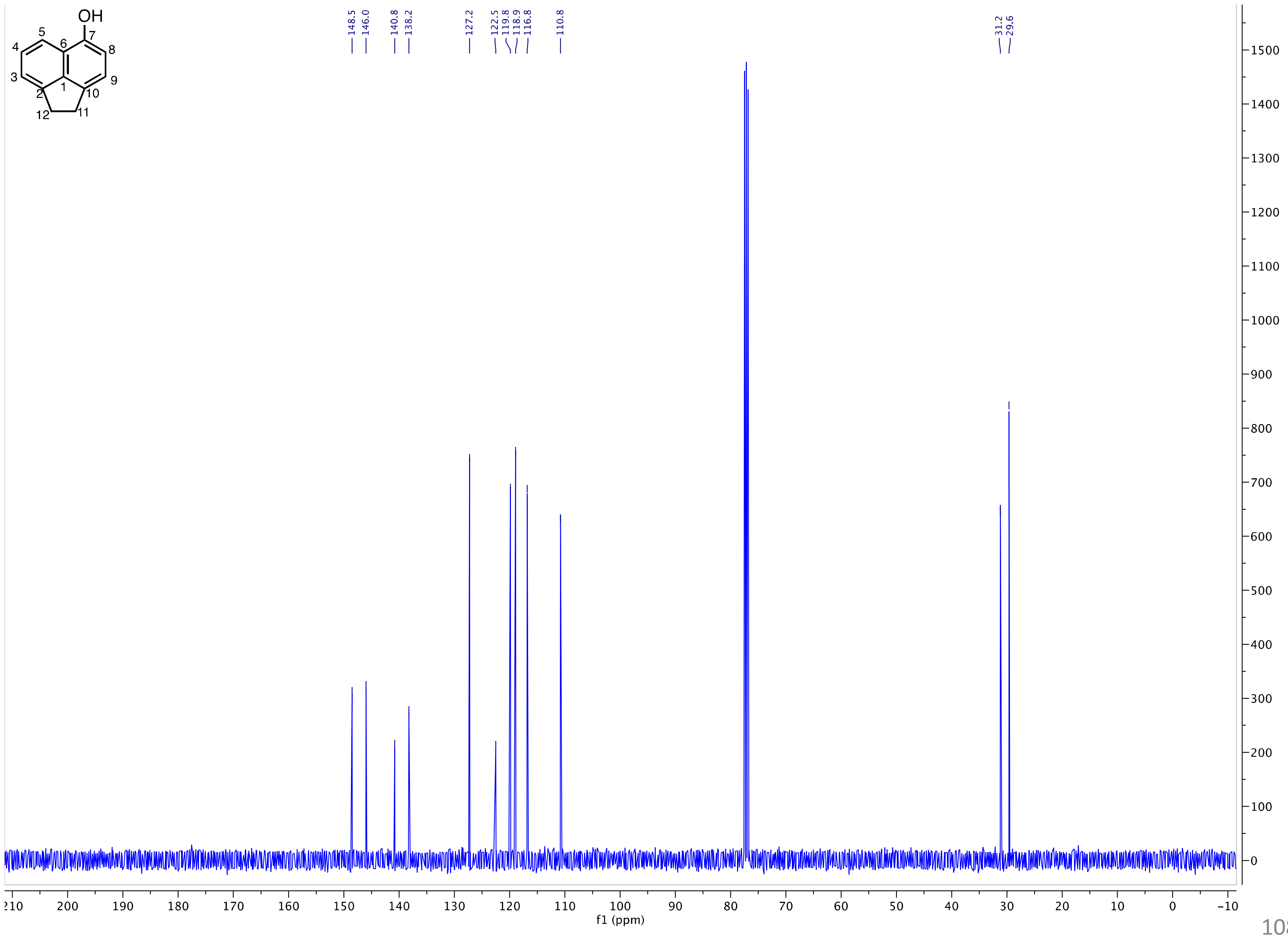

^{1}H NMR ($CDCl_3$): 6-Methoxy-4-methylnaphthalen-1-ol (**1f**)

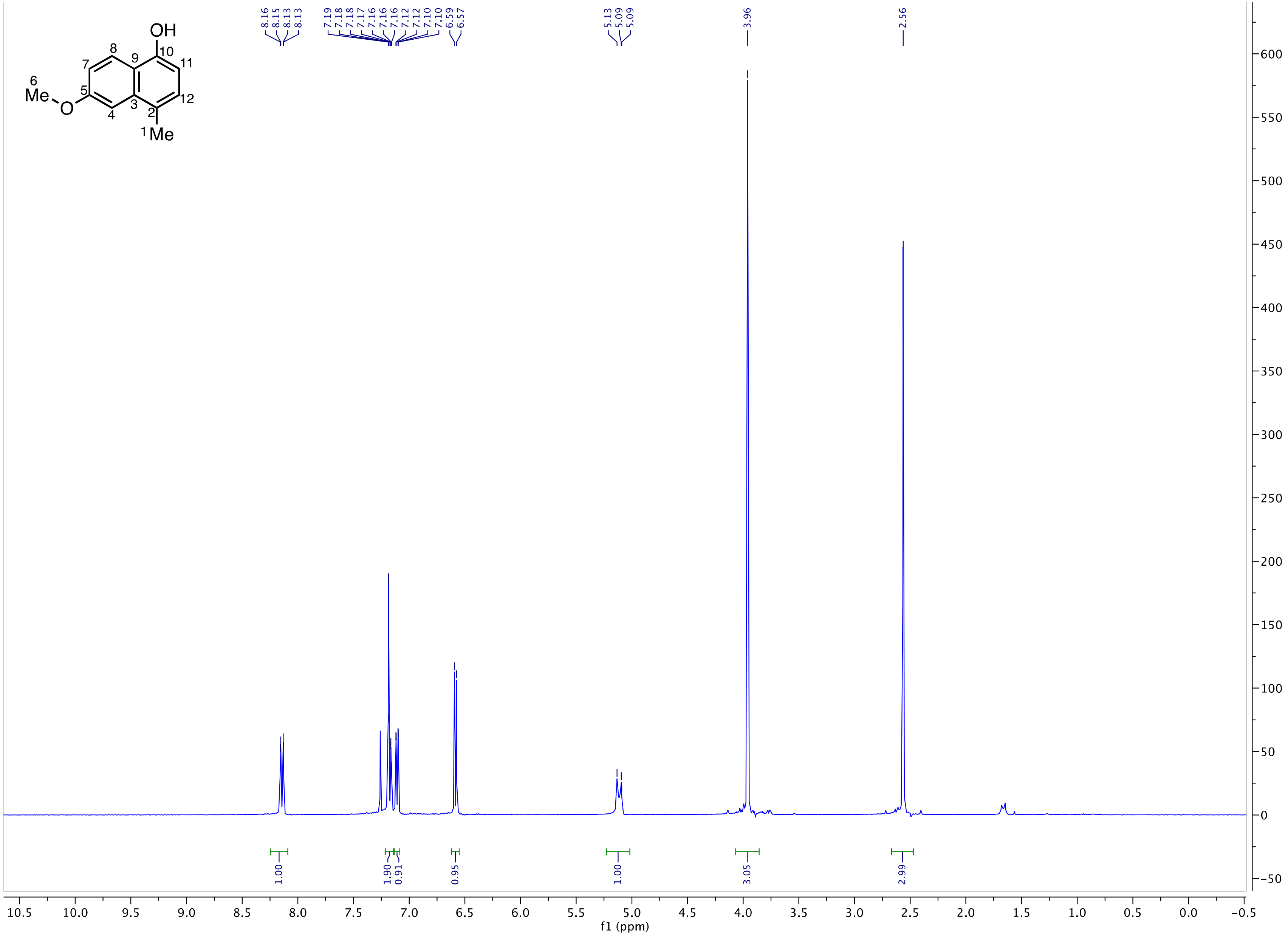

^{13}C **NMR** (CDCl$_3$): 6-Methoxy-4-methylnaphthalen-1-ol (**1f**)

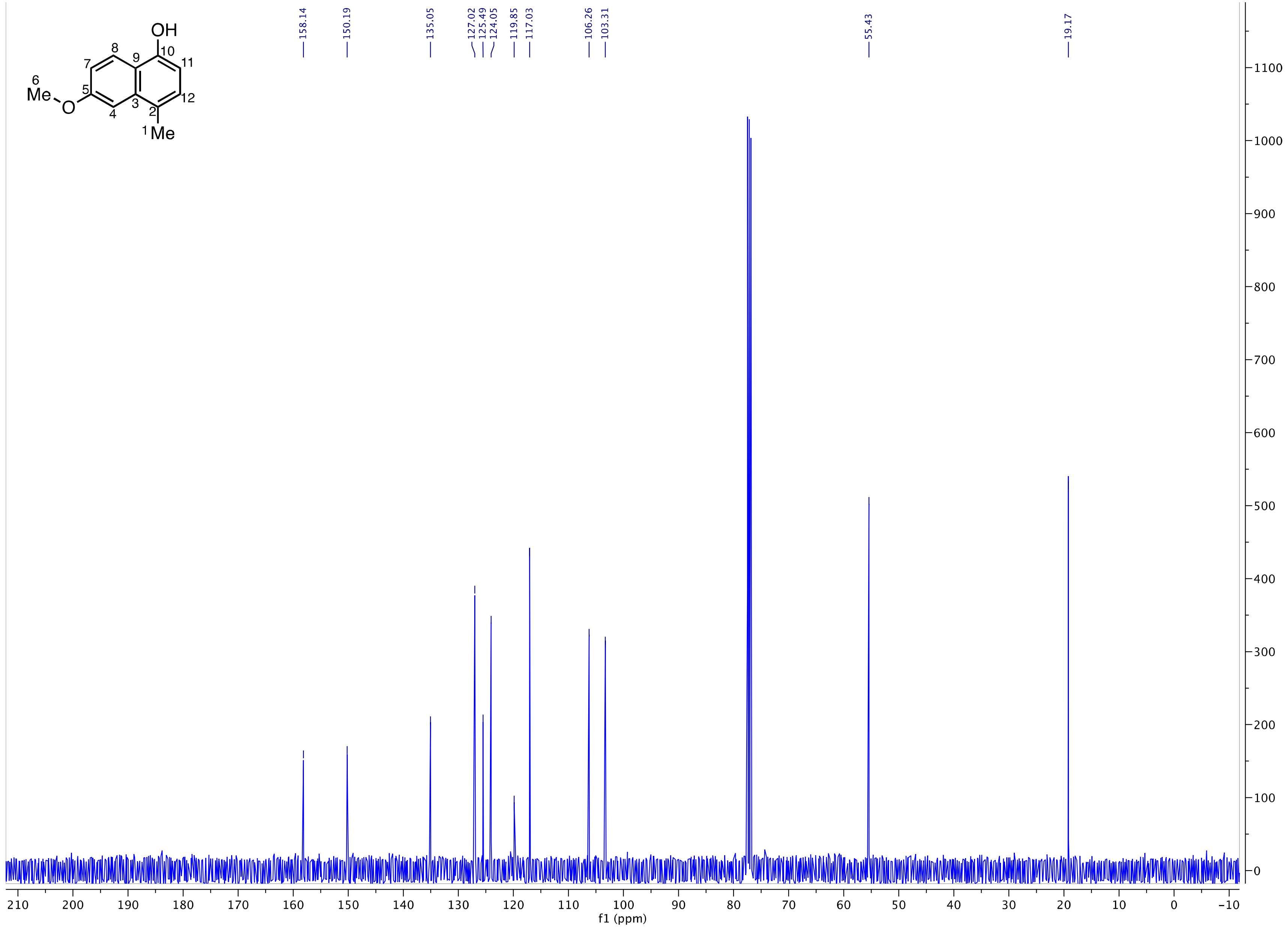

^{1}H NMR ($CDCl_3$): 7-Methoxy-4-methylnaphthalen-1-ol (1g)

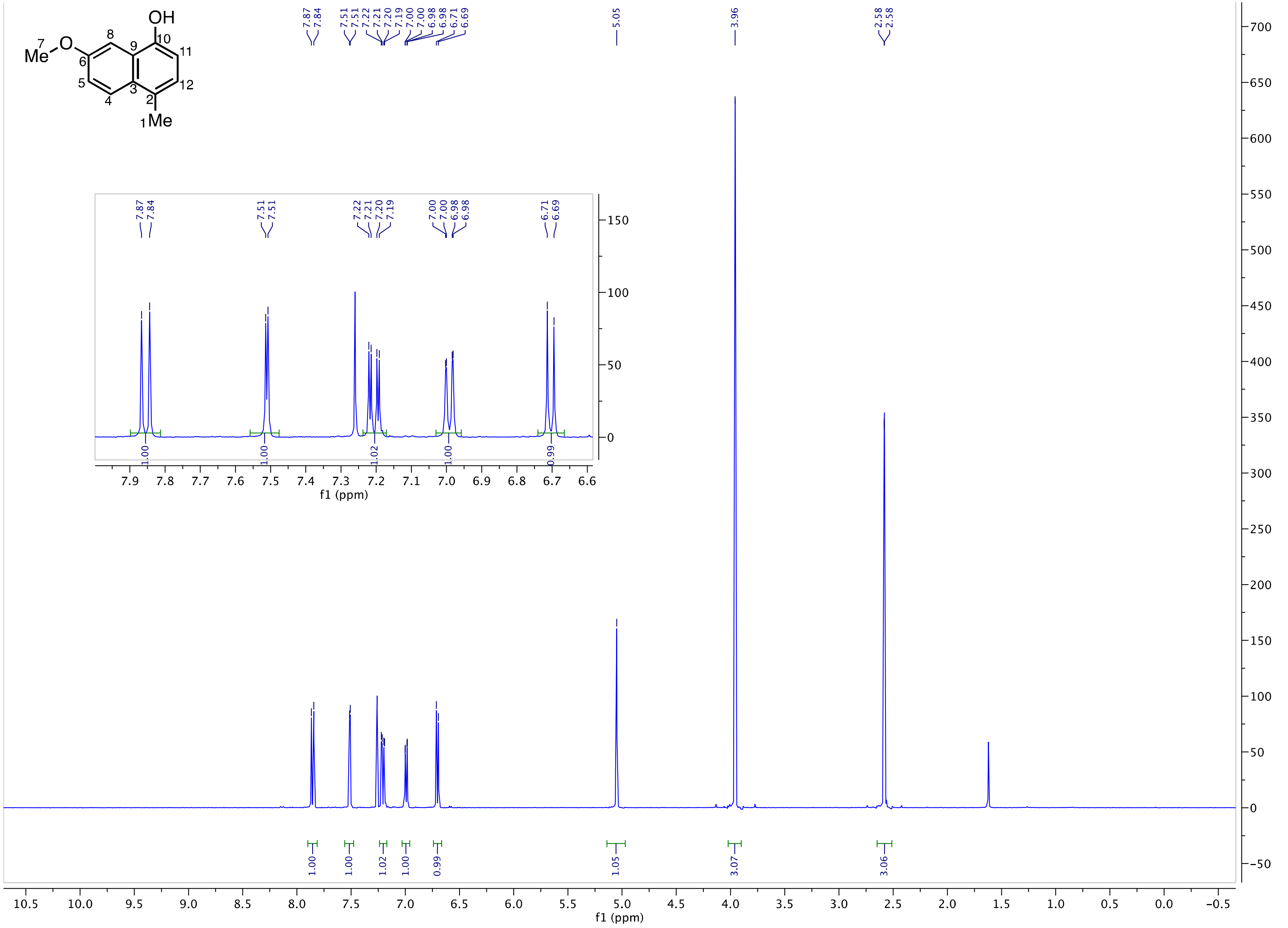

^{13}C NMR ($CDCl_3$): 7-Methoxy-4-methylnaphthalen-1-ol (**1g**)

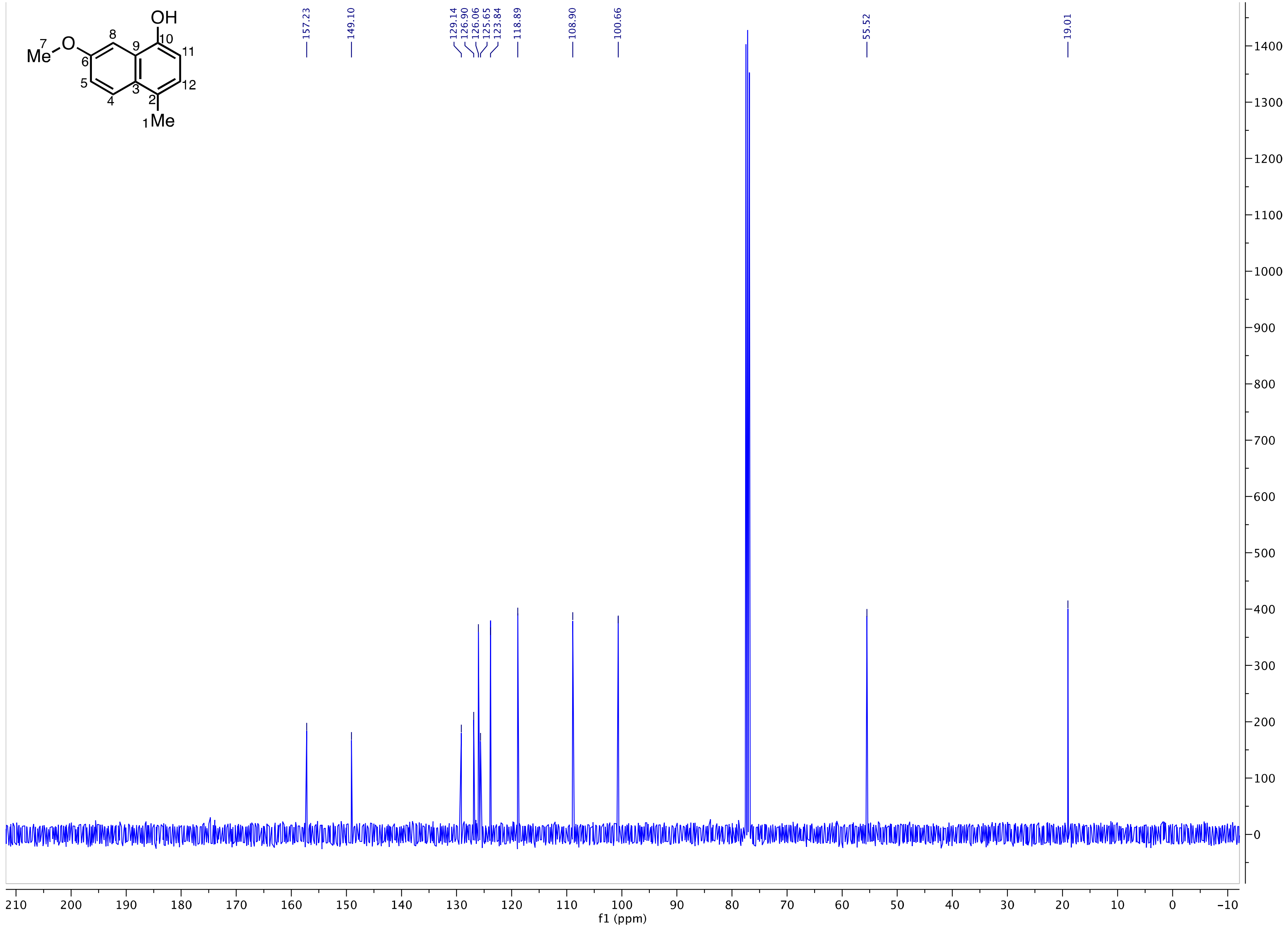

^{1}H NMR ($CDCl_3$): 1,2-Dimethyl-1,4-dihydro-1,4-epoxynaphthalene (S17)

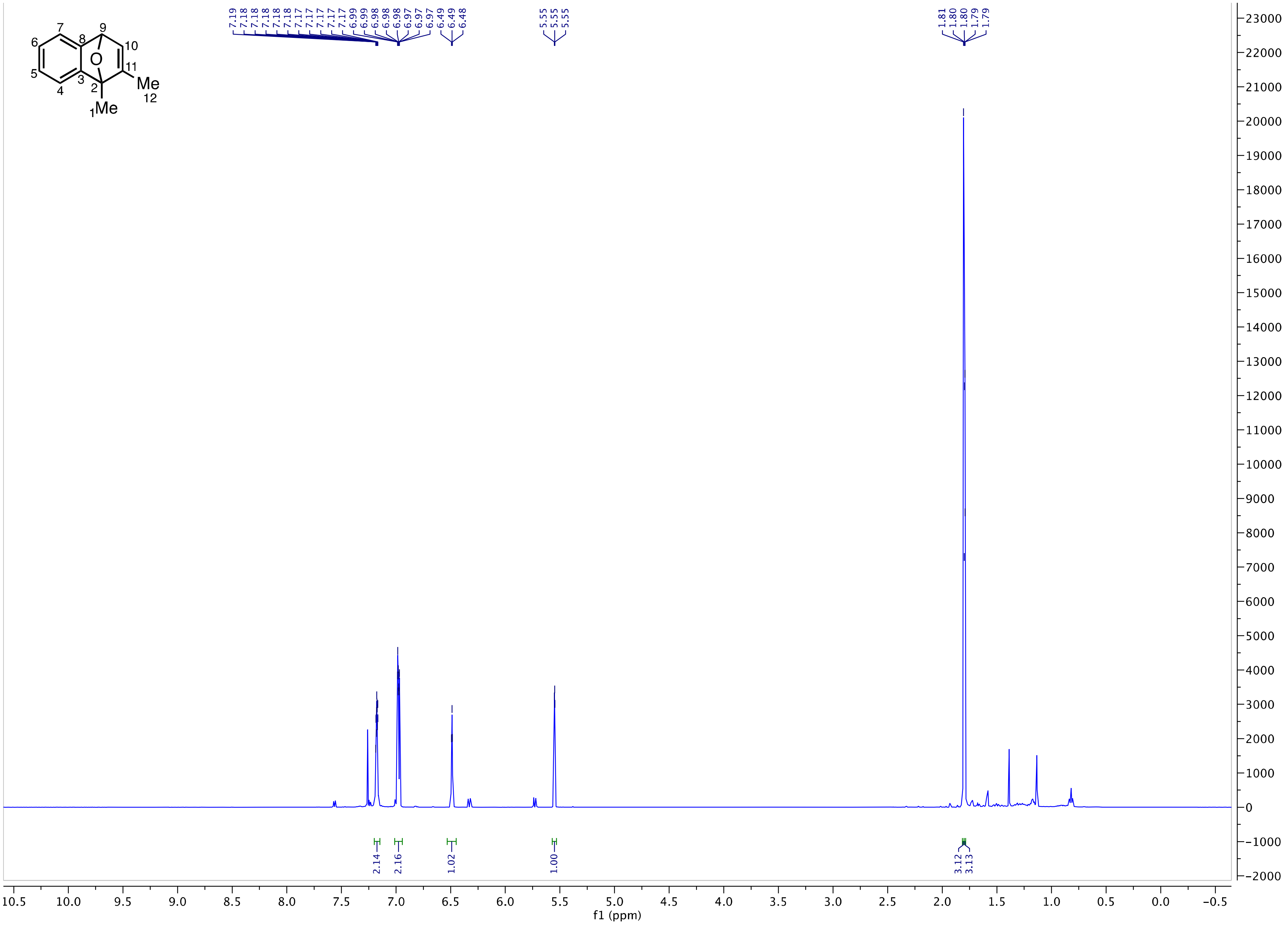

^{13}C NMR ($CDCl_3$): 1,2-Dimethyl-1,4-dihydro-1,4-epoxynaphthalene (S17)

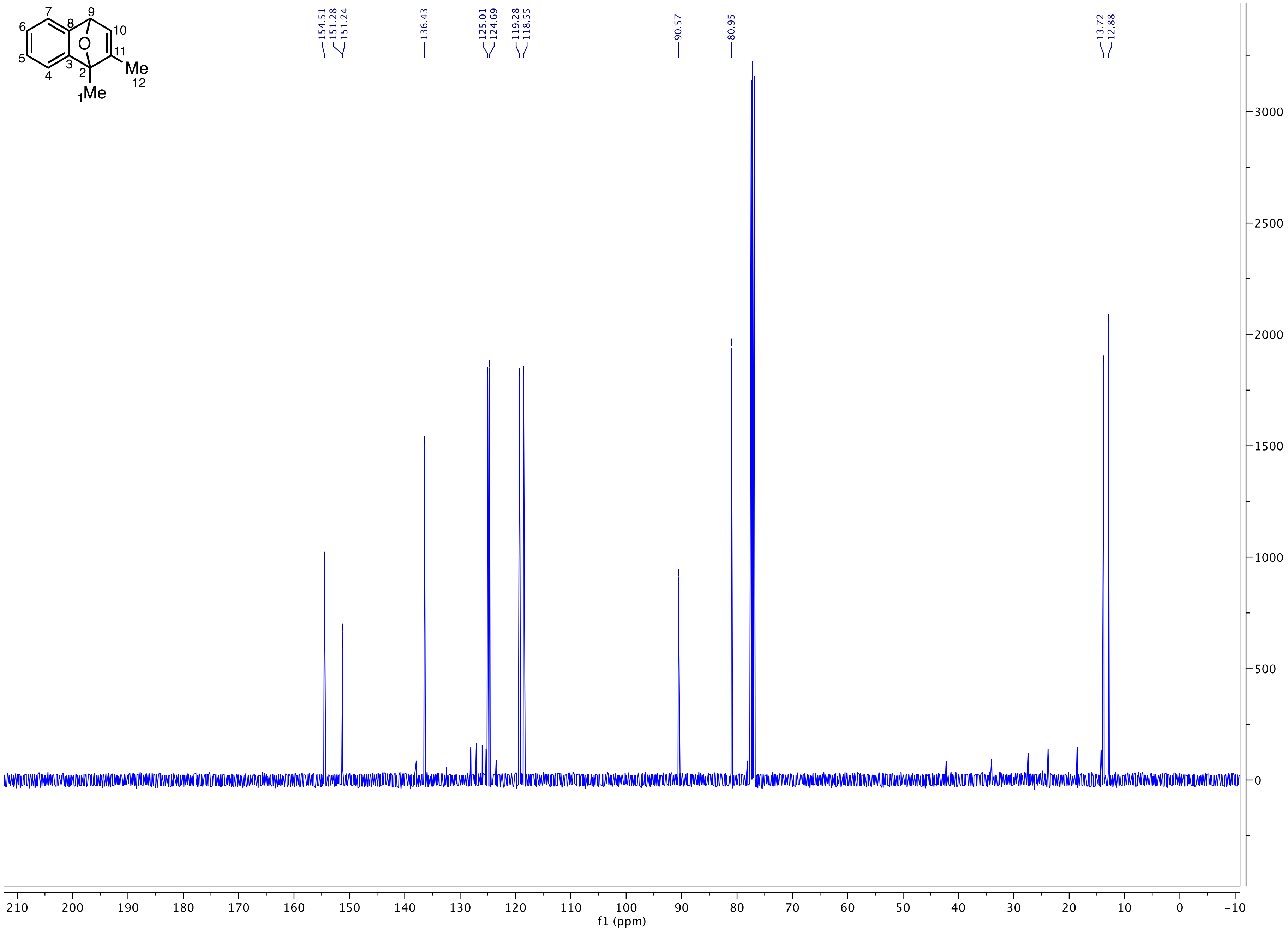

^{1}H NMR ($CDCl_3$): 3,4-Dimethylnaphthalen-1-ol (1i)

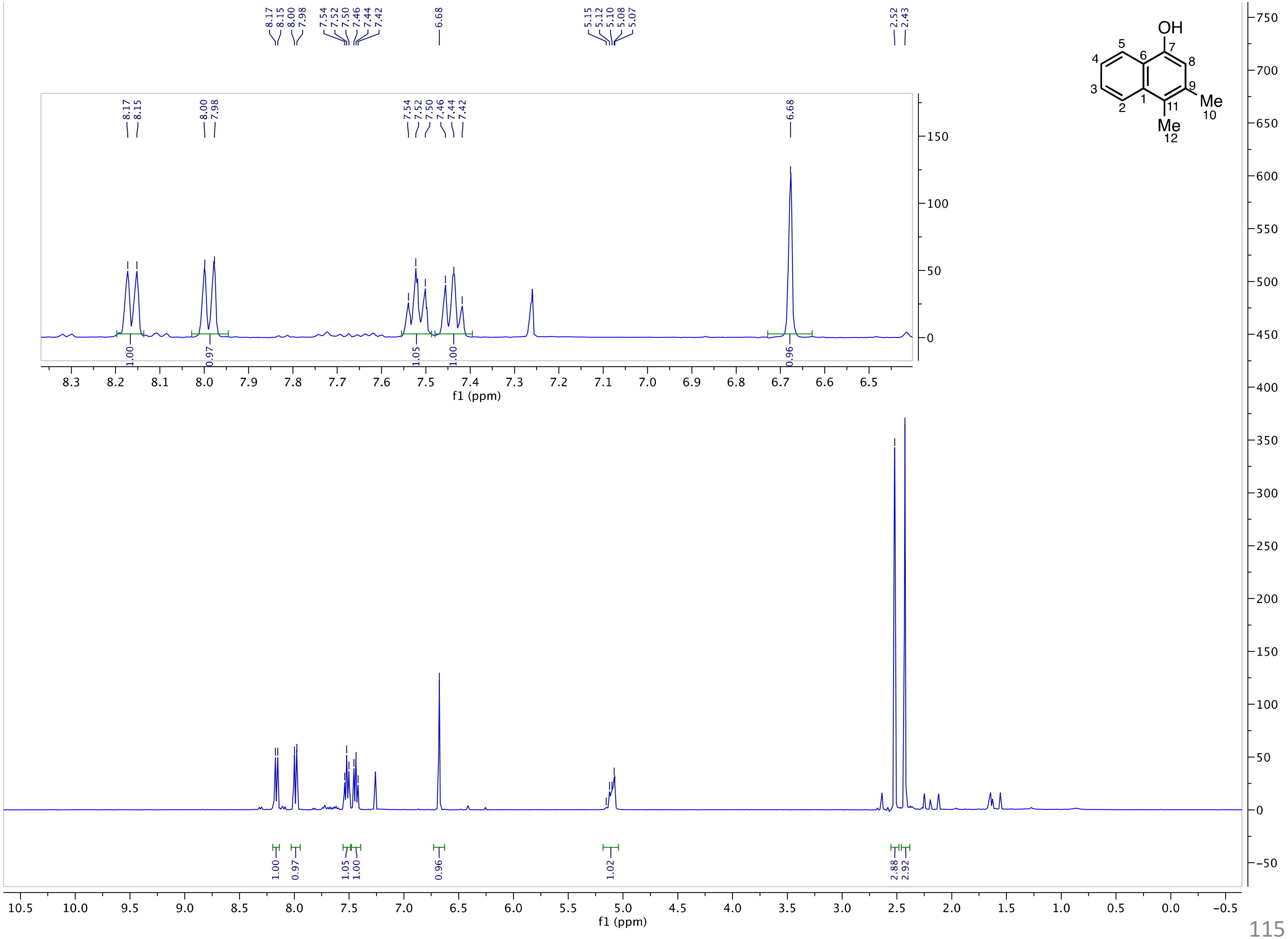

^{13}C NMR ($CDCl_3$): 3,4-Dimethylnaphthalen-1-ol (1i)

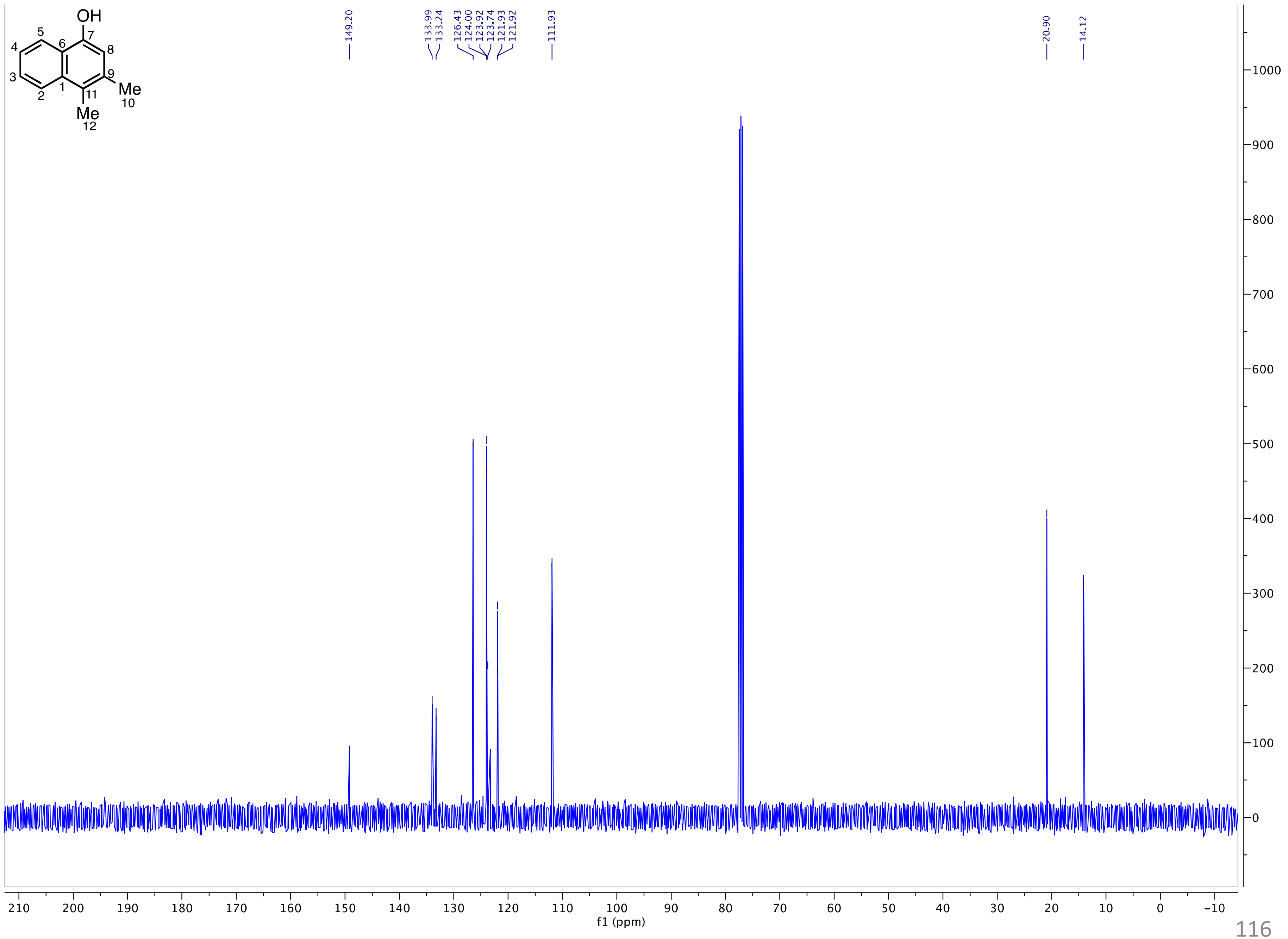

^{1}H NMR ($CDCl_3$): 4-Bromo-1,2-bis(methoxymethoxy)benzene (S18)

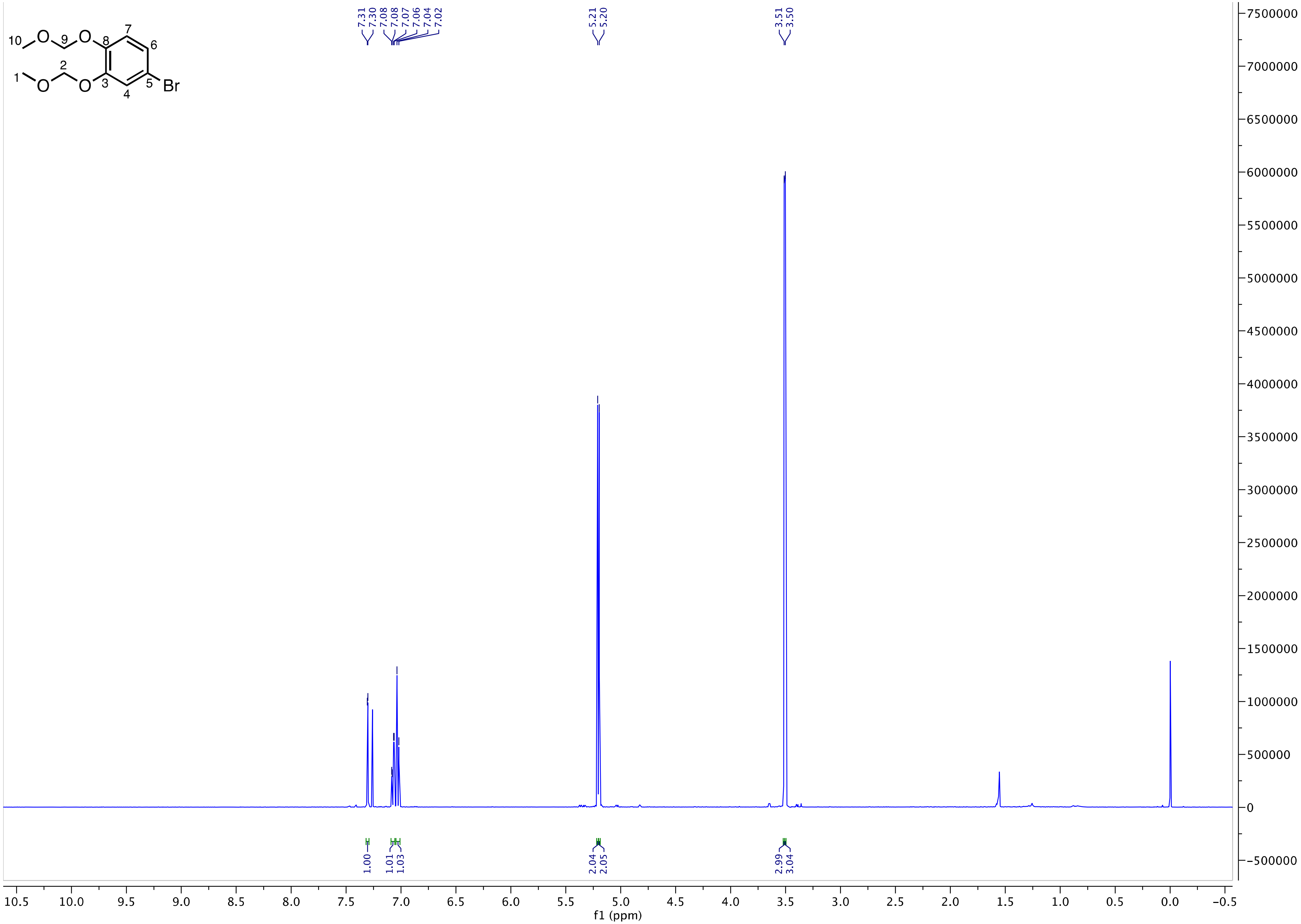

^{13}C NMR ($CDCl_3$): 4-Bromo-1,2-bis(methoxymethoxy)benzene (**S18**)

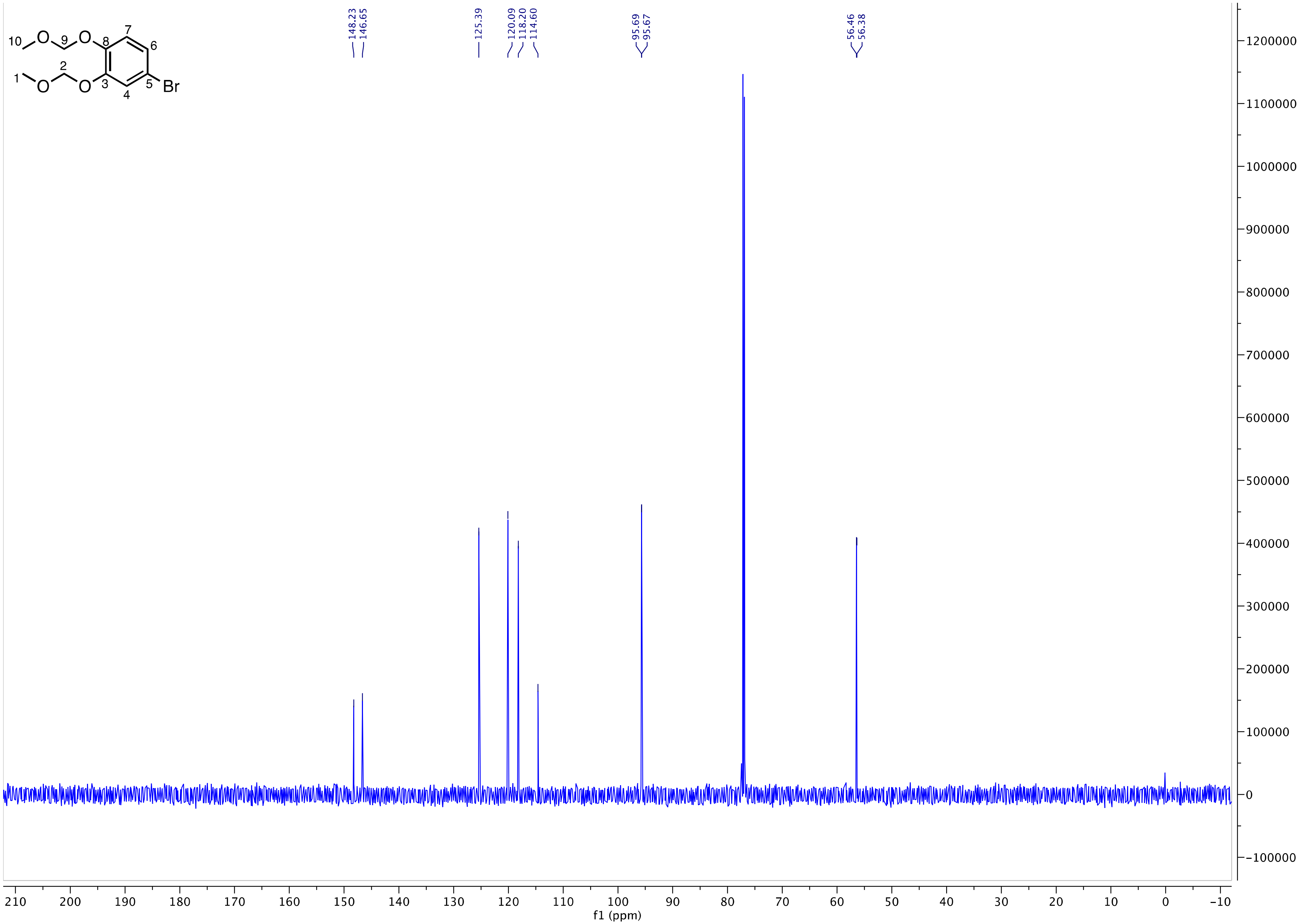

^{1}H NMR (CDCl$_3$): (*R*)-4-Methyl-4-phenylnaphthalen-1(4*H*)-one (**2a**)

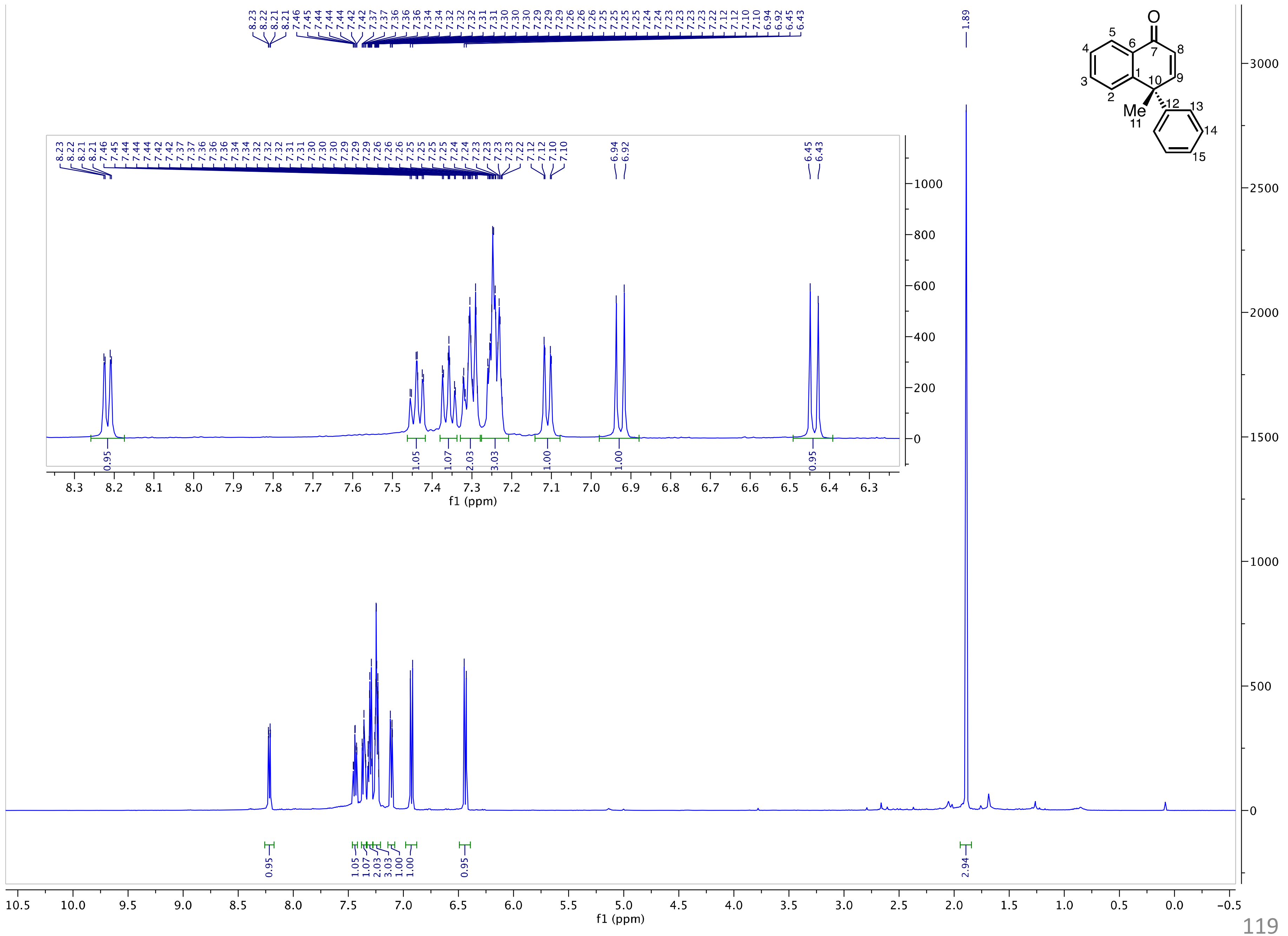

^{13}C NMR (CDCl$_3$): (*R*)-4-Methyl-4-phenylnaphthalen-1(4*H*)-one (**2a**)

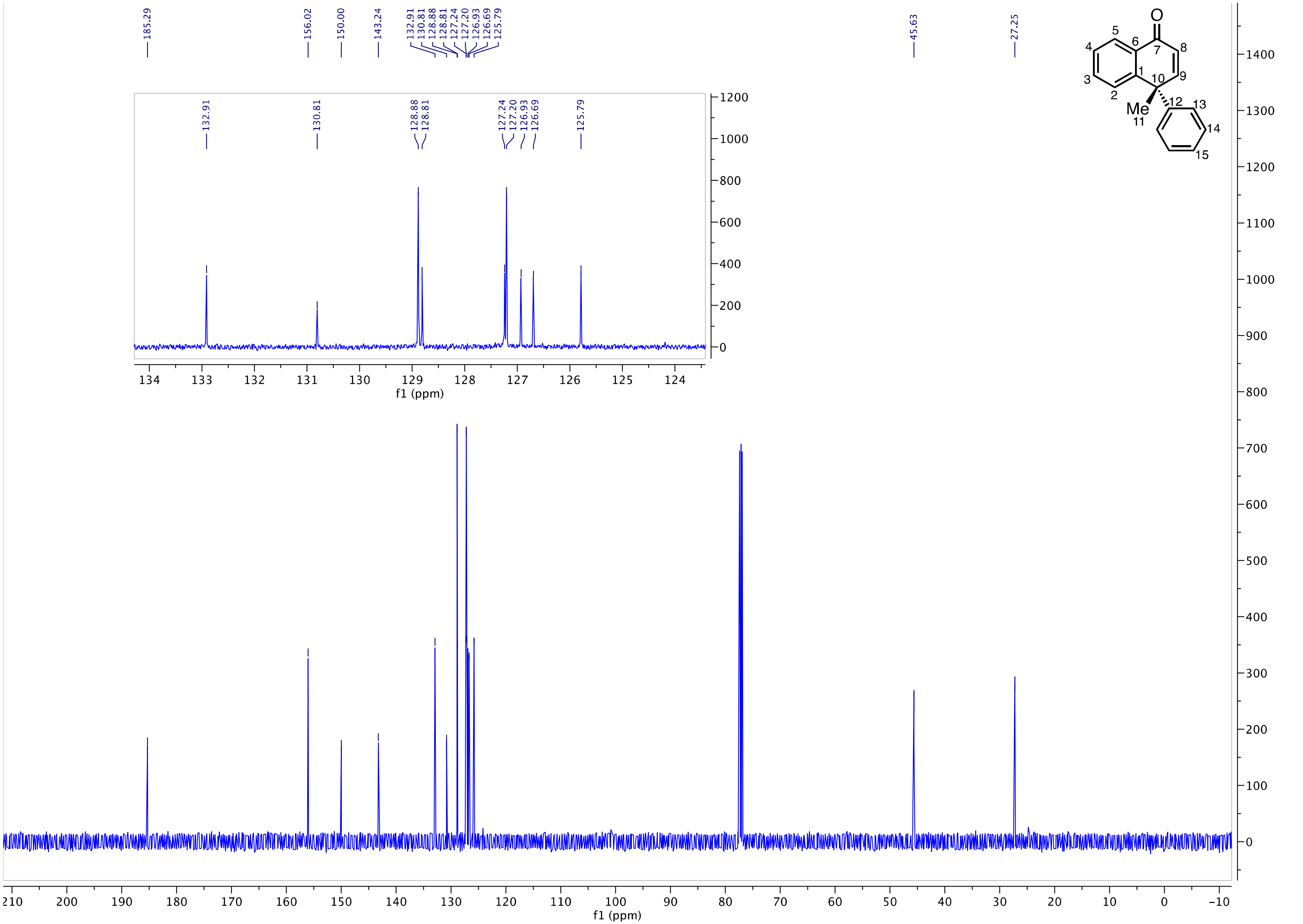

^{1}H NMR ($CDCl_3$): (*R*)-4-Ethyl-4-phenylnaphthalen-1(4*H*)-one (**2b**)

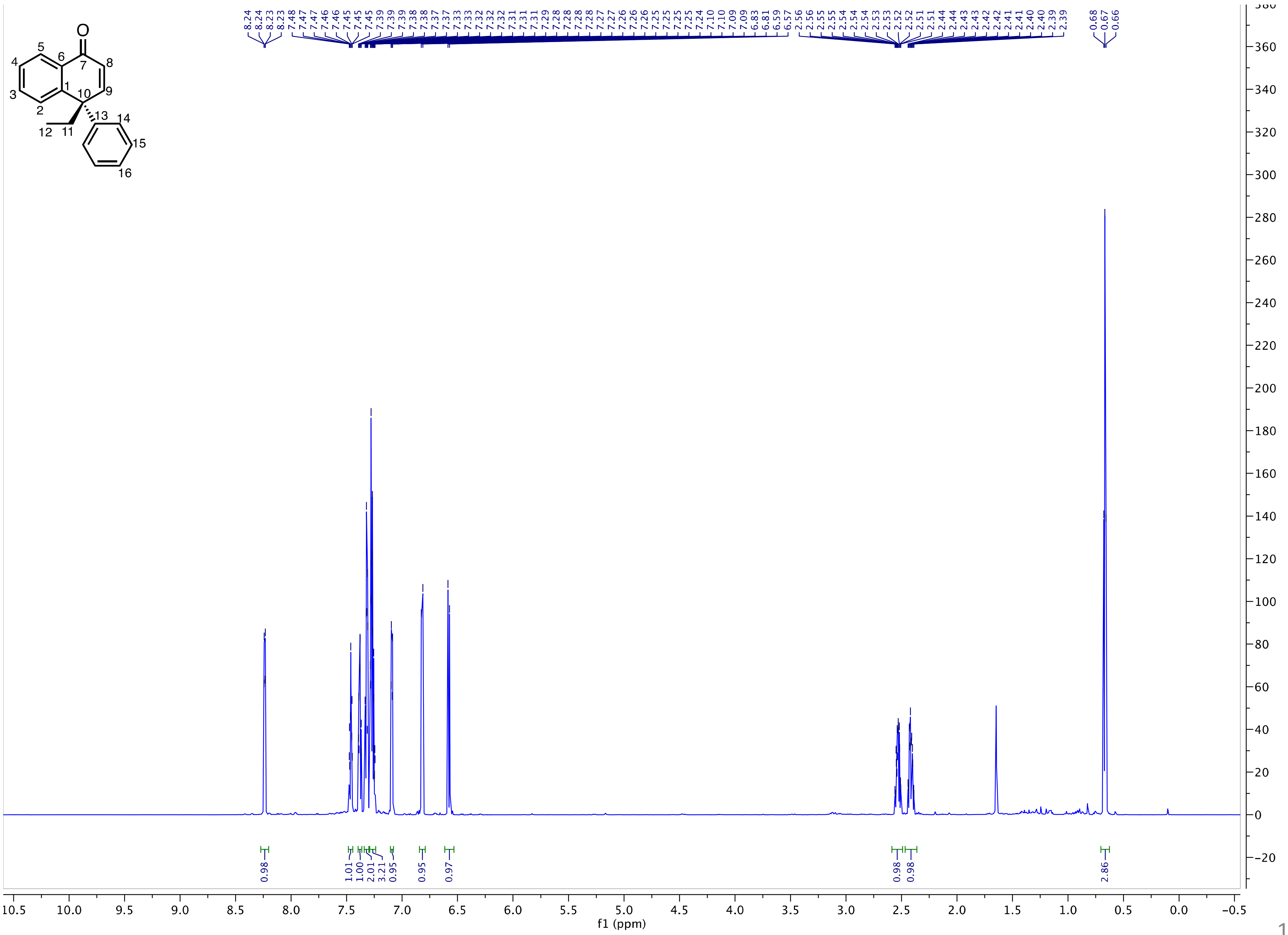

^{13}C NMR (CDCl$_3$): (*R*)-4-Ethyl-4-phenylnaphthalen-1(4*H*)-one (**2b**)

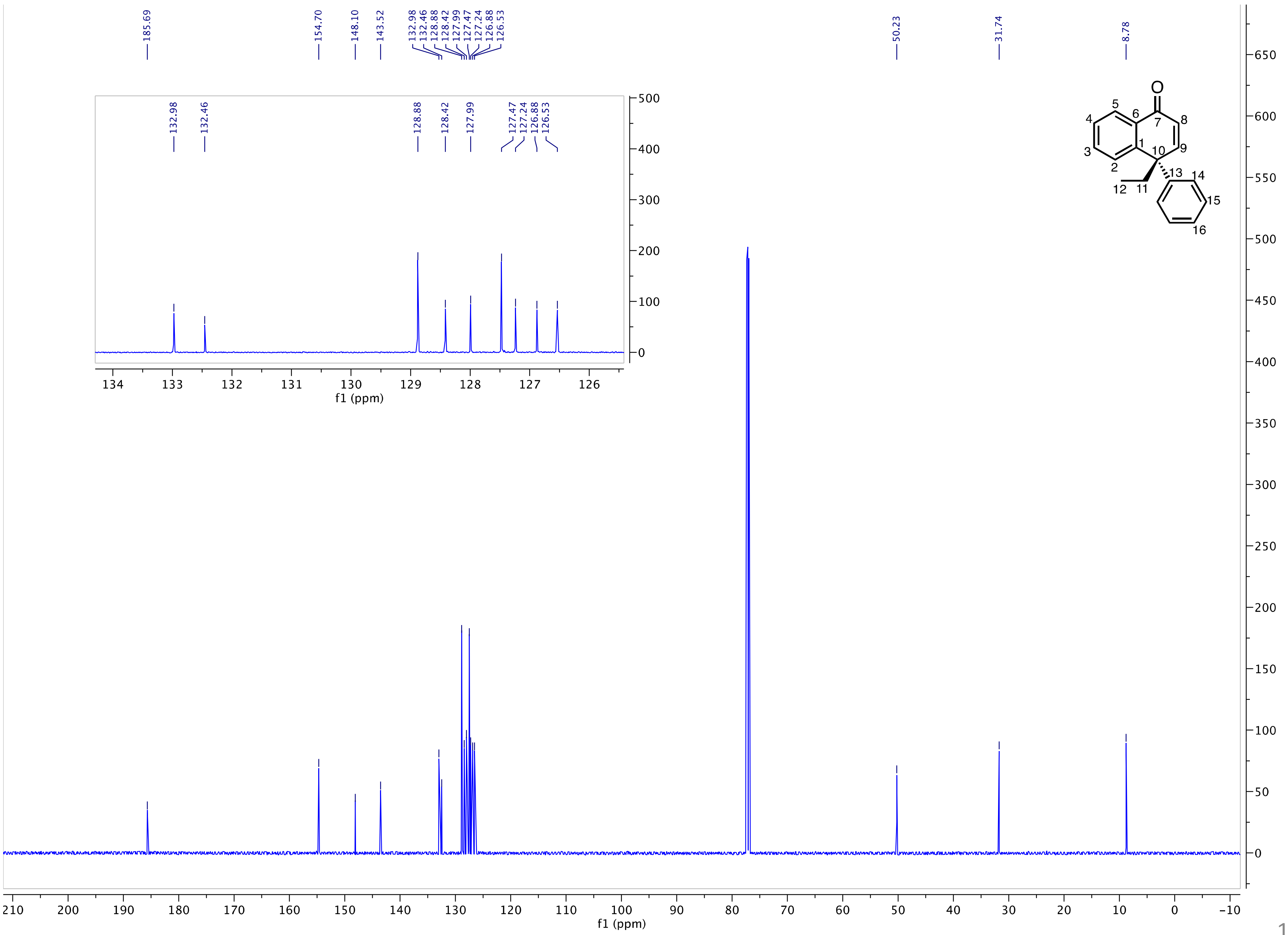

^{1}H NMR ($CDCl_3$): (*R*)-4-Hexyl-4-phenylnaphthalen-1(4*H*)-one (**2c**)

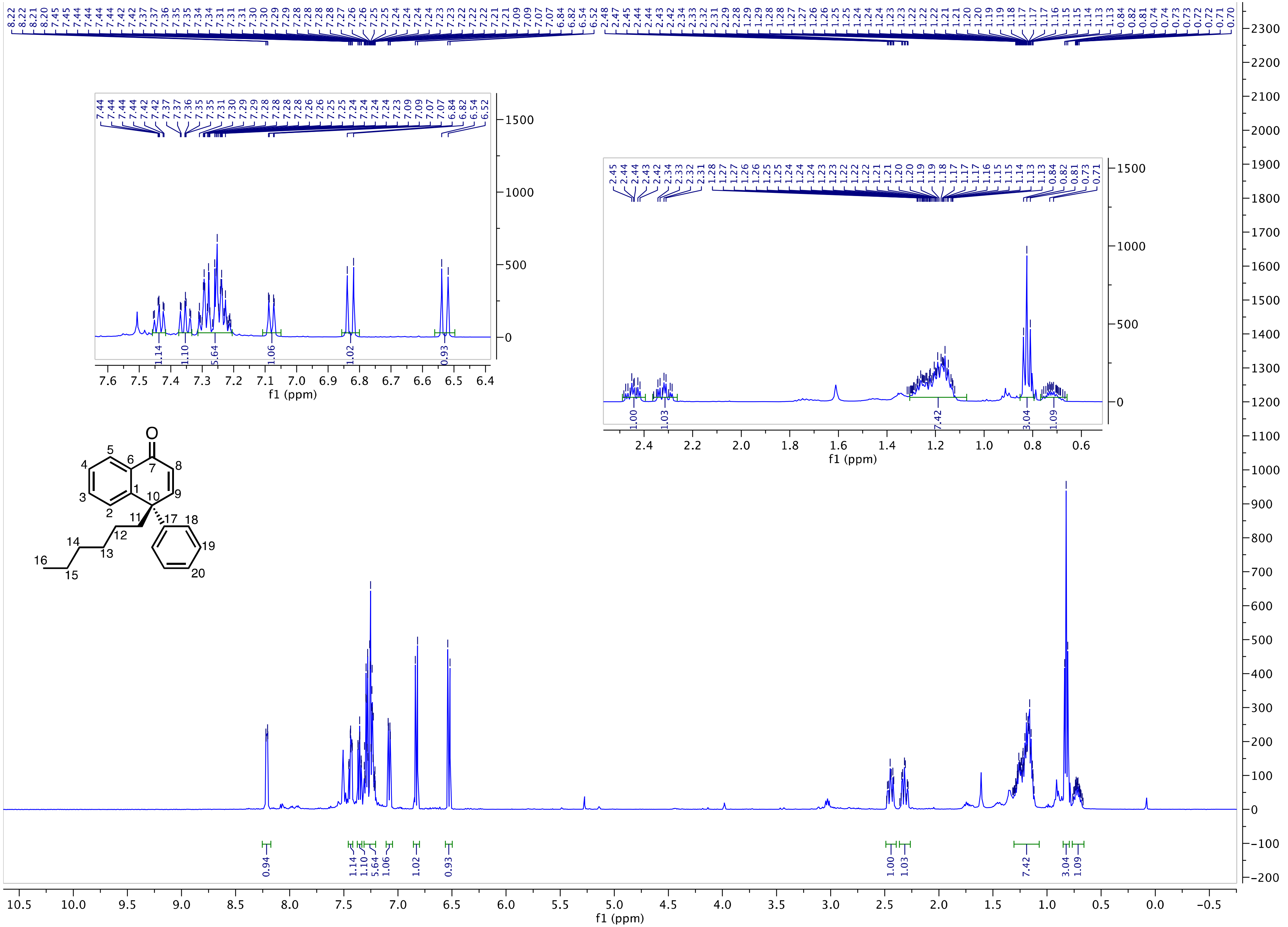

^{13}C **NMR** (CDCl$_3$): (*R*)-4-Hexyl-4-phenylnaphthalen-1(4*H*)-one (**2c**)

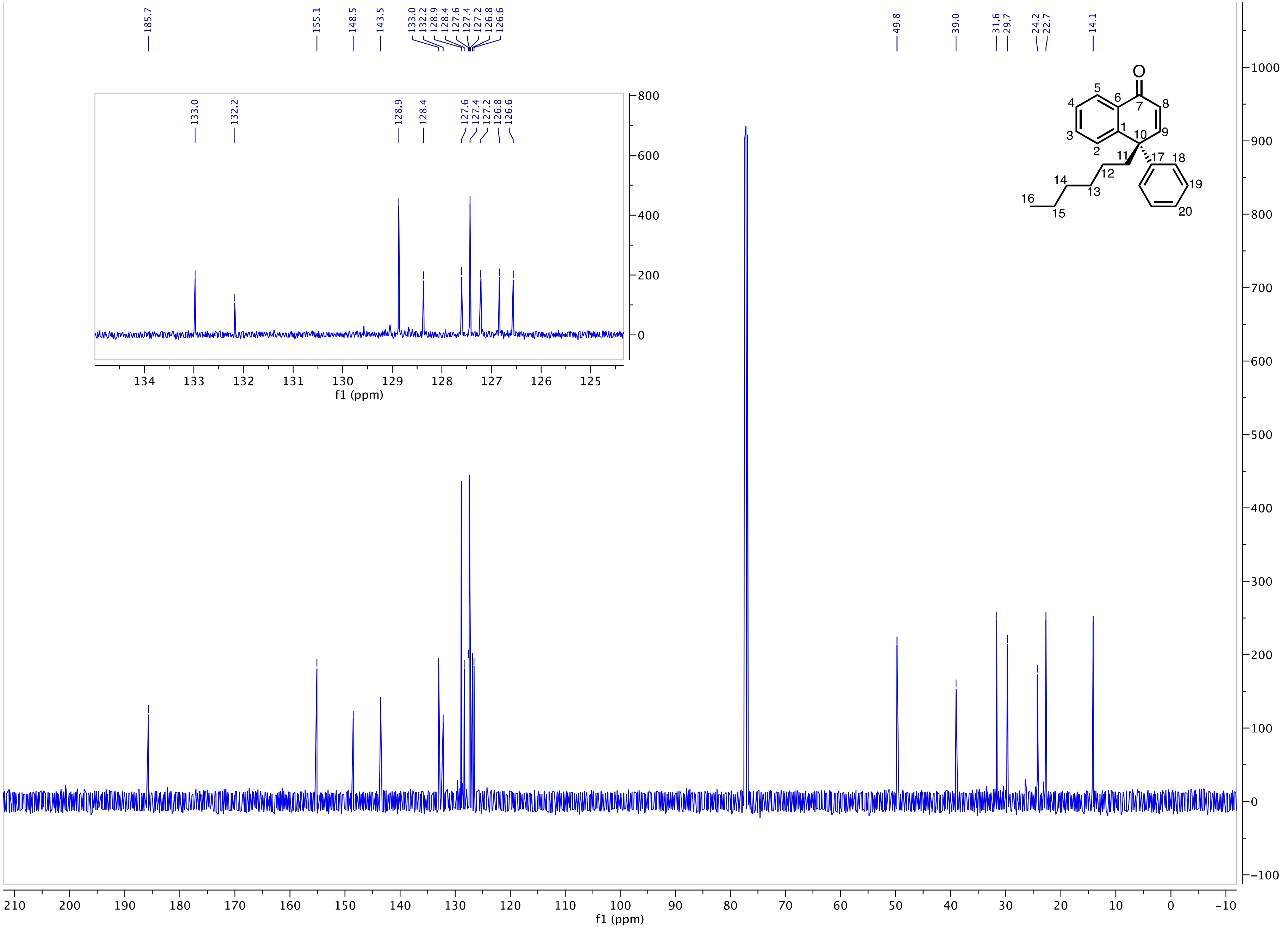

^{1}H NMR ($CDCl_3$): (*R*)-4-Phenethyl-4-phenylnaphthalen-1(4*H*)-one (**2d**)

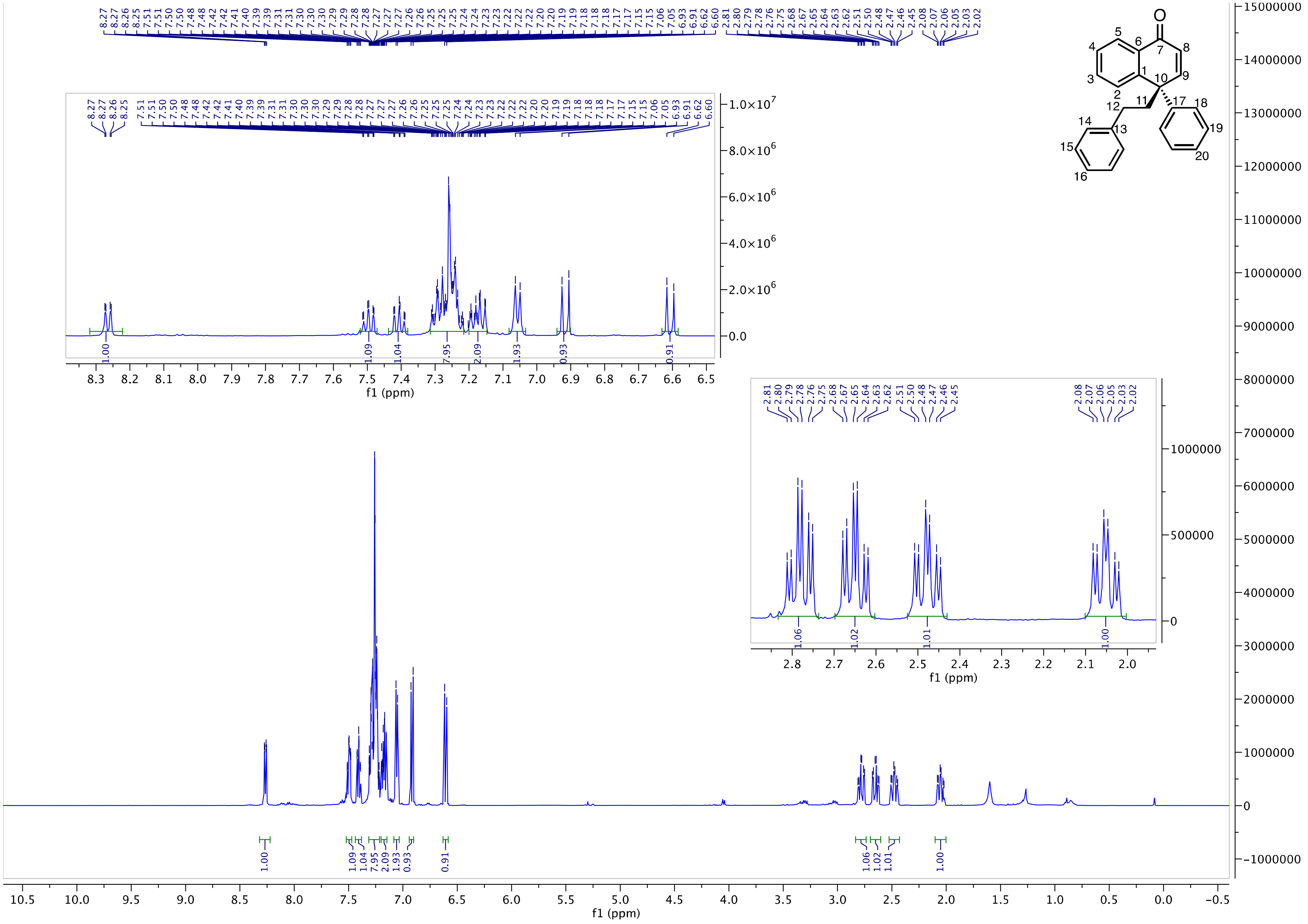

^{13}C NMR ($CDCl_3$): (*R*)-4-Phenethyl-4-phenylnaphthalen-1(4*H*)-one (**2d**)

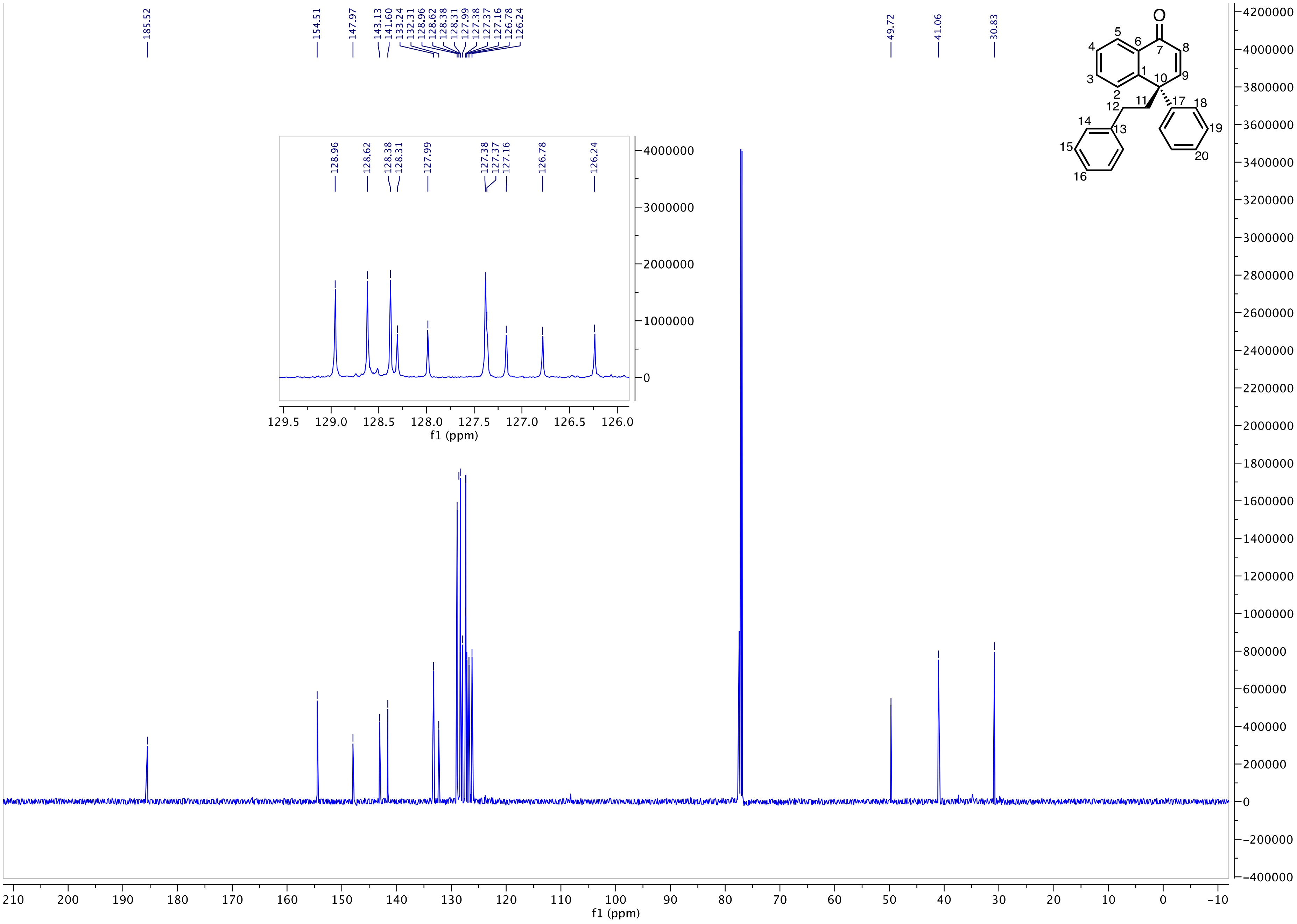

^{1}H NMR ($CDCl_3$): (*R*)-2a-Phenyl-2,2a-dihydroacenaphthylen-5(1*H*)-one (**2e**)

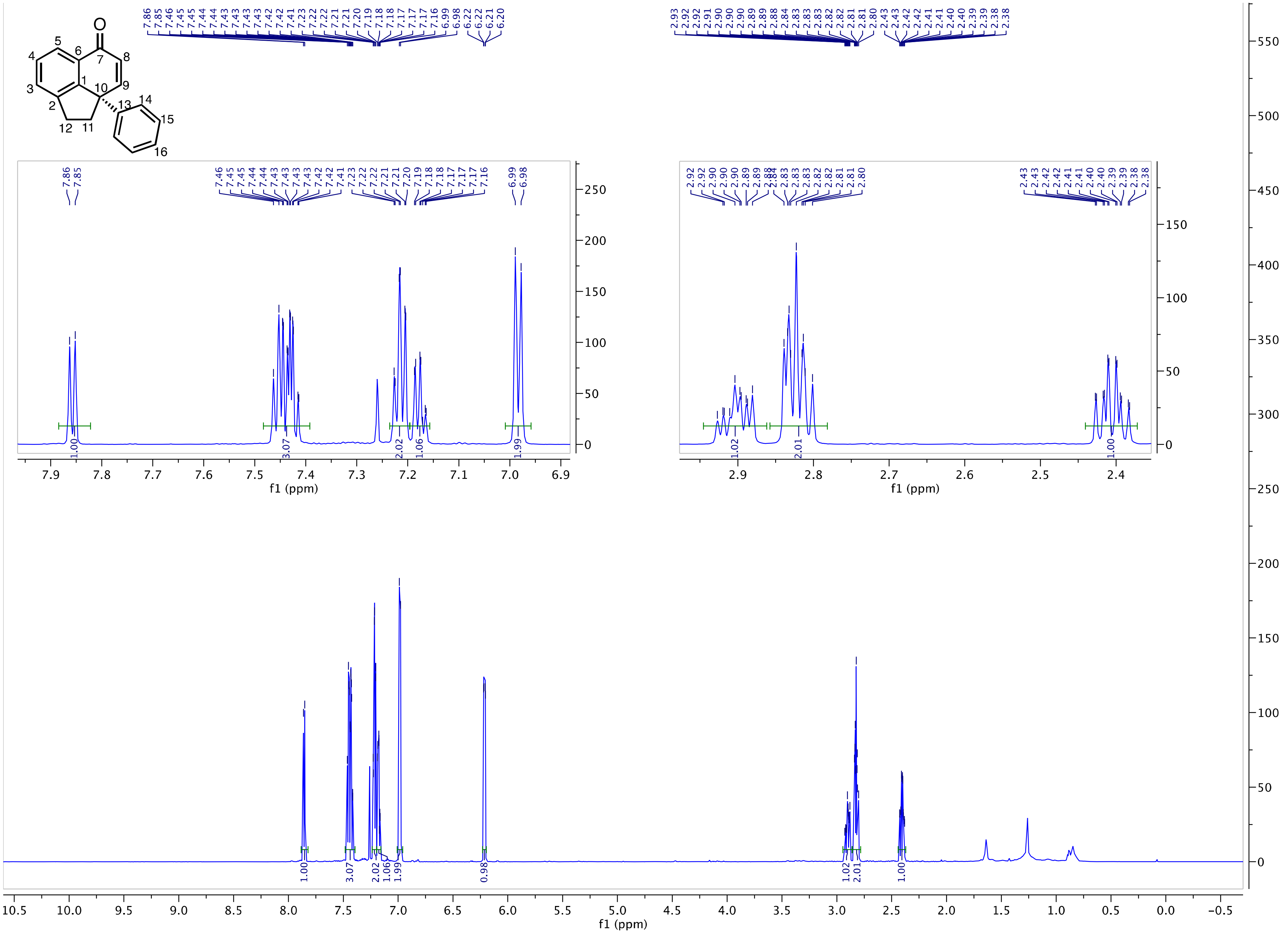

^{13}C NMR ($CDCl_3$): (*R*)-2a-Phenyl-2,2a-dihydroacenaphthylen-5(1*H*)-one (**2e**)

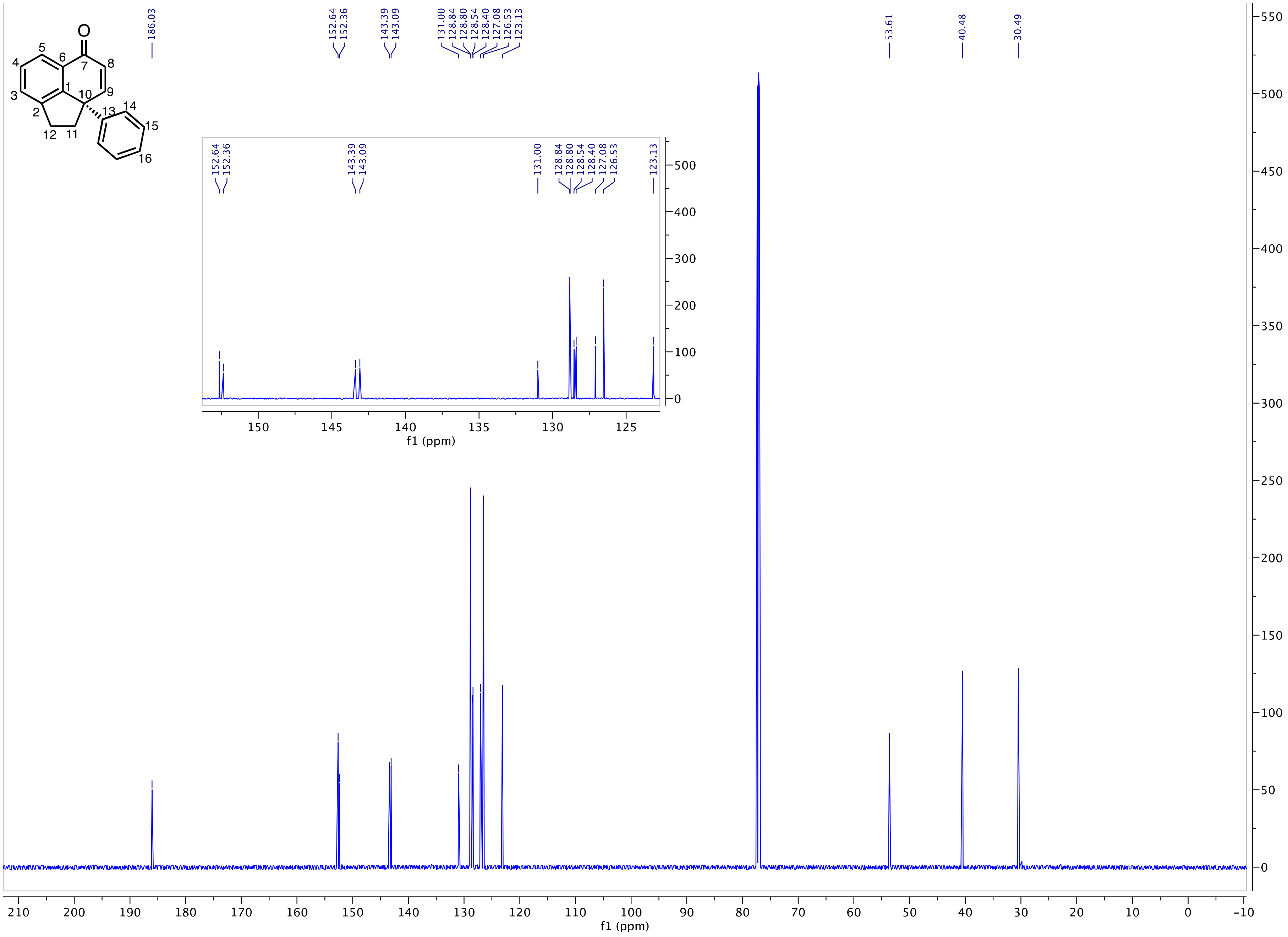

^{1}H NMR ($CDCl_3$): (*R*)-6-Methoxy-4-methyl-4-phenylnaphthalen-1(4*H*)-one (**2f**)

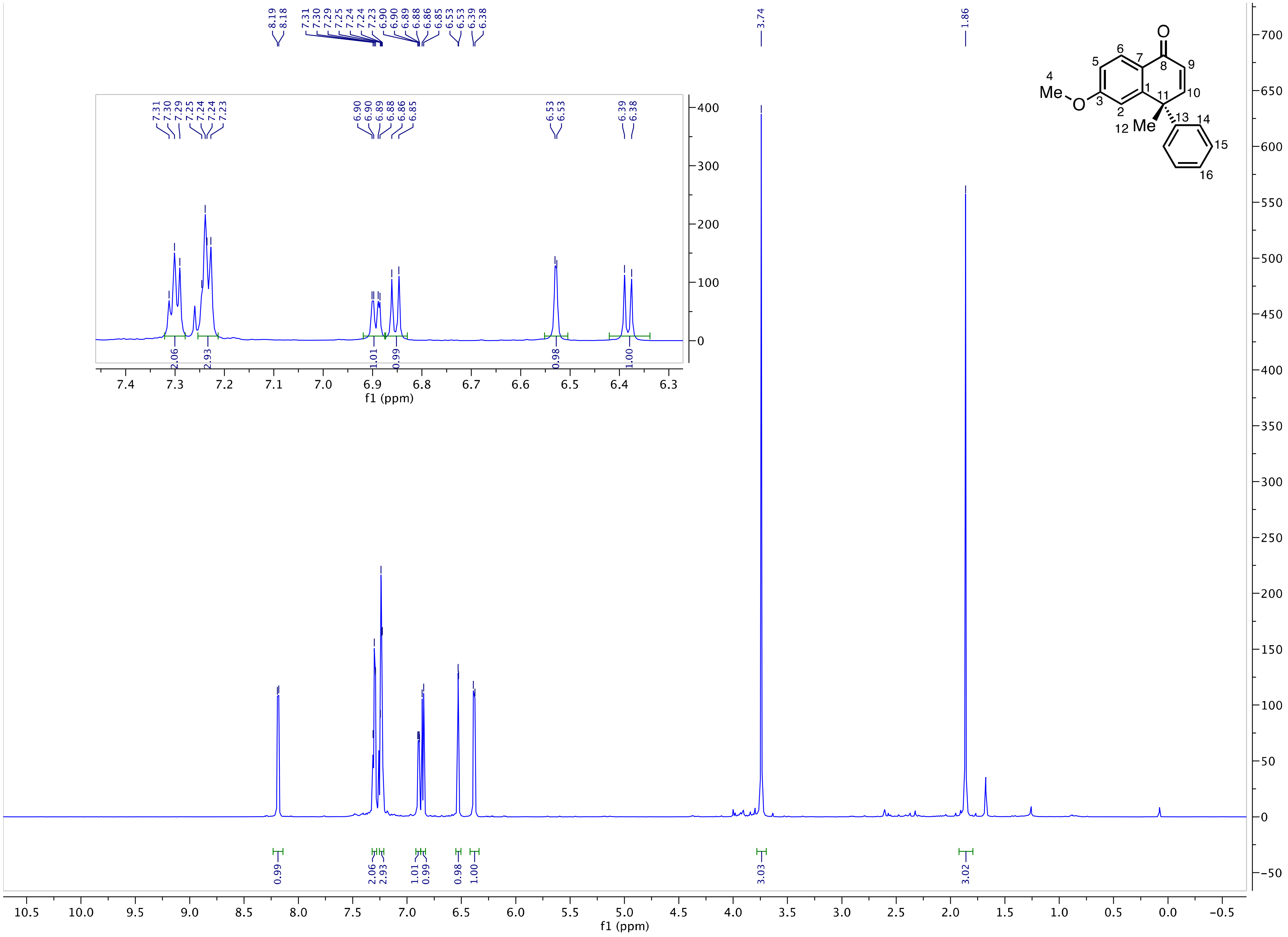

^{13}C NMR (CDCl$_3$): (*R*)-6-Methoxy-4-methyl-4-phenylnaphthalen-1(4*H*)-one (**2f**)

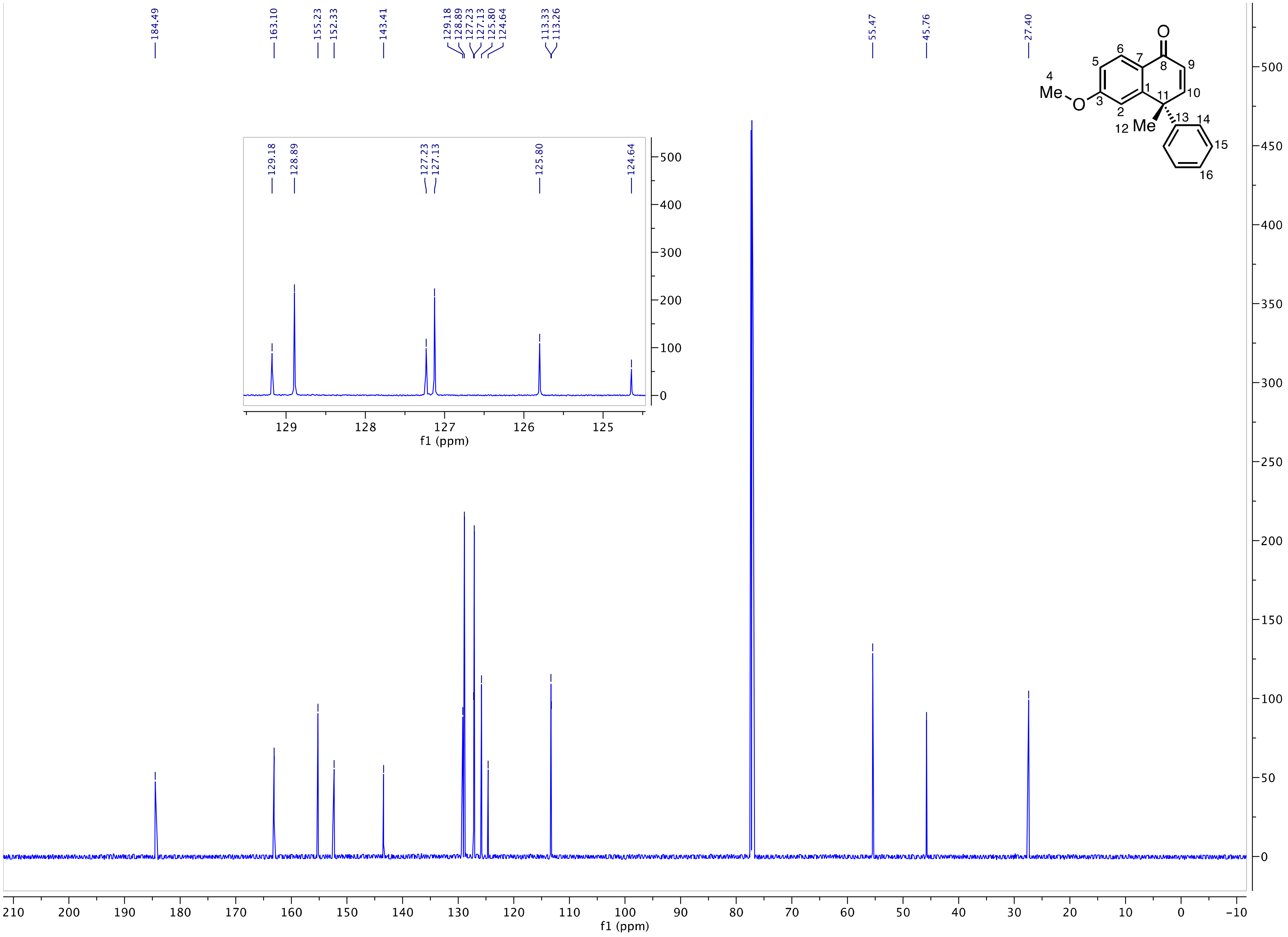

^{1}H NMR ($CDCl_3$): (*R*)-7-Methoxy-4-methyl-4-phenylnaphthalen-1(4*H*)-one (**2g**)

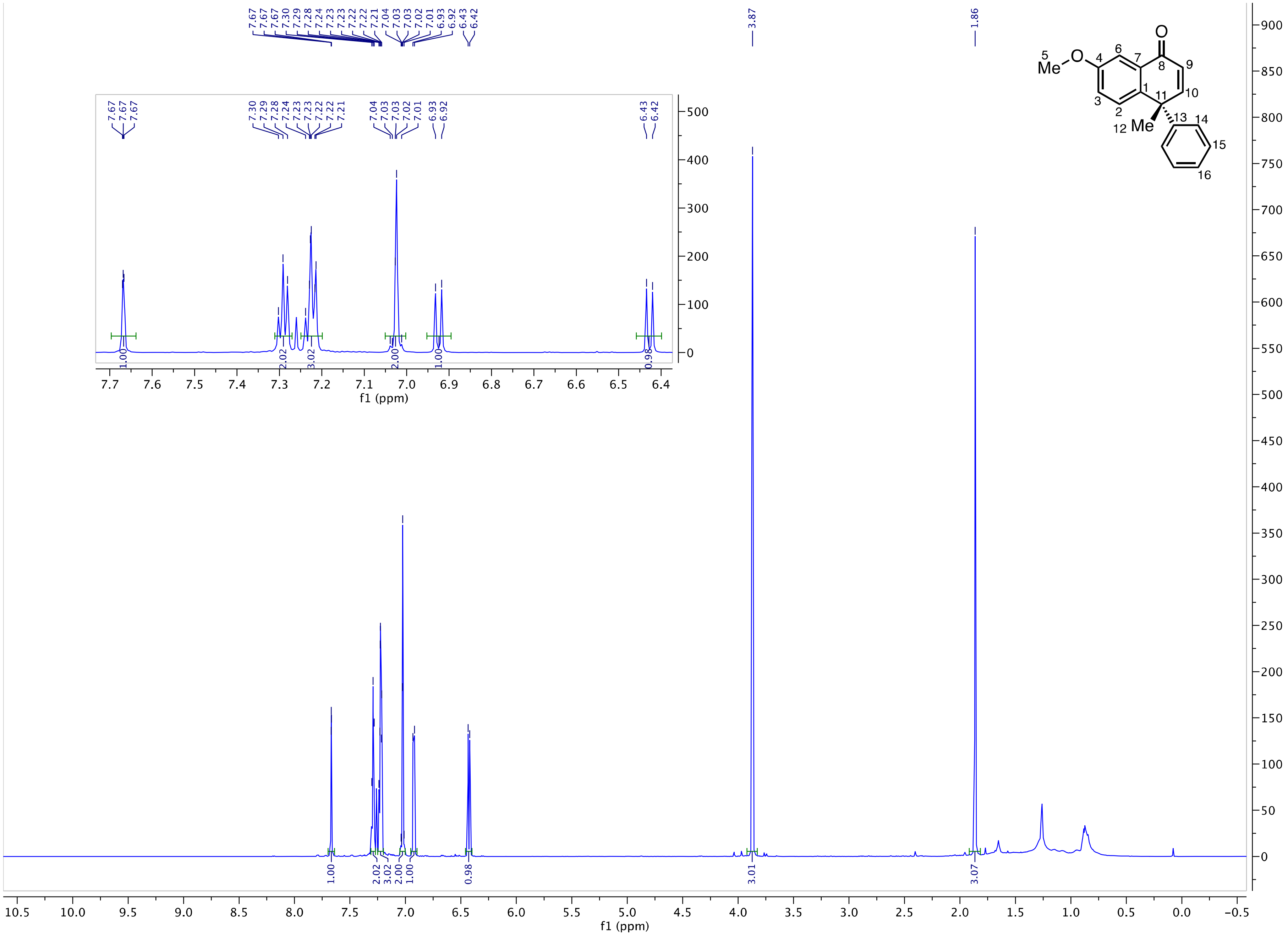

^{13}C NMR (CDCl$_3$): (*R*)-7-Methoxy-4-methyl-4-phenylnaphthalen-1(4*H*)-one (**2g**)

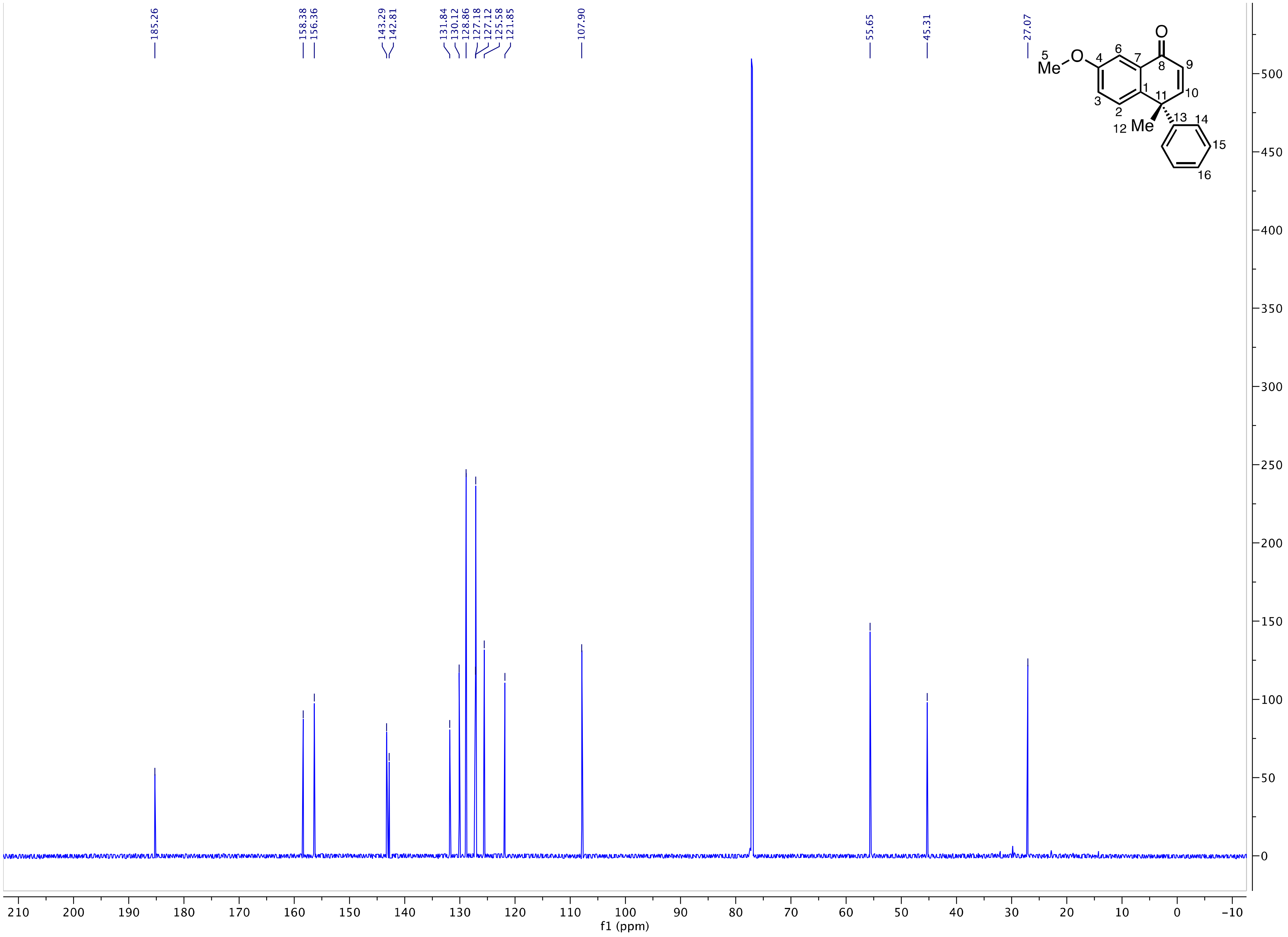

^{1}H NMR (CDCl$_3$): (*R*)-7-(methoxymethoxy)-4,6-Dimethyl-4-phenylnaphthalen-1(4*H*)-one (**2h**)

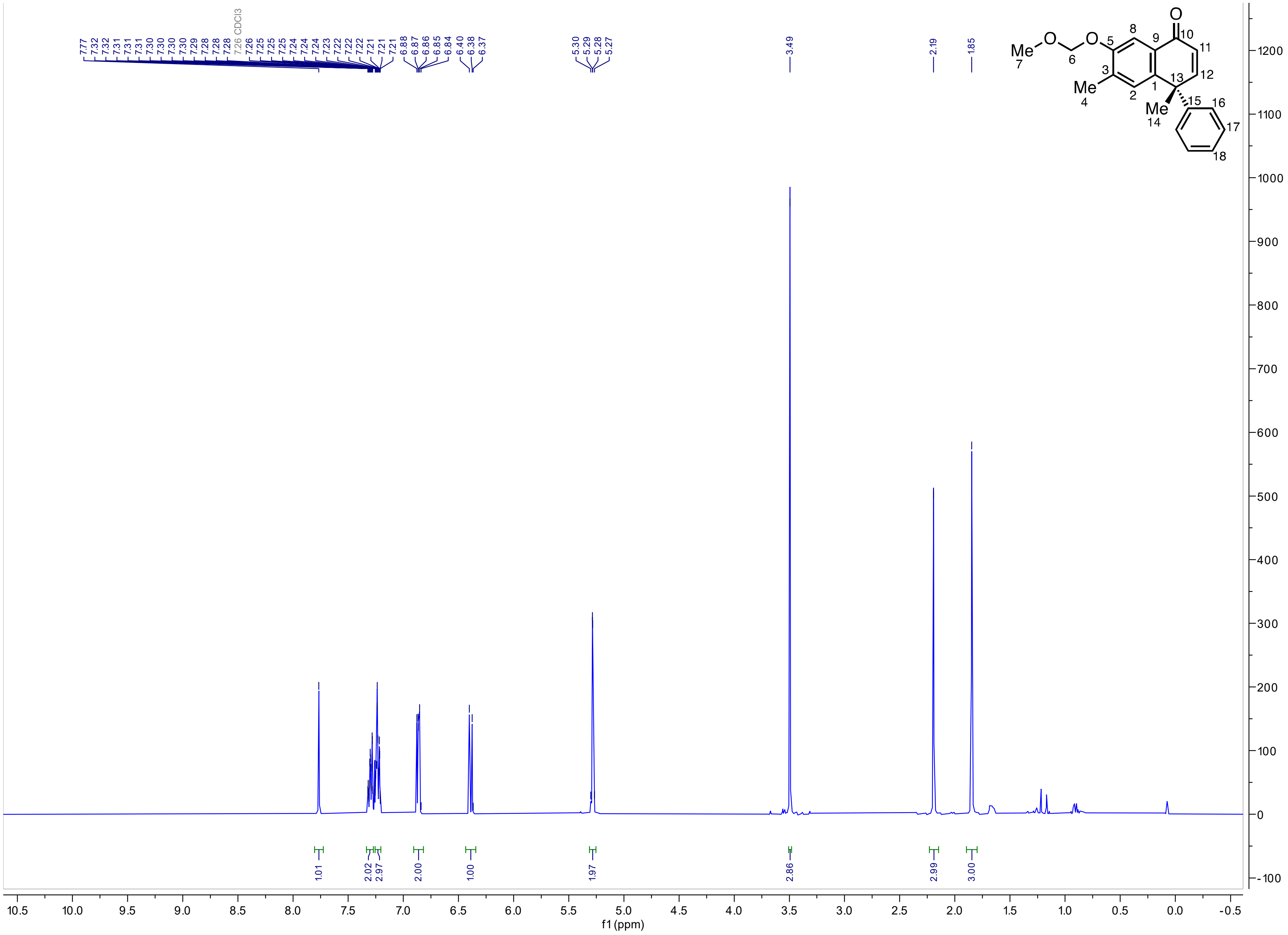

^{13}C NMR ($CDCl_3$): (*R*)-7-(methoxymethoxy)-4,6-Dimethyl-4-phenylnaphthalen-1(4*H*)-one (**2h**)

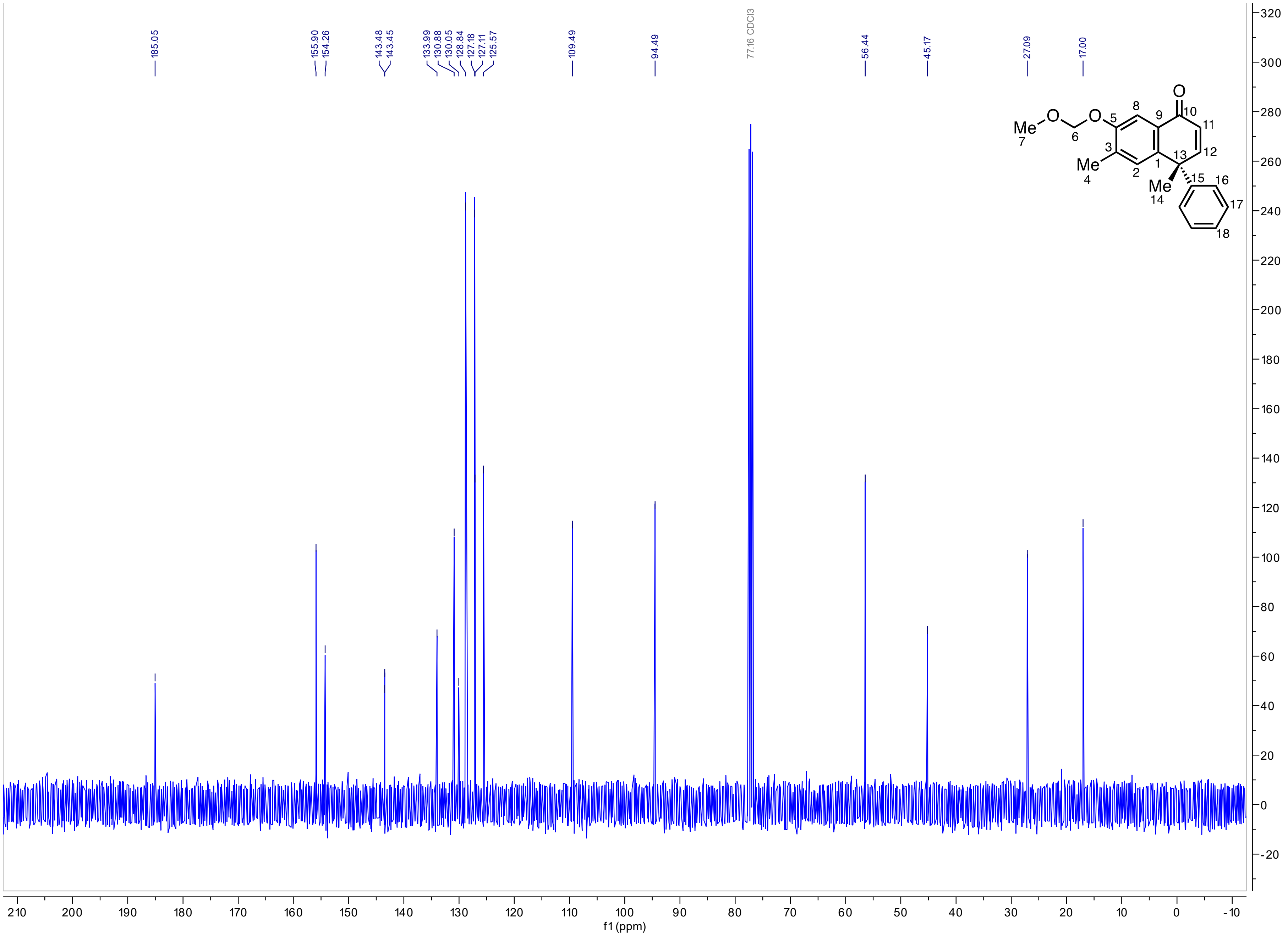

^{1}H NMR ($CDCl_3$): (*R*)-3,4-Dimethyl-4-phenylnaphthalen-1(4*H*)-one (**2i**)

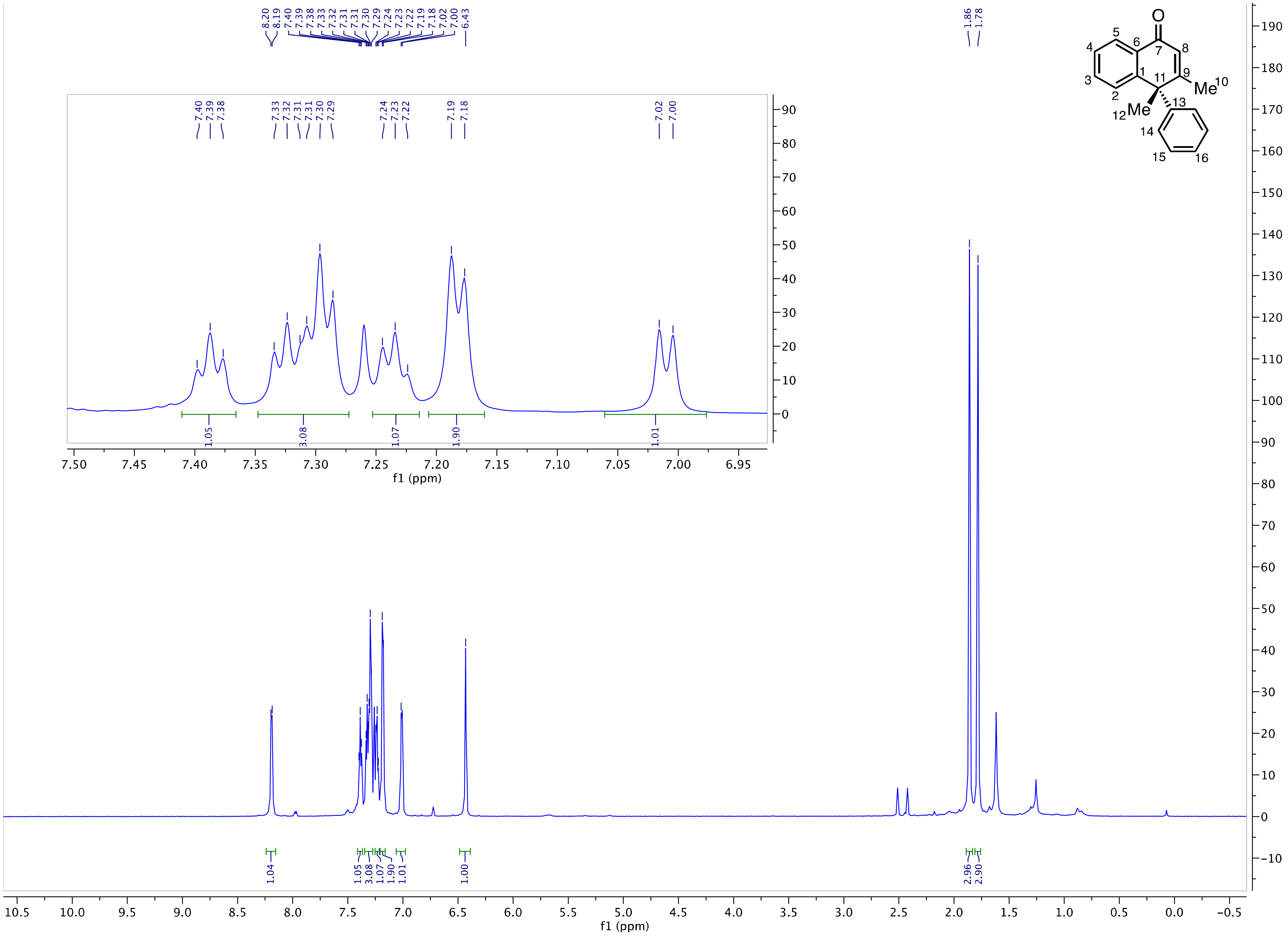

^{13}C NMR (CDCl$_3$): (*R*)-3,4-Dimethyl-4-phenylnaphthalen-1(4*H*)-one (**2i**)

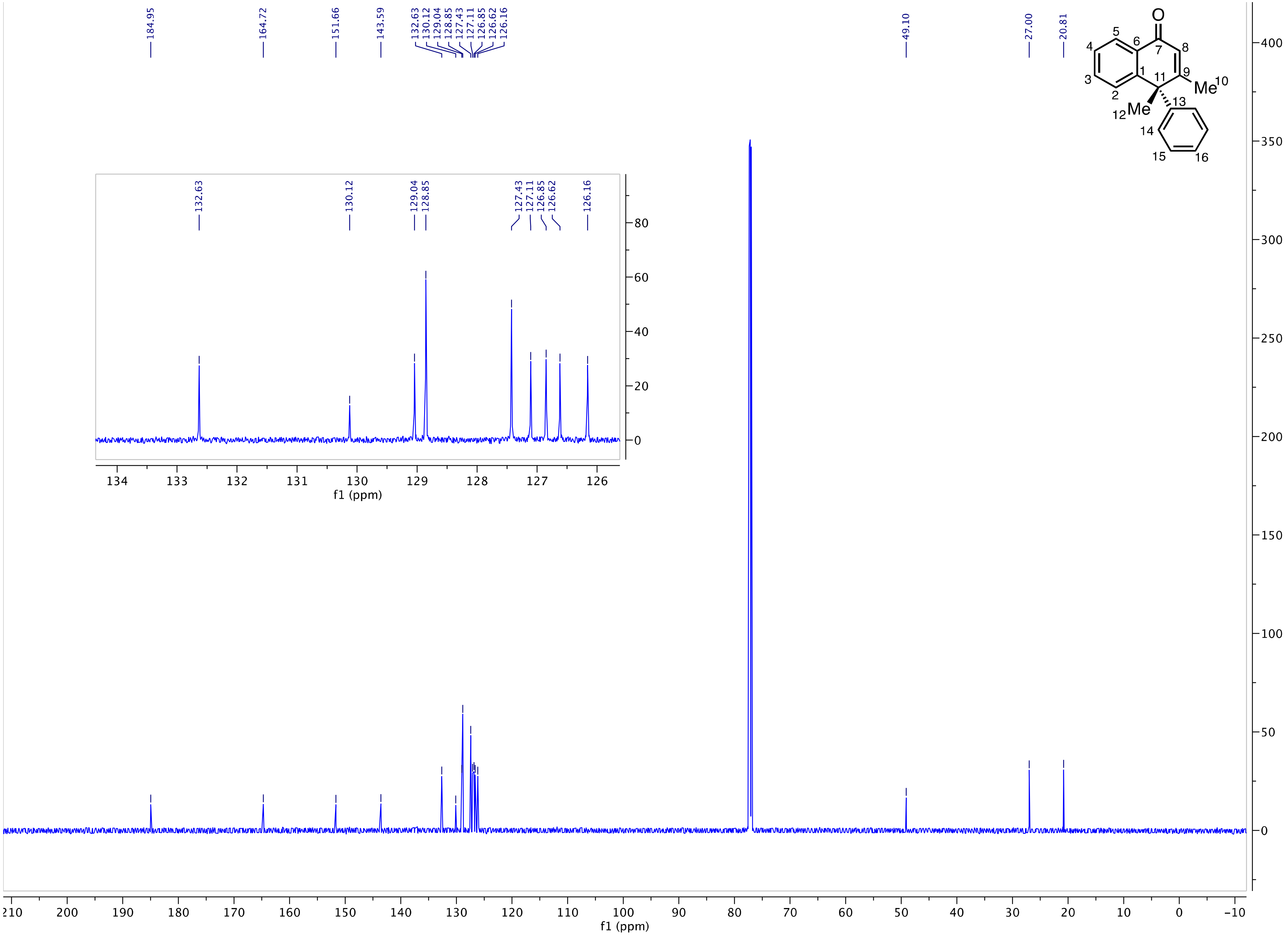

^{1}H NMR ($CDCl_3$): (*R*)-4-(4-methoxyphenyl)-4-Methylnaphthalen-1(4*H*)-one (**2j**)

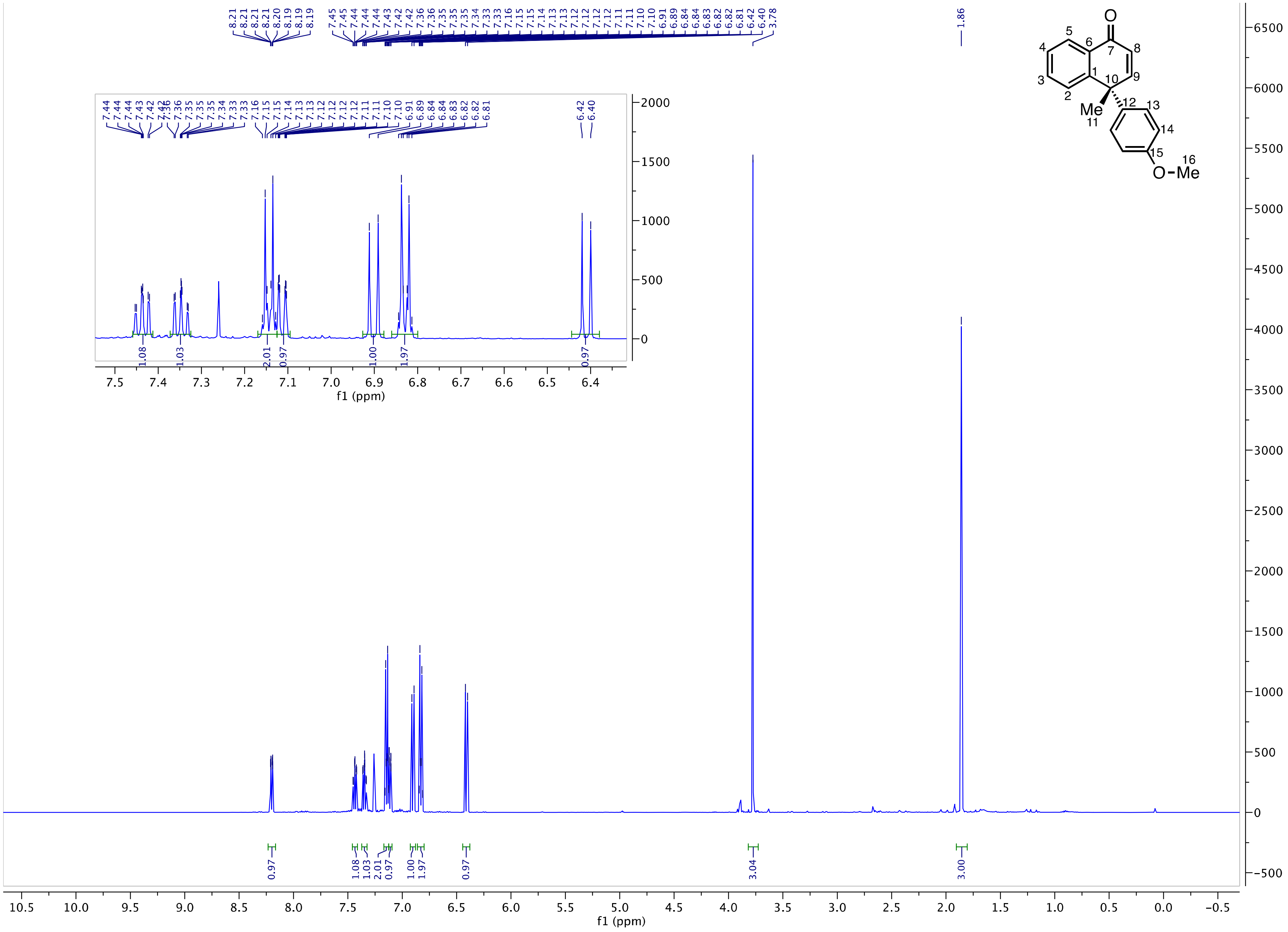

^{13}C NMR (CDCl$_3$): (*R*)-4-(4-methoxyphenyl)-4-Methylnaphthalen-1(4*H*)-one (**2j**)

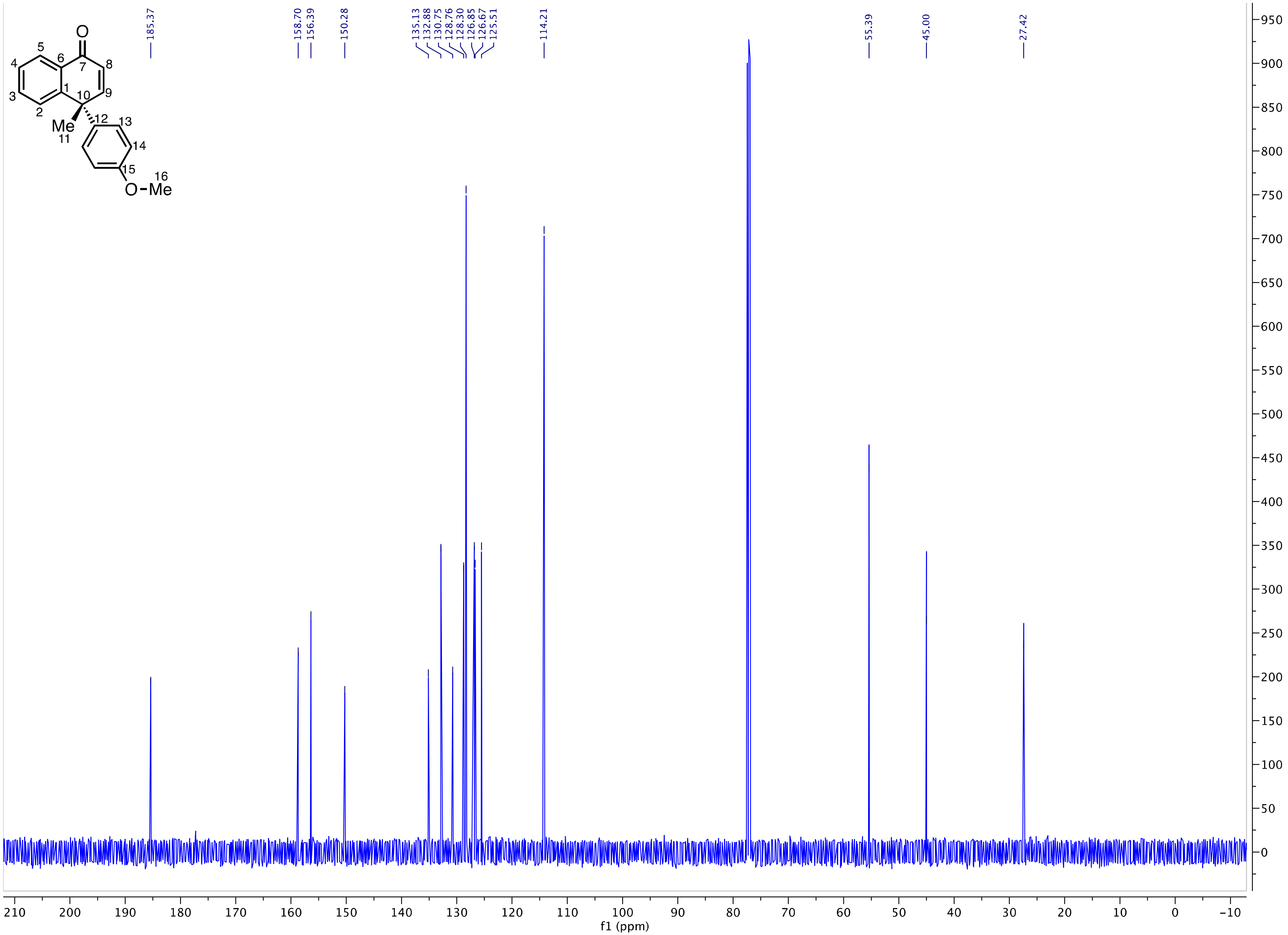

^{1}H NMR (CDCl$_3$): (*R*)-4-Methyl-4-(3,4,5-trimethoxyphenyl)naphthalen-1(4*H*)-one (**2k**)

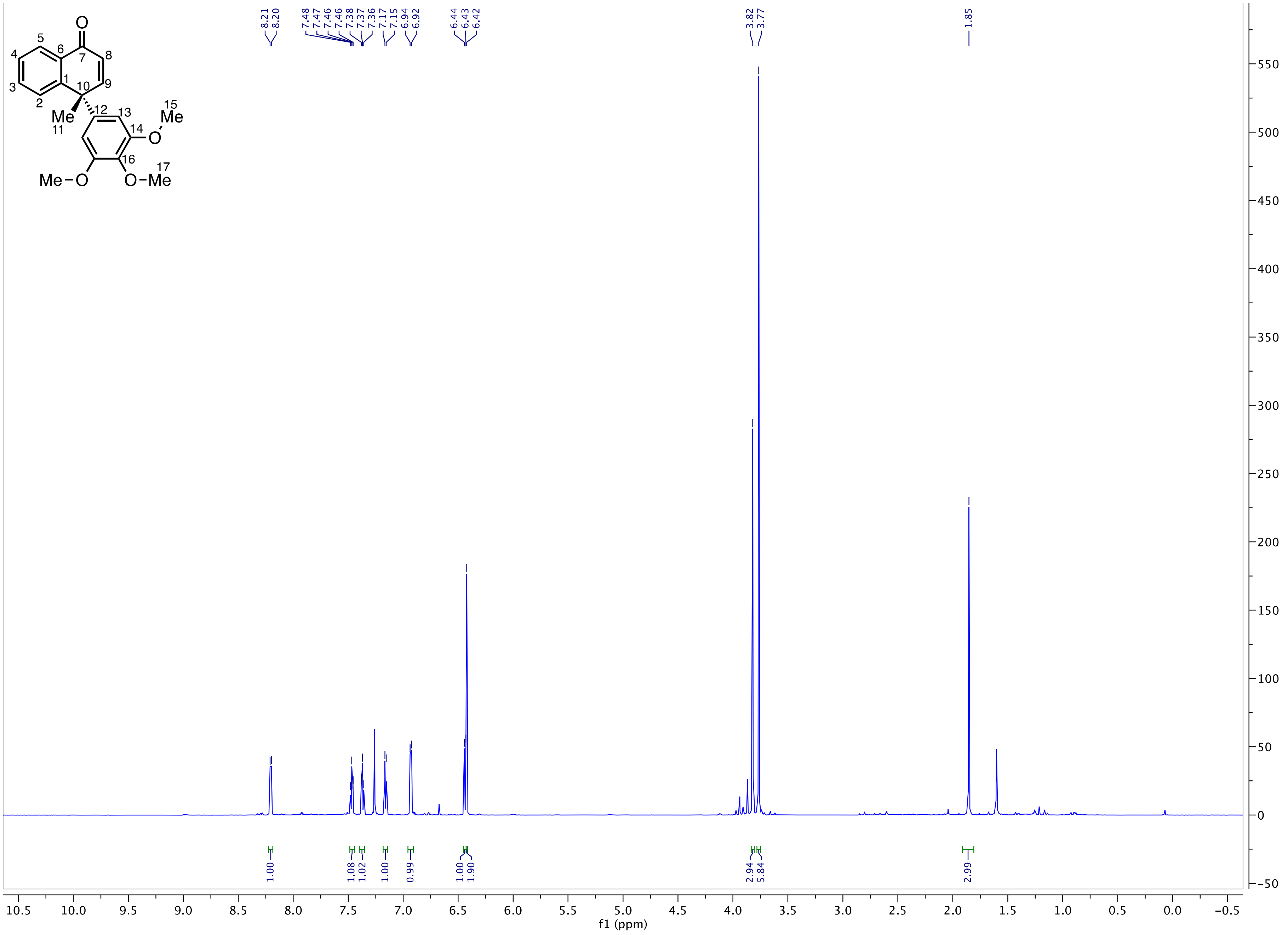

^{13}C NMR (CDCl$_3$): (*R*)-4-Methyl-4-(3,4,5-trimethoxyphenyl)naphthalen-1(4*H*)-one (**2k**)

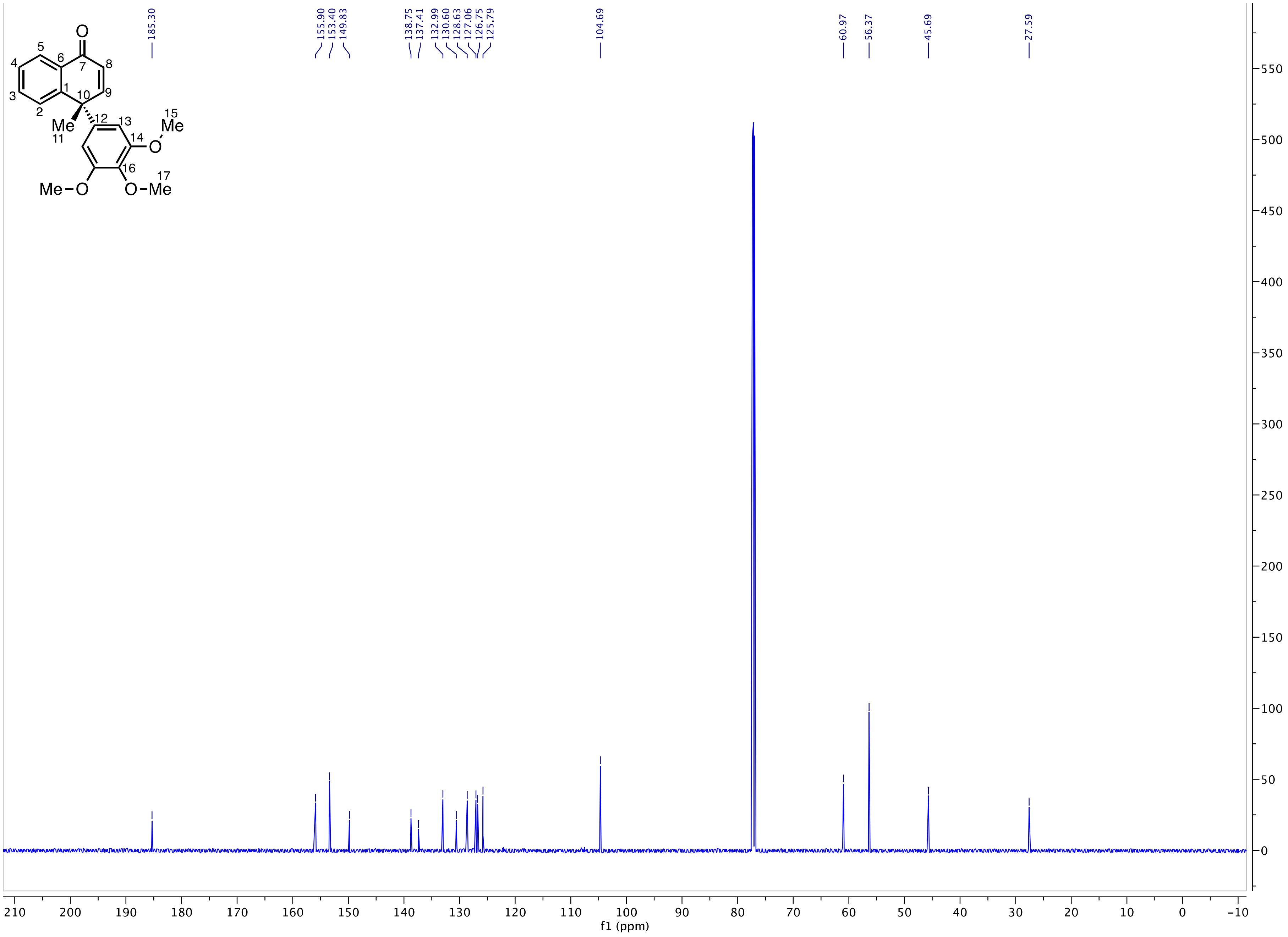

^{1}H NMR ($CDCl_3$): (*S*)-4-(2-methoxyphenyl)-4-Methylnaphthalen-1(4*H*)-one (**2l**)

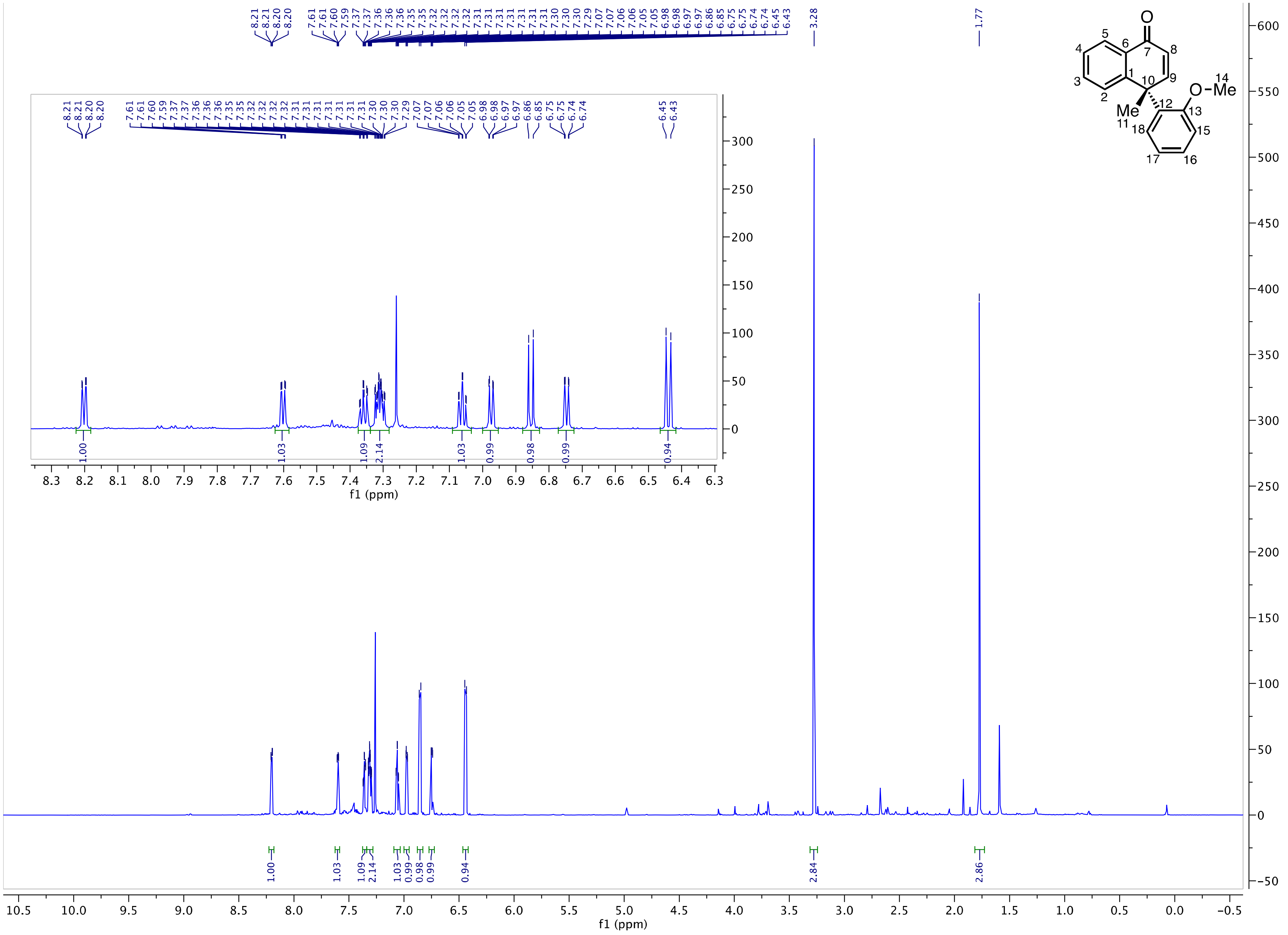

^{13}C NMR ($CDCl_3$): (*S*)-4-(2-methoxyphenyl)-4-Methylnaphthalen-1(4*H*)-one (**2l**)

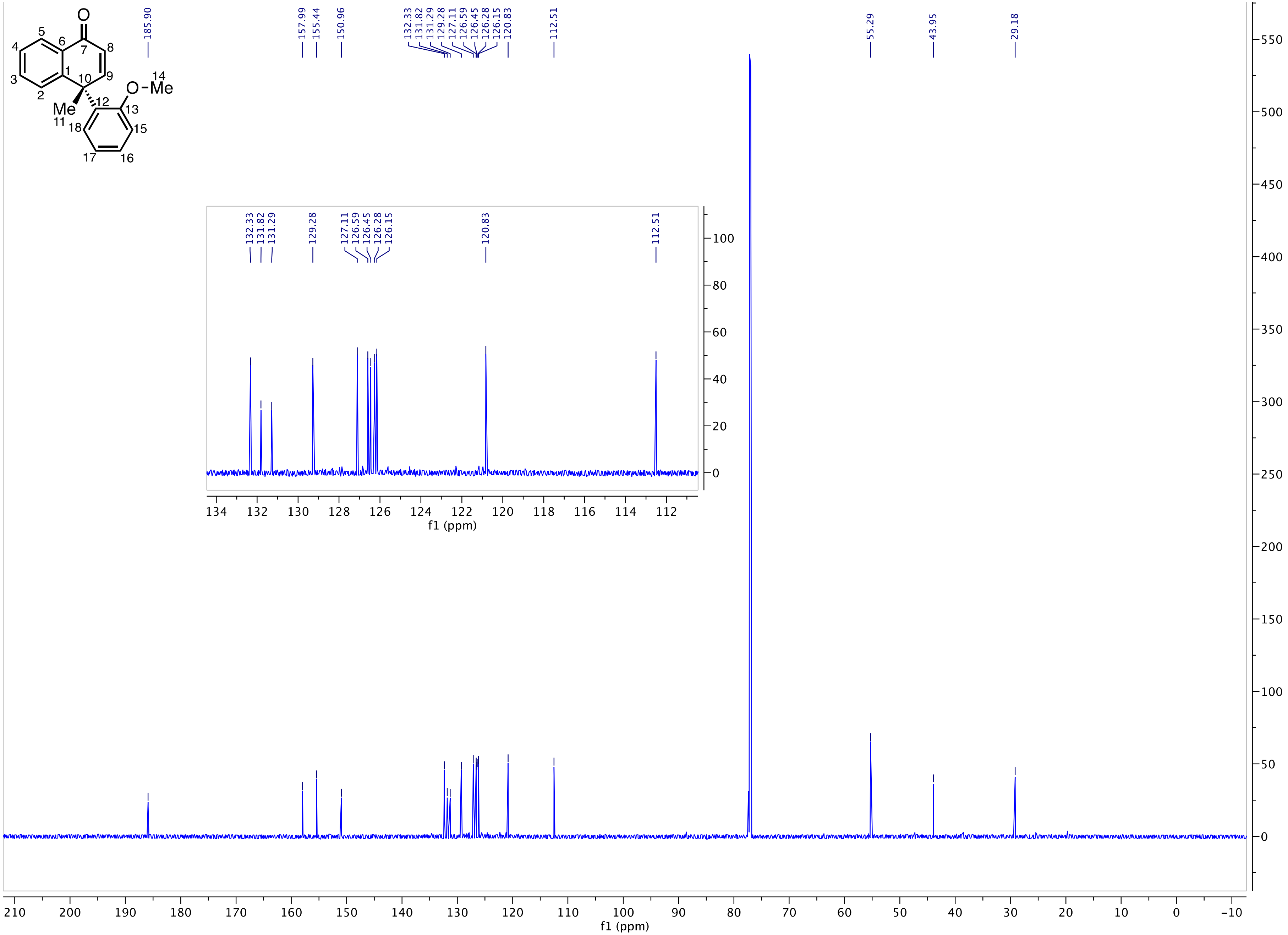

^{1}H NMR ($CDCl_3$): (*R*)-4-(3-fluoro-5-methoxyphenyl)-4-Methylnaphthalen-1(4*H*)-one (**2m**)

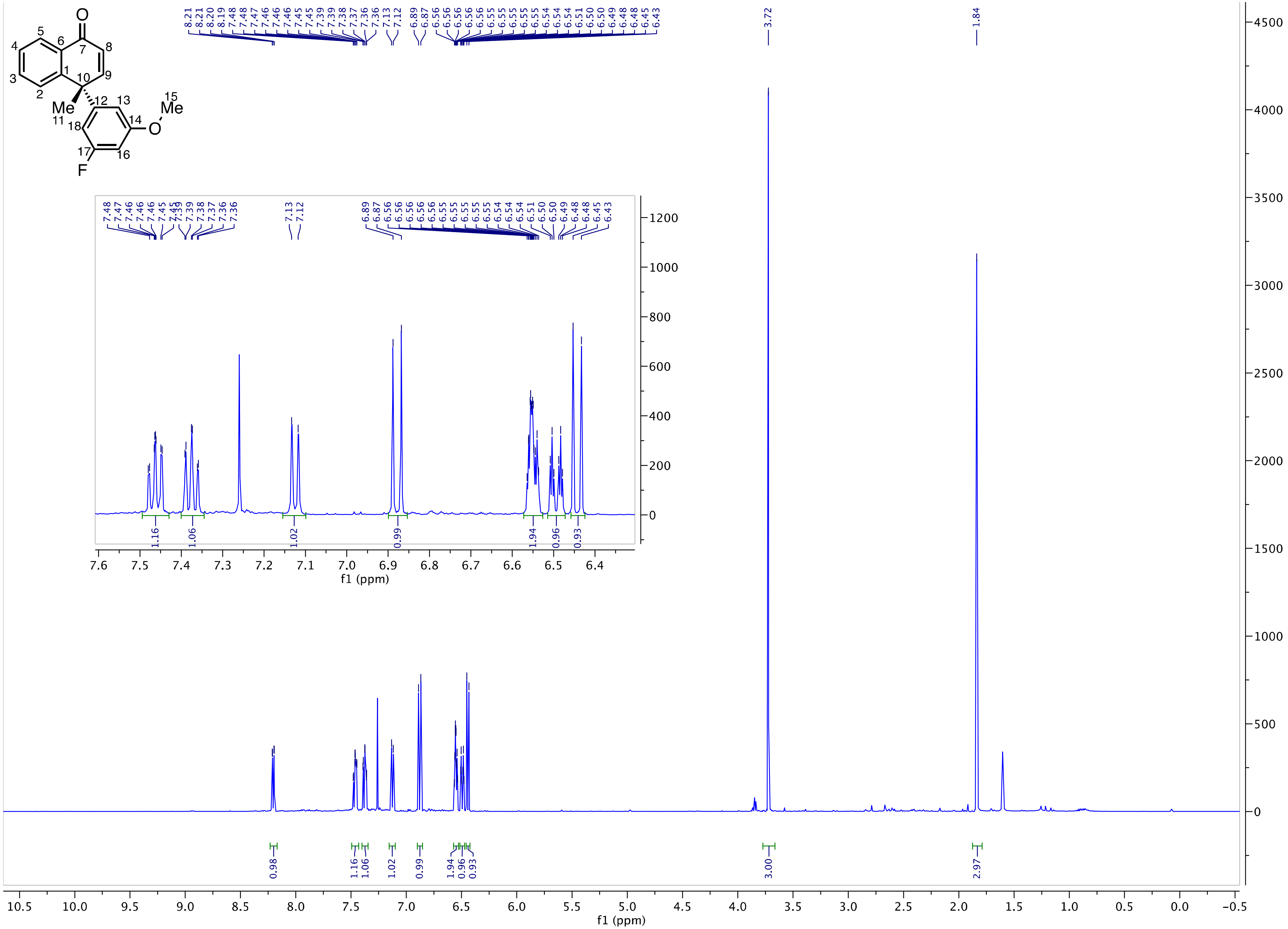

^{13}C NMR ($CDCl_3$): (*R*)-4-(3-fluoro-5-methoxyphenyl)-4-Methylnaphthalen-1(4*H*)-one (**2m**)

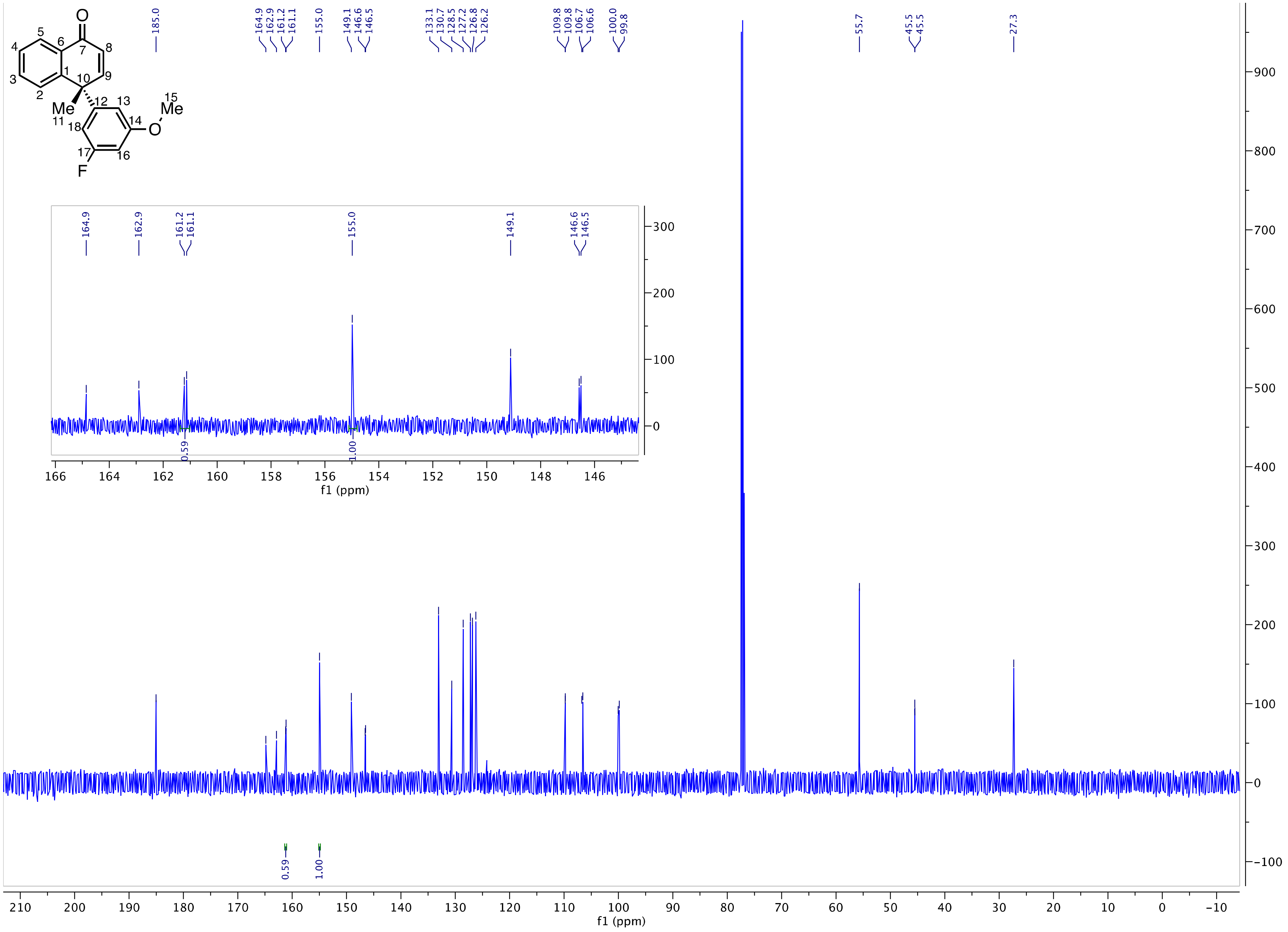

^{19}**F NMR** ($CDCl_3$): (*R*)-4-(3-fluoro-5-methoxyphenyl)-4-Methylnaphthalen-1(4*H*)-one (**2m**)

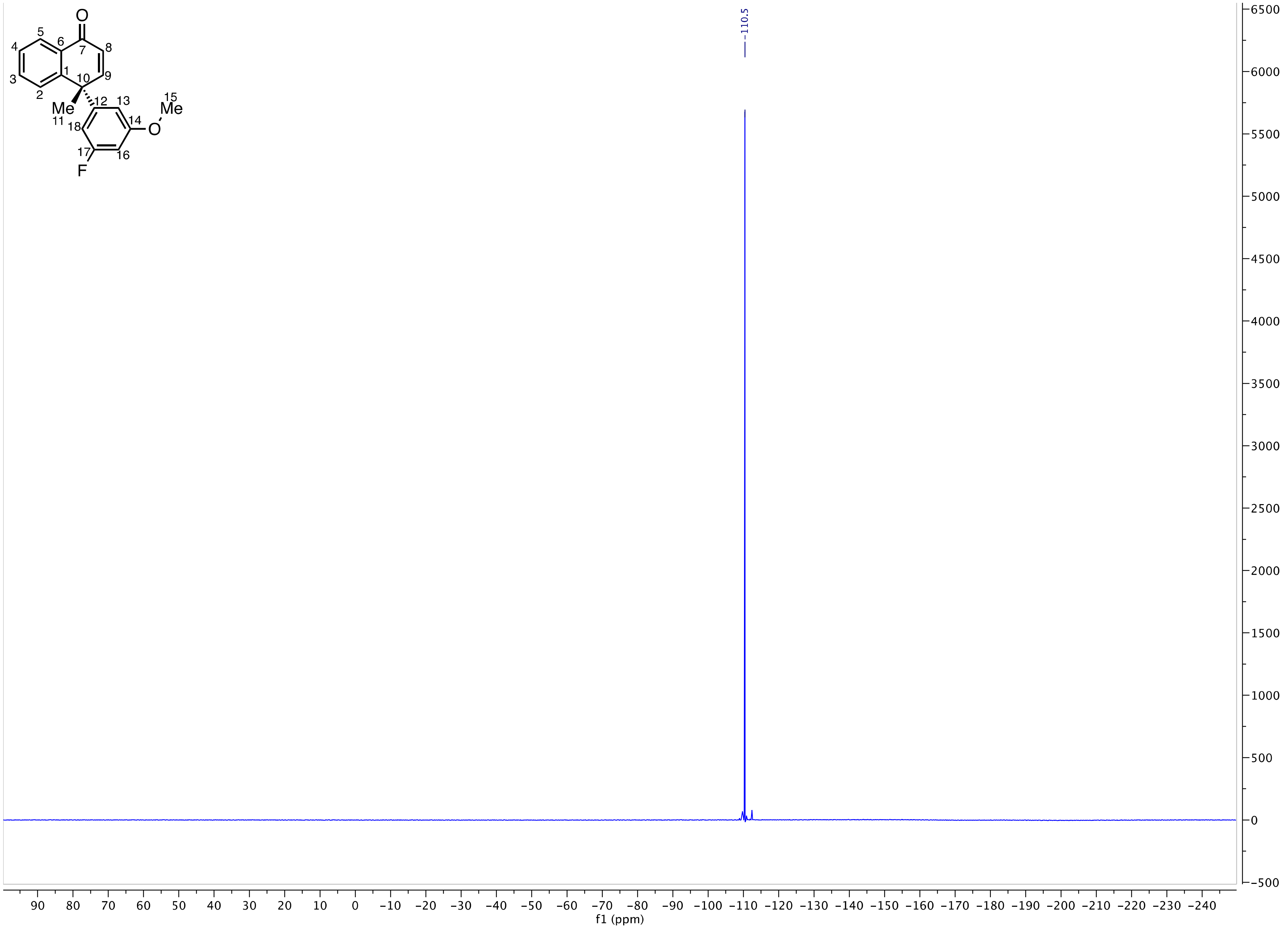

^{1}**H NMR** ($CDCl_3$): (*R*)-4-(3,4-bis(methoxymethoxy)phenyl)-4-Methylnaphthalen-1(4*H*)-one (**2n**)

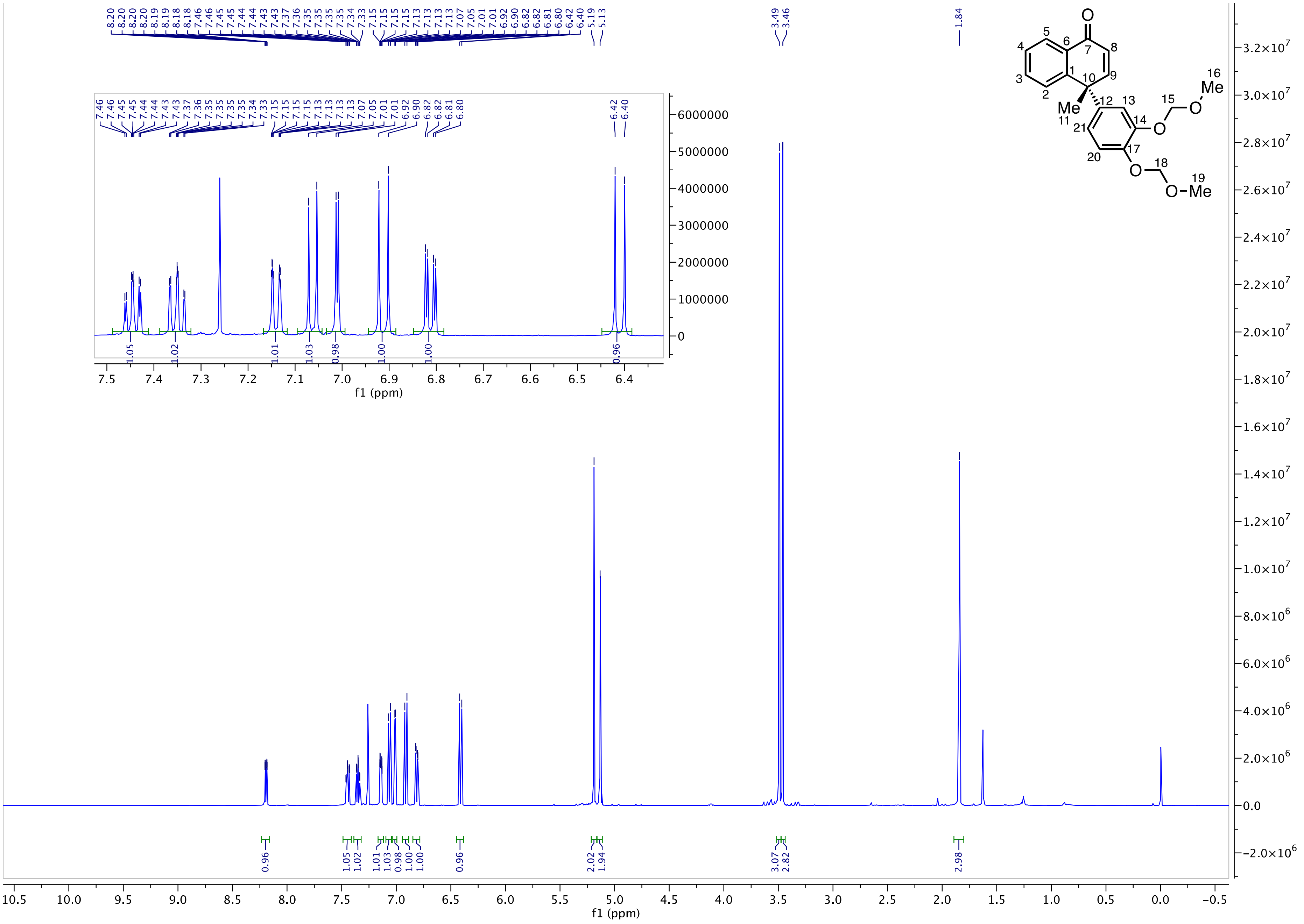

^{13}C NMR ($CDCl_3$): (*R*)-4-(3,4-bis(methoxymethoxy)phenyl)-4-Methylnaphthalen-1(4*H*)-one (**2n**)

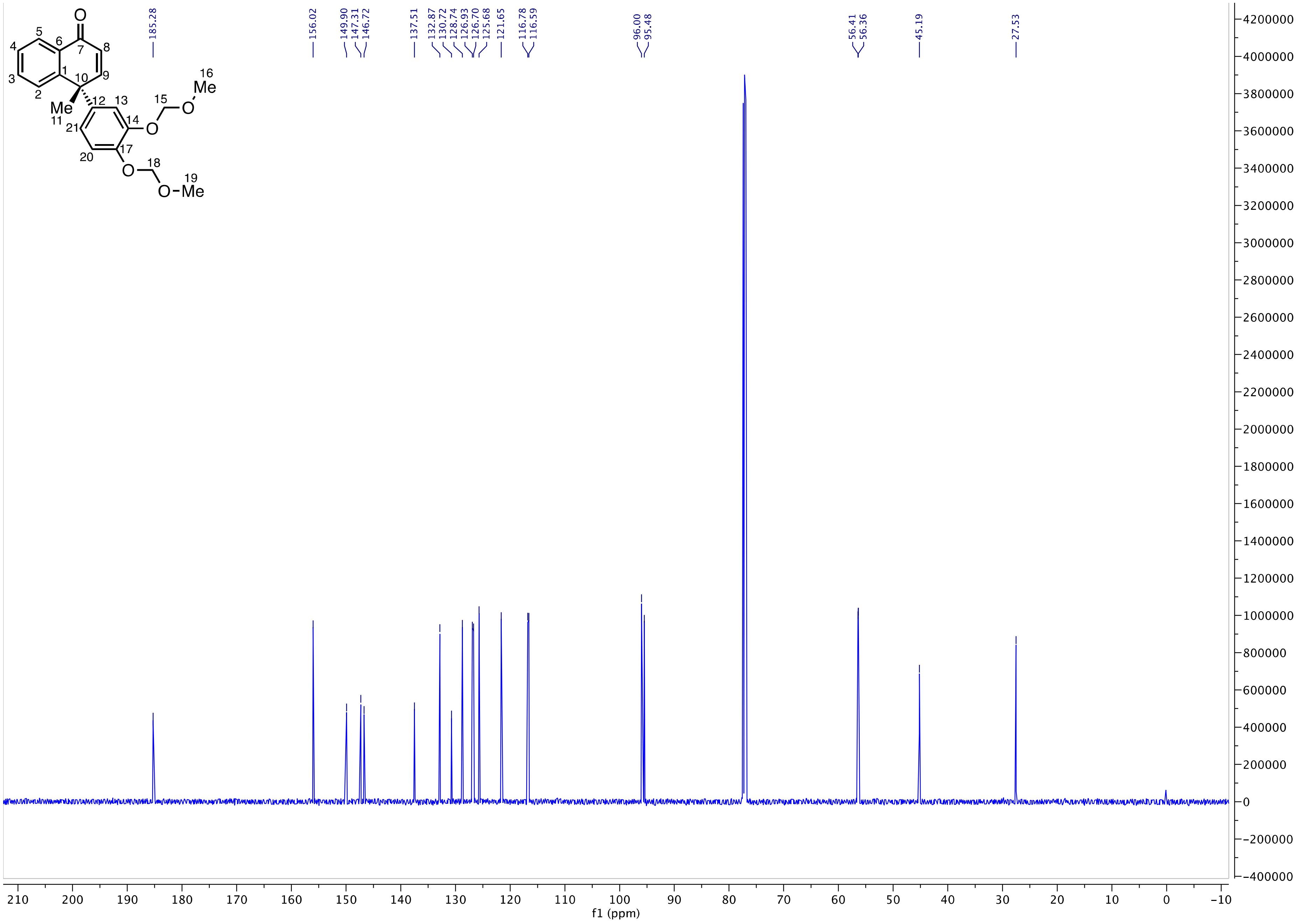

^{1}H NMR ($CDCl_3$): (*R*)-4-(4-(dimethylamino)phenyl)-4-Methylnaphthalen-1(4*H*)-one (**2o**)

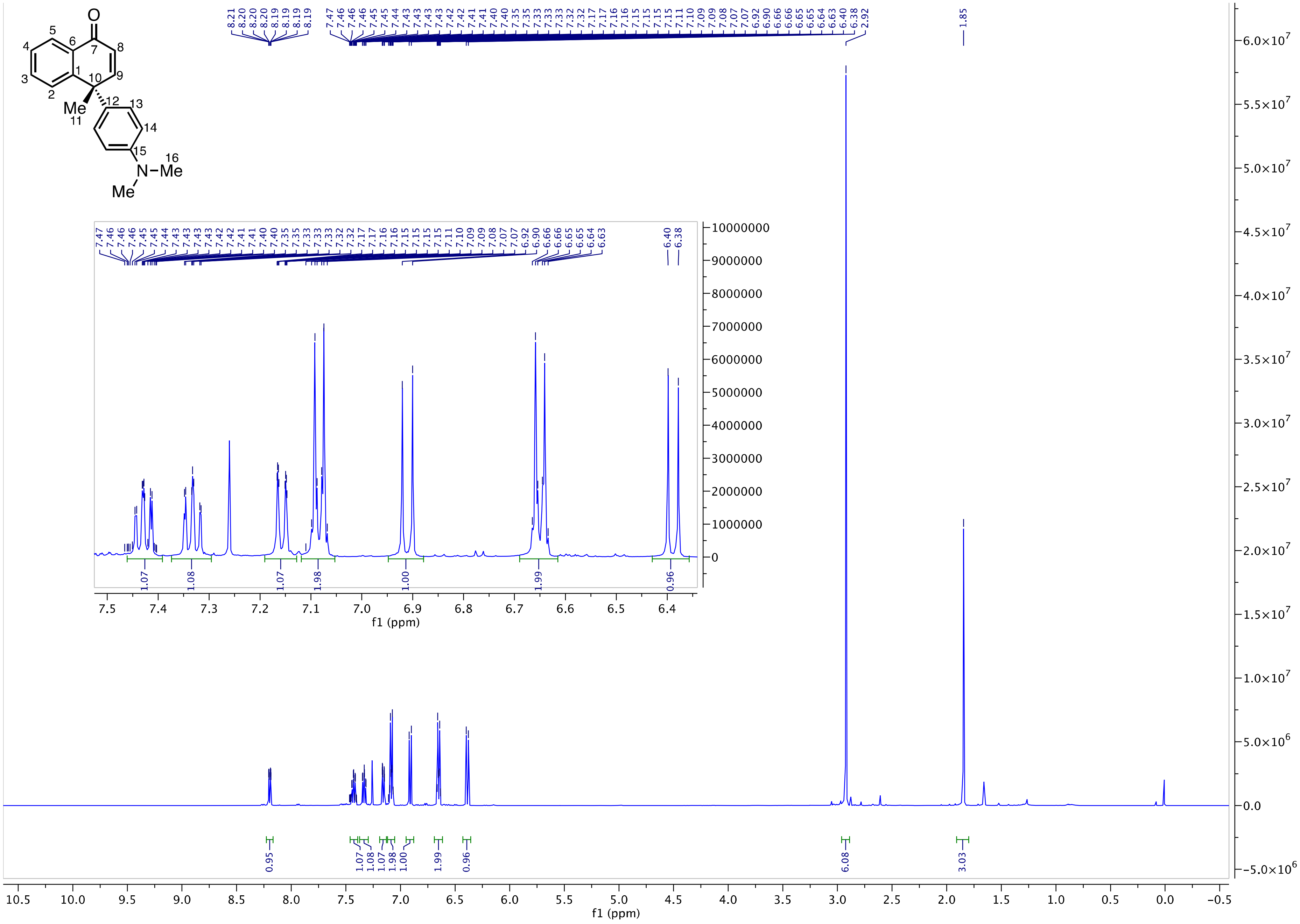

^{13}**C NMR** (CDCl$_3$): (*R*)-4-(4-(dimethylamino)phenyl)-4-Methylnaphthalen-1(4*H*)-one (**2o**)

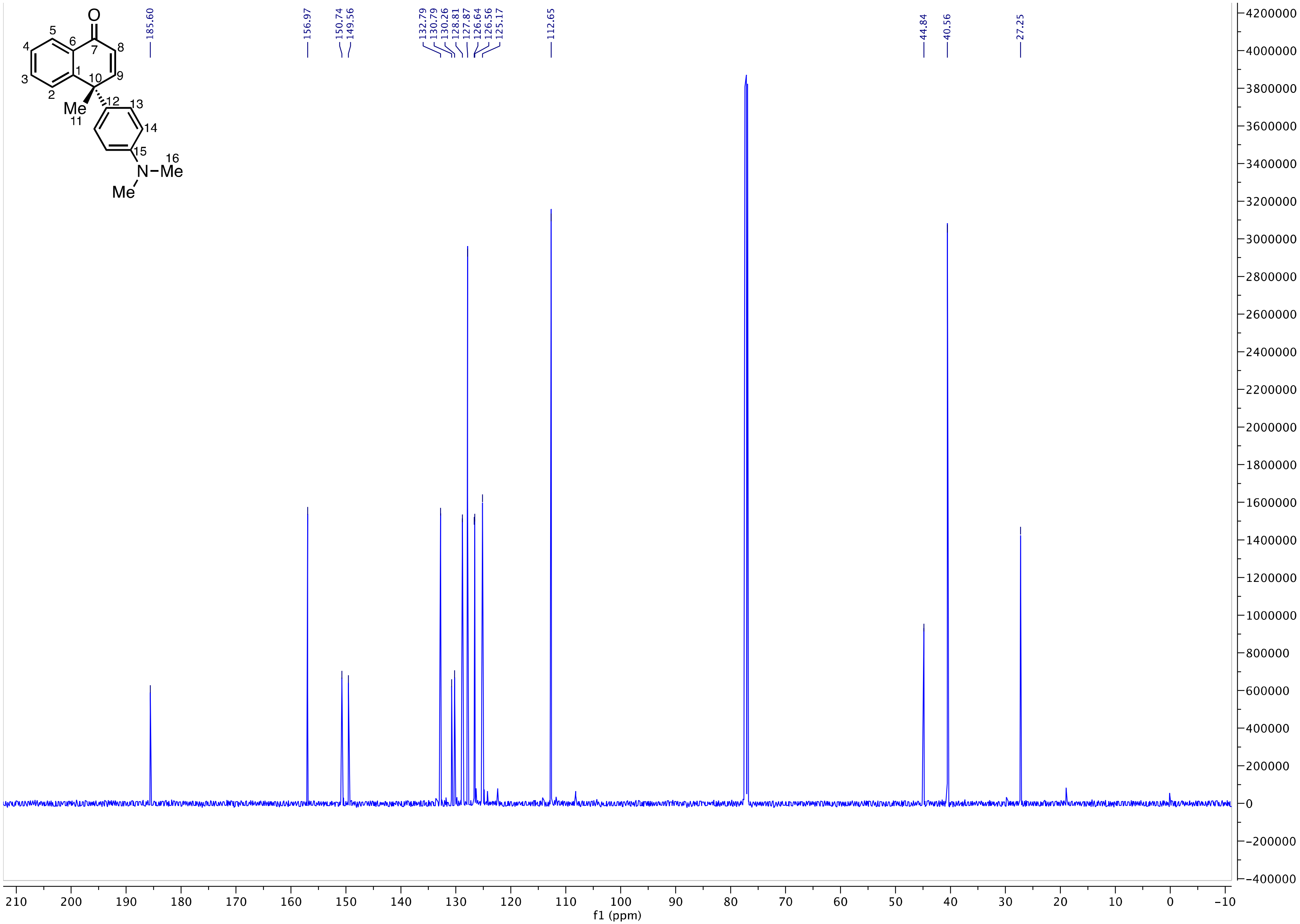

^{1}H NMR ($CDCl_3$): (*R*)-4-(3-(dibenzylamino)phenyl)-4-Methylnaphthalen-1(4*H*)-one (**2p**)

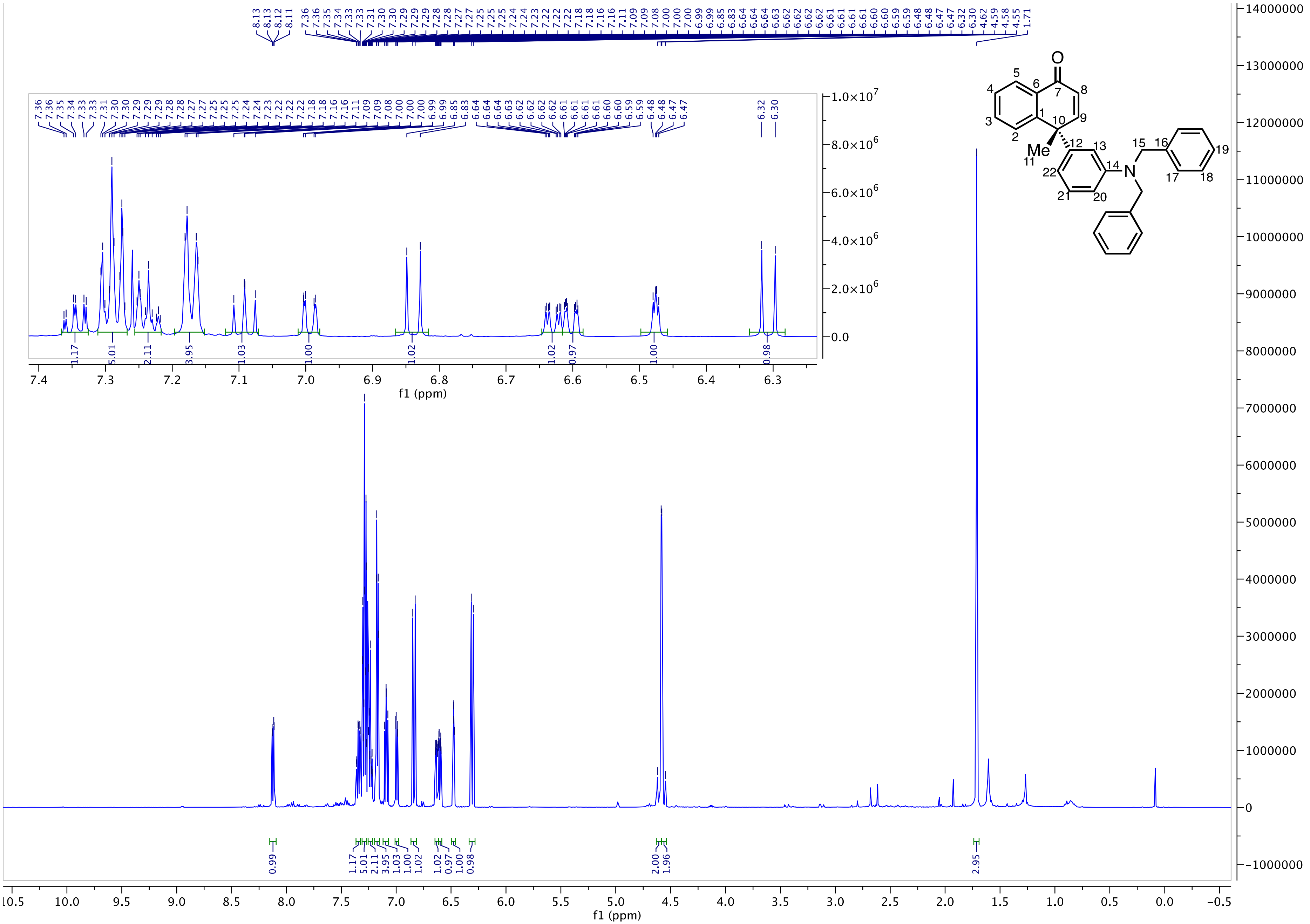

^{13}**C NMR** ($CDCl_3$): (*R*)-4-(3-(dibenzylamino)phenyl)-4-Methylnaphthalen-1(4*H*)-one (**2p**)

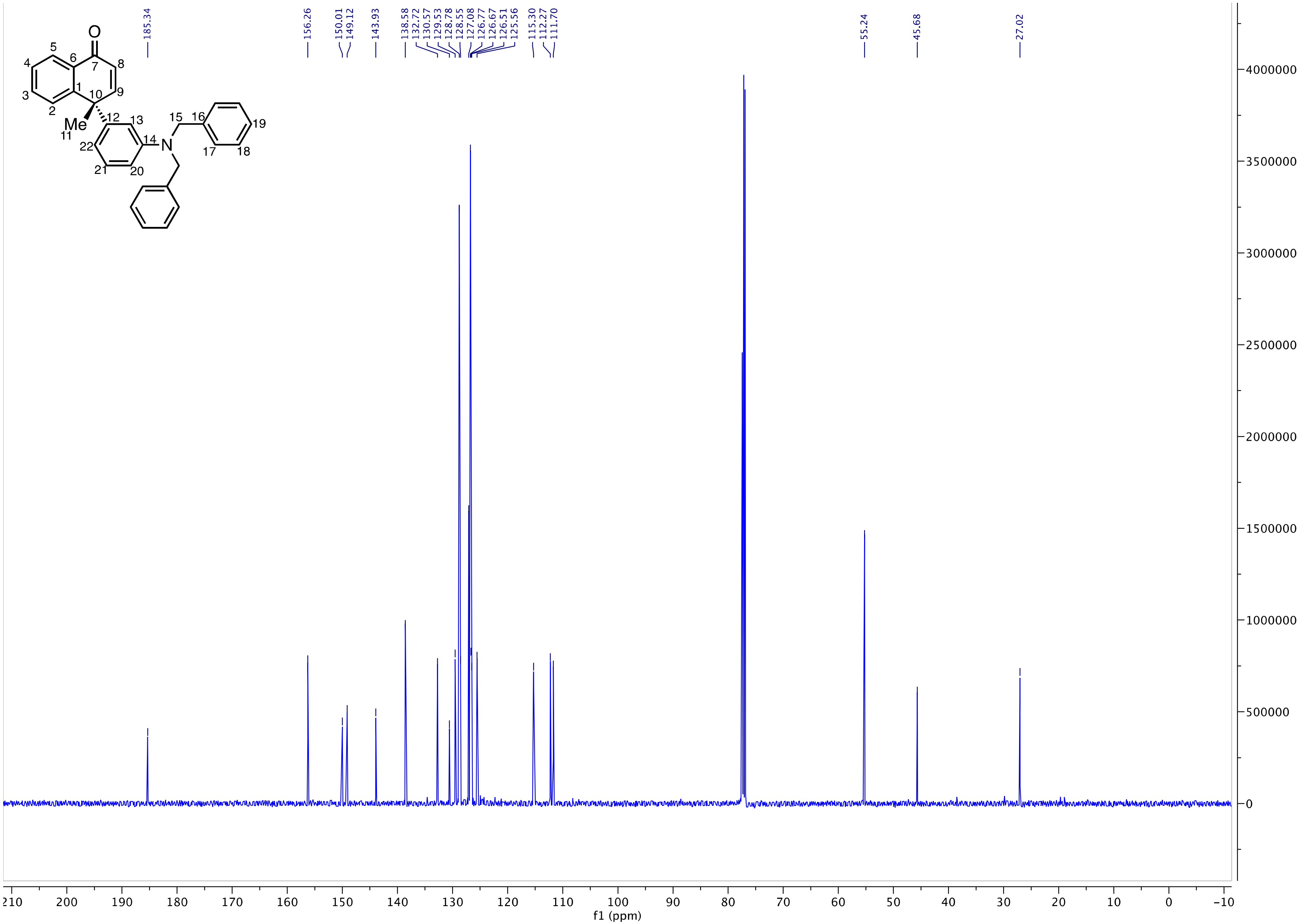

^{1}**H NMR** ($CDCl_3$): (*R*)-4-(3,5-dimethylphenyl)-4-Methylnaphthalen-1(4*H*)-one (**2q**)

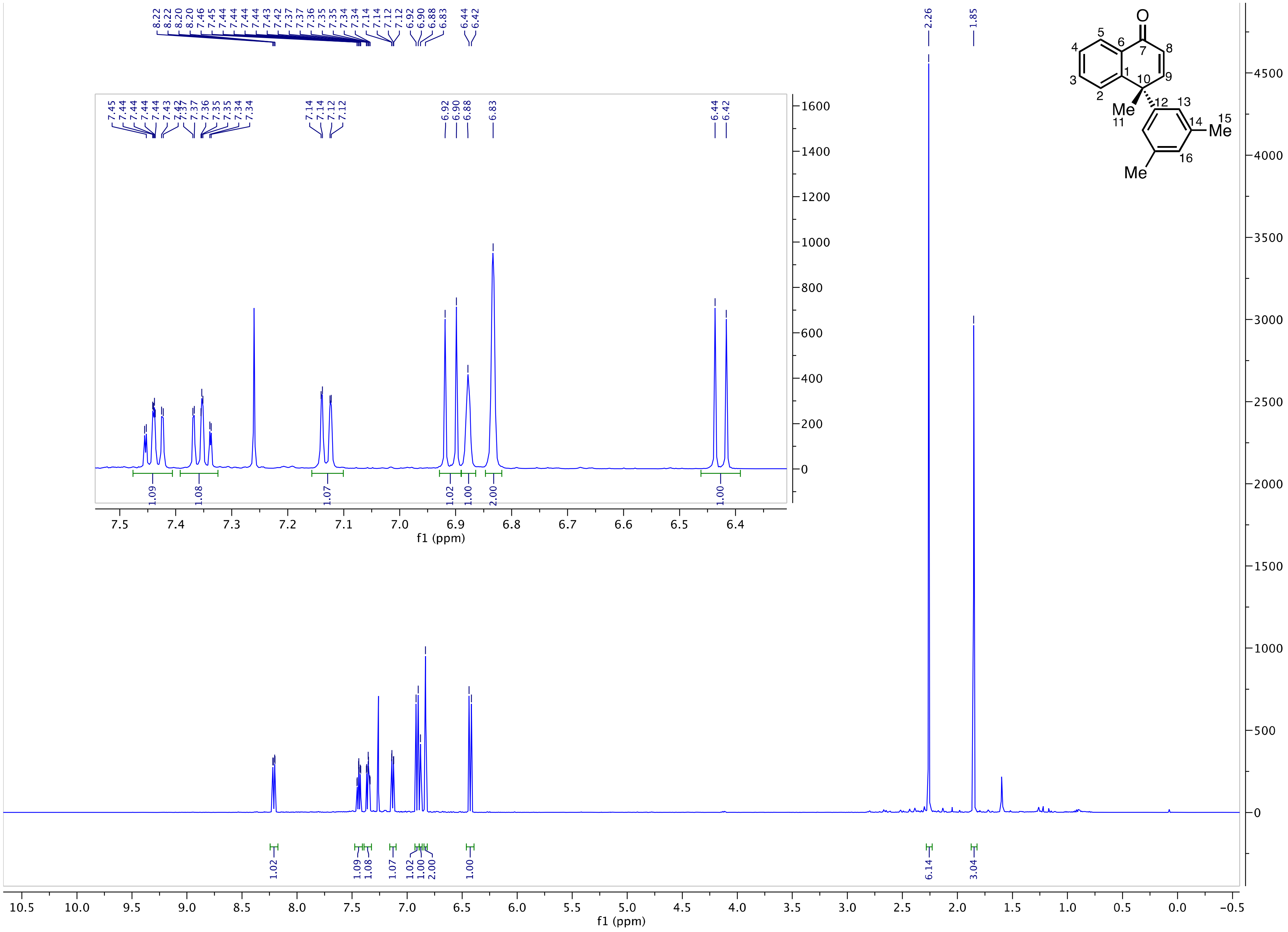

^{13}C NMR ($CDCl_3$): (*R*)-4-(3,5-dimethylphenyl)-4-Methylnaphthalen-1(4*H*)-one **(2q)**

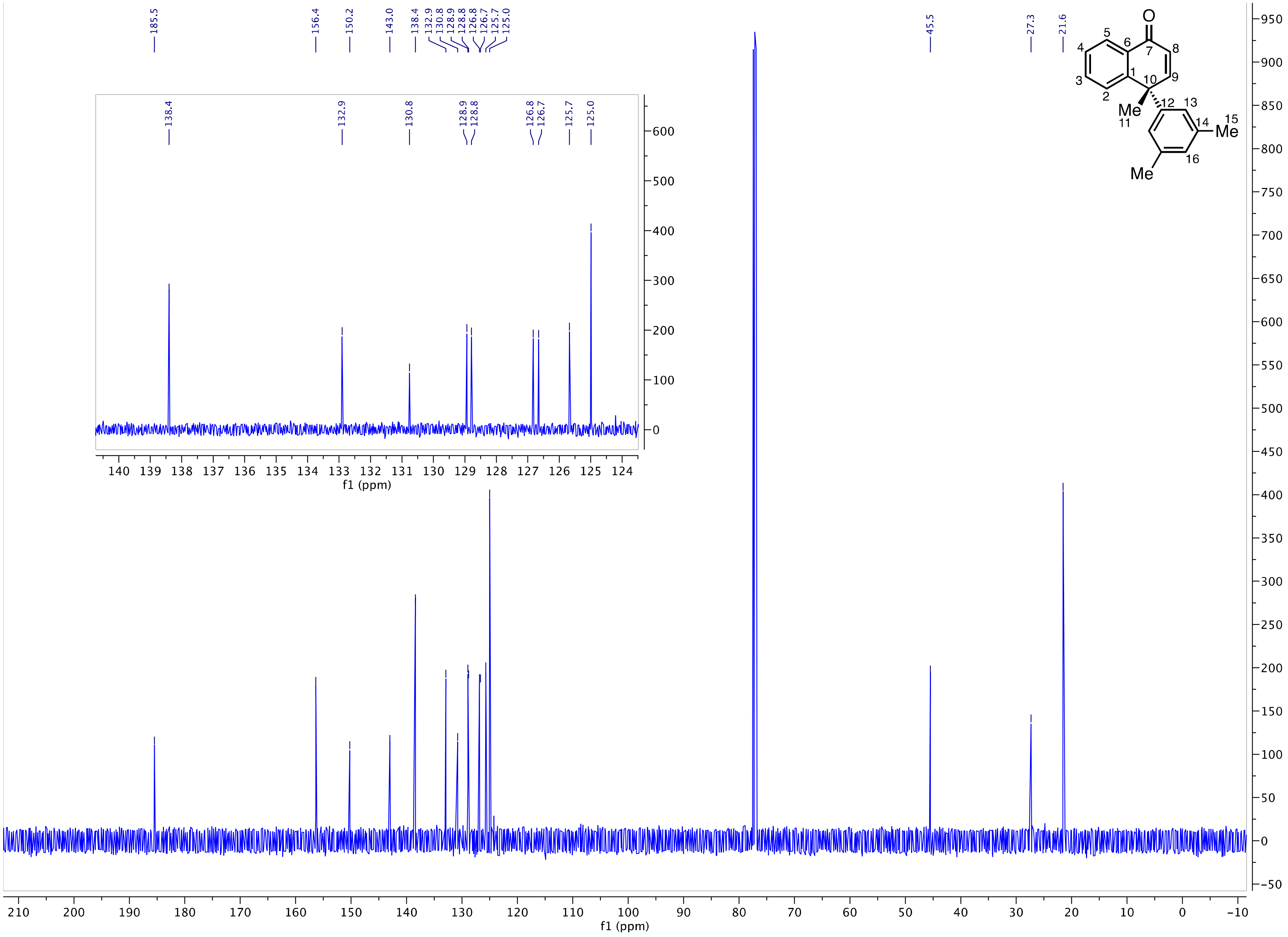

^{1}H NMR ($CDCl_3$): (*R*)-4-(3-chlorophenyl)-4-Methylnaphthalen-1(4*H*)-one (**2r**)

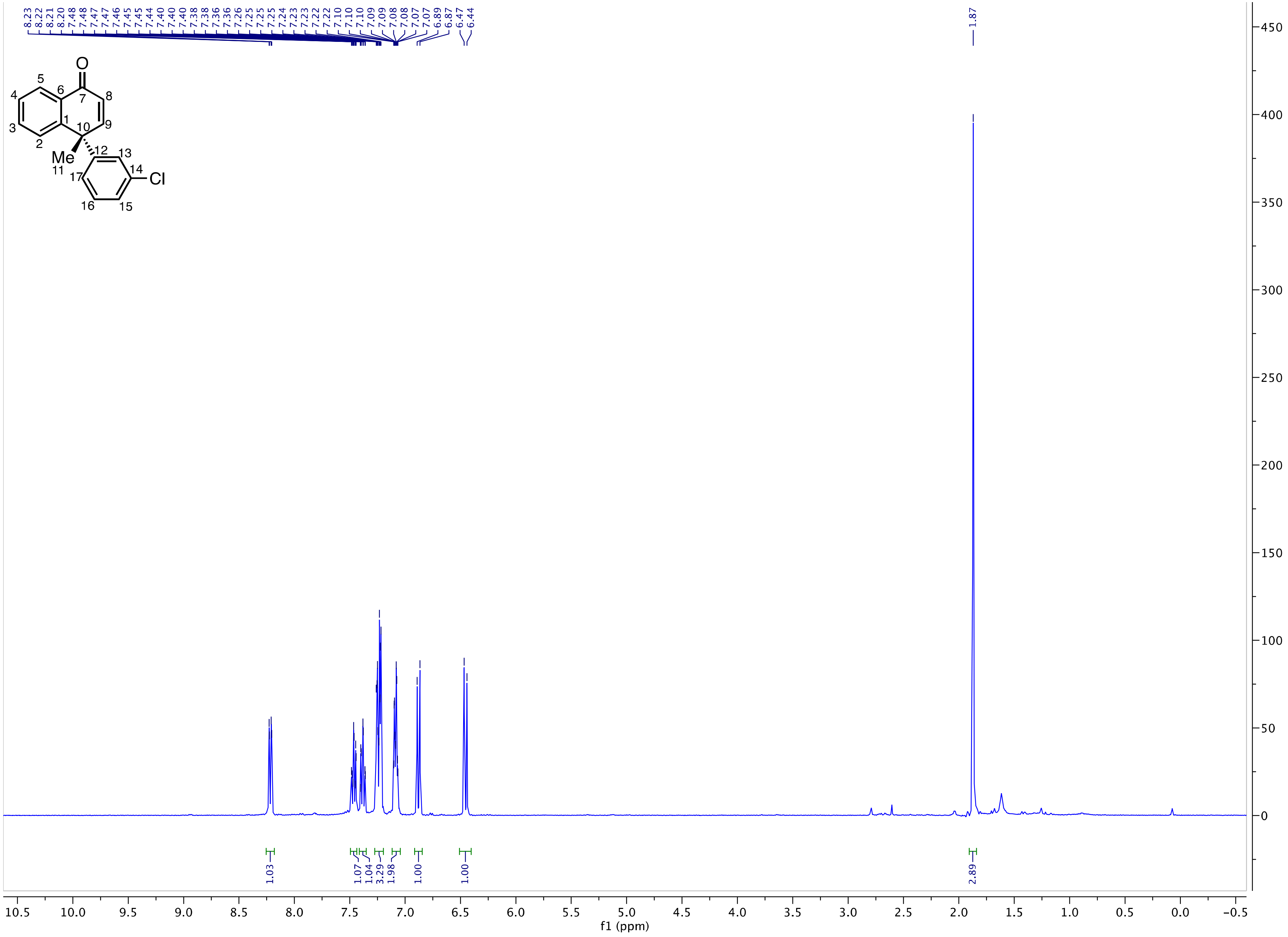

^{13}C NMR ($CDCl_3$): (*R*)-4-(3-chlorophenyl)-4-Methylnaphthalen-1(4*H*)-one (**2r**)

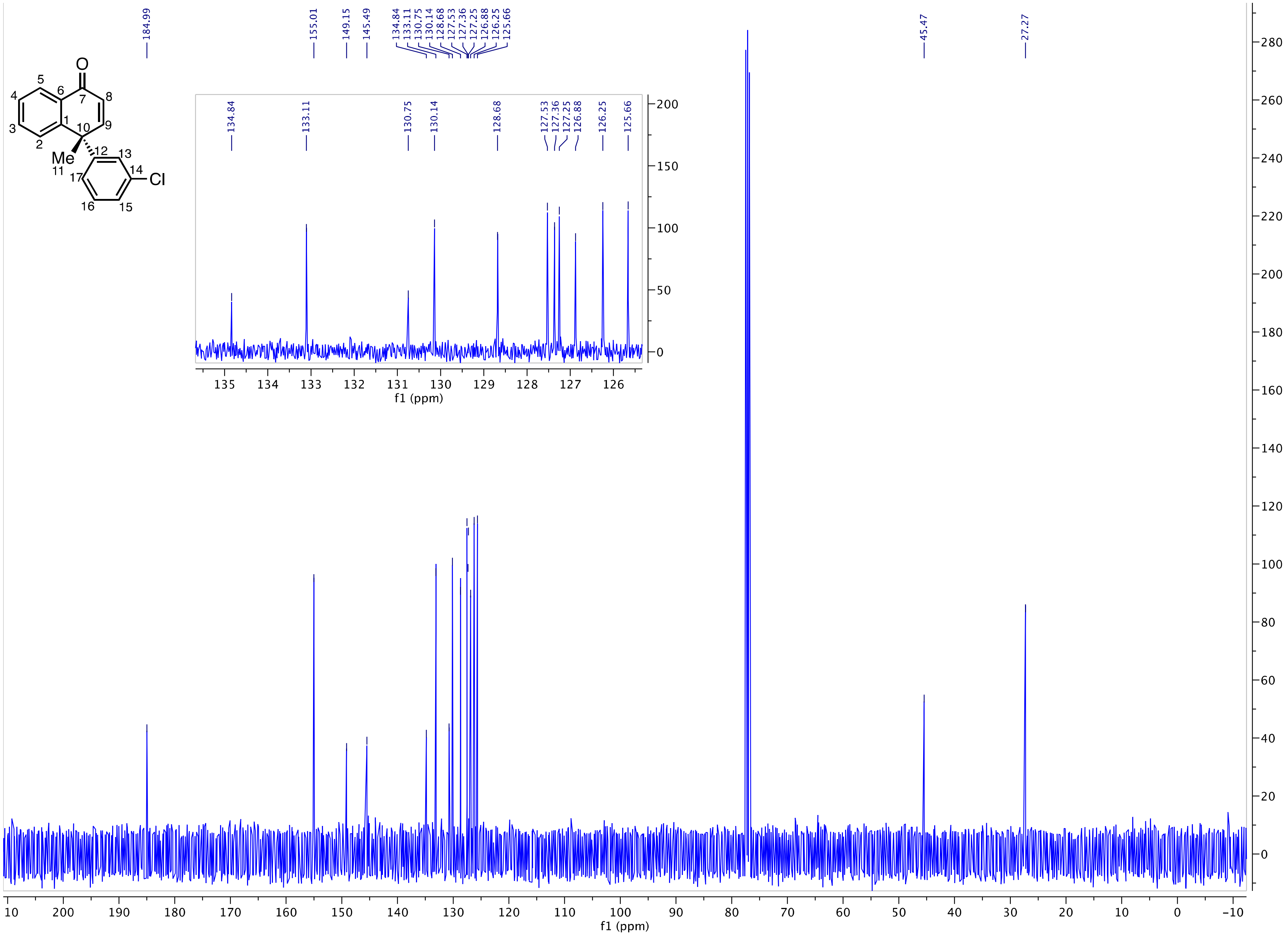

^{1}H NMR ($CDCl_3$): (*R*)-4-(3-chloro-5-fluorophenyl)-4-Methylnaphthalen-1(4*H*)-one (**2s**)

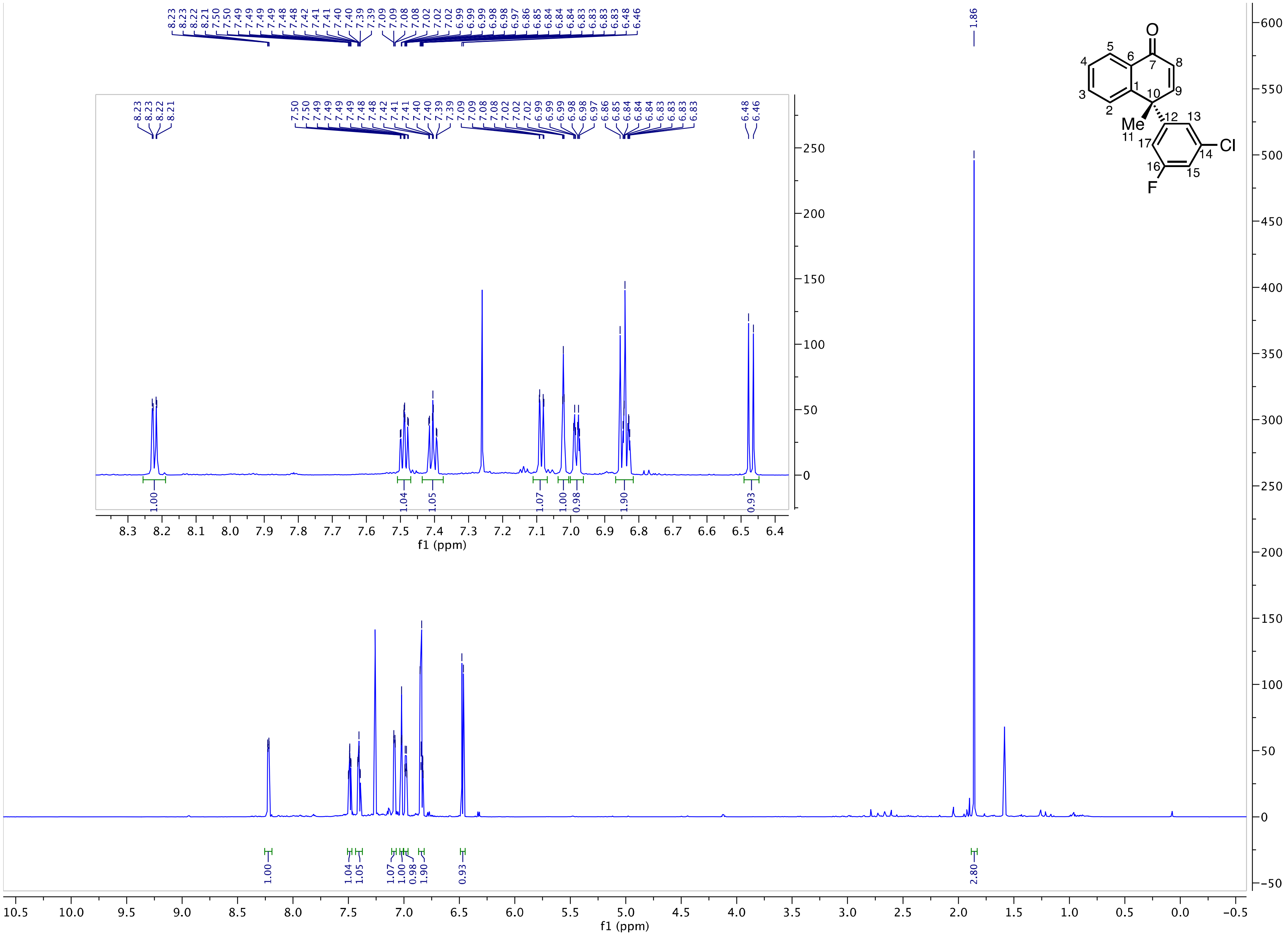

^{13}C **NMR** (CDCl$_3$): (*R*)-4-(3-chloro-5-fluorophenyl)-4-Methylnaphthalen-1(4*H*)-one (**2s**)

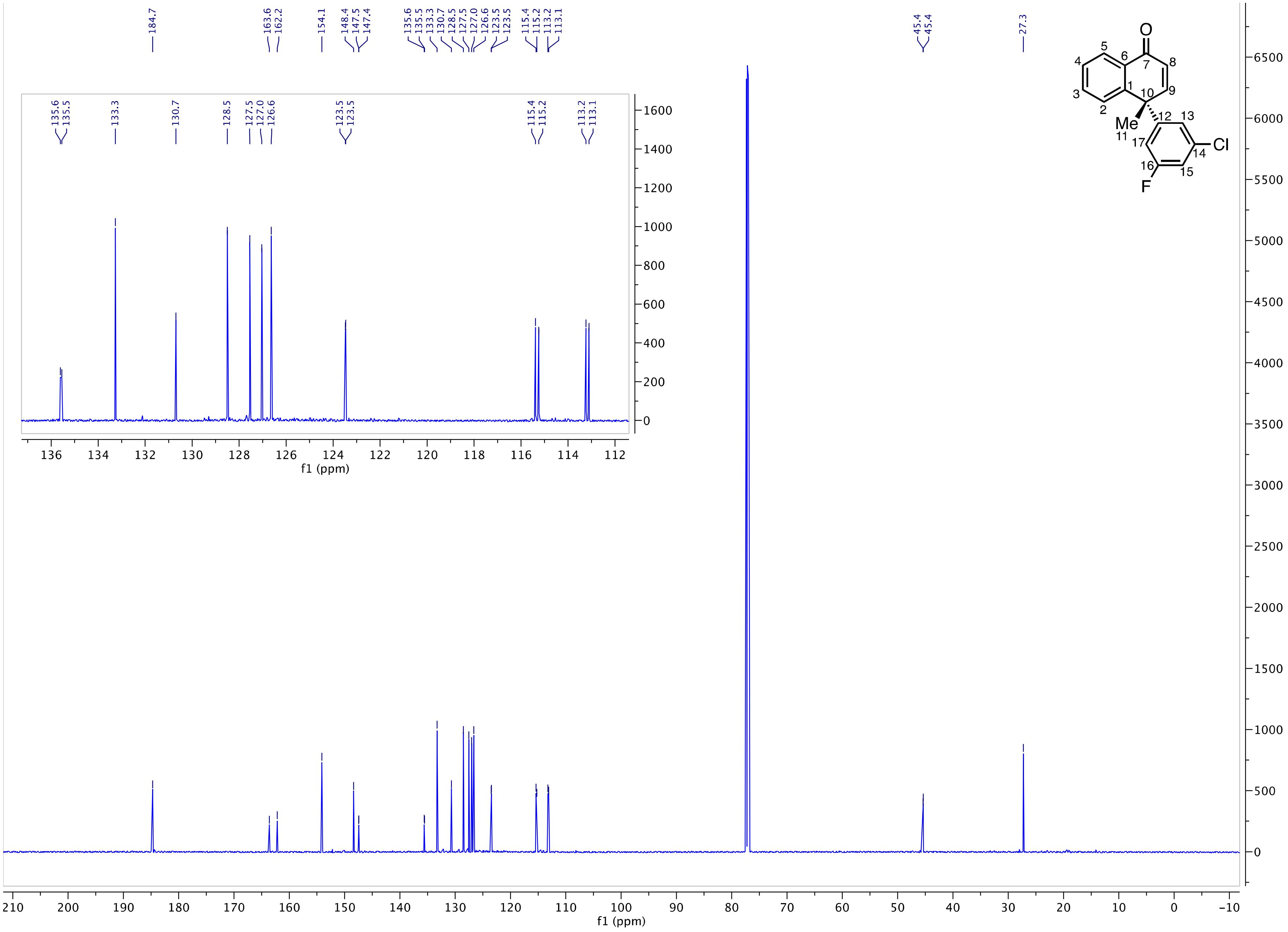

^{19}**F NMR** (CDCl$_3$): (*R*)-4-(3-chloro-5-fluorophenyl)-4-Methylnaphthalen-1(4*H*)-one (**2s**)

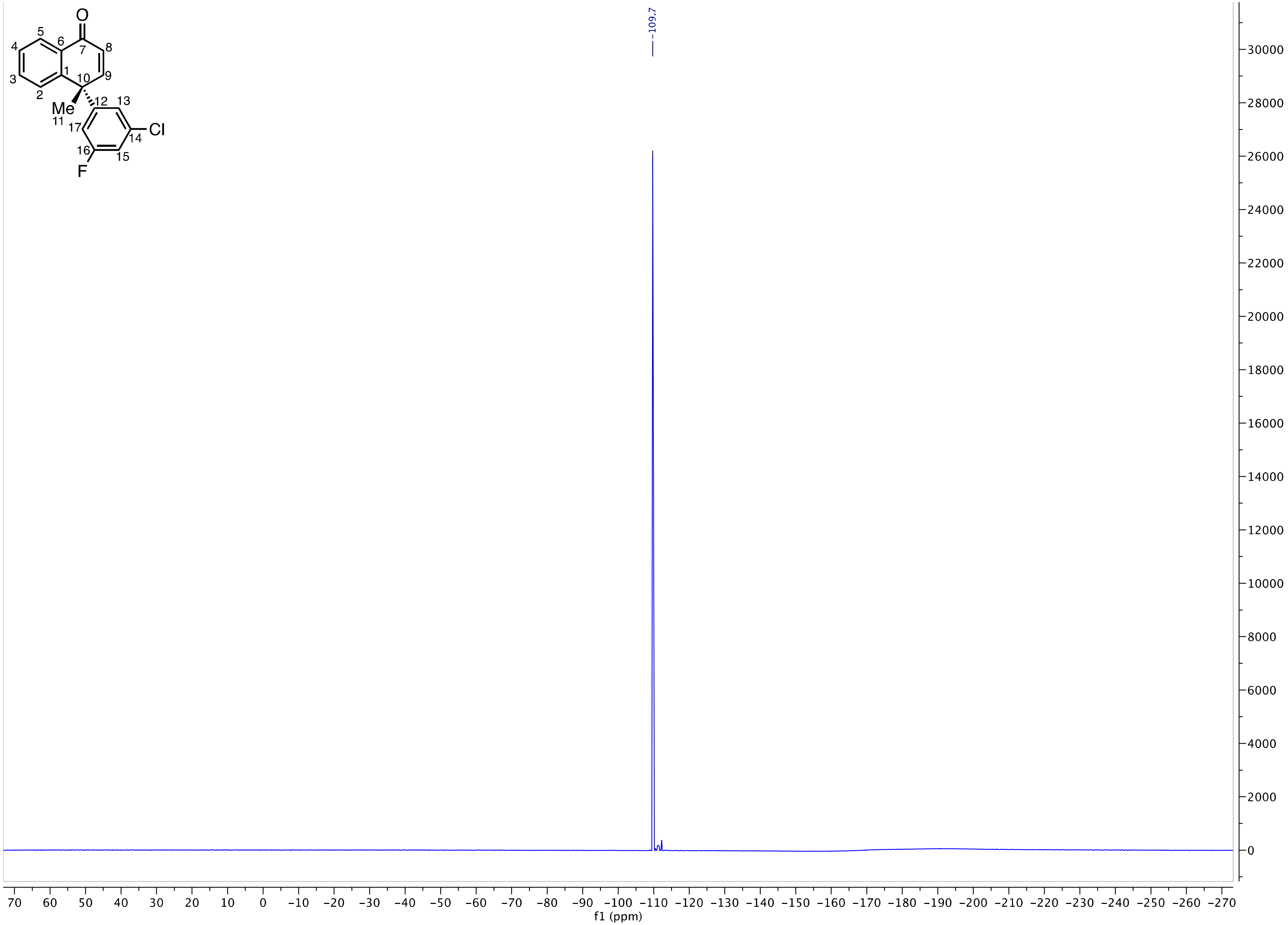

^{1}H NMR ($CDCl_3$): Ethyl (*R*)-4-(1-methyl-4-oxo-1,4-dihydronaphthalen-1-yl)benzoate (**2t**)

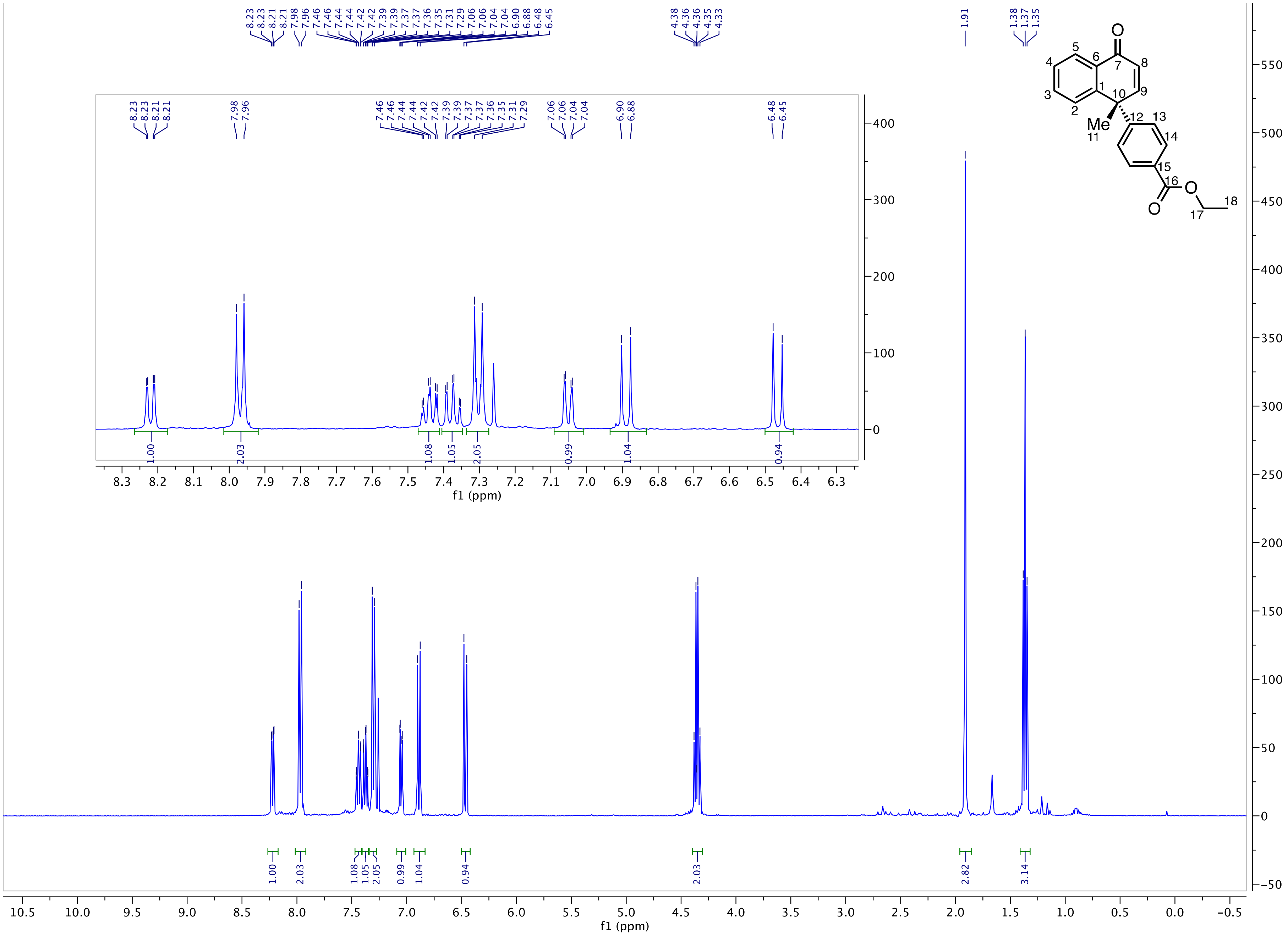

^{13}**C NMR** ($CDCl_3$): Ethyl (*R*)-4-(1-methyl-4-oxo-1,4-dihydronaphthalen-1-yl)benzoate (**2t**)

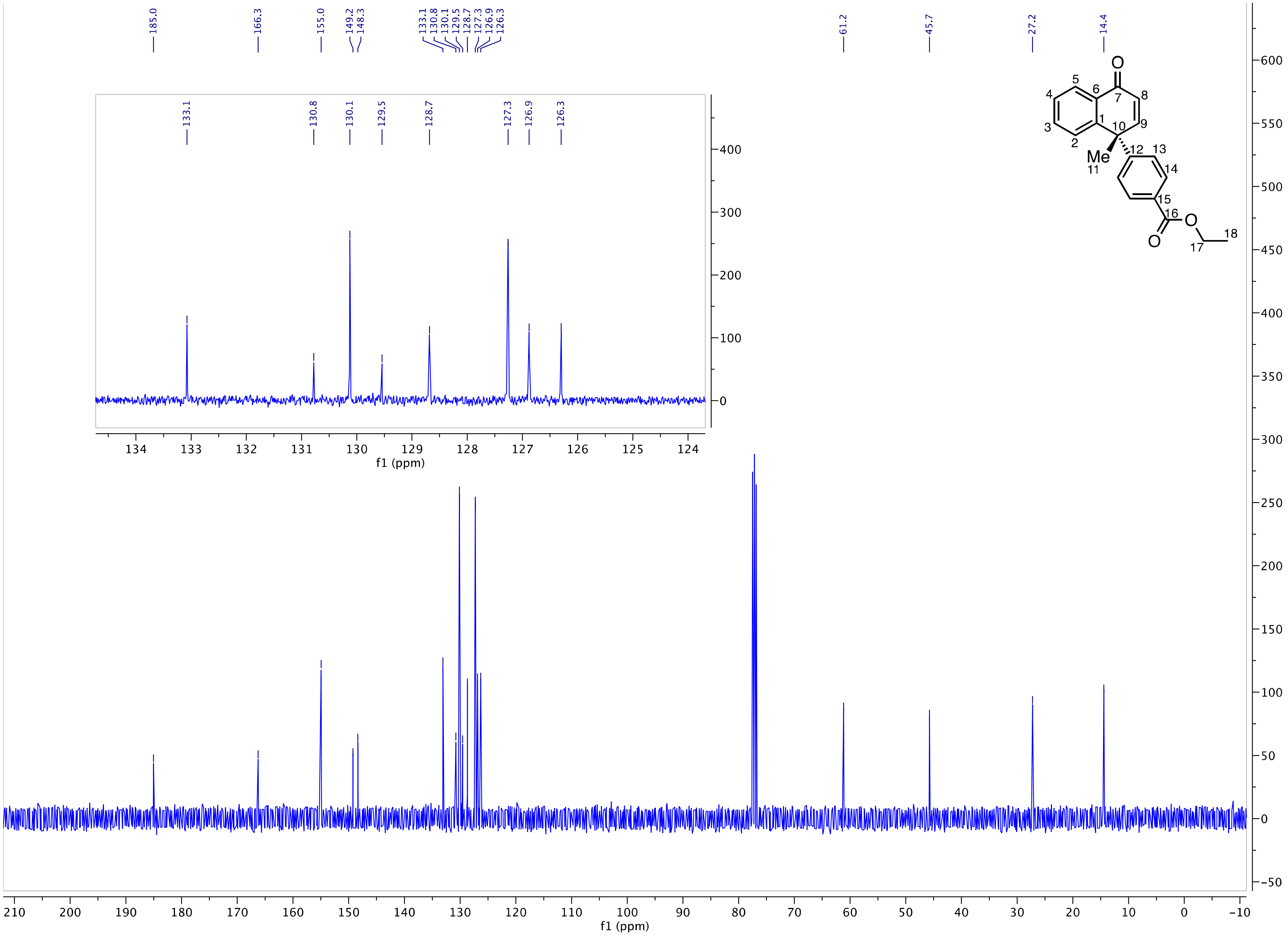

^{1}H NMR ($CDCl_3$): (*R*)-4-(1-methyl-4-oxo-1,4-dihydronaphthalen-1-yl)Benzonitrile (**2u**)

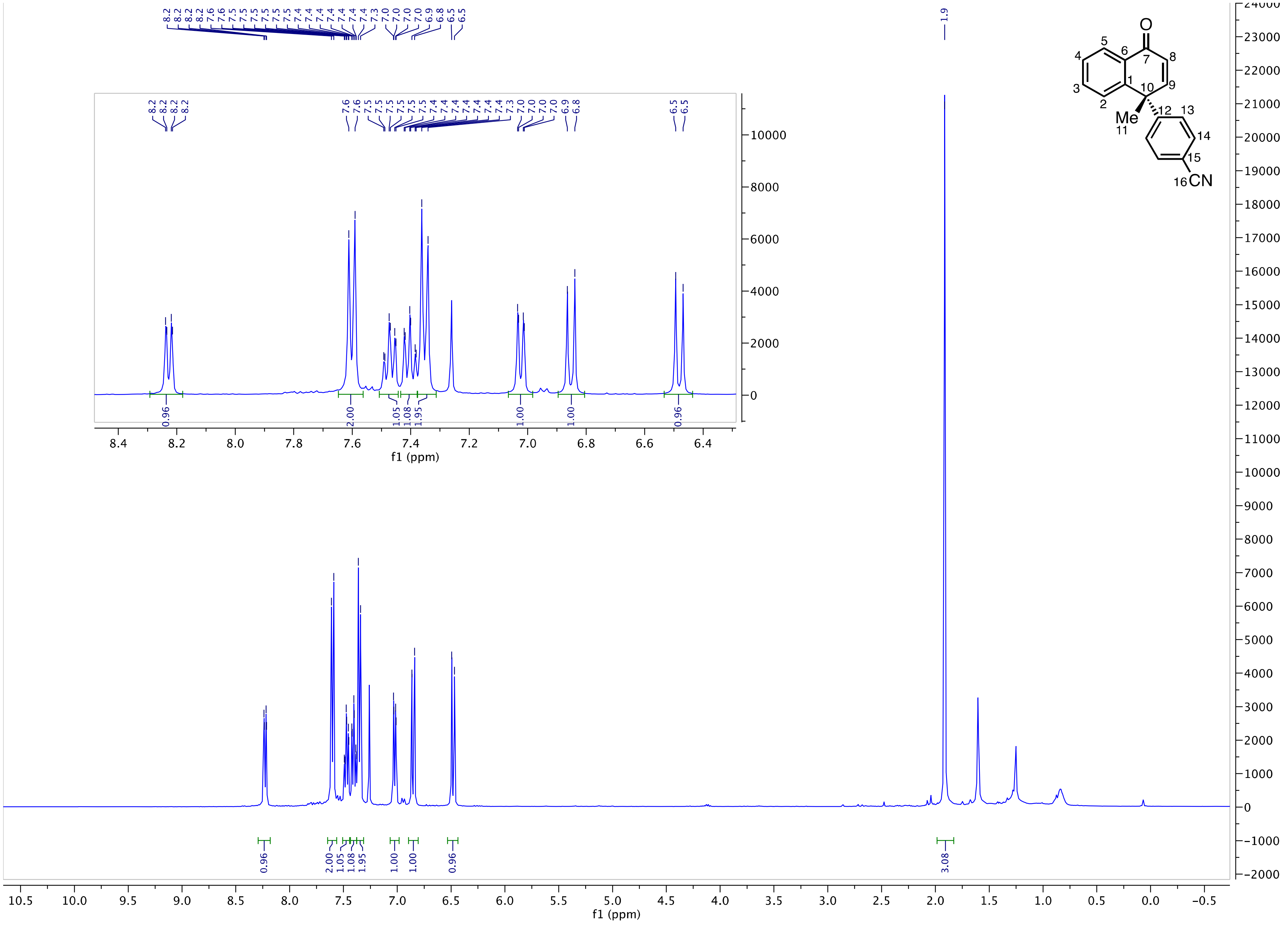

^{13}C NMR ($CDCl_3$): (*R*)-4-(1-methyl-4-oxo-1,4-dihydronaphthalen-1-yl)Benzonitrile (**2u**)

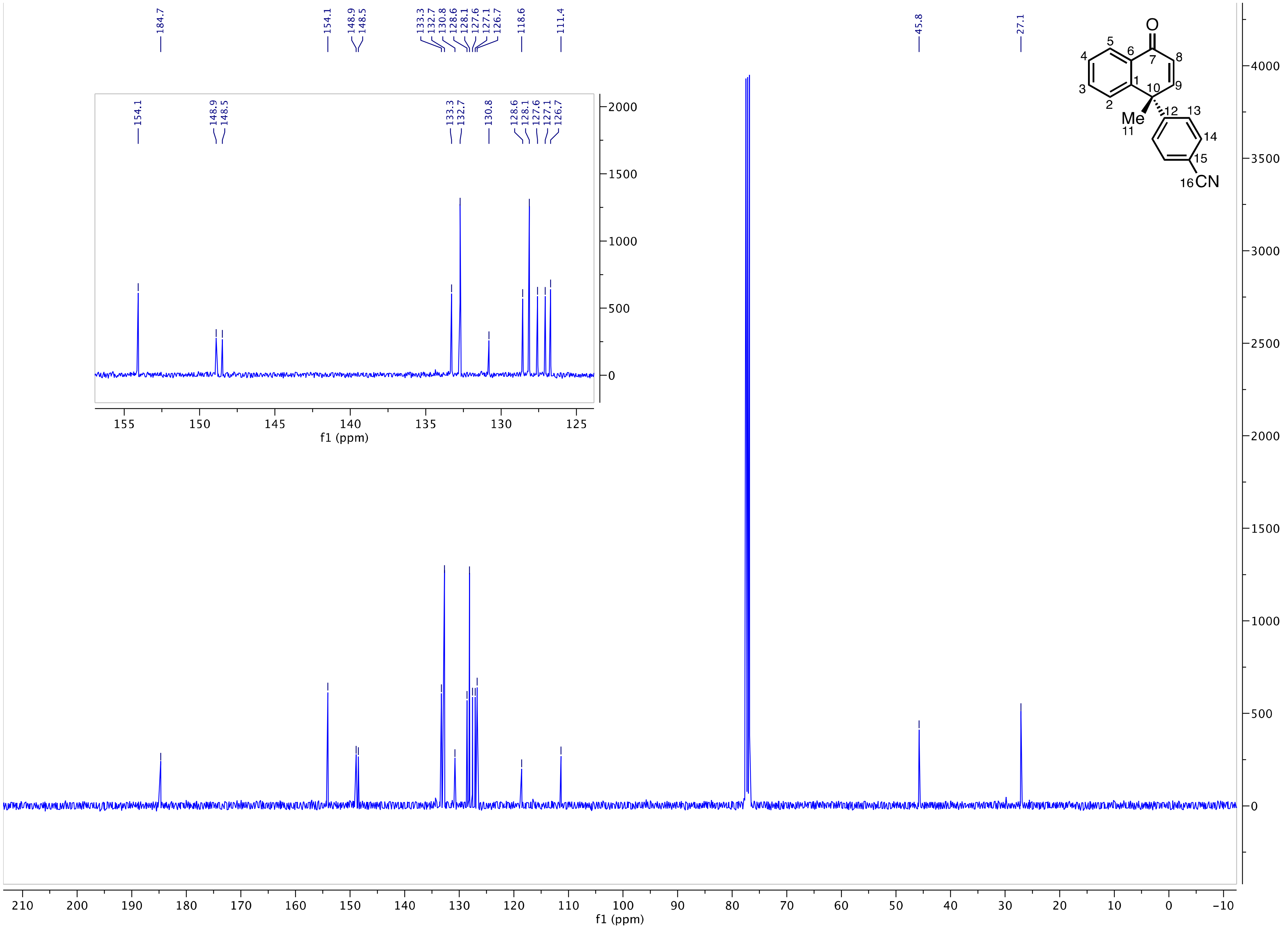

^{1}H NMR (CDCl$_3$): (*R*)-4-Methyl-4-(4-nitrophenyl)naphthalen-1(4*H*)-one (**2v**)

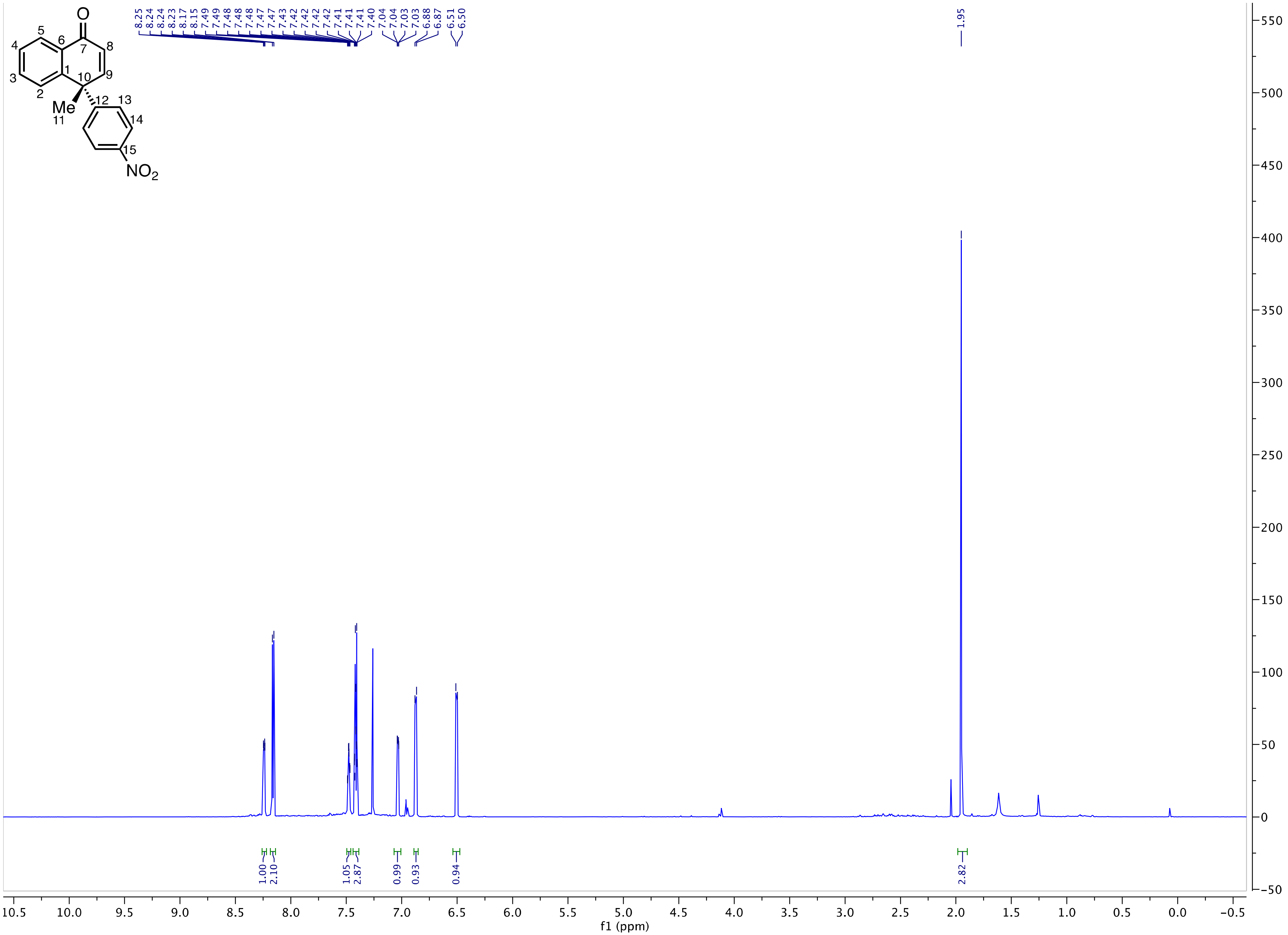

^{13}C NMR ($CDCl_3$): (*R*)-4-Methyl-4-(4-nitrophenyl)naphthalen-1(4*H*)-one (**2v**)

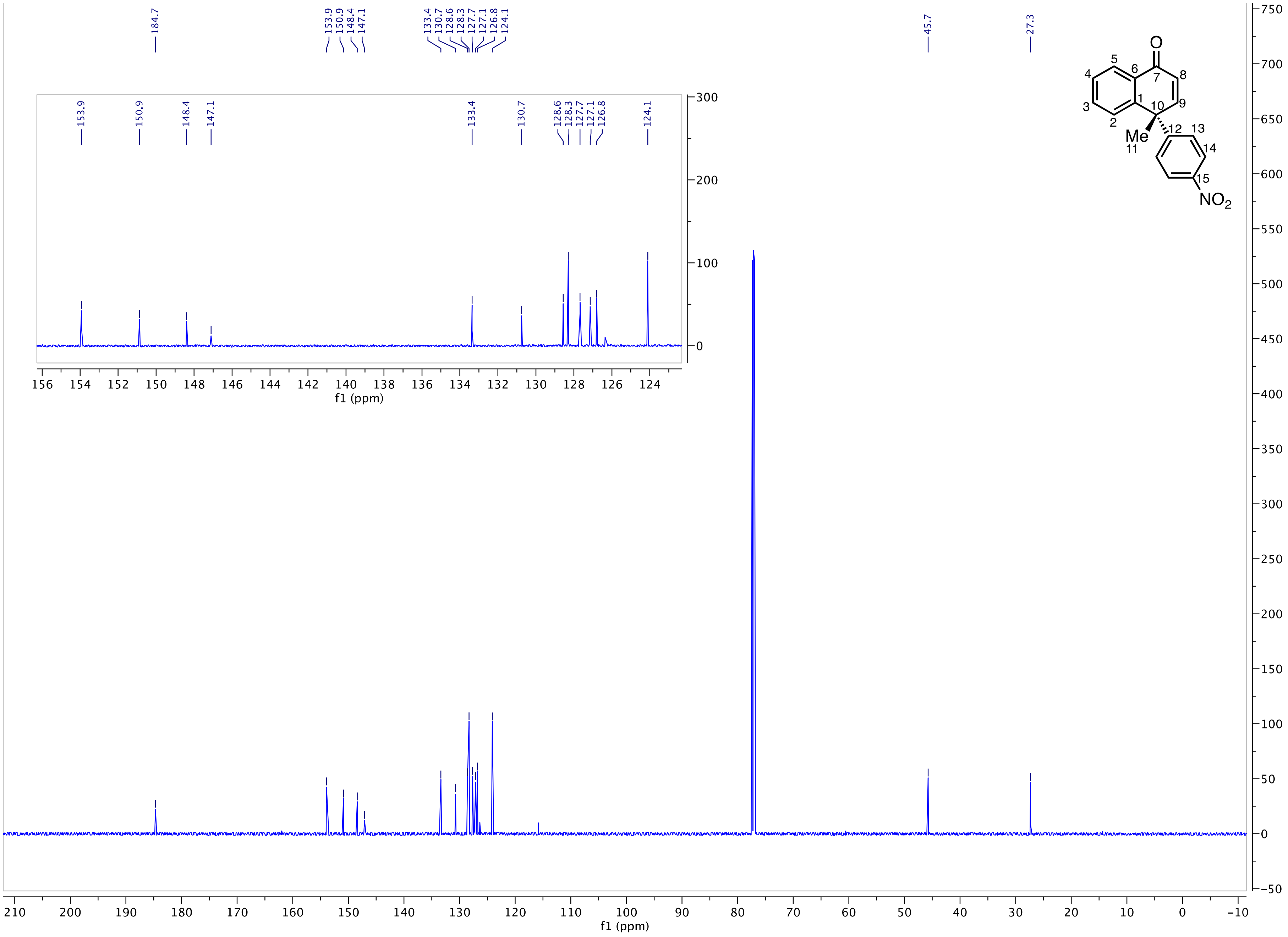

^{1}H NMR ($CDCl_3$): (*R*)-4-Methyl-4-(4-(trifluoromethyl)phenyl)naphthalen-1(4*H*)-one (**2w**)

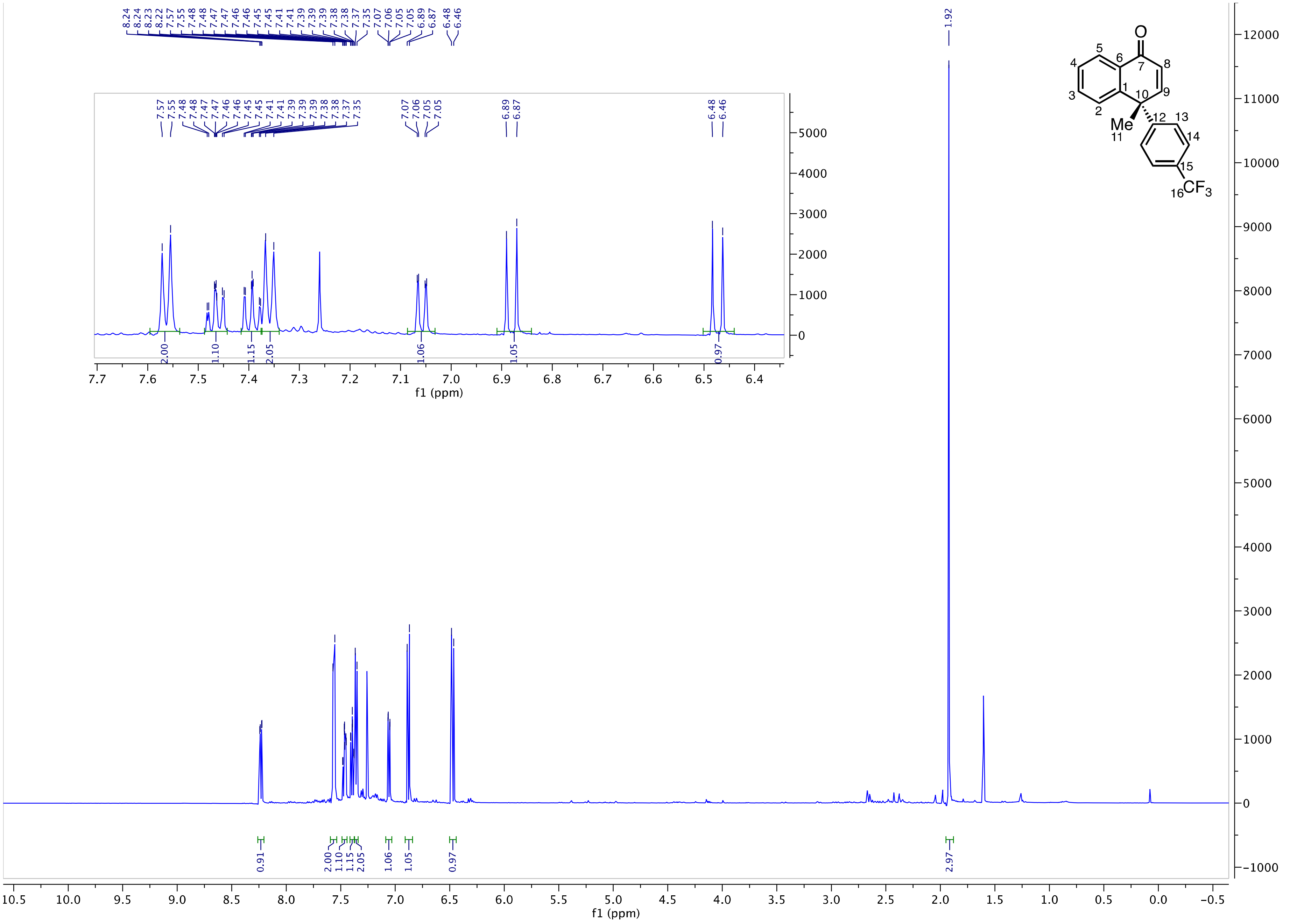

^{13}C NMR ($CDCl_3$): (*R*)-4-Methyl-4-(4-(trifluoromethyl)phenyl)naphthalen-1(4*H*)-one (**2w**)

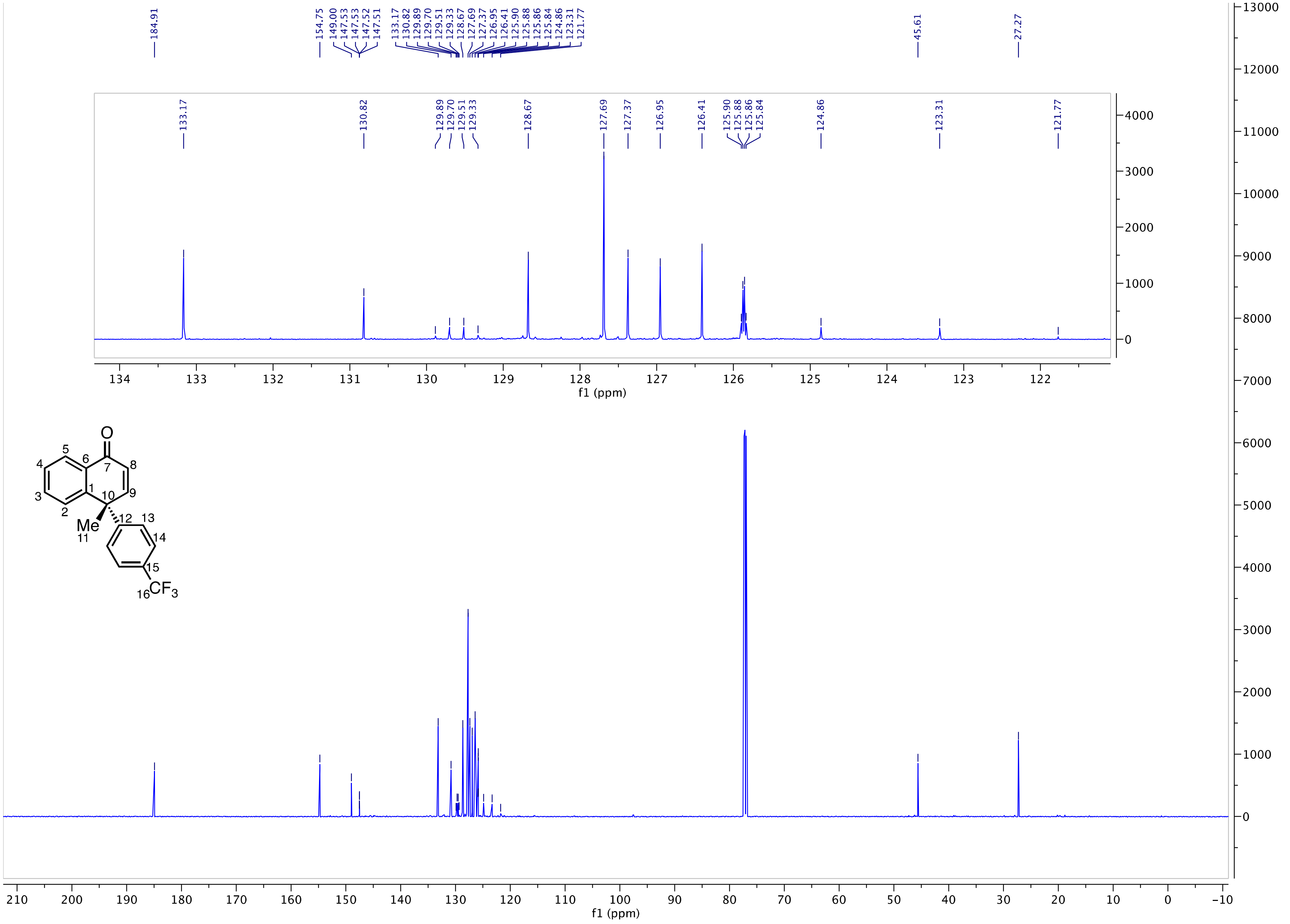

^{19}F NMR ($CDCl_3$): (*R*)-4-Methyl-4-(4-(trifluoromethyl)phenyl)naphthalen-1(4*H*)-one (**2w**)

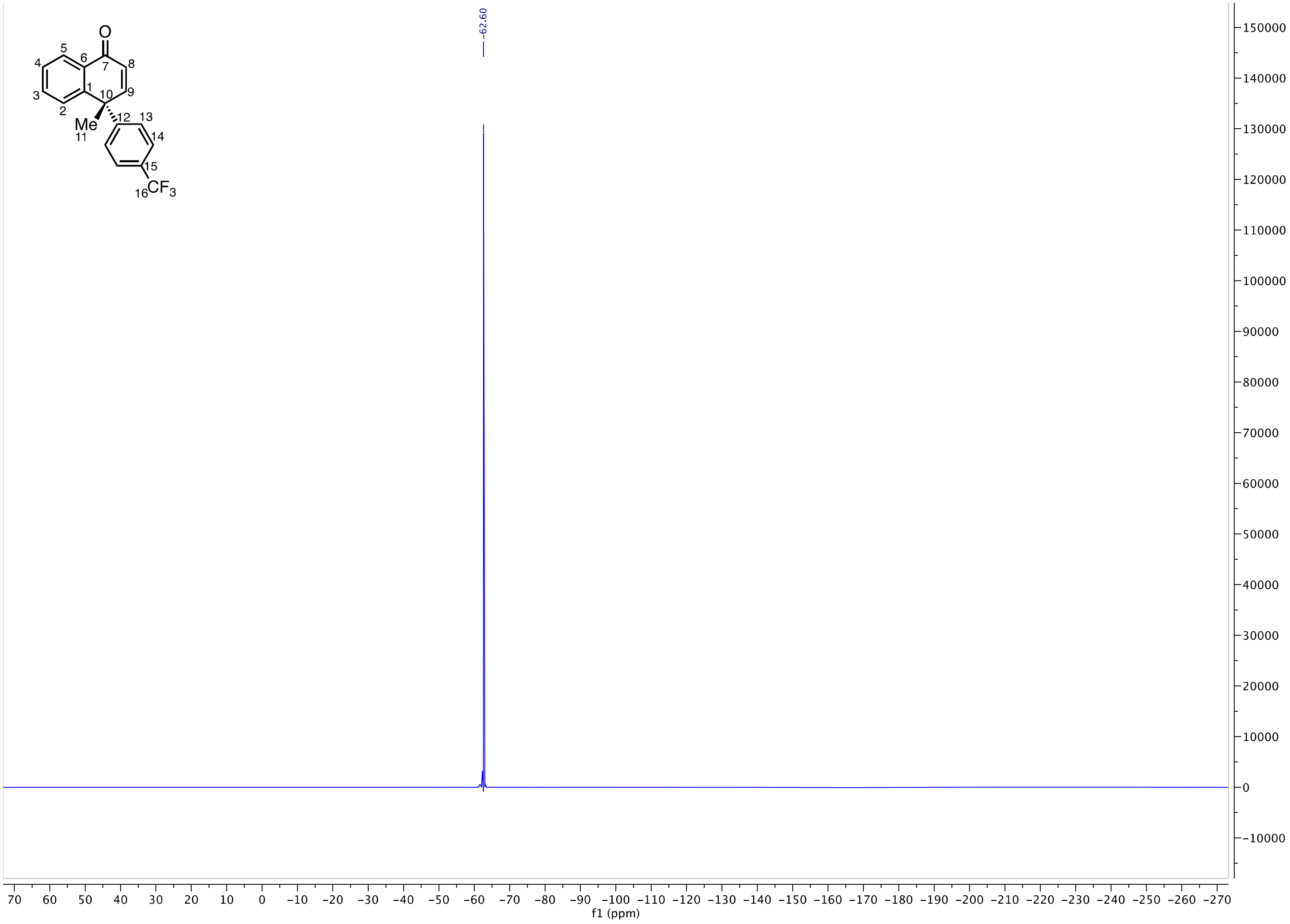

^{1}H NMR (CDCl$_3$): (*R*)-4-([1,1'-biphenyl]-4-yl)-4-Methylnaphthalen-1(4*H*)-one (**2x**)

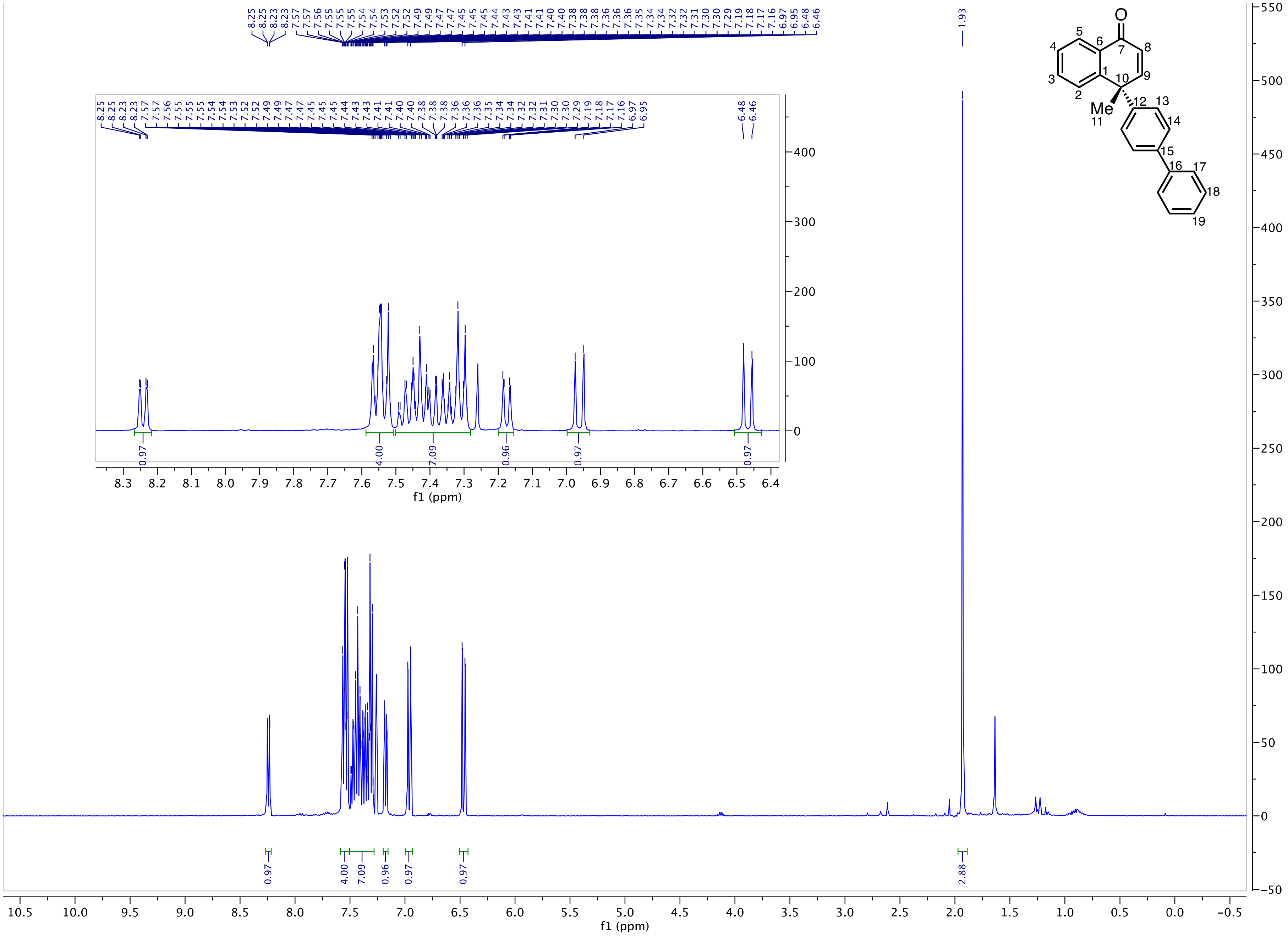

^{13}C NMR ($CDCl_3$): (*R*)-4-([1,1'-biphenyl]-4-yl)-4-Methylnaphthalen-1(4*H*)-one (**2x**)

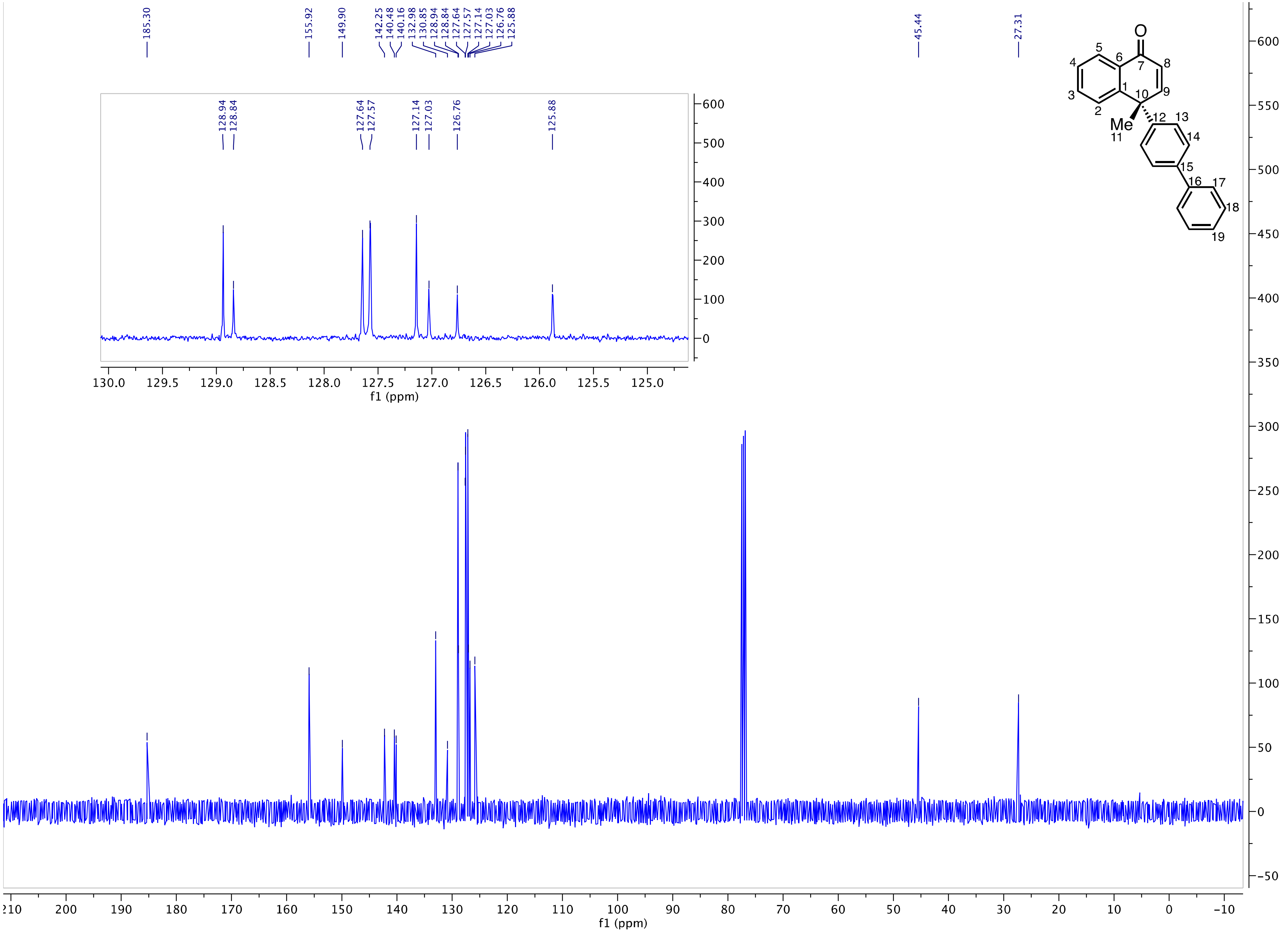

^{1}H NMR ($CDCl_3$): (*R*)-1-Methyl-[1,2'-binaphthalen]-4(1*H*)-one (**2y**)

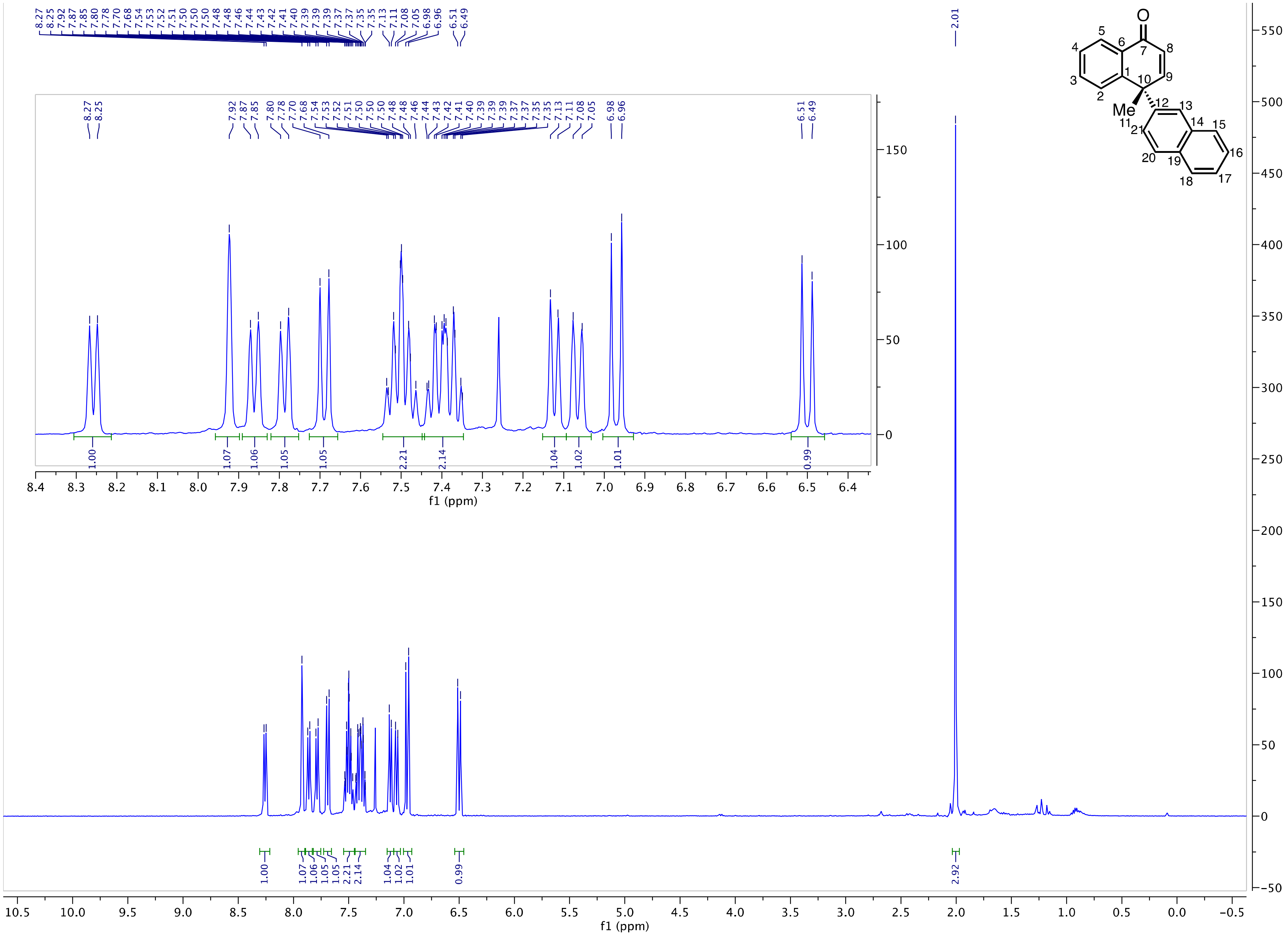

^{13}C NMR (CDCl$_3$): (*R*)-1-Methyl-[1,2'-binaphthalen]-4(1*H*)-one (**2y**)

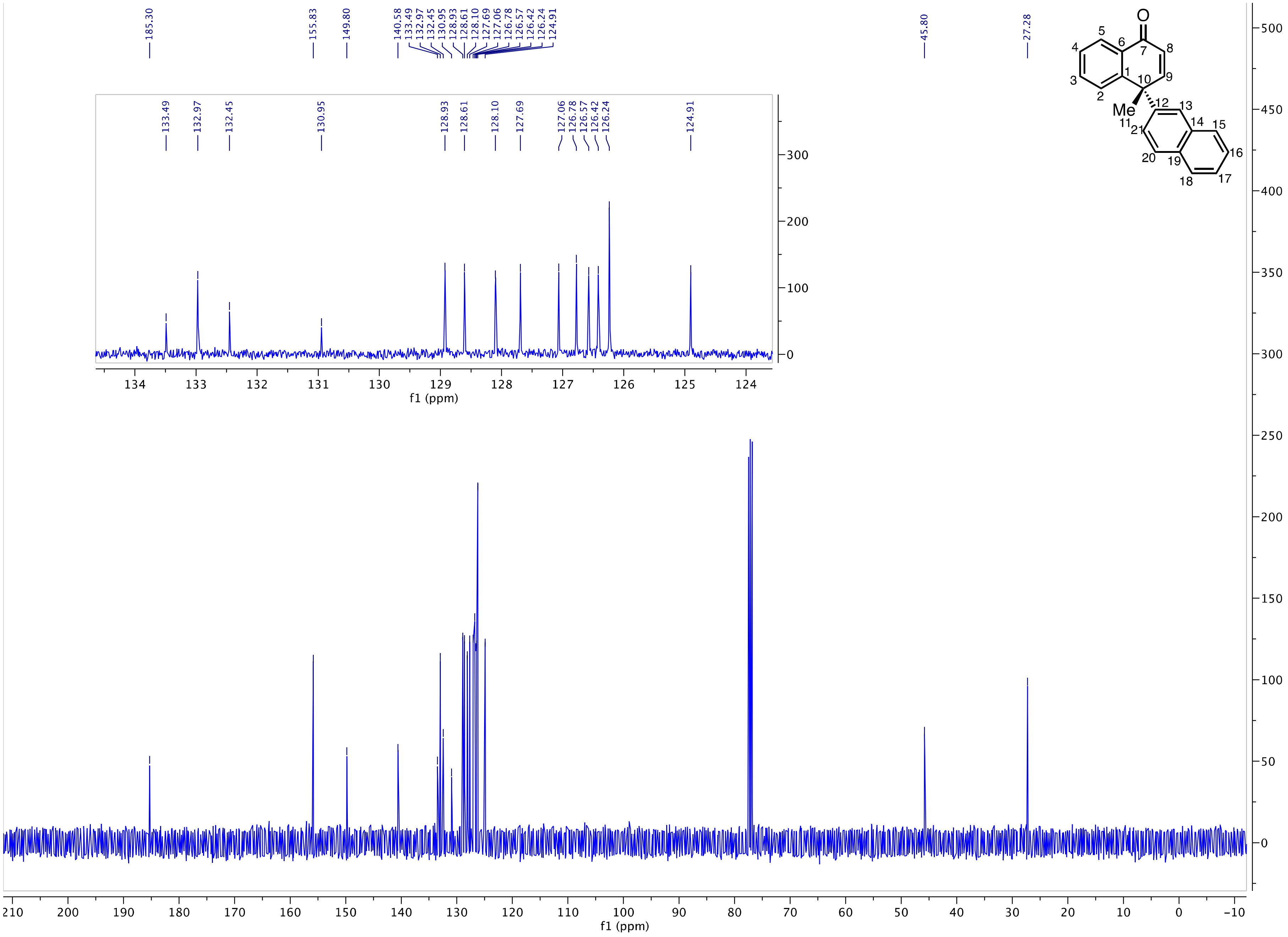

^{1}**H NMR** (CDCl$_3$): (*R*)-4-(3-(*tert*-butyl)phenyl)-4-Methylnaphthalen-1(4*H*)-one (**2z**)

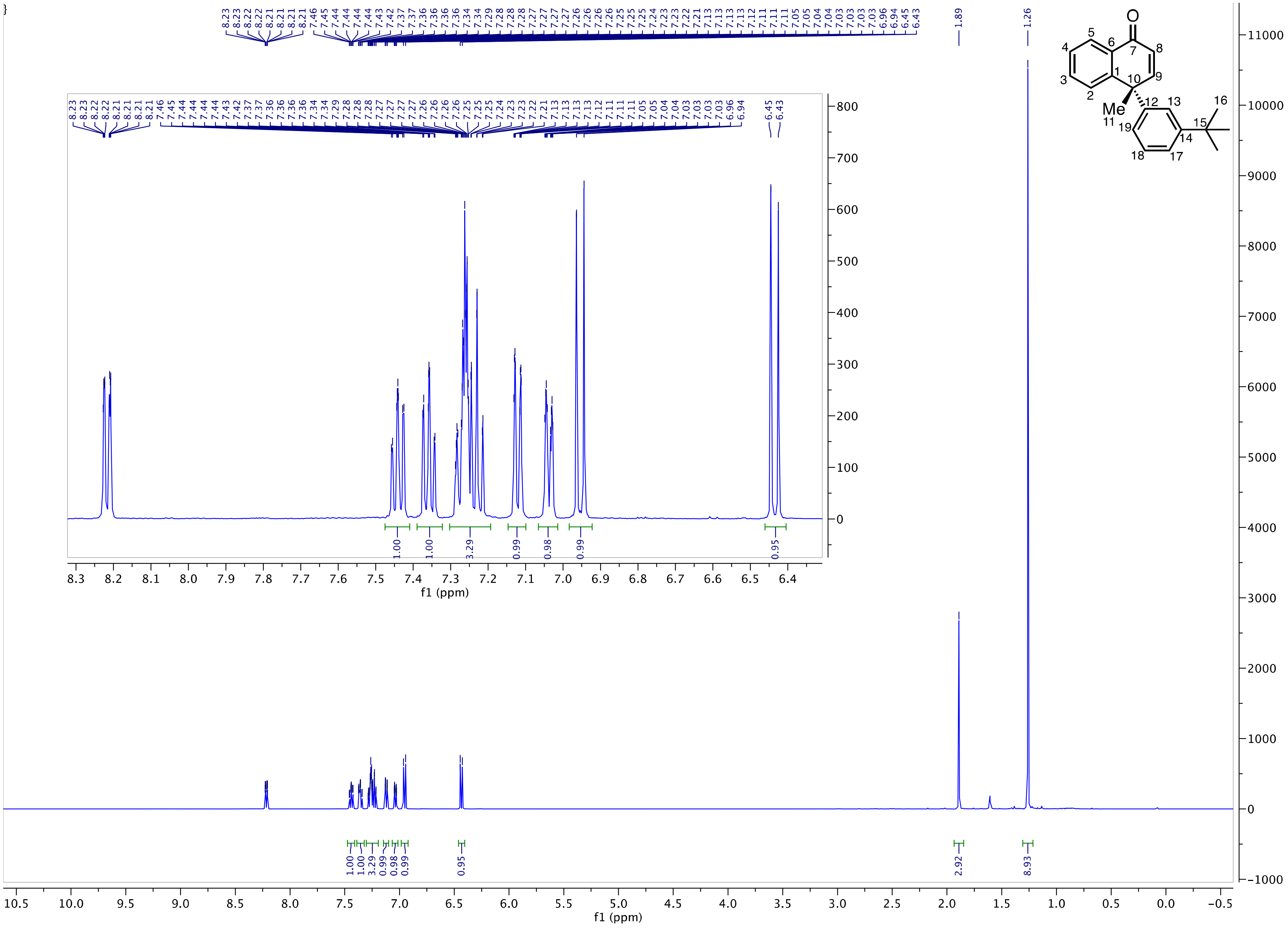

^{13}C NMR (CDCl$_3$): (*R*)-4-(3-(*tert*-butyl)phenyl)-4-Methylnaphthalen-1(4*H*)-one (**2z**)

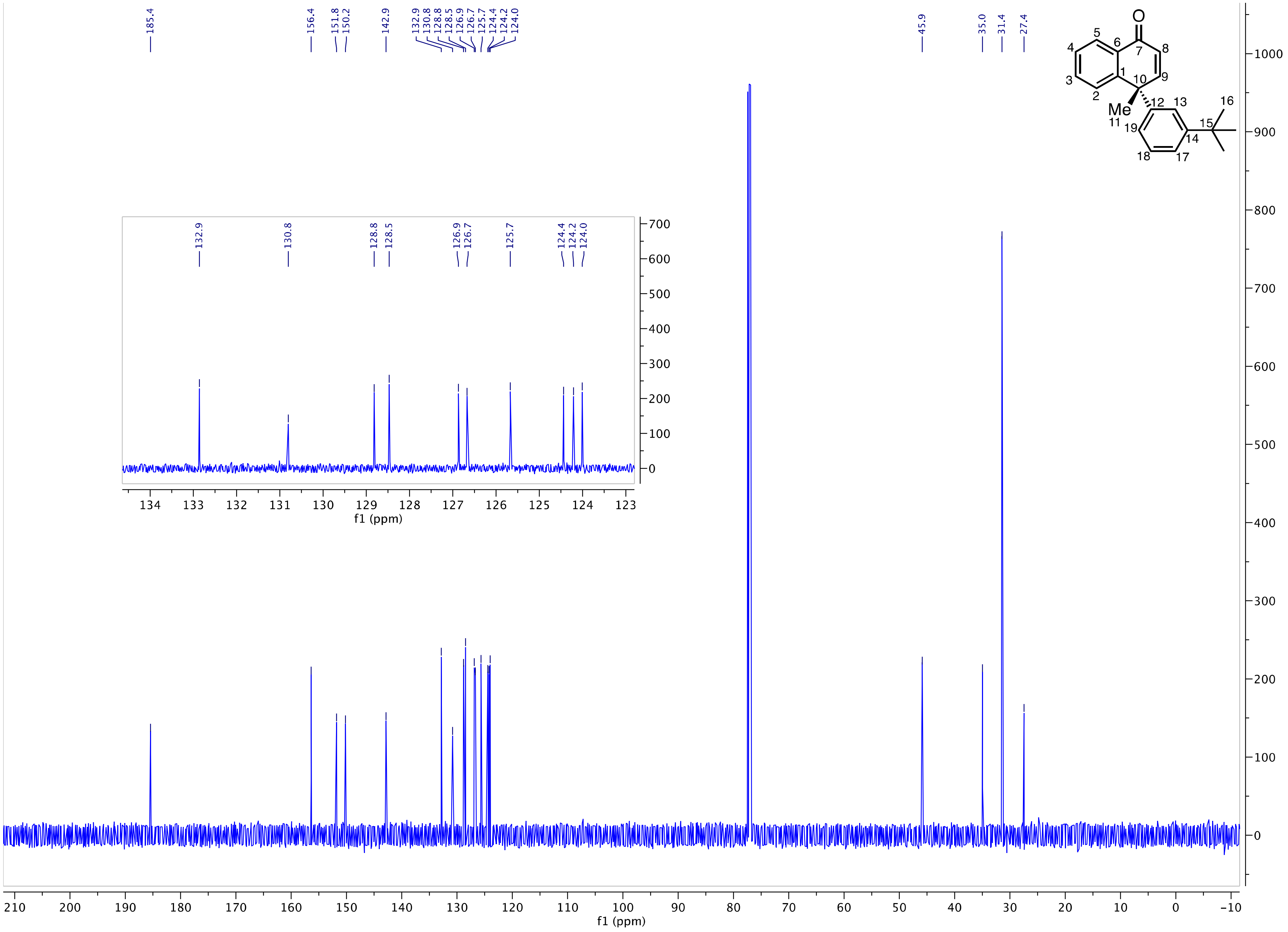

^{1}H NMR ($CDCl_3$): (*R*)-4-(4-(*tert*-butyl)phenyl)-4-Methylnaphthalen-1(4*H*)-one (**2aa**)

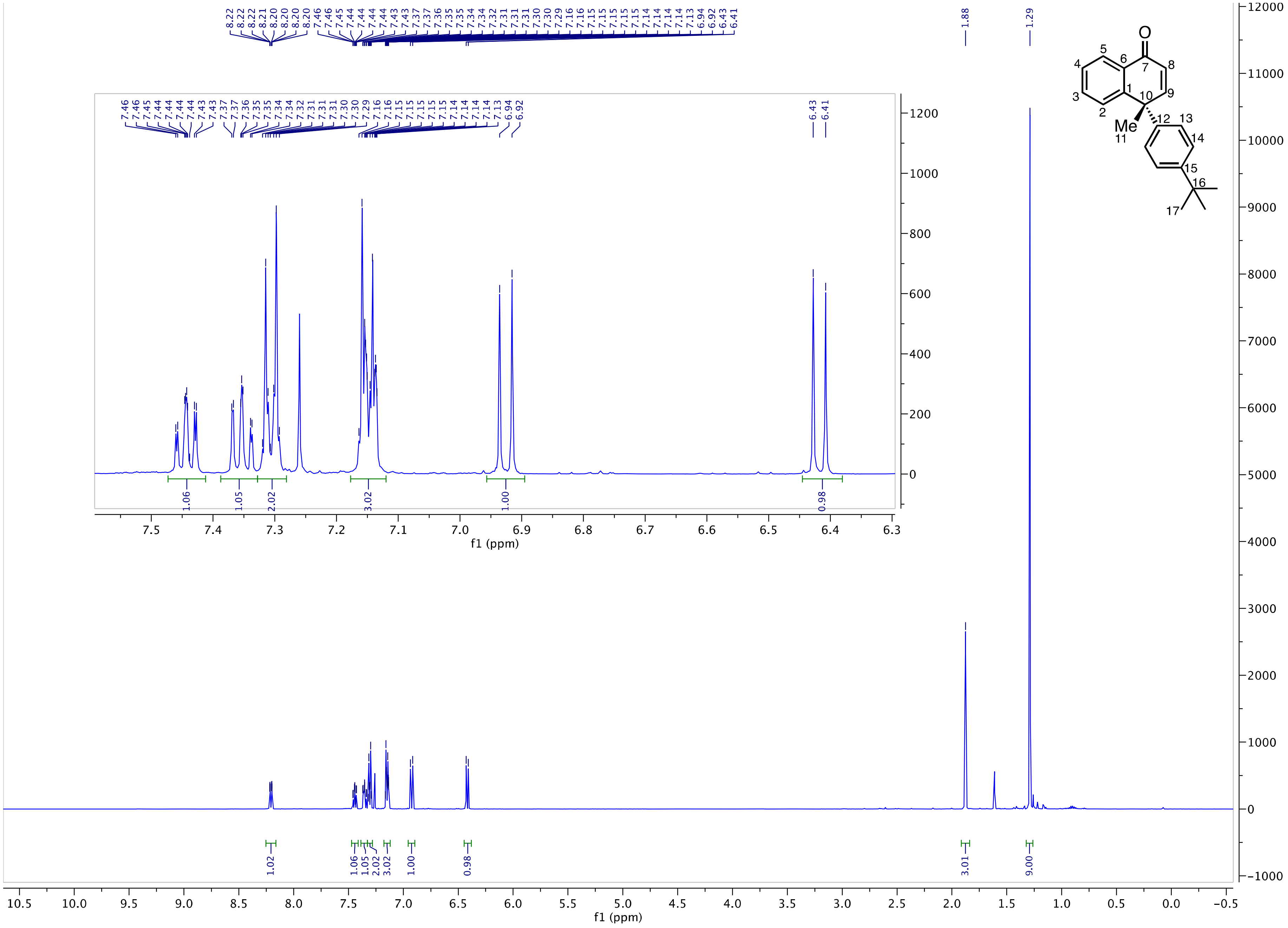

^{13}**C NMR** ($CDCl_3$): (*R*)-4-(4-(*tert*-butyl)phenyl)-4-Methylnaphthalen-1(4*H*)-one (**2aa**)

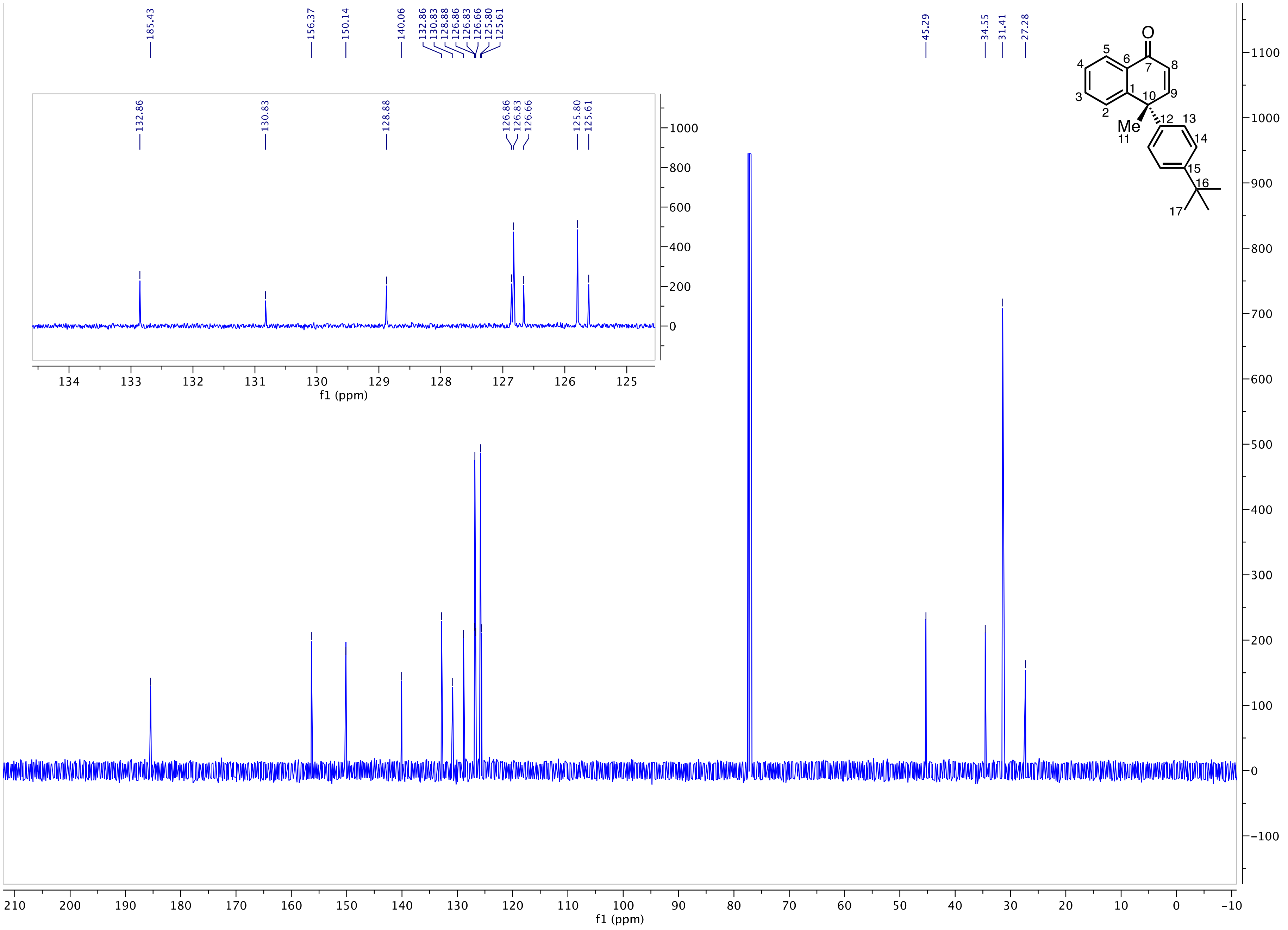

^{1}H NMR ($CDCl_3$): (*R*)-1-Methyl-[1,1'-binaphthalen]-4(1*H*)-one (**2ab**)

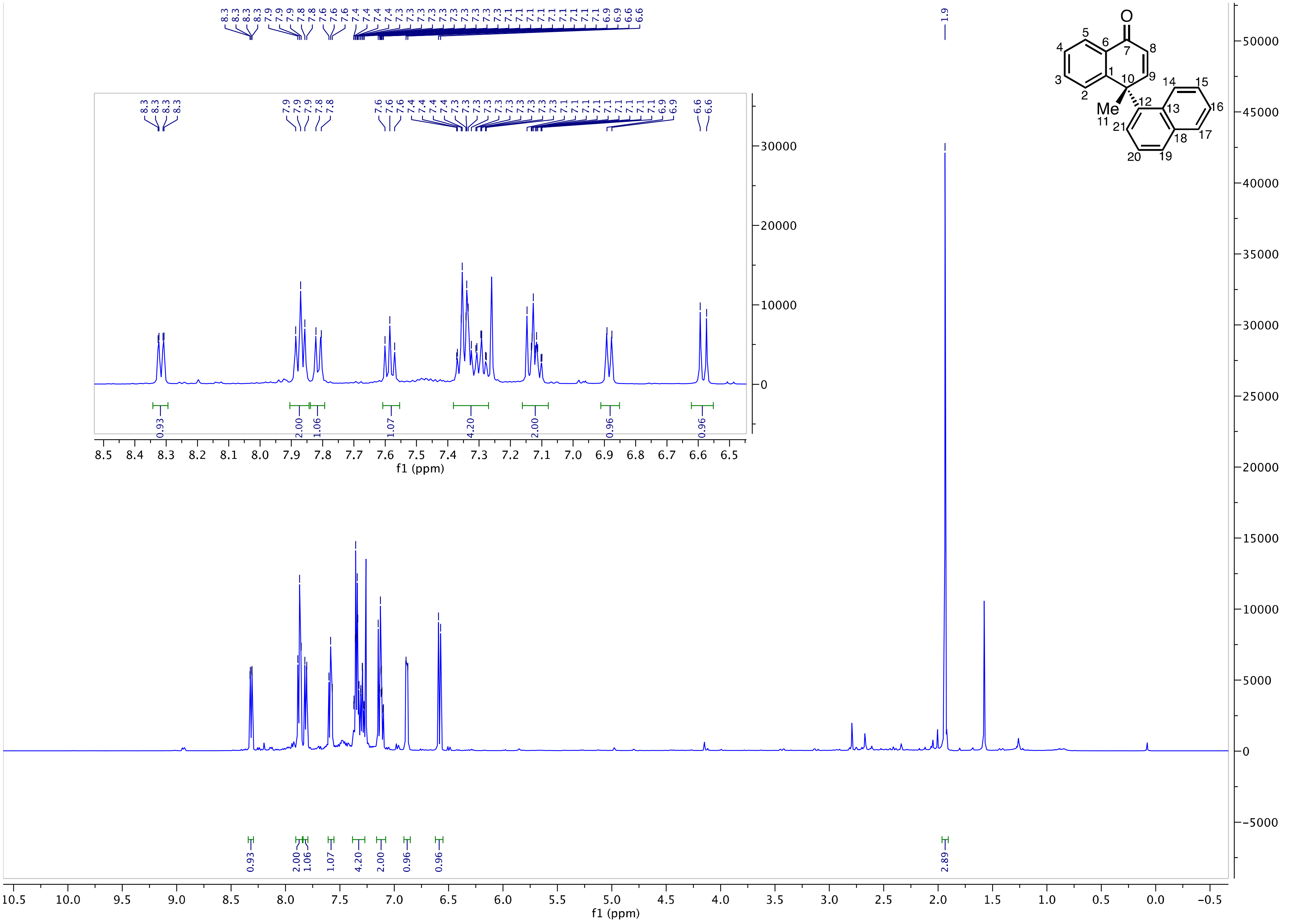

^{13}C NMR ($CDCl_3$): (*R*)-1-Methyl-[1,1'-binaphthalen]-4(1*H*)-one (**2ab**)

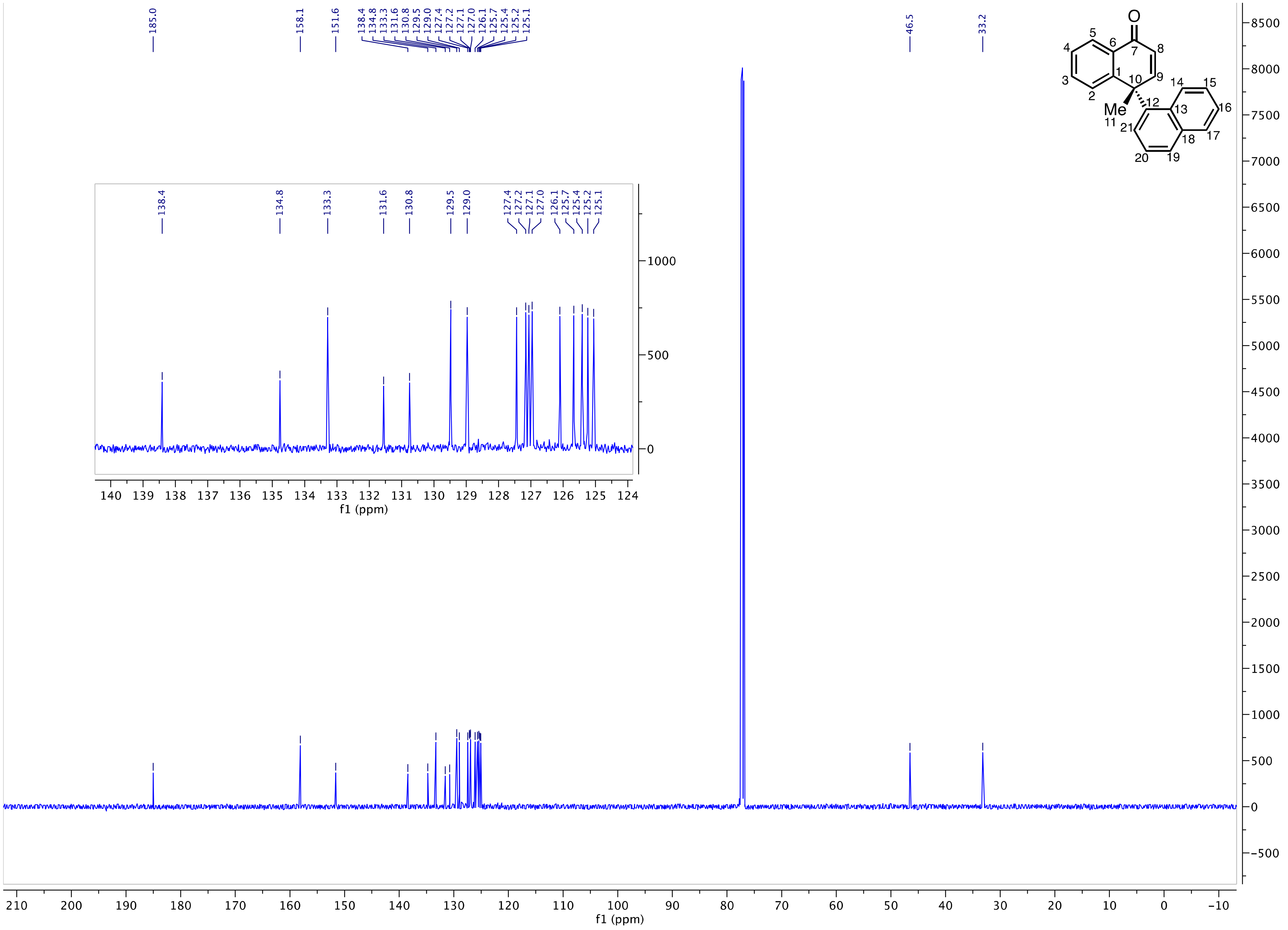

^{1}H NMR ($CDCl_3$): (*R*)-3-(4-(1-methyl-4-oxo-1,4-dihydronaphthalen-1-yl)phenyl)Propanenitrile (**2ac**)

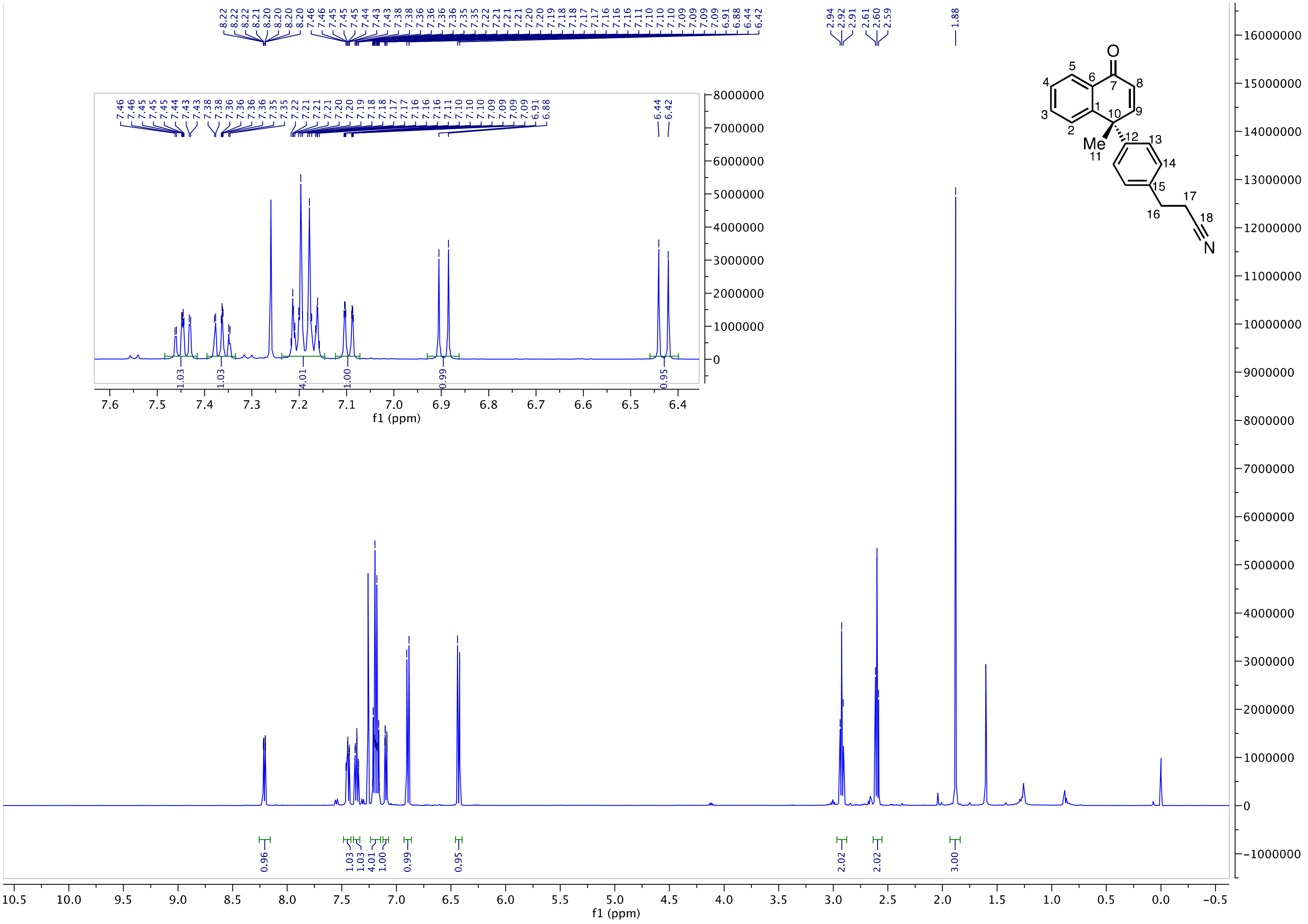

^{13}C NMR ($CDCl_3$): (*R*)-3-(4-(1-methyl-4-oxo-1,4-dihydronaphthalen-1-yl)phenyl)Propanenitrile (**2ac**)

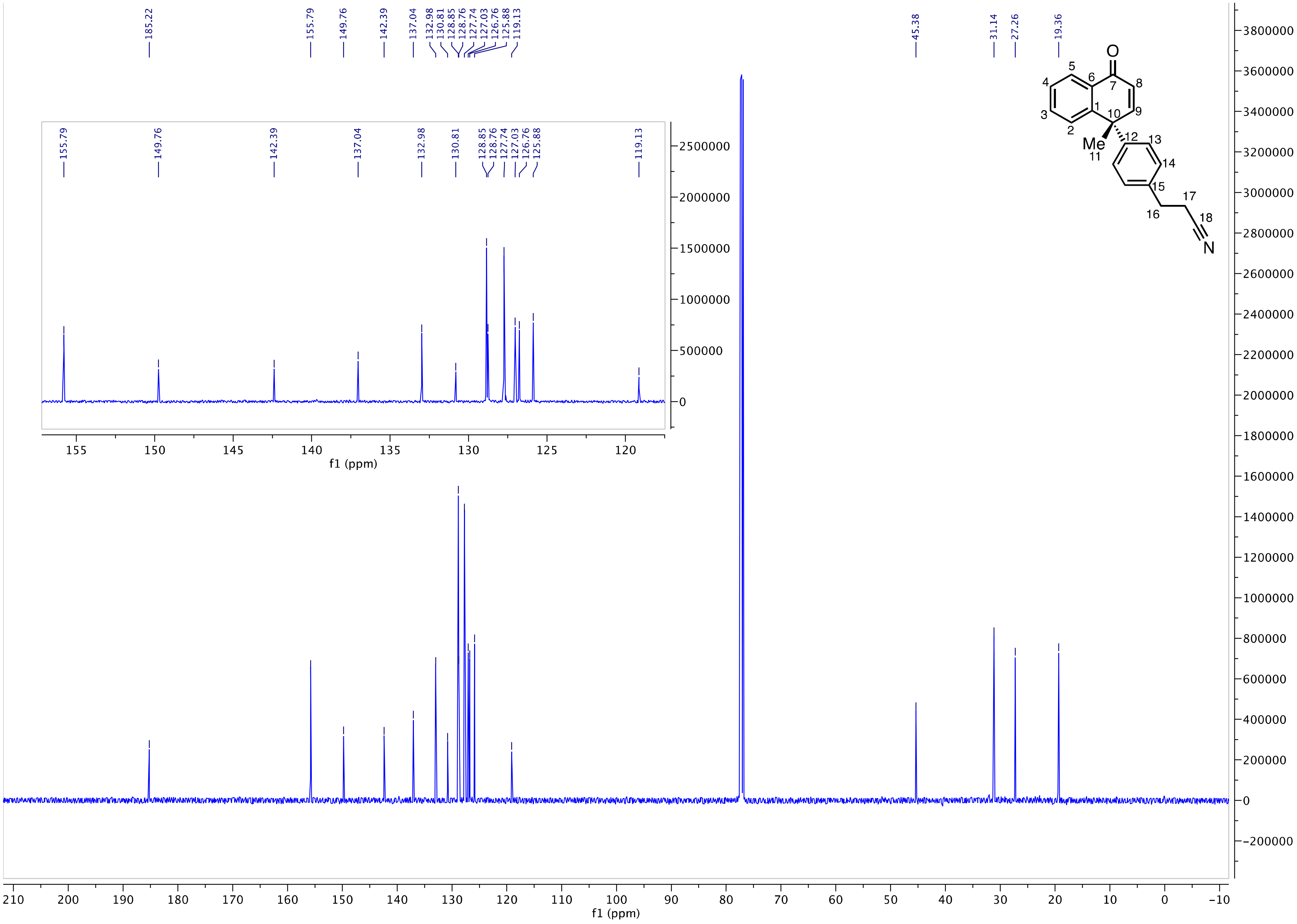

^{1}H NMR ($CDCl_3$): (*R*)-4-(benzofuran-5-yl)-4-Methylnaphthalen-1(4*H*)-one (**2ad**)

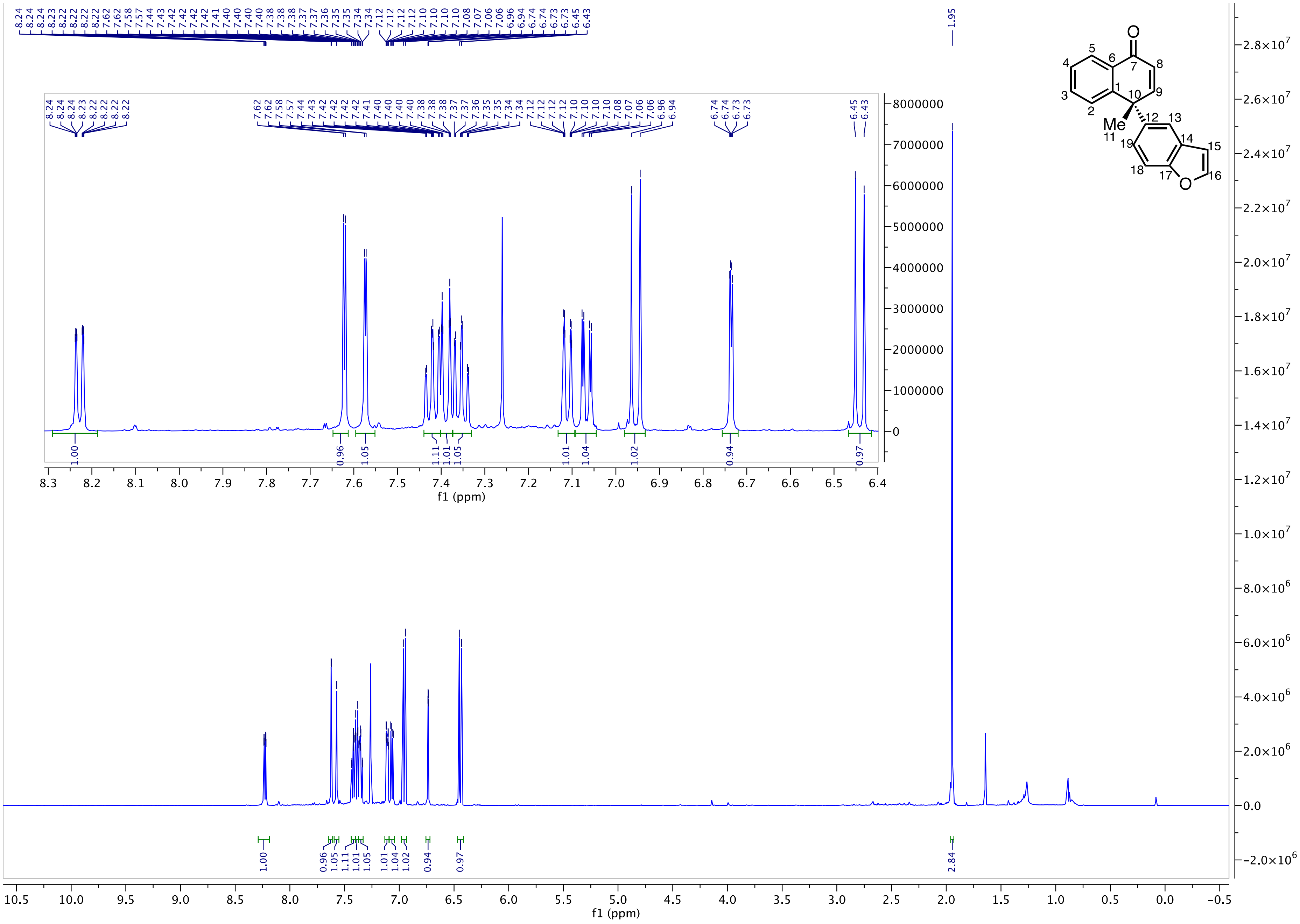

^{13}C NMR (CDCl$_3$): (*R*)-4-(benzofuran-5-yl)-4-Methylnaphthalen-1(4*H*)-one (**2ad**)

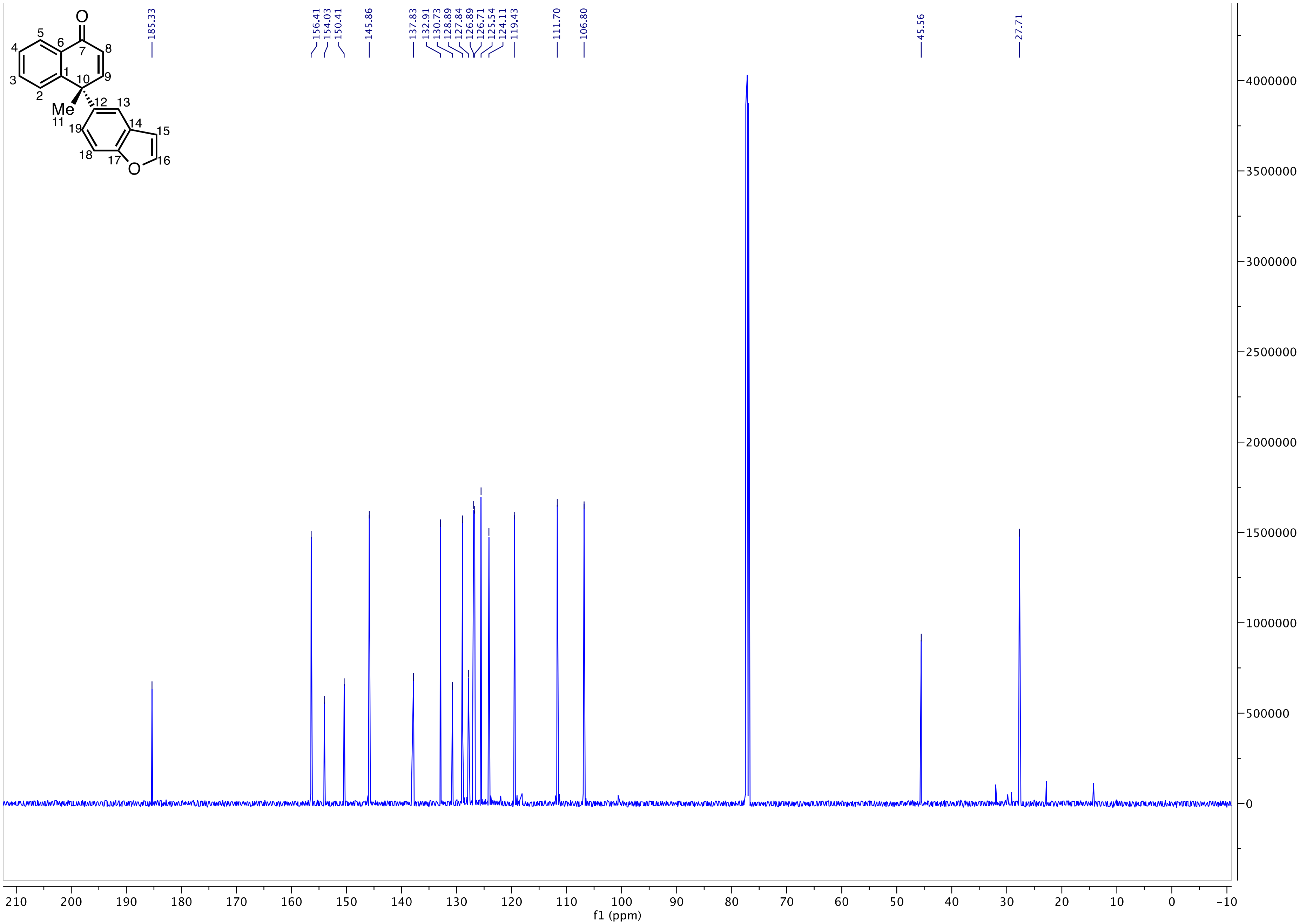

^{1}H NMR ($CDCl_3$): (*R*)-4-(1*H*-indol-6-yl)-4-Methylnaphthalen-1(4*H*)-one (**2ae**)

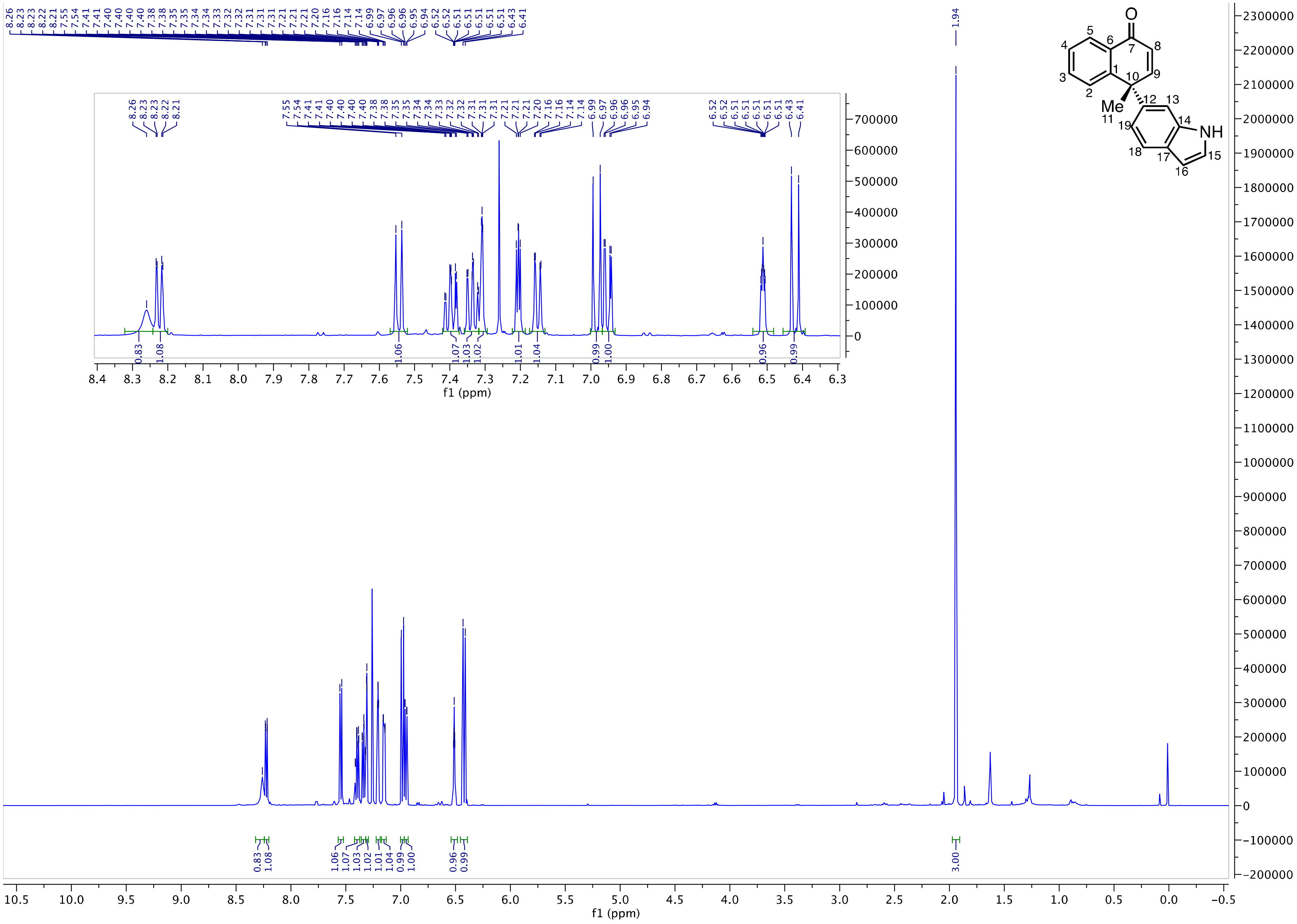

^{13}C NMR (CDCl$_3$): (*R*)-4-(1*H*-indol-6-yl)-4-Methylnaphthalen-1(4*H*)-one (**2ae**)

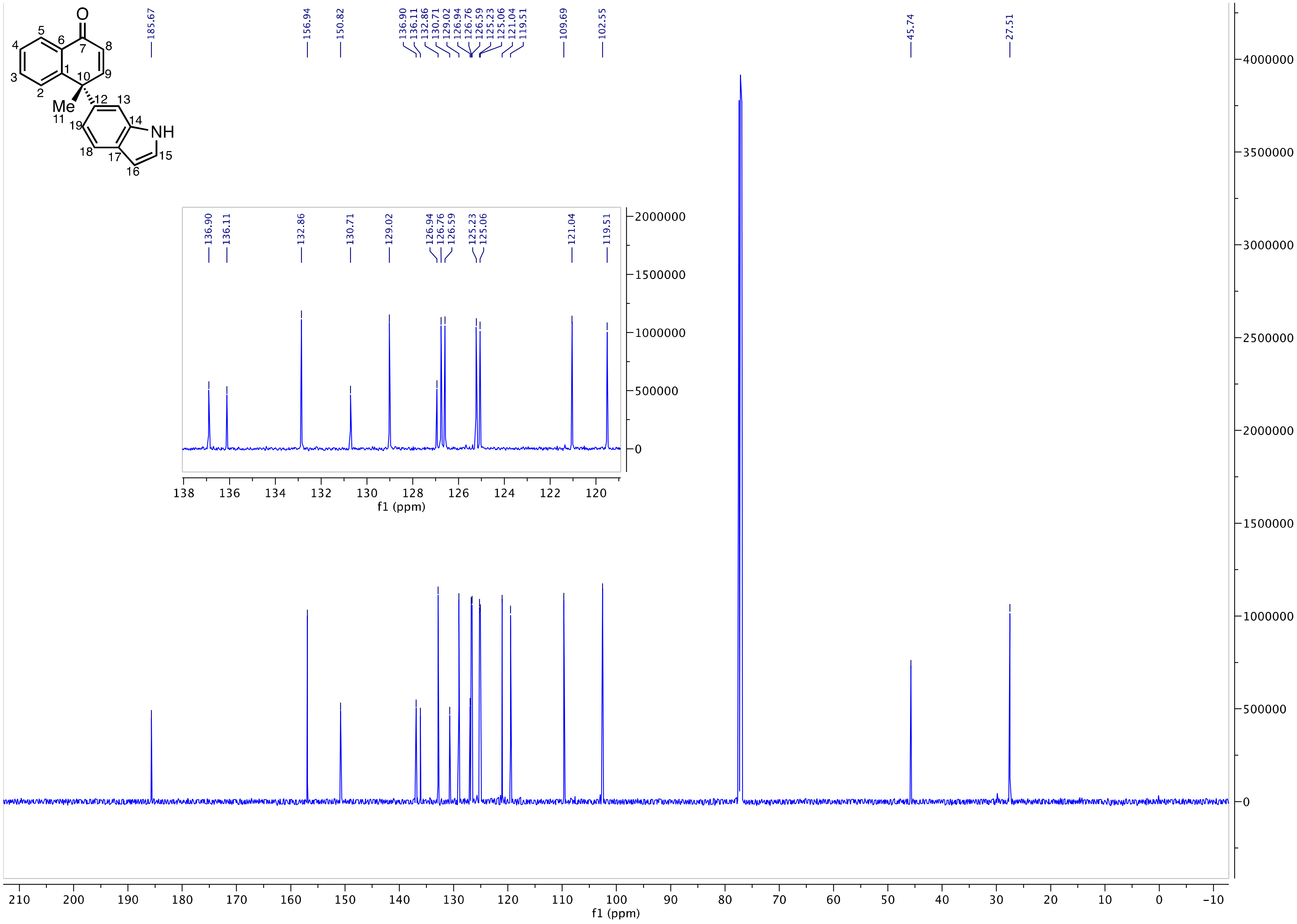

^{1}H NMR ($CDCl_3$): (*R*)-4-(1*H*-indol-5-yl)-4-Methylnaphthalen-1(4*H*)-one (**2af**)

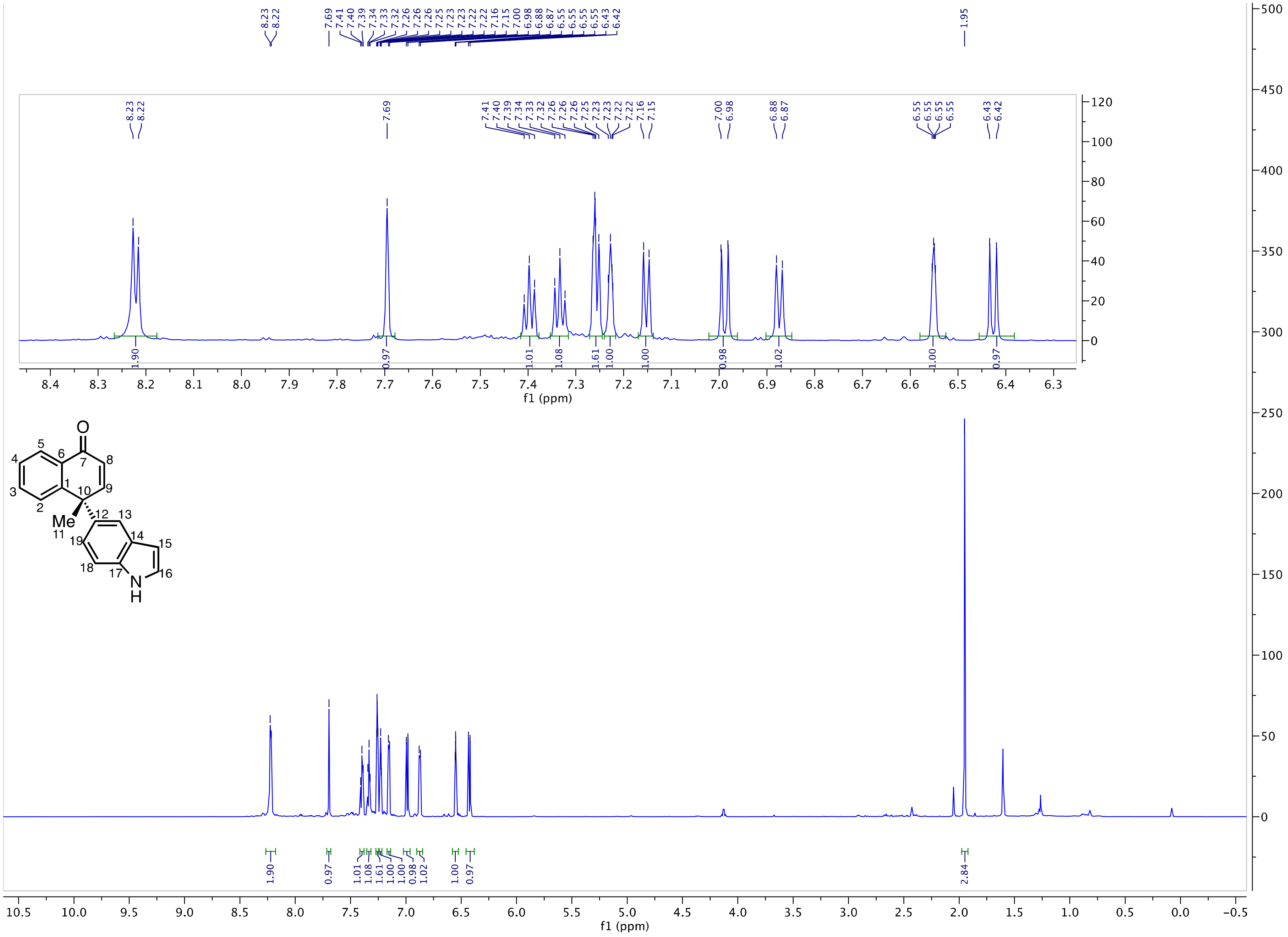

^{13}C NMR ($CDCl_3$): (*R*)-4-(1*H*-indol-5-yl)-4-Methylnaphthalen-1(4*H*)-one (**2af**)

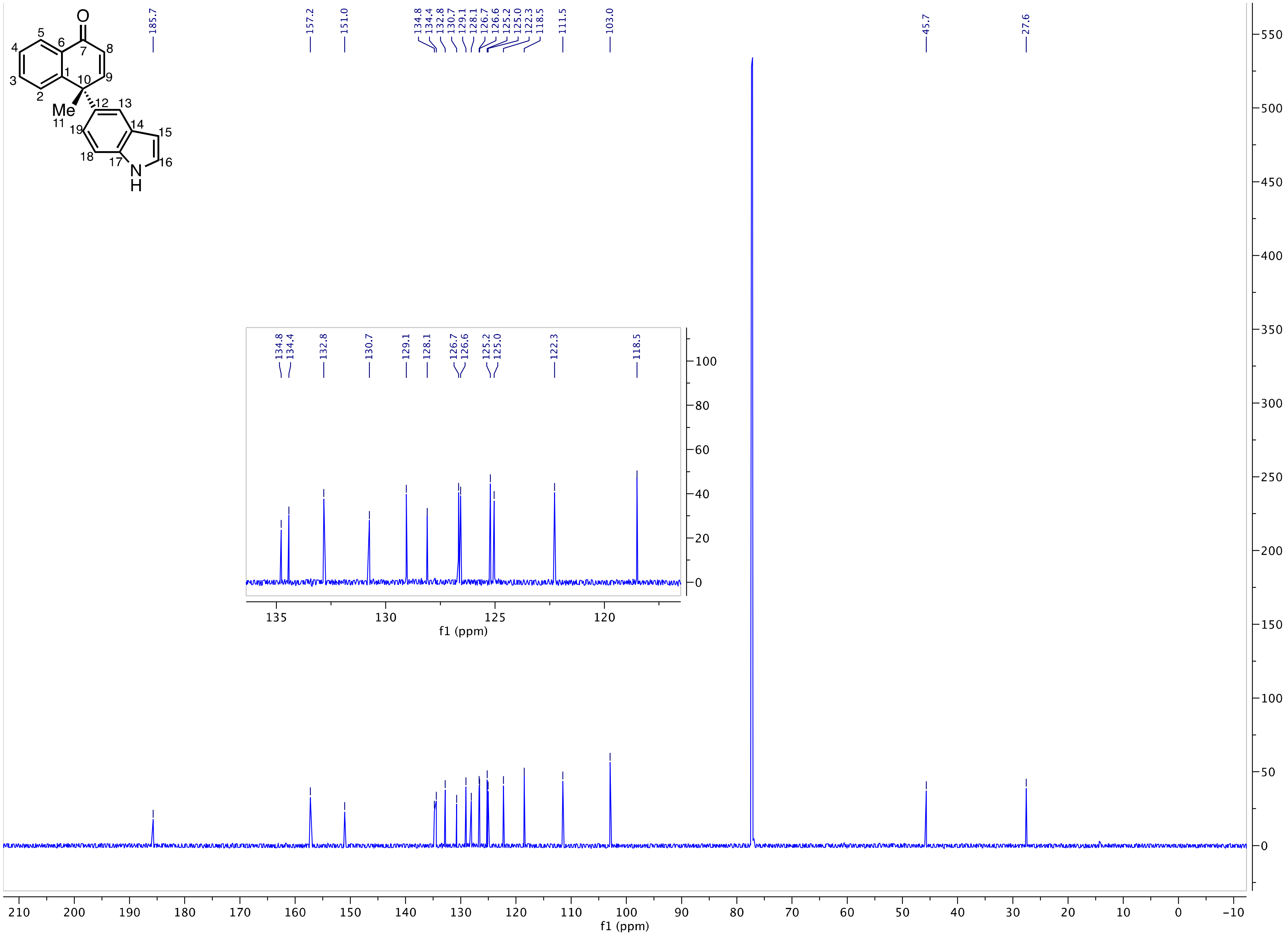

^{1}H NMR ($CDCl_3$): (*S*)-4-Methyl-4-(thiophen-3-yl)naphthalen-1(4*H*)-one **(2ag)**

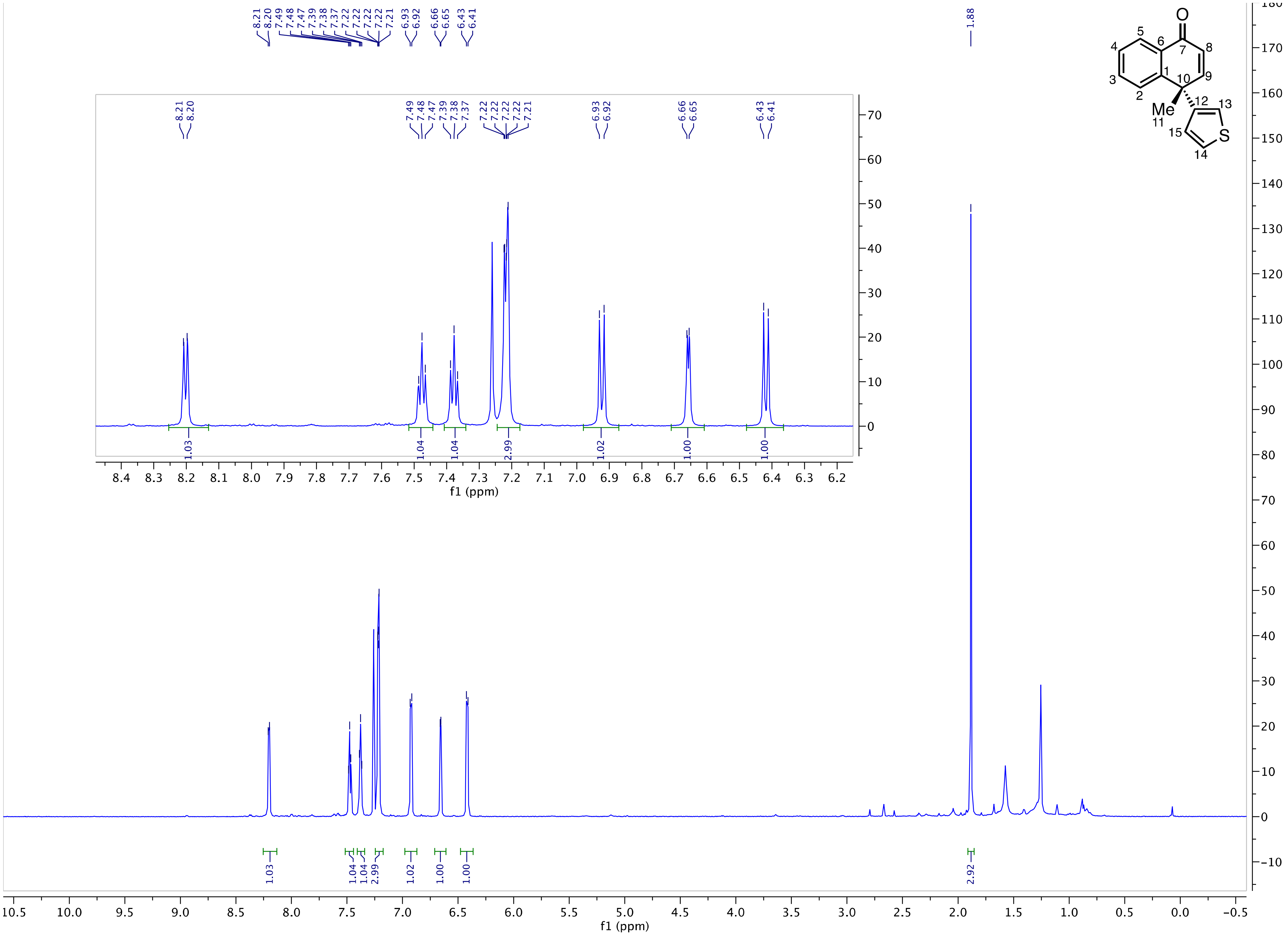

^{13}C NMR ($CDCl_3$): (*S*)-4-Methyl-4-(thiophen-3-yl)naphthalen-1(4*H*)-one **(2ag)**

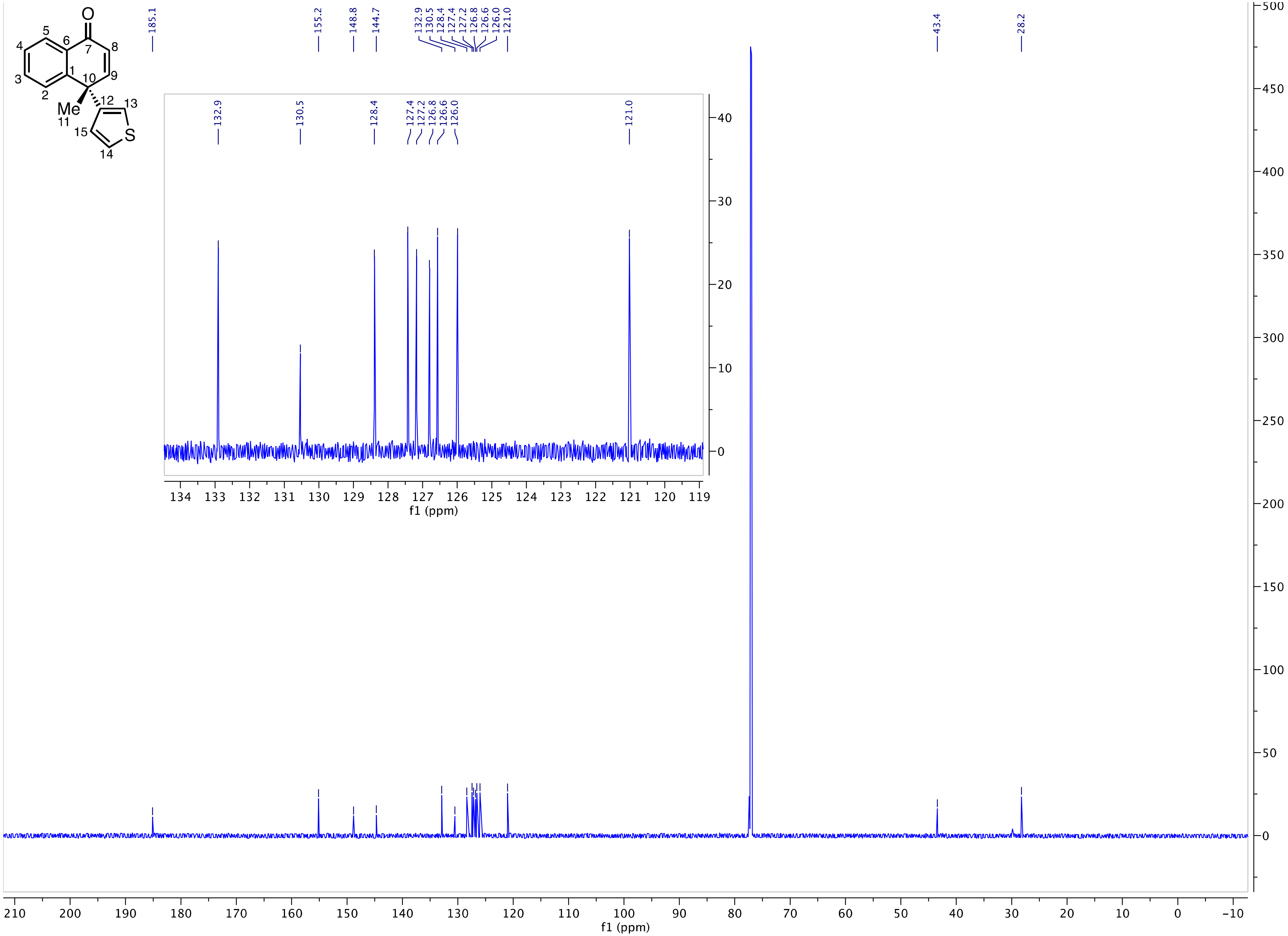

^{1}H NMR ($CDCl_3$): (*R*)-4-(cyclohex-1-en-1-yl)-4-Methylnaphthalen-1(4*H*)-one (**2al**)

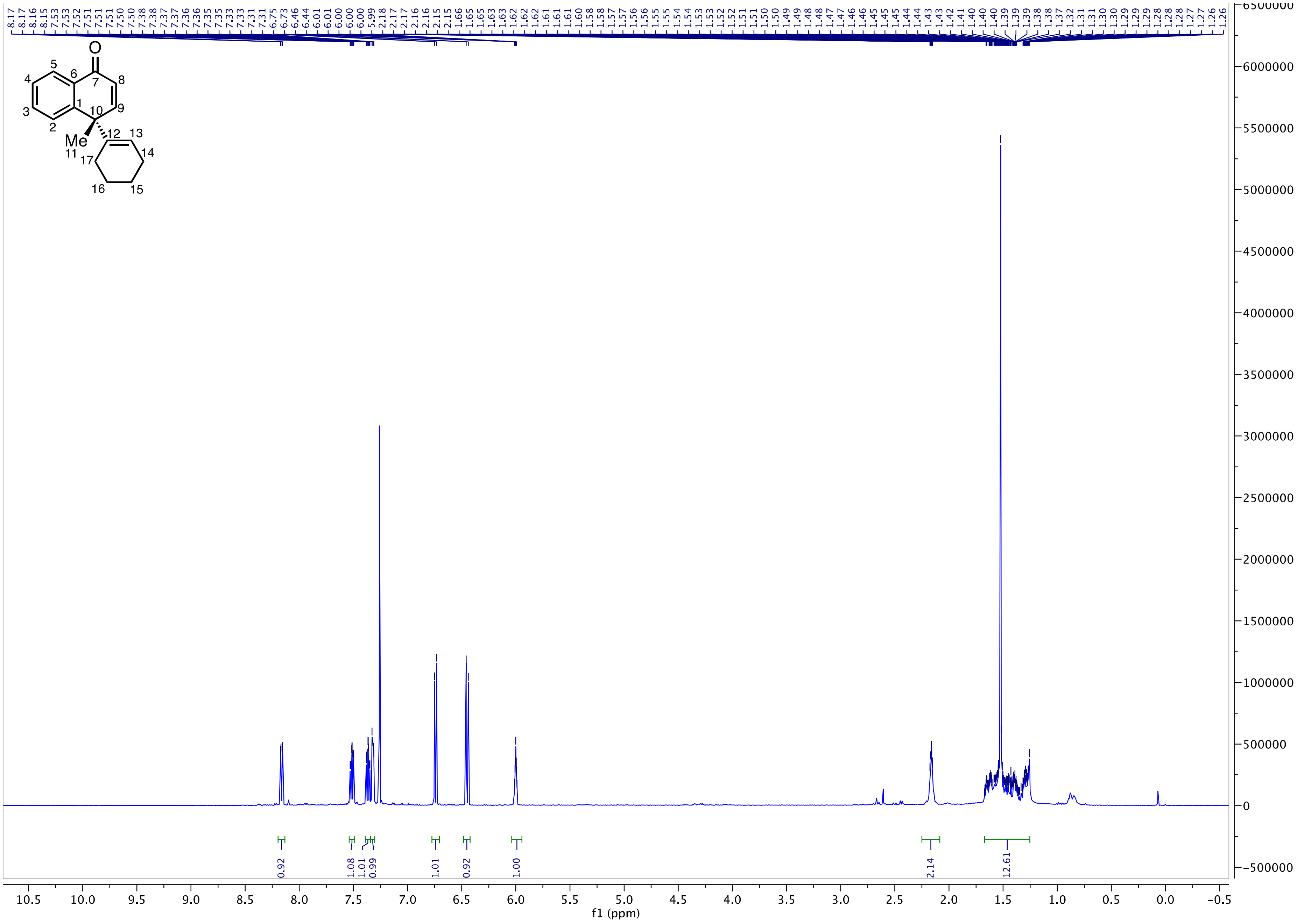

^{13}C NMR ($CDCl_3$): (*R*)-4-(cyclohex-1-en-1-yl)-4-Methylnaphthalen-1(4*H*)-one (**2al**)

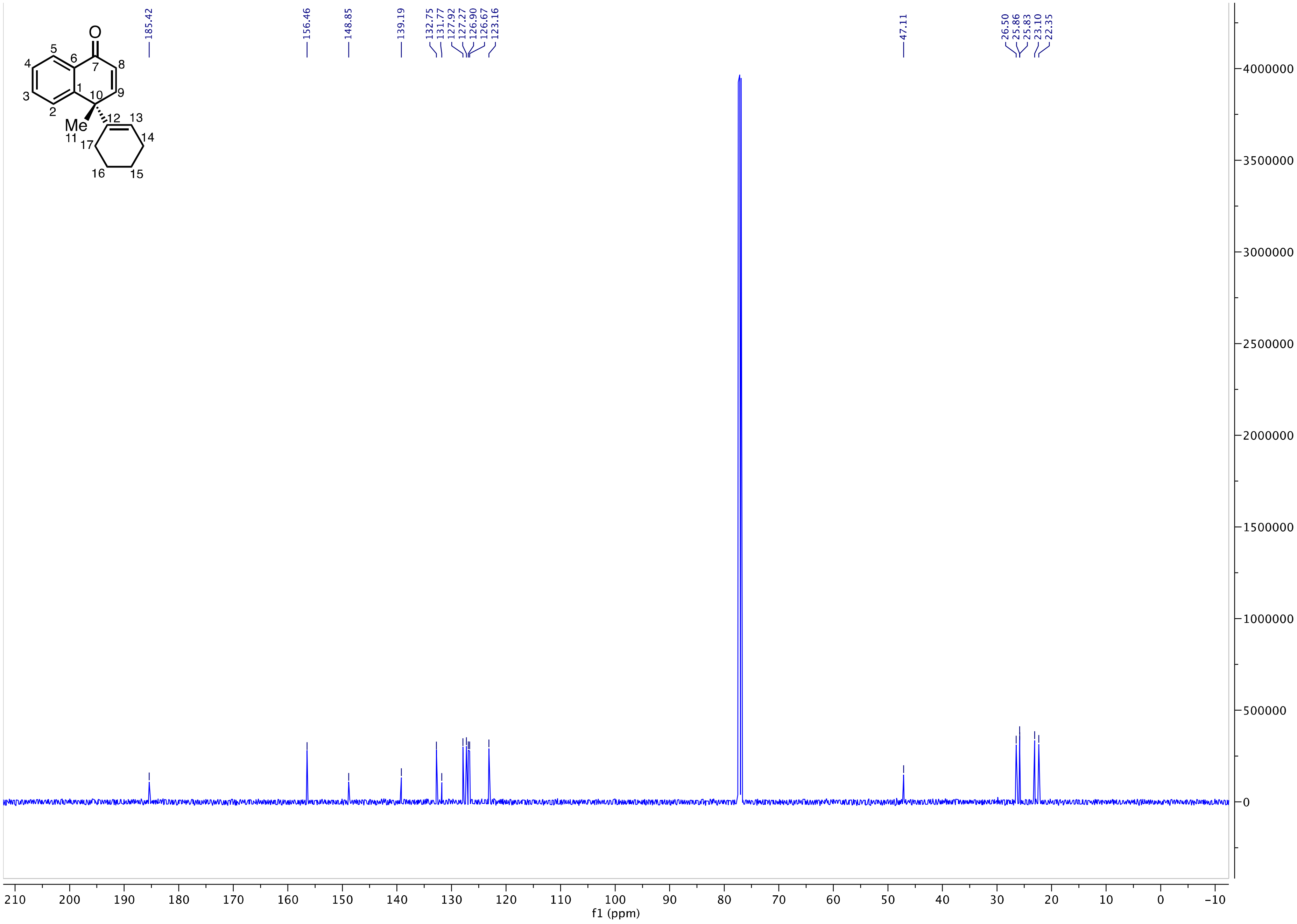

^{1}H NMR ($CDCl_3$): (*S*,*E*)-4-Methyl-4-styrylnaphthalen-1(4*H*)-one (**2am**)

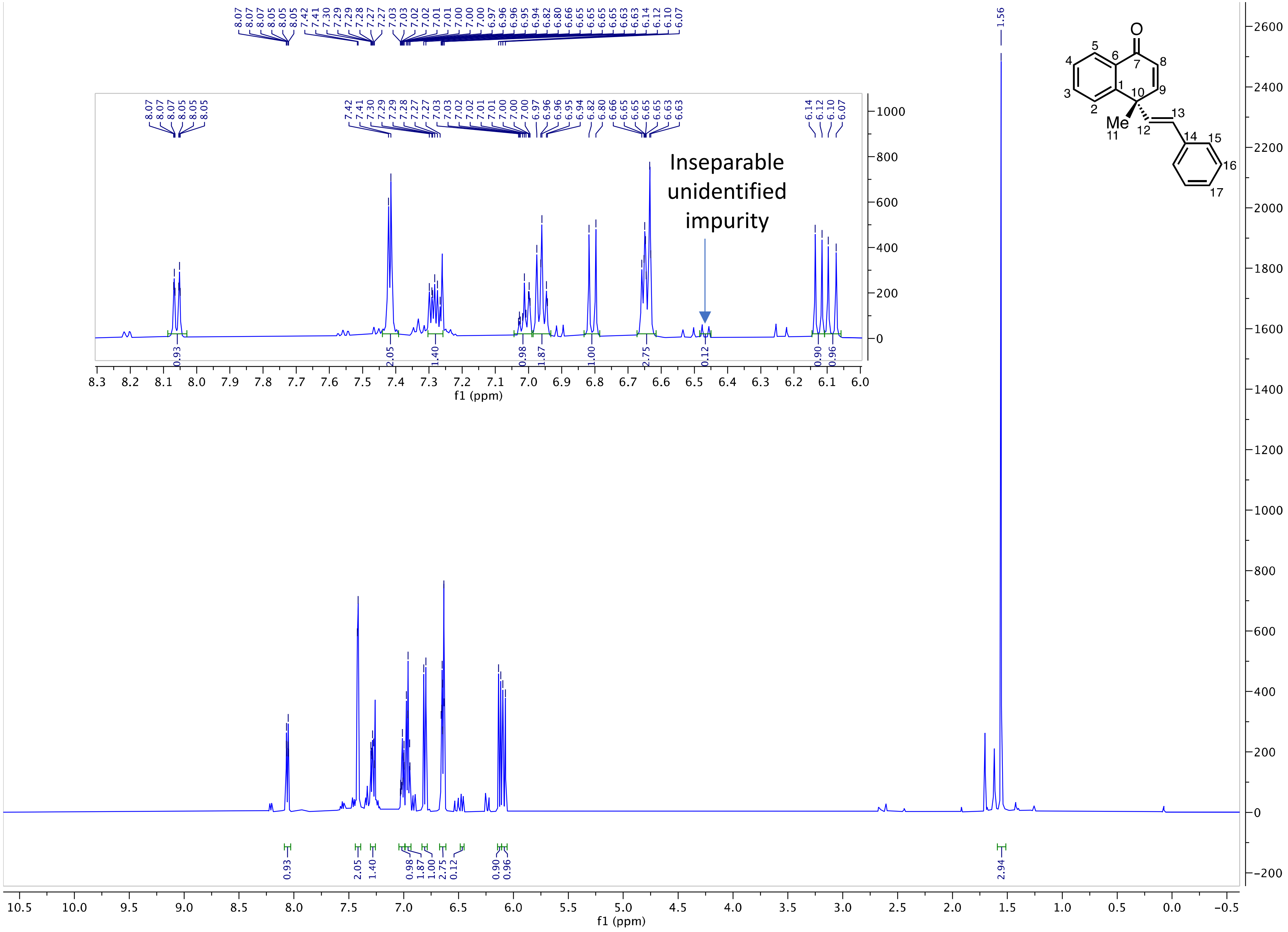

^{13}**C NMR** (CDCl$_3$): (*S*,*E*)-4-Methyl-4-styrylnaphthalen-1(4*H*)-one (**2am**)

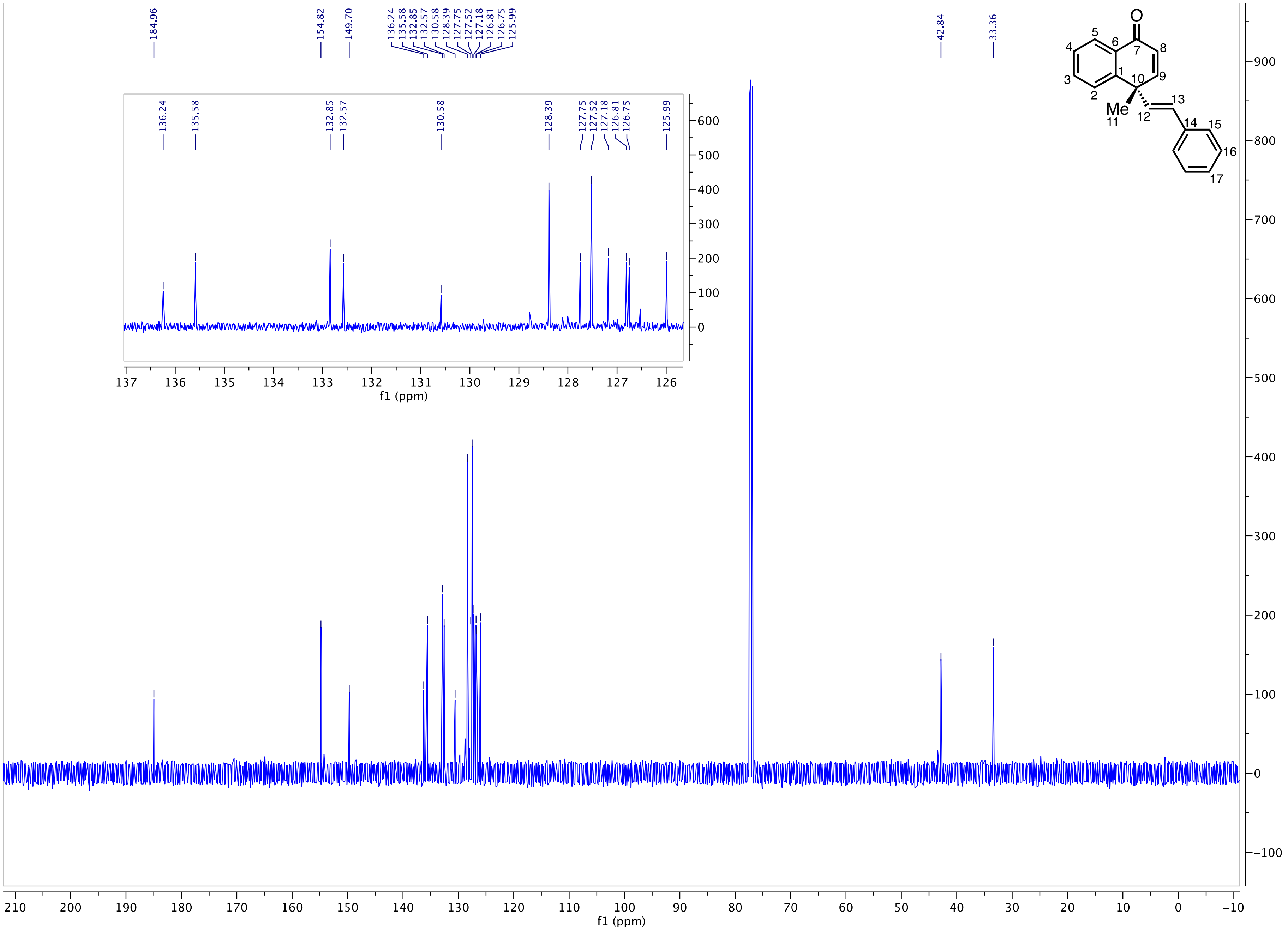

^{1}H NMR ($CDCl_3$): (*R*)-4-(9,9-dibutyl-9*H*-fluoren-2-yl)-4-Methylnaphthalen-1(4*H*)-one (**2an**)

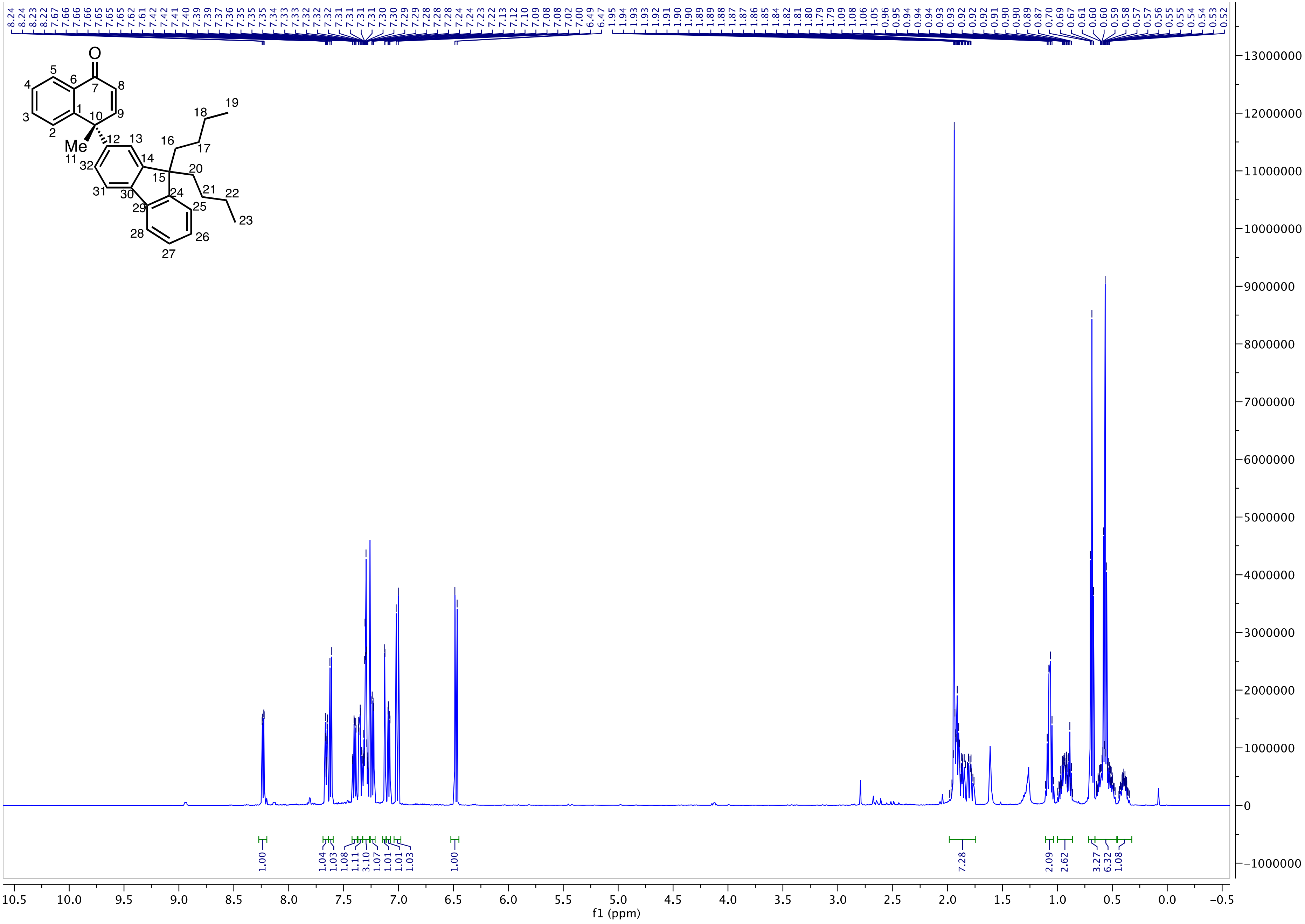

^{13}**C NMR** (CDCl$_3$): (*R*)-4-(9,9-dibutyl-9*H*-fluoren-2-yl)-4-Methylnaphthalen-1(4*H*)-one (**2an**)

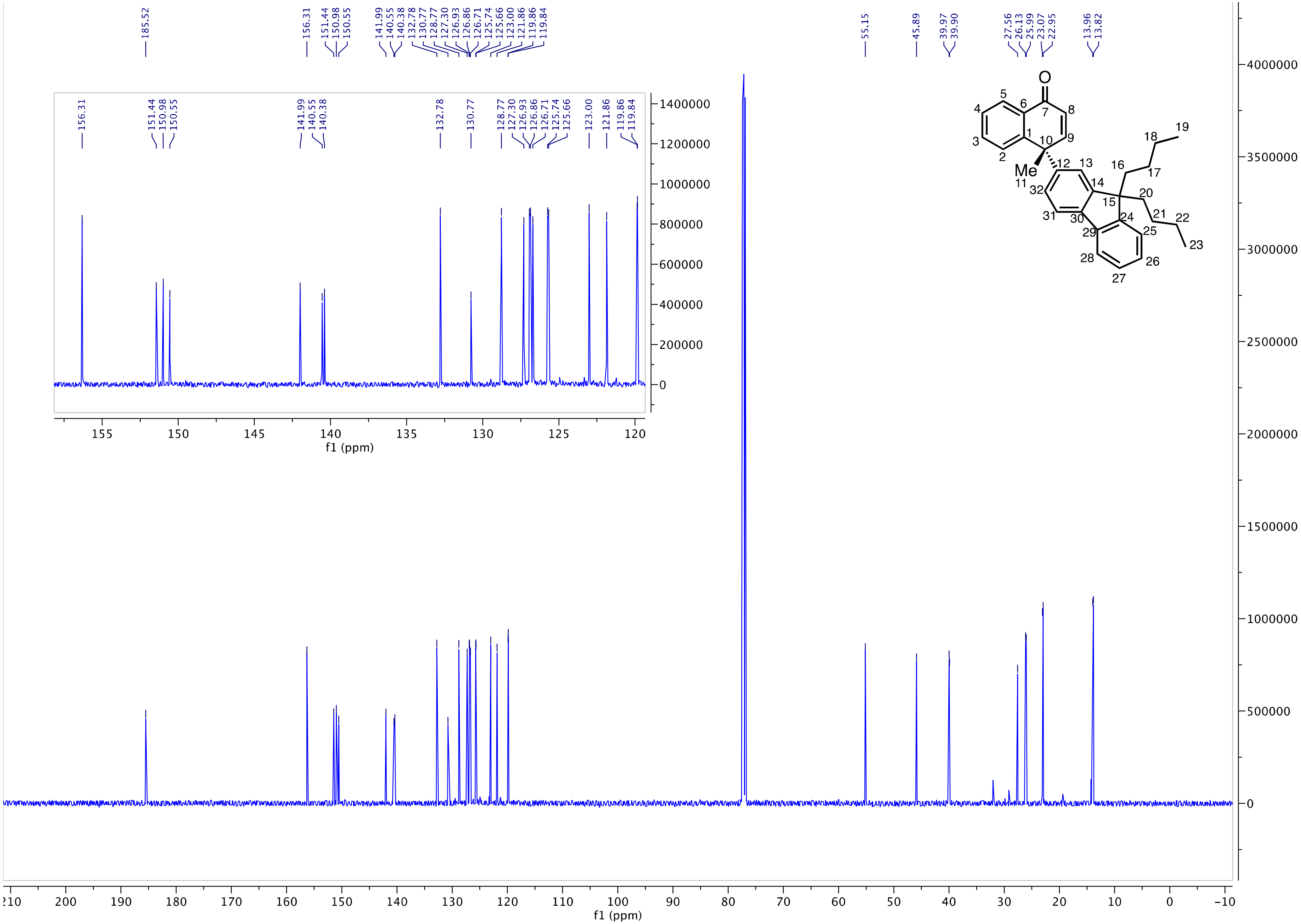

^{1}H NMR ($CDCl_3$): Benzyl (*R*)-4-(4-(1-methyl-4-oxo-1,4-dihydronaphthalen-1-yl)phenyl)piperazine-1-carboxylate (**2ao**)

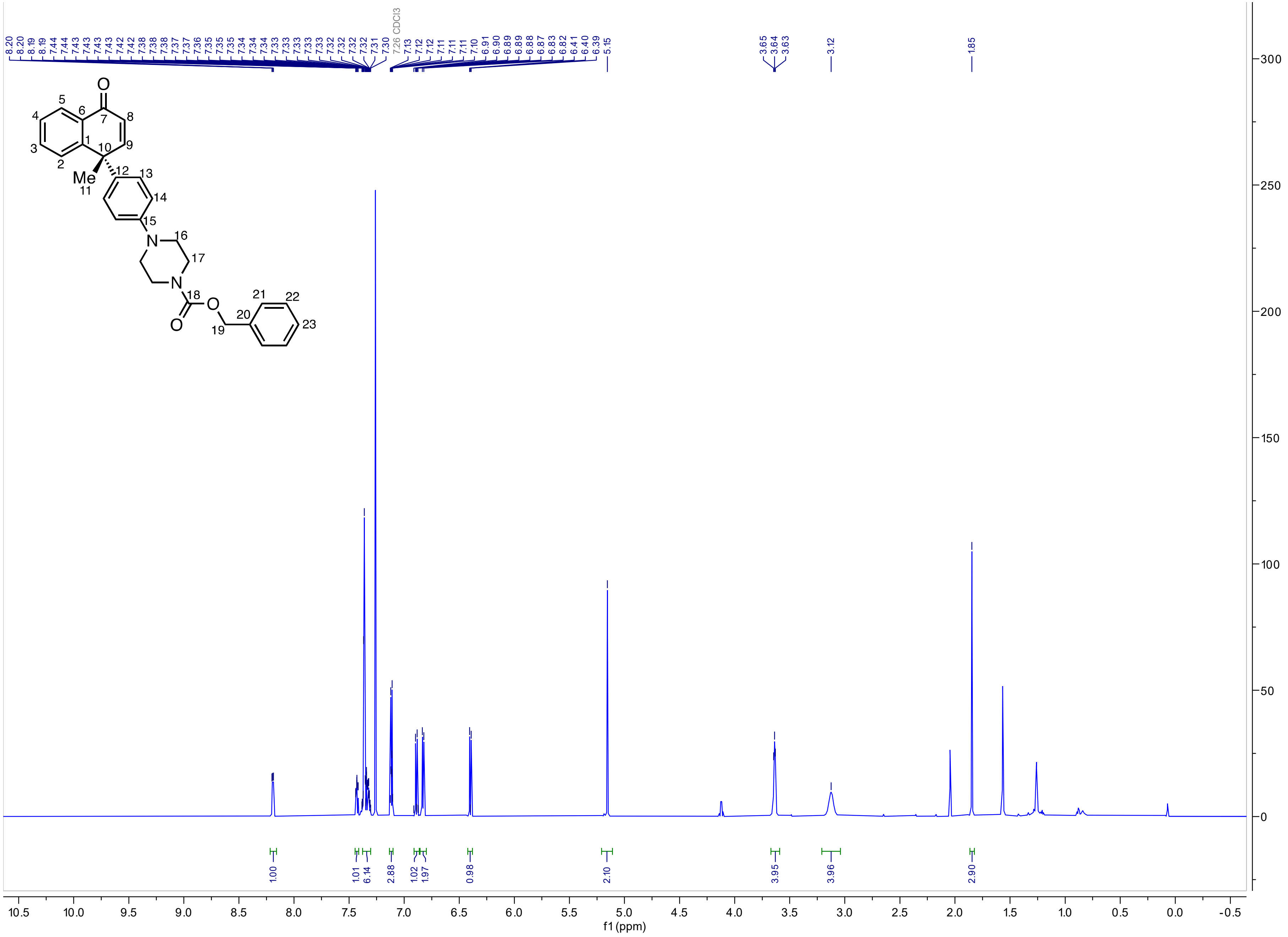

^{13}C NMR ($CDCl_3$): Benzyl (*R*)-4-(4-(1-methyl-4-oxo-1,4-dihydronaphthalen-1-yl)phenyl)piperazine-1-carboxylate **(2ao)**

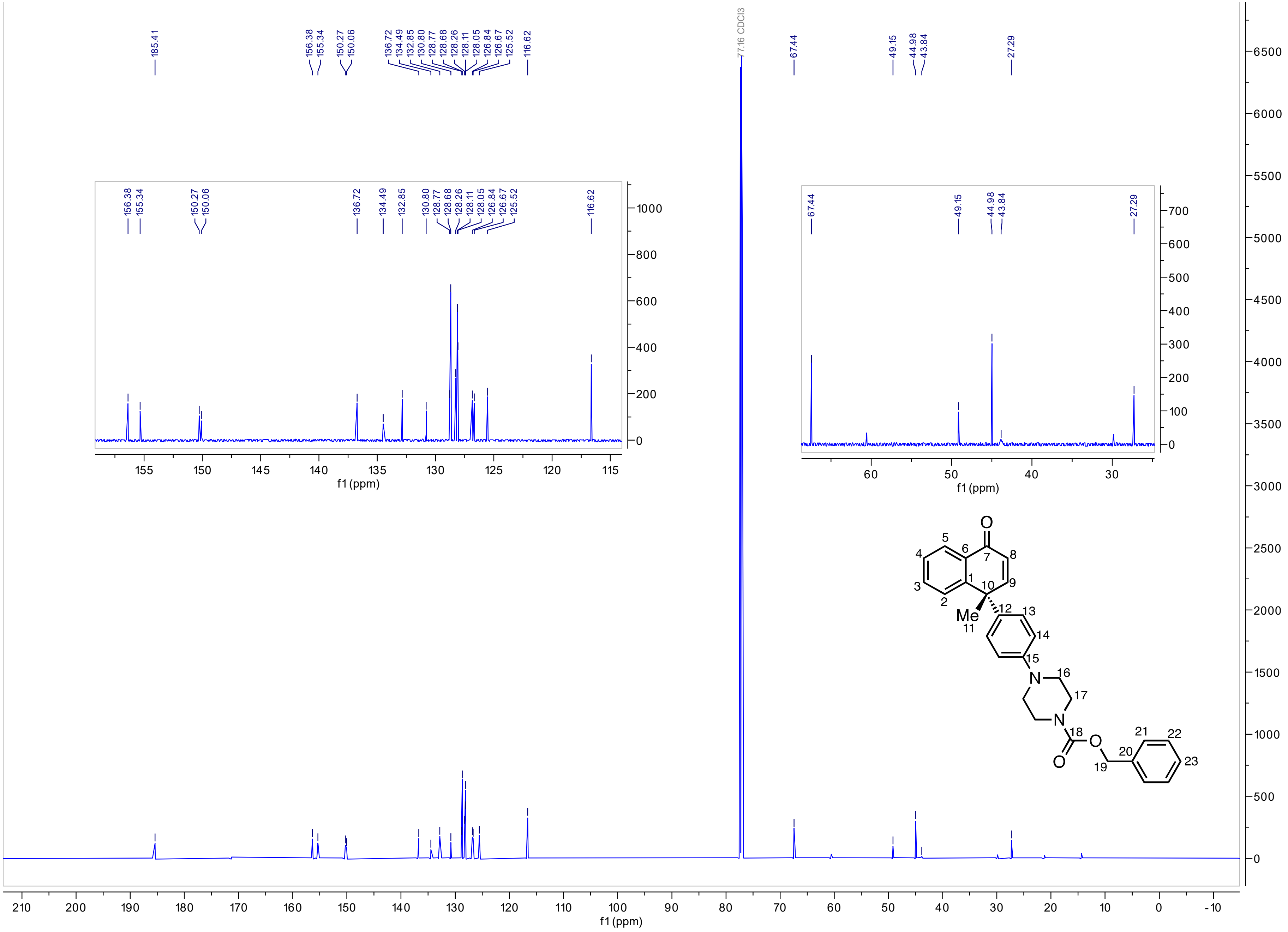

^{1}H NMR ($CDCl_3$): (*R*)-4-(4-(2,6-diphenylpyrimidin-4-yl)phenyl)-4-Methylnaphthalen-1(4*H*)-one (**2ap**)

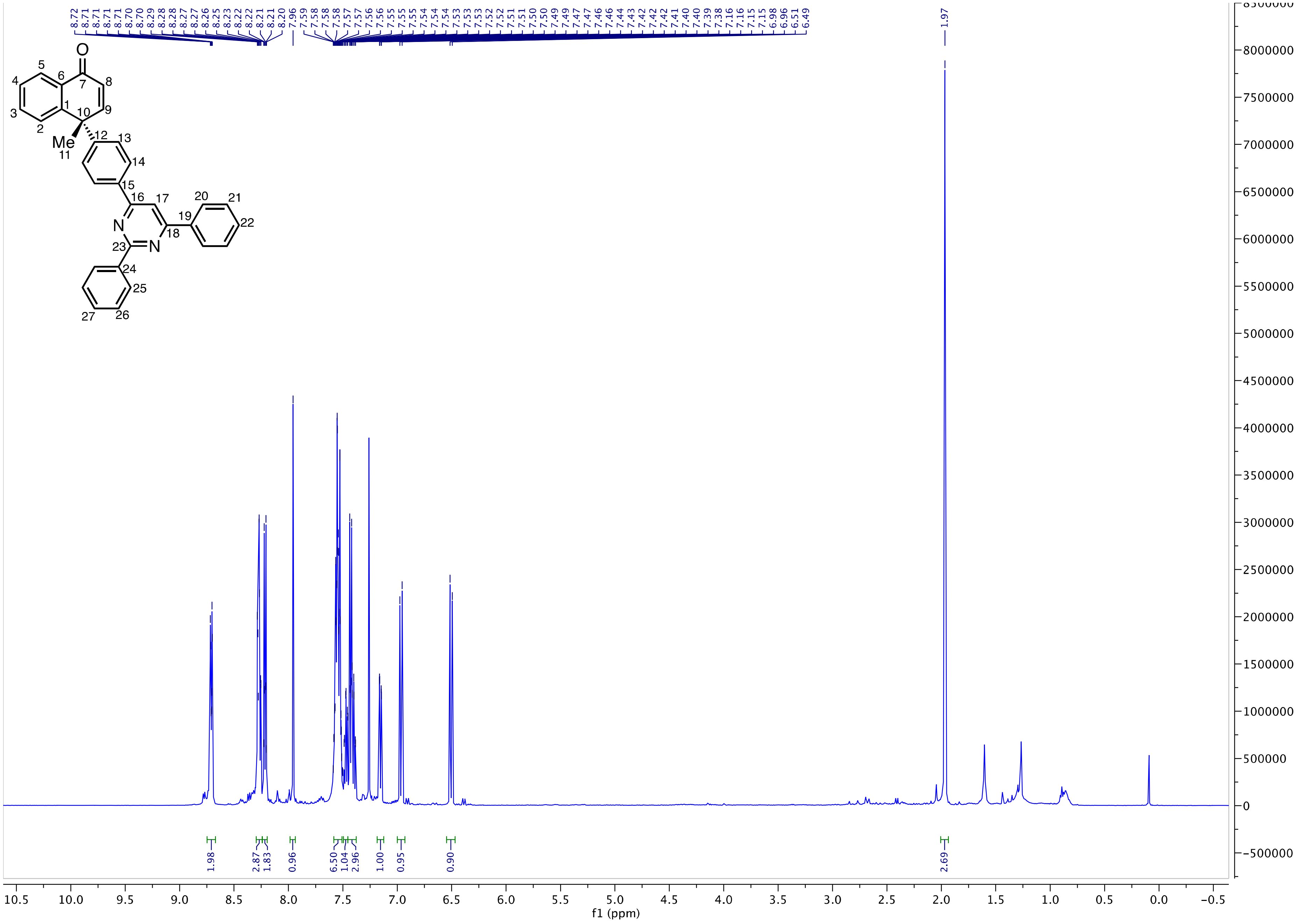

^{13}C NMR ($CDCl_3$): (*R*)-4-(4-(2,6-diphenylpyrimidin-4-yl)phenyl)-4-Methylnaphthalen-1(4*H*)-one (**2ap**)

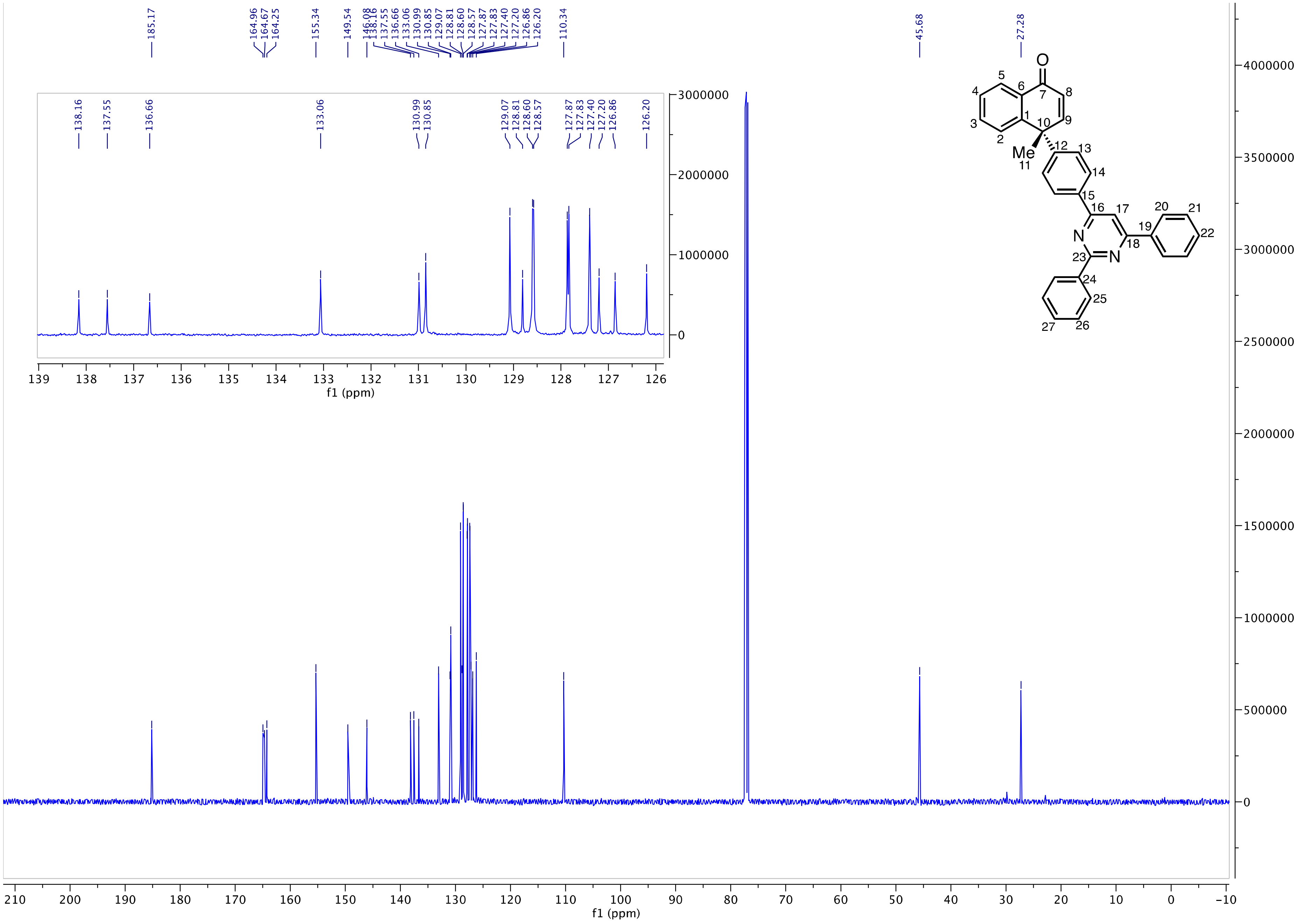

^{1}H NMR ($CDCl_3$): (*R*)-4-(4-chloro-3-(4-(((*R*)-tetrahydrofuran-3-yl)oxy)benzyl)phenyl)-4-Methylnaphthalen-1(4*H*)-one (**2aq**)

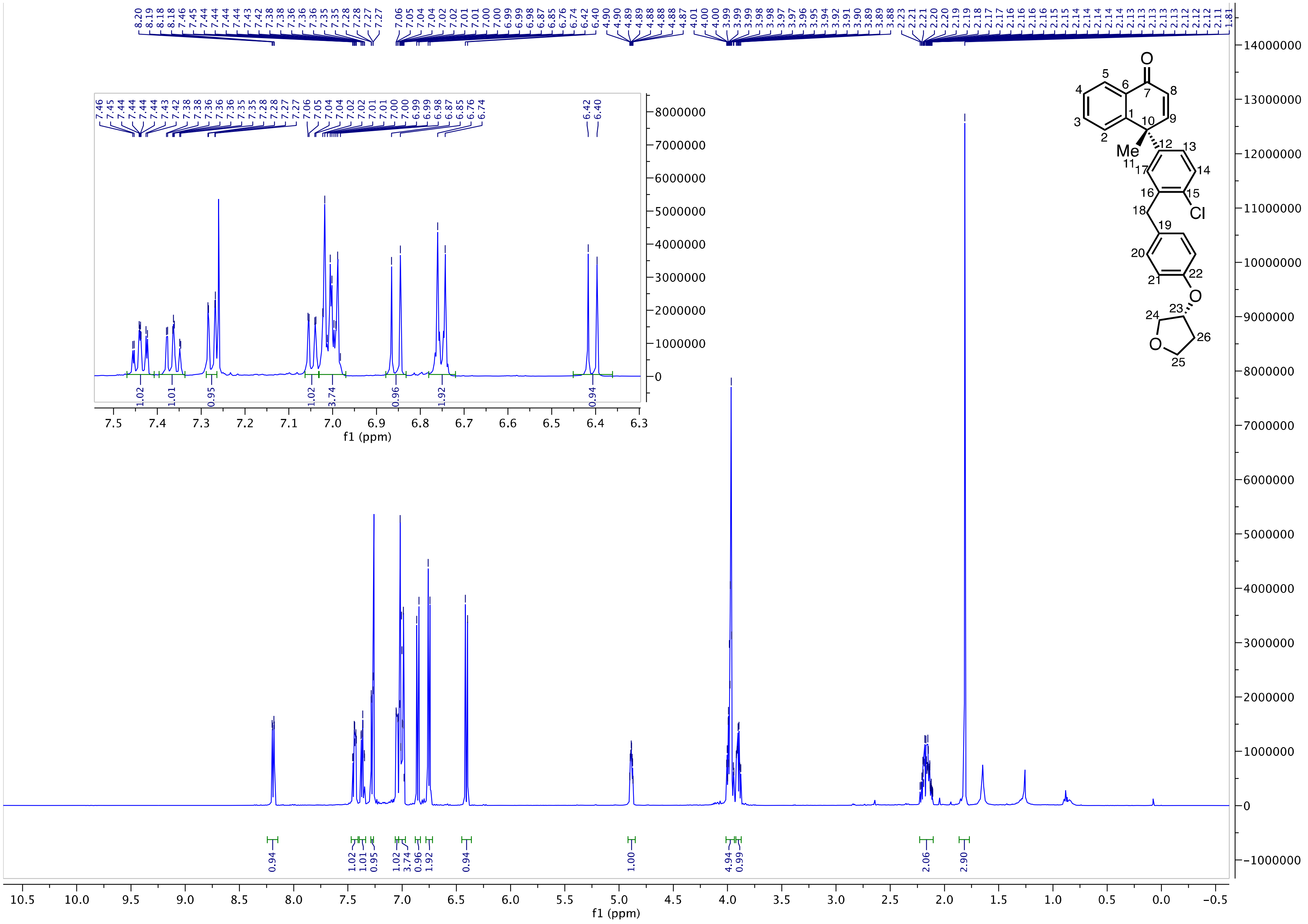

^{13}C NMR ($CDCl_3$): (*R*)-4-(4-chloro-3-(4-(((*R*)-tetrahydrofuran-3-yl)oxy)benzyl)phenyl)-4-Methylnaphthalen-1(4*H*)-one (**2aq**)

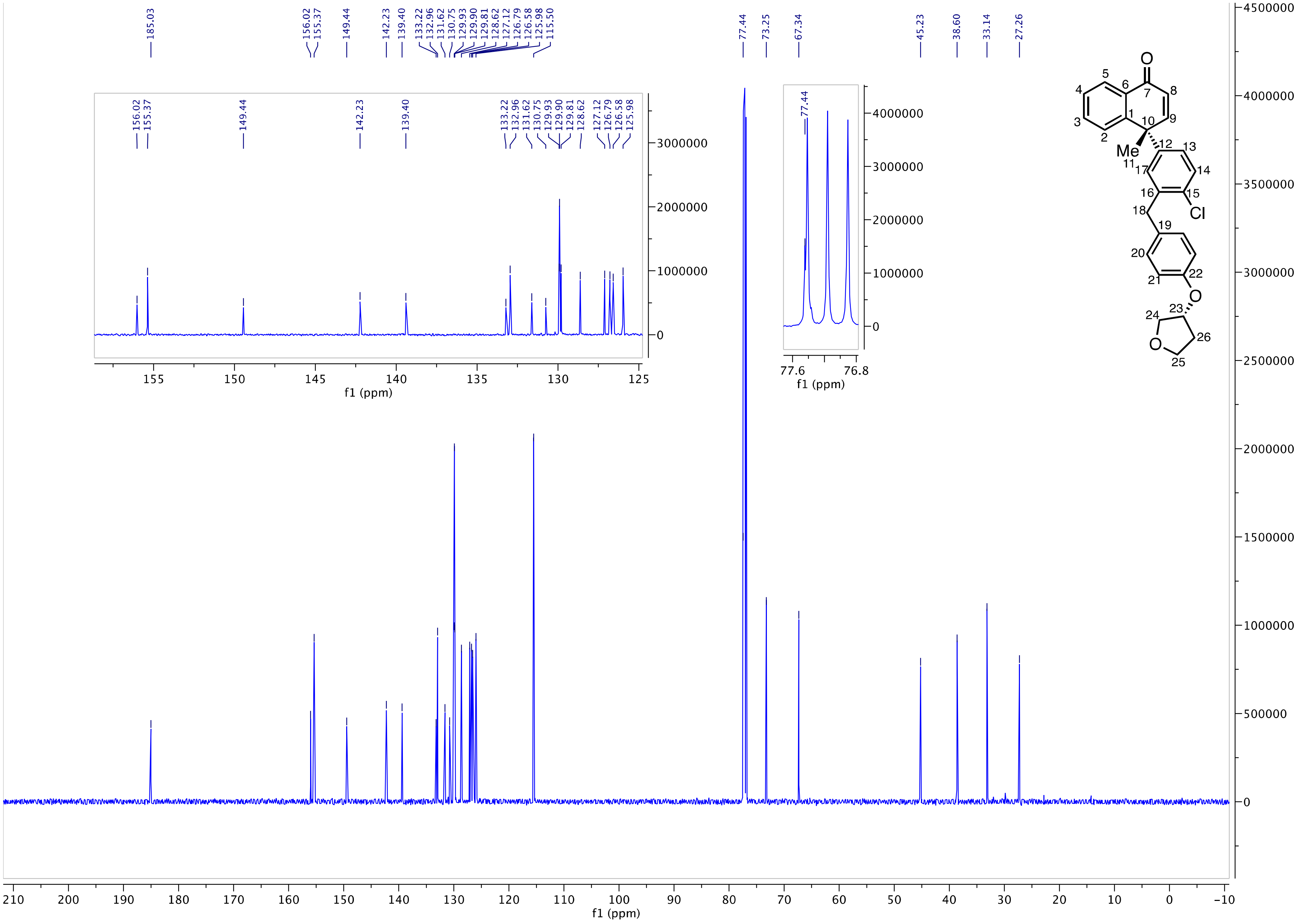

^{1}H NMR (CDCl$_3$):(4*R*,4'*R*)-4,4'-(1,4-phenylene)Bis(4-methylnaphthalen-1(4*H*)-one) (**3a**)

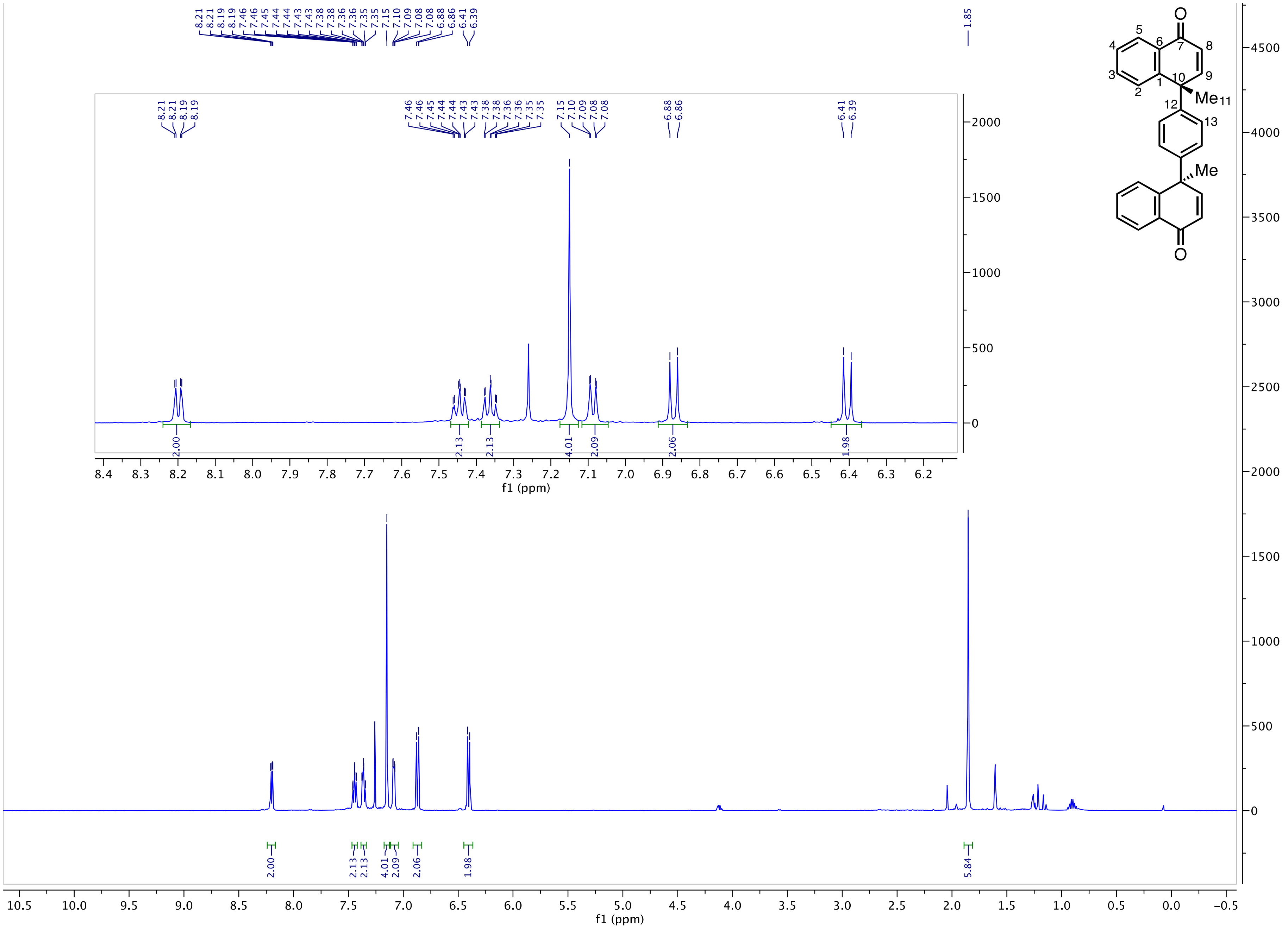

^{13}C NMR (CDCl$_3$):(4*R*,4'*R*)-4,4'-(1,4-phenylene)Bis(4-methylnaphthalen-1(4*H*)-one) (**3a**)

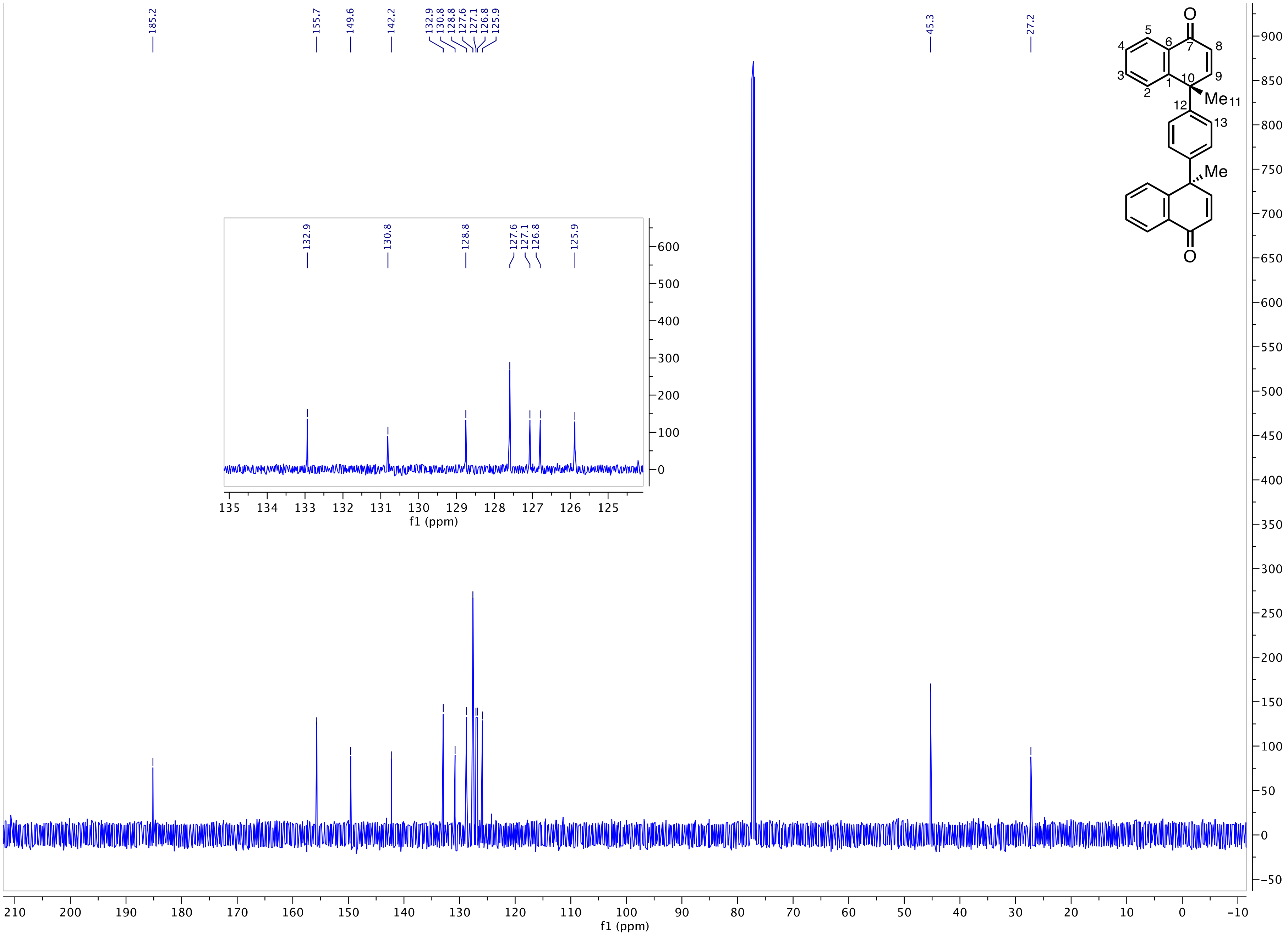

^{1}H NMR ($CDCl_3$): (4*R*,4'*R*)-4,4'-([1,1':4',1''-terphenyl]-4,4''-diyl)Bis(4-methylnaphthalen-1(4*H*)-one) (**3b**)

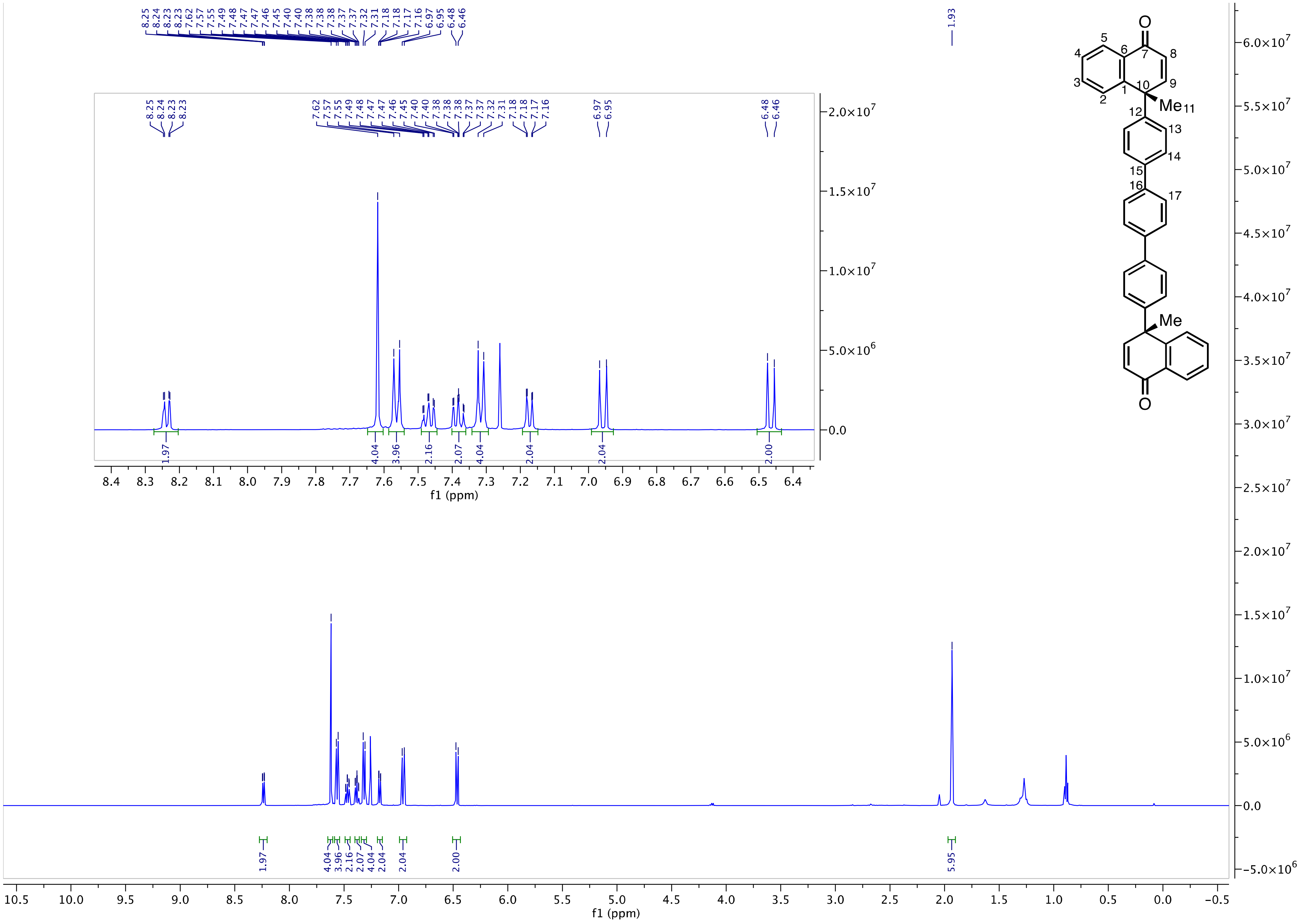

^{13}C NMR ($CDCl_3$): (4*R*,4'*R*)-4,4'-([1,1':4',1''-terphenyl]-4,4''-diyl)Bis(4-methylnaphthalen-1(4*H*)-one) (**3b**)

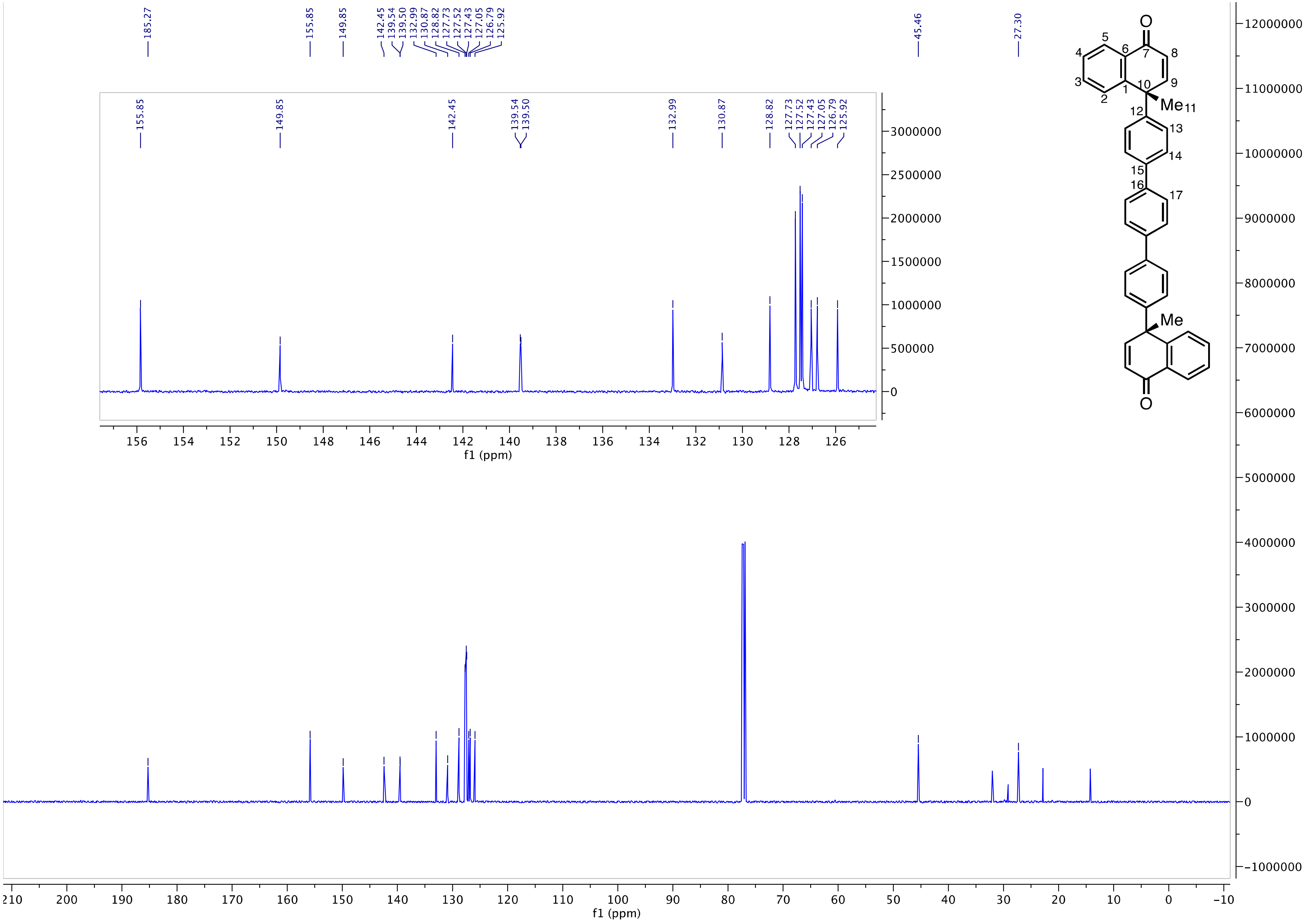

^{1}H NMR ($CDCl_3$): (4*R*,4'*R*)-4,4'-([1,1':3',1"-terphenyl]-4,4"-diyl)Bis(4-methylnaphthalen-1(4*H*)-one) (**3c**)

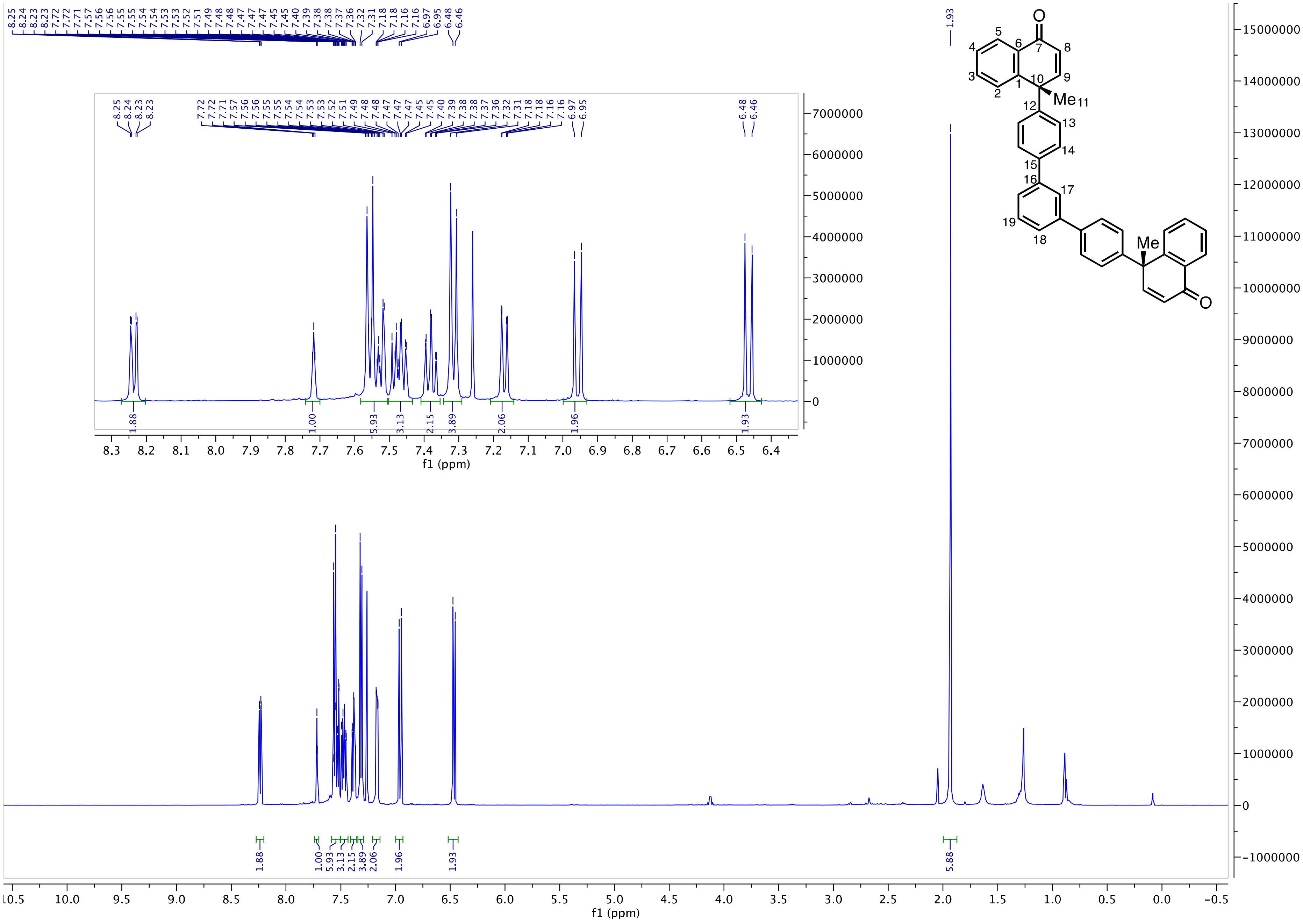

^{13}C NMR ($CDCl_3$): (4*R*,4'*R*)-4,4'-([1,1':3',1''-terphenyl]-4,4''-diyl)Bis(4-methylnaphthalen-1(4*H*)-one) (**3c**)

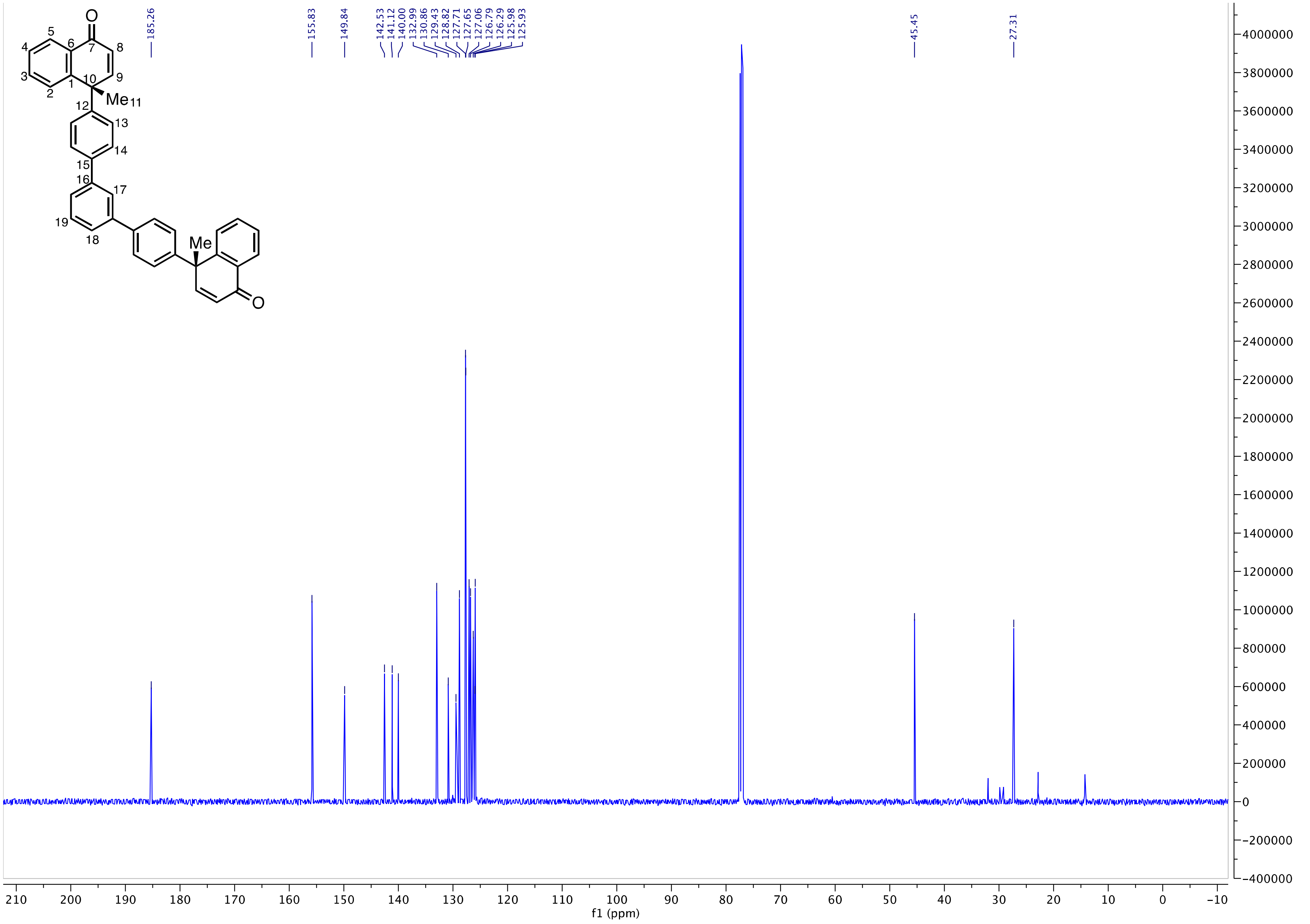

^{1}H NMR ($CDCl_3$): 1-Methyl-1-phenylnaphthalen-2(1H)-one (**S1**)

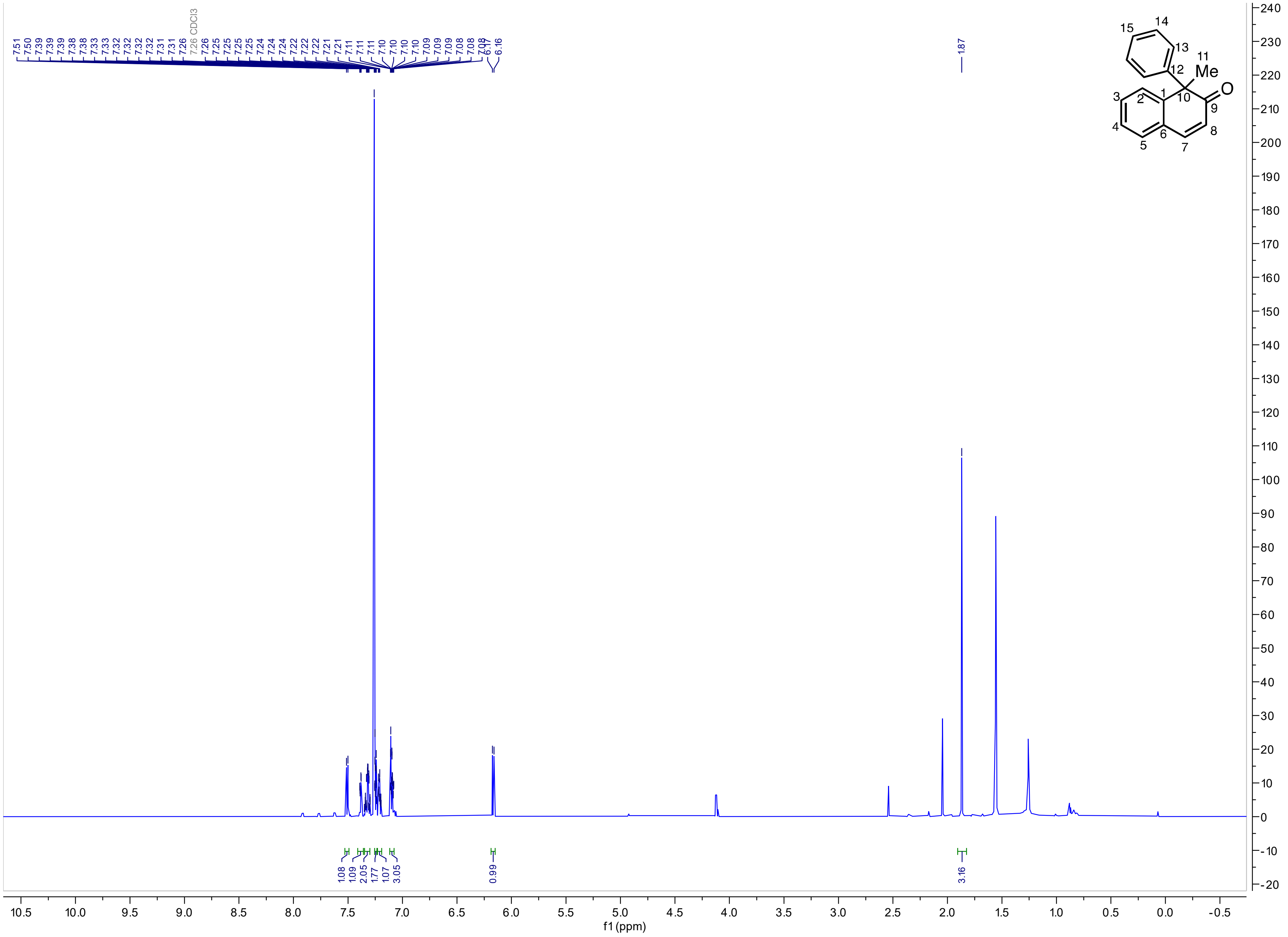

^{13}C NMR ($CDCl_3$): 1-Methyl-1-phenylnaphthalen-2(1H)-one (S1)

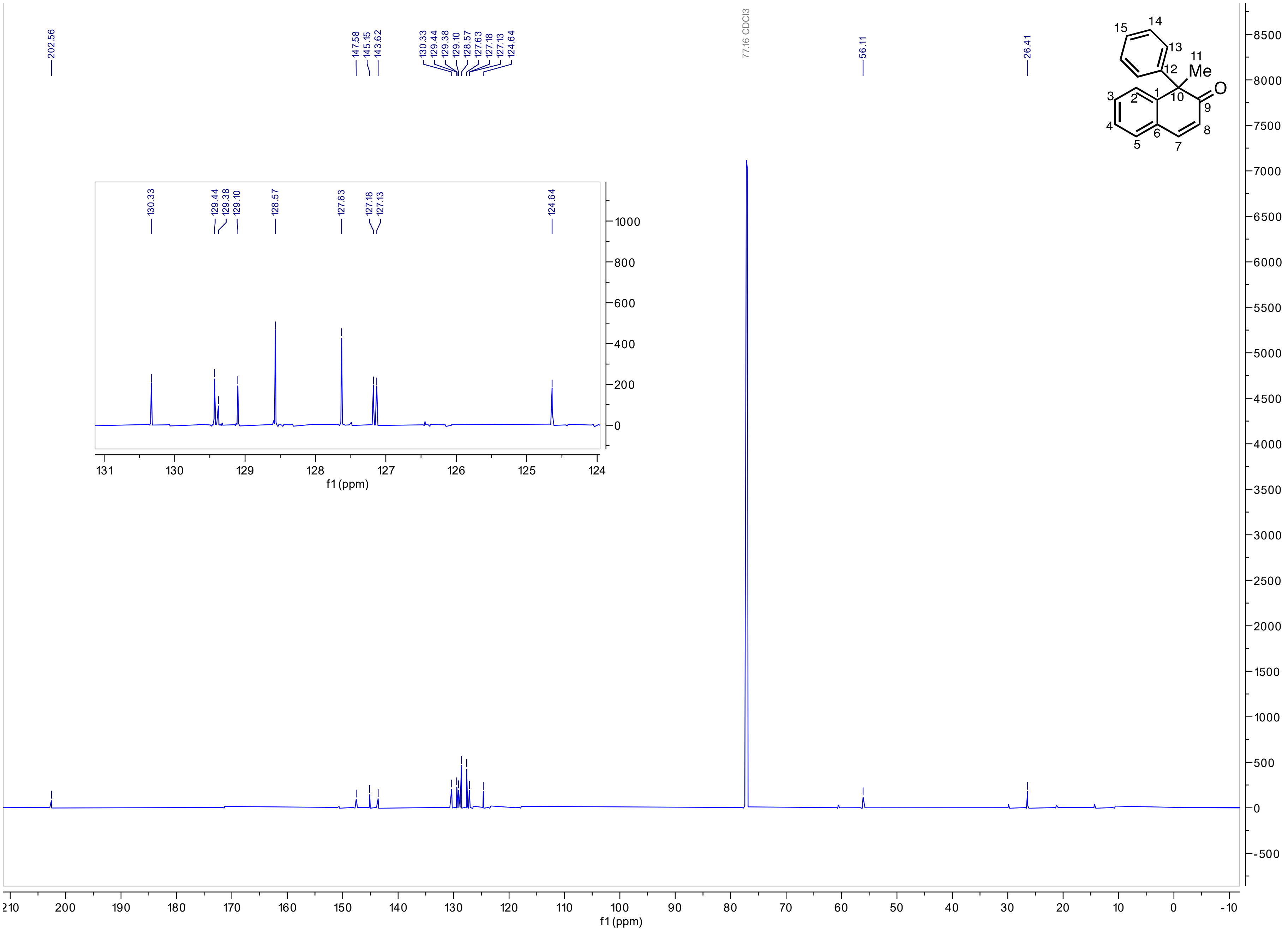

^{1}H NMR ($CDCl_3$): (3*S*,4*R*)-4-Methyl-4-phenyl-3-vinyl-3,4-dihydronaphthalen-1(2*H*)-one (**4a**)

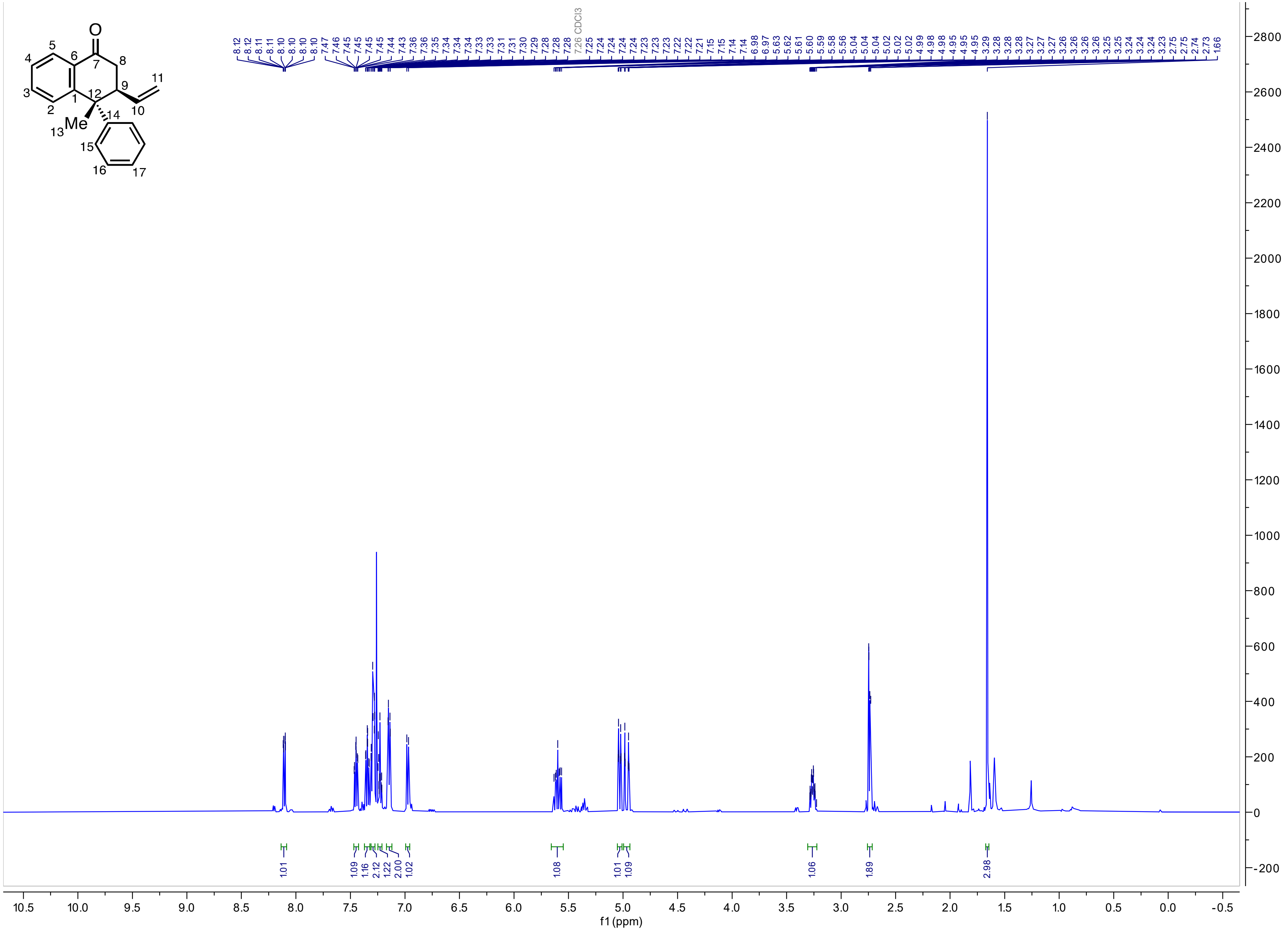

^{13}**C NMR** ($CDCl_3$): (3*S*,4*R*)-4-Methyl-4-phenyl-3-vinyl-3,4-dihydronaphthalen-1(2*H*)-one (**4a**)

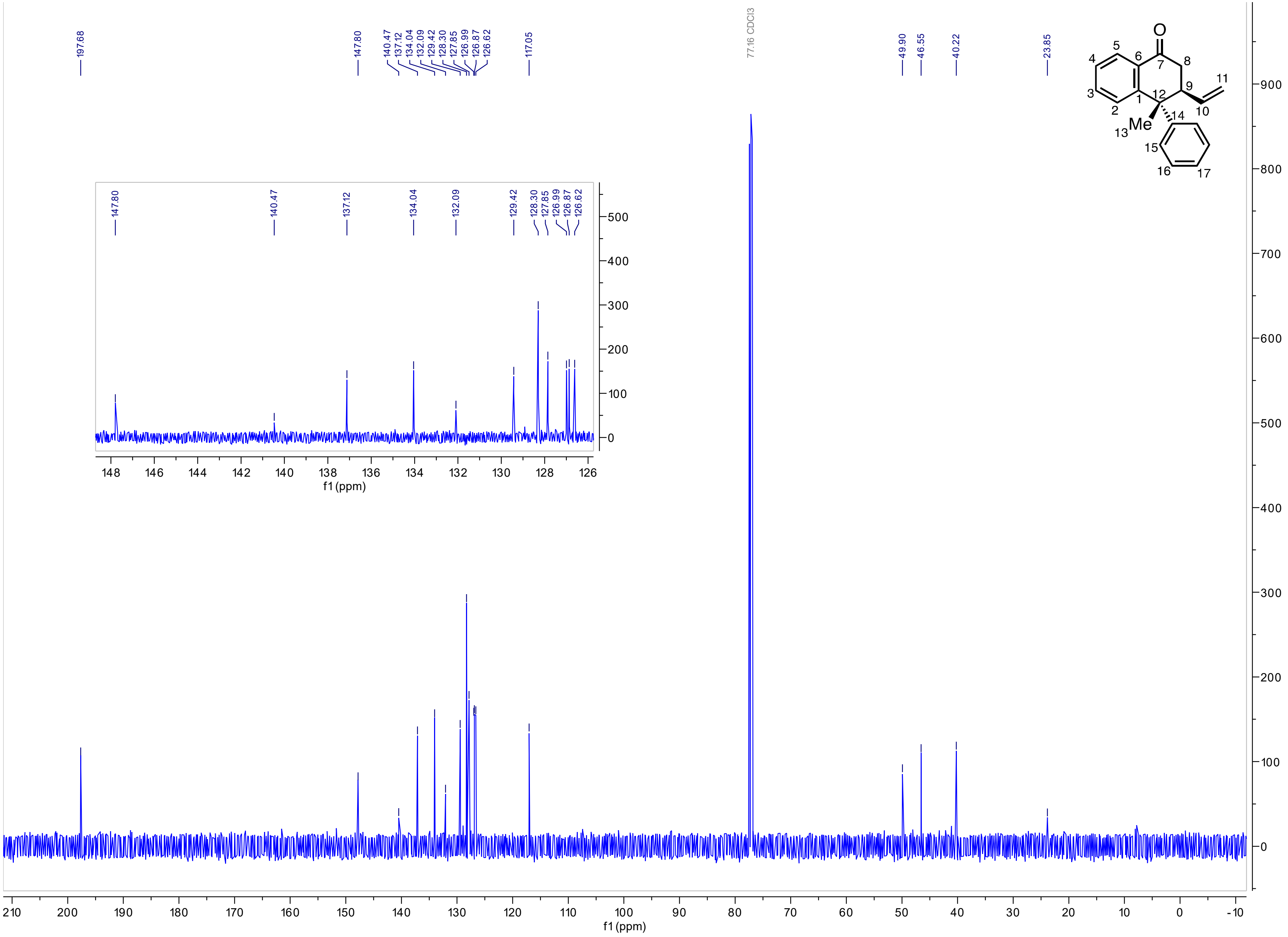

^{1}H NMR ($CDCl_3$): (1a*S*,7R,7a*S*)-7-Methyl-7-phenyl-7,7a-dihydronaphtho[2,3-*b*]oxiren-2(1a*H*)-one (**4b**)

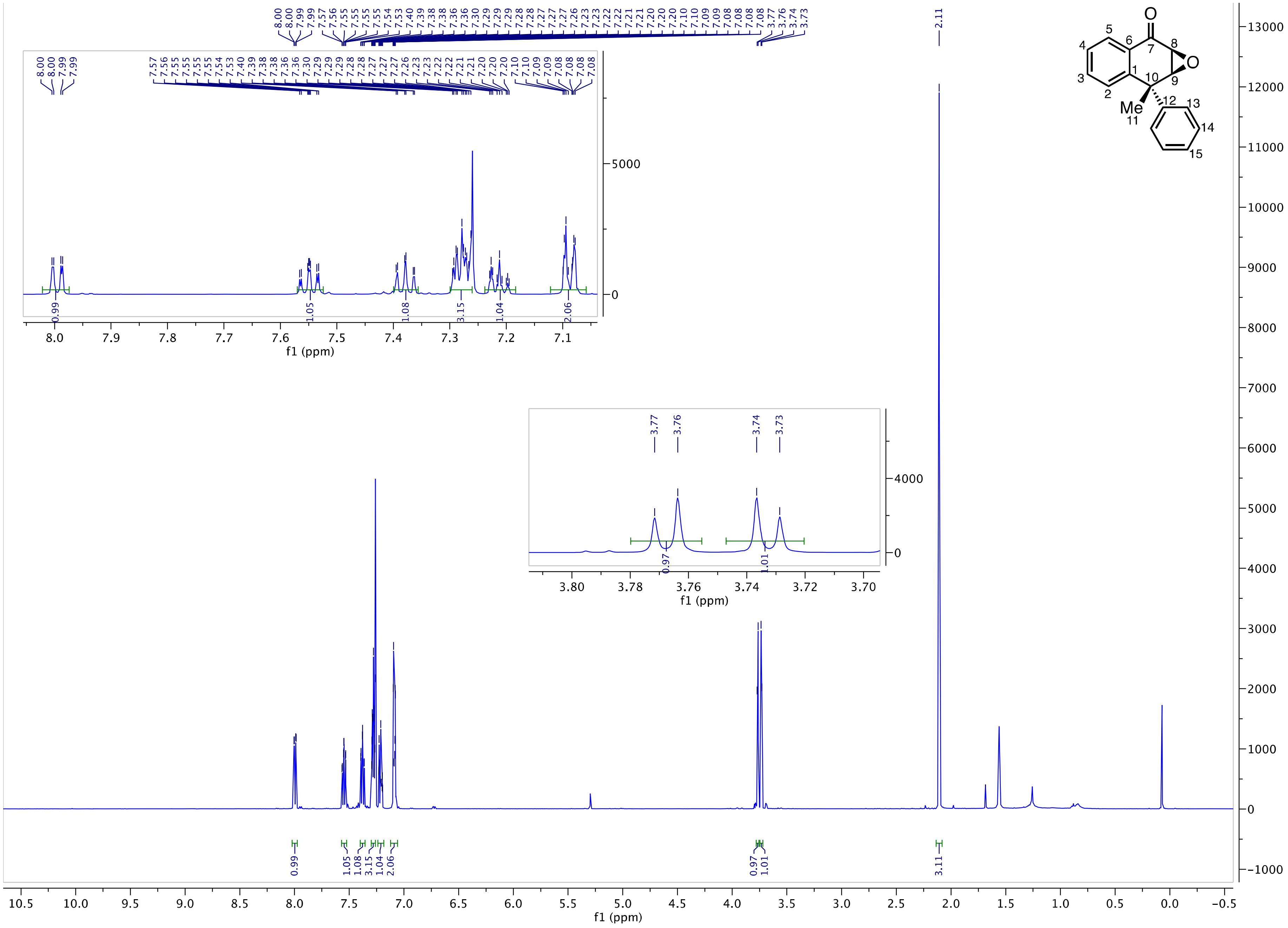

^{13}C **NMR** ($CDCl_3$): (1a*S*,7R,7a*S*)-7-Methyl-7-phenyl-7,7a-dihydronaphtho[2,3-*b*]oxiren-2(1a*H*)-one (**4b**)

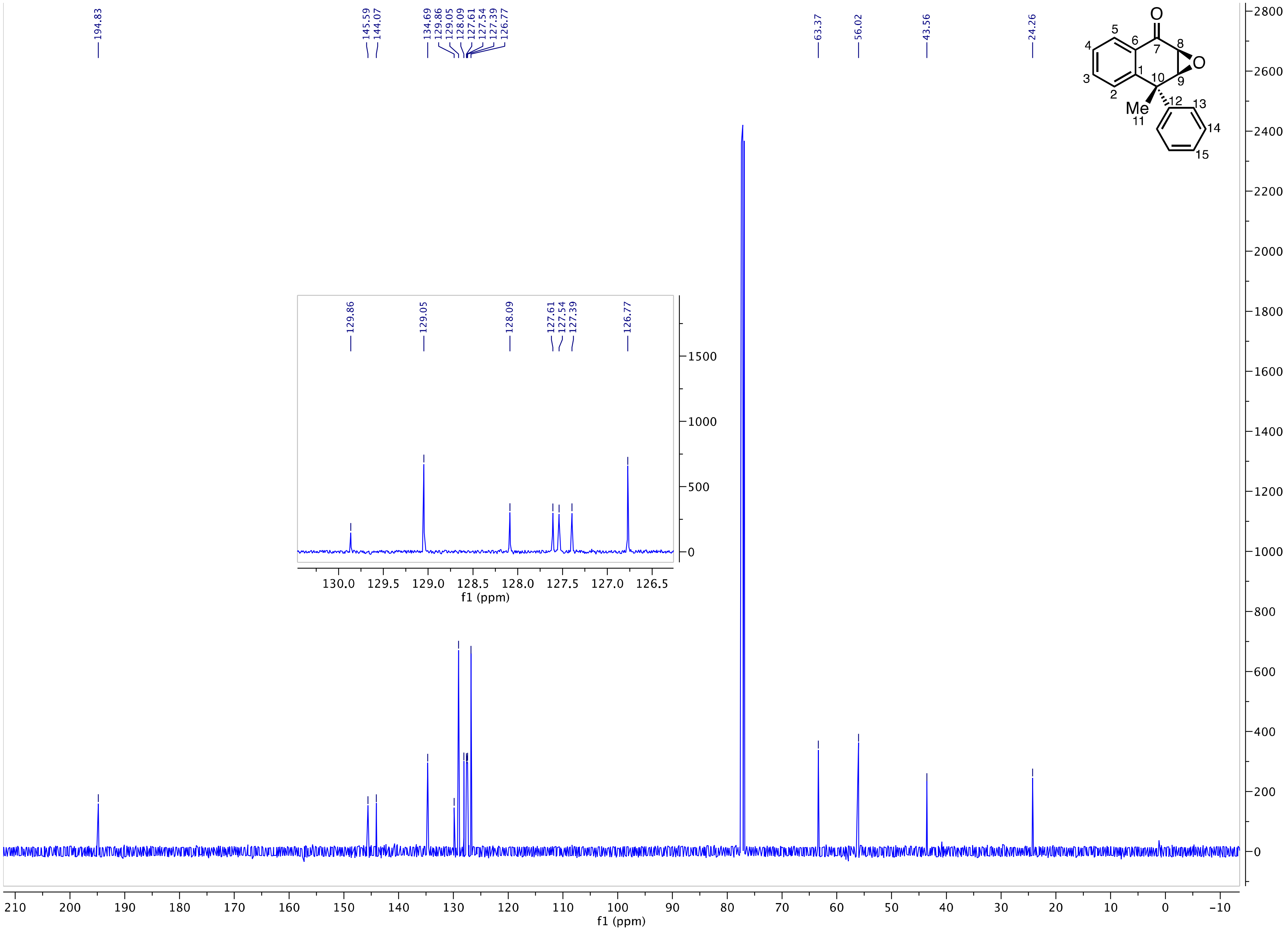

^{1}H NMR ($CDCl_3$): (*S*)-4-(3,4-dichlorophenyl)-4-Methylnaphthalen-1(4*H*)-one (**5a**)

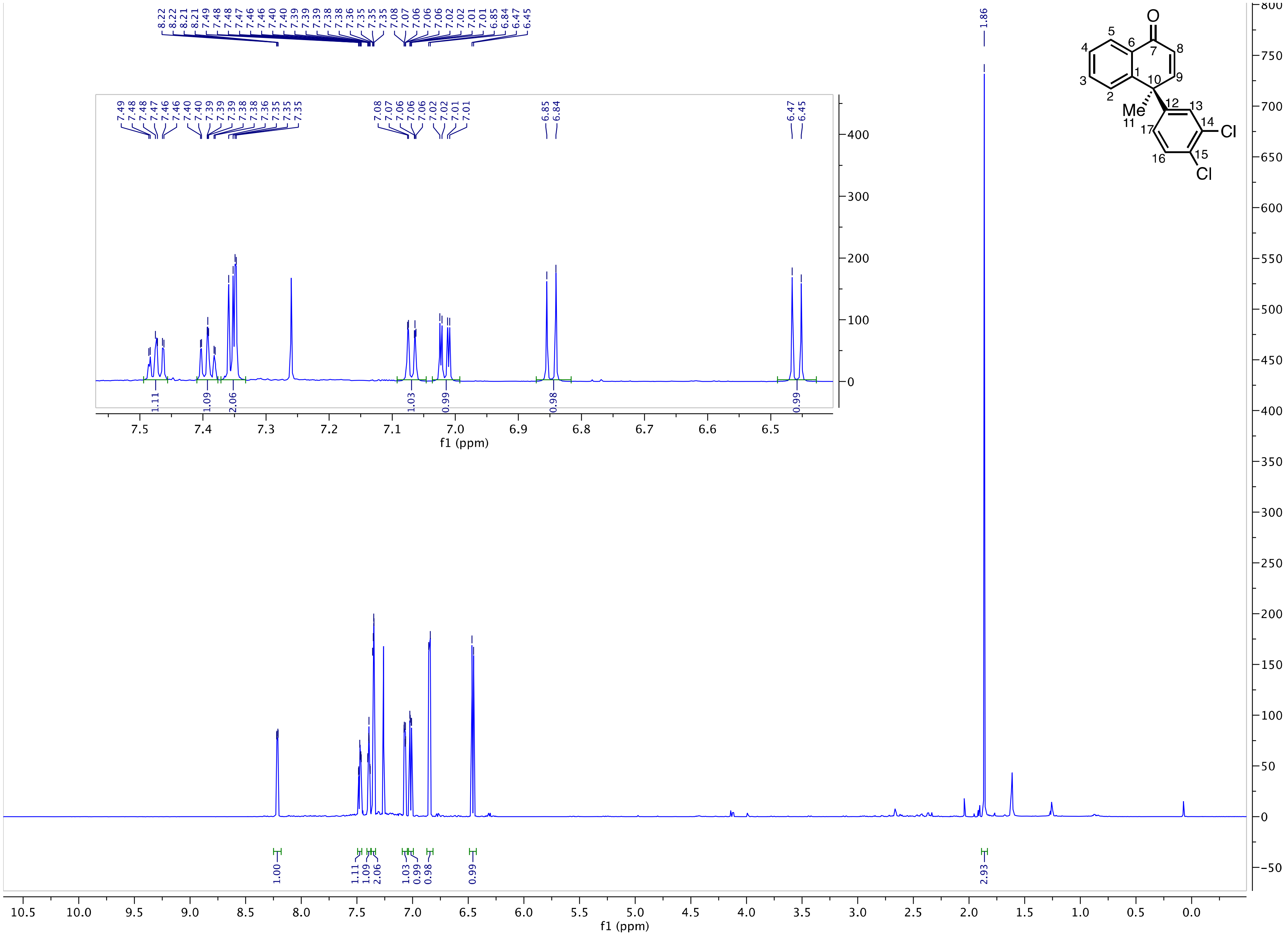

^{13}C NMR ($CDCl_3$): (*S*)-4-(3,4-dichlorophenyl)-4-Methylnaphthalen-1(4*H*)-one (**5a**)

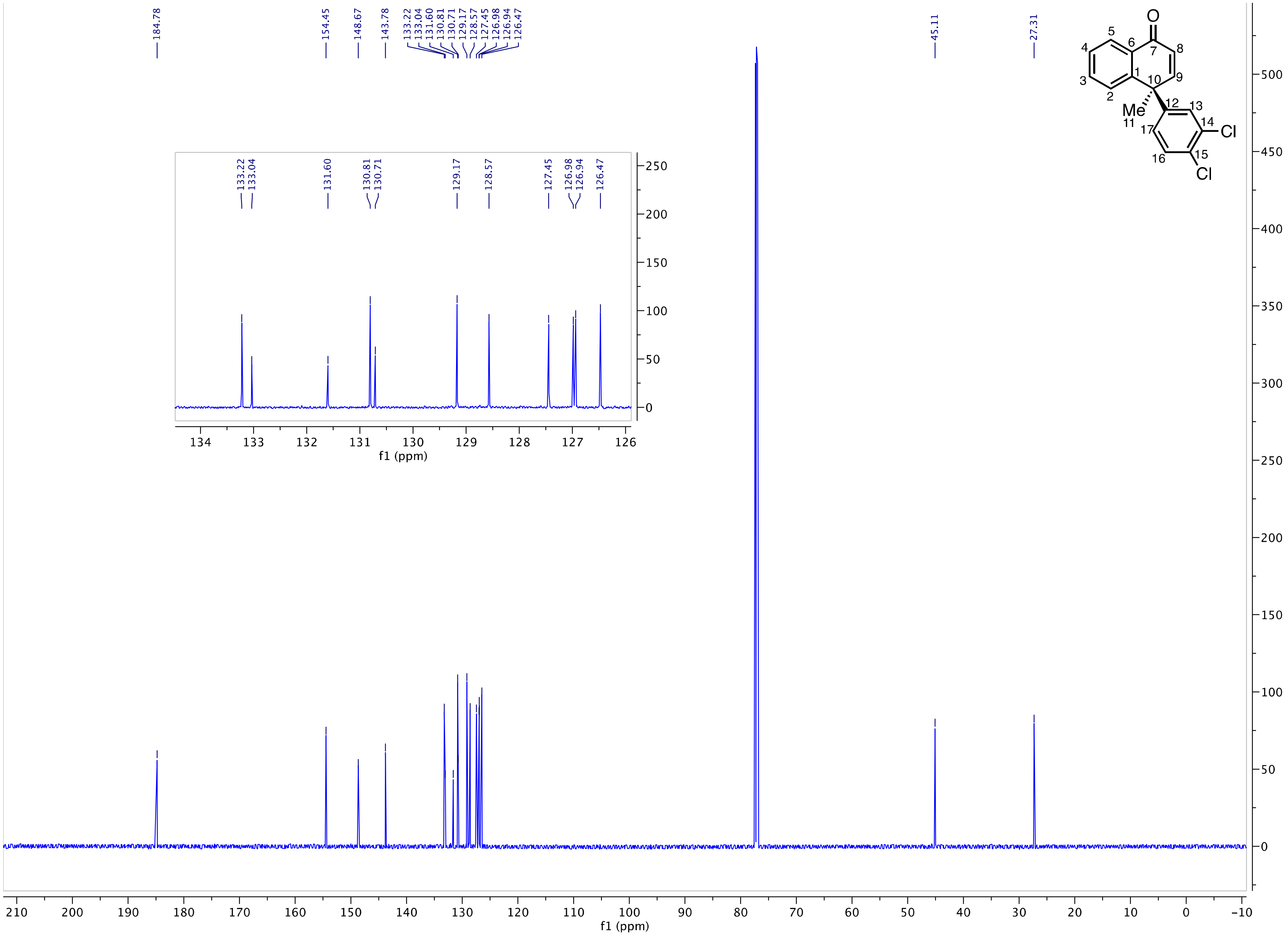

^{1}H NMR ($CDCl_3$): (*S*)-4-(3,4-dichlorophenyl)-4-Methyl-3,4-dihydronaphthalen-1(2*H*)-one (**5b**)

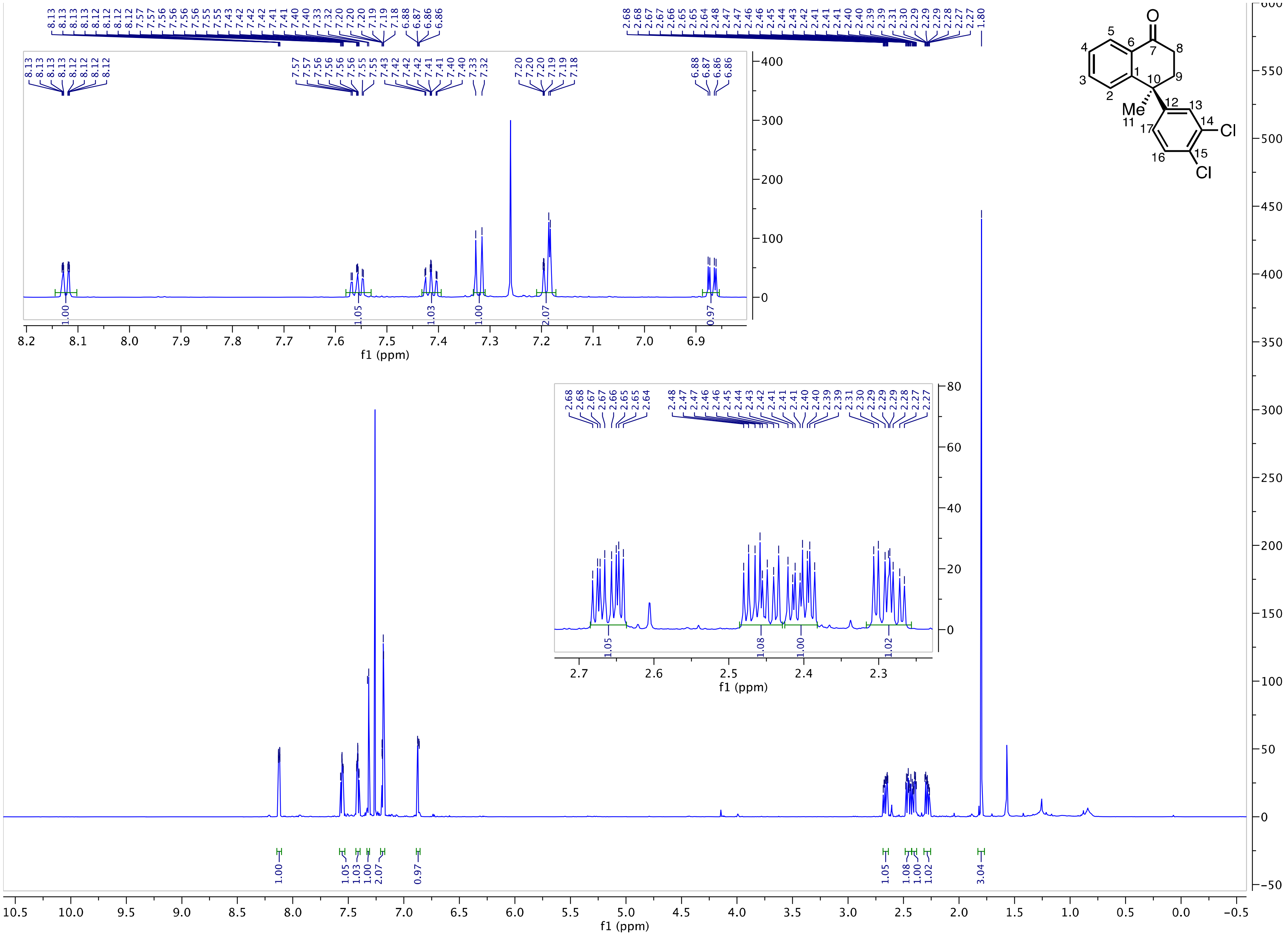

^{13}C NMR ($CDCl_3$): (*S*)-4-(3,4-dichlorophenyl)-4-Methyl-3,4-dihydronaphthalen-1(2*H*)-one (**5b**)

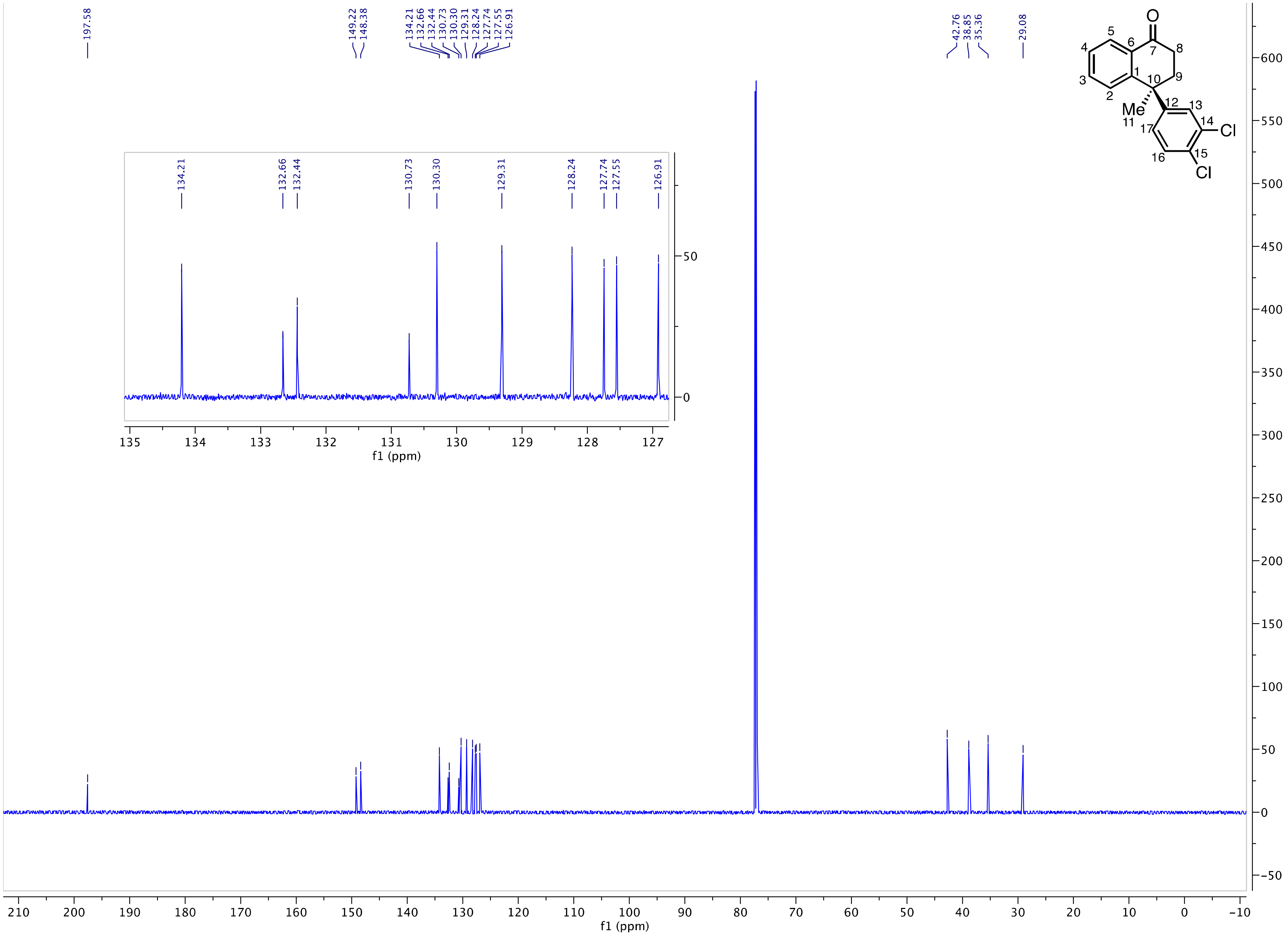

^{1}**H NMR** ($CDCl_3$): (1*S*,4*S*)-4-(3,4-dichlorophenyl)-4-Methyl-1,2,3,4-tetrahydronaphthalen-1-ol (**5cb**)

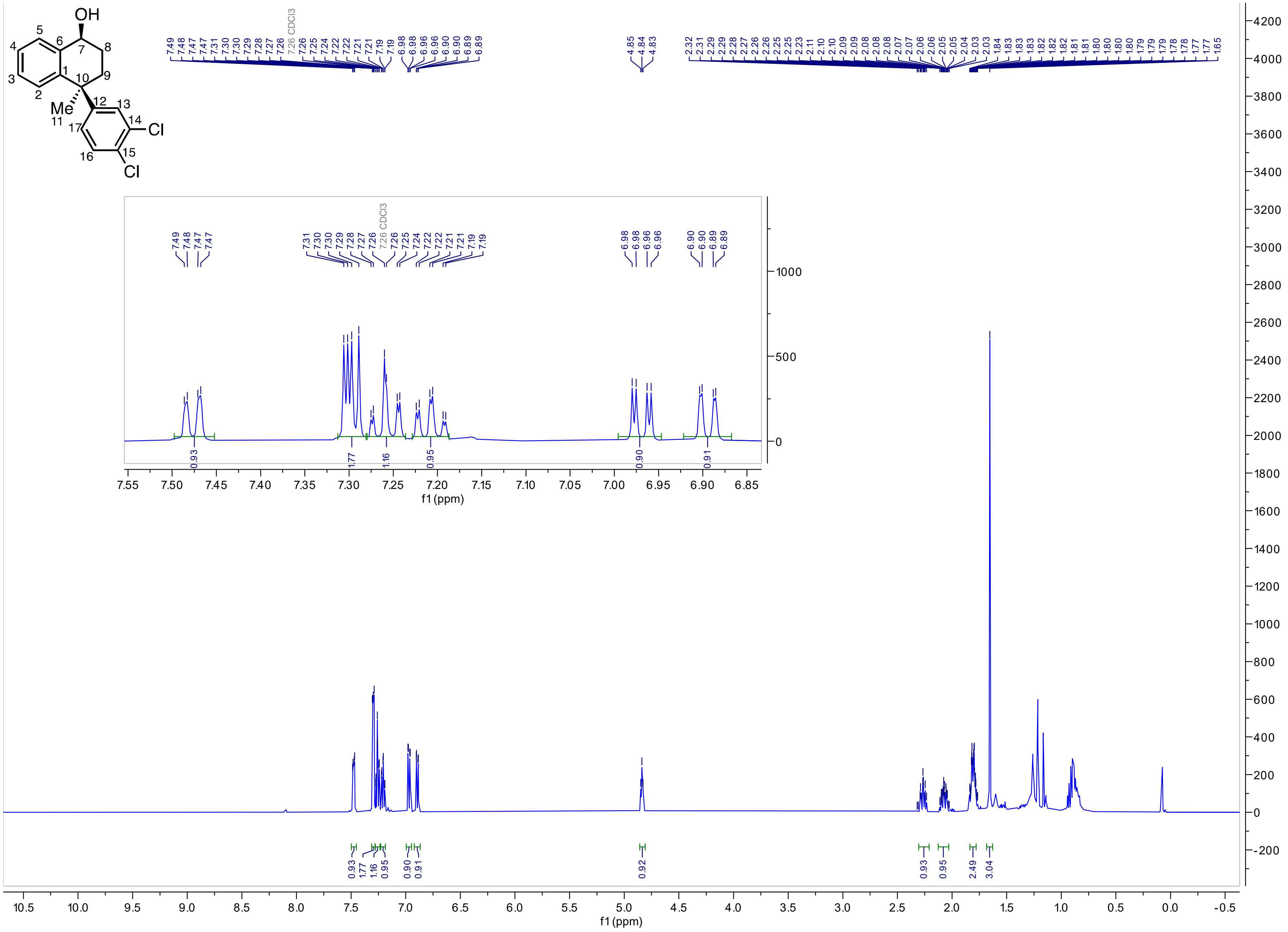

^{13}**C NMR** ($CDCl_3$): (1*S*,4*S*)-4-(3,4-dichlorophenyl)-4-Methyl-1,2,3,4-tetrahydronaphthalen-1-ol (**5cb**)

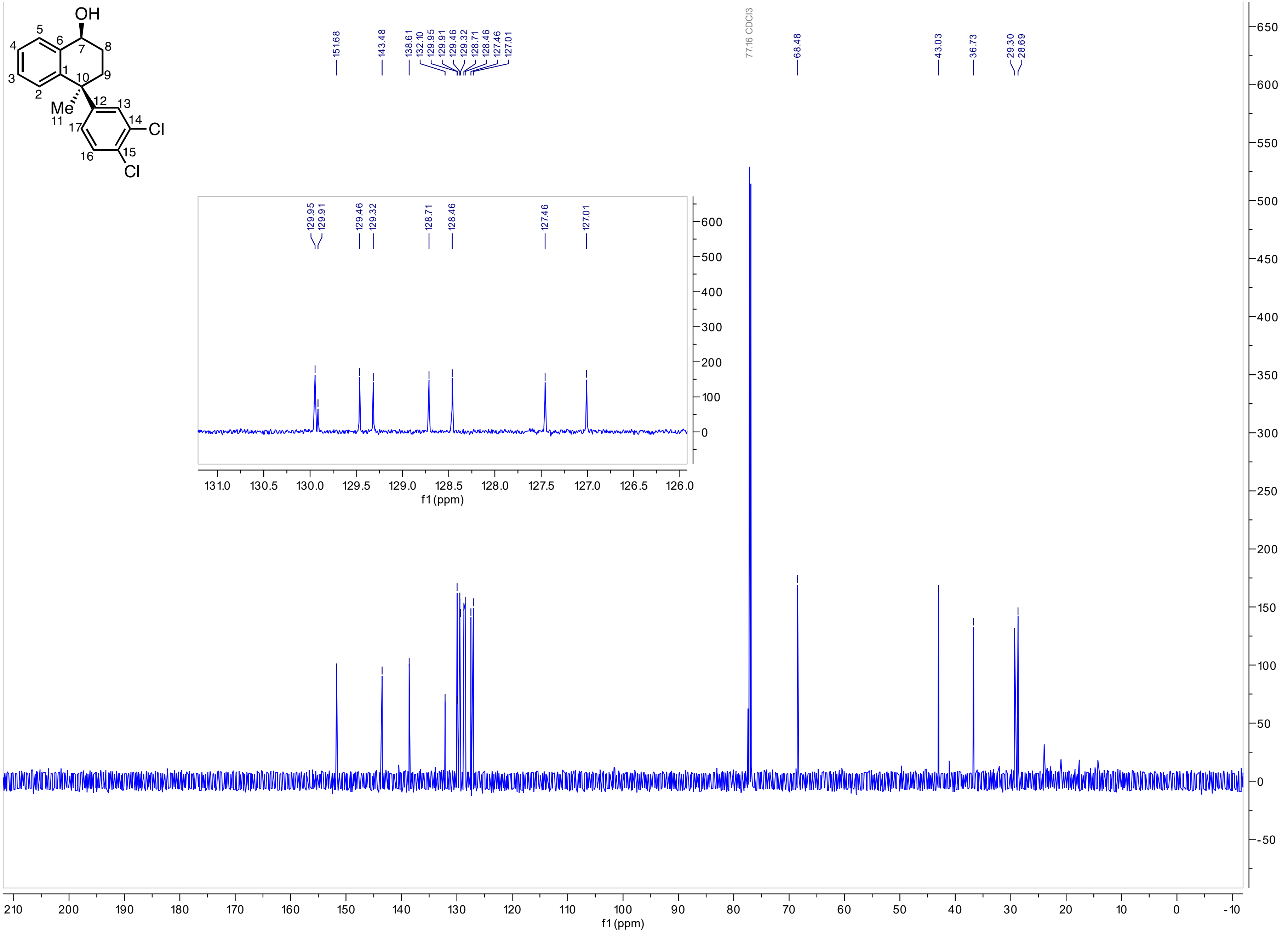

^{1}H NMR ($CDCl_3$): (1*R*,4*S*)-4-(3,4-dichlorophenyl)-4-Methyl-1,2,3,4-tetrahydronaphthalen-1-ol (**5ca**)

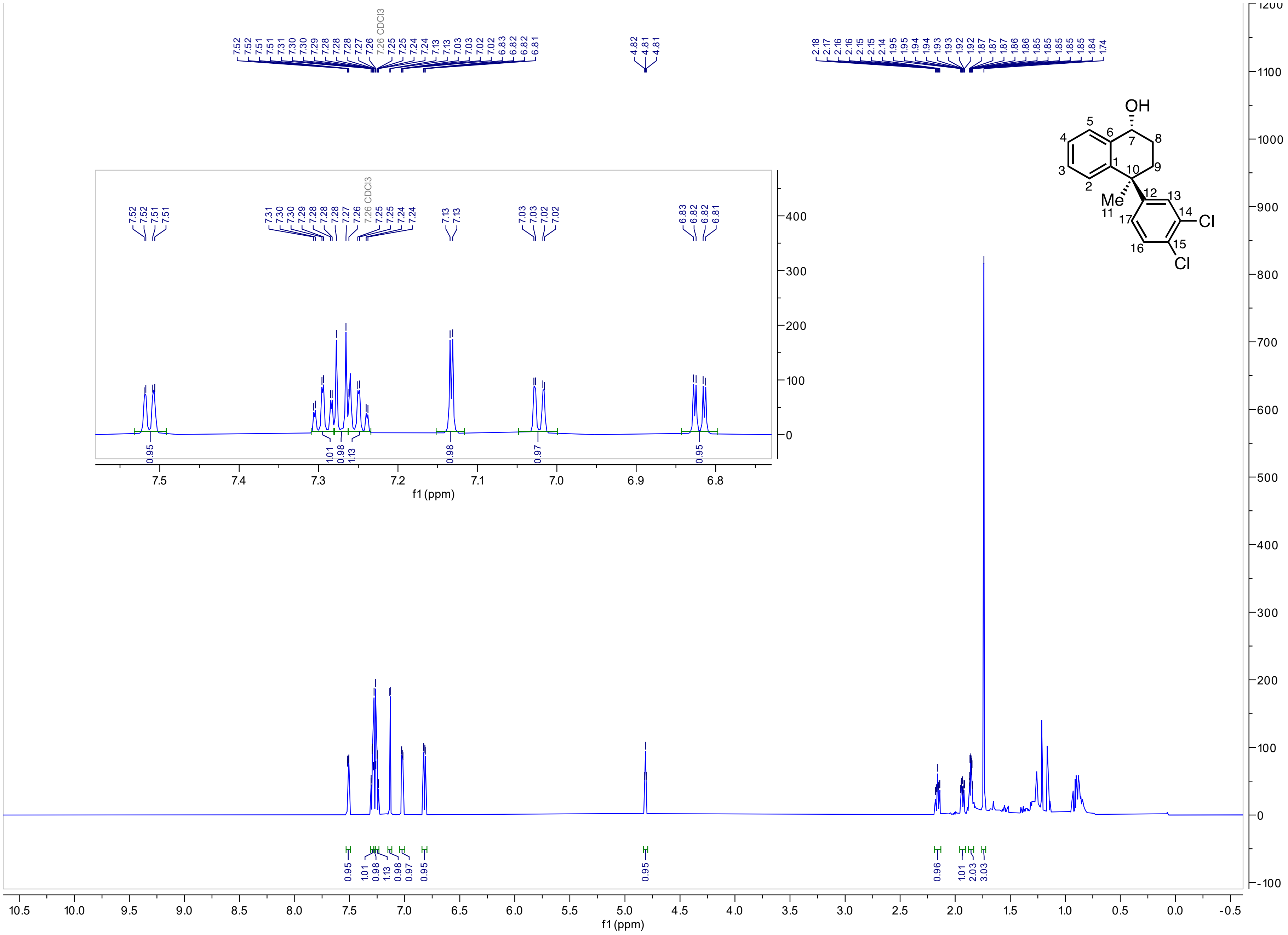

^{13}C NMR (CDCl$_3$): (1*R*,4*S*)-4-(3,4-dichlorophenyl)-4-Methyl-1,2,3,4-tetrahydronaphthalen-1-ol (**5ca**)

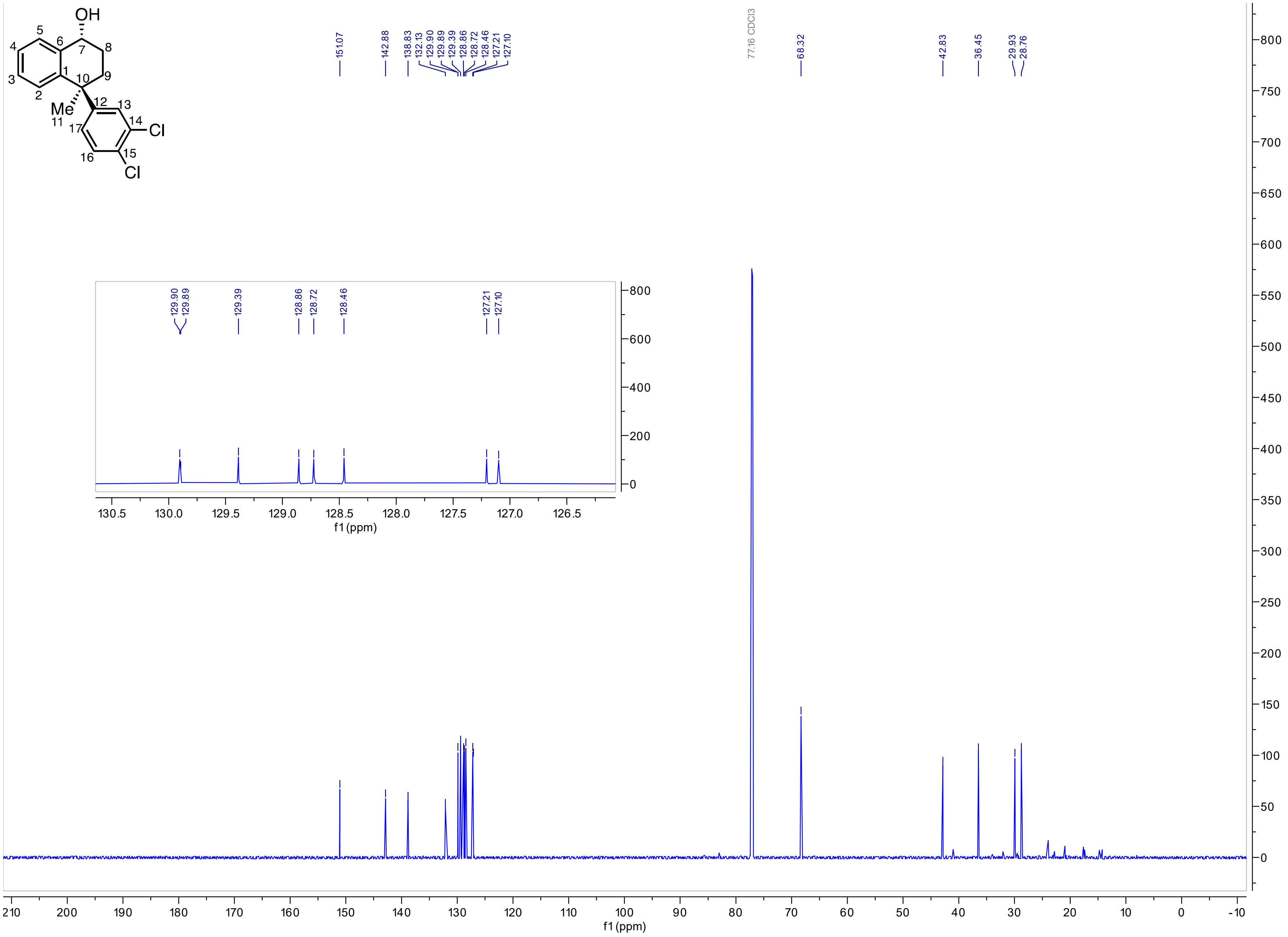

^{1}H NMR ($CDCl_3$): (1*S*,4*R*)-4-Azido-1-(3,4-dichlorophenyl)-1-methyl-1,2,3,4-tetrahydronaphthalene (**S19b**)

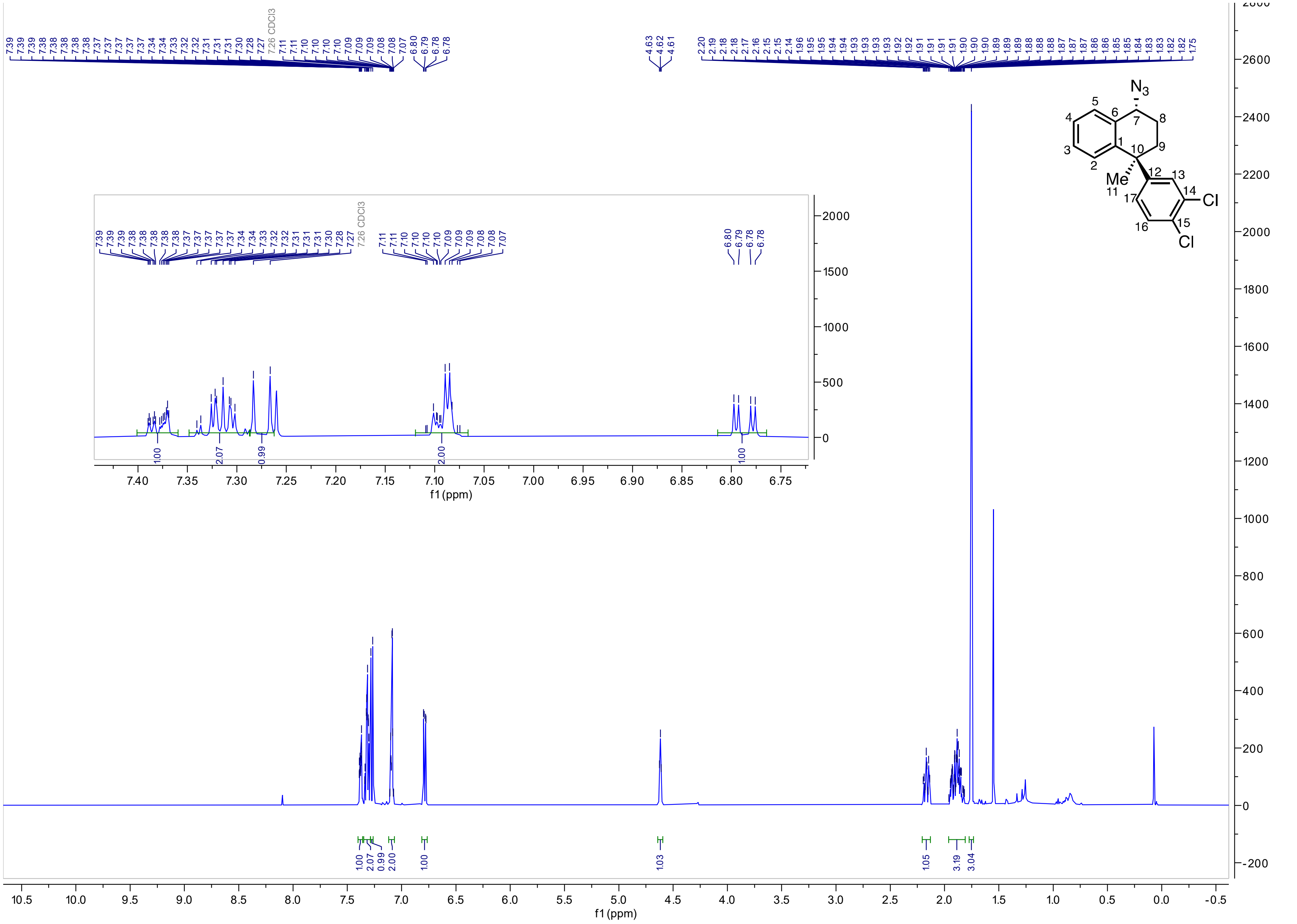

^{13}C NMR ($CDCl_3$): (1*S*,4*R*)-4-Azido-1-(3,4-dichlorophenyl)-1-methyl-1,2,3,4-tetrahydronaphthalene (**S19b**)

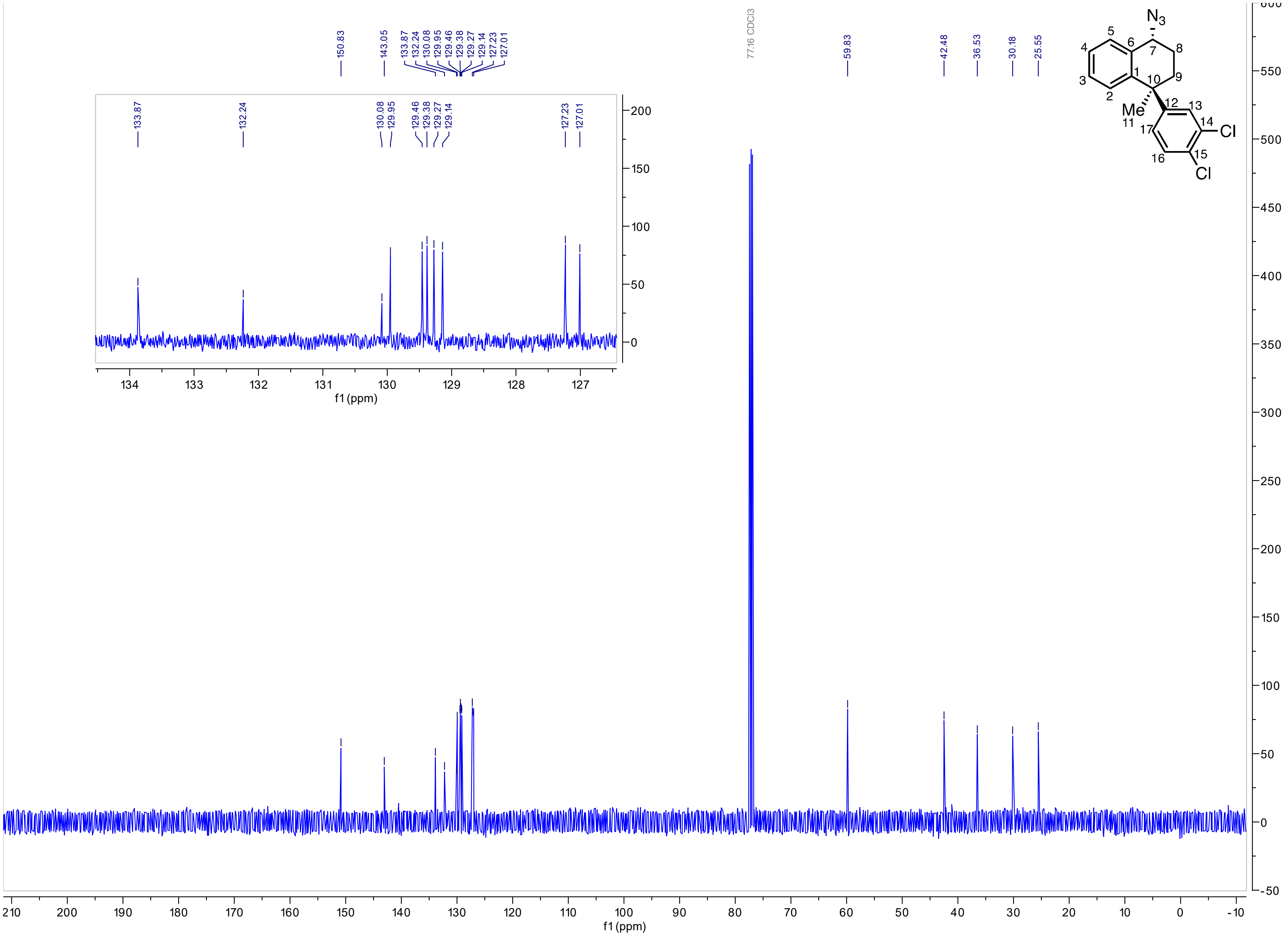

^{1}H NMR ($CDCl_3$): (1*S*,4*S*)-4-Azido-1-(3,4-dichlorophenyl)-1-methyl-1,2,3,4-tetrahydronaphthalene (**S19a**)

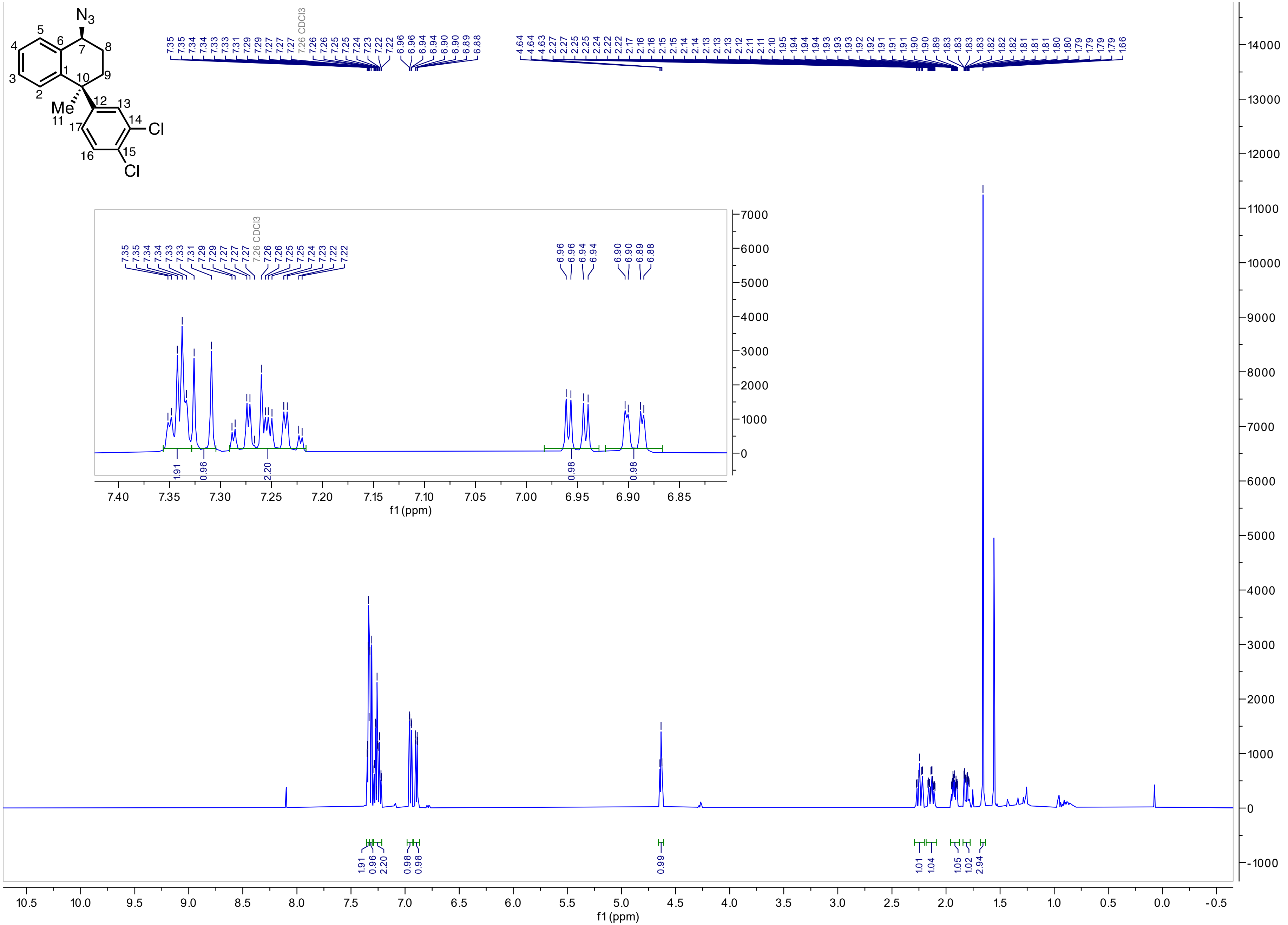

^{13}C NMR ($CDCl_3$): (1*S*,4*S*)-4-Azido-1-(3,4-dichlorophenyl)-1-methyl-1,2,3,4-tetrahydronaphthalene (**S19a**)

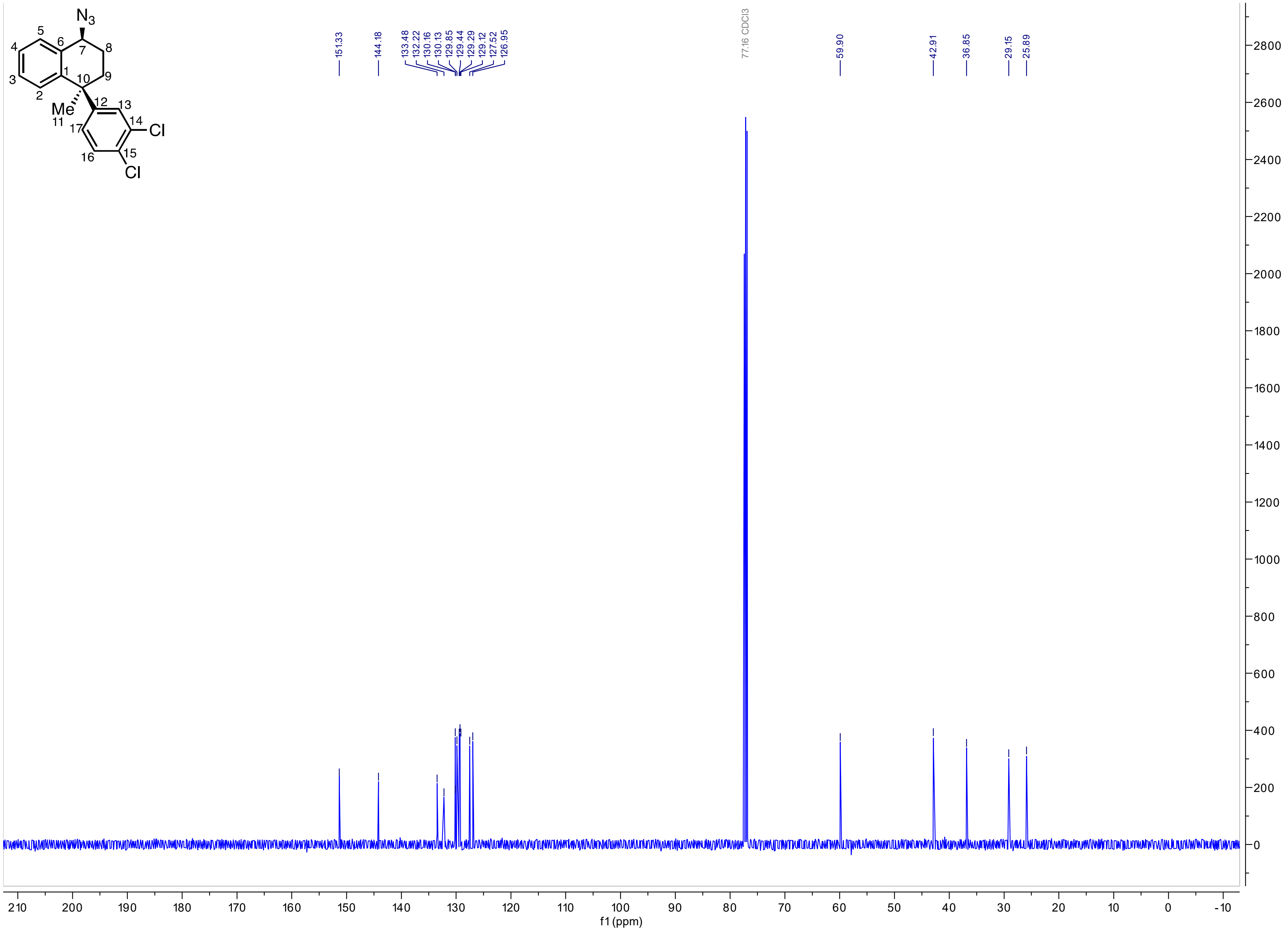

^{1}H NMR ($CDCl_3$): (1*R*,4*S*)-4-(3,4-dichlorophenyl)-4-Methyl-1,2,3,4-tetrahydronaphthalen-1-amine [“Dasotraline variant] (**5db**)

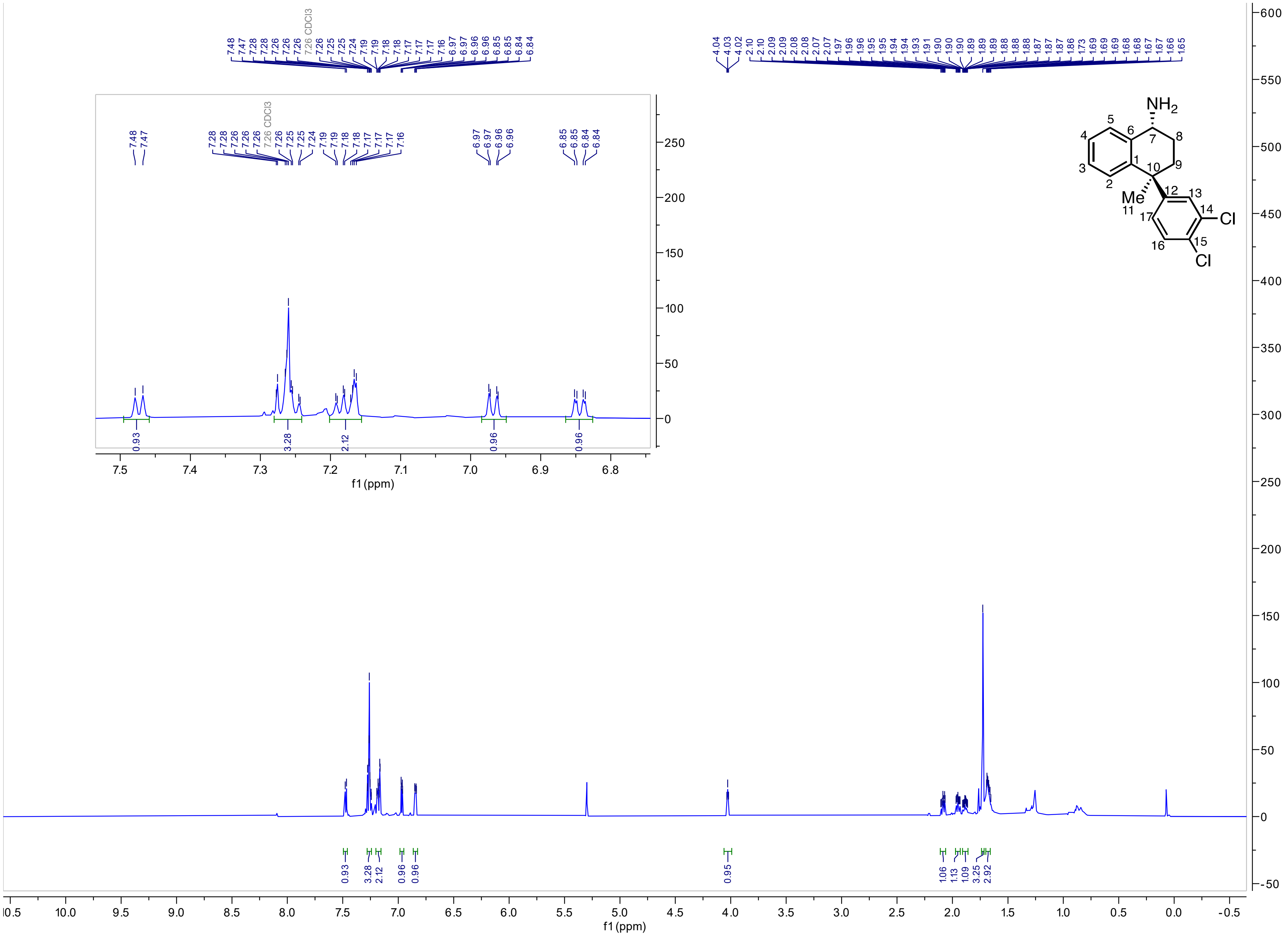

^{13}C NMR (CDCl$_3$): (1*R*,4*S*)-4-(3,4-dichlorophenyl)-4-Methyl-1,2,3,4-tetrahydronaphthalen-1-amine [“Dasotraline variant] (**5db**)

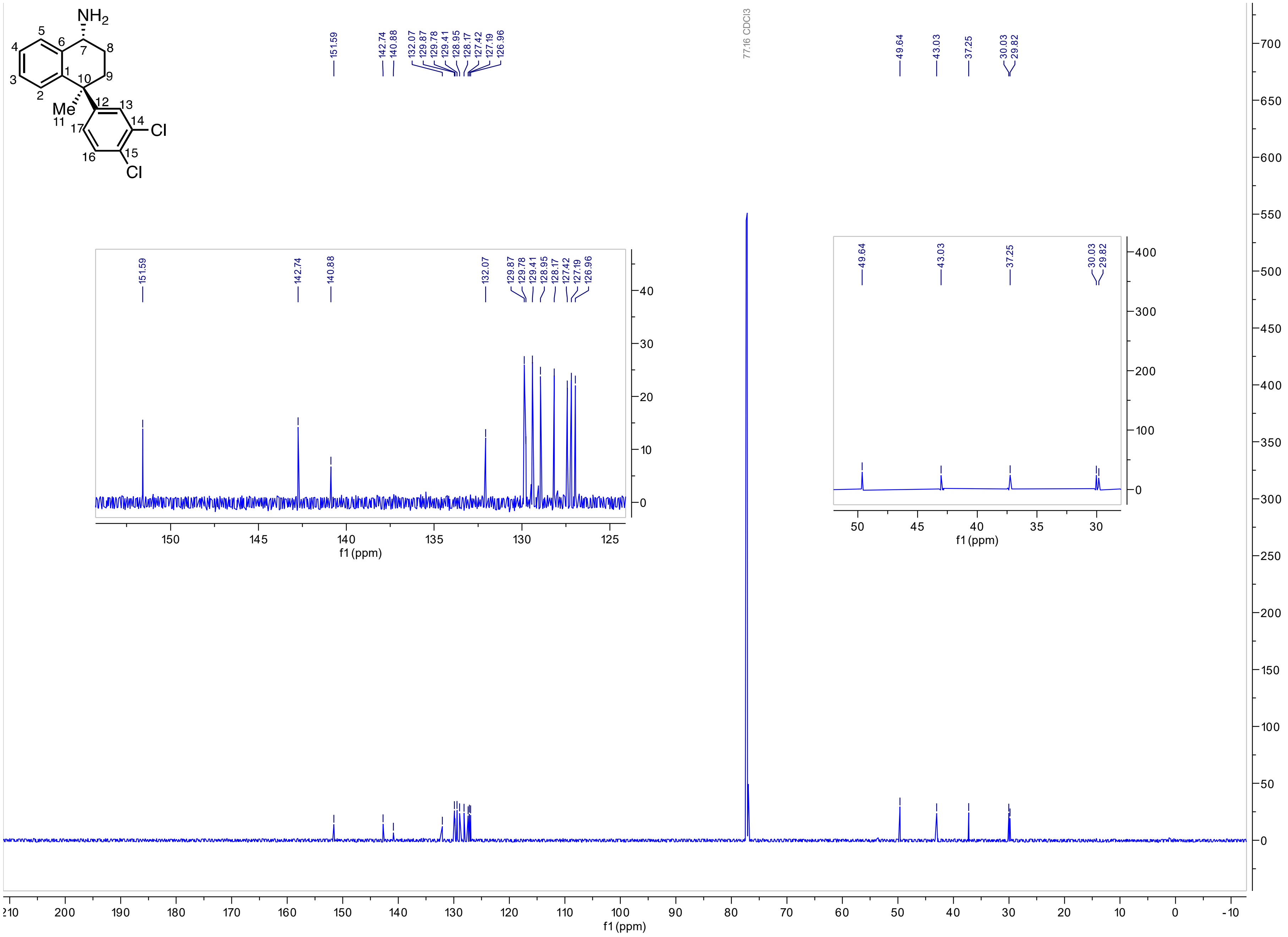

^{1}H NMR ($CDCl_3$): (1*S*,4*S*)-4-(3,4-dichlorophenyl)-4-Methyl-1,2,3,4-tetrahydronaphthalen-1-amine ["Desmethylsertraline variant"] (**5da**)

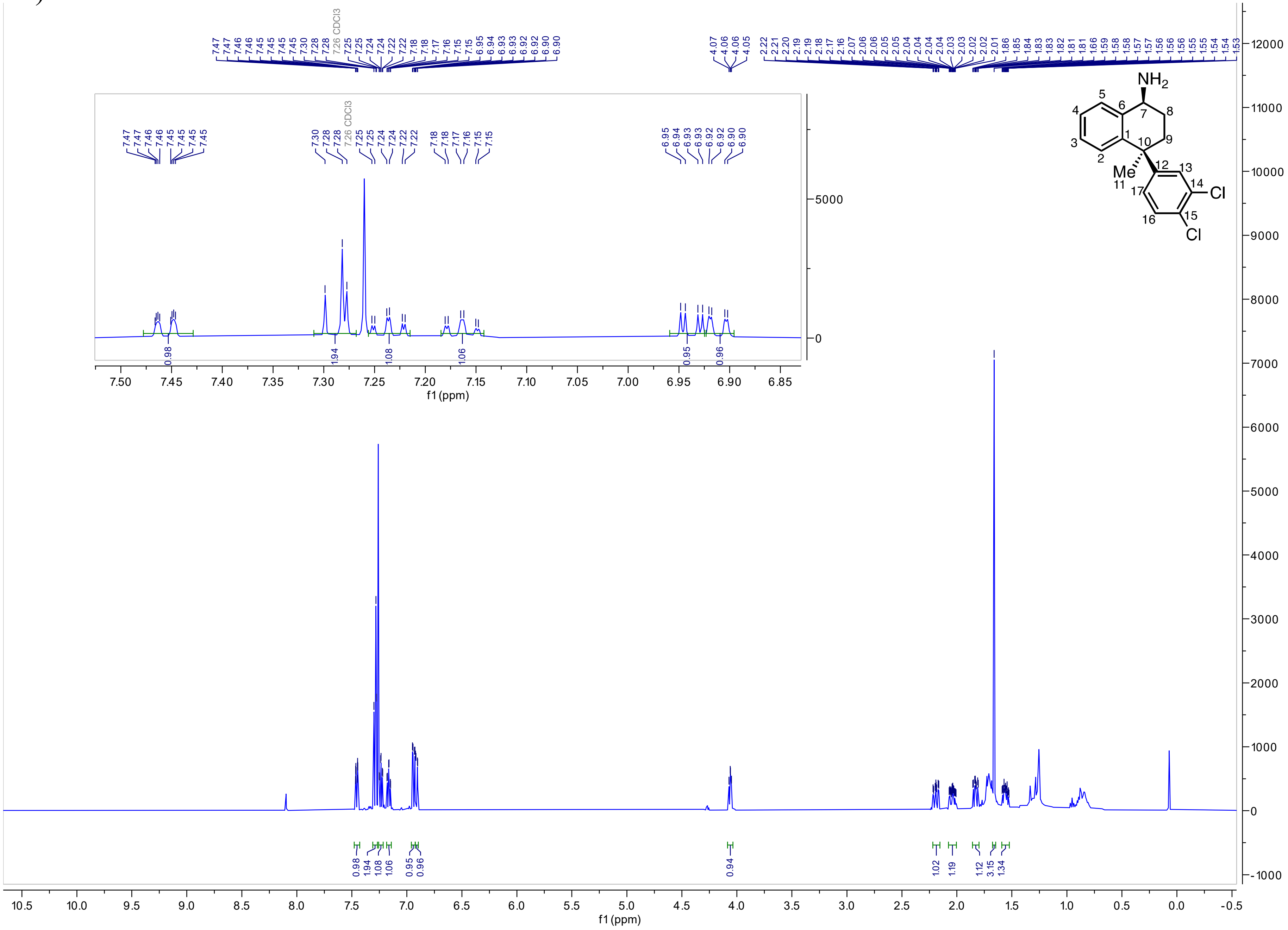

^{13}C NMR ($CDCl_3$): (1*S*,4*S*)-4-(3,4-dichlorophenyl)-4-Methyl-1,2,3,4-tetrahydronaphthalen-1-amine [“Desmethylsertraline variant”] (**5da**)

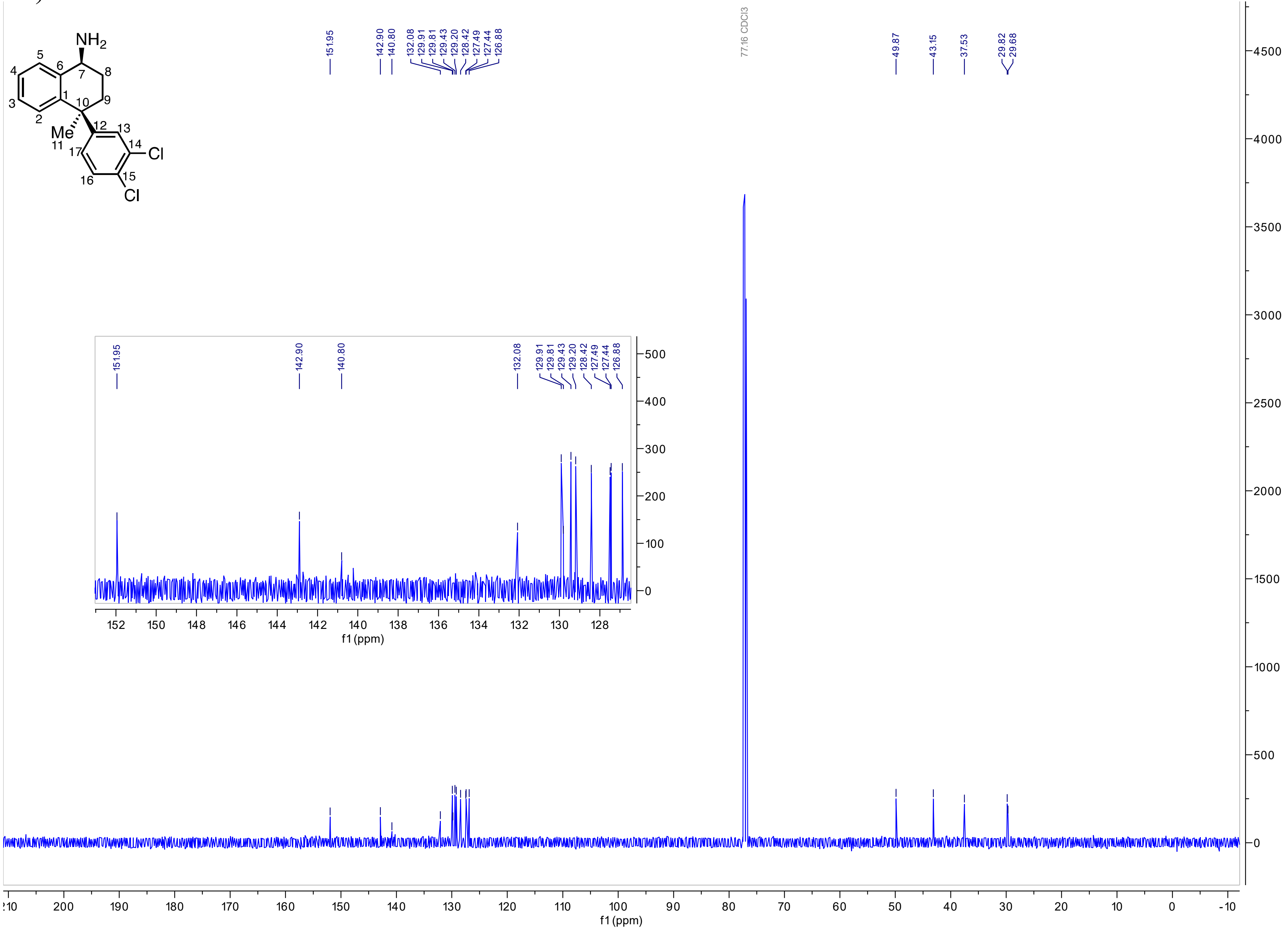

^{1}H NMR ($CDCl_3$): 5-Bromo-4-iodo-2-methylphenol (8)

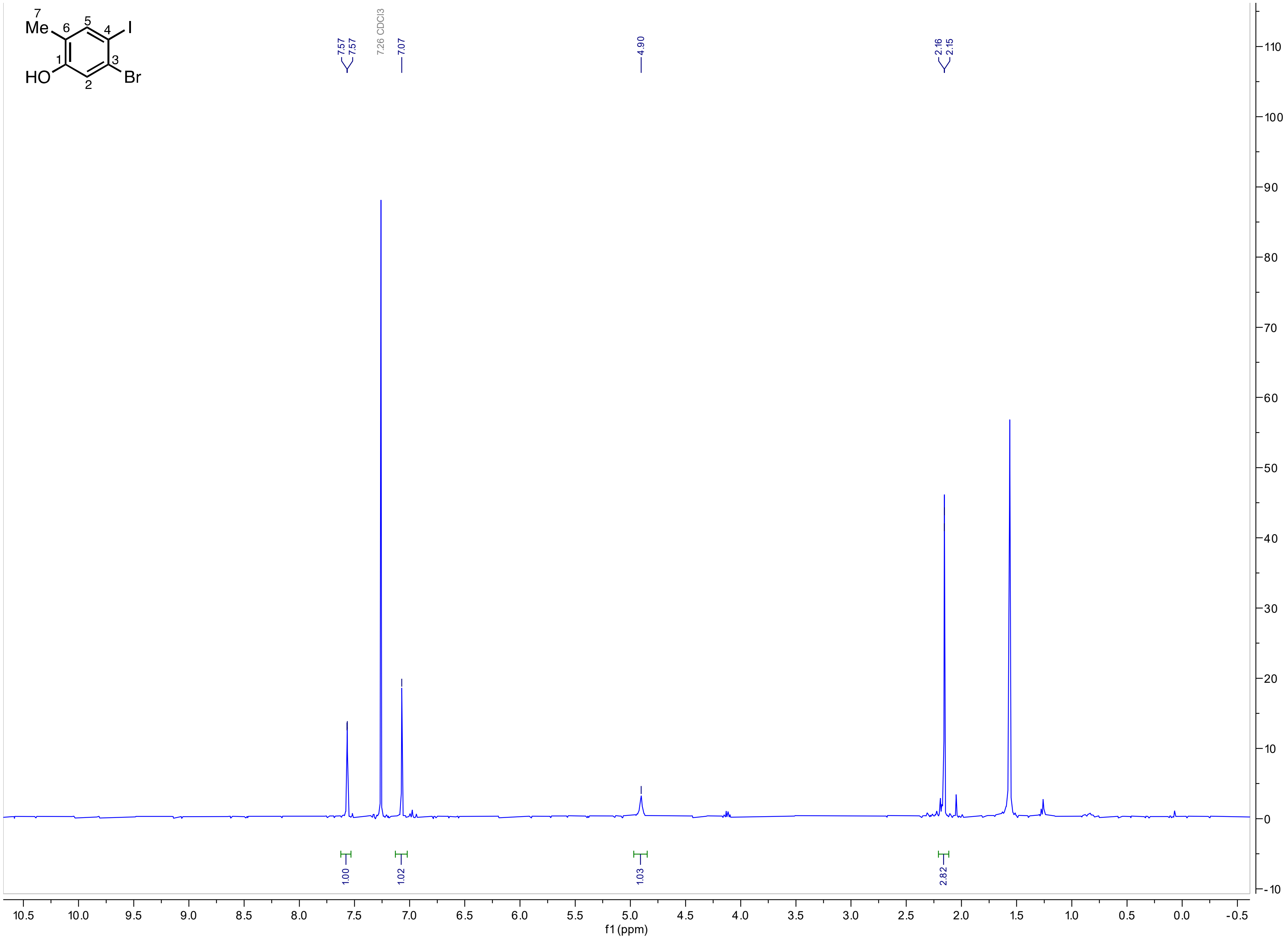

^{13}C NMR ($CDCl_3$): 5-Bromo-4-iodo-2-methylphenol (**8**)

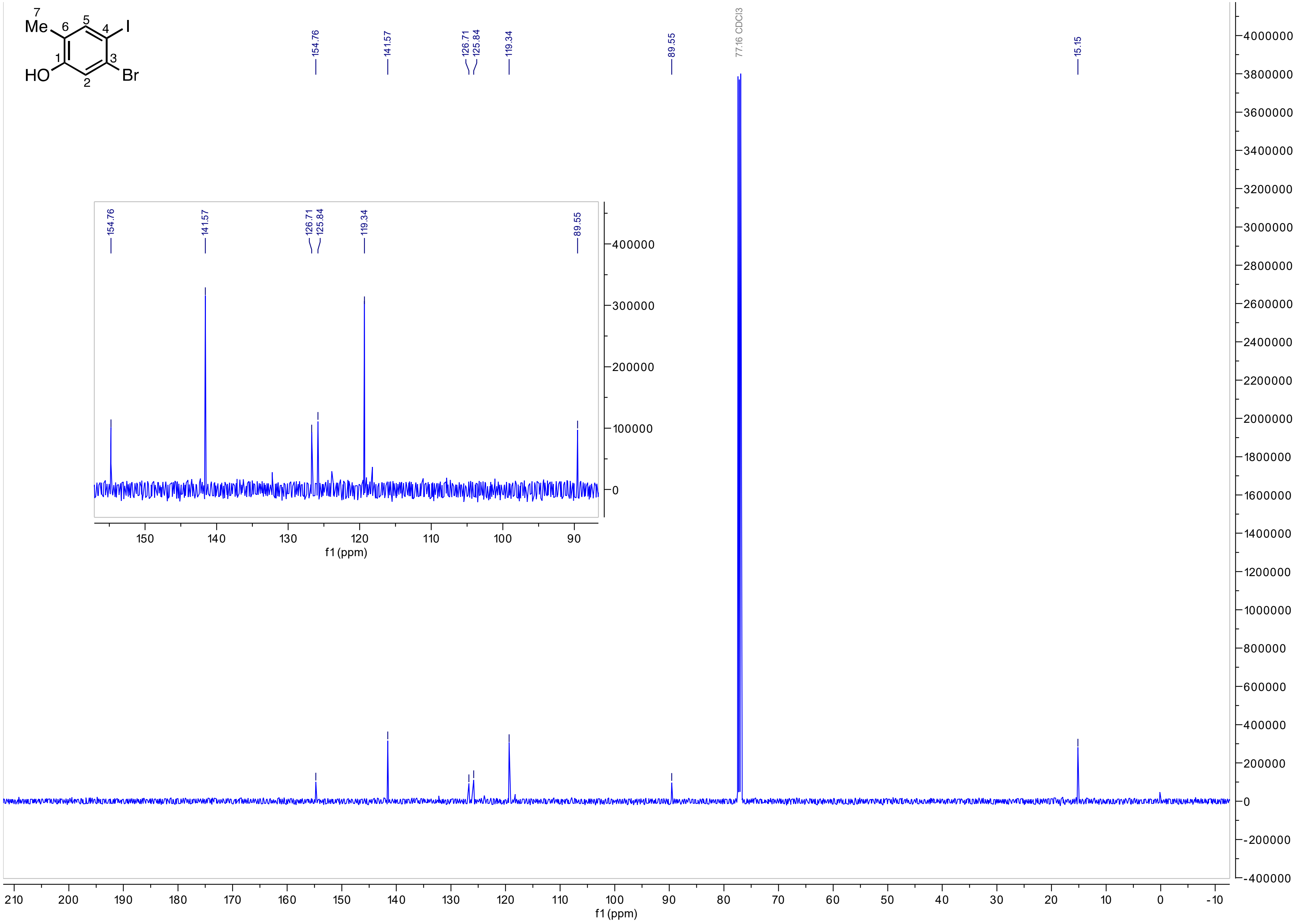

^{1}H NMR ($CDCl_3$): 1-Bromo-2-iodo-5-(methoxymethoxy)-4-methylbenzene (9)

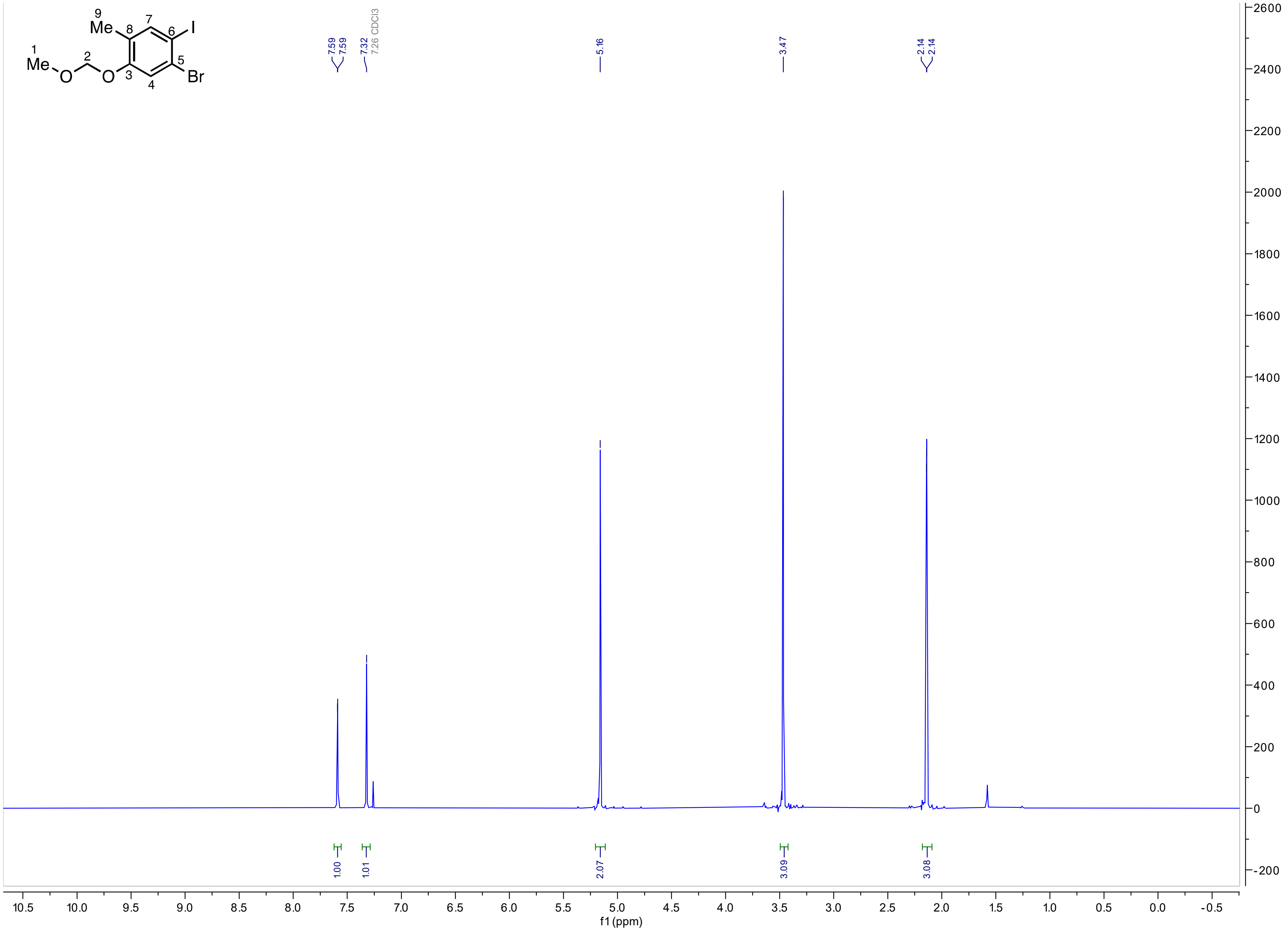

^{13}C NMR ($CDCl_3$): 1-Bromo-2-iodo-5-(methoxymethoxy)-4-methylbenzene (**9**)

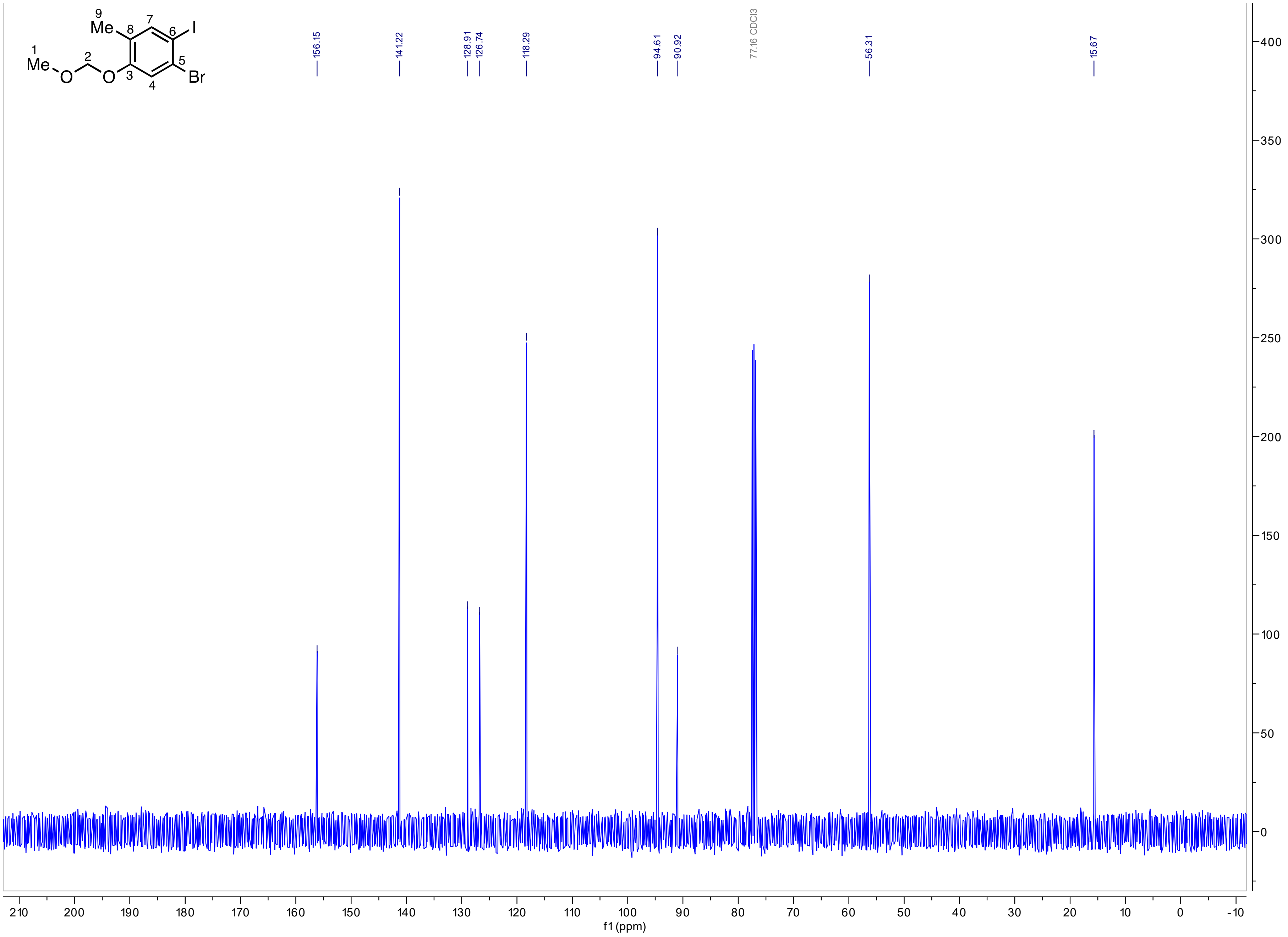

^{1}H NMR ($CDCl_3$): Band A

Major component: 7-(methoxymethoxy)-1,6-Dimethyl-1,4-dihydro-1,4-epoxynaphthalene (**S20**)

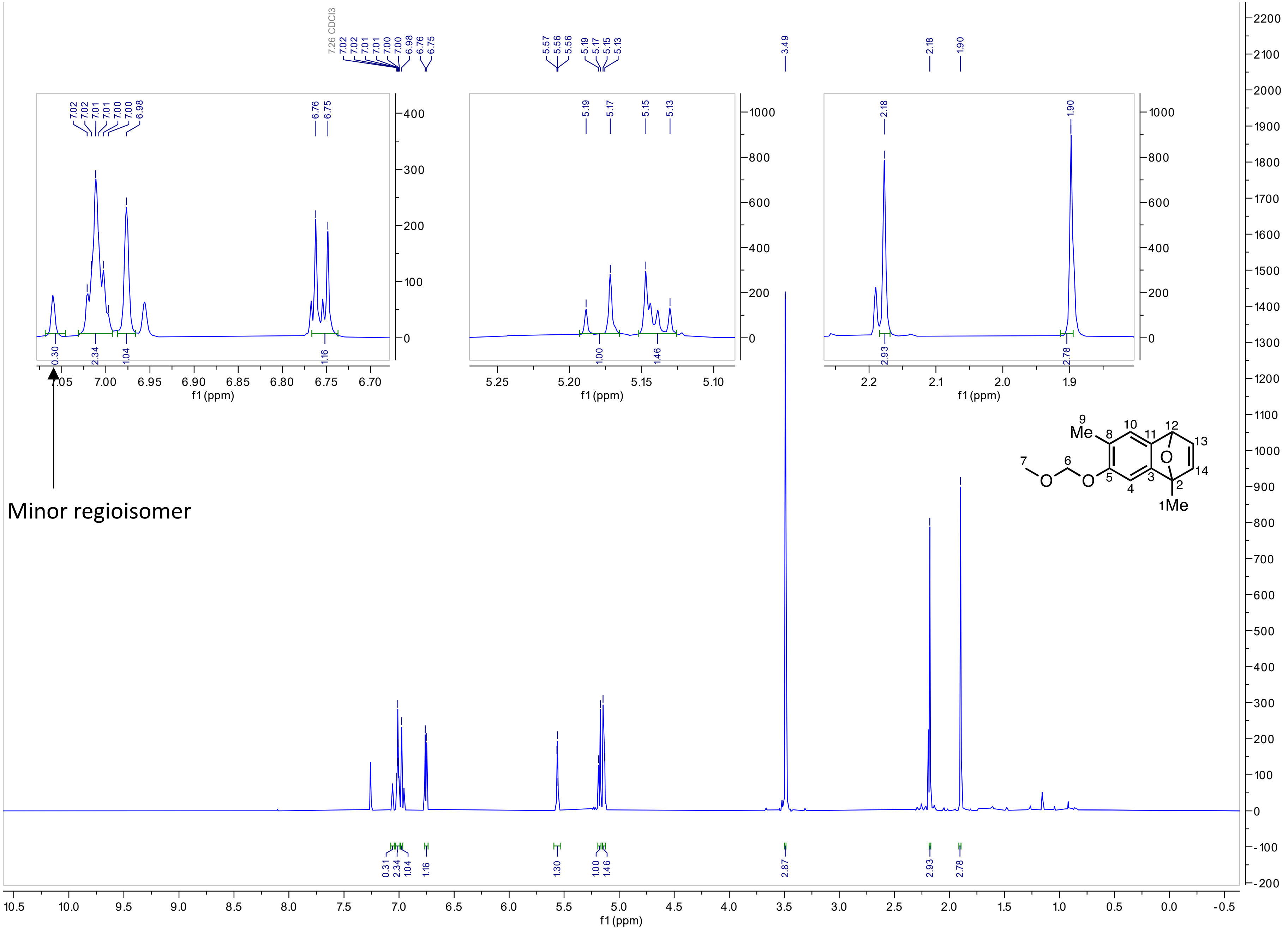

^{13}C NMR (CDCl$_3$): **Band A**

Major component: 7-(methoxymethoxy)-1,6-Dimethyl-1,4-dihydro-1,4-epoxynaphthalene (**S20**)

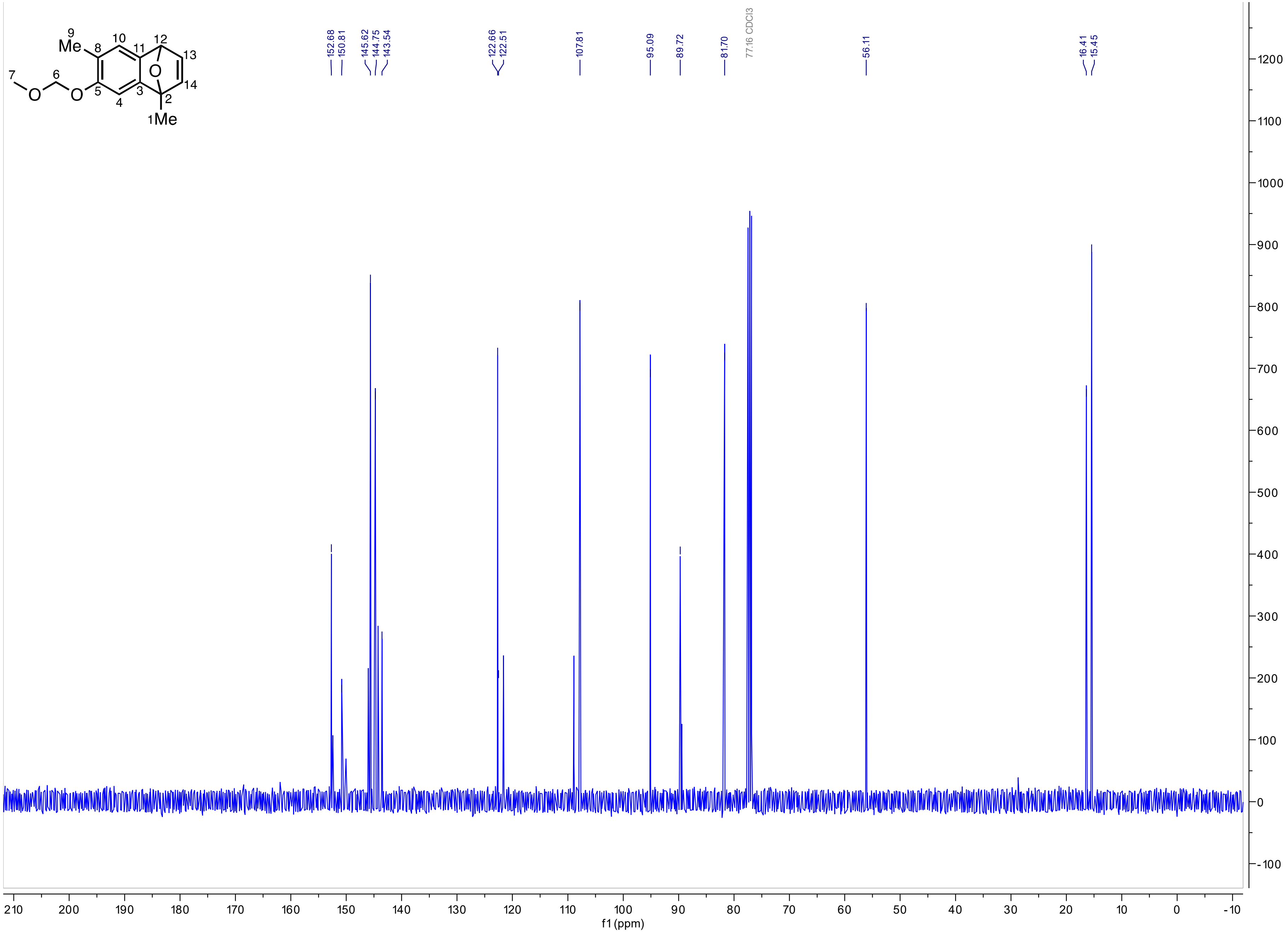

^{1}H NMR ($CDCl_3$): **Band C**

Major component: 6-(methoxymethoxy)-1,7-Dimethyl-1,4-dihydro-1,4-epoxynaphthalene (**S21**)

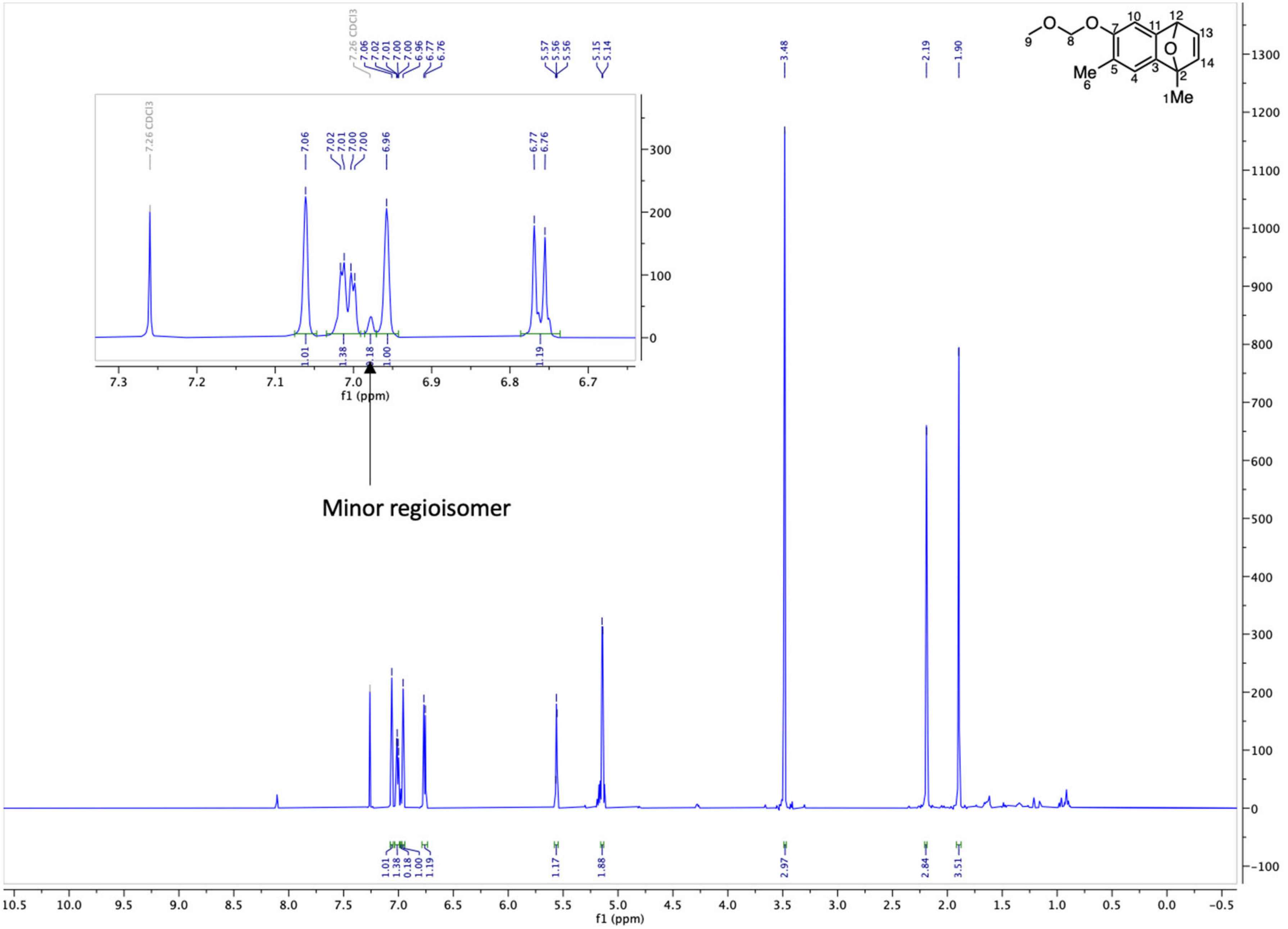

^{13}**C NMR** ($CDCl_3$): **Band C**

Major component: 6-(methoxymethoxy)-1,7-Dimethyl-1,4-dihydro-1,4-epoxynaphthalene (**S21**)

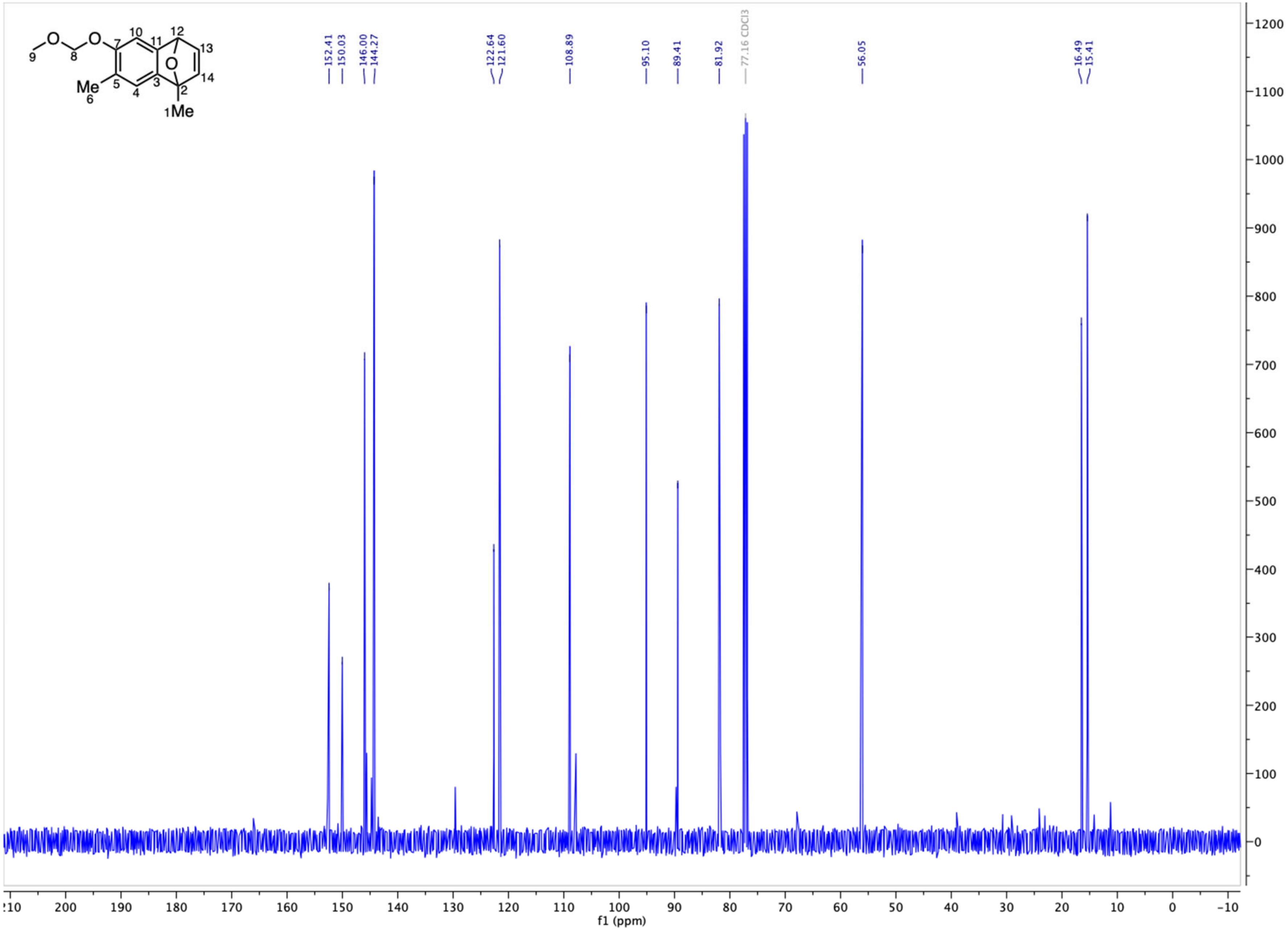

^{1}H NMR ($CDCl_3$): 6-(methoxymethoxy)-4,7-Dimethylnaphthalen-1-ol (**10**)

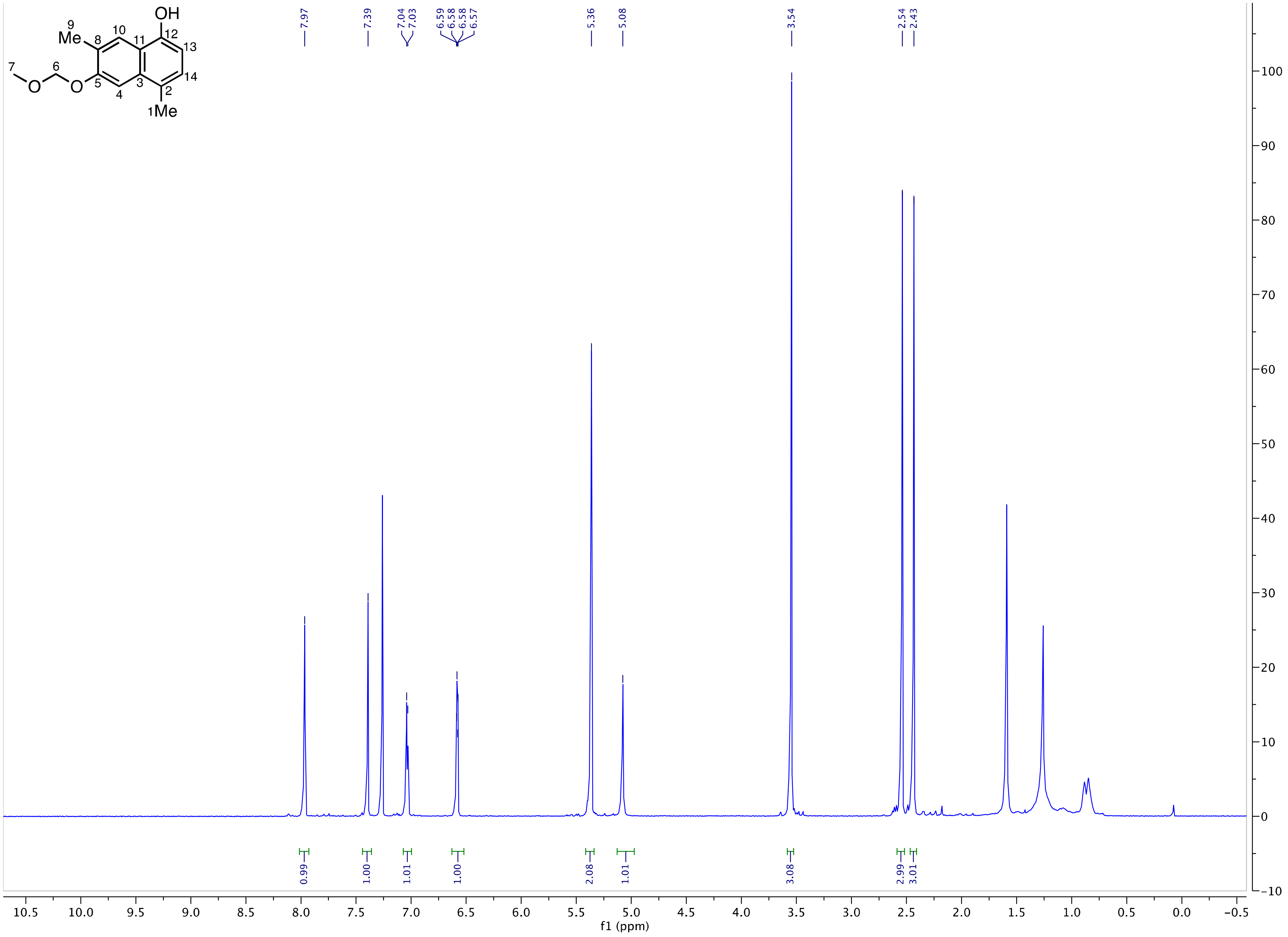

^{13}C NMR ($CDCl_3$): 6-(methoxymethoxy)-4,7-Dimethylnaphthalen-1-ol (10)

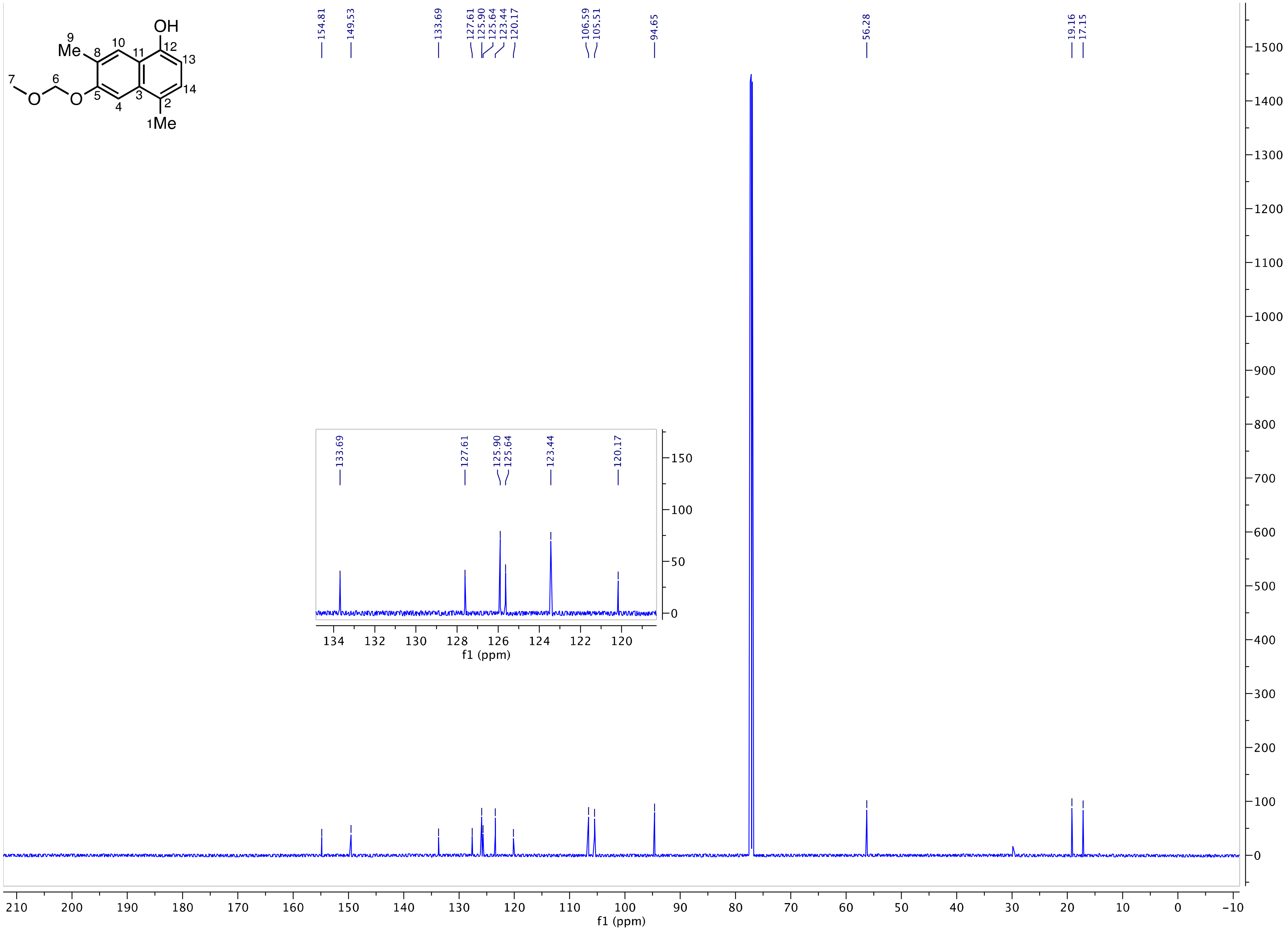

^{1}H NMR ($CDCl_3$): 7-(methoxymethoxy)-4,6-Dimethylnaphthalen-1-ol (**1h**)

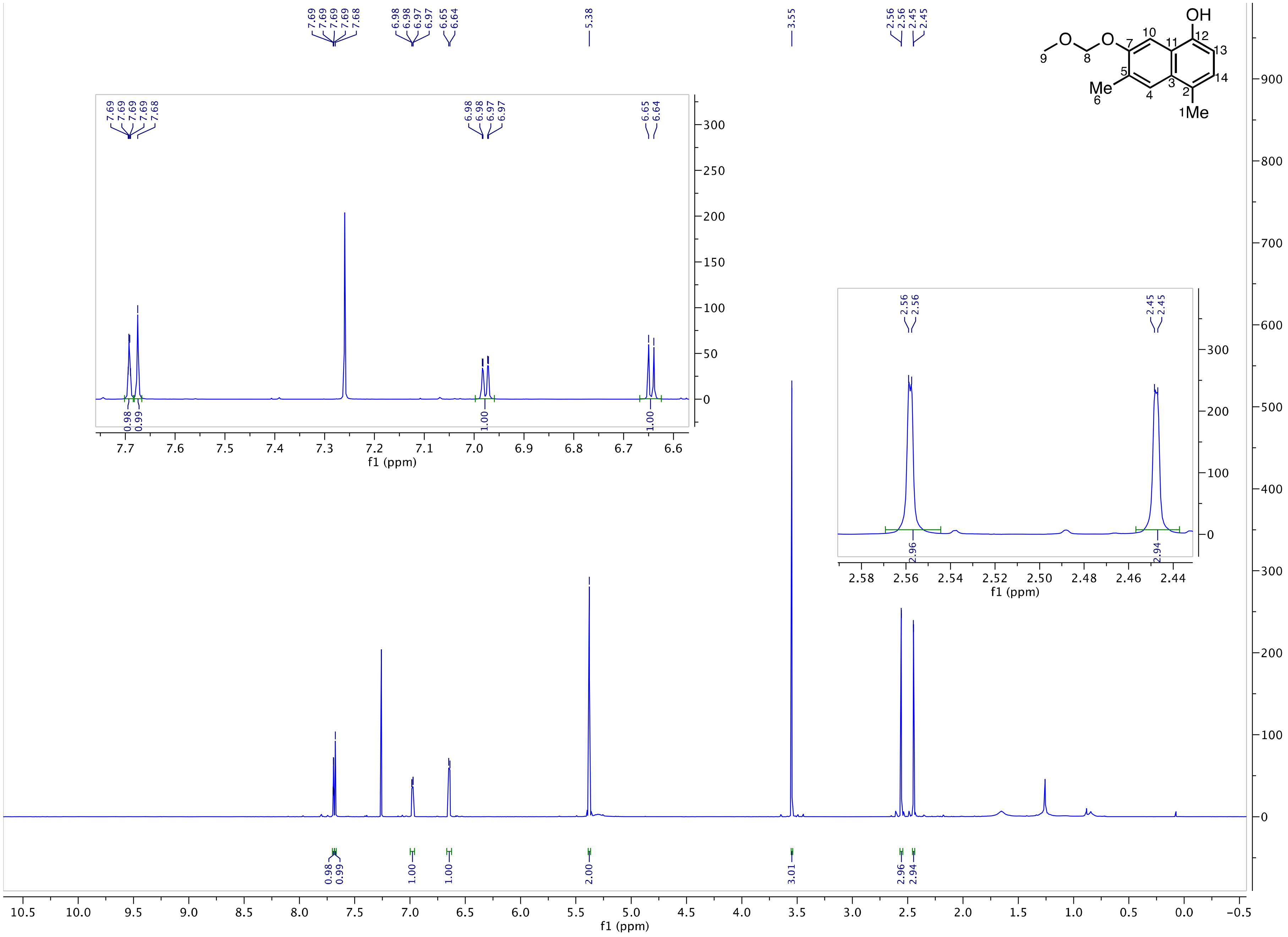

^{13}C NMR ($CDCl_3$): 7-(methoxymethoxy)-4,6-Dimethylnaphthalen-1-ol (1h)

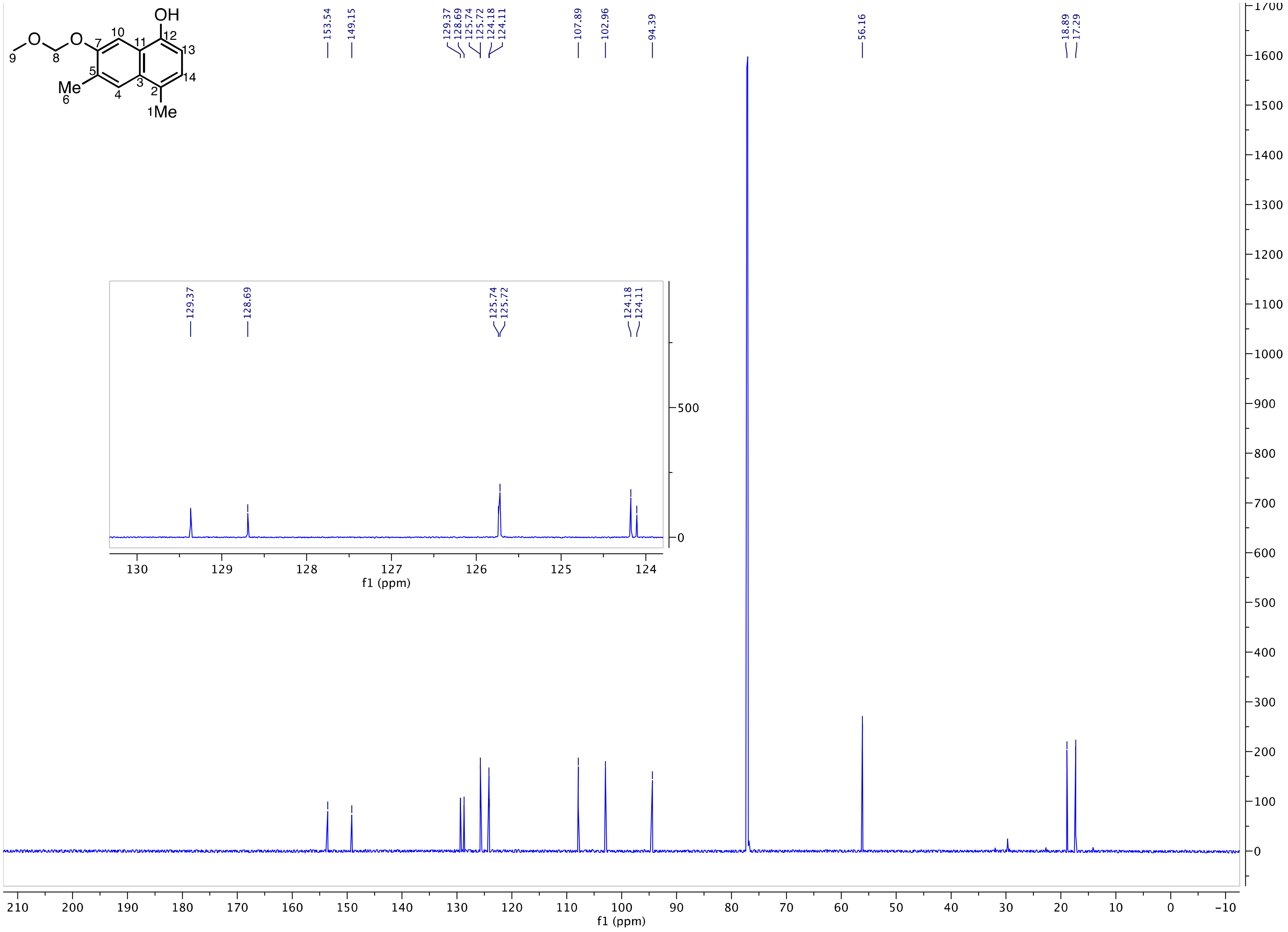

^{1}H NMR ($CDCl_3$): (*R*)-4-(3,4-bis(methoxymethoxy)phenyl)-6-(methoxymethoxy)-4,7-Dimethylnaphthalen-1(4*H*)-one (**11**)

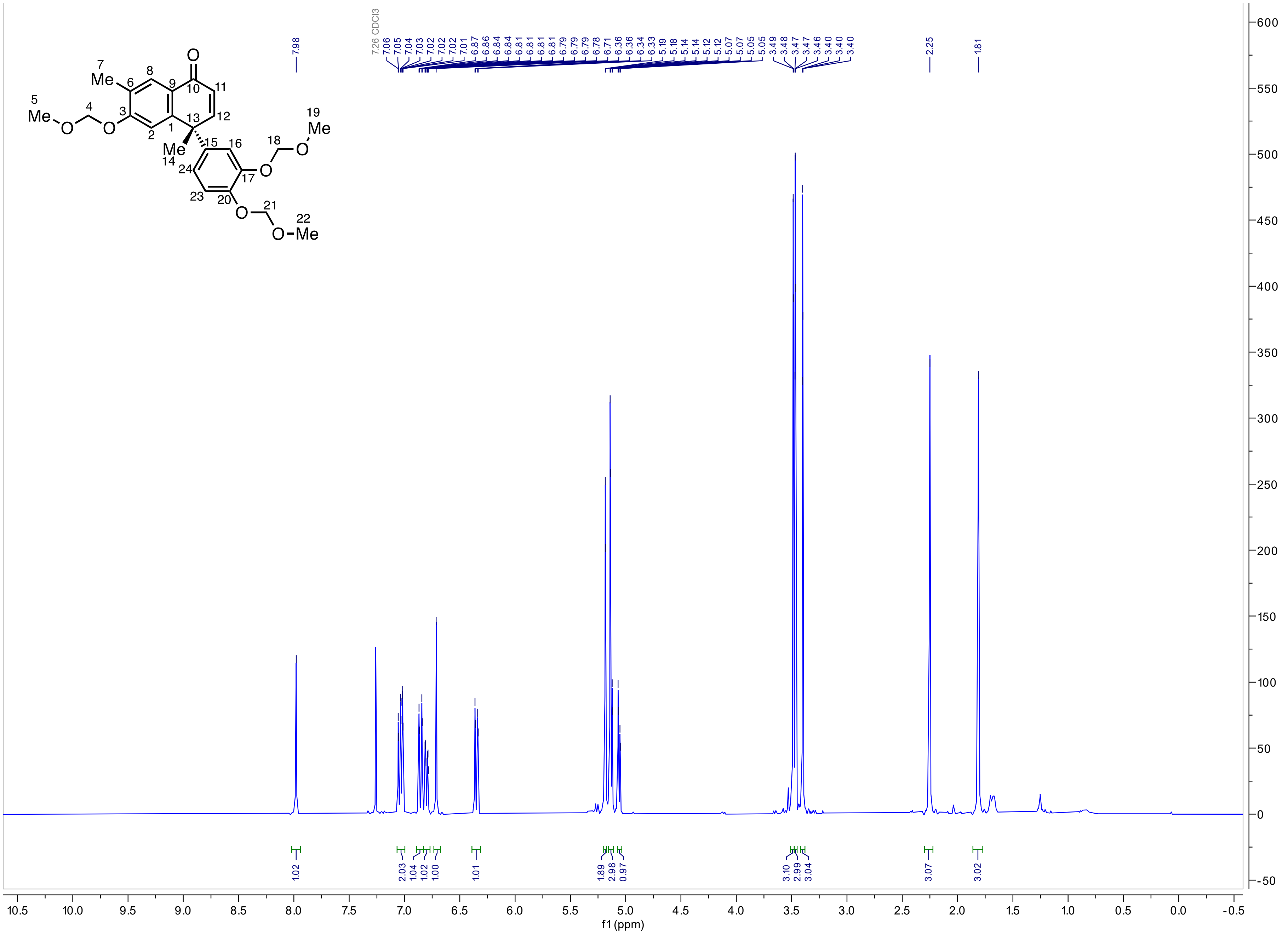

^{13}C NMR ($CDCl_3$): (*R*)-4-(3,4-bis(methoxymethoxy)phenyl)-6-(methoxymethoxy)-4,7-Dimethylnaphthalen-1(4*H*)-one (**11**)

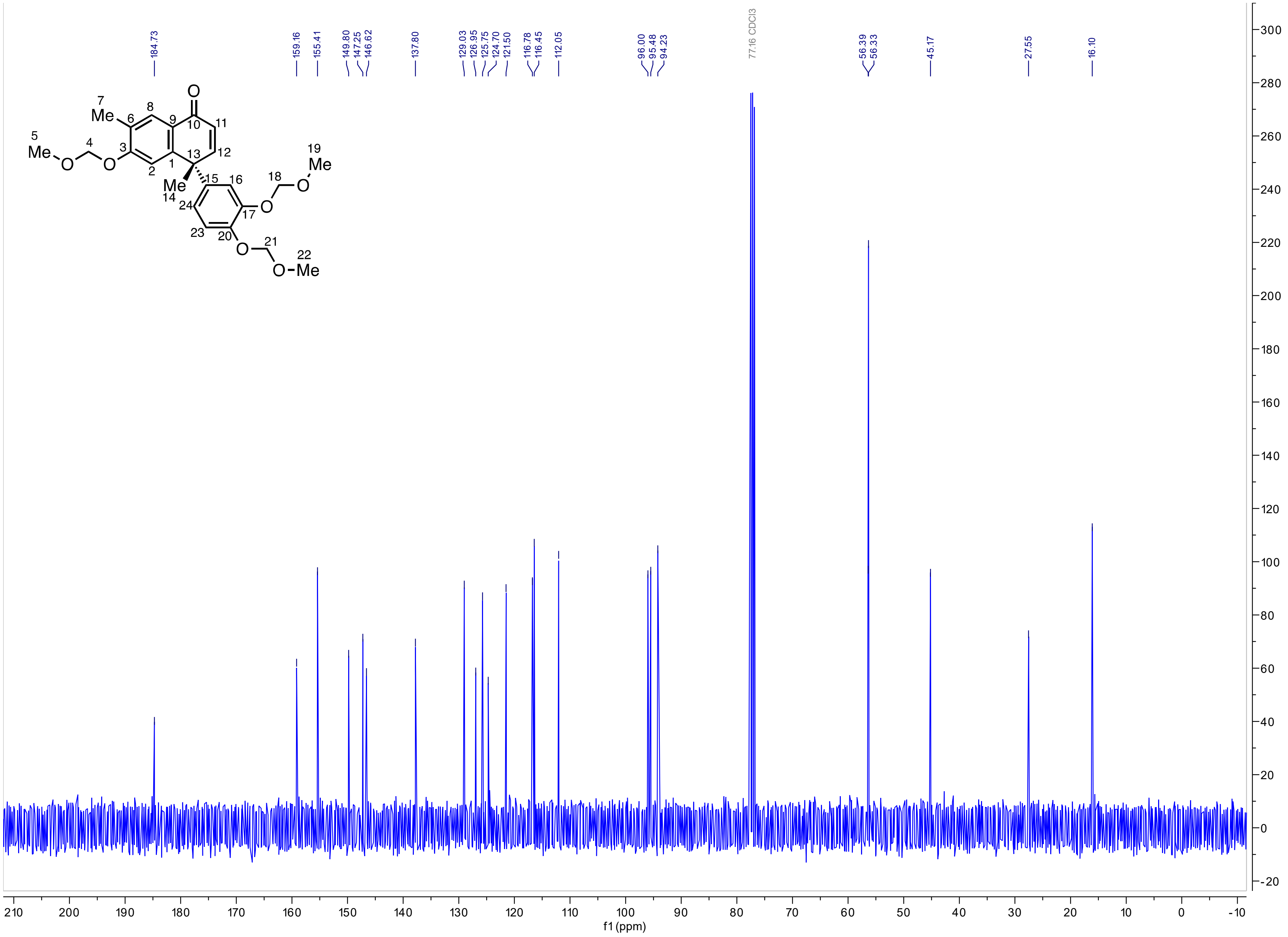

^{1}H NMR ($CDCl_3$): (*R*)-4-(3,4-bis(methoxymethoxy)phenyl)-6-(methoxymethoxy)-4,7-Dimethyl-3,4-dihydronaphthalen-1(2*H*)-one (**7**)

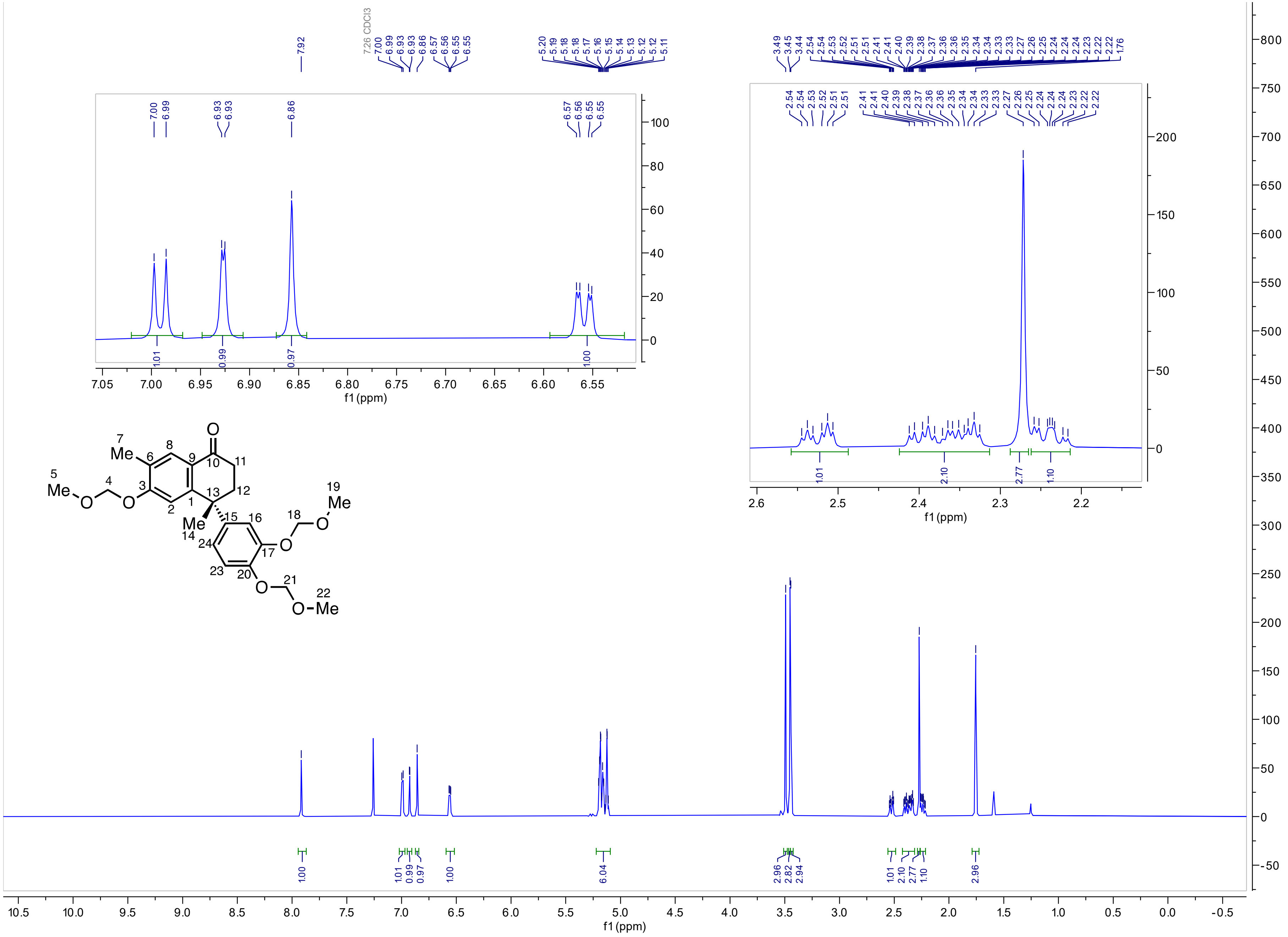

^{13}C NMR ($CDCl_3$): (*R*)-4-(3,4-bis(methoxymethoxy)phenyl)-6-(methoxymethoxy)-4,7-Dimethyl-3,4-dihydronaphthalen-1(2*H*)-one (**7**)

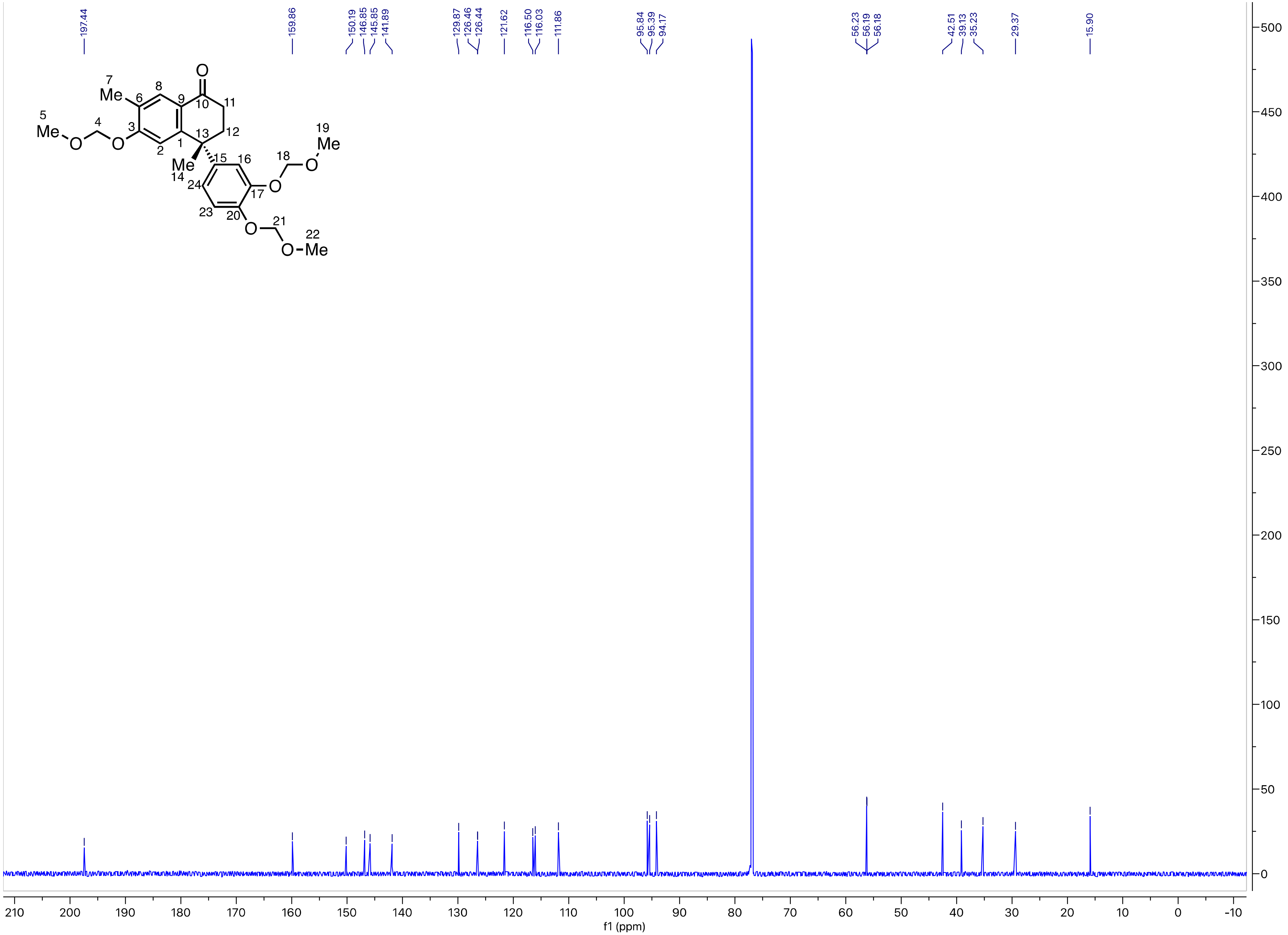

^{1}H NMR ($CDCl_3$): 4-Phenylnaphthalen-1-ol (12a)

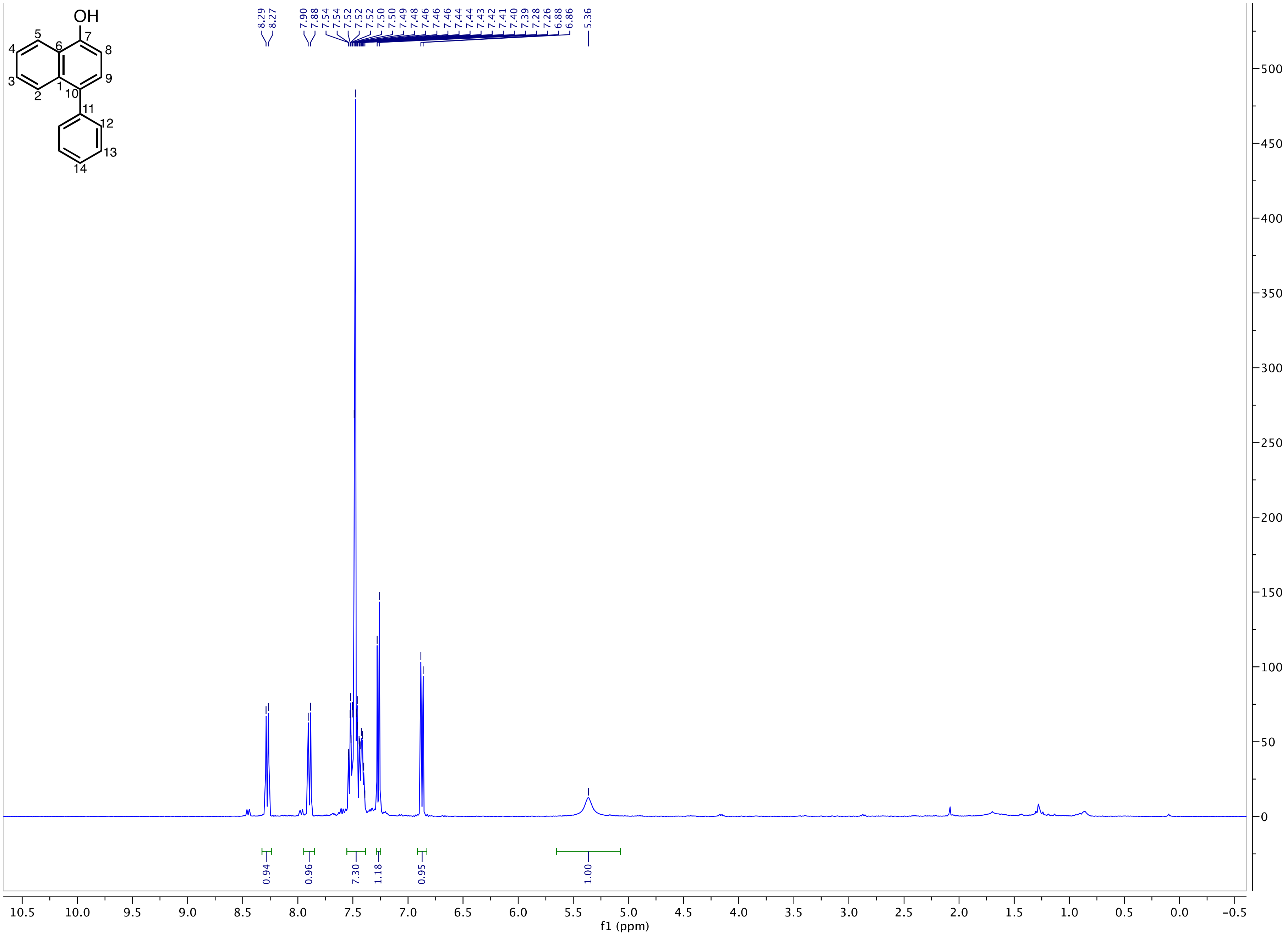

^{13}C NMR ($CDCl_3$): 4-Phenylnaphthalen-1-ol (**12a**)

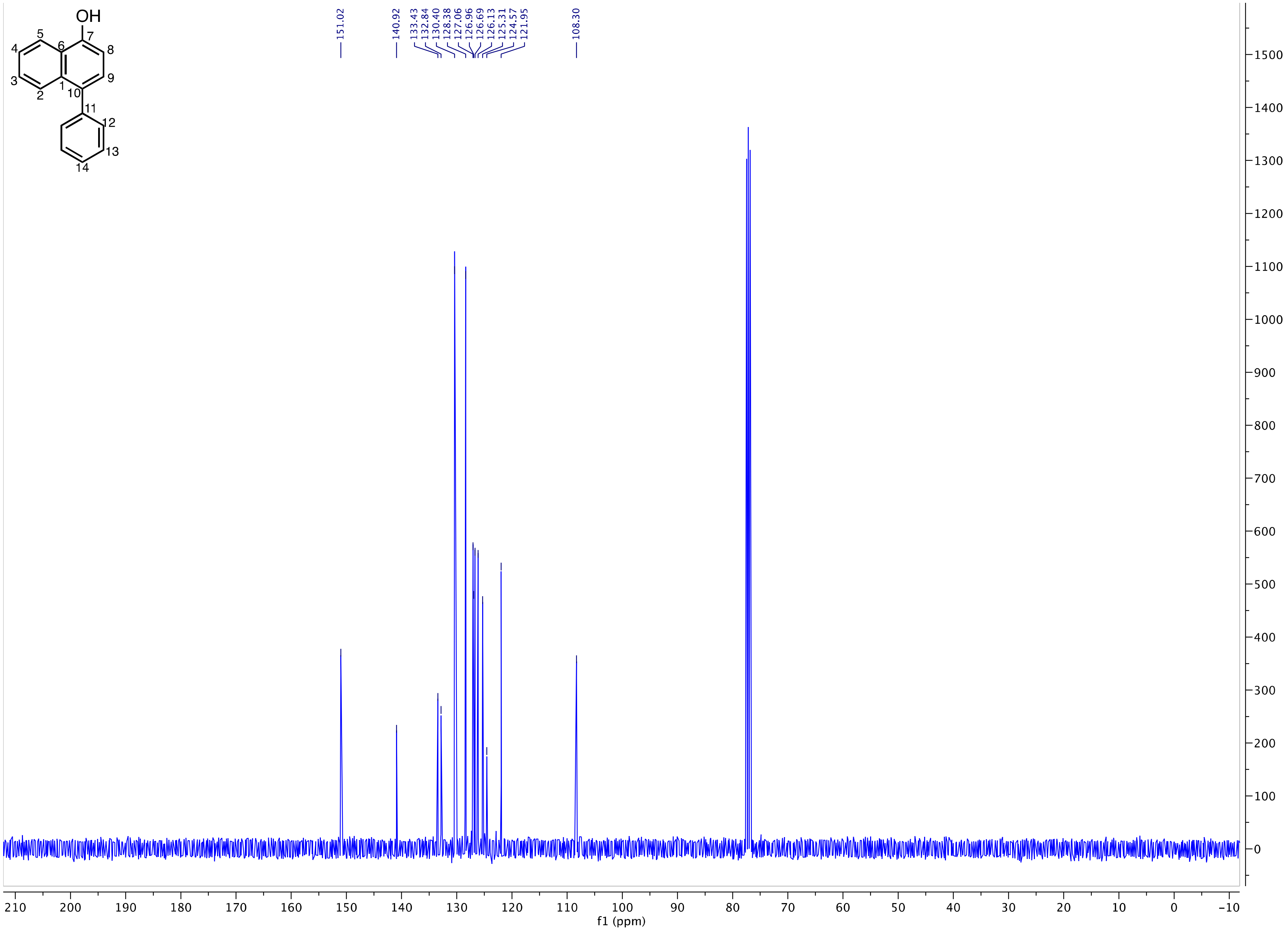

^{1}H NMR ($CDCl_3$): 4-(4-methoxyphenyl)Naphthalen-1-ol (**12b**)

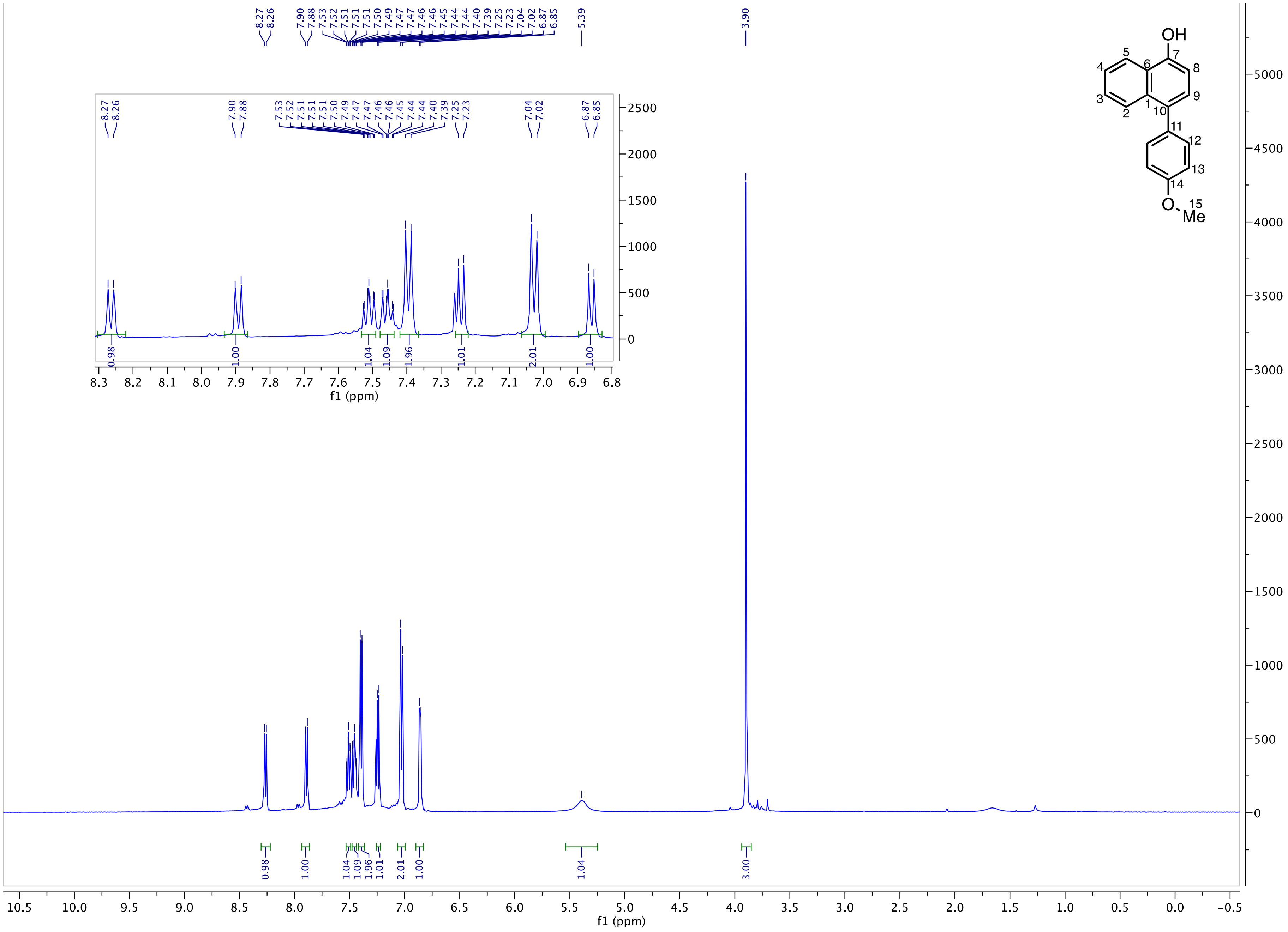

^{13}C NMR ($CDCl_3$): 4-(4-methoxyphenyl)Naphthalen-1-ol (**12b**)

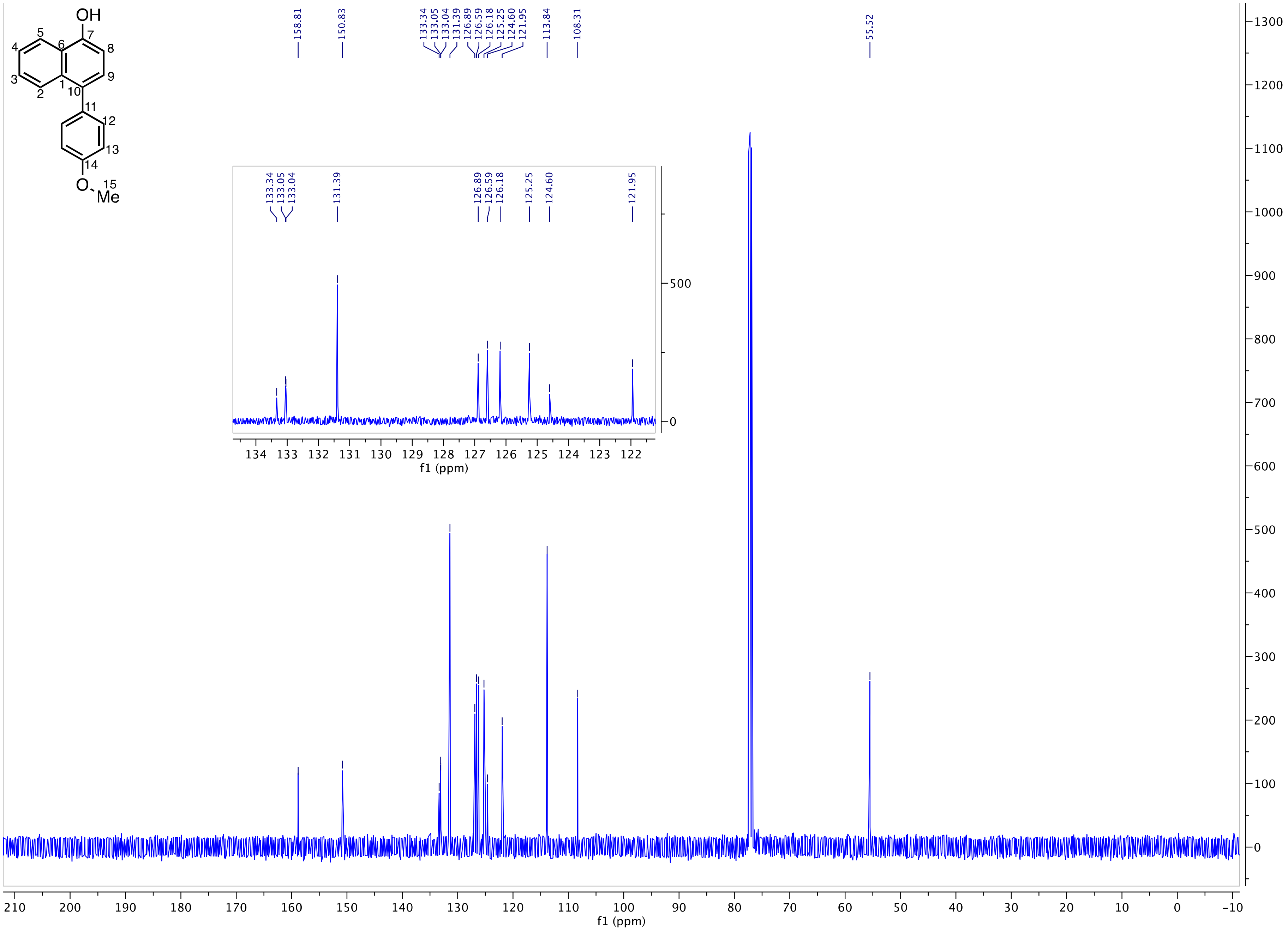

^{1}H NMR ($CDCl_3$): 4-(6-methoxypyridin-3-yl)Naphthalen-1-ol (**12c**)

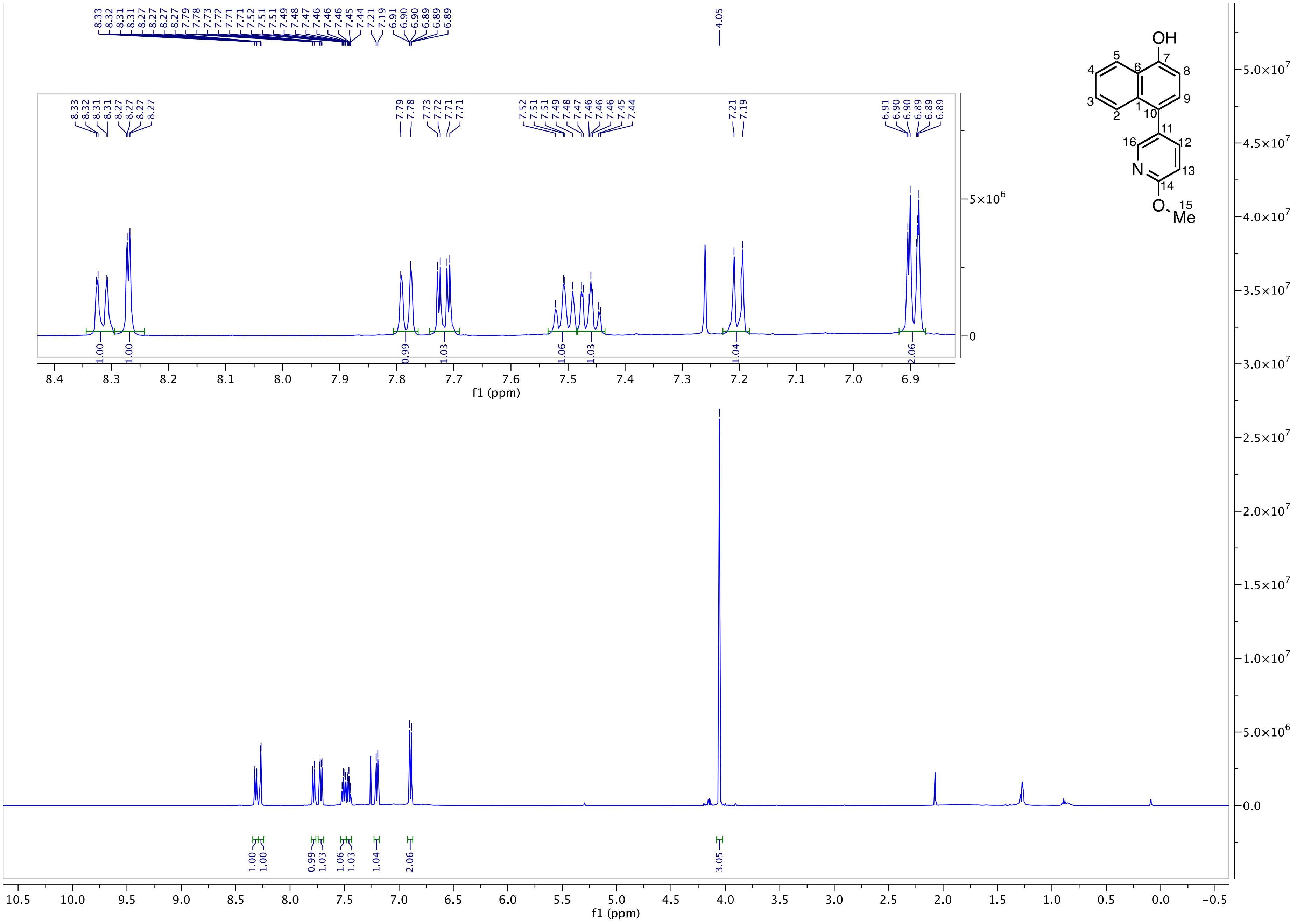

^{13}C NMR ($CDCl_3$): 4-(6-methoxypyridin-3-yl)Naphthalen-1-ol (**12c**)

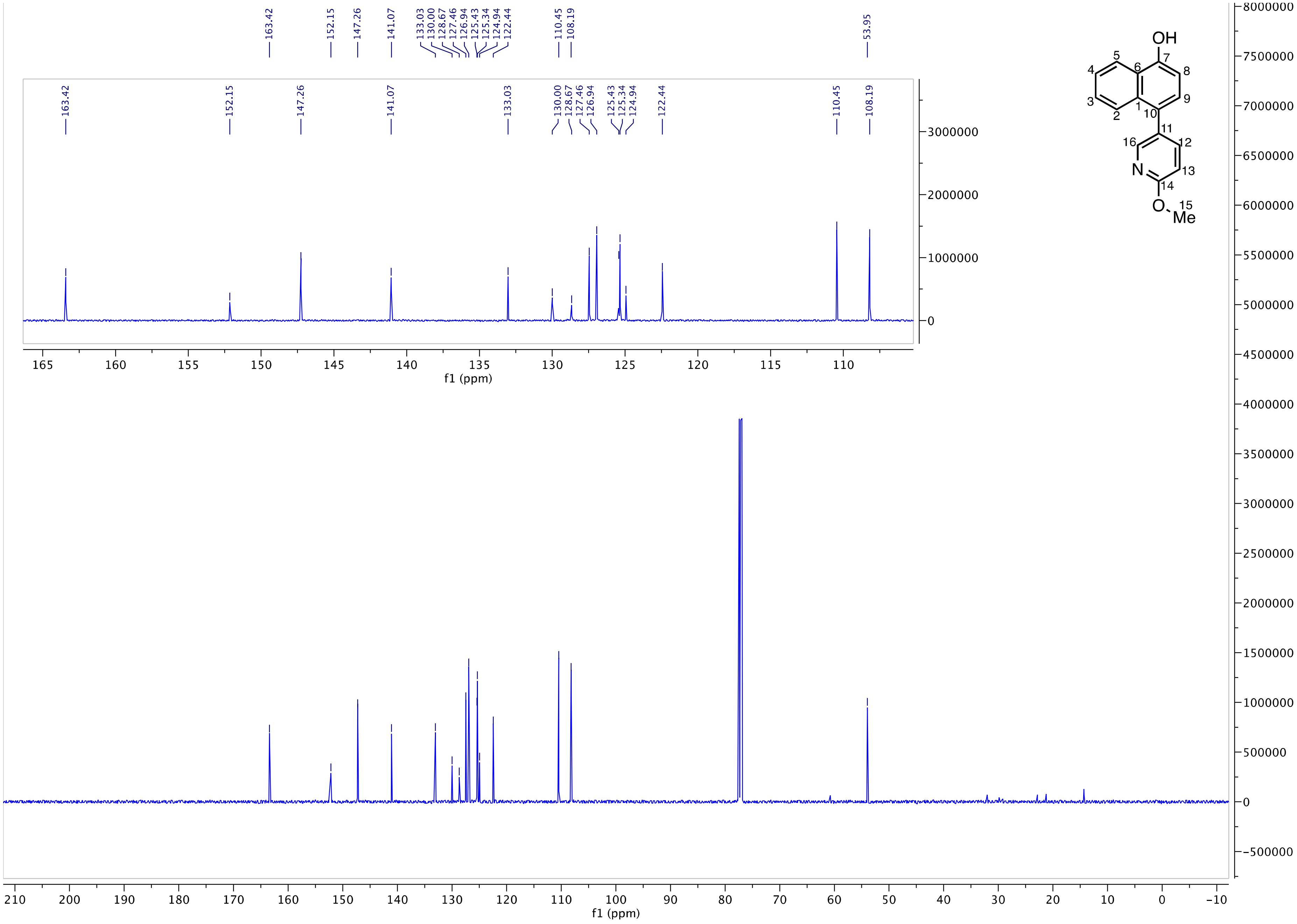

^{1}H NMR ($CDCl_3$): 4-(1*H*-indol-5-yl)Naphthalen-1-ol (**12d**)

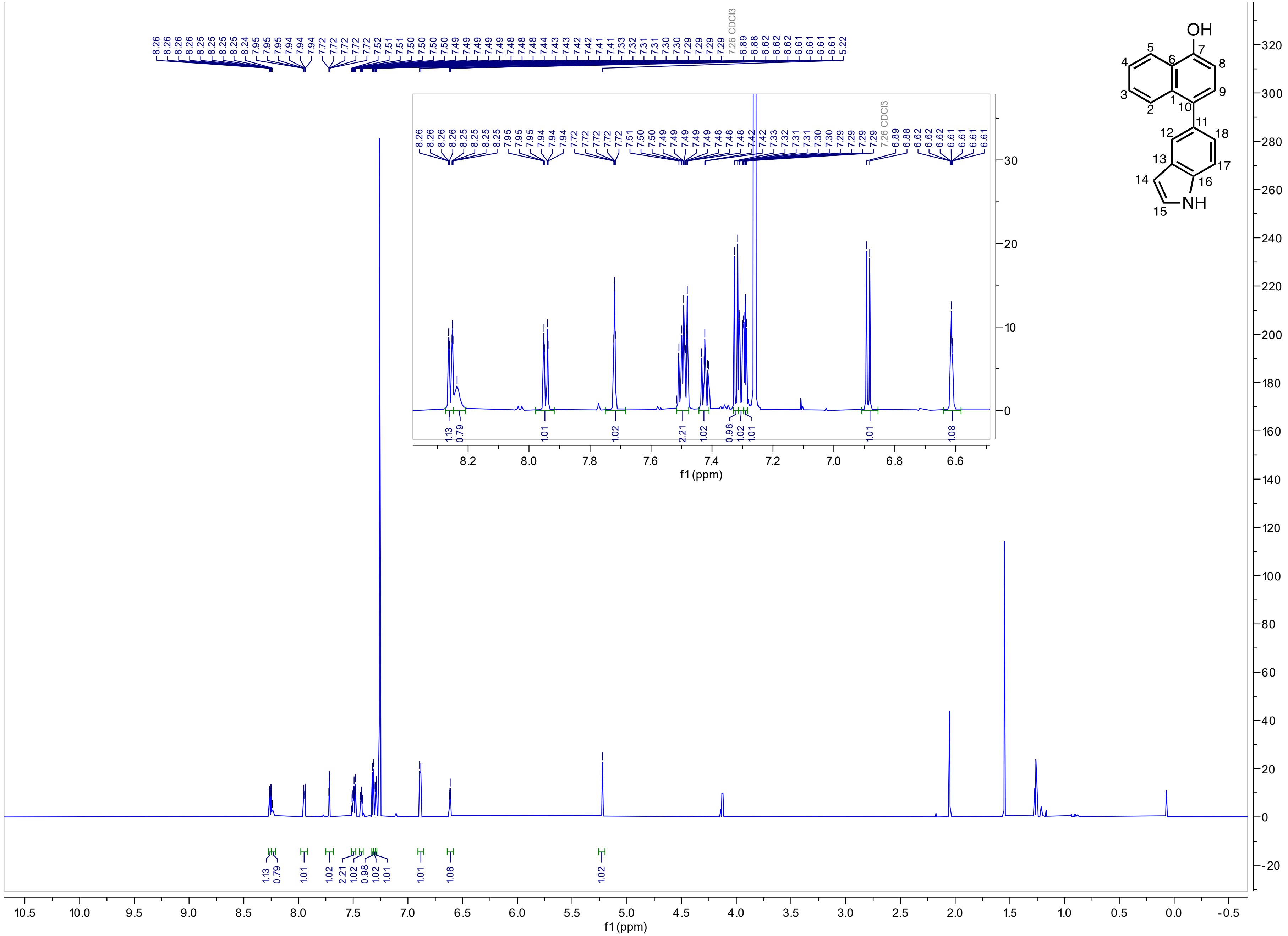

^{13}C NMR (CDCl$_3$): 4-(1*H*-indol-5-yl)Naphthalen-1-ol (**12d**)

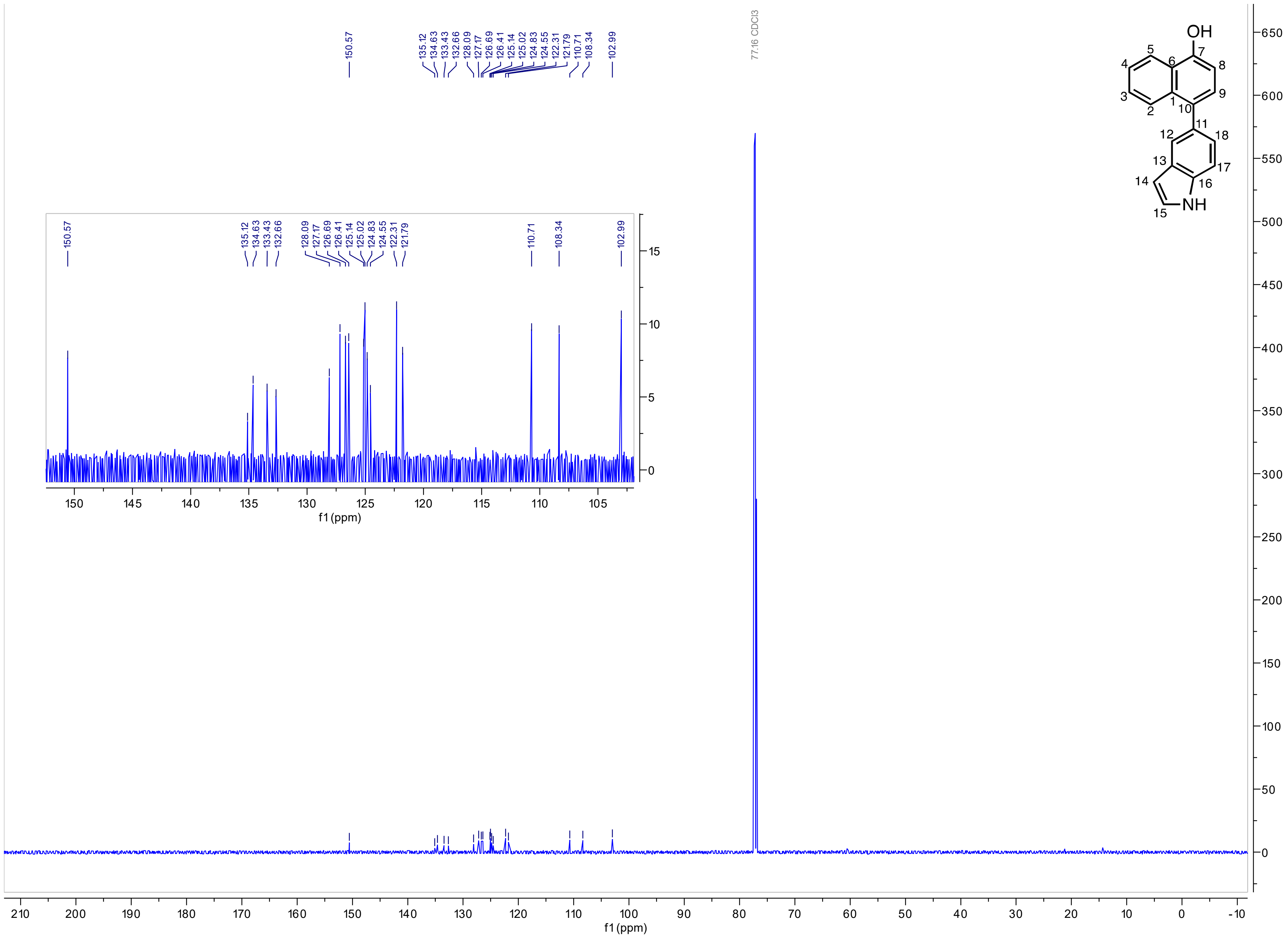

^{1}H NMR ($CDCl_3$): 4-(9,9-dibutyl-9H-fluoren-2-yl)Naphthalen-1-ol (**12e**)

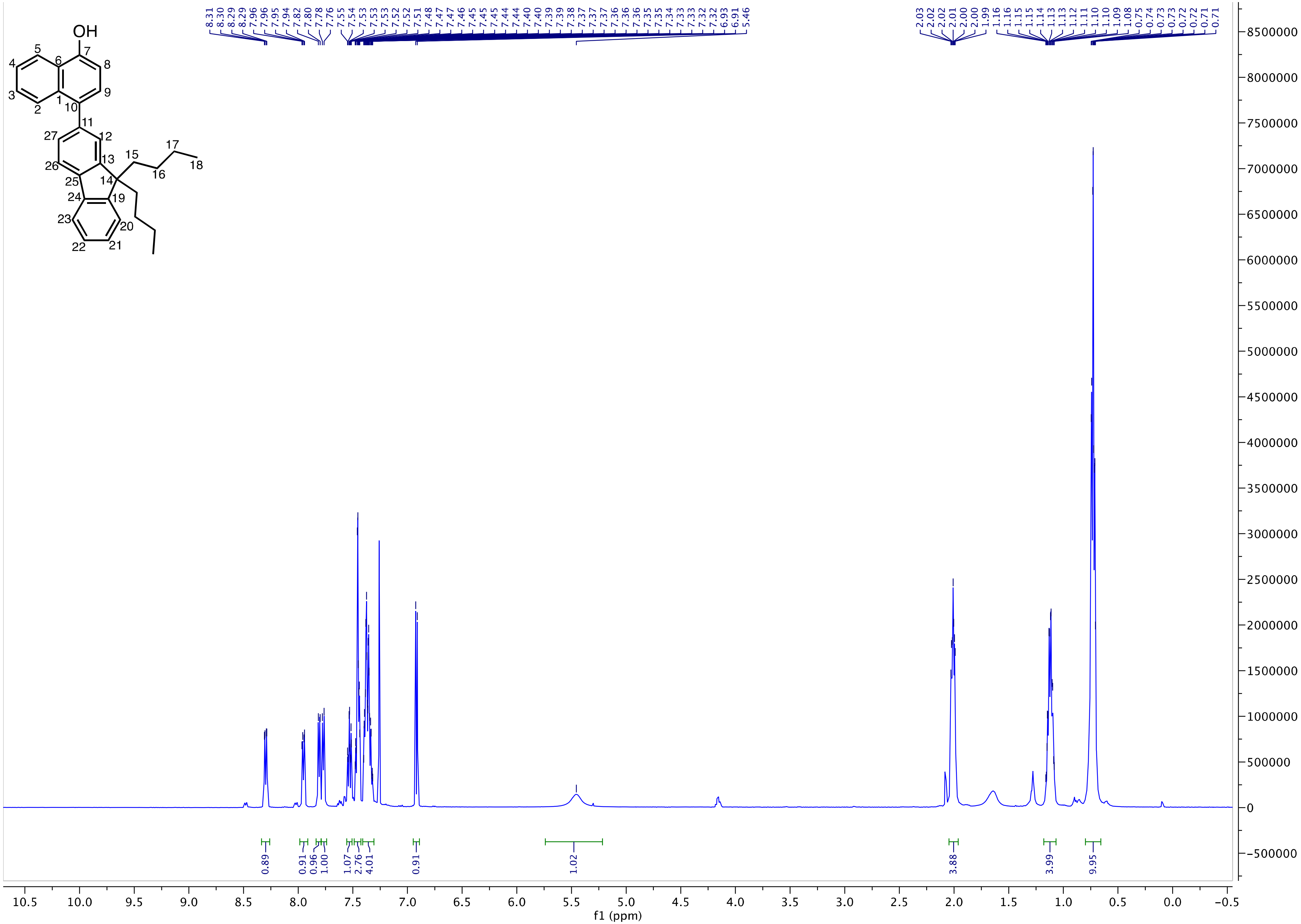

^{13}C NMR ($CDCl_3$): 4-(9,9-dibutyl-9H-fluoren-2-yl)Naphthalen-1-ol (**12e**)

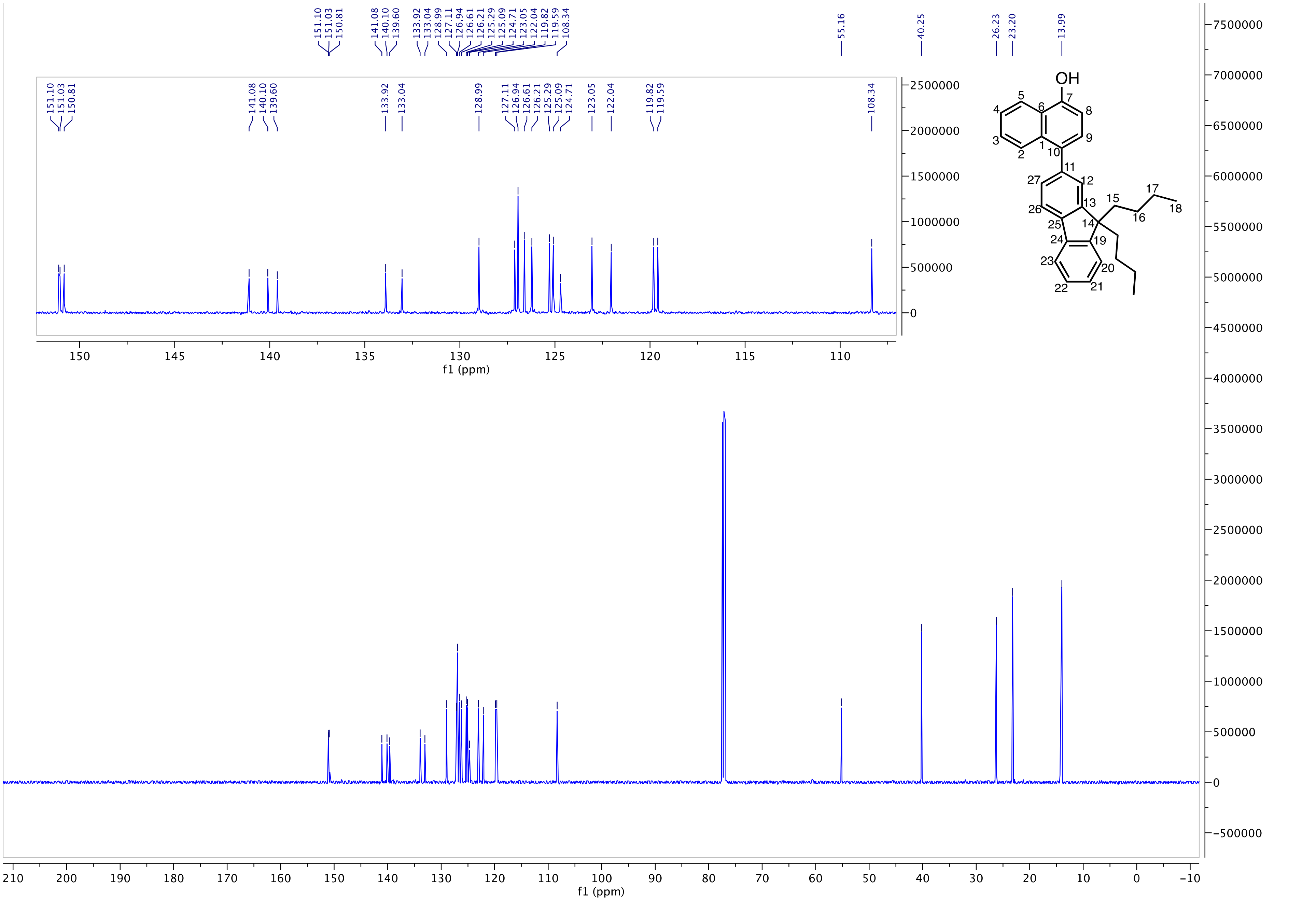

^{1}H NMR ($CDCl_3$): 1-(2-bromophenethyl)Naphthalen-2-ol (**S24**)

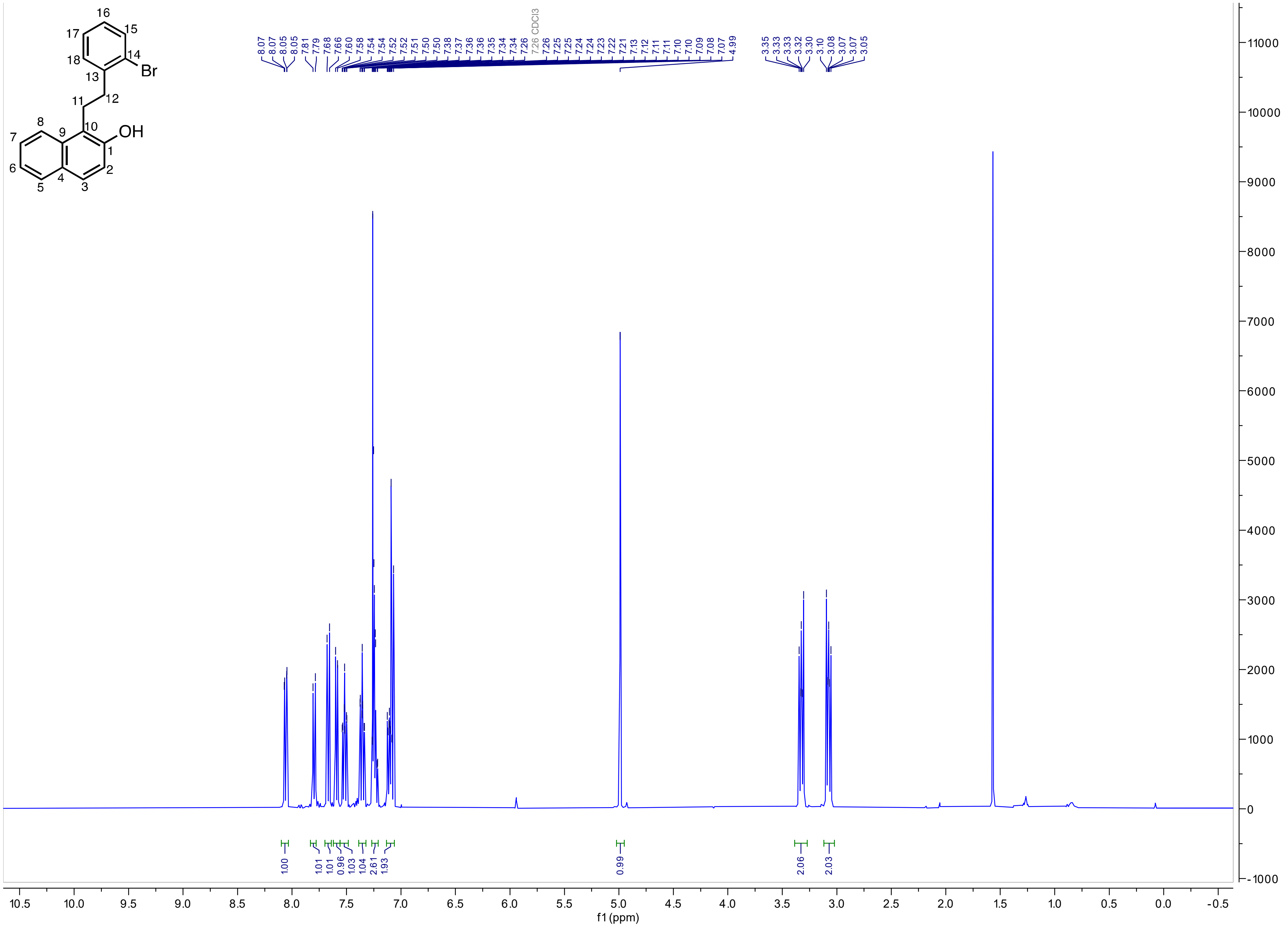

^{13}C NMR ($CDCl_3$): 1-(2-bromophenethyl)Naphthalen-2-ol (S24)

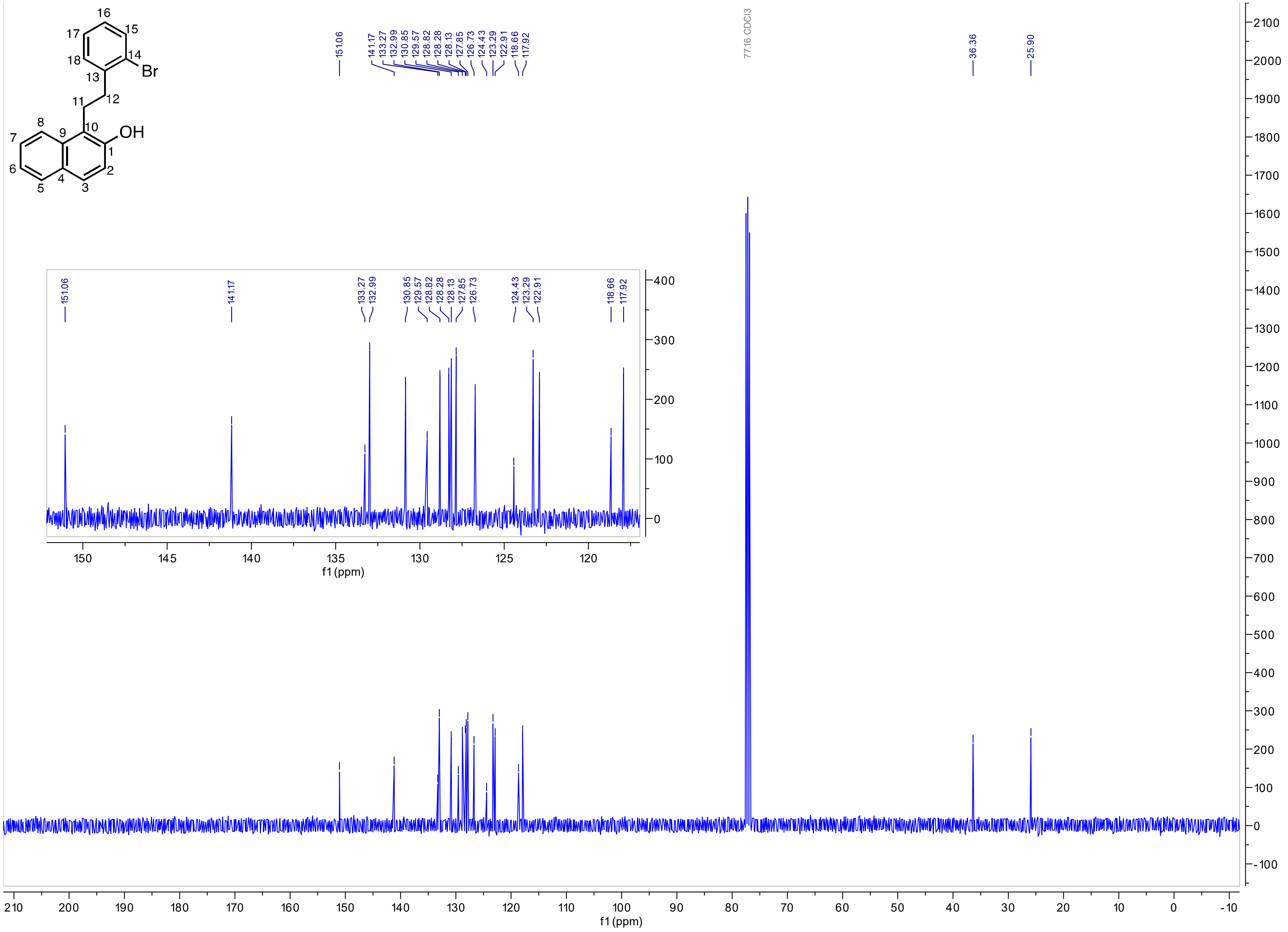

^{1}H NMR ($CDCl_3$): 2,3-Dihydro-2'*H*-spiro[indene-1,1'-naphthalen]-2'-one (**S25**)

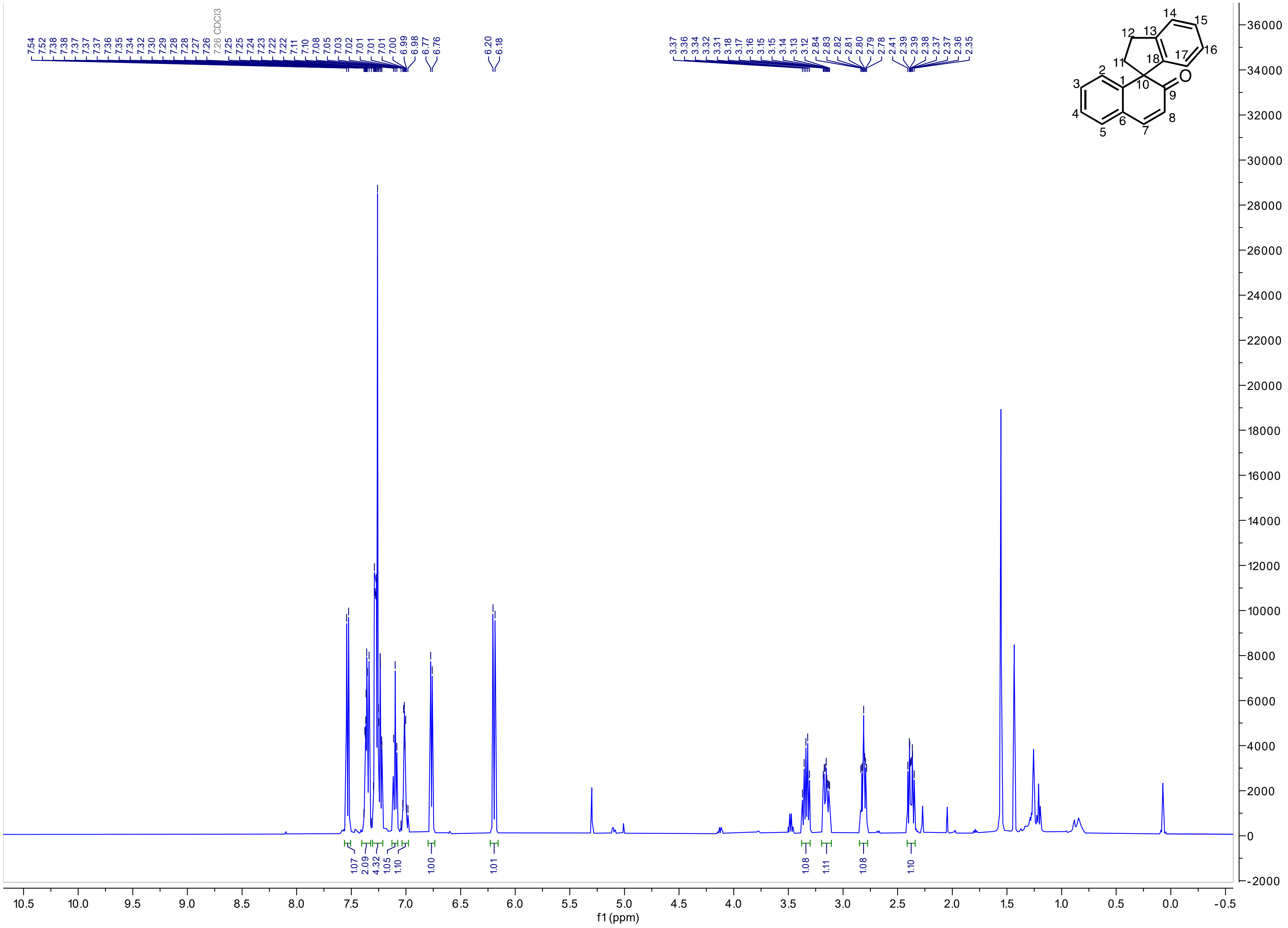

^{13}C NMR ($CDCl_3$): 2,3-Dihydro-2'*H*-spiro[indene-1,1'-naphthalen]-2'-one (**S25**)

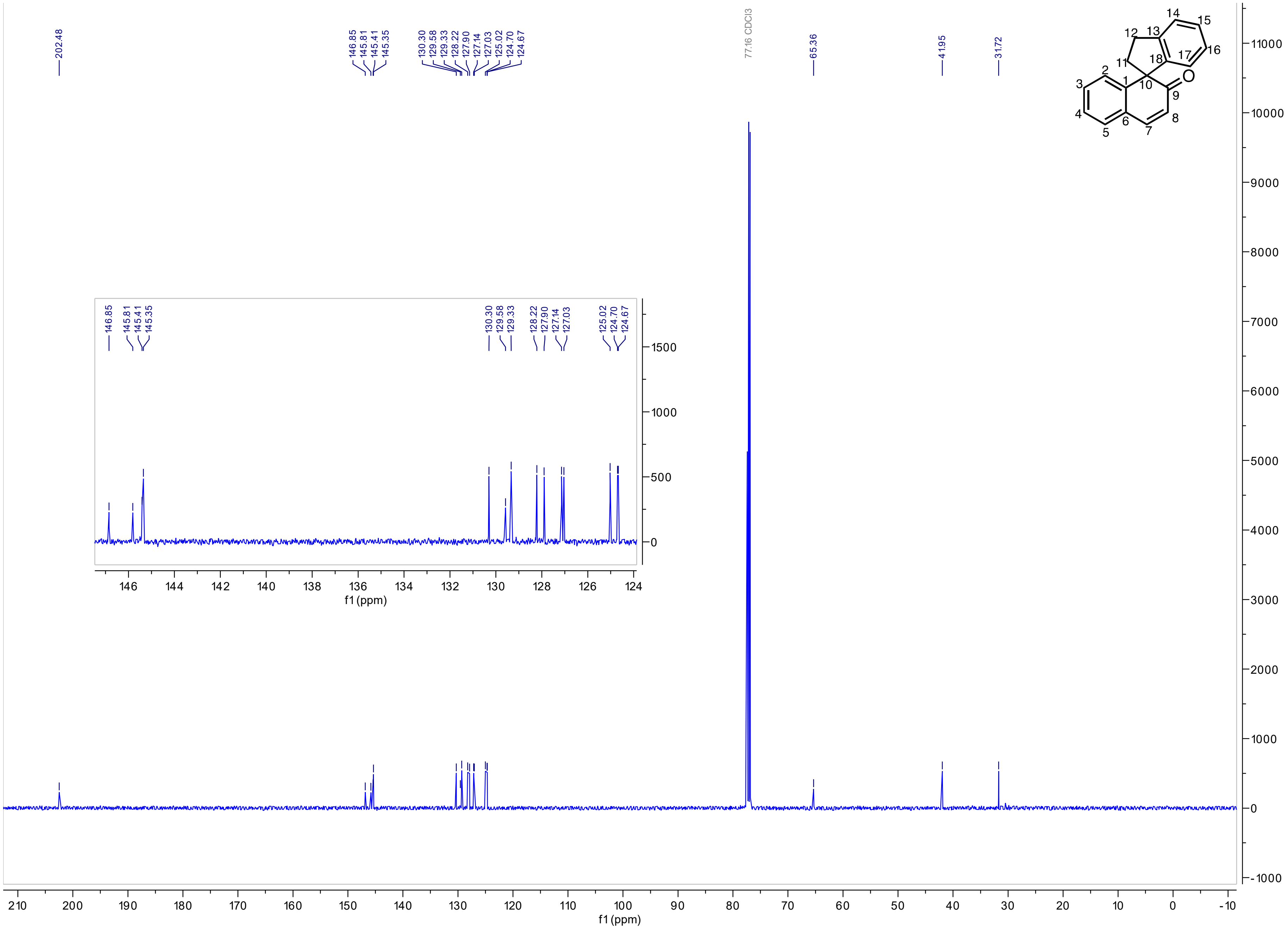

^{1}H NMR ($CDCl_3$): 1,3-Dimethylnaphthalen-2-ol (S26)

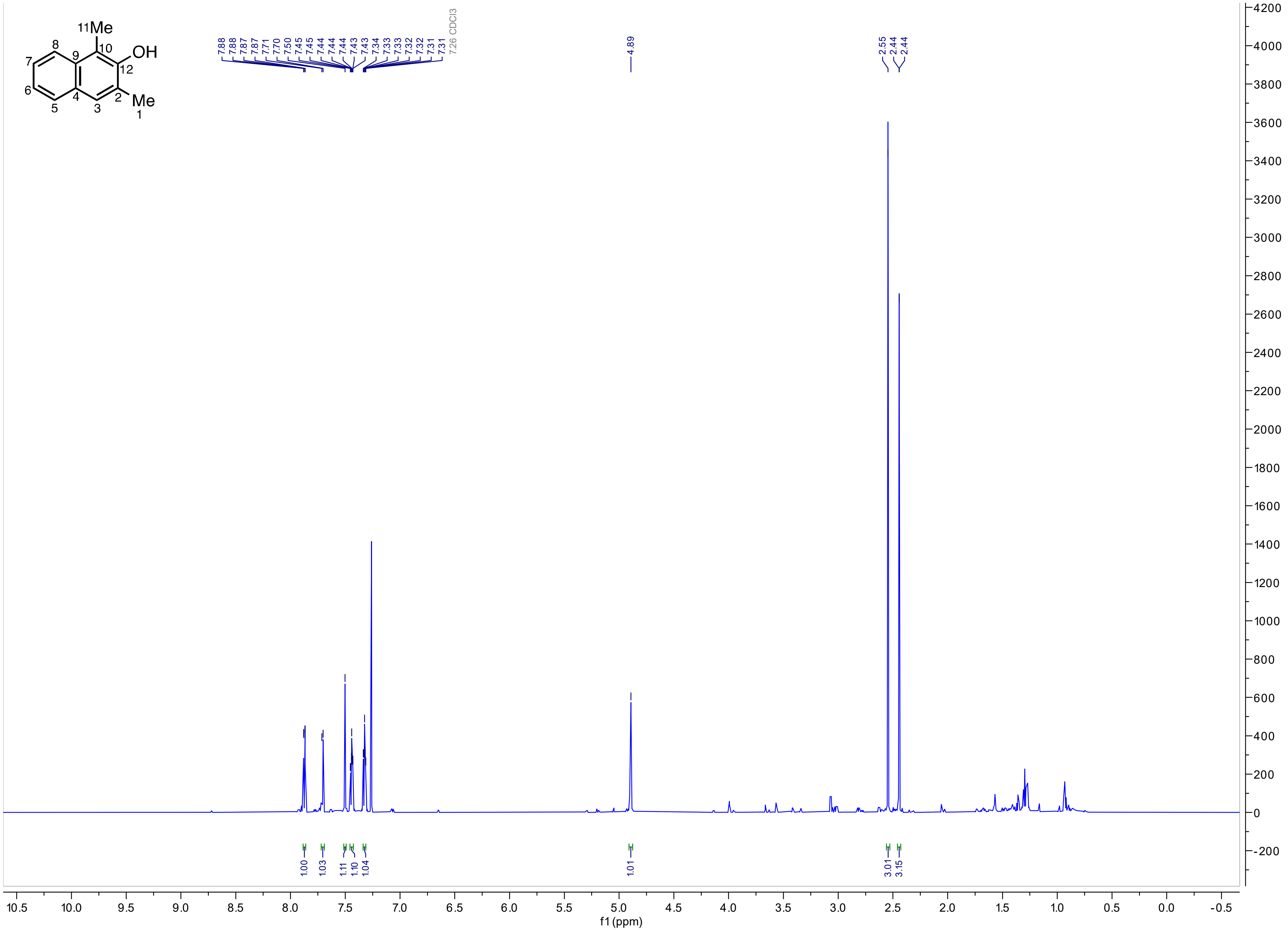

^{13}C NMR ($CDCl_3$): 1,3-Dimethylnaphthalen-2-ol (S26)

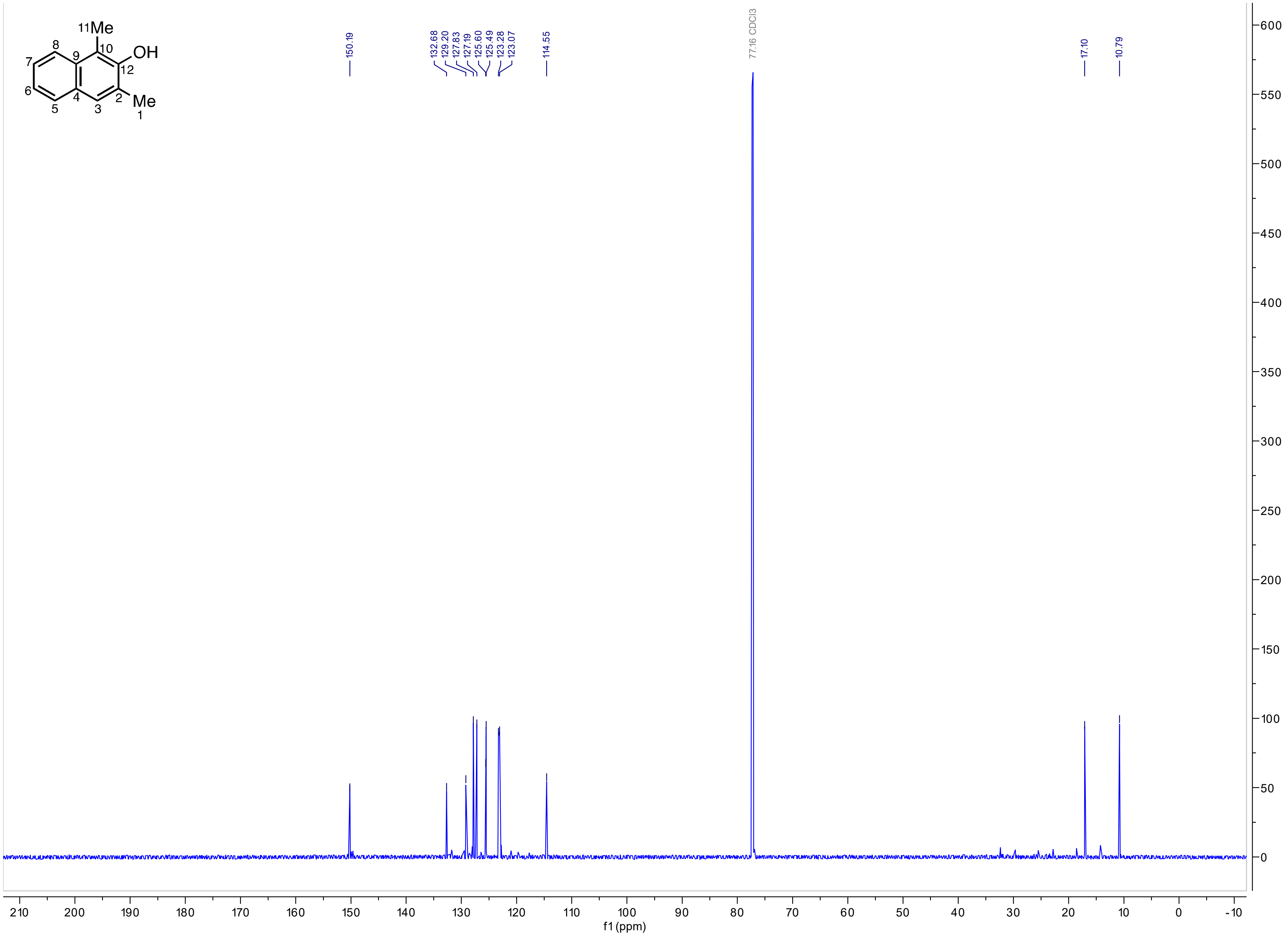

^{1}H NMR (CDCl$_3$): 3-Methoxy-4-methylphenol (**S27**)

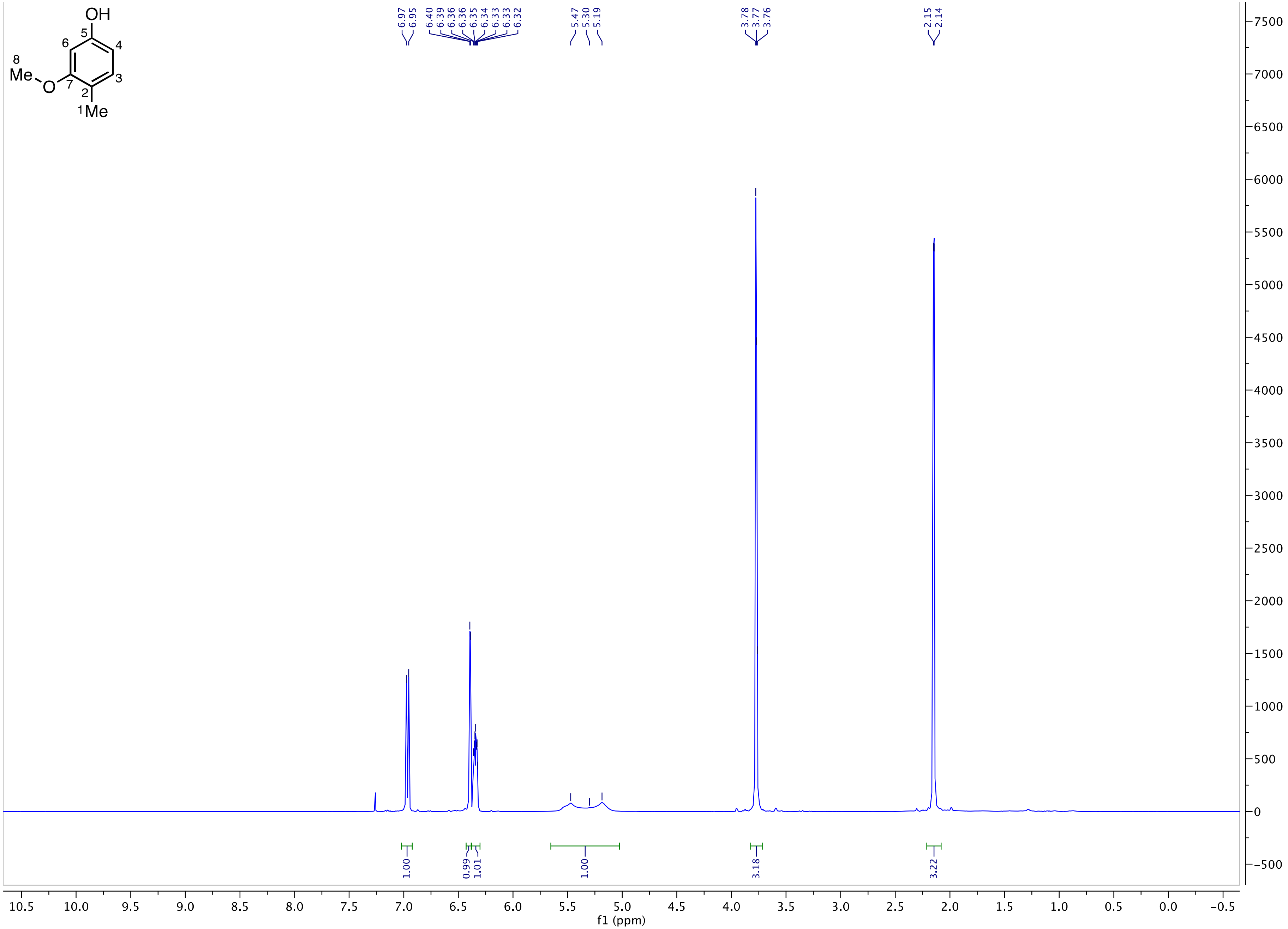

^{13}C NMR ($CDCl_3$): 3-Methoxy-4-methylphenol (S27)

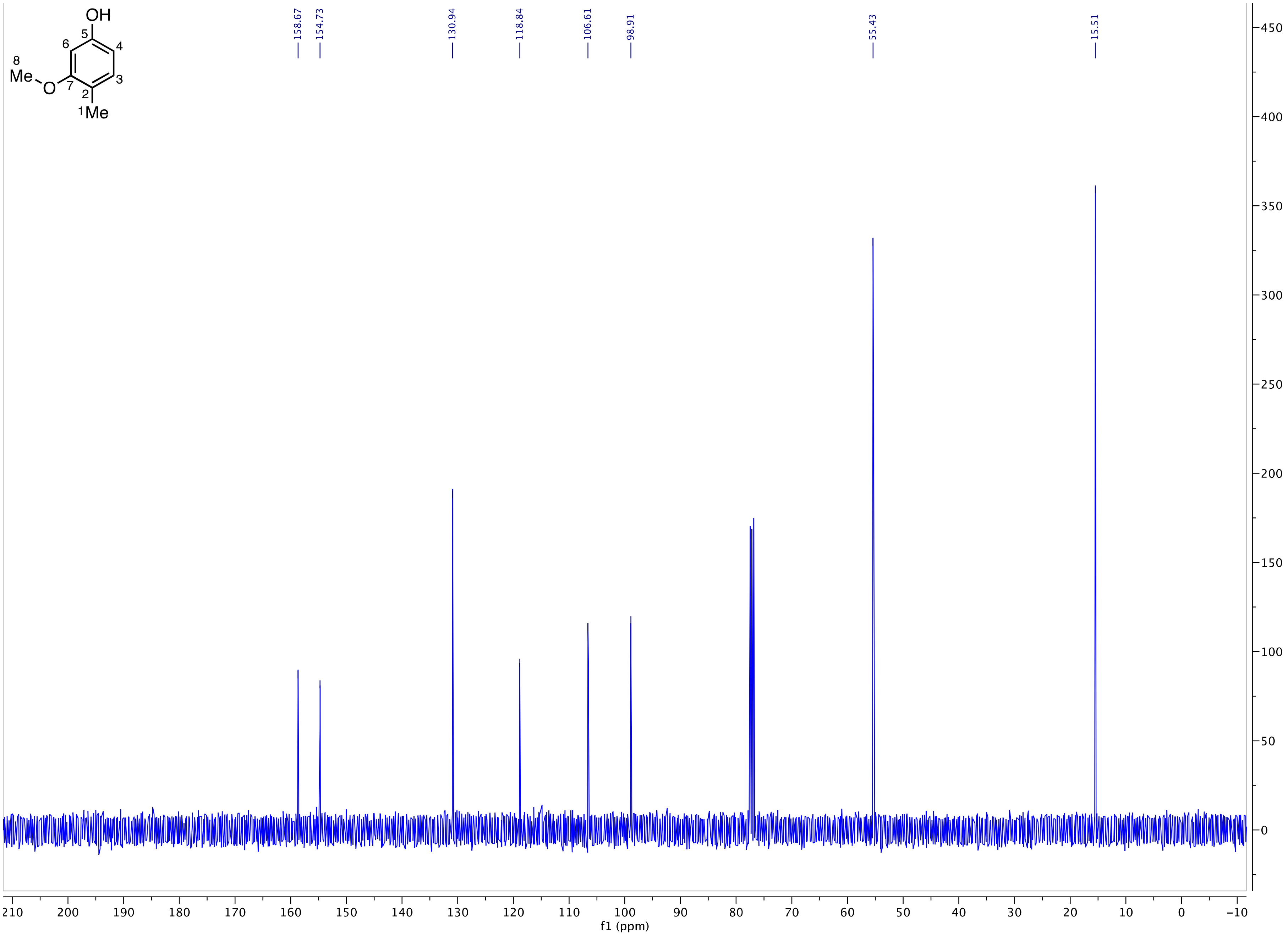

^{1}H NMR ($CDCl_3$): 2,4-Dimethylnaphthalen-1-ol (S28)

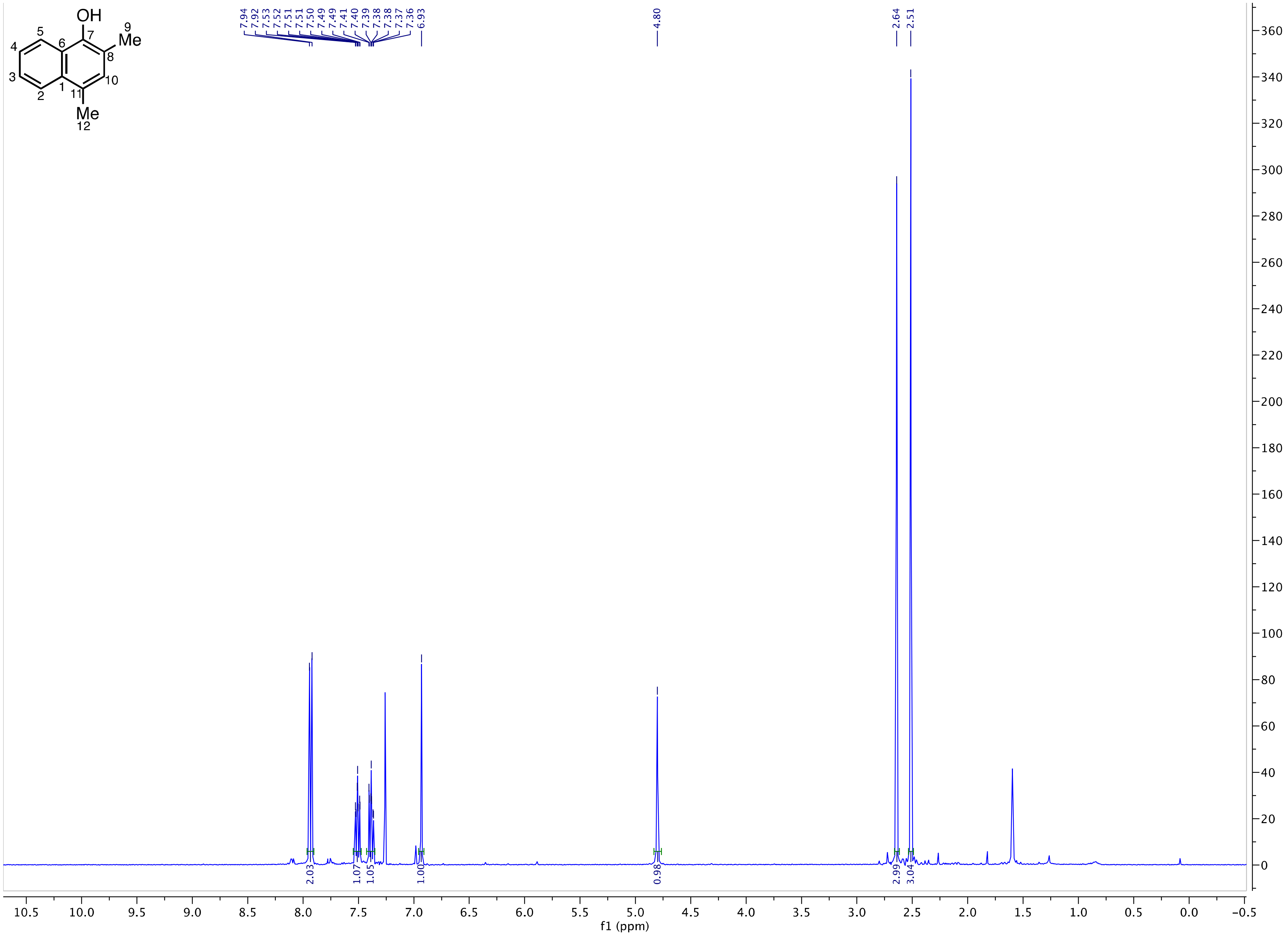

^{13}C NMR ($CDCl_3$): 2,4-Dimethylnaphthalen-1-ol (**S28**)

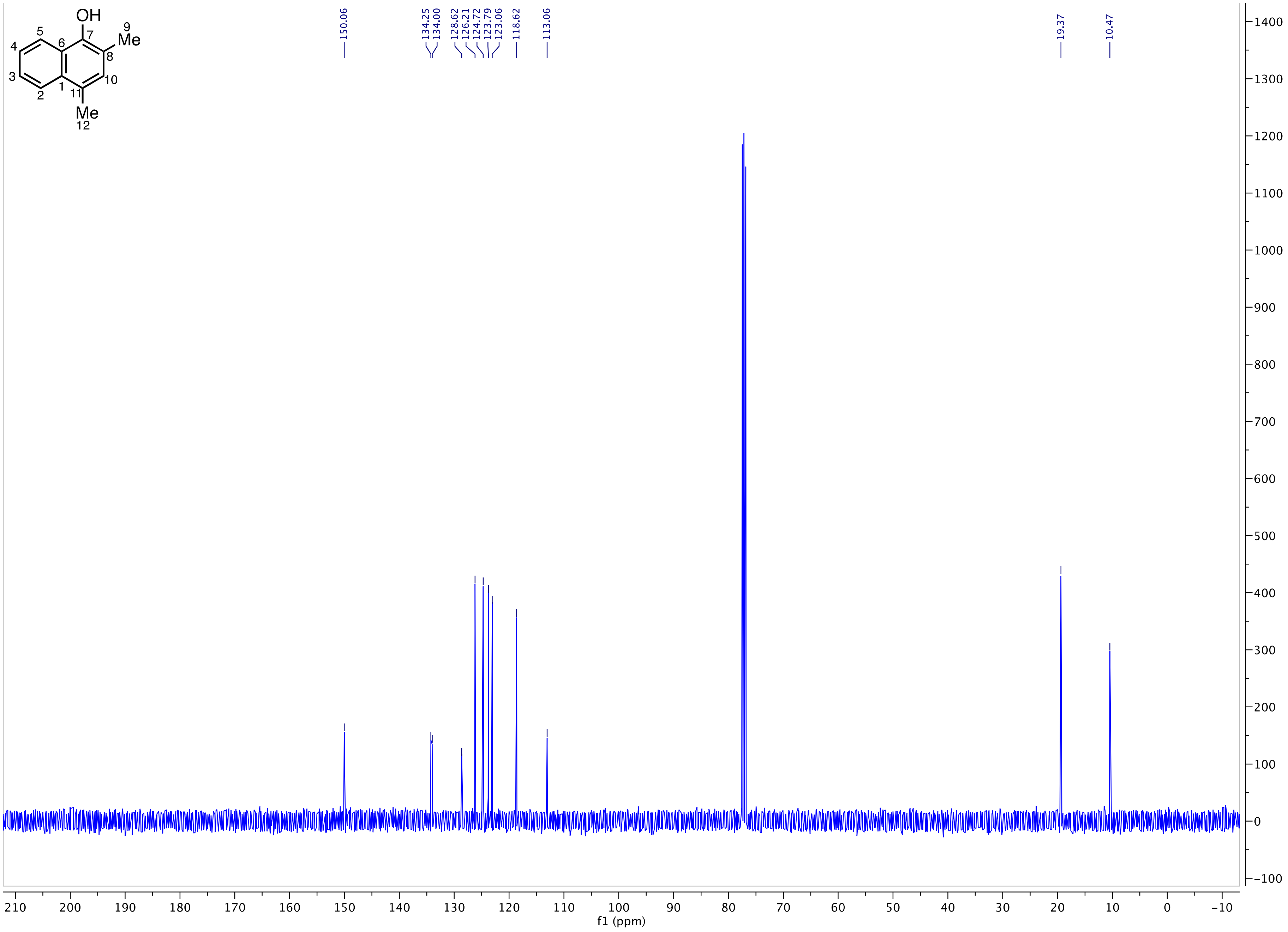

^{1}H NMR ($CDCl_3$): 2-Chloro-4-methylnaphthalen-1-ol (**S29**)

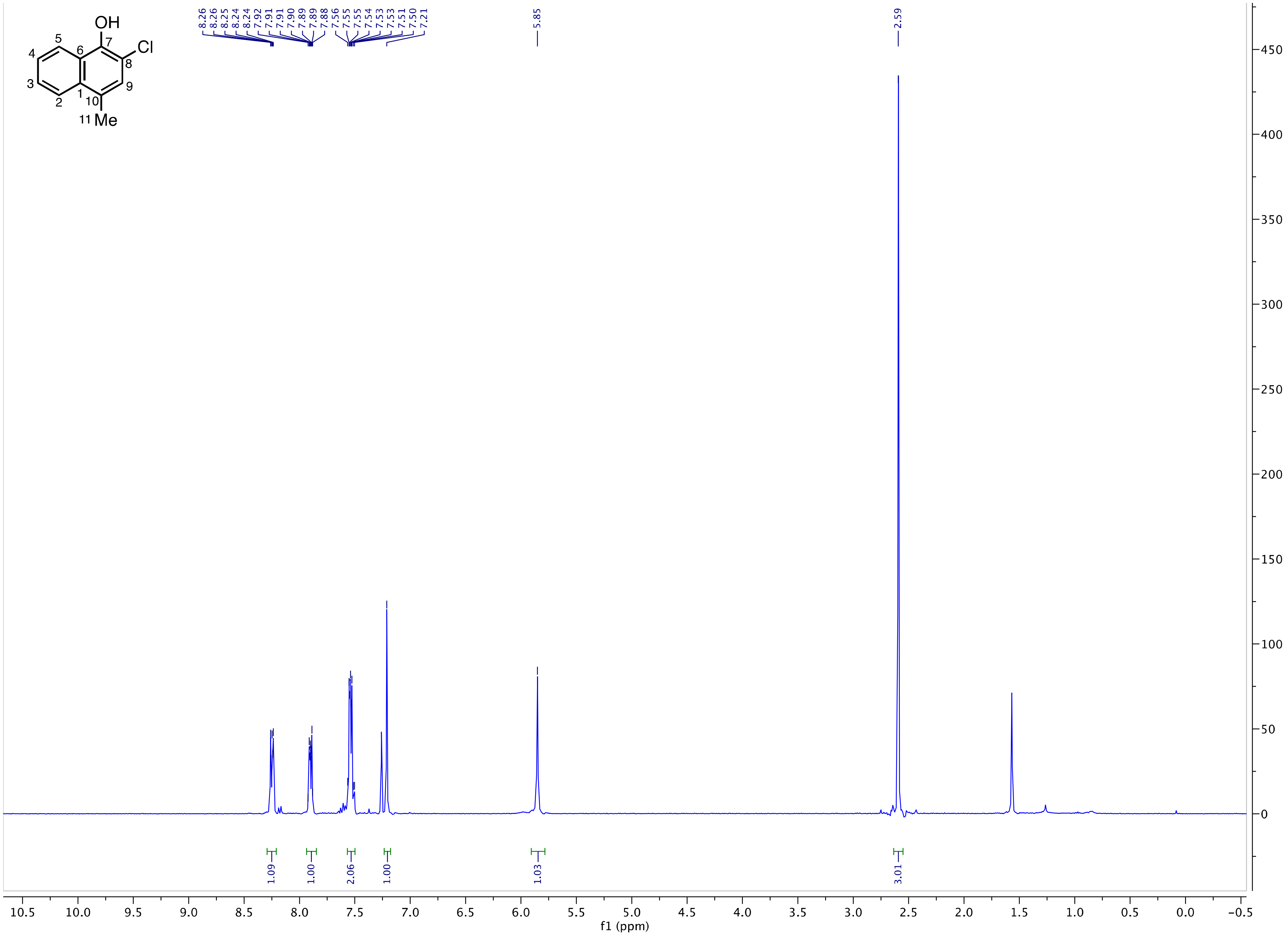

^{13}C NMR ($CDCl_3$): 2-Chloro-4-methylnaphthalen-1-ol (**S29**)

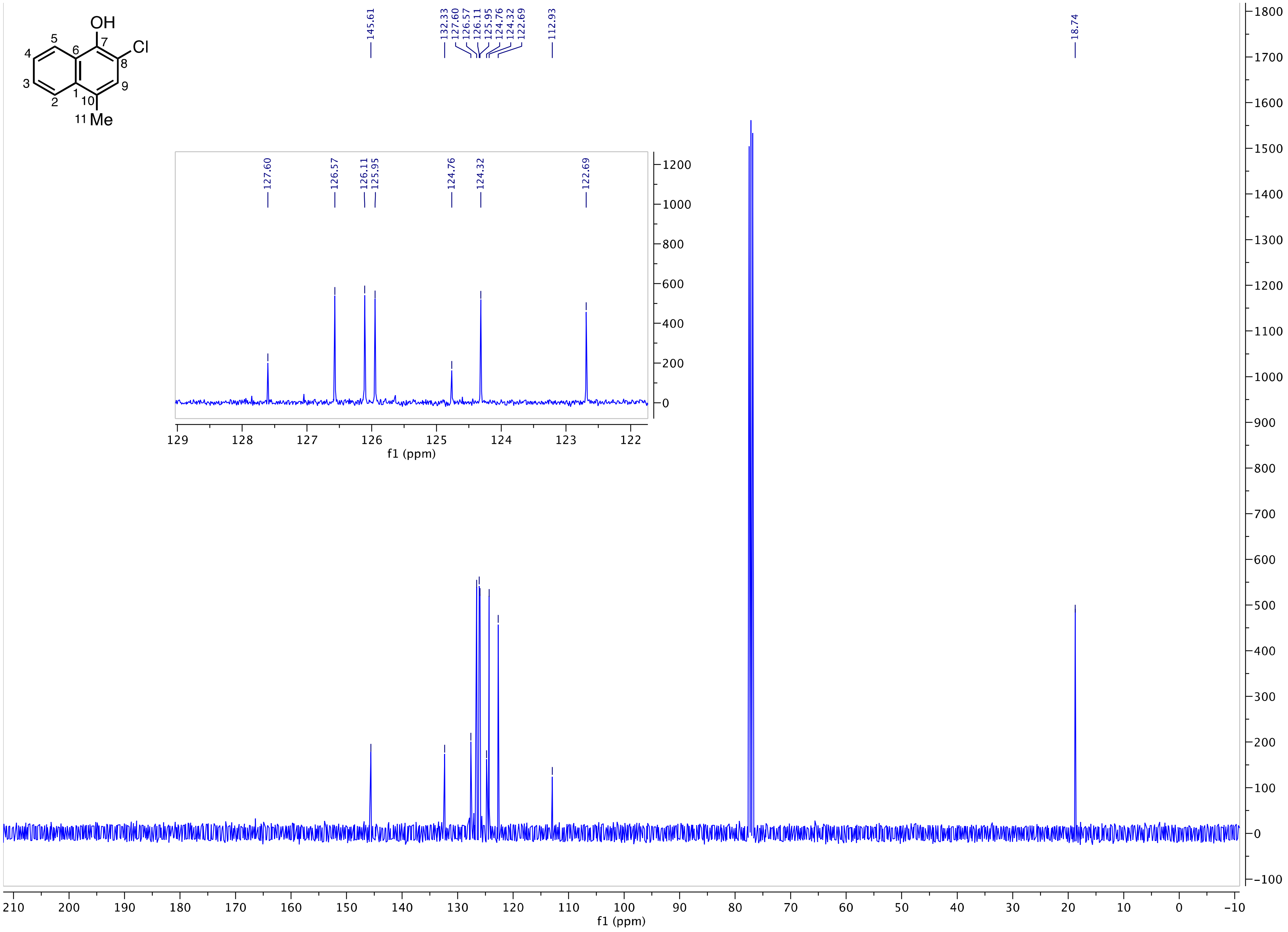

^{1}H NMR ($CDCl_3$): *tert*-Butyl (4-methylnaphthalen-1-yl)carbamate (**S30**)

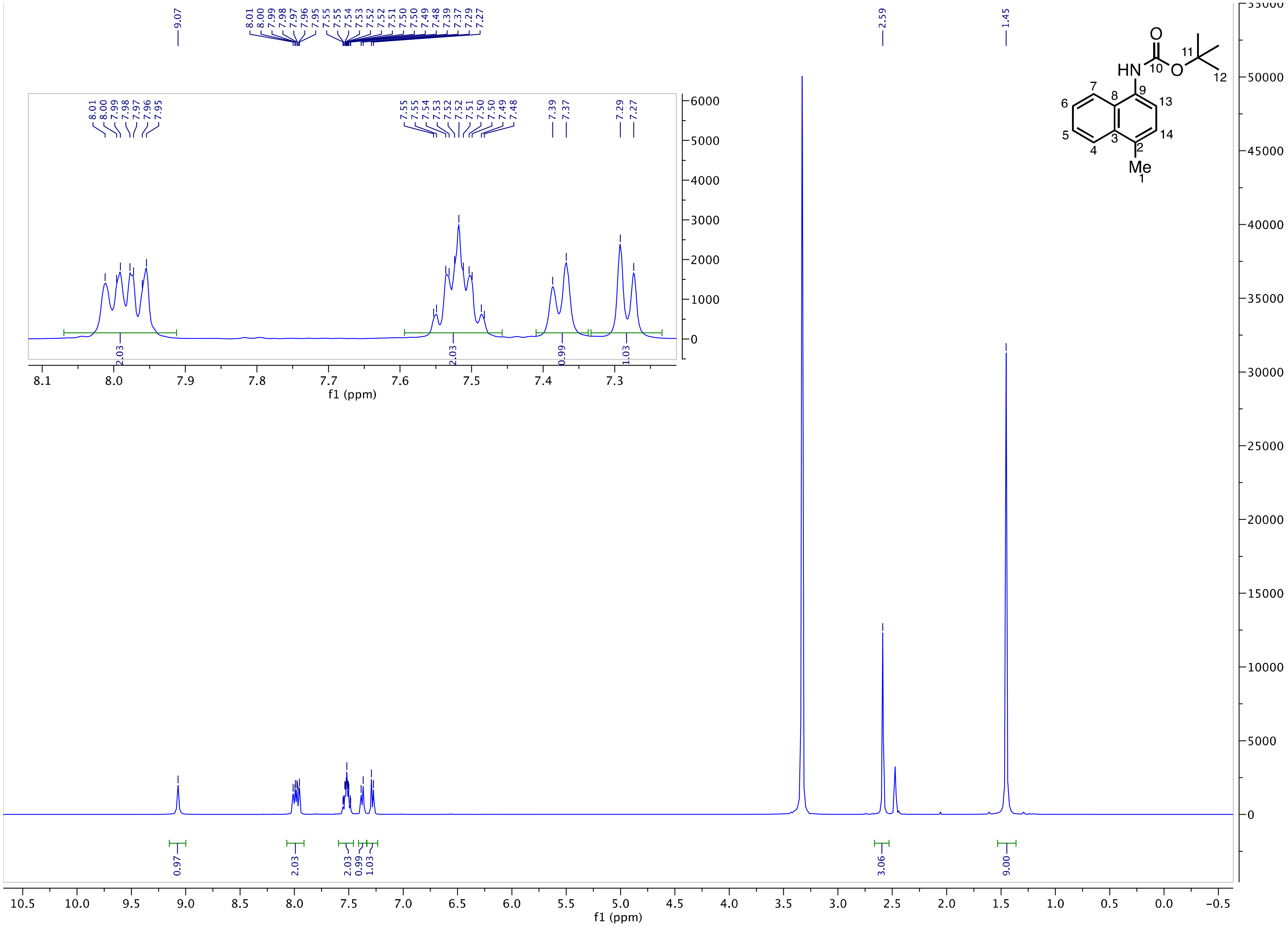

^{13}C NMR ($CDCl_3$): *tert*-Butyl (4-methylnaphthalen-1-yl)carbamate (**S30**)

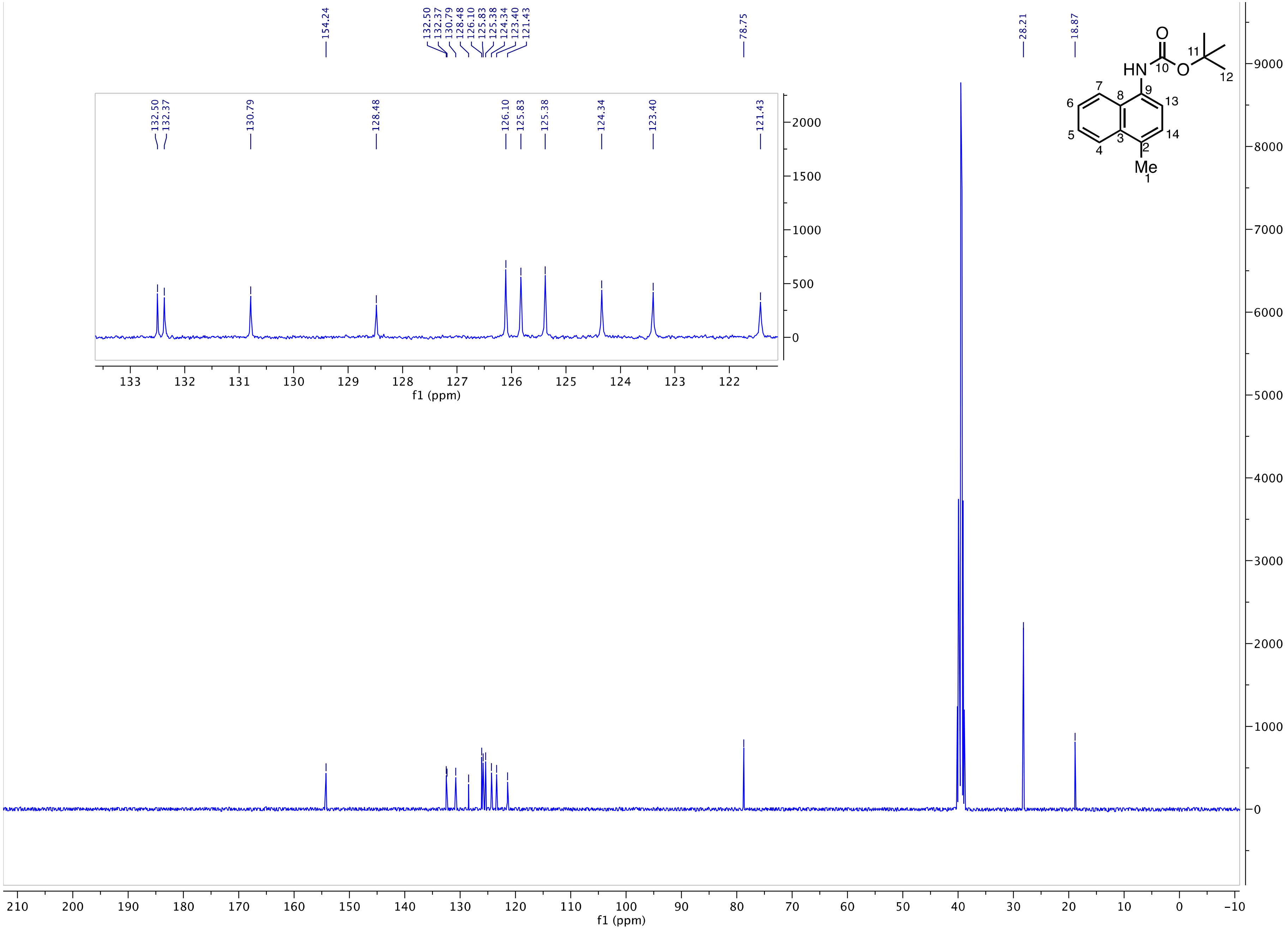

^{1}H NMR ($CDCl_3$): *N*-(1,2-dihydroacenaphthylen-5-yl)-4-Methylbenzenesulfonamide (**S31**)

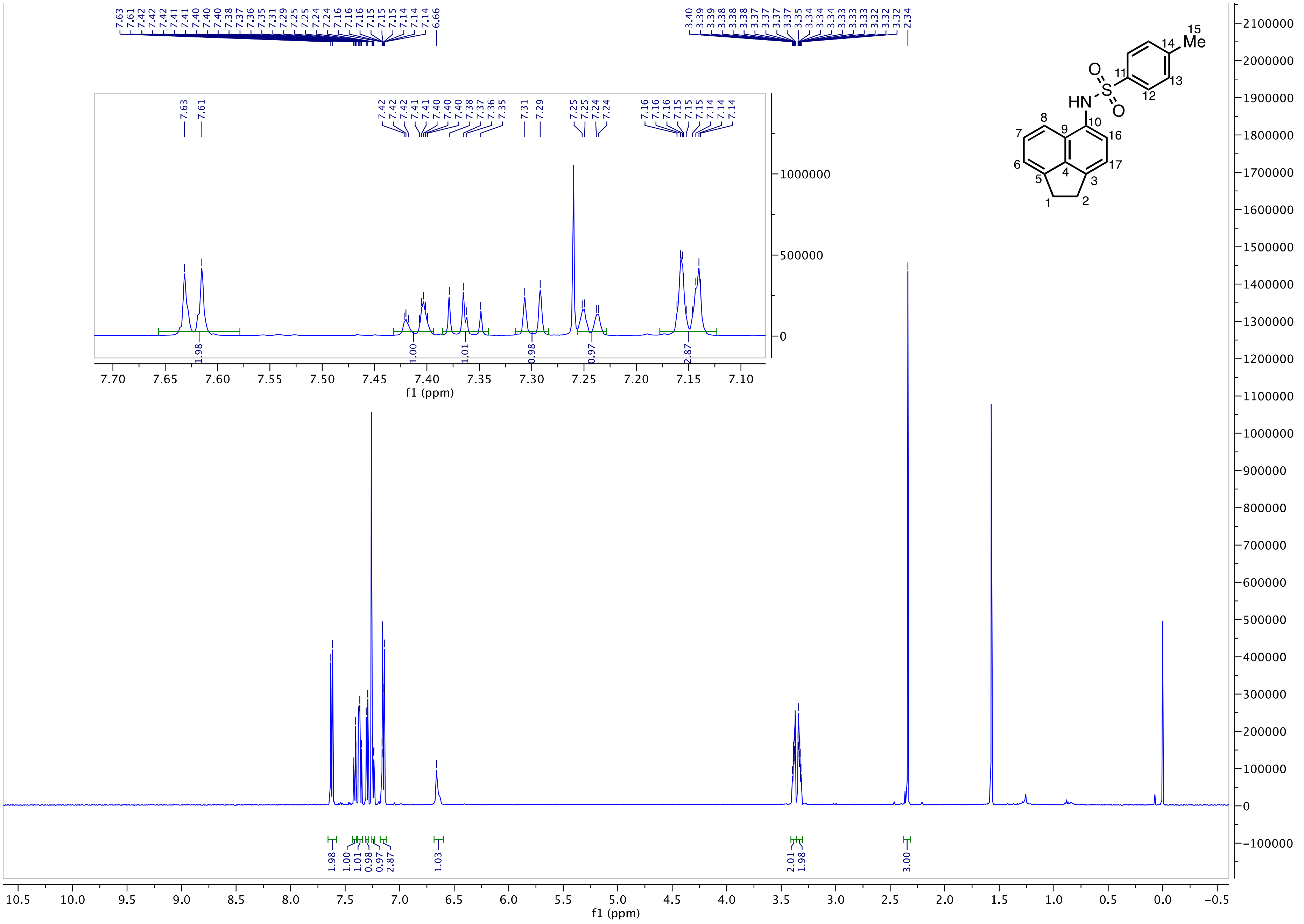

^{13}C NMR ($CDCl_3$): *N*-(1,2-dihydroacenaphthylen-5-yl)-4-Methylbenzenesulfonamide (**S31**)

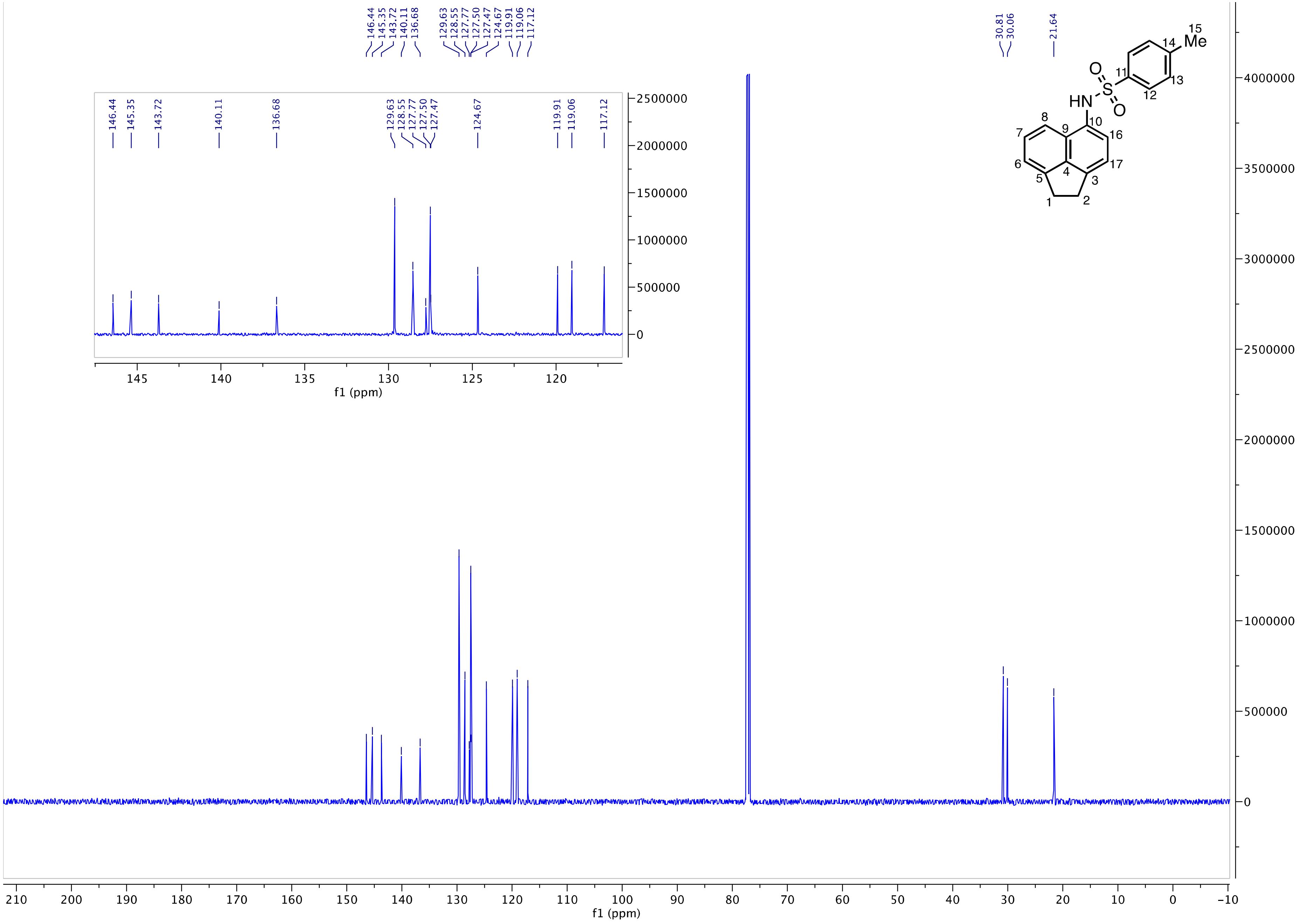

(*R*)-4-Methyl-4-phenylnaphthalen-1(4*H*)-one (2a) (0.1 mmol scale)

Chiral SFC Analysis: CHIRALPAK IE (CO_2:MeOH, 90:10, 2.5 mL min^{-1}, 40 °C, 250 nm) indicated 97% *ee*, t_R = 5.33 (minor), 5.68 (major) minutes.

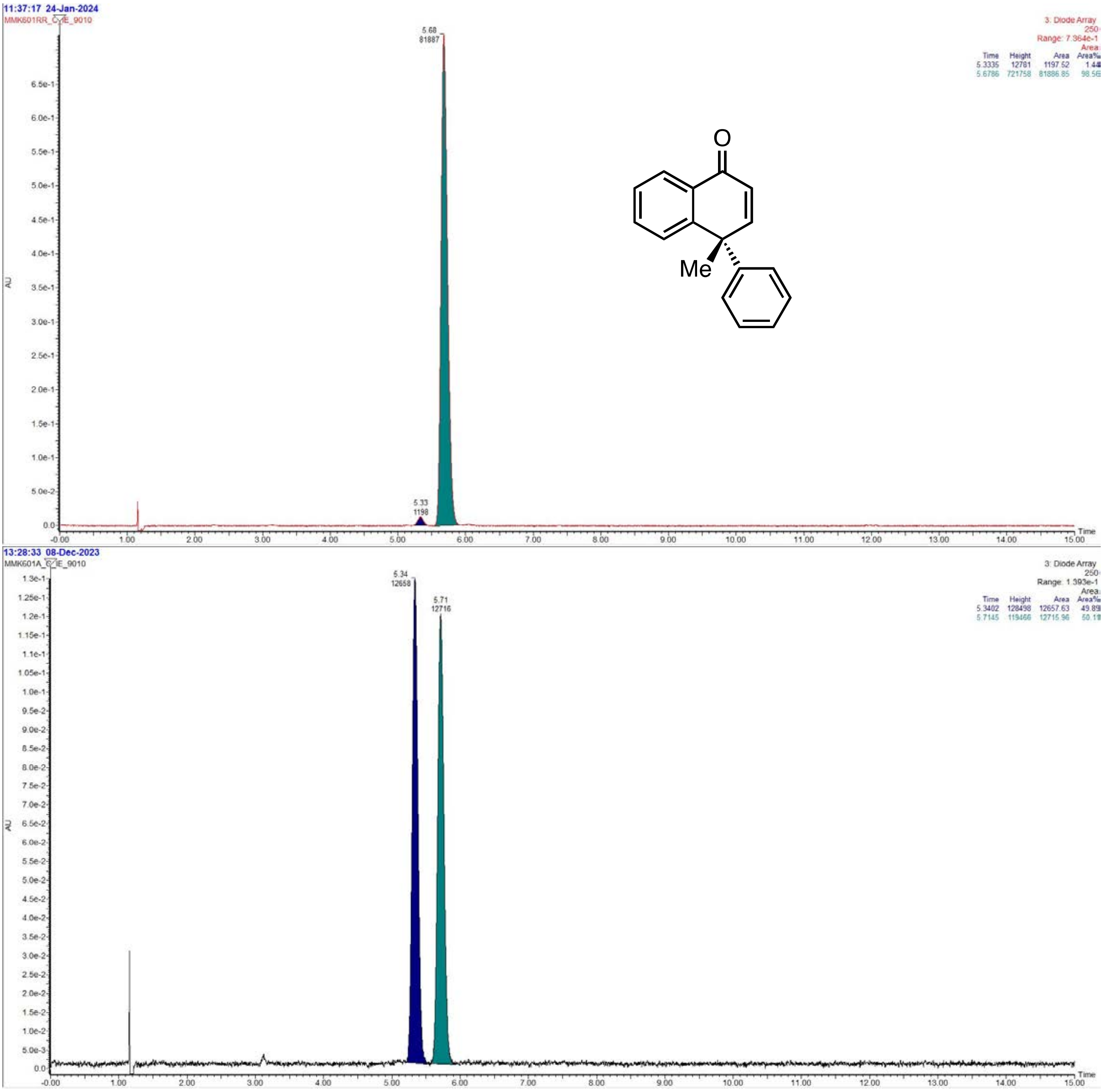

(*R*)-4-Methyl-4-phenylnaphthalen-1(4*H*)-one (2a) (1 mmol scale)

Chiral SFC Analysis: CHIRALPAK IE (CO_2:MeOH, 90:10, 2.5 mL min^{-1}, 40 °C, 250 nm) indicated 98% *ee*, t_R = 5.22 (minor), 5.53 (major) minutes.

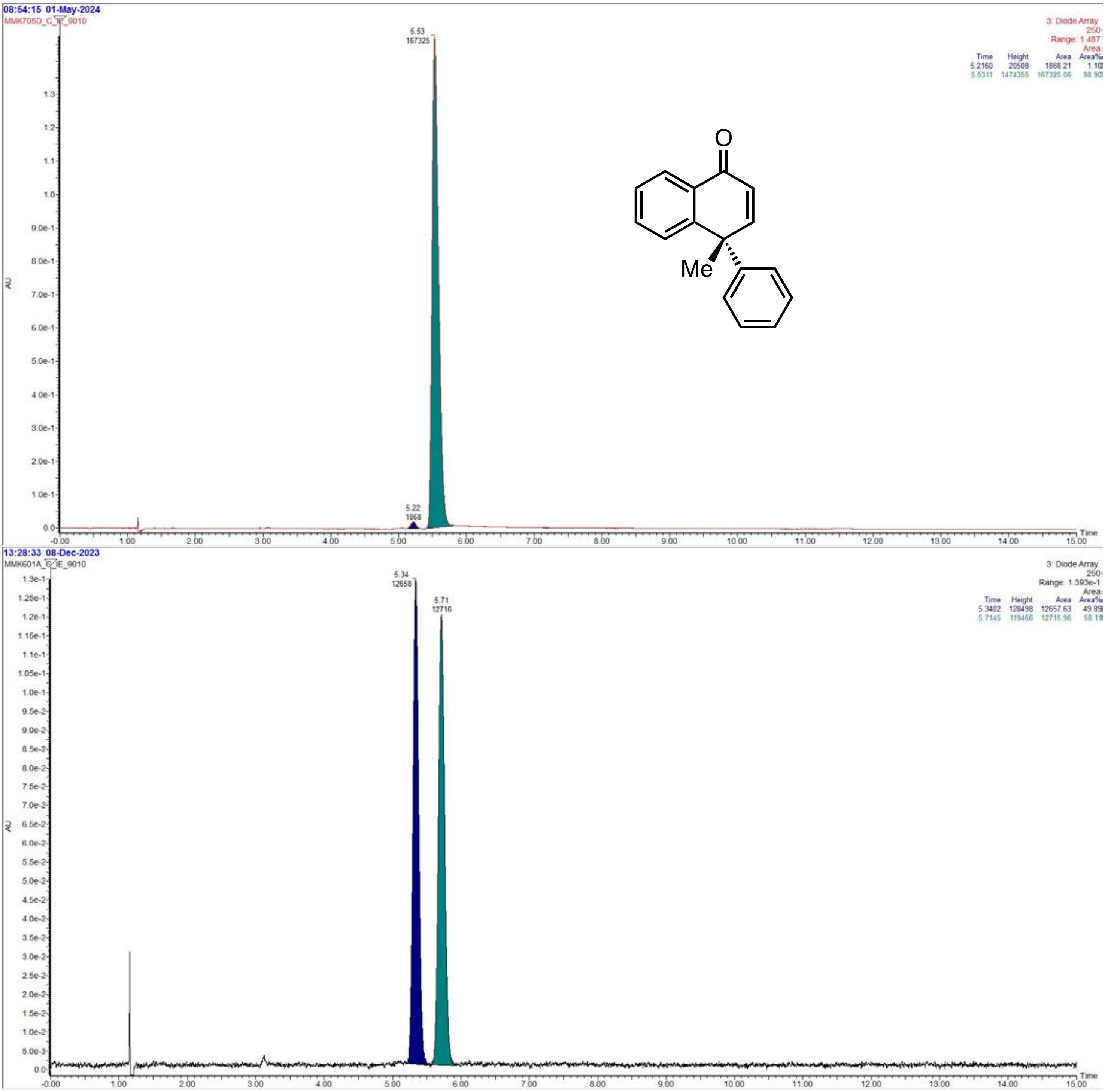

(*R*)-4-Ethyl-4-phenylnaphthalen-1(4*H*)-one (2b)

Chiral SFC Analysis: CHIRALPAK IE (CO_2:MeOH, 90:10, 2.5 mL min^{-1}, 40 °C, 250 nm) indicated 98% *ee*, t_R = 5.51 (minor), 6.31 (major) minutes.

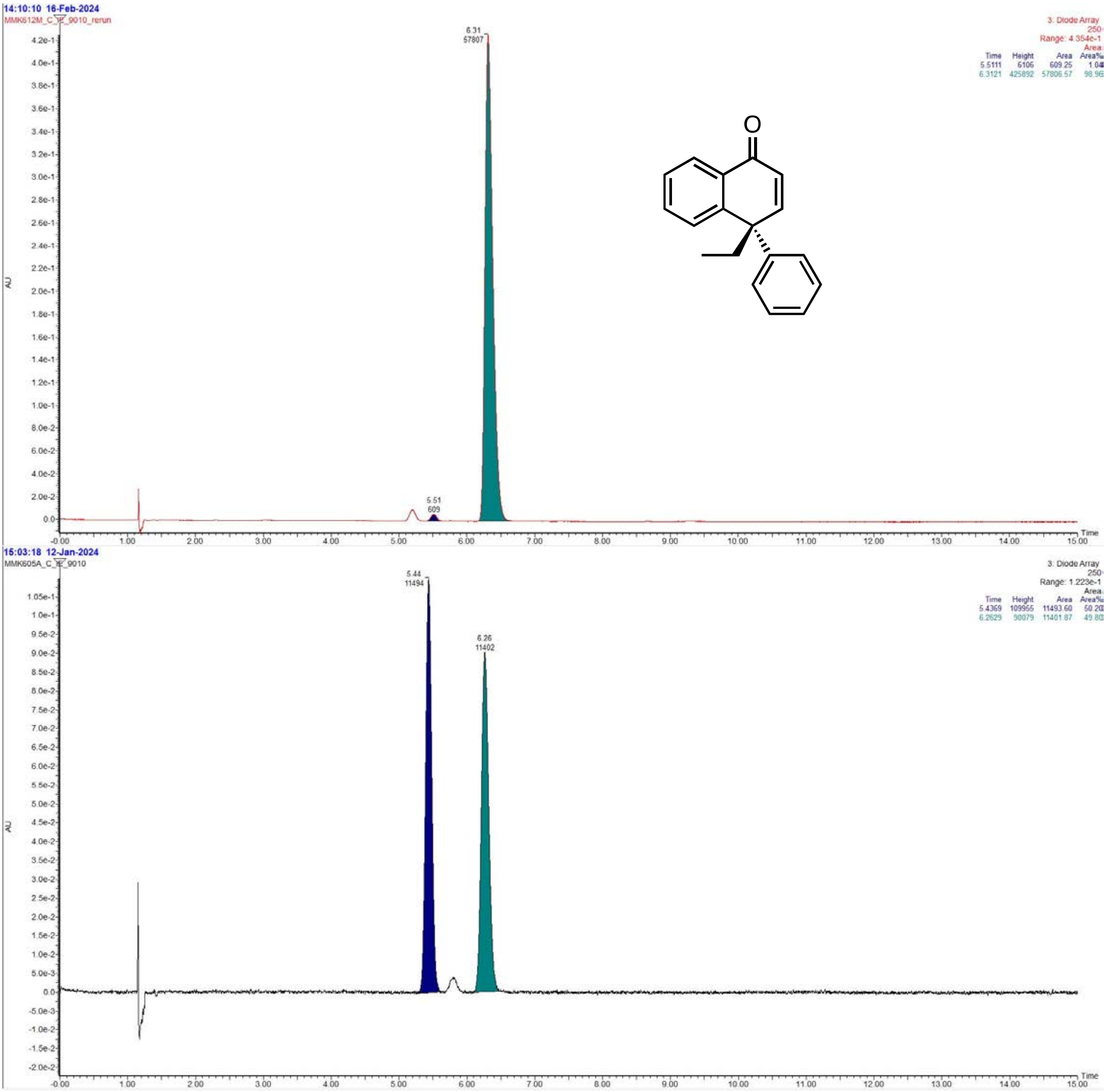

(*R*)-4-Hexyl-4-phenylnaphthalen-1(4*H*)-one (2c)

Chiral SFC Analysis: CHIRALPAK IE (CO_2:MeOH, 90:10, 2.5 mL min^{-1}, 40 °C, 250 nm) indicated 75% *ee*, t_R = 5.24 (minor), 5.56 (major) minutes.

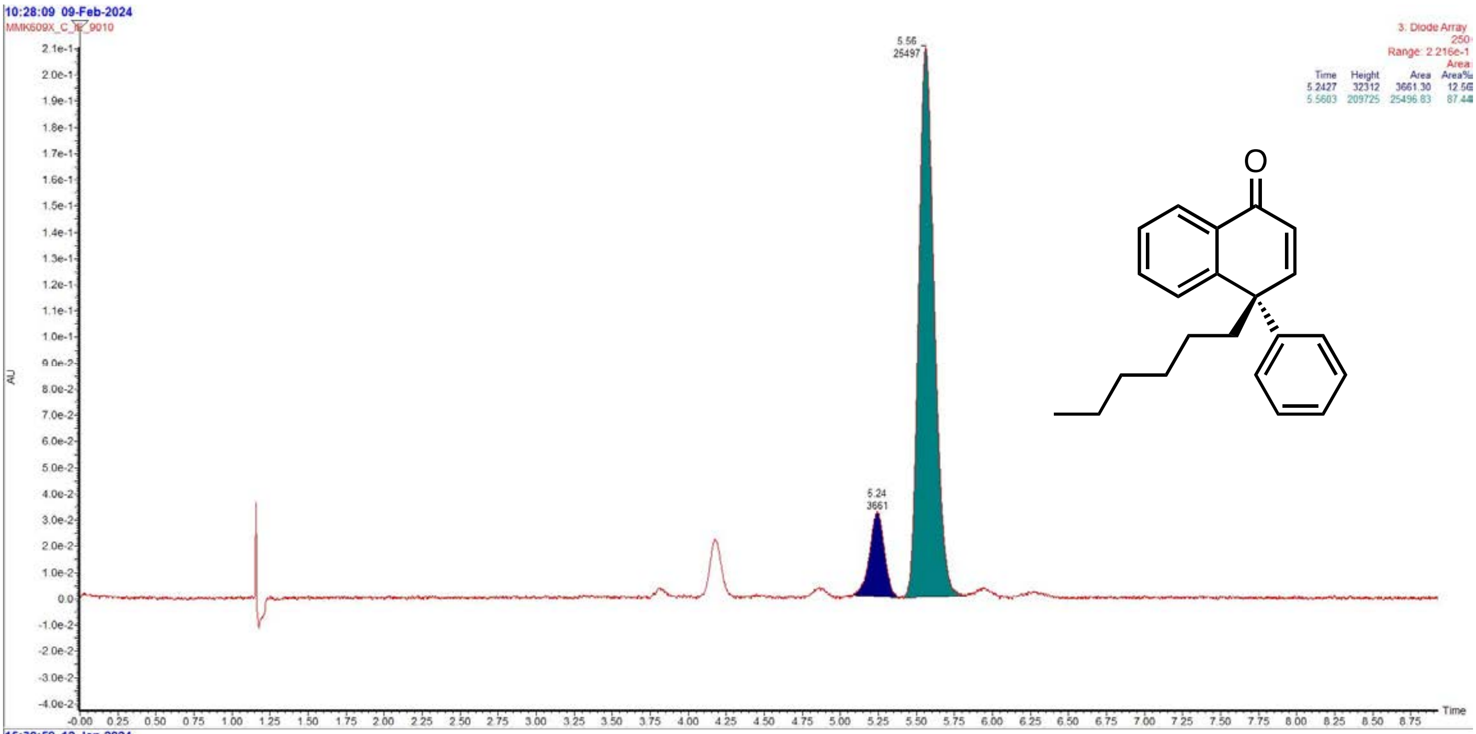

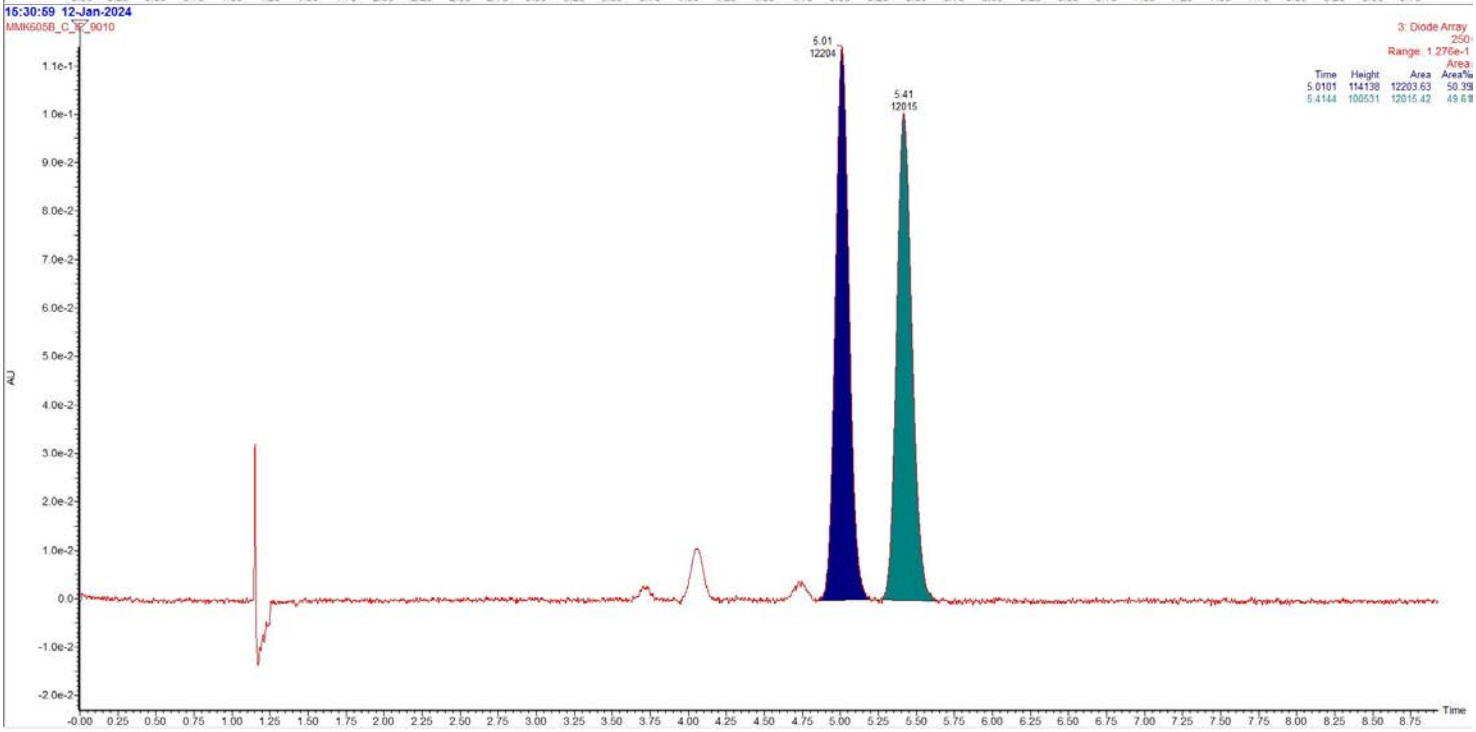

(*R*)-4-Phenethyl-4-phenylnaphthalen-1(4*H*)-one (2d)

Chiral SFC Analysis: CHIRALPAK IK (CO_2:MeOH, 90:10, 2.5 mL min^{-1}, 40 °C, 250 nm) indicated 96% *ee*, t_R = 8.40 (major), 9.41 (minor) minutes.

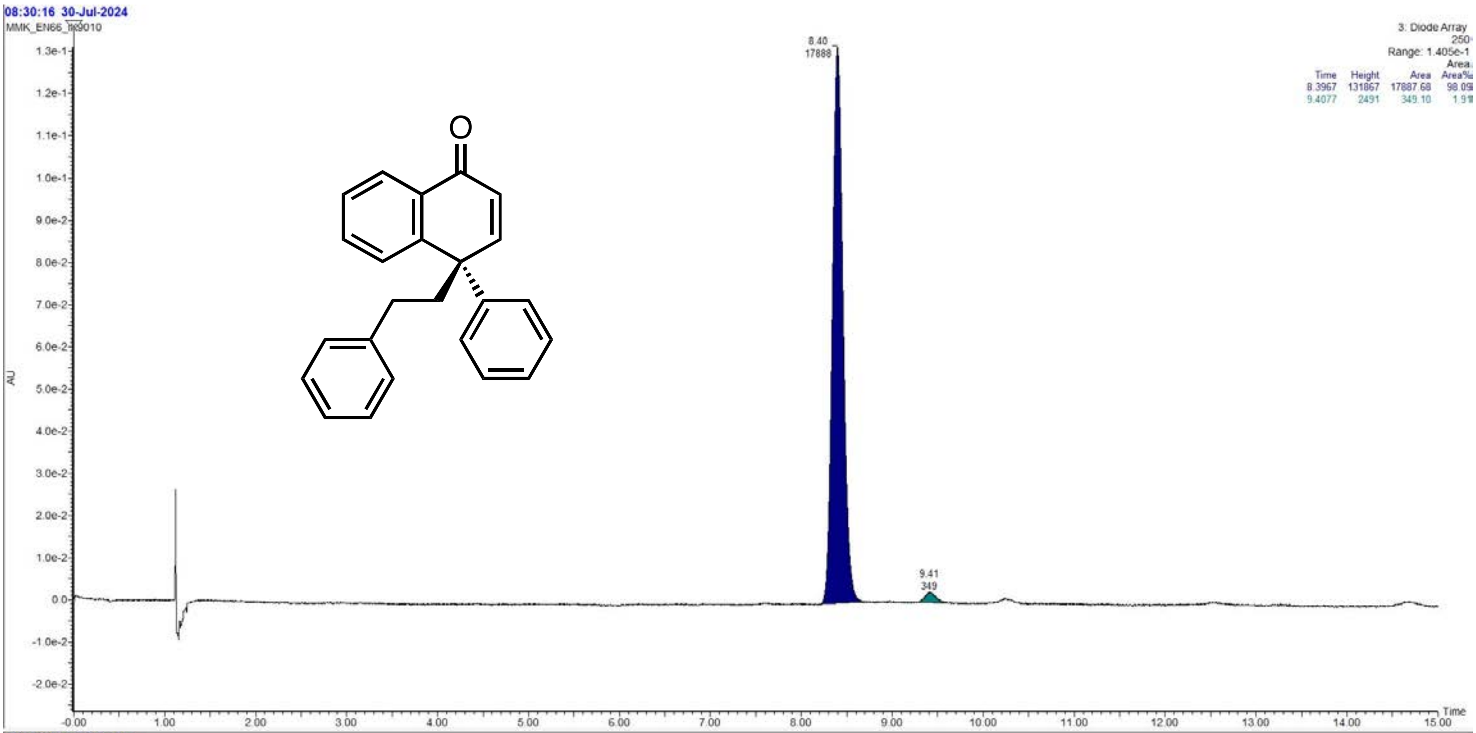

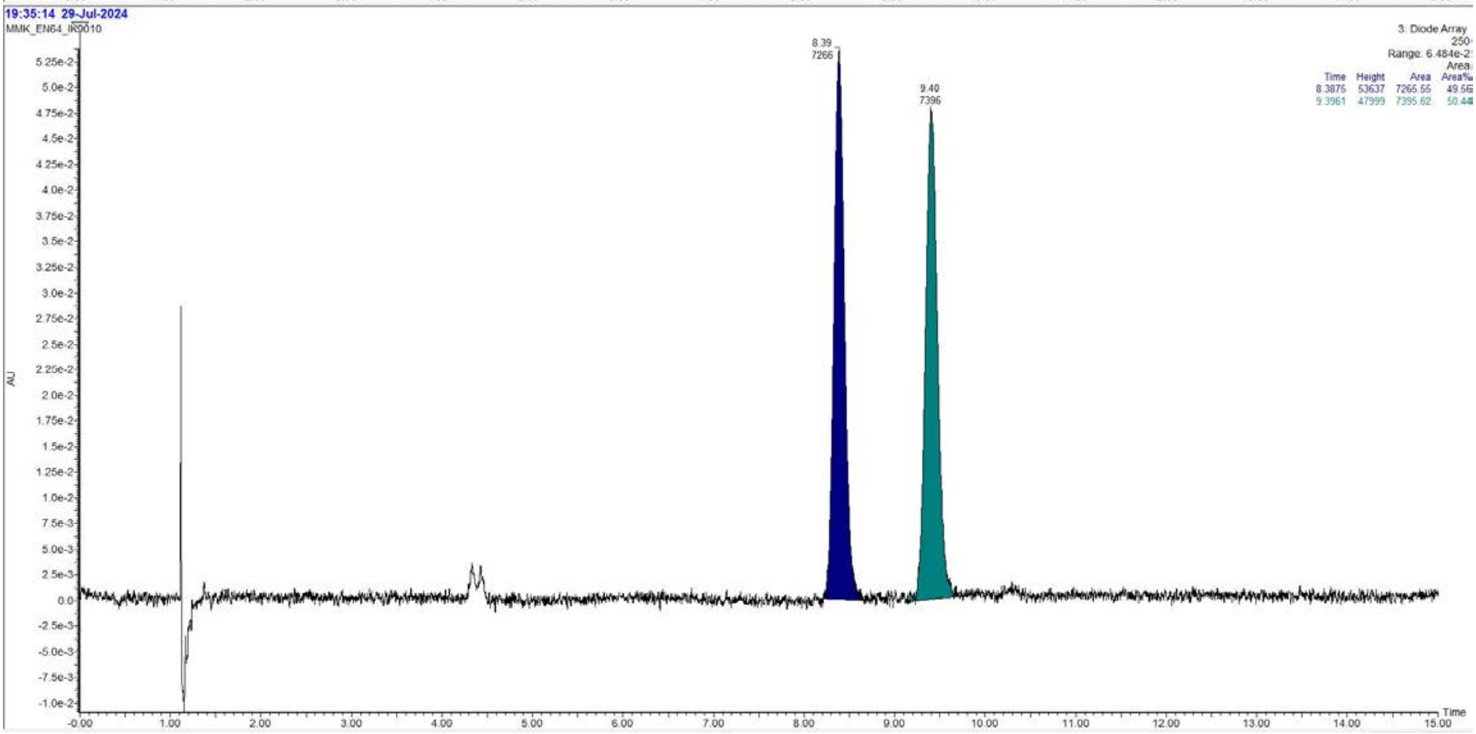

(*R*)-2a-Phenyl-2,2a-dihydroacenaphthylen-5(1*H*)-one (2e)

Chiral SFC Analysis: CHIRALPAK IJ (CO_2:MeOH, 90:10, 2.5 mL min^{-1}, 40 °C, 260 nm) indicated 93% *ee*, t_R = 4.22 (major), 5.19 (minor) minutes.

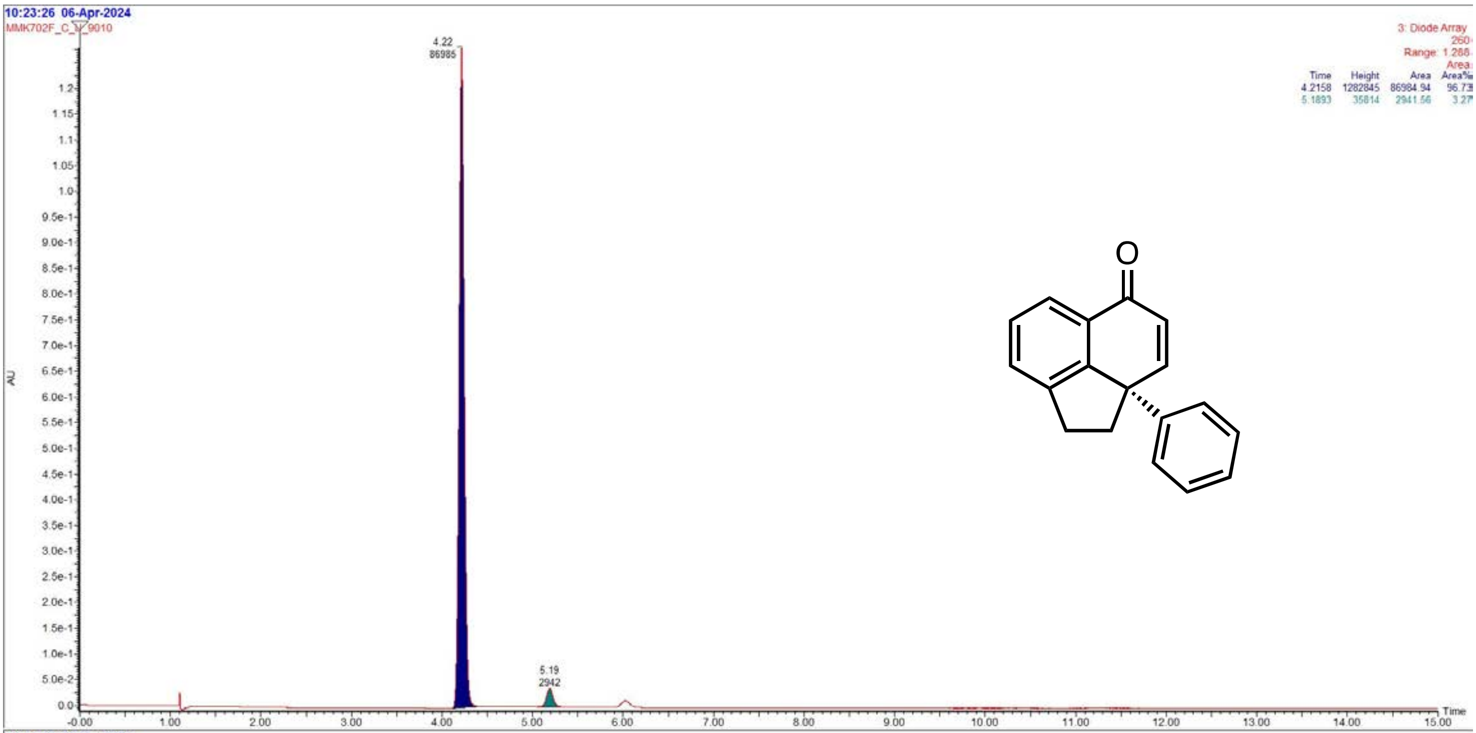

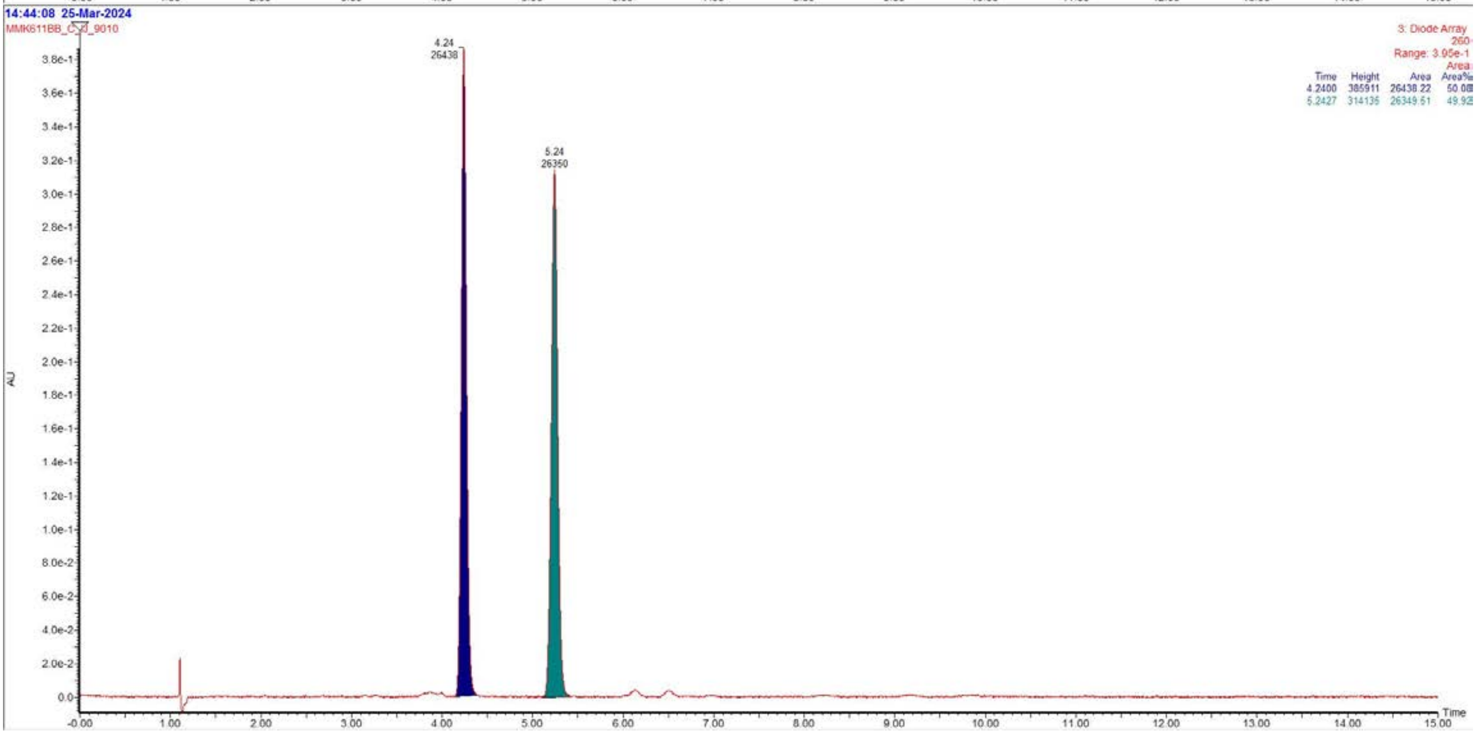

(*R*)-6-Methoxy-4-methyl-4-phenylnaphthalen-1(4*H*)-one (2f)

Chiral SFC Analysis: CHIRALPAK IJ (CO_2:MeOH, 90:10, 2.5 mL min^{-1}, 40 °C, 250 nm) indicated 93% *ee*, t_R = 3.37 (major), 3.67 (minor) minutes.

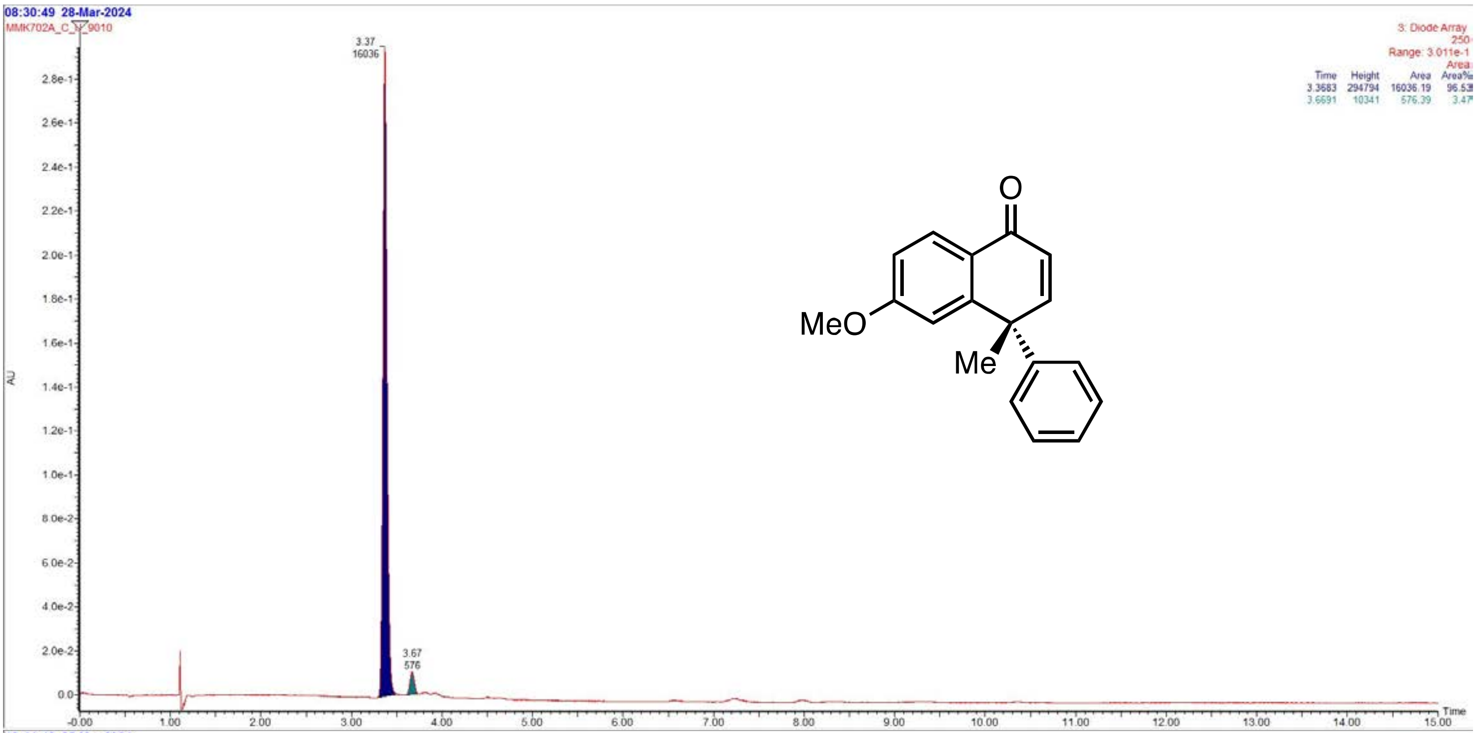

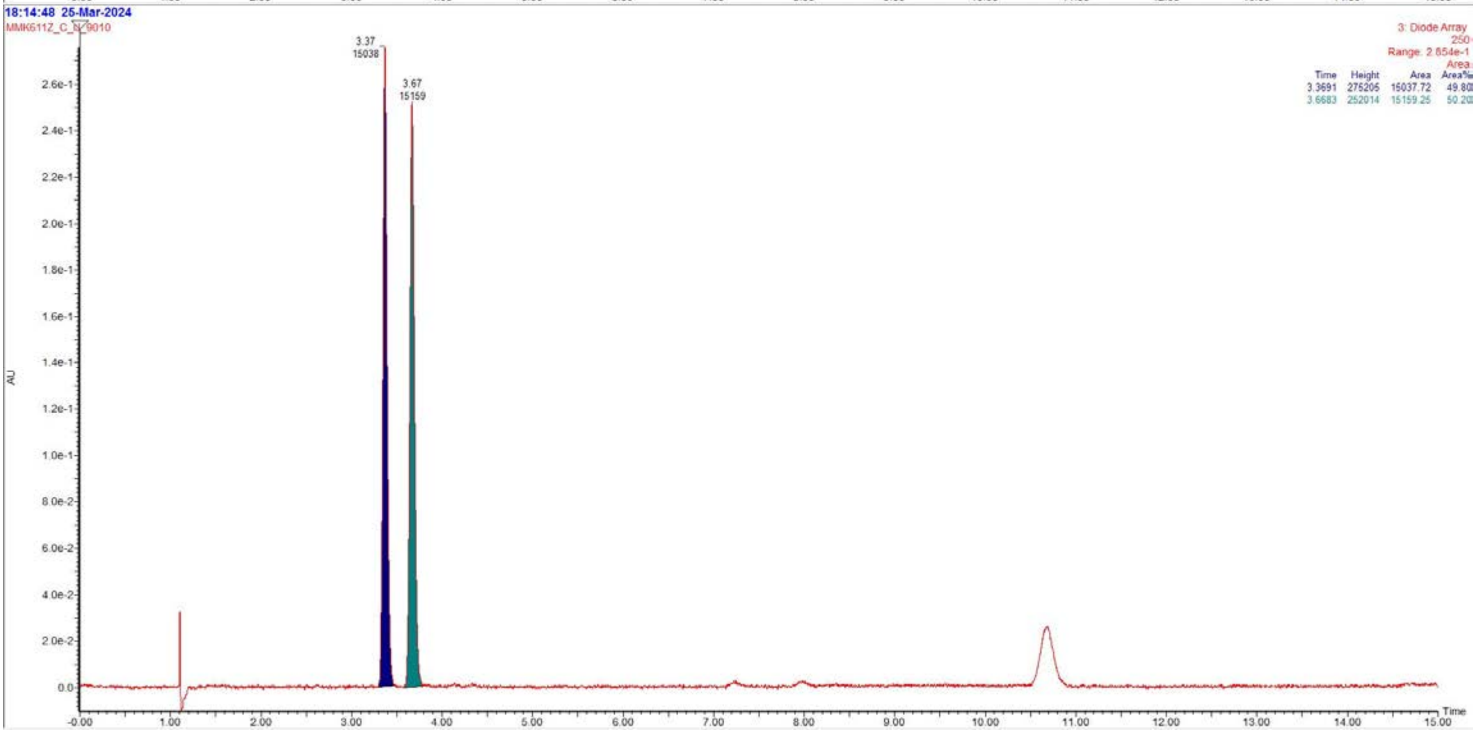

(*R*)-7-Methoxy-4-methyl-4-phenylnaphthalen-1(4*H*)-one (2g)

Chiral SFC Analysis: CHIRALPAK IE (CO_2:MeOH, 90:10, 2.5 mL min^{-1}, 40 °C, 250 nm) indicated 96% *ee*, t_R = 5.81 (minor), 6.22 (major) minutes.

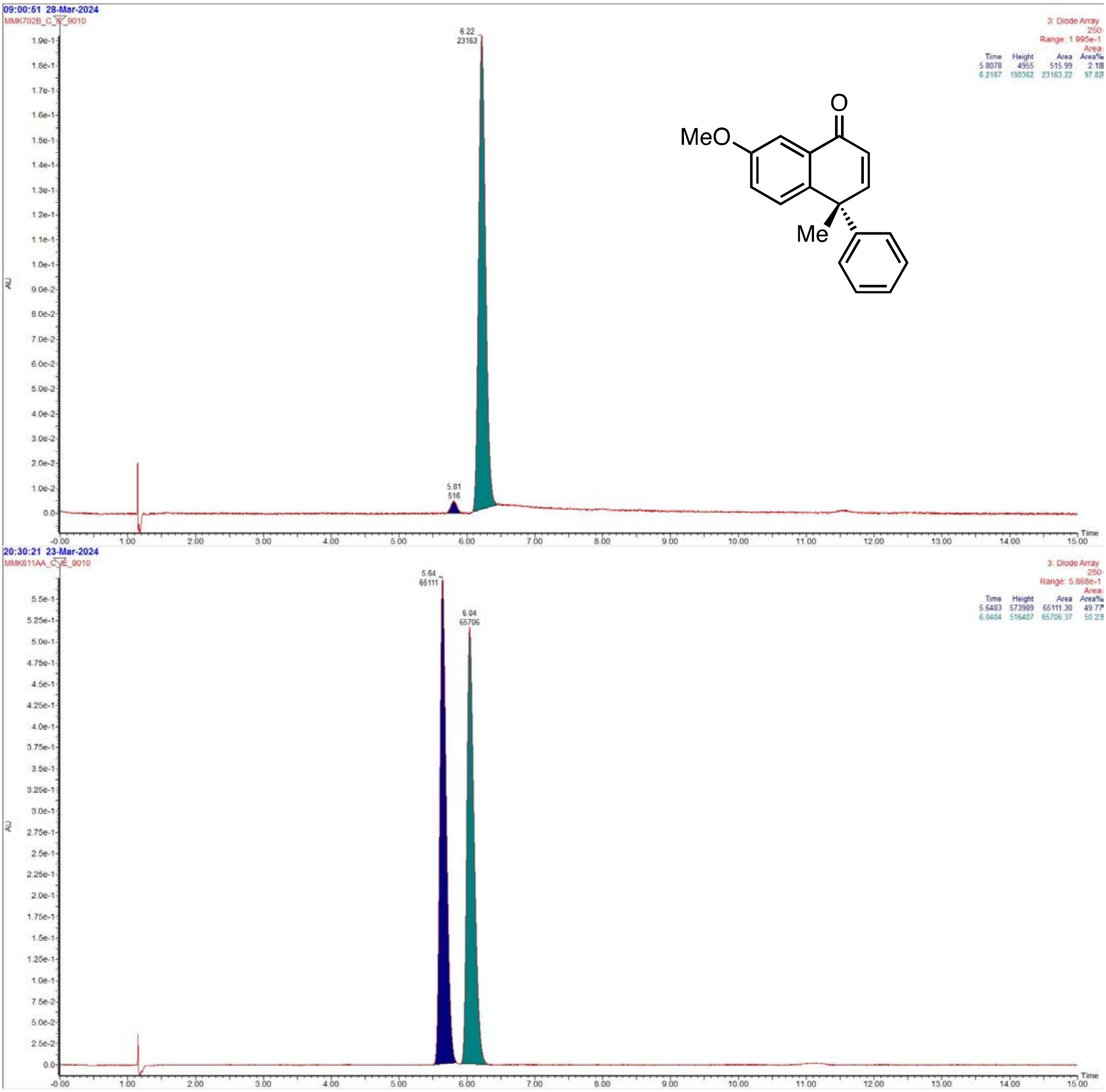

(*R*)-7-(methoxymethoxy)-4,6-Dimethyl-4-phenylnaphthalen-1(4*H*)-one (2h)

Chiral SFC Analysis: CHIRALPAK IA (CO_2:MeOH, 95:05, 2.5 mL min^{-1}, 40 °C, 250 nm) indicated 95% *ee*, t_R = 6.00 (minor), 6.65 (major) minutes.

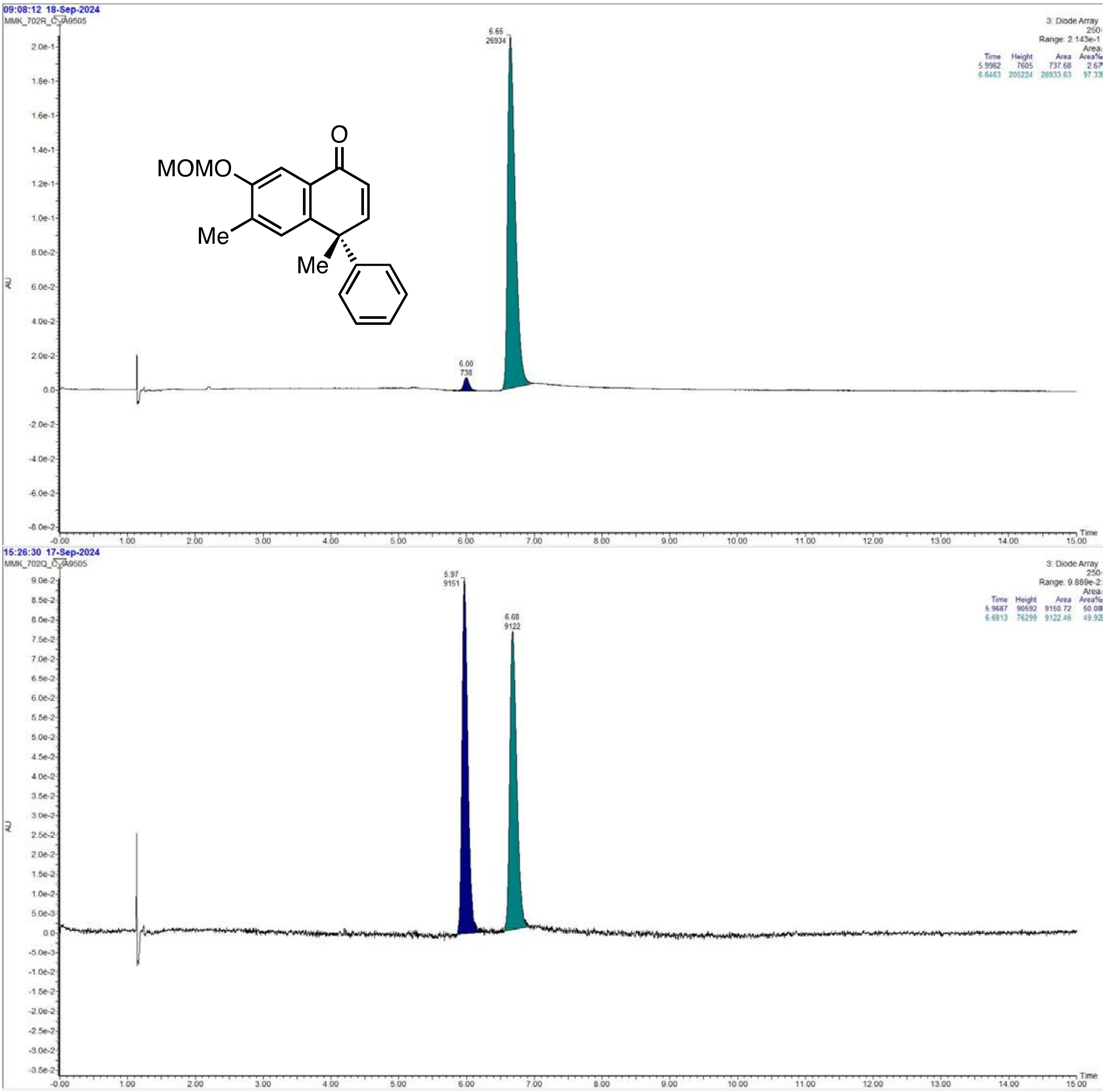

(*R*)-3,4-Dimethyl-4-phenylnaphthalen-1(4*H*)-one (2i)

Chiral SFC Analysis: CHIRALPAK IJ (CO_2:MeOH, 90:10, 2.5 mL min^{-1}, 40 °C, 260 nm) indicated 95% *ee*, t_R = 2.63 (major), 3.05 (minor) minutes.

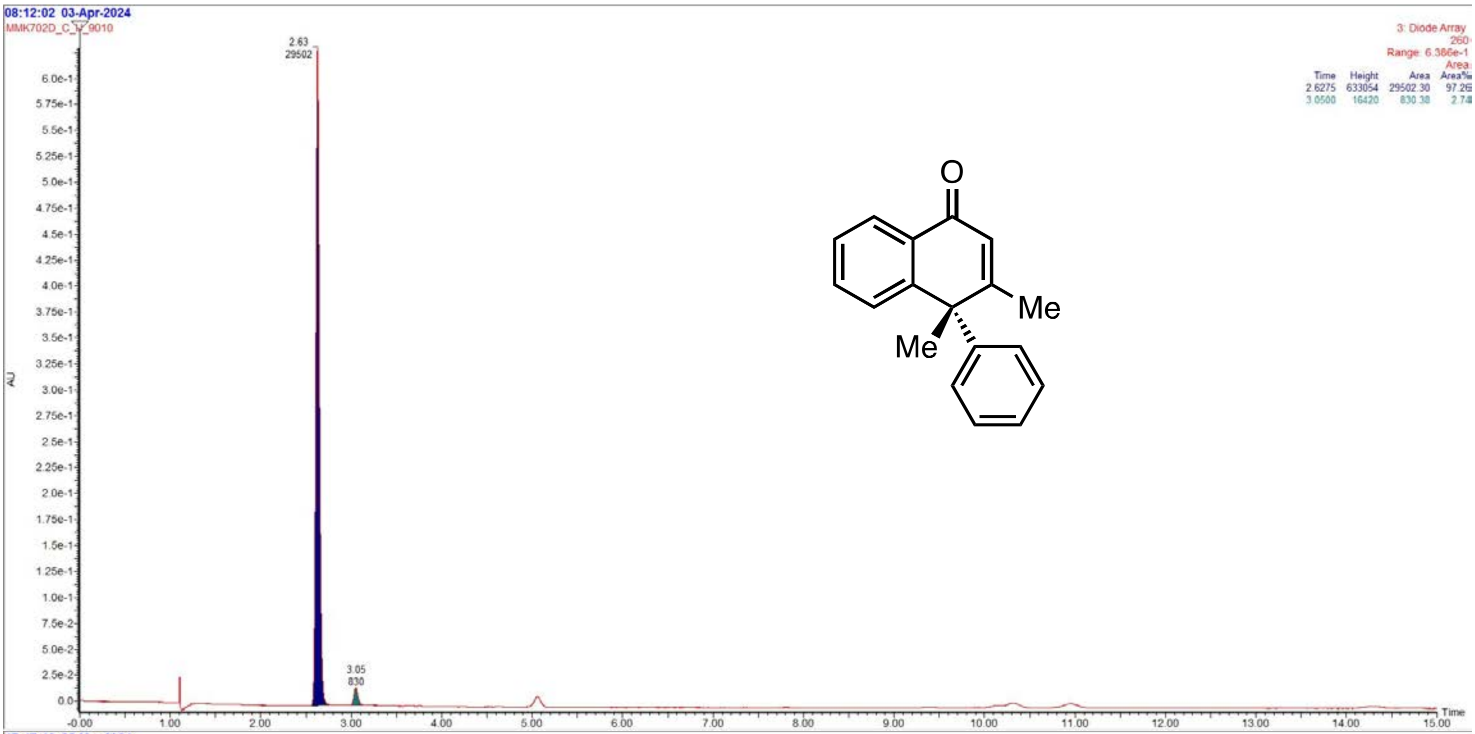

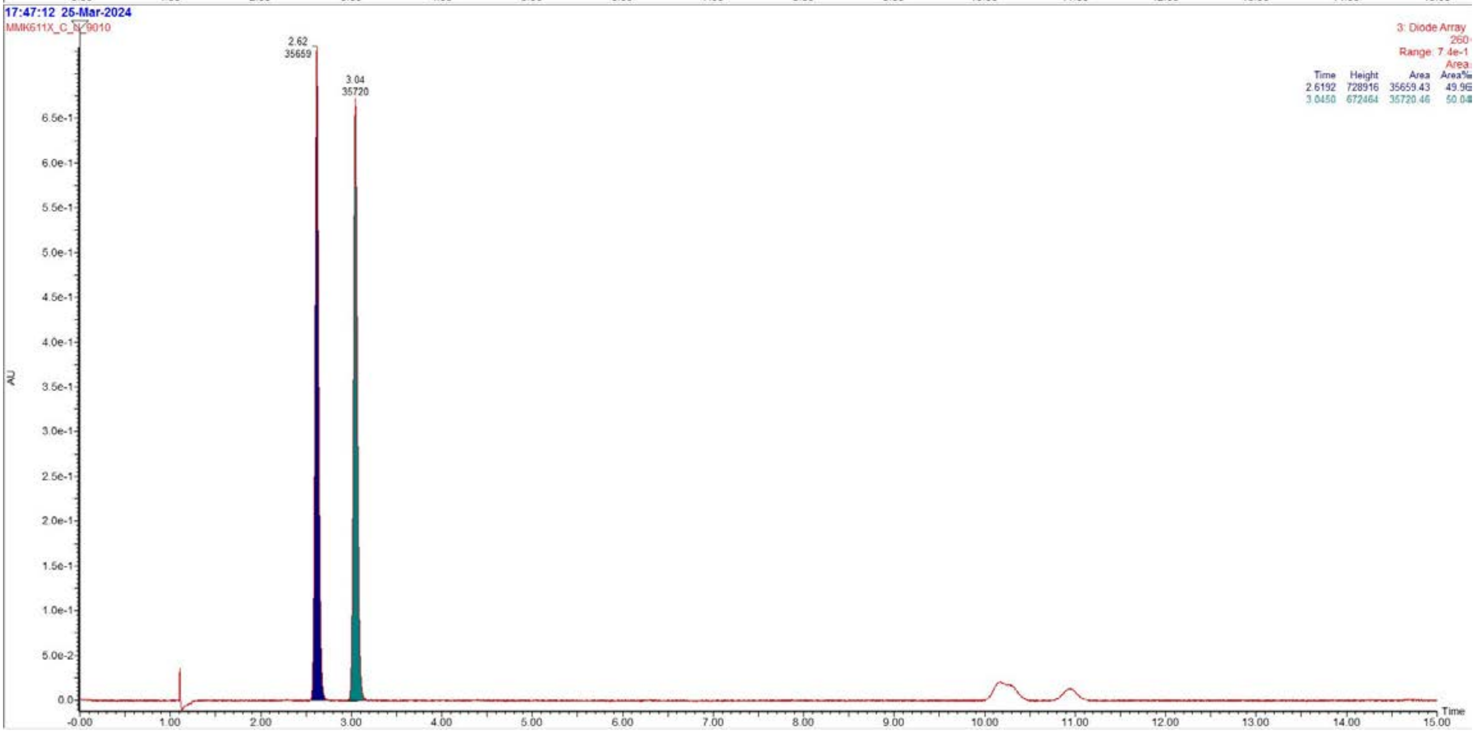

(*R*)-4-(4-methoxyphenyl)-4-Methylnaphthalen-1(4*H*)-one (2j)

Chiral SFC Analysis: CHIRALPAK IE (CO_2:MeOH, 90:10, 2.5 mL min^{-1}, 40 °C, 260 nm) indicated 98% *ee*, t_R = 7.89 (minor), 8.76 (major) minutes.

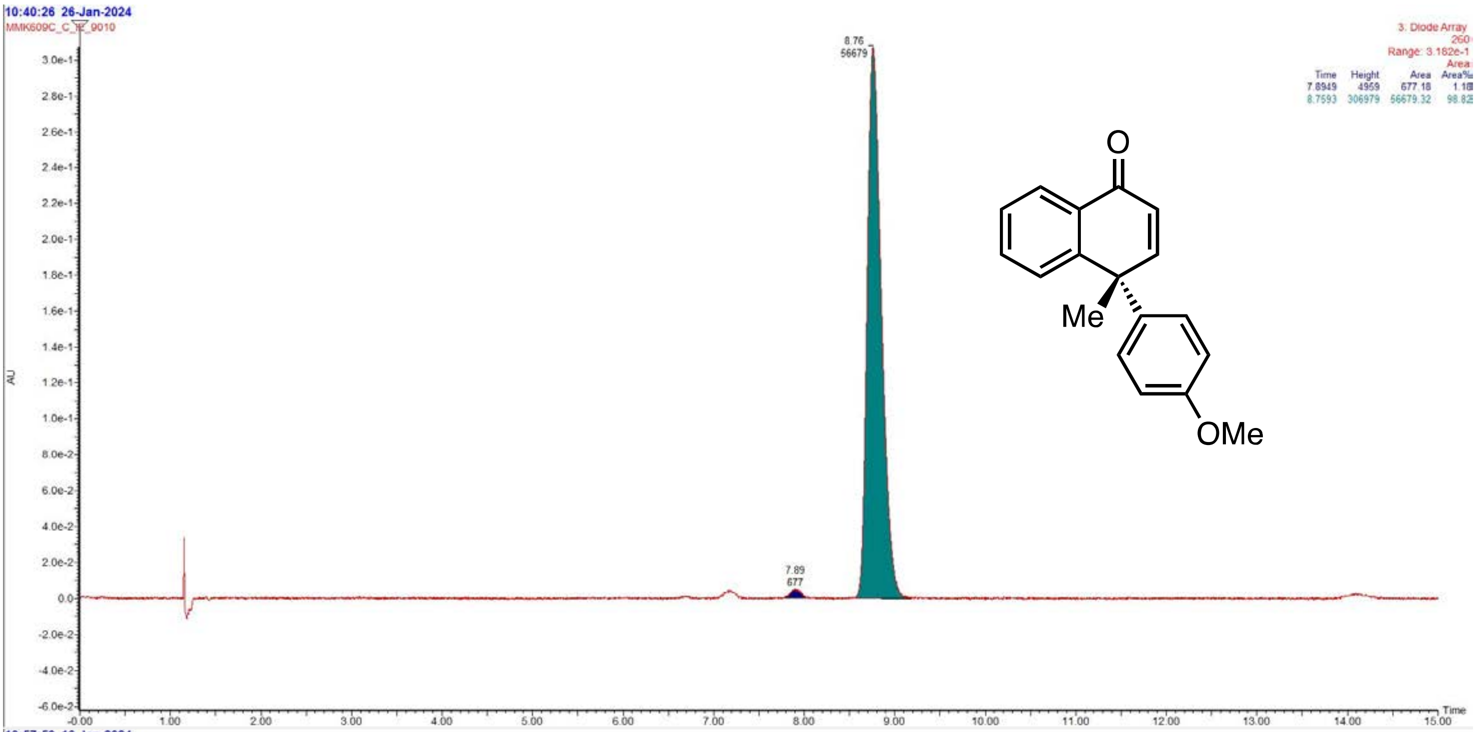

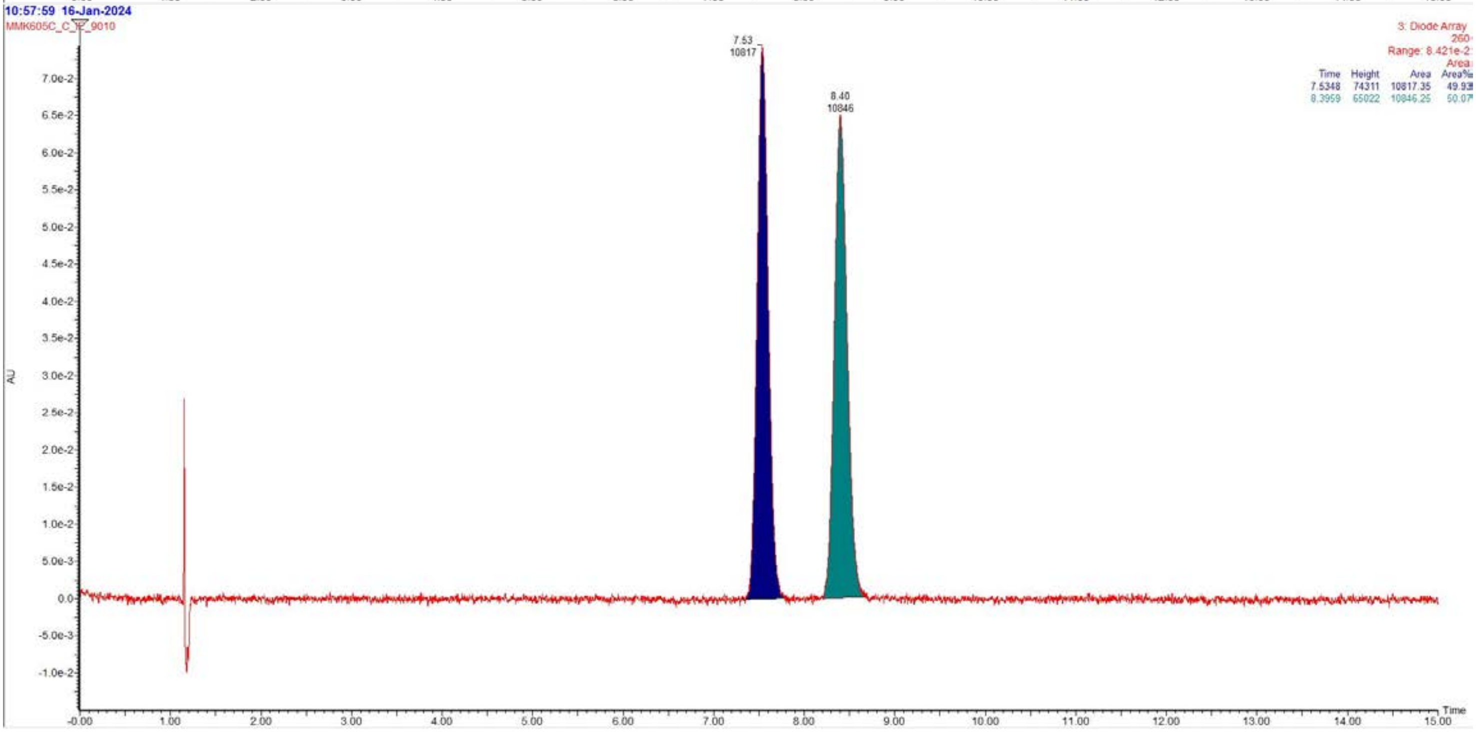

(*R*)-4-Methyl-4-(3,4,5-trimethoxyphenyl)naphthalen-1(4*H*)-one (2k)

Chiral SFC Analysis: CHIRALPAK IK (CO_2:MeOH, 80:20, 2.5 mL min^{-1}, 40 °C, 250 nm) indicated 89% *ee*, t_R = 4.69 (major), 5.52 (minor) minutes.

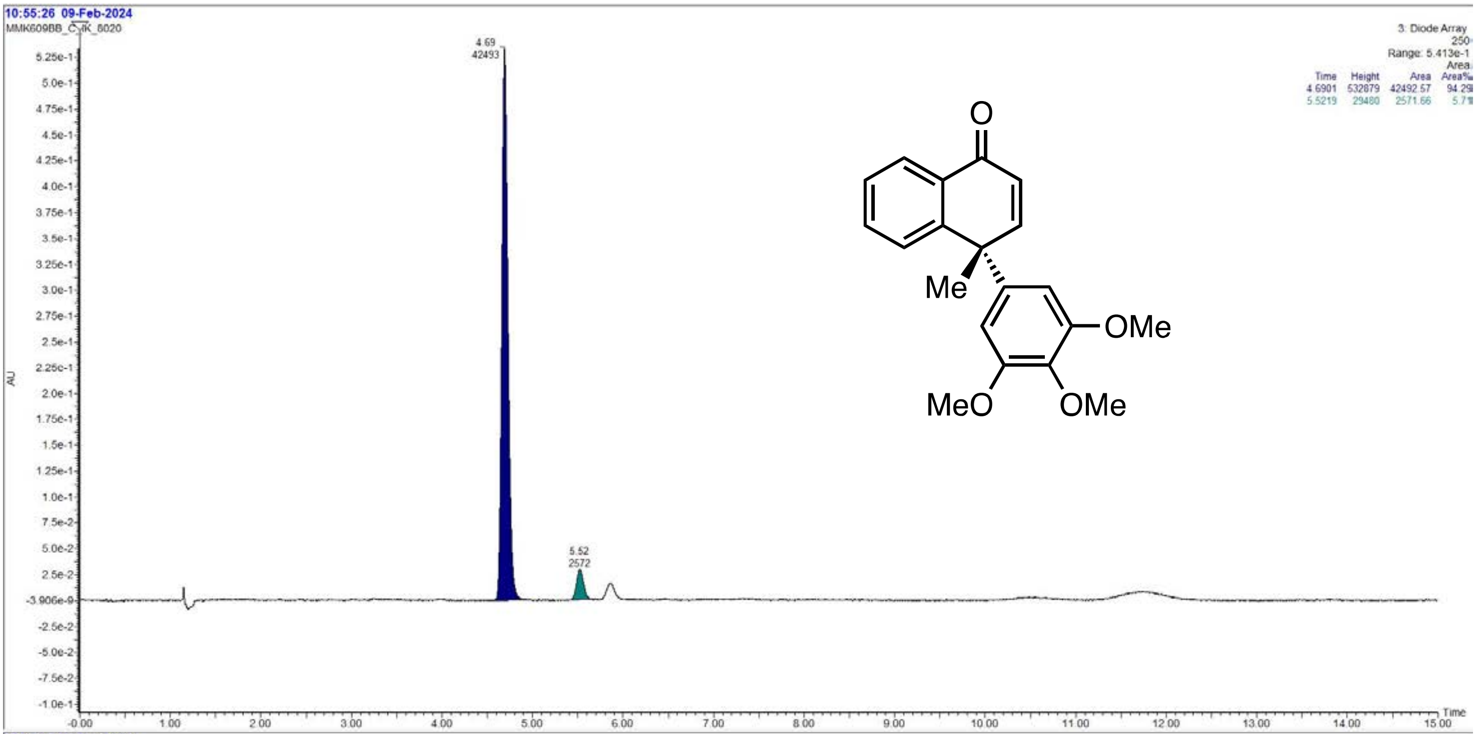

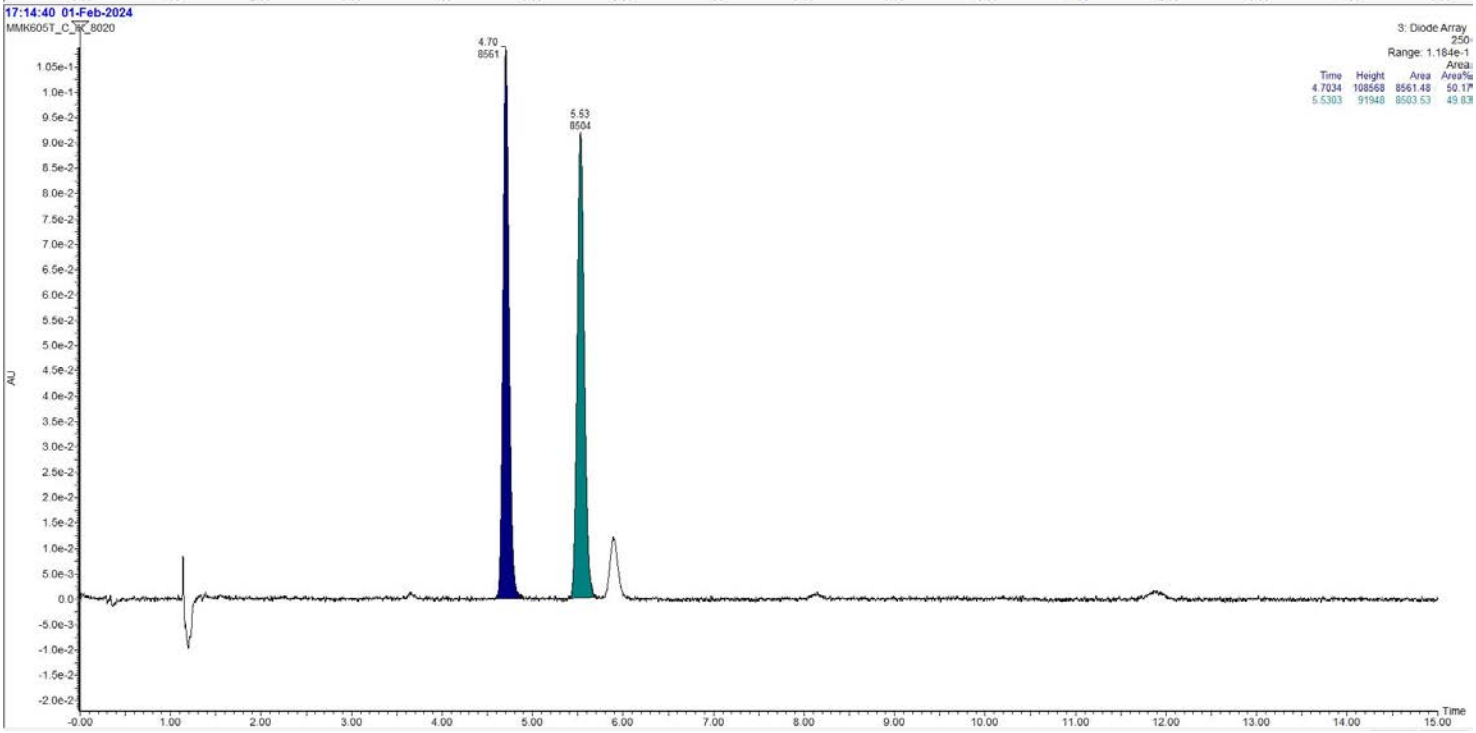

(*S*)-4-(2-methoxyphenyl)-4-Methylnaphthalen-1(4*H*)-one (2l)

Chiral SFC Analysis: CHIRALPAK IJ (CO_2:MeOH, 95:05, 2.5 mL min^{-1}, 40 °C, 250 nm) indicated 98% *ee*, t_R = 3.86 (major), 4.17 (minor) minutes.

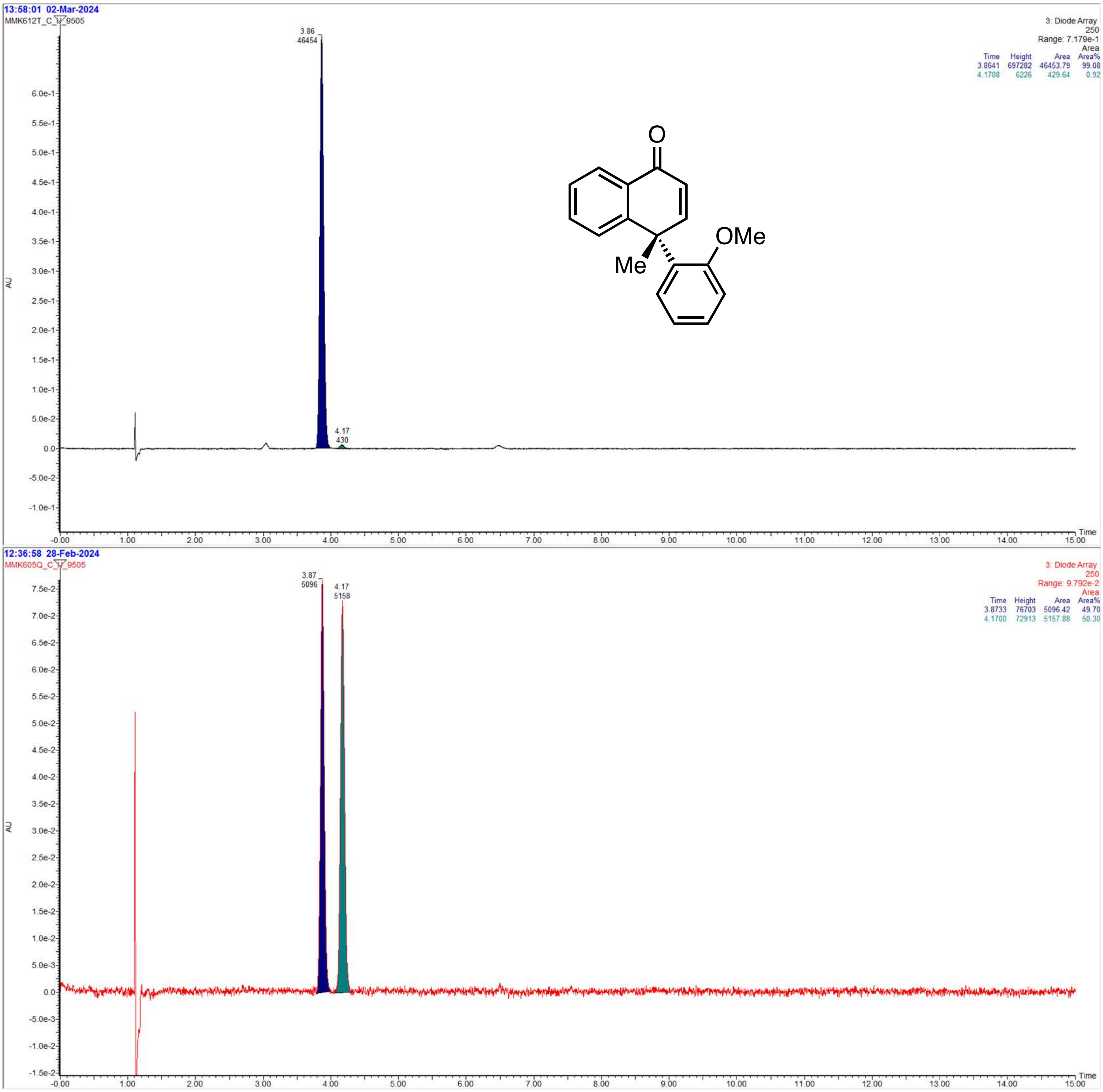

(*R*)-4-(3-fluoro-5-methoxyphenyl)-4-Methylnaphthalen-1(4*H*)-one (2m)

Chiral SFC Analysis: CHIRALPAK IK (CO_2:MeOH, 95:05, 2.5 mL min^{-1}, 40 °C, 250 nm) indicated 91% *ee*, t_R = 6.94 (minor), 7.33 (major) minutes.

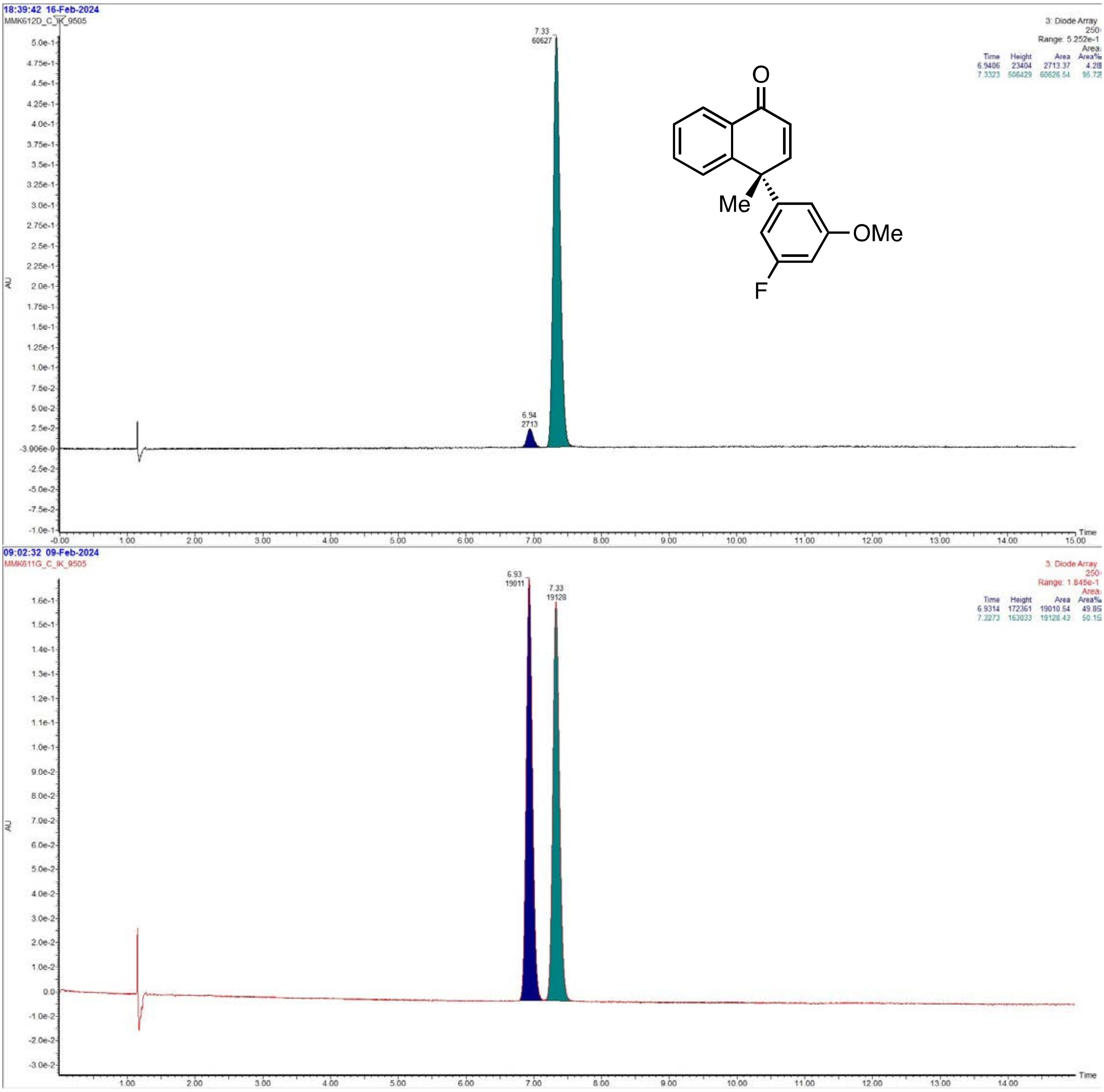

(*R*)-4-(3,4-bis(methoxymethoxy)phenyl)-4-Methylnaphthalen-1(4*H*)-one (2n)

Chiral SFC Analysis: CHIRALPAK IF (CO_2 (A):0.1% NH_3 in MeOH (B), isocratic 5% B, 2.5 mL min^{-1}, 40 °C, 254 nm) indicated 95% *ee*, t_R = 2.04 (minor), 2.25 (major) minutes.

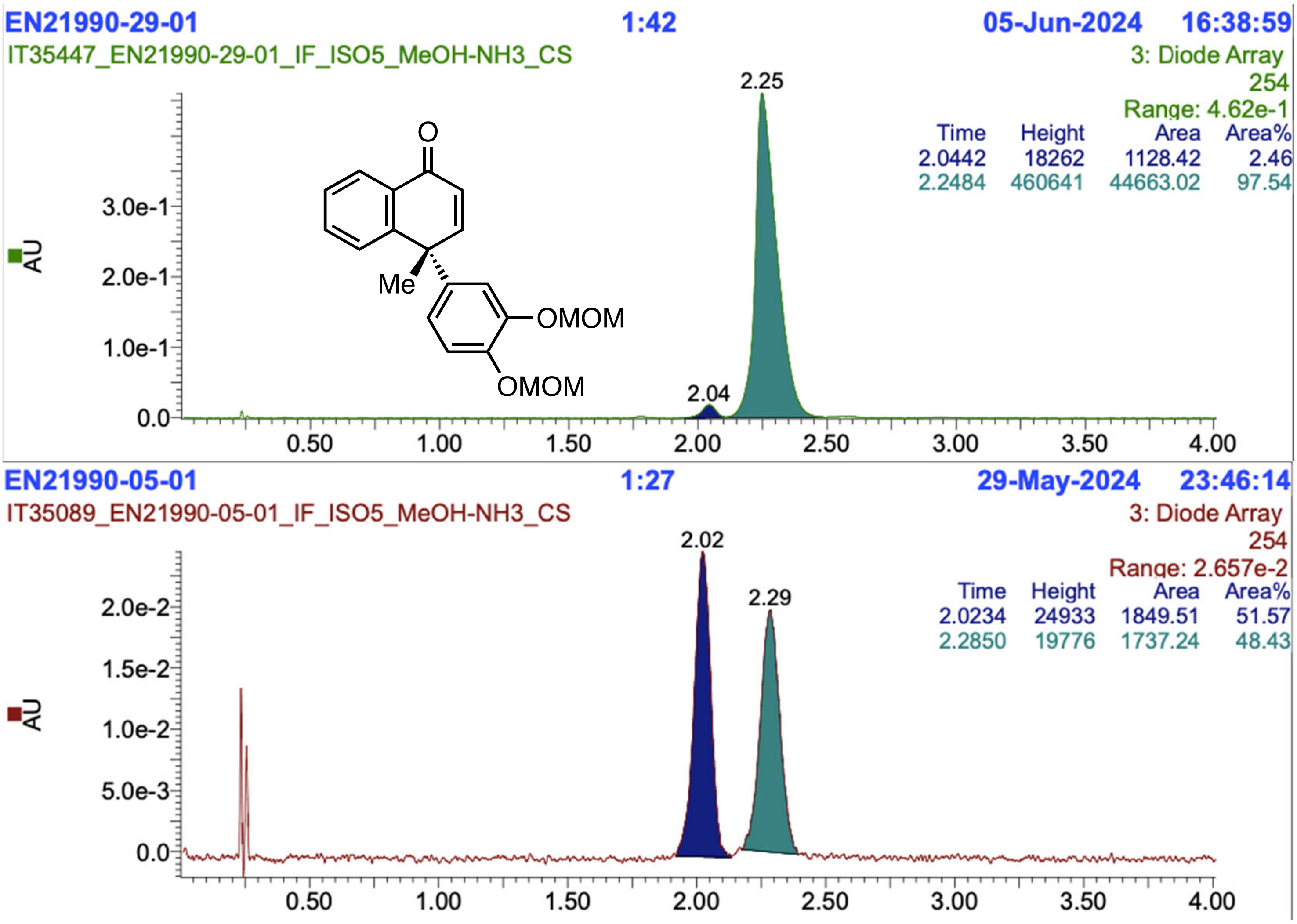

(*R*)-4-(4-(dimethylamino)phenyl)-4-Methylnaphthalen-1(4*H*)-one (2o)

Chiral SFC Analysis: CHIRALPAK AD (CO_2 (A):0.1% NH_3 in MeOH (B), 5% B – 50% B over two minutes, then isocratic 50% B for 2 minutes, 2.5 mL min^{-1}, 40 °C, 254 nm) indicated 96% *ee*, t_R = 1.20 (minor), 1.60 (major) minutes.

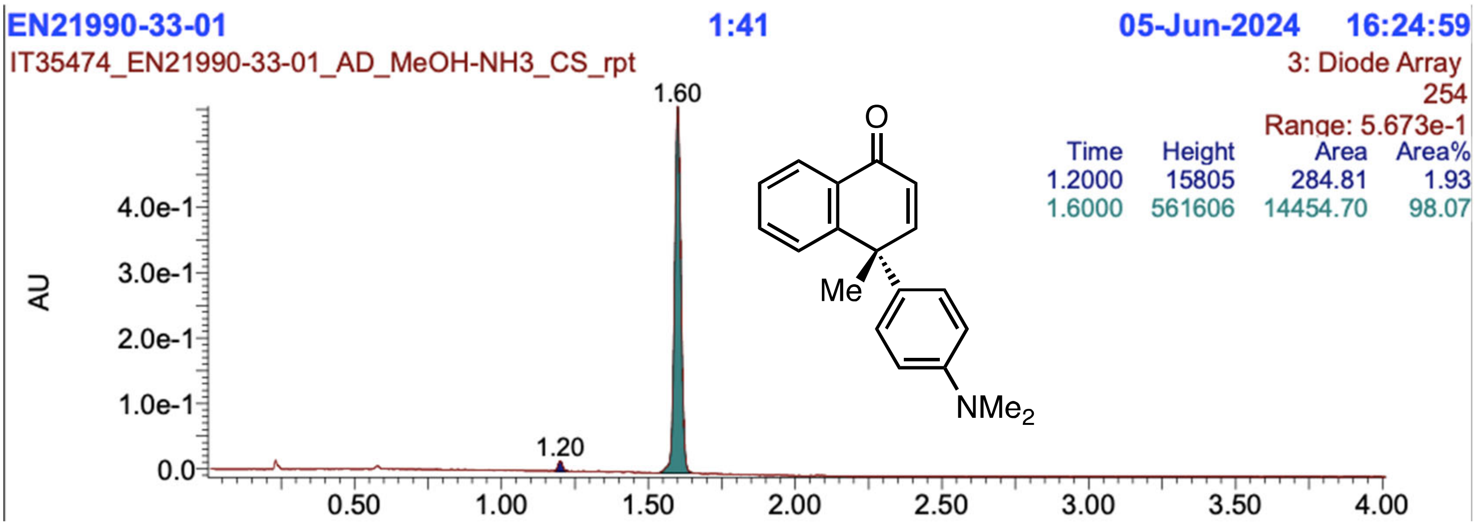

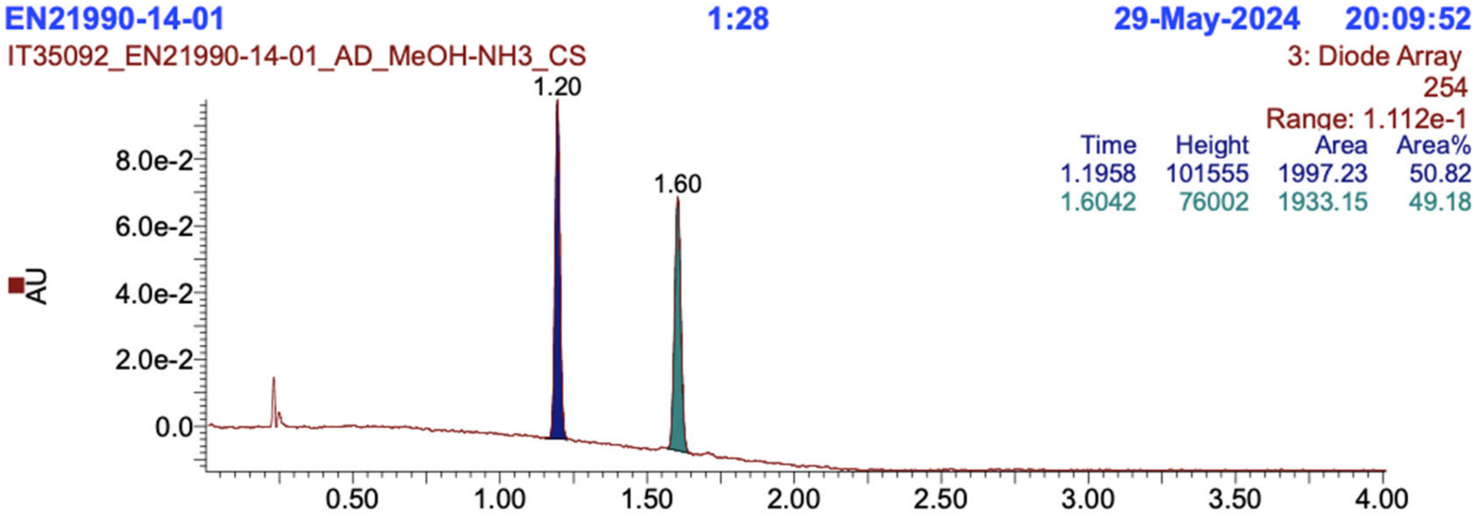

(*R*)-4-(3-(dibenzylamino)phenyl)-4-Methylnaphthalen-1(4*H*)-one (2p)

Chiral SFC Analysis: CHIRALPAK AD (CO_2 (A):0.1% DEA in IPA (B), 5% B – 50% B over two minutes, then isocratic 50% B for 2 minutes, 2.5 mL min^{-1}, 40 °C, 270 nm) indicated 96% *ee*, t_R = 1.53 (minor), 1.66 (major) minutes.

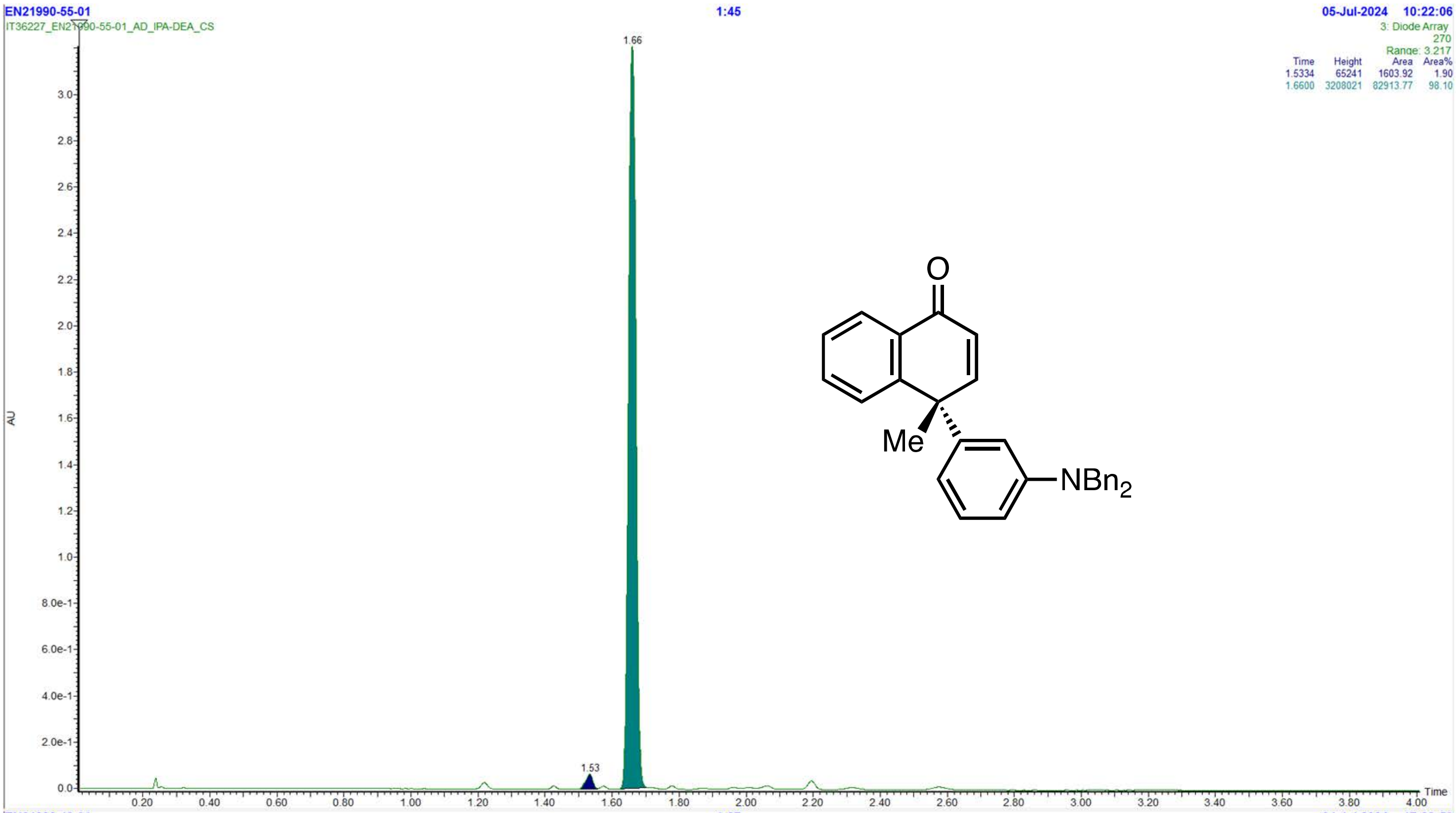

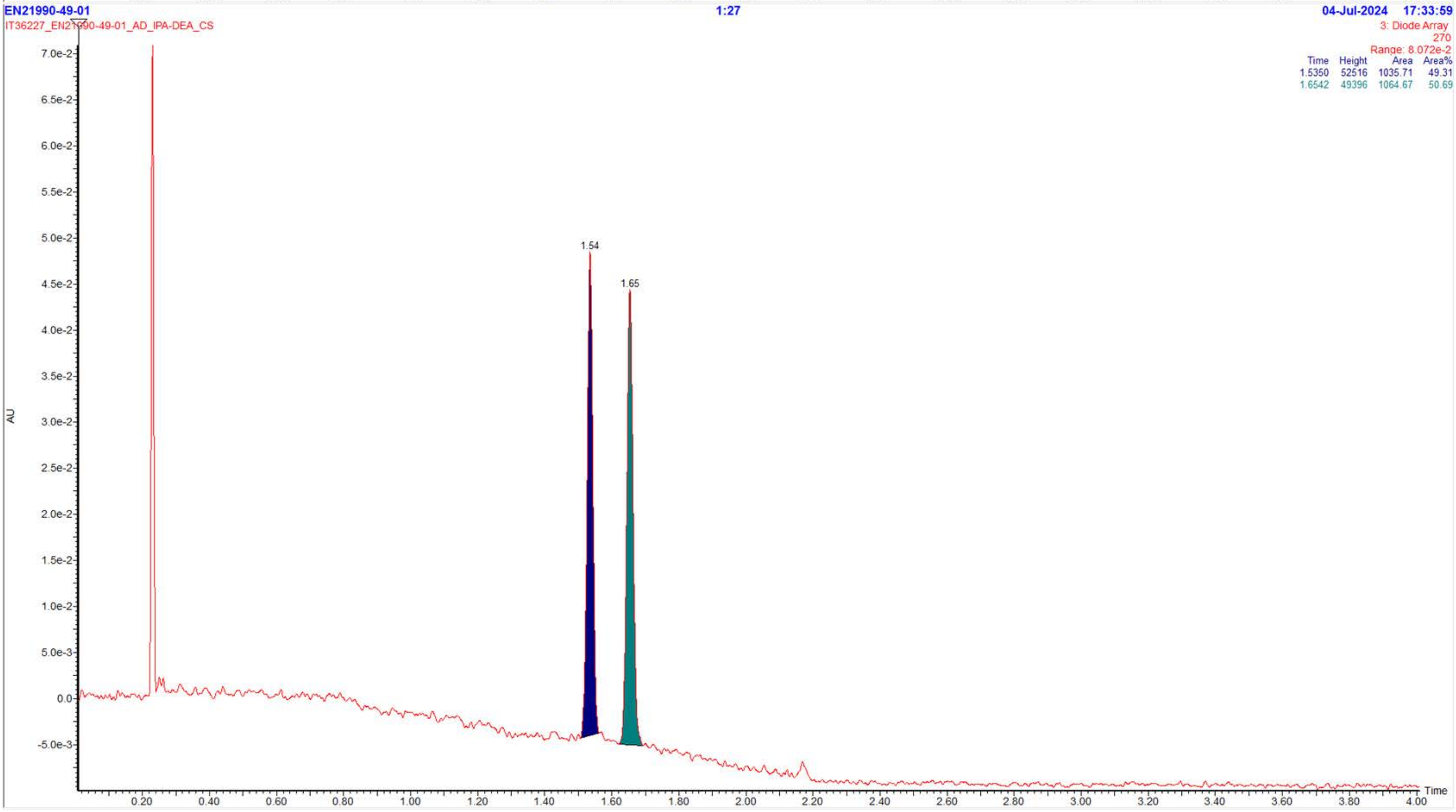

(*R*)-4-(3,5-dimethylphenyl)-4-Methylnaphthalen-1(4*H*)-one (2q)

Chiral SFC Analysis: CHIRALPAK IK (CO_2:MeOH, 95:05, 2.5 mL min^{-1}, 40 °C, 250 nm) indicated 98% *ee*, t_R = 6.82 (major), 7.34 (minor) minutes.

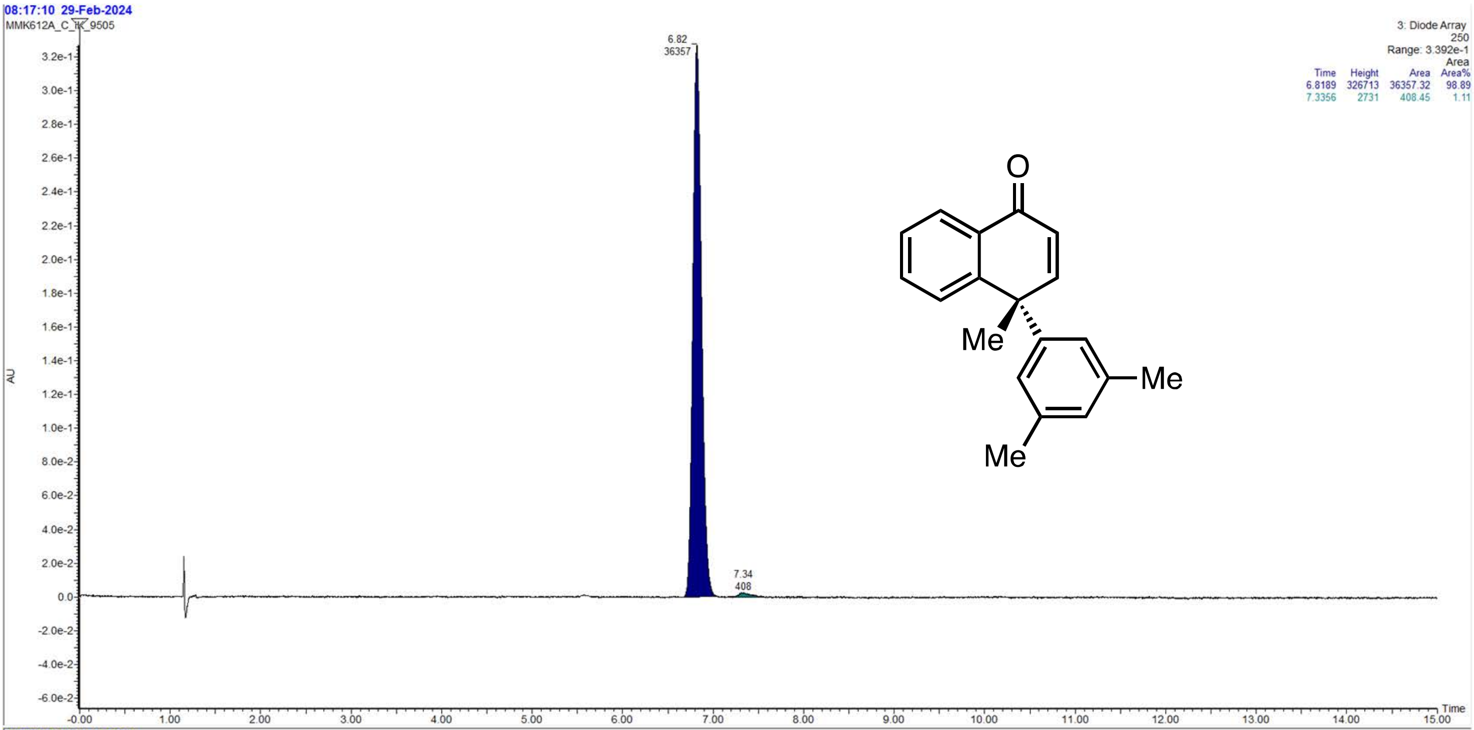

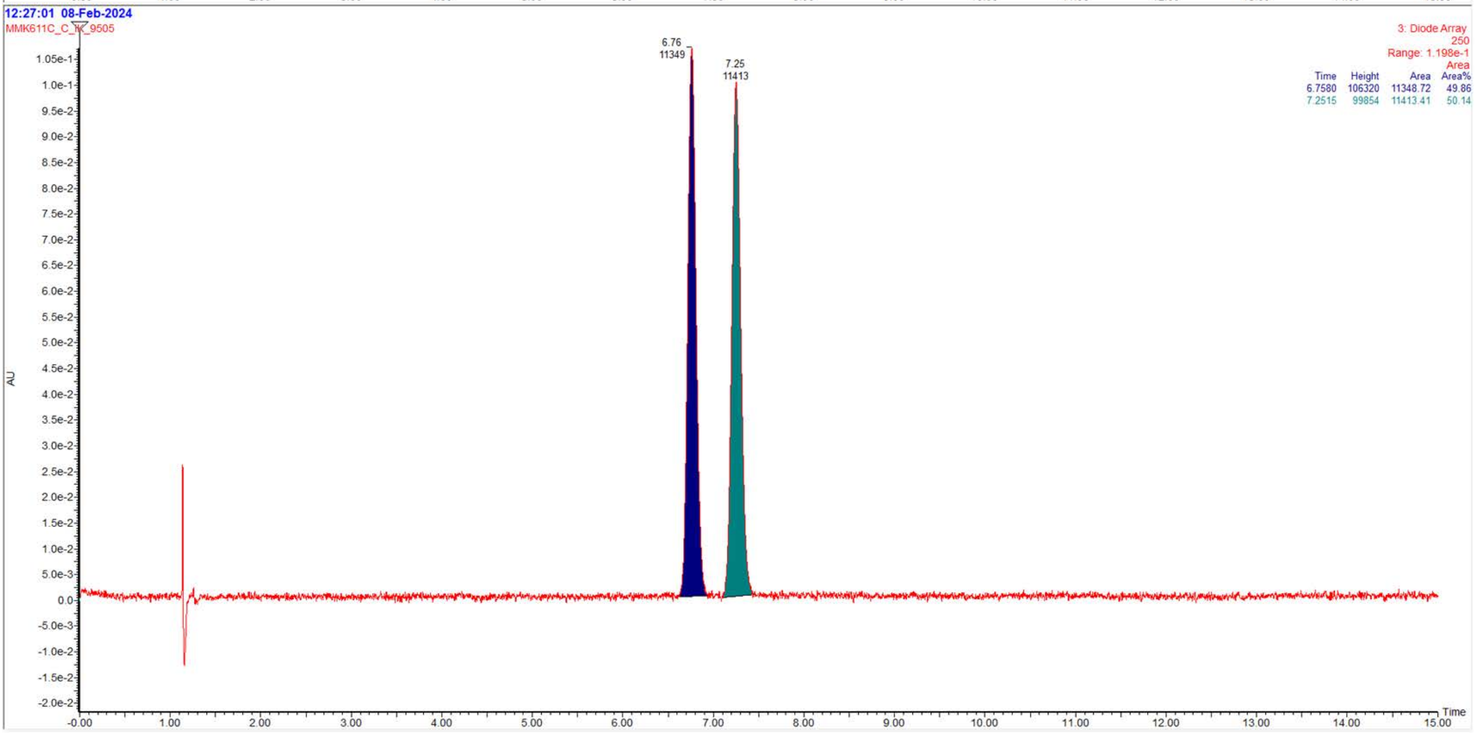

(*R*)-4-(3-chlorophenyl)-4-Methylnaphthalen-1(4*H*)-one (2r)

Chiral SFC Analysis: CHIRALPAK IE (CO_2:MeOH, 90:10, 2.5 mL min^{-1}, 40 °C, 250 nm) indicated 95% *ee*, t_R = 5.72 (minor), 6.15 (major) minutes.

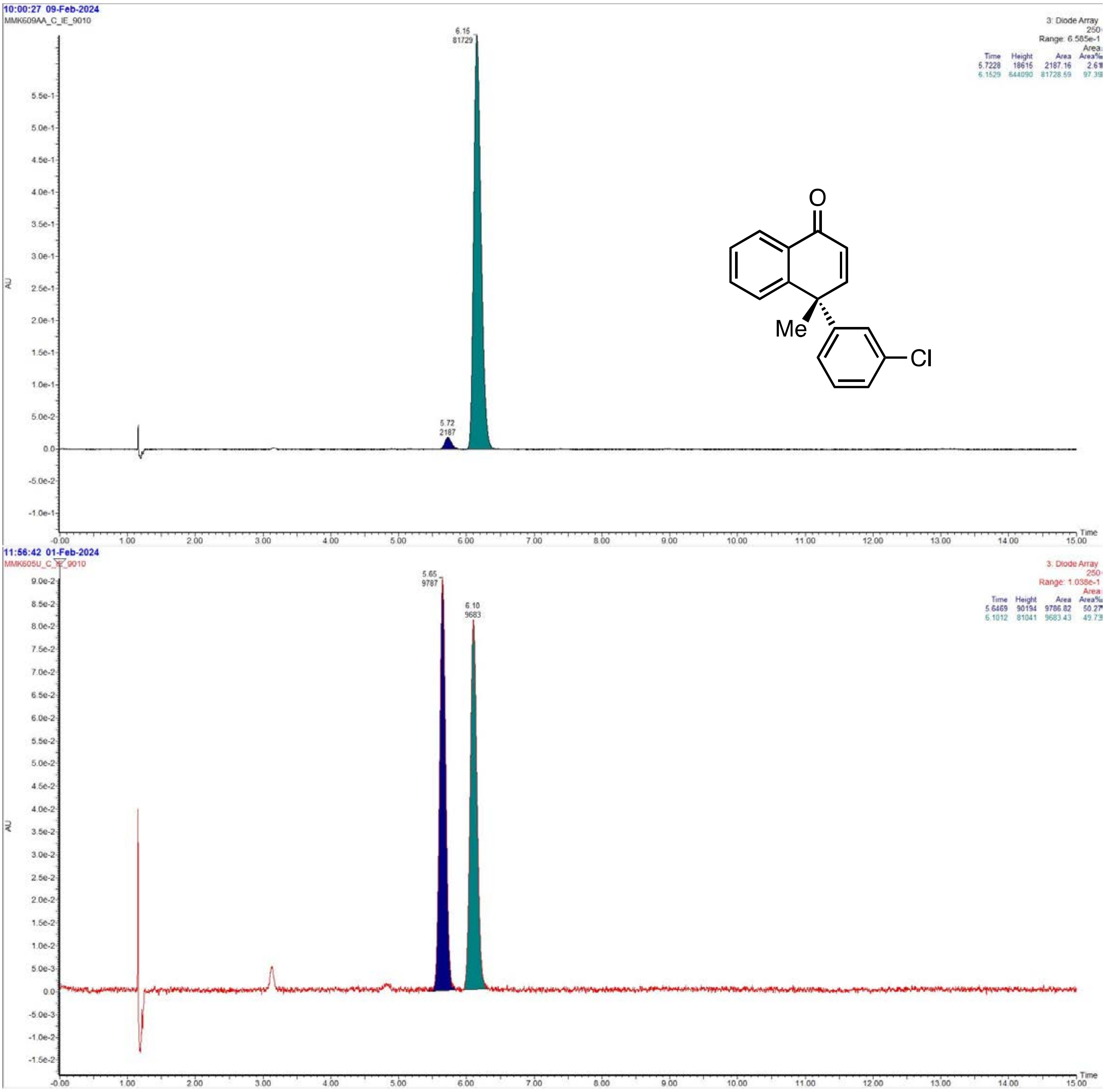

(*R*)-4-(3-chloro-5-fluorophenyl)-4-Methylnaphthalen-1(4*H*)-one (2s)

Chiral SFC Analysis: CHIRALPAK IG (CO_2:MeOH, 95:05, 2.5 mL min^{-1}, 40 °C, 250 nm) indicated 89% *ee*, t_R = 6.11 (minor), 7.20 (major) minutes.

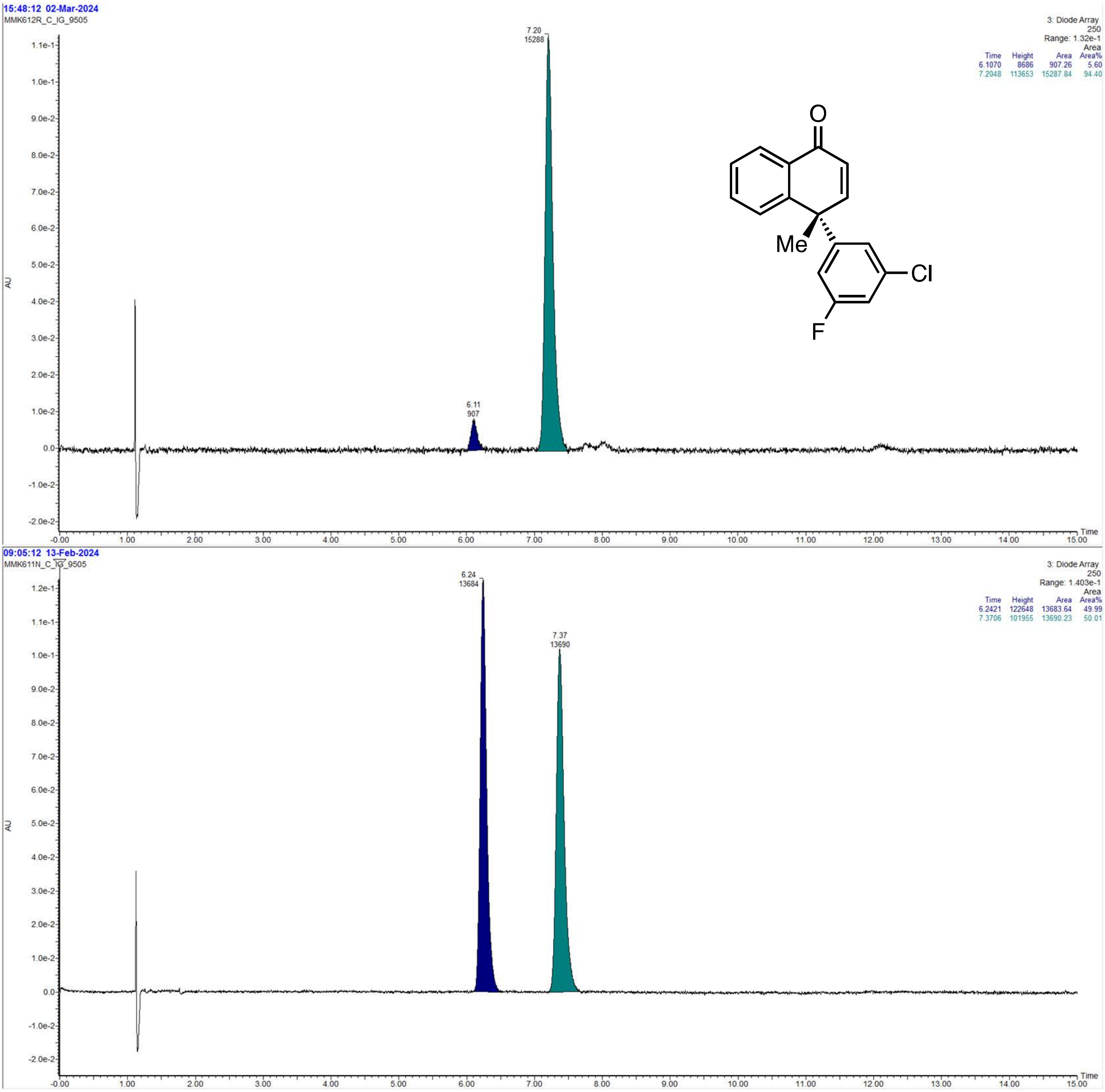

Ethyl (*R*)-4-(1-methyl-4-oxo-1,4-dihydronaphthalen-1-yl)benzoate (2t)

Chiral SFC Analysis: CHIRALPAK IE (CO_2:MeOH, 80:20, 2.5 mL min^{-1}, 40 °C, 250 nm) indicated 96% *ee*, t_R = 6.15 (minor), 7.14 (major) minutes.

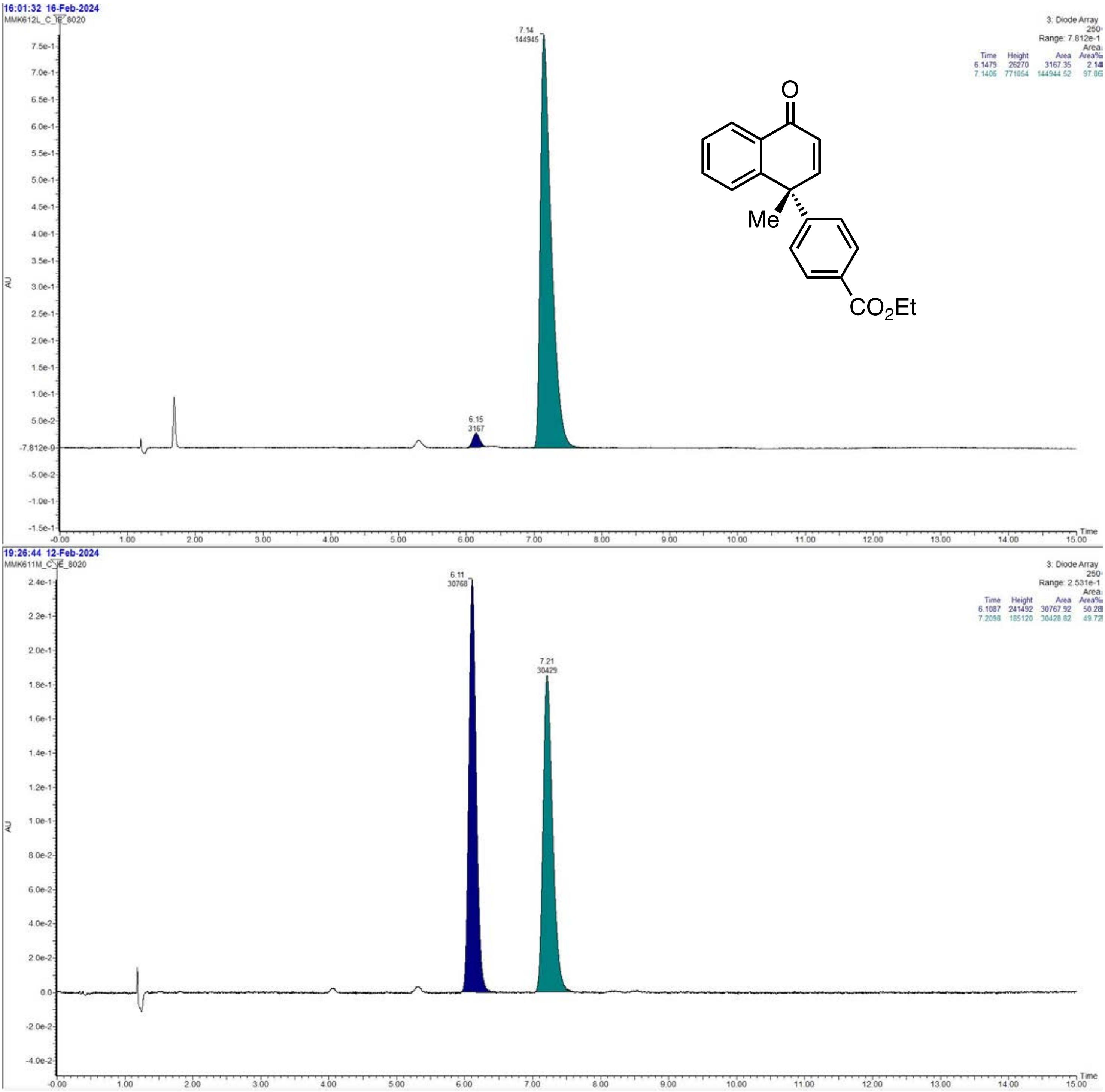

(*R*)-4-(1-methyl-4-oxo-1,4-dihydronaphthalen-1-yl)Benzonitrile (2u)

Chiral SFC Analysis: CHIRALPAK IE (CO_2:MeOH, 80:20, 2.5 mL min^{-1}, 40 °C, 250 nm) indicated 90% *ee*, t_R = 6.08 (minor), 6.52 (major) minutes.

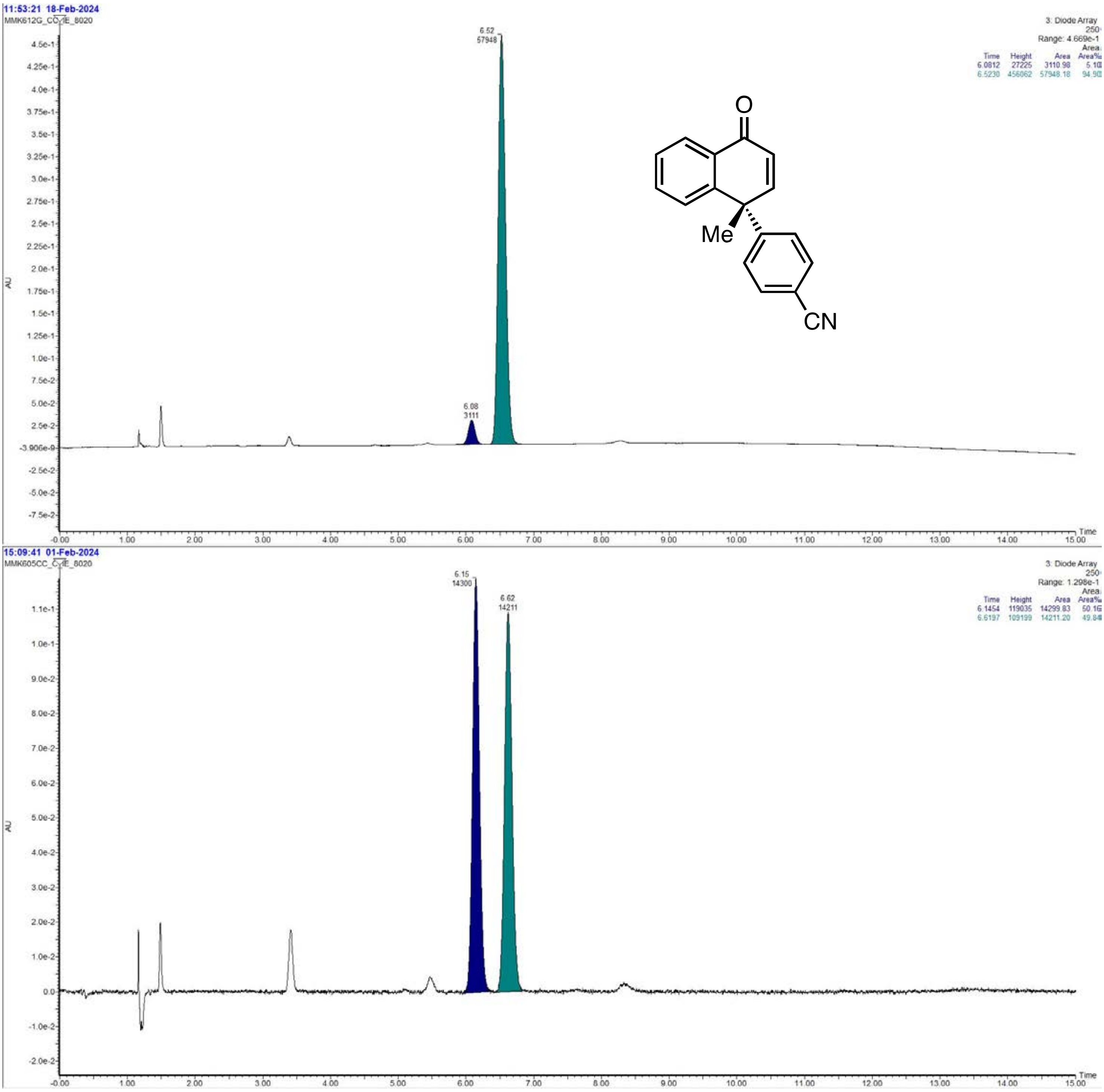

(*R*)-4-Methyl-4-(4-nitrophenyl)naphthalen-1(4*H*)-one (2v)

Chiral SFC Analysis: CHIRALPAK IE (CO_2:MeOH, 80:20, 2.5 mL min^{-1}, 40 °C, 260 nm) indicated 81% *ee*, t_R = 7.02 (minor), 7.58 (major) minutes.

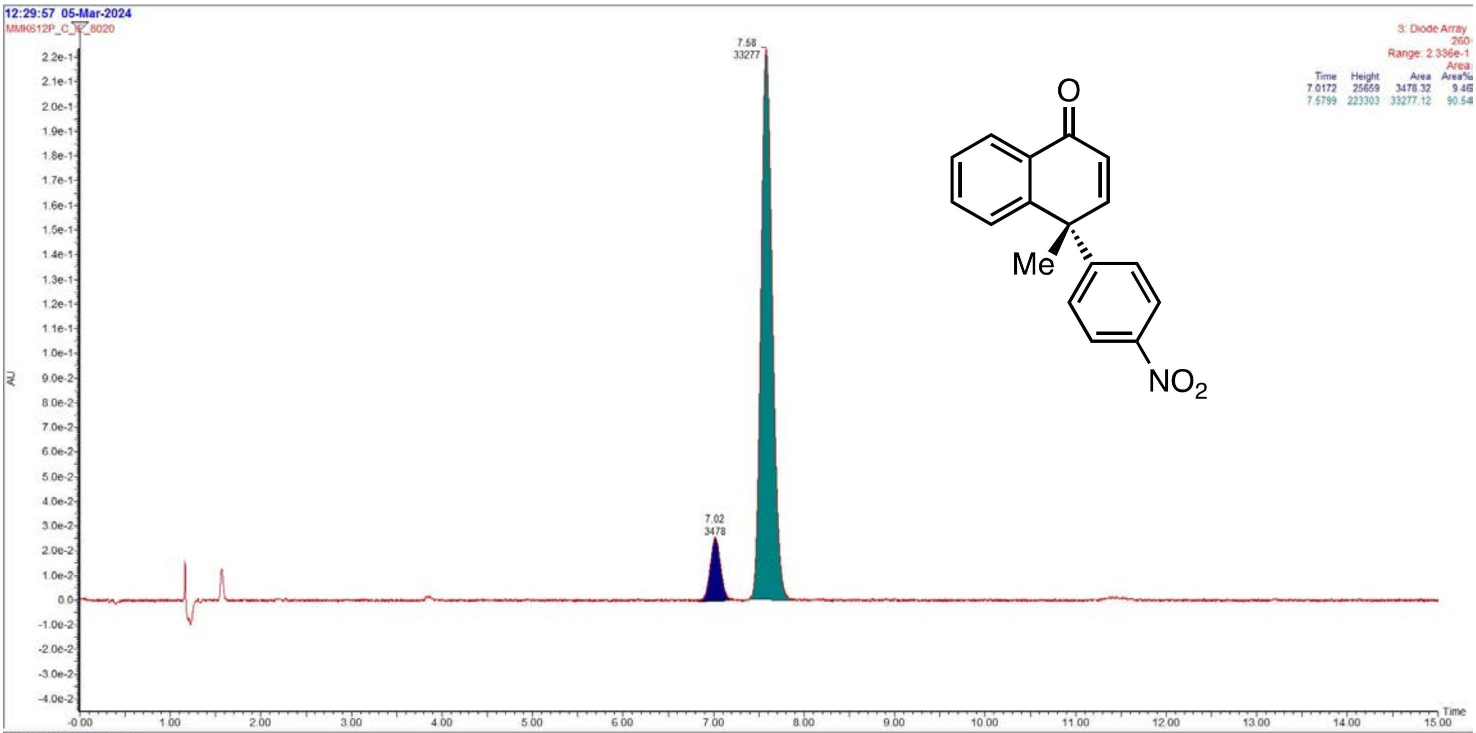

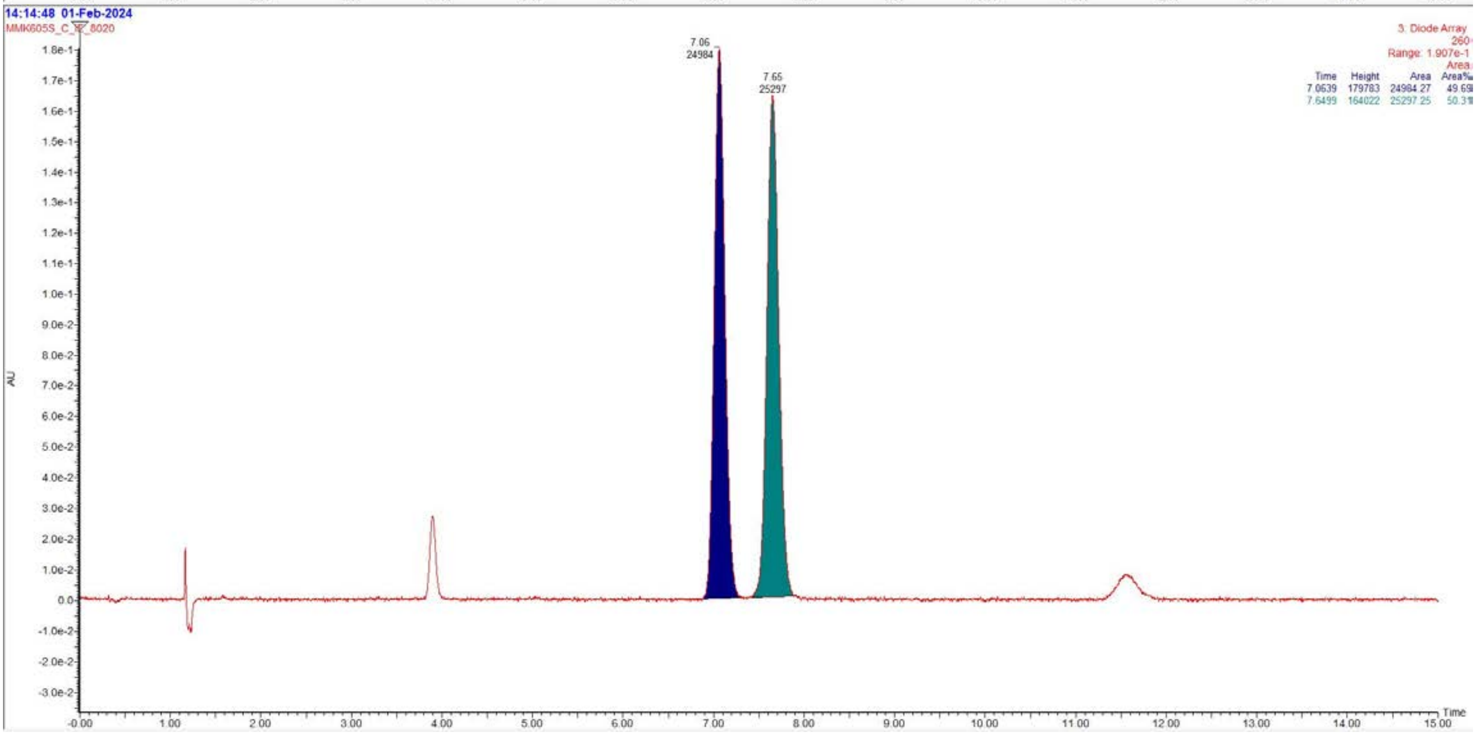

(*R*)-4-Methyl-4-(4-(trifluoromethyl)phenyl)naphthalen-1(4*H*)-one (2w)

Chiral SFC Analysis: CHIRALPAK IE (CO_2:MeOH, 98:02, 2.5 mL min^{-1}, 40 °C, 250 nm) indicated 95% *ee*, t_R = 11.54 (major), 12.70 (minor) minutes.

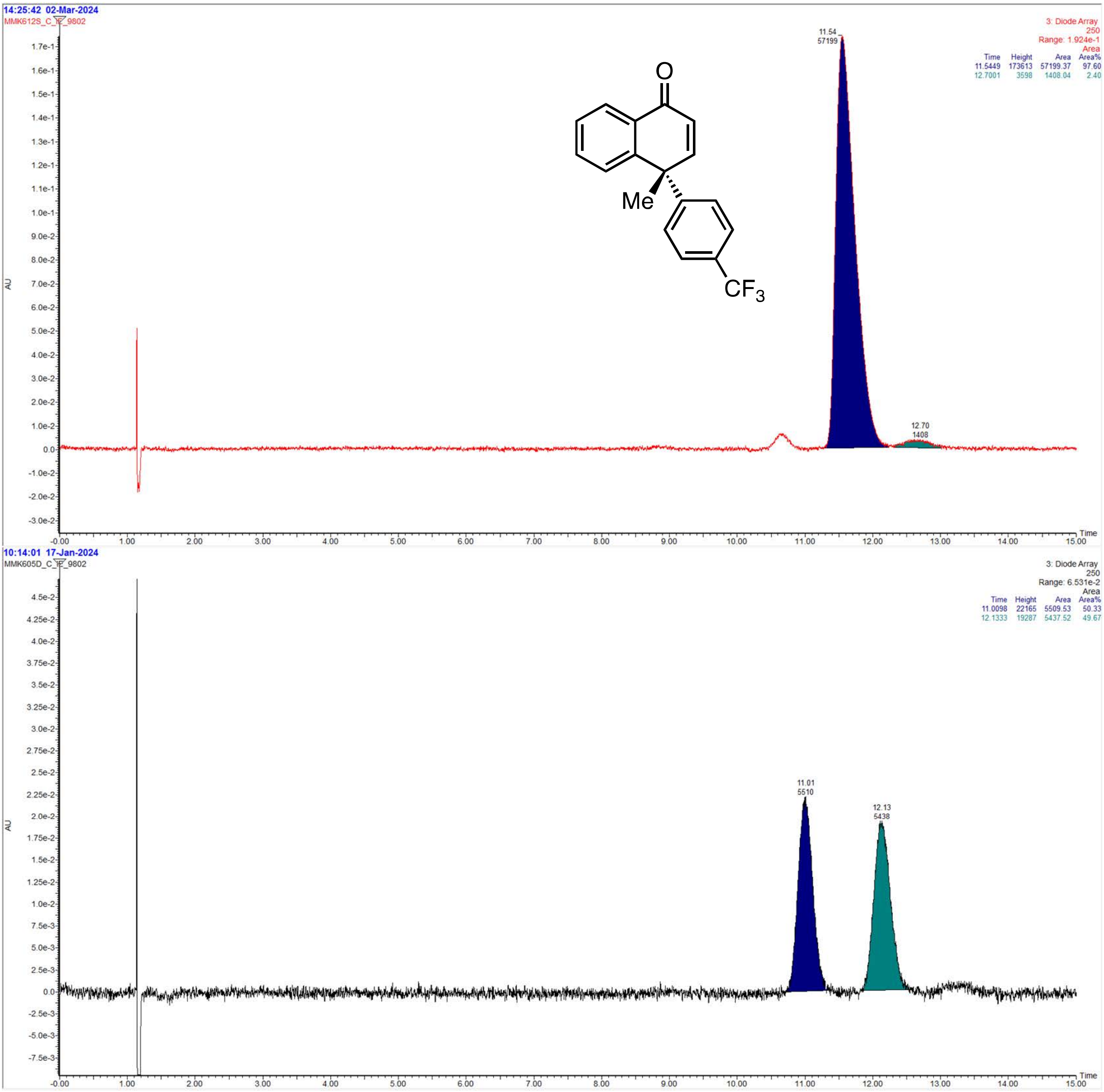

(*R*)-4-([1,1'-biphenyl]-4-yl)-4-Methylnaphthalen-1(4*H*)-one (2x)

Chiral SFC Analysis: CHIRALPAK IK (CO_2:MeOH, 80:20, 2.5 mL min^{-1}, 40 °C, 250 nm) indicated 97% *ee*, t_R = 7.36 (minor), 8.68 (major) minutes.

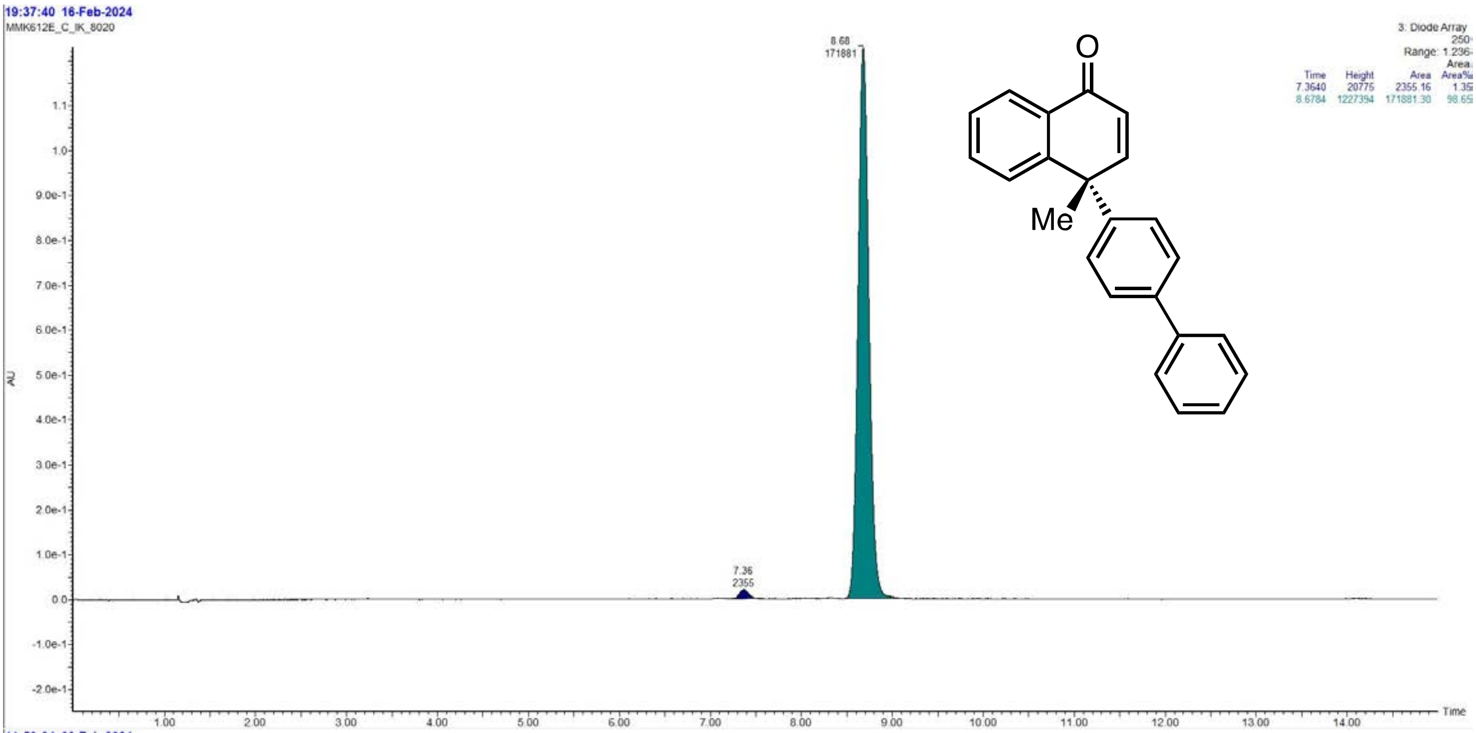

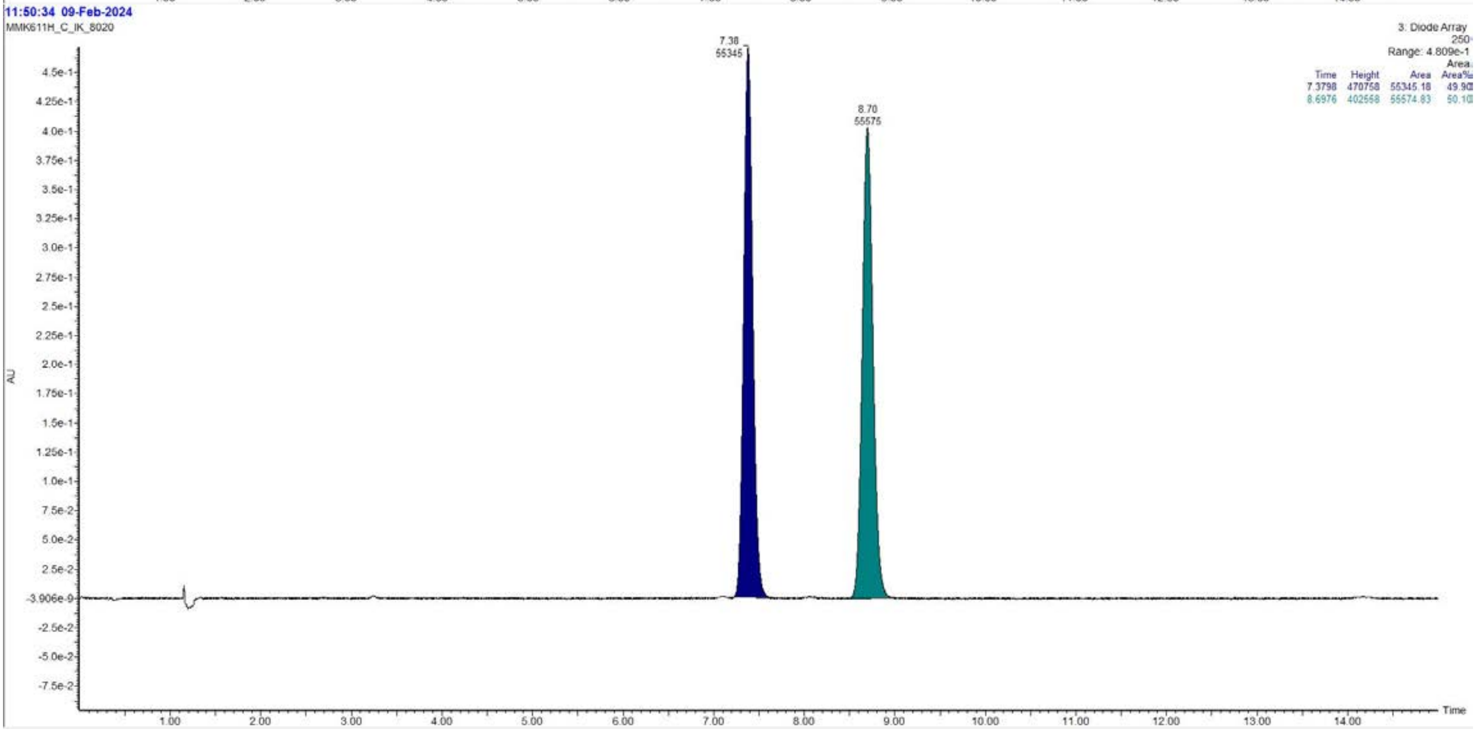

(*R*)-1-Methyl-[1,2'-binaphthalen]-4(1*H*)-one (2y)

Chiral SFC Analysis: CHIRALPAK IE (CO_2:MeOH, 90:10, 2.5 mL min^{-1}, 40 °C, 250 nm) indicated 97% *ee*, t_R = 12.28 (minor), 14.33 (major) minutes.

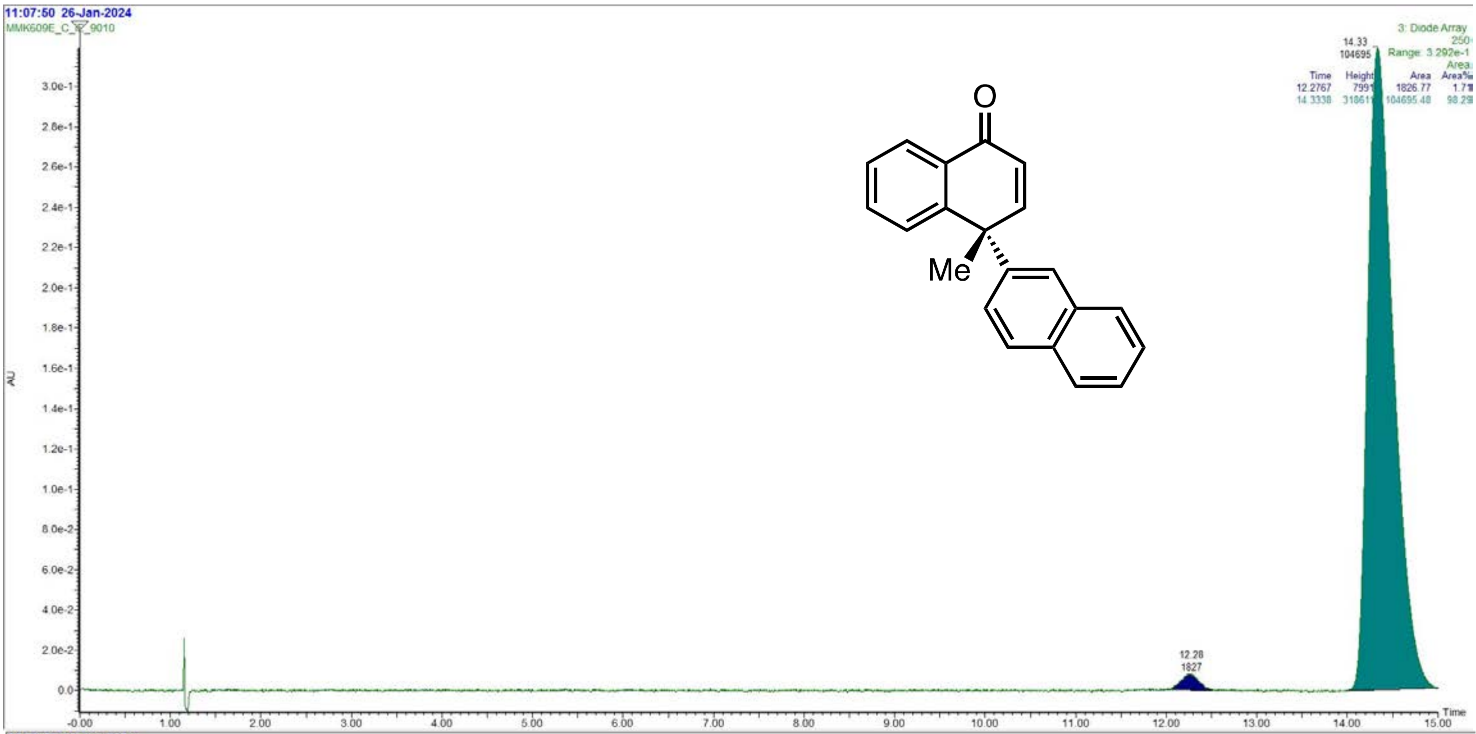

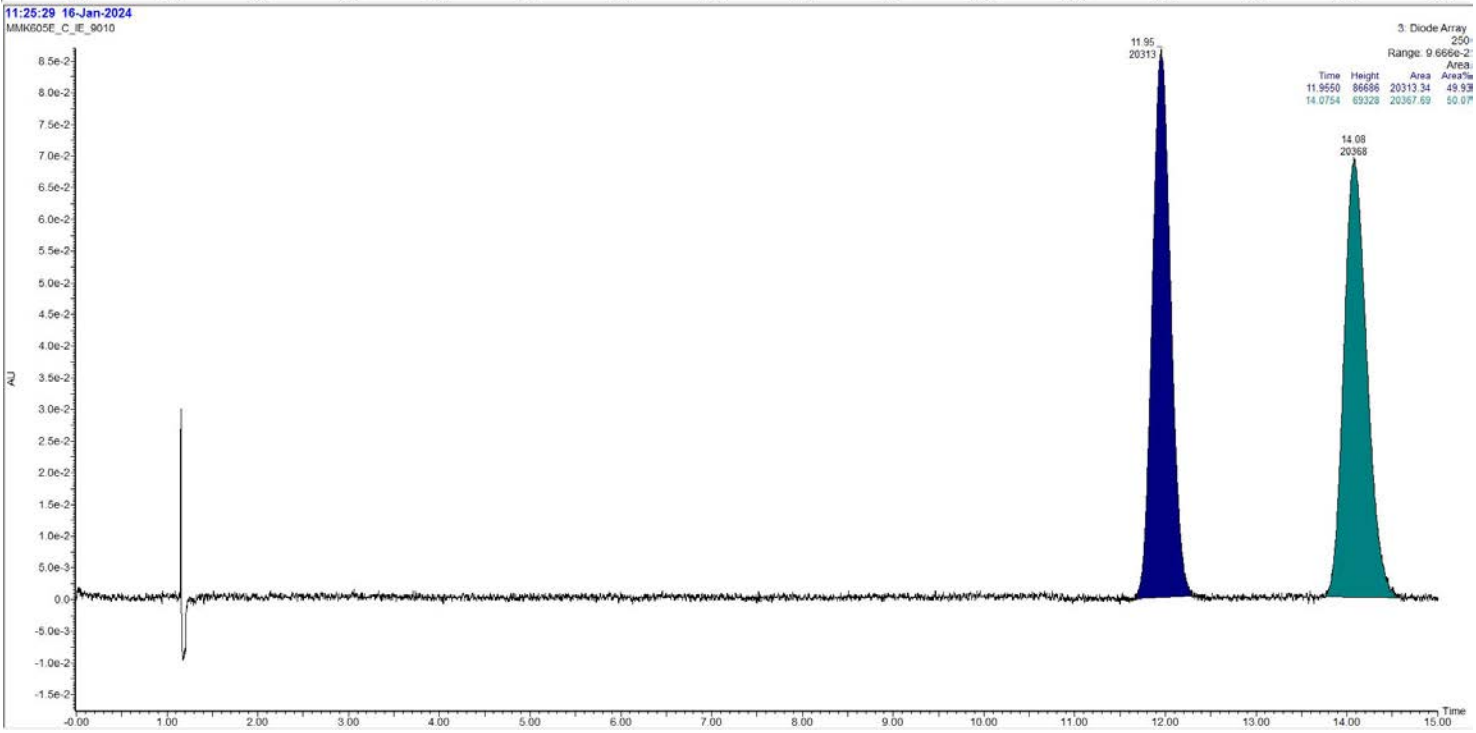

(R)-4-(3-(*tert*-butyl)phenyl)-4-Methylnaphthalen-1(4H)-one (2z)

Chiral SFC Analysis: CHIRALPAK IC (CO_2:MeOH, 95:05, 2.5 mL min^{-1}, 40 °C, 250 nm) indicated 98% *ee*, t_R = 6.44 (major), 6.78 (minor) minutes.

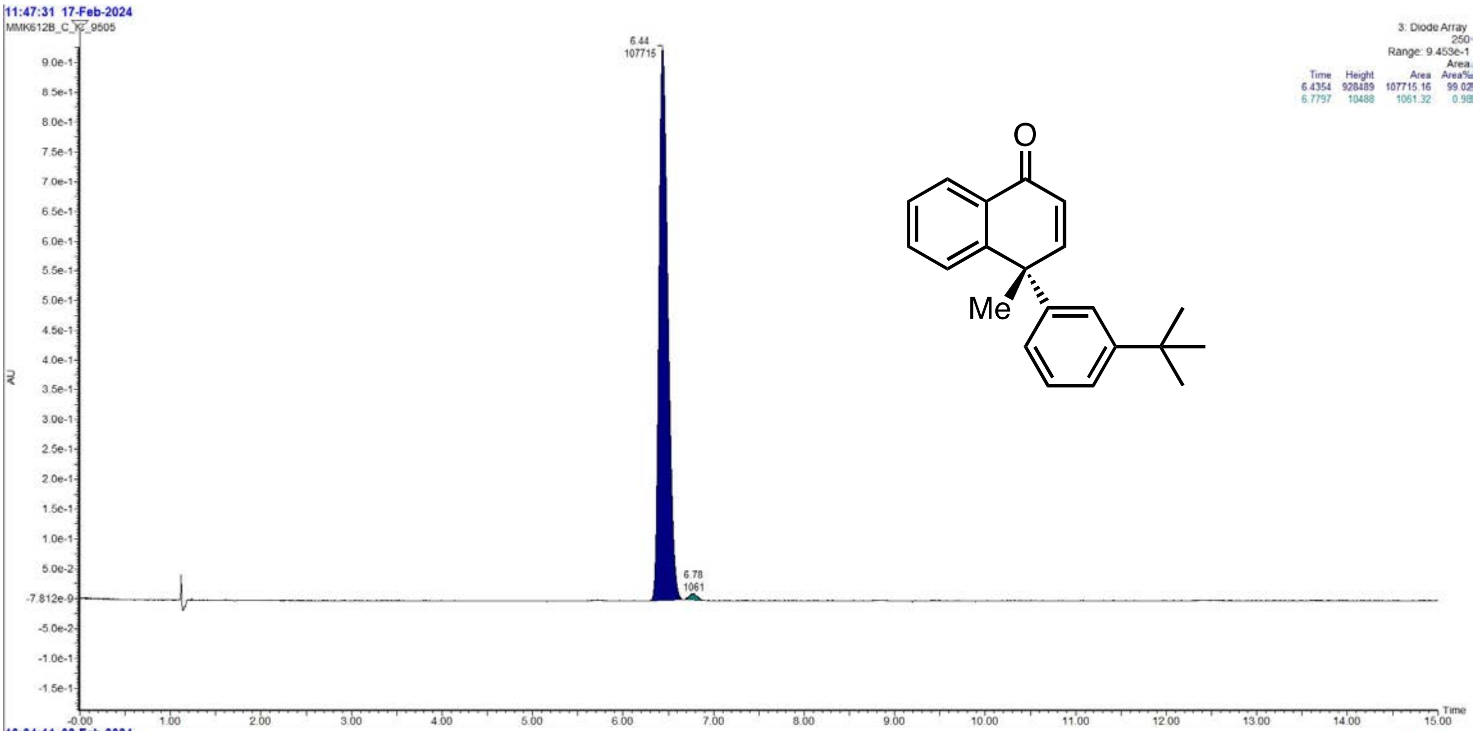

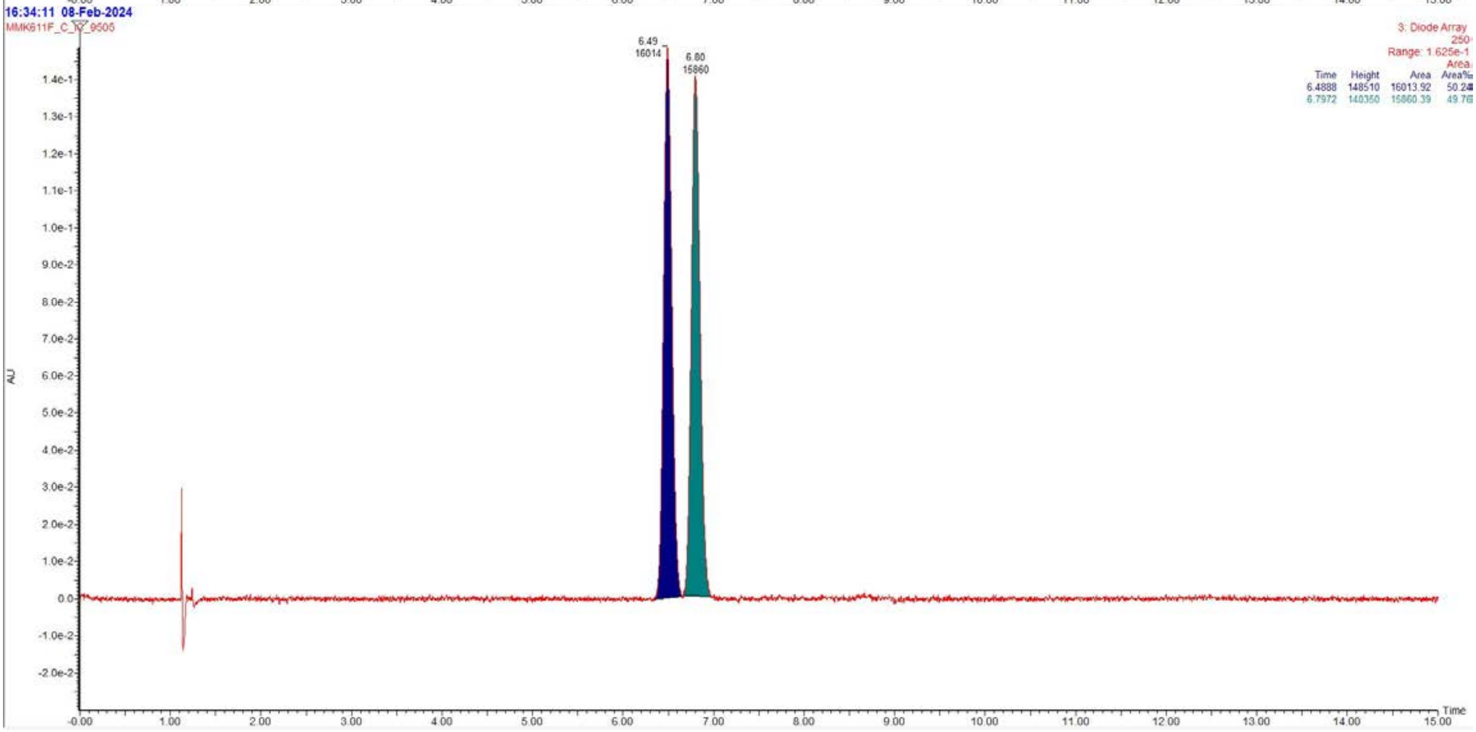

(*R*)-4-(4-(*tert*-butyl)phenyl)-4-Methylnaphthalen-1(4*H*)-one (2aa)

Chiral SFC Analysis: CHIRALPAK IE (CO_2:MeOH, 95:05, 2.5 mL min^{-1}, 40 °C, 250 nm) indicated 99% *ee*, t_R = 10.40 (minor), 11.41 (major) minutes.

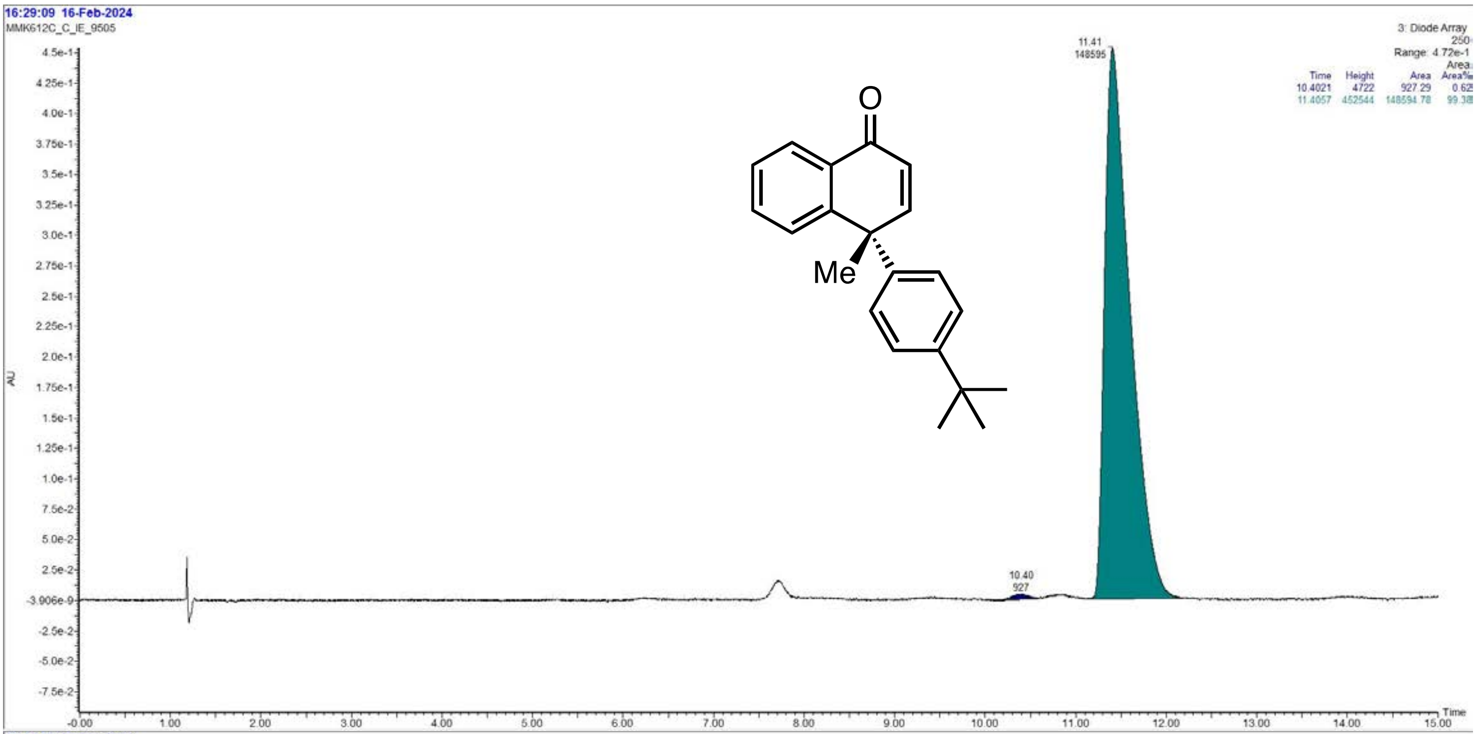

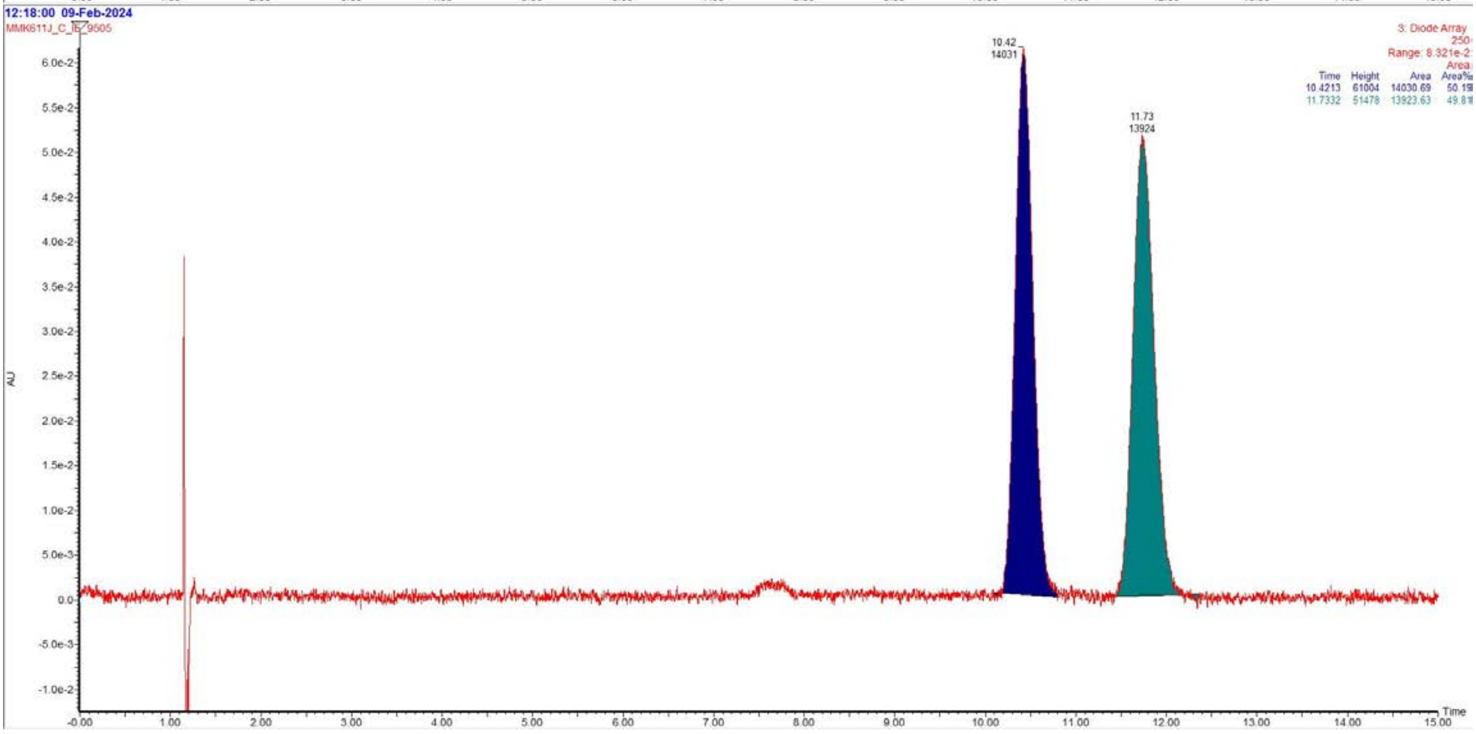

(*R*)-1-Methyl-[1,1'-binaphthalen]-4(1*H*)-one (2ab)

Chiral SFC Analysis: CHIRALPAK IE (CO_2:MeOH, 90:10, 2.5 mL min^{-1}, 40 °C, 260 nm) indicated 96% *ee*, t_R = 9.31 (minor), 9.77 (major) minutes.

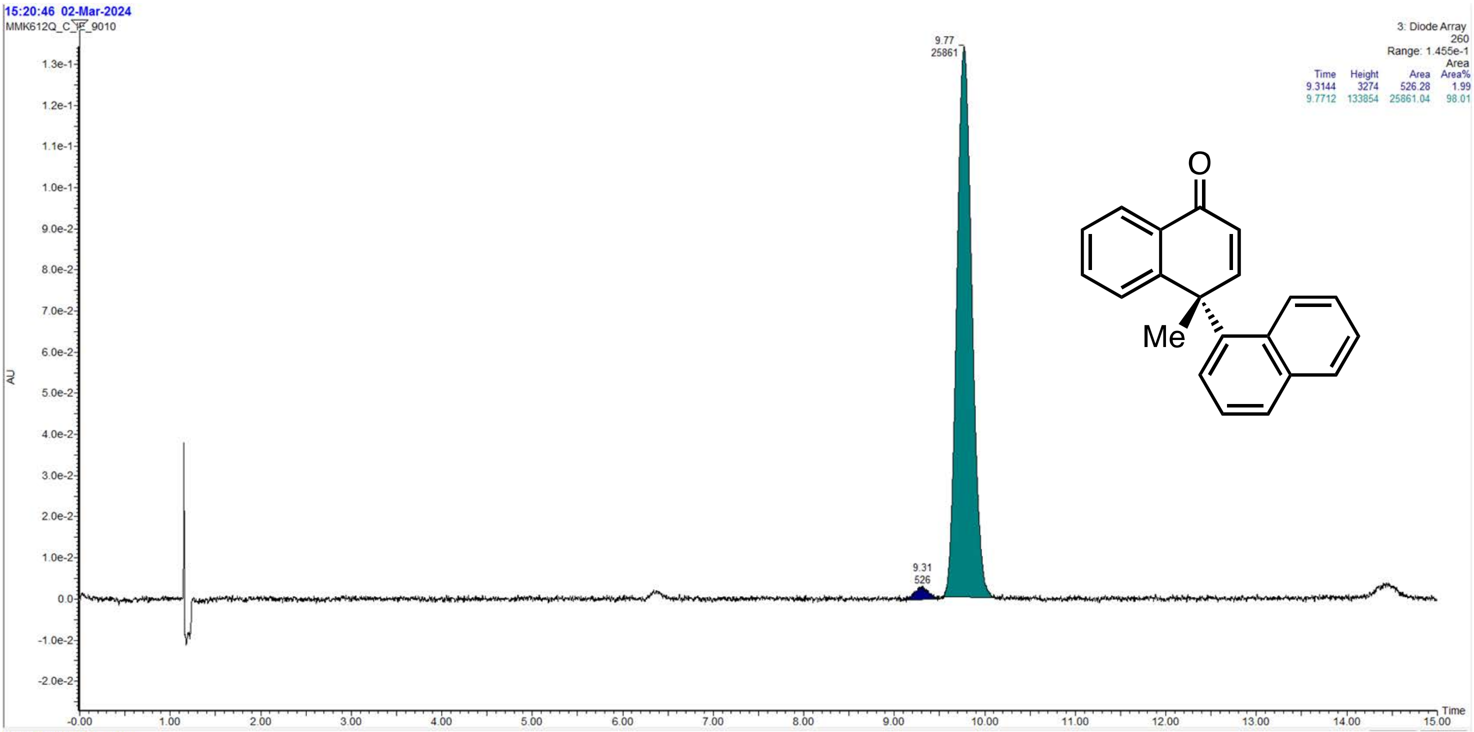

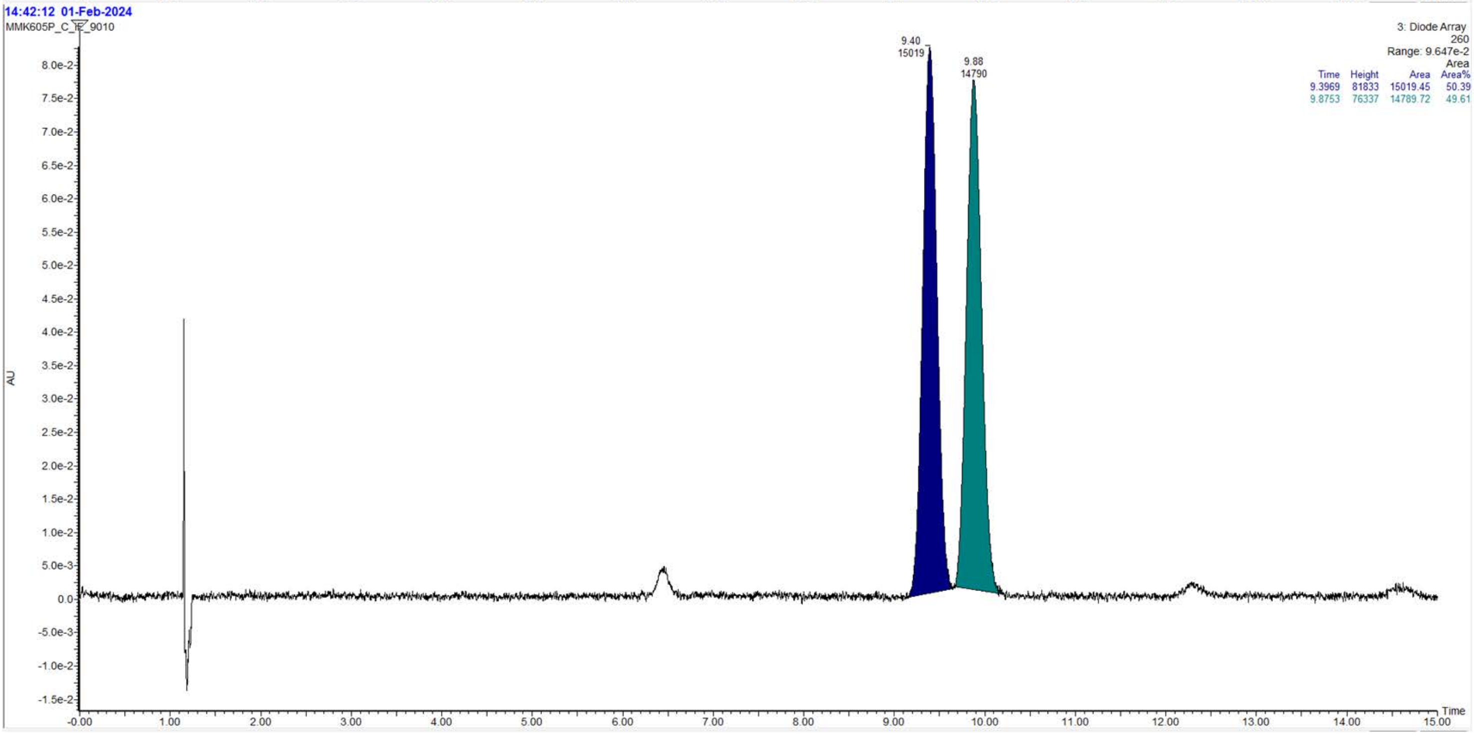

(*R*)-3-(4-(1-methyl-4-oxo-1,4-dihydronaphthalen-1-yl)phenyl)Propanenitrile (2ac)

Chiral SFC Analysis: CHIRALPAK AD (CO_2 (A):0.1% NH_3 in MeOH (B), 5% B – 50% B over two minutes, then isocratic 50% B for 2 minutes, 2.5 mL min^{-1}, 40 °C, 220 nm) indicated 90% *ee*, t_R = 1.24 (minor), 1.38 (major) minutes.

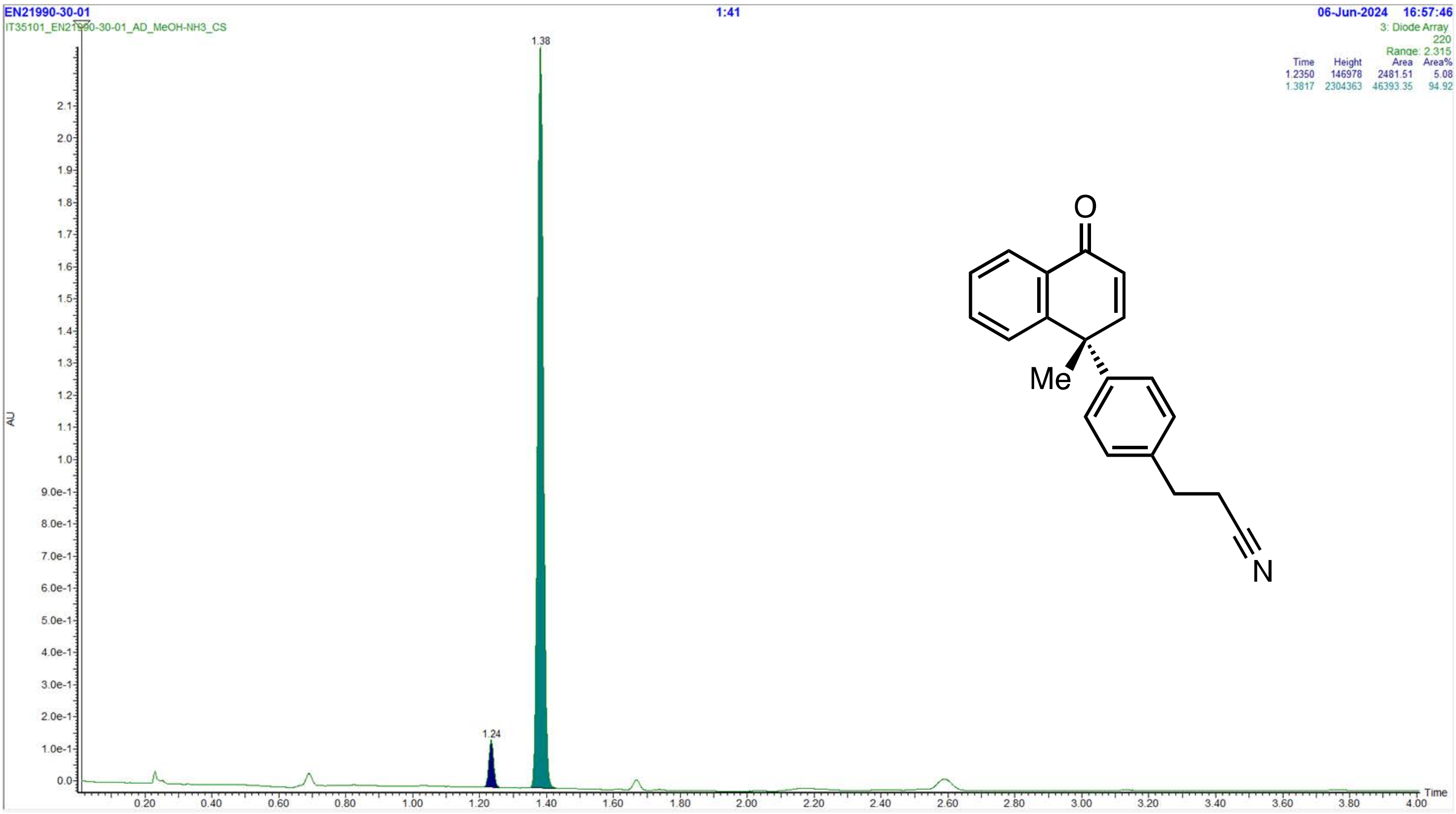

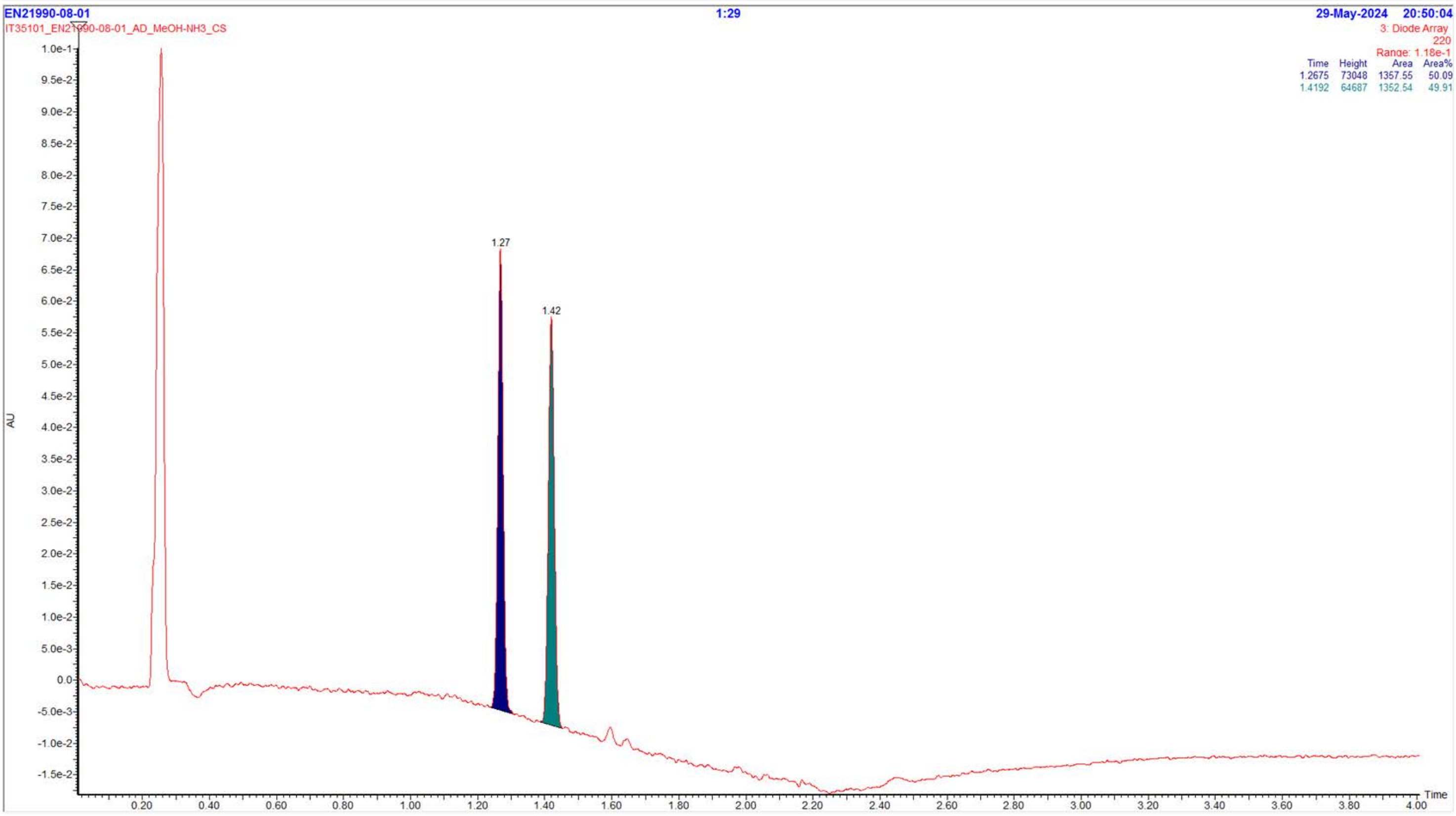

(*R*)-4-(benzofuran-5-yl)-4-Methylnaphthalen-1(4*H*)-one (2ad)

Chiral SFC Analysis: CHIRALPAK AD (CO_2 (A):0.1% NH_3 in MeOH (B), 5% B – 50% B over two minutes, then isocratic 50% B for 2 minutes, 2.5 mL min^{-1}, 40 °C, 250 nm) indicated 93% *ee*, t_R = 1.06 (minor), 1.23 (major) minutes.

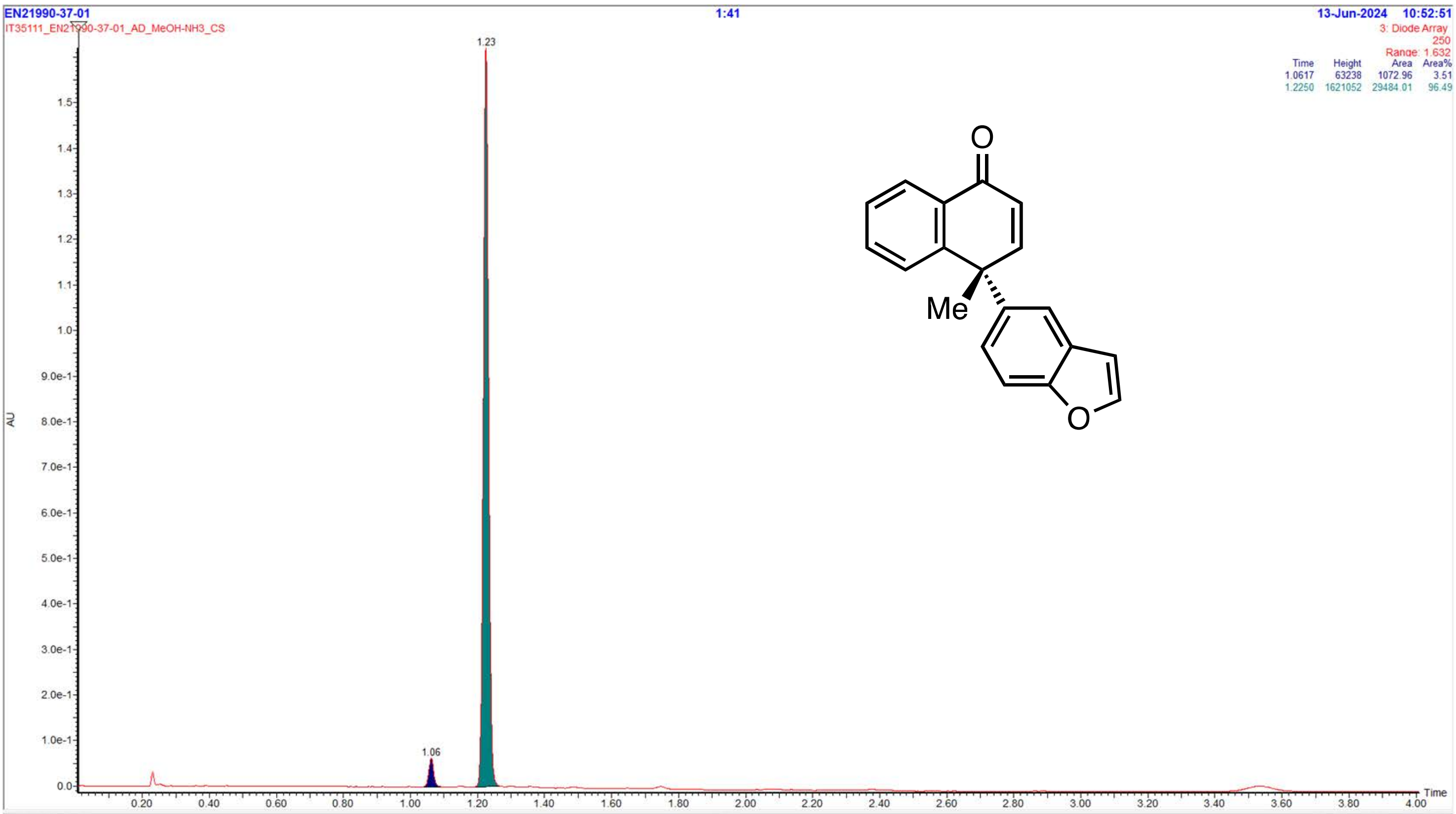

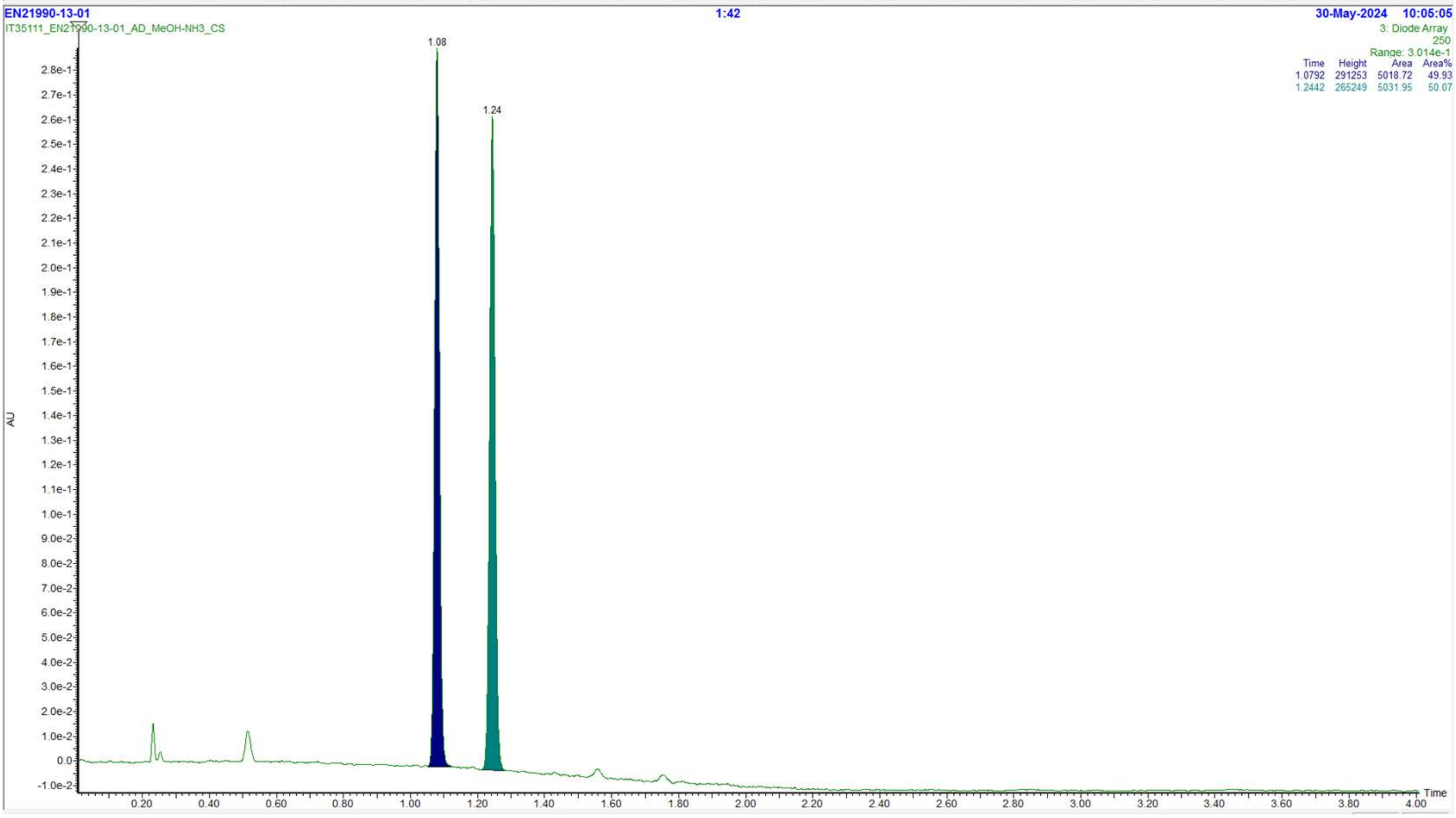

(*R*)-4-(1*H*-indol-6-yl)-4-Methylnaphthalen-1(4*H*)-one (2ae)

Chiral SFC Analysis: CHIRALPAK AD (CO_2 (A):0.1% NH_3 in MeOH (B), 5% B – 50% B over two minutes, then isocratic 50% B for 2 minutes, 2.5 mL min^{-1}, 40 °C, 230 nm) indicated 85% *ee*, t_R = 1.46 (minor), 1.56 (major) minutes.

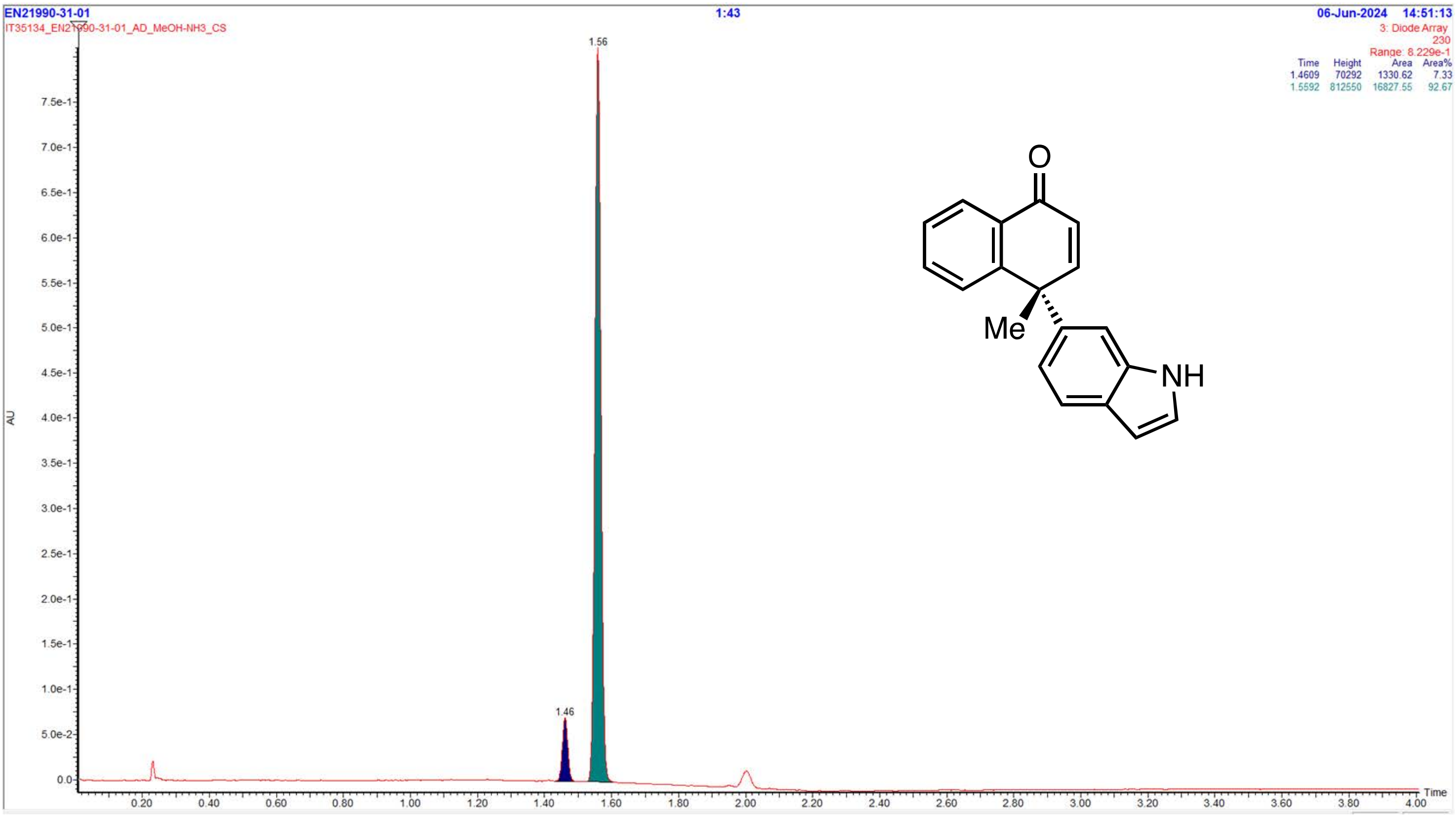

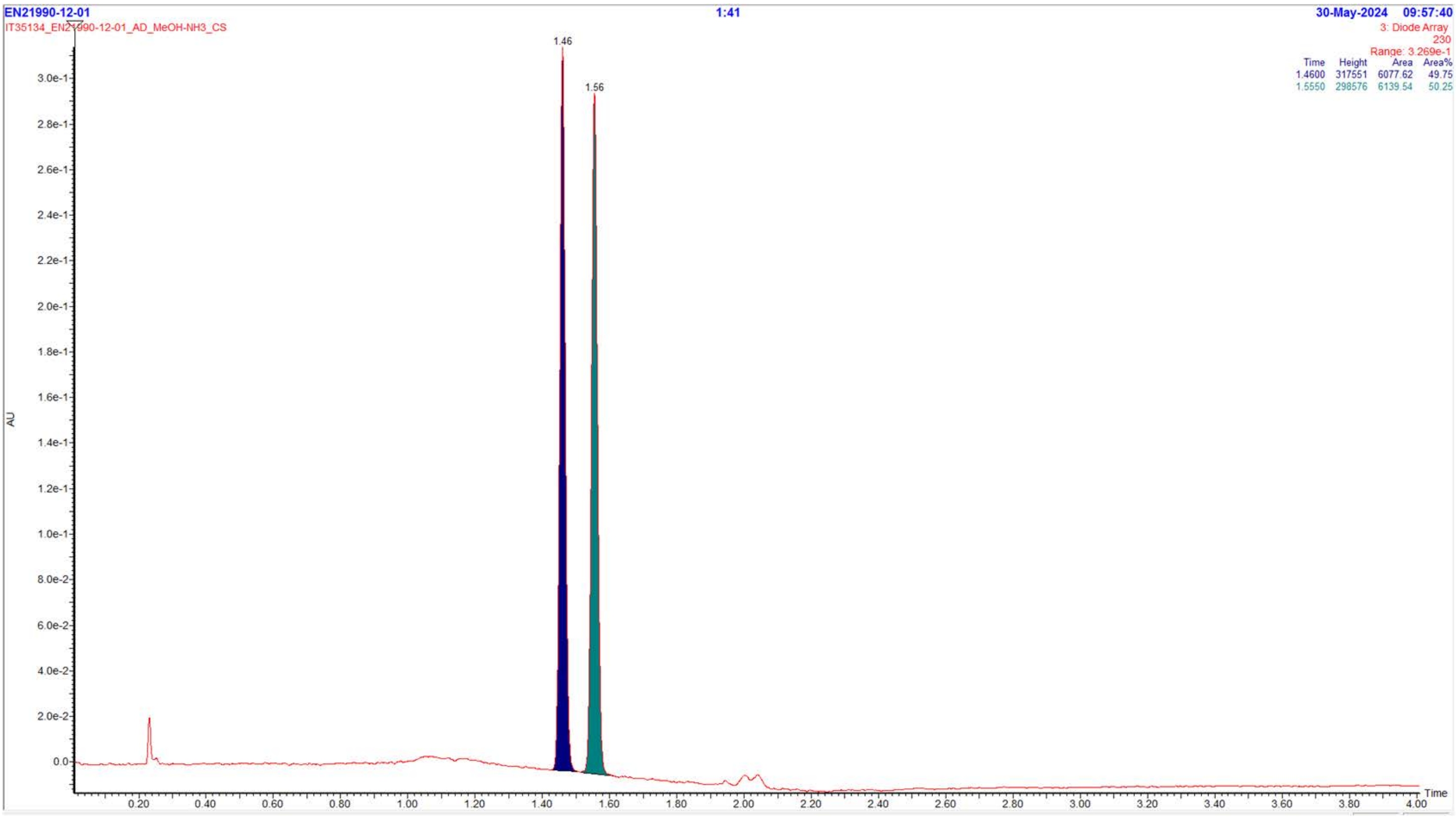

(*R*)-4-(1*H*-indol-5-yl)-4-Methylnaphthalen-1(4*H*)-one (2af)

Chiral SFC Analysis: CHIRALPAK IE (CO_2:MeOH, 80:20, 2.5 mL min^{-1}, 40 °C, 270 nm) indicated 97% *ee*, t_R = 5.79 (minor), 9.52 (major) minutes.

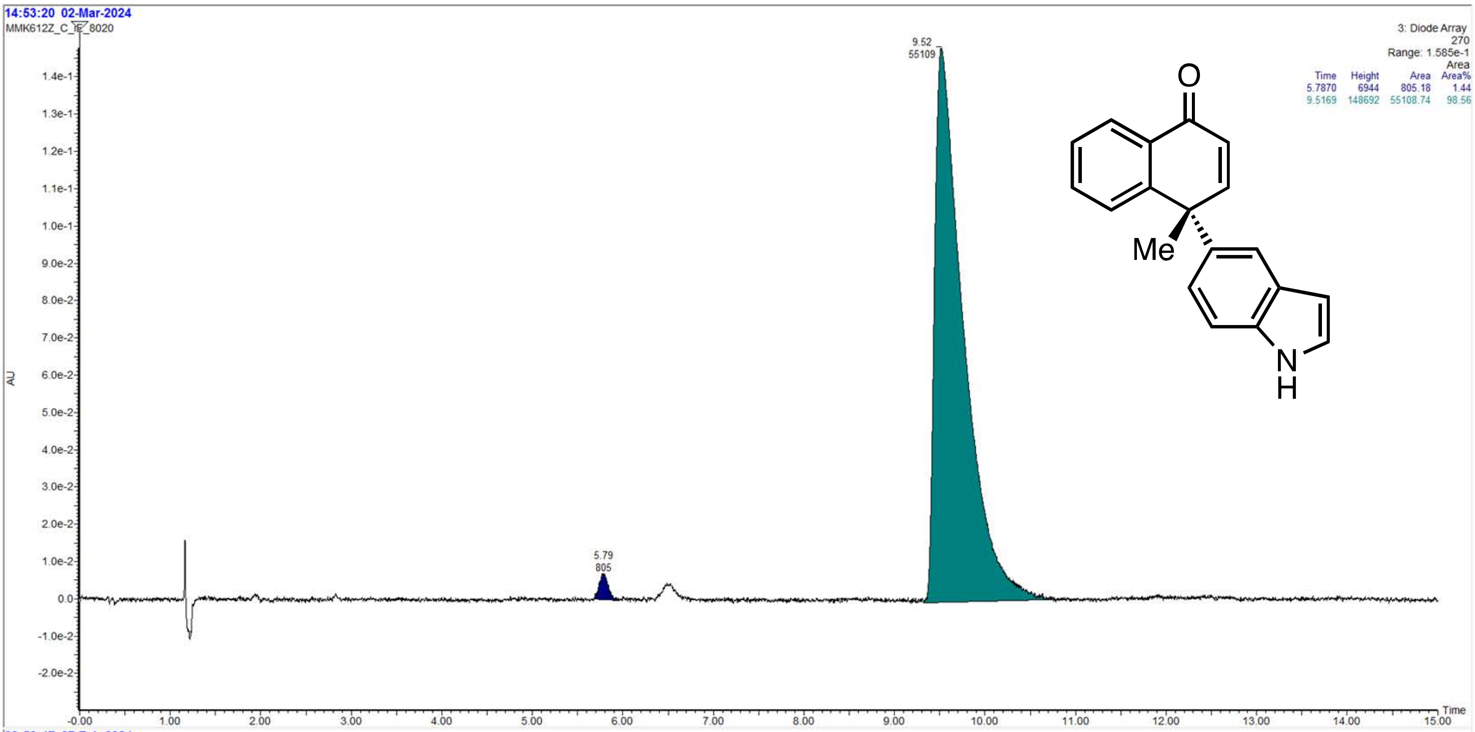

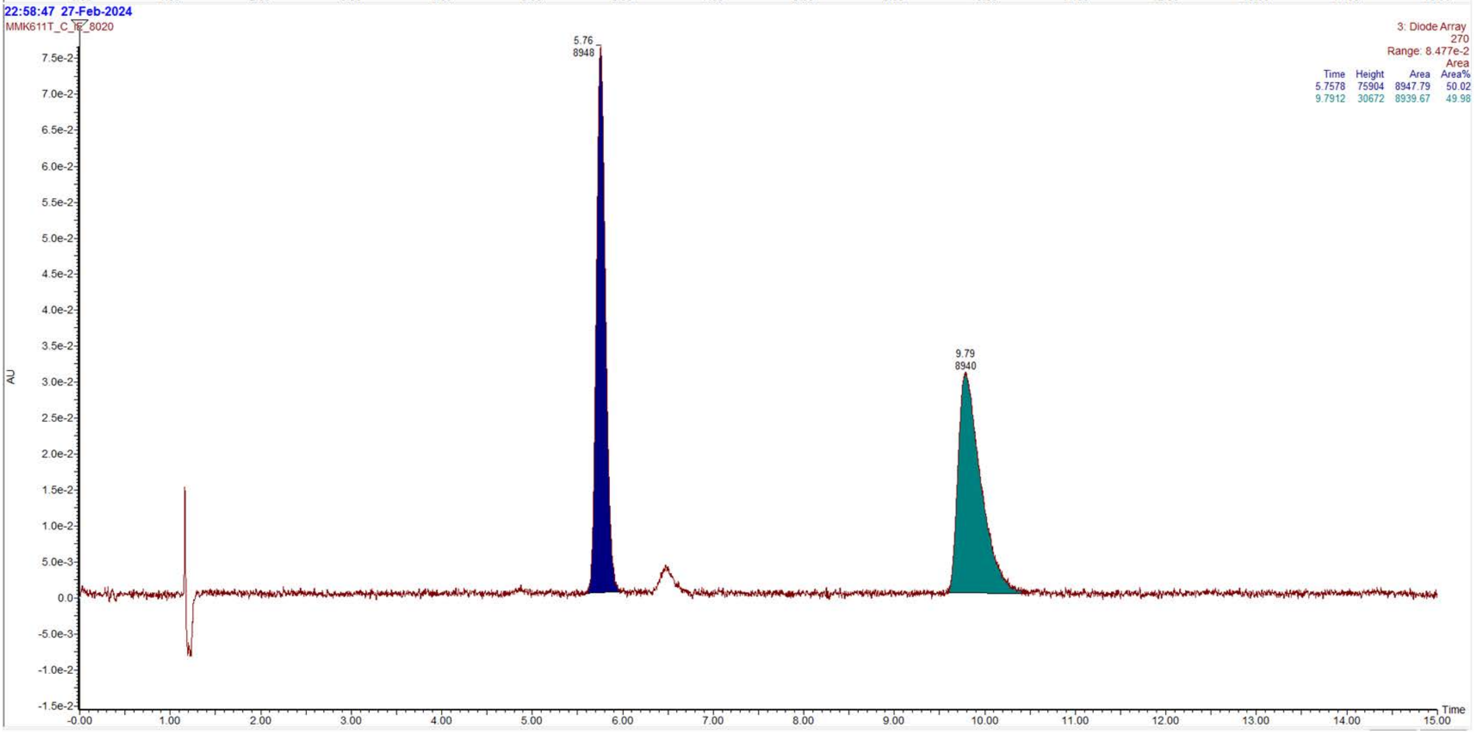

(*S*)-4-Methyl-4-(thiophen-3-yl)naphthalen-1(4*H*)-one (2ag)

Chiral SFC Analysis: CHIRALPAK IE (CO_2:MeOH, 90:10, 2.5 mL min^{-1}, 40 °C, 250 nm) indicated 96% *ee*, t_R = 6.06 (minor), 6.47 (major) minutes.

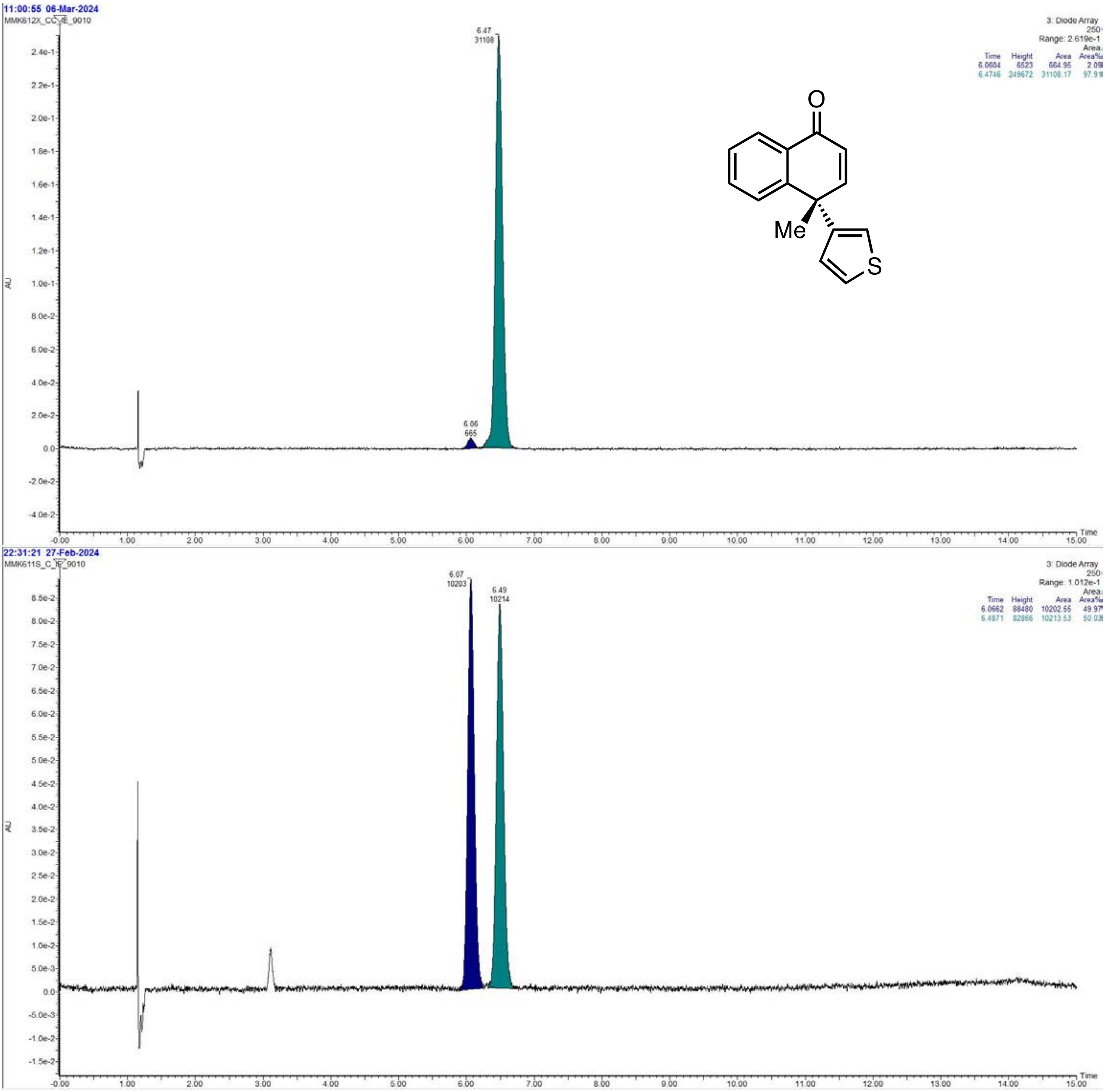

(*R*)-4-(cyclohex-1-en-1-yl)-4-Methylnaphthalen-1(4*H*)-one (2al)

Chiral SFC Analysis: CHIRALPAK AD (CO_2 (A):0.1% NH_3 in MeOH (B), 5% B – 50% B over two minutes, then isocratic 50% B for 2 minutes, 2.5 mL min^{-1}, 40 °C, 250 nm) indicated 98% *ee*, t_R = 0.82 (minor), 0.95 (major) minutes.

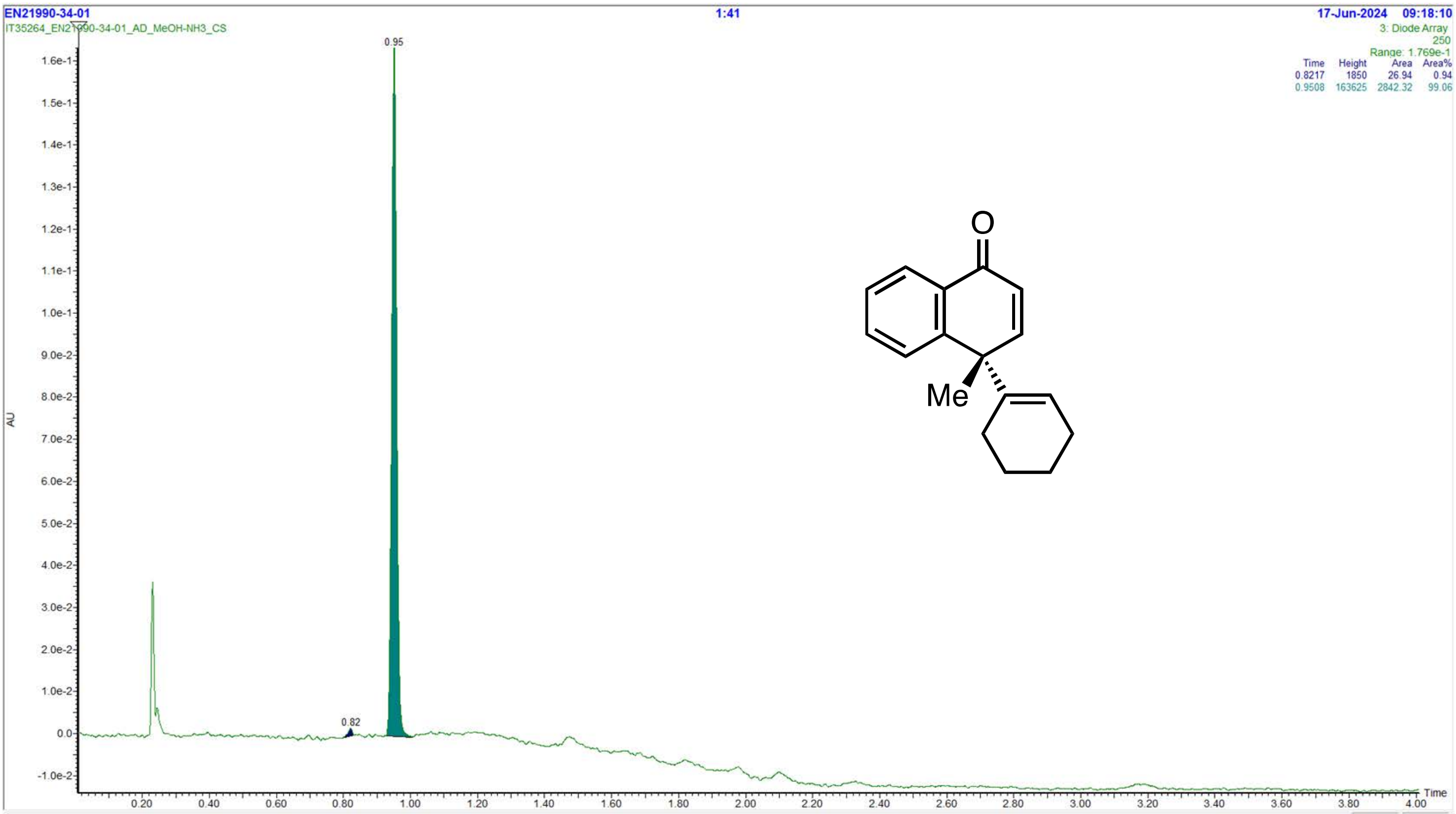

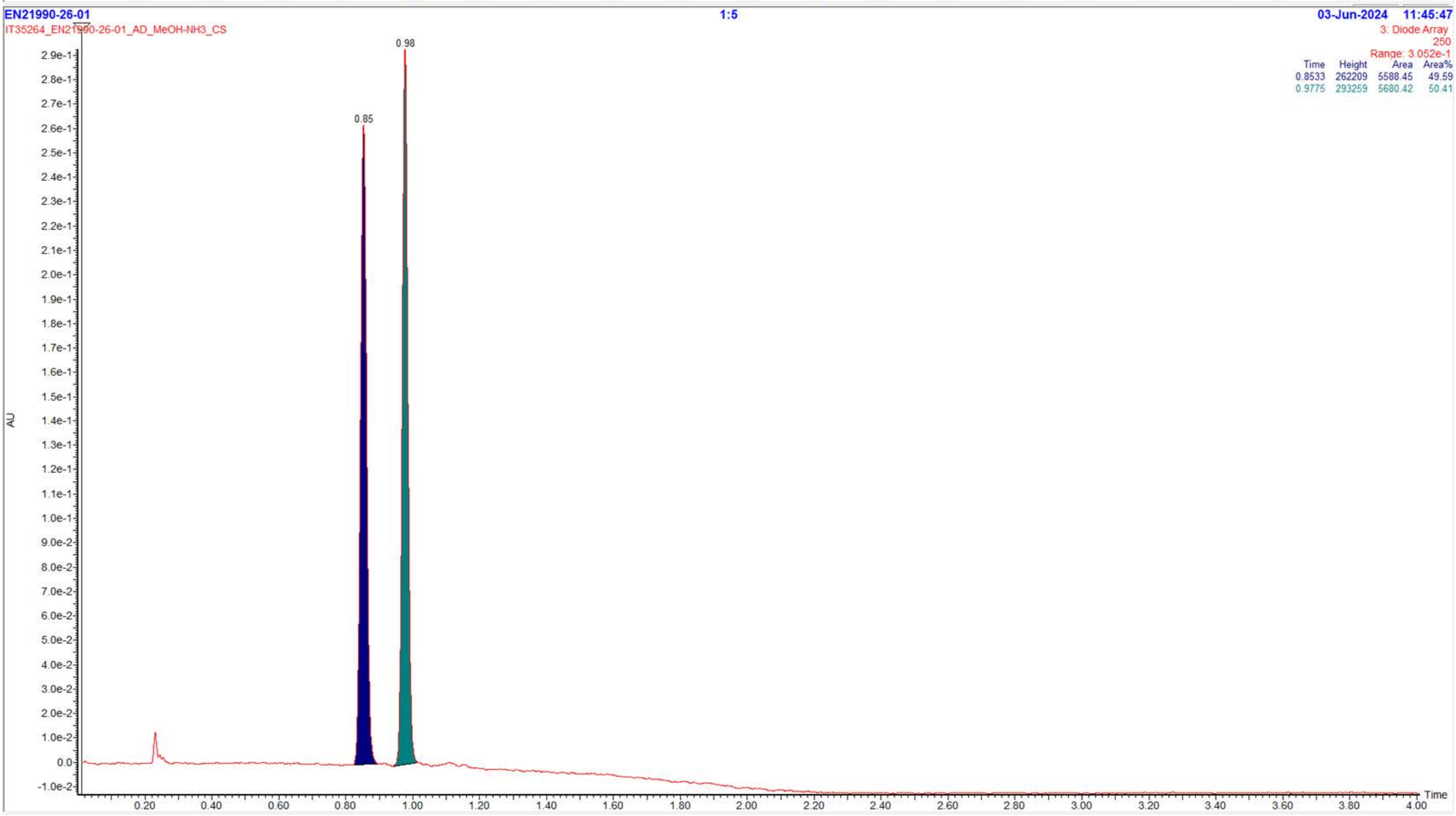

(S,E)-4-Methyl-4-styrylnaphthalen-1(4H)-one (2am)

Chiral SFC Analysis: CHIRALPAK IJ (CO_2:MeOH, 97:03, 2.5 mL min^{-1}, 40 °C, 250 nm) indicated 98% *ee*, t_R = 4.64 (major), 5.02 (minor) minutes.

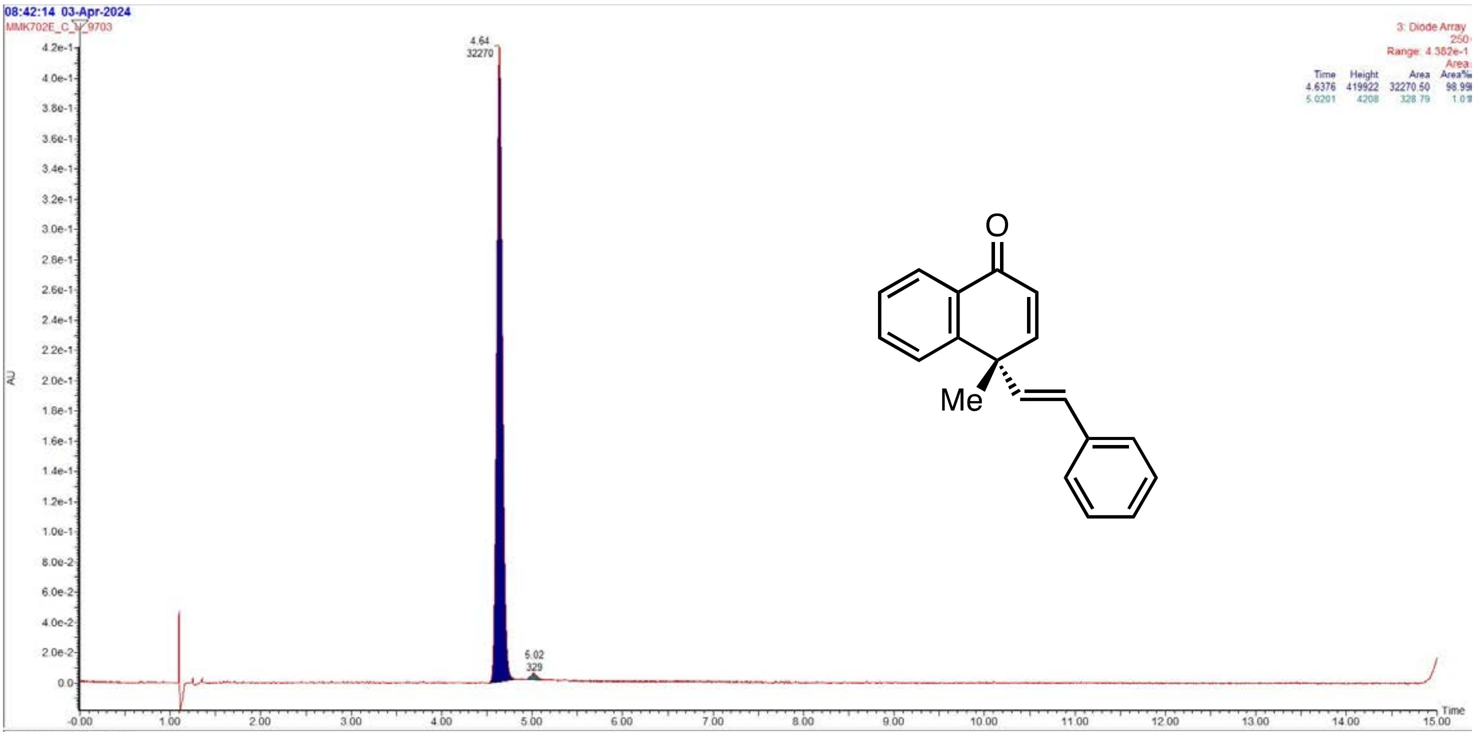

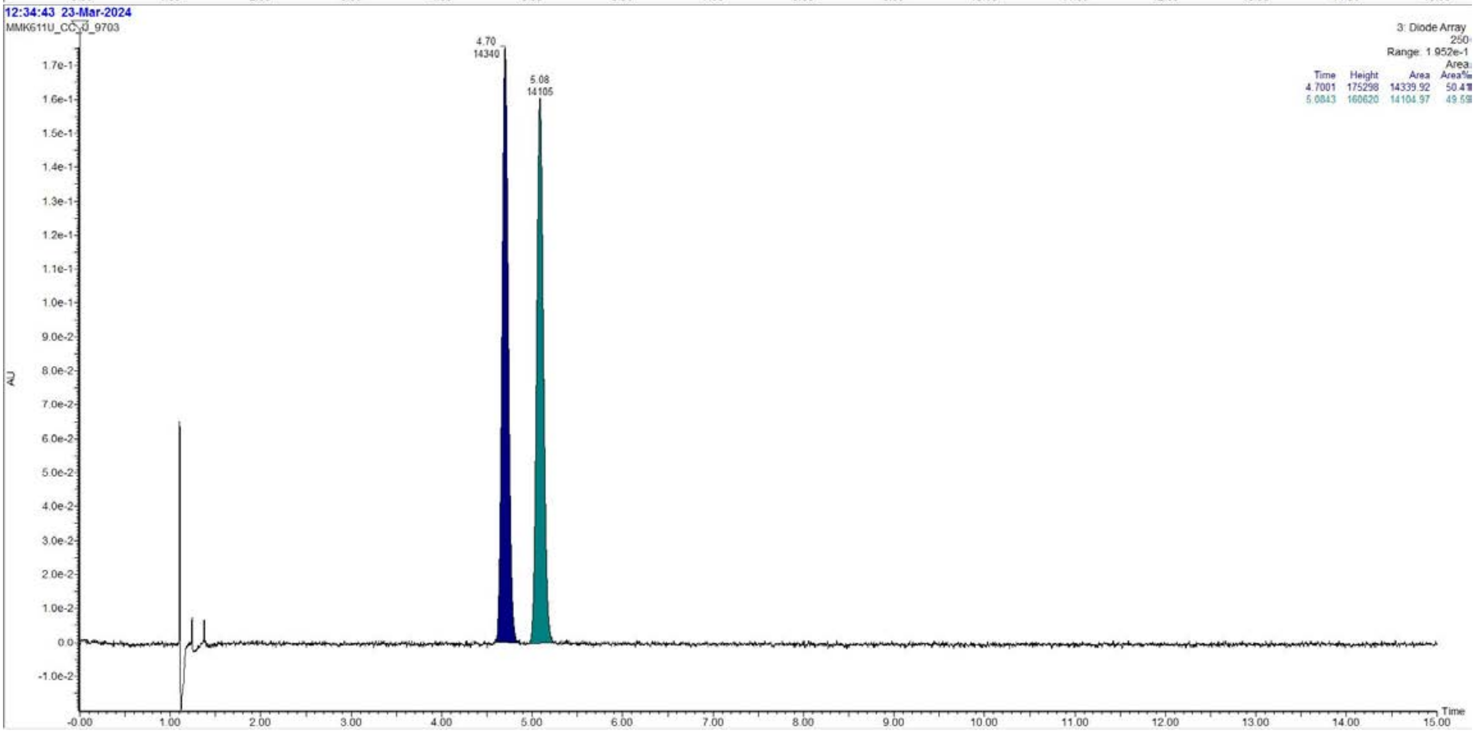

(*R*)-4-(9,9-dibutyl-9*H*-fluoren-2-yl)-4-Methylnaphthalen-1(4*H*)-one (2an)

Chiral SFC Analysis: CHIRALPAK IA (CO_2 (A):0.1% DEA in IPA (B), 5% B – 50% B over two minutes, then isocratic 50% B for 2 minutes, 2.5 mL min^{-1}, 40 °C, 250 nm) indicated 95% *ee*, t_R = 1.13 (minor), 1.20 (major) minutes.

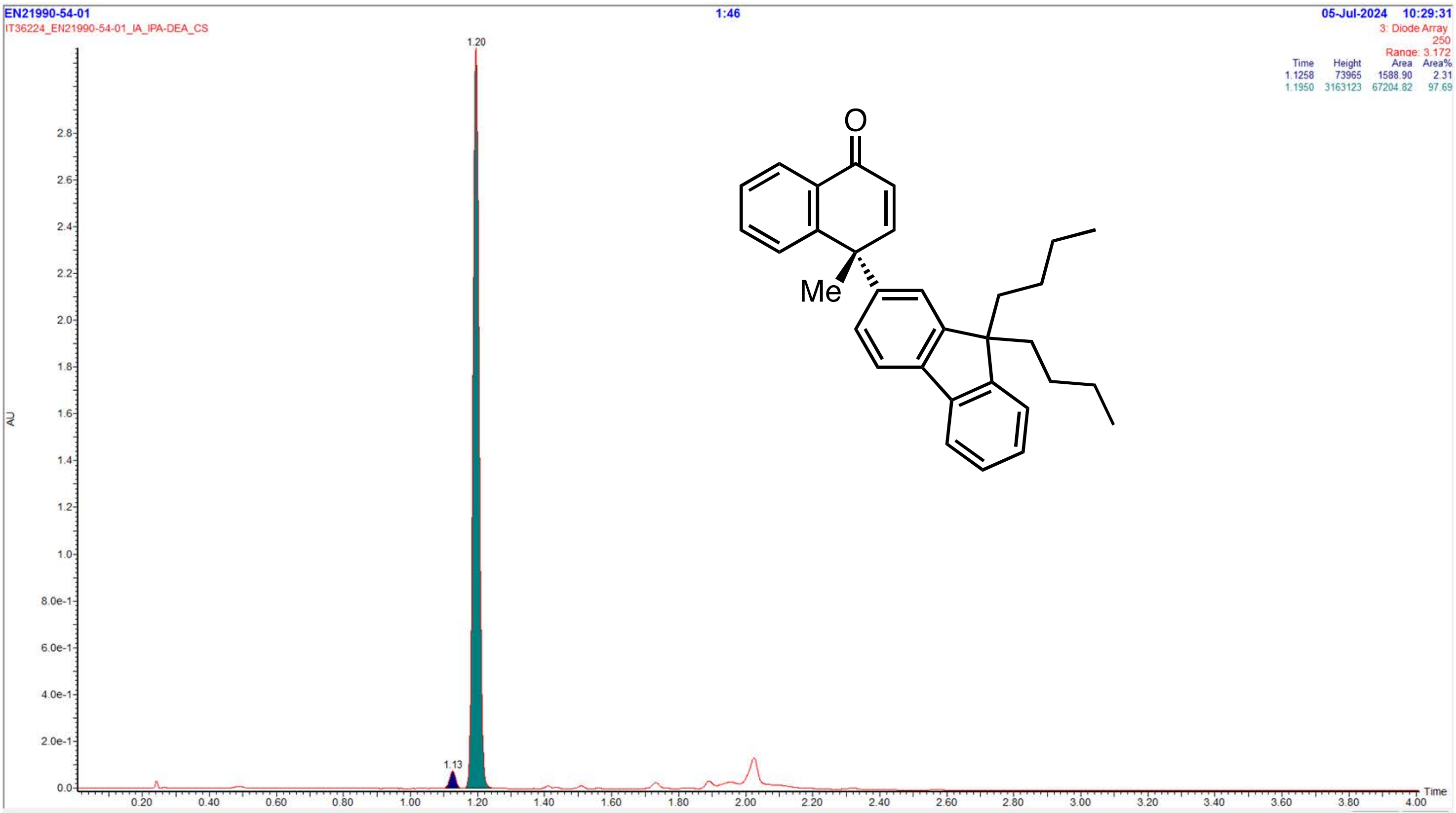

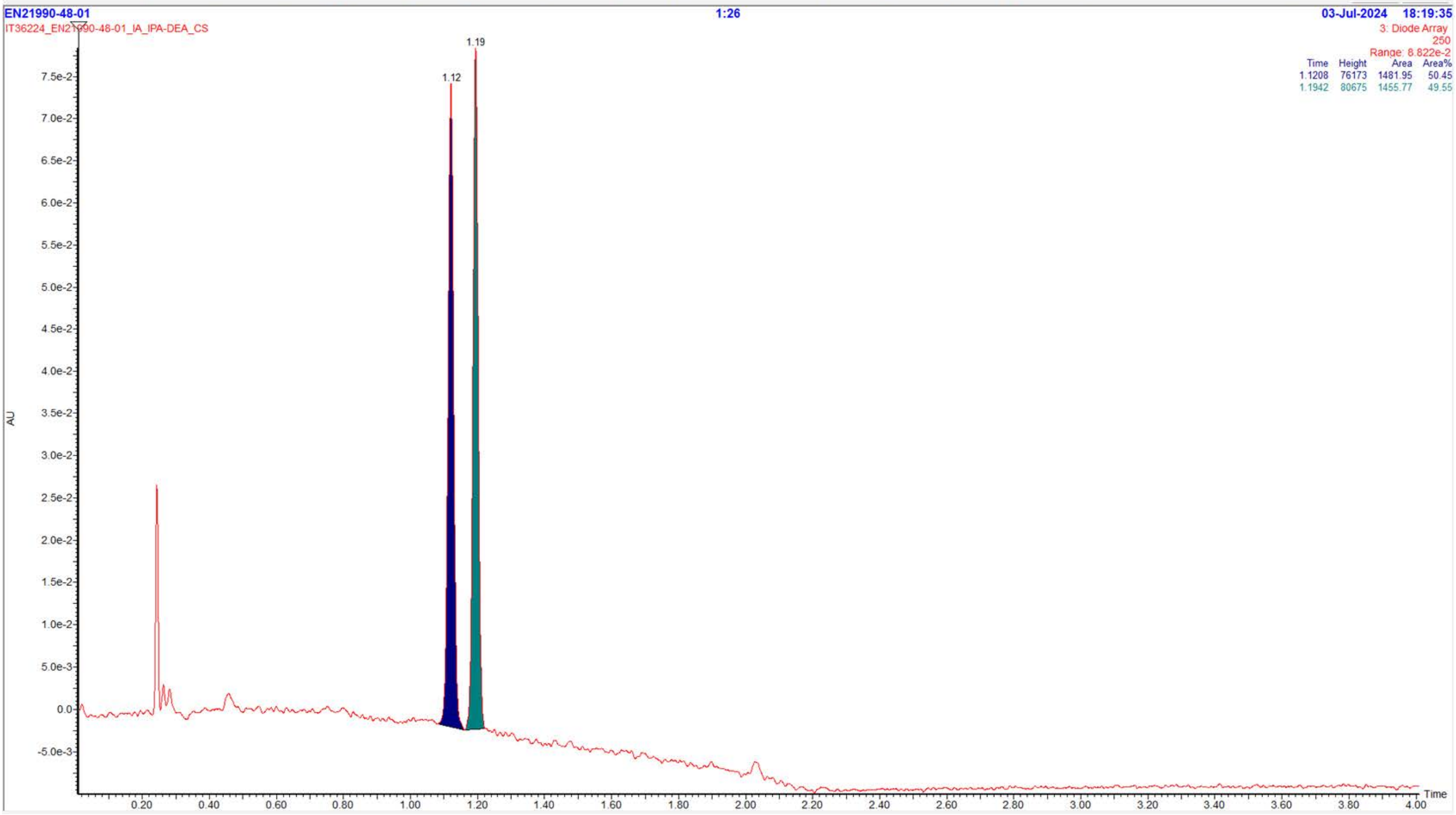

Benzyl (*R*)-4-(4-(1-methyl-4-oxo-1,4-dihydronaphthalen-1-yl)phenyl)Piperazine-1-carboxylate (2ao)

Chiral SFC Analysis: CHIRALPAK IA (CO_2 (A):0.1% DEA in IPA (B), 5% B – 50% B over two minutes, then isocratic 50% B for 2 minutes, 2.5 mL min^{-1}, 40 °C, 250 nm) indicated 96% *ee*, t_R = 2.14 (minor), 2.29 (major) minutes.

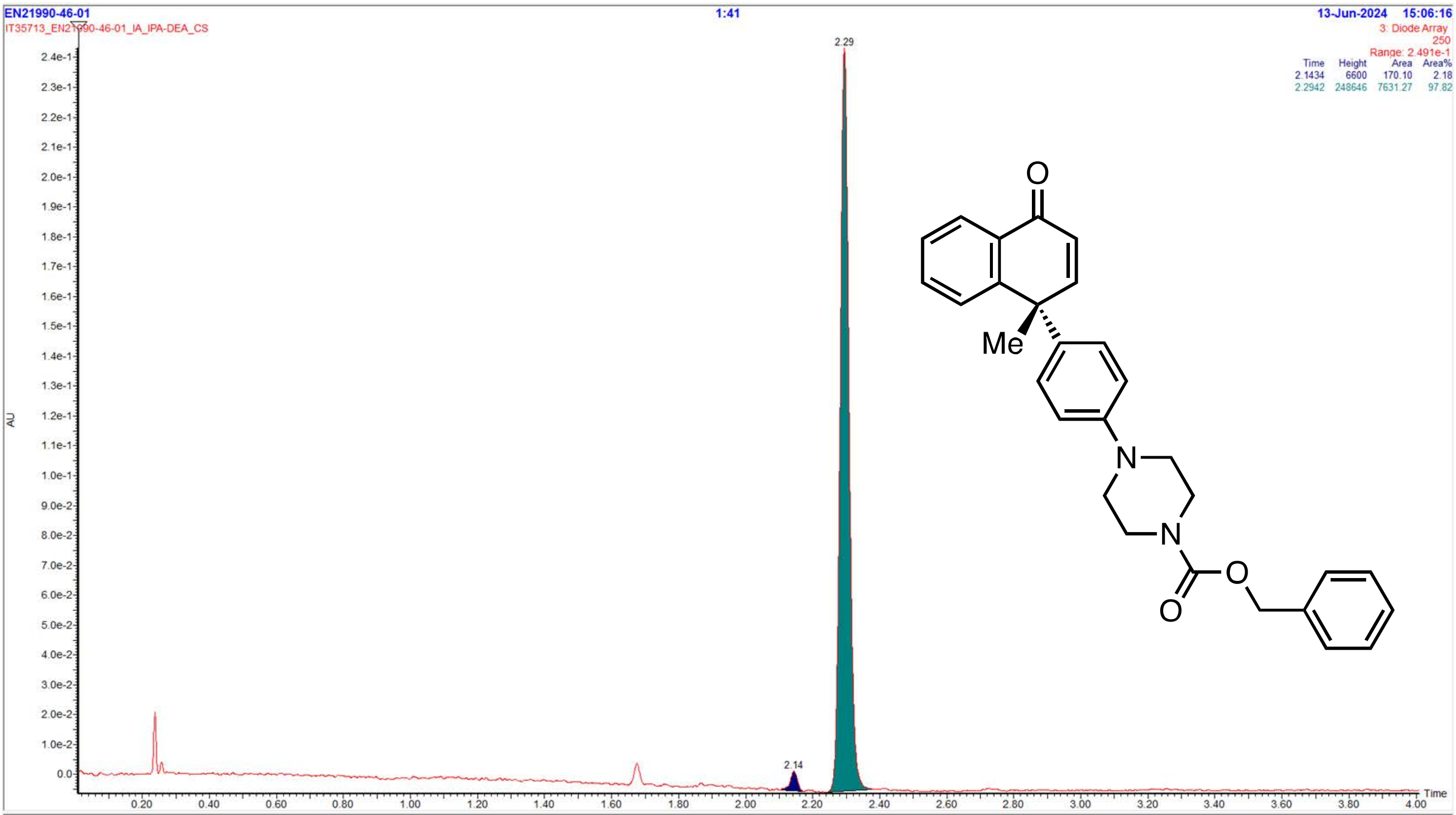

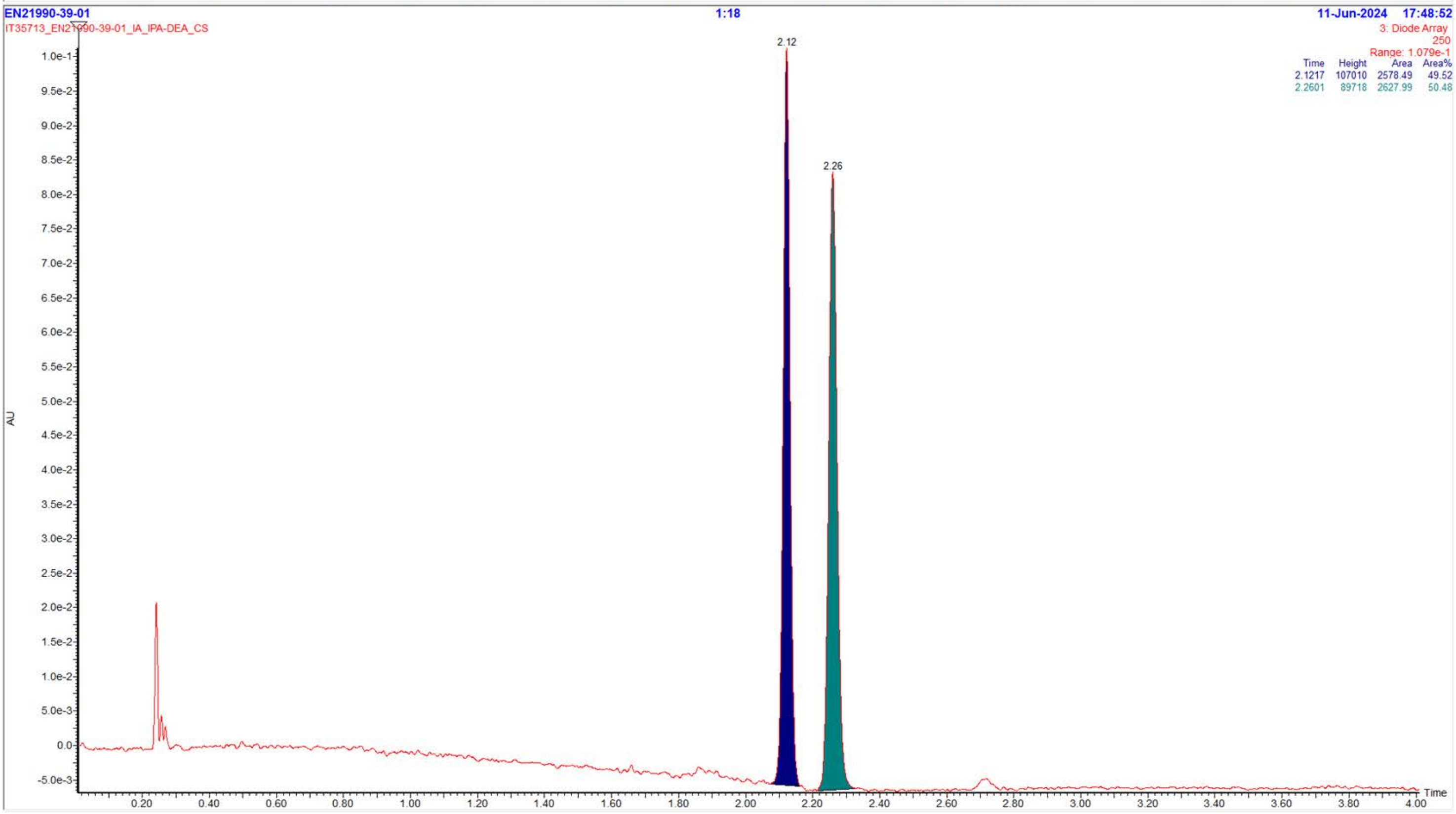

(*R*)-4-(4-(2,6-diphenylpyrimidin-4-yl)phenyl)-4-Methylnaphthalen-1(4*H*)-one (2ap)

Chiral SFC Analysis: CHIRALPAK IA (CO_2 (A):0.1% NH_3 in MeOH (B), isocratic 50% B, 2.5 mL min^{-1}, 40 °C, 254 nm) indicated 89% *ee*, t_R = 2.05 (minor), 2.61 (major) minutes.

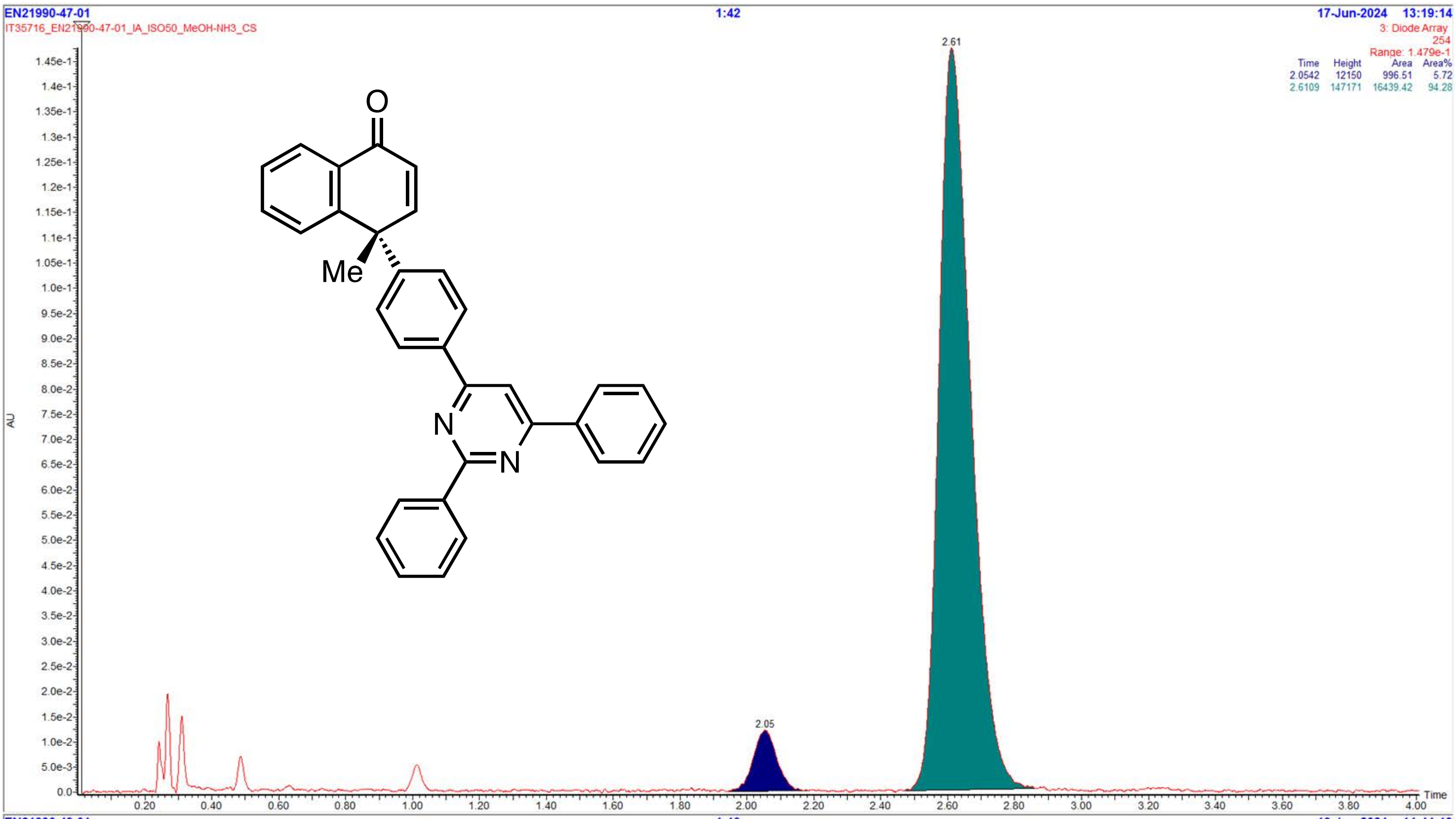

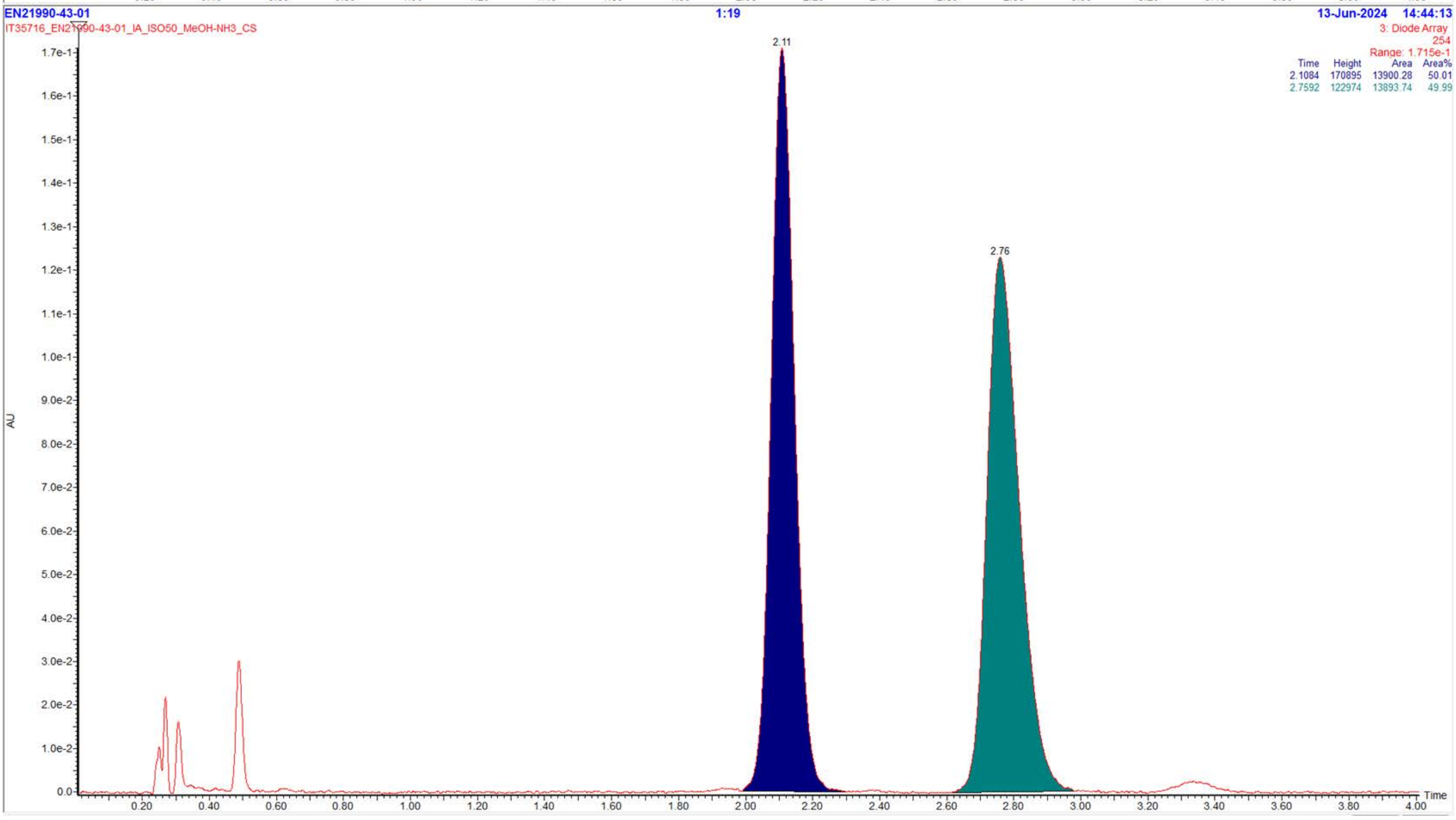

(*R*)-4-(4-chloro-3-(4-(((*R*)-tetrahydrofuran-3-yl)oxy)benzyl)phenyl)-4-Methylnaphthalen-1(4*H*)-one (2aq)

Chiral SFC Analysis: CHIRALPAK IK (CO_2:MeOH, 70:30, 2.5 mL min^{-1}, 40 °C, 270 nm) indicated 25:1 dr, t_R = 8.15 (major), 8.99 (minor) minutes.

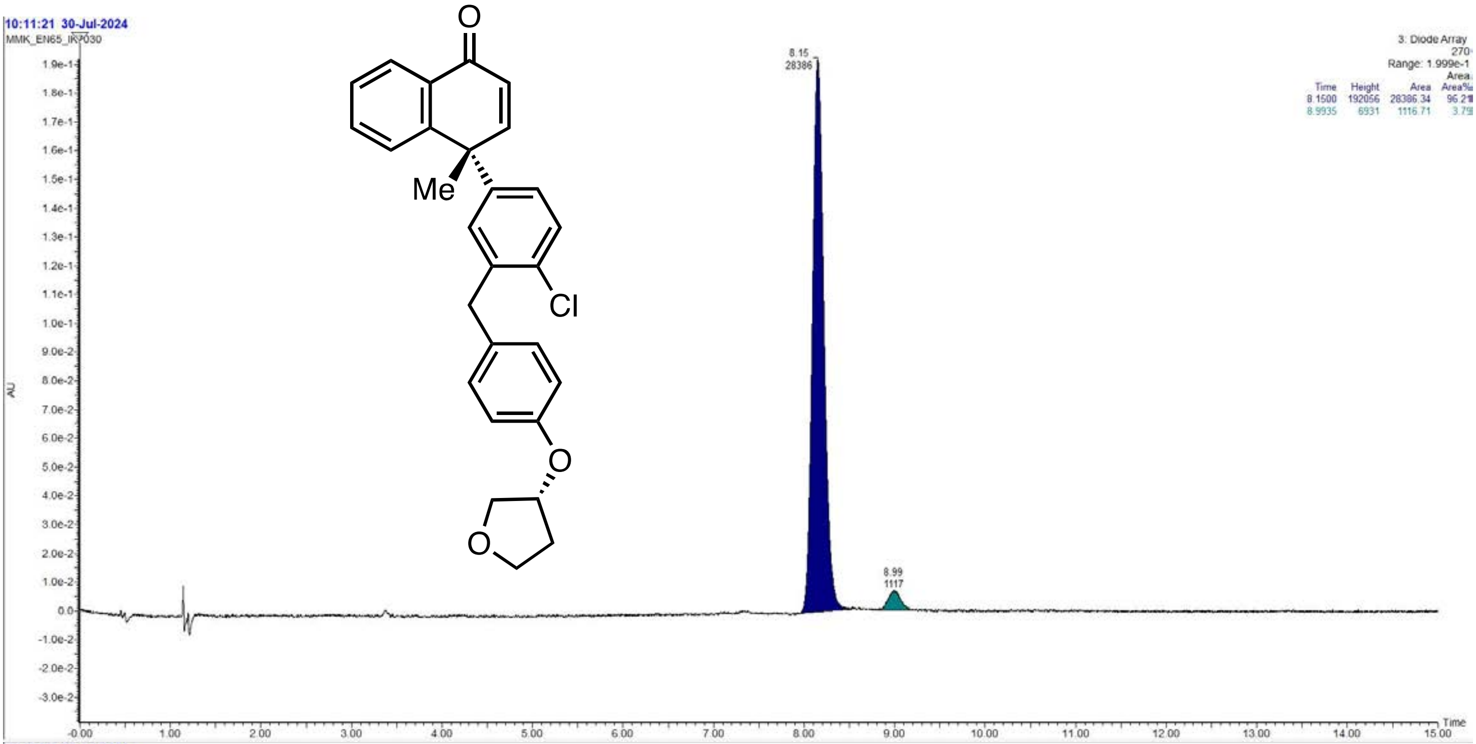

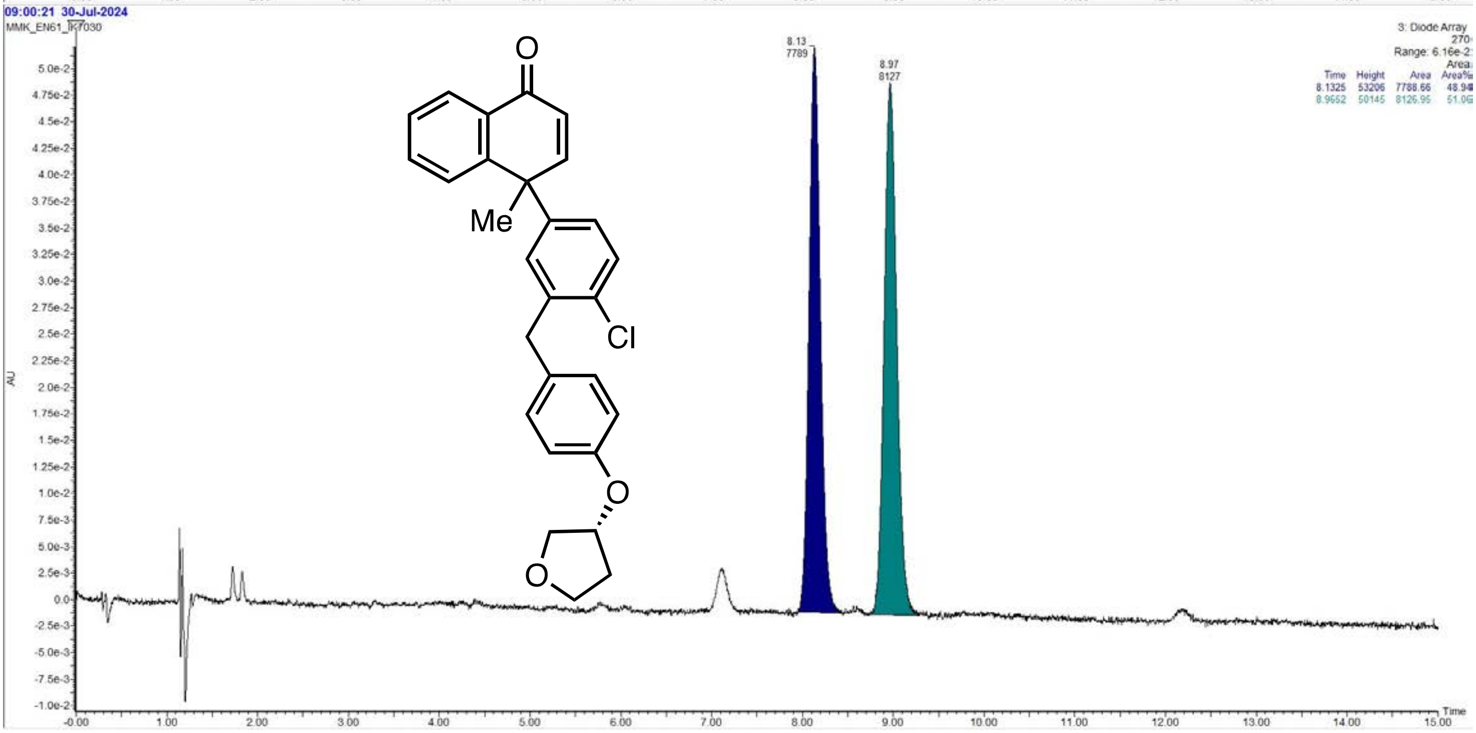

(4*R*,4'*R*)-4,4'-(1,4-phenylene)Bis(4-methylnaphthalen-1(4*H*)-one) (3a)

Chiral SFC Analysis: CHIRALPAK IJ (CO_2:MeOH, 85:15, 2.5 mL min^{-1}, 40 °C, 250 nm) indicated > 99% *ee* and 17:1 dr, t_R = 6.66 (major enantiomer), 7.46 (minor diastereomer [meso compound]), 7.77 (minor enantiomer) minutes.

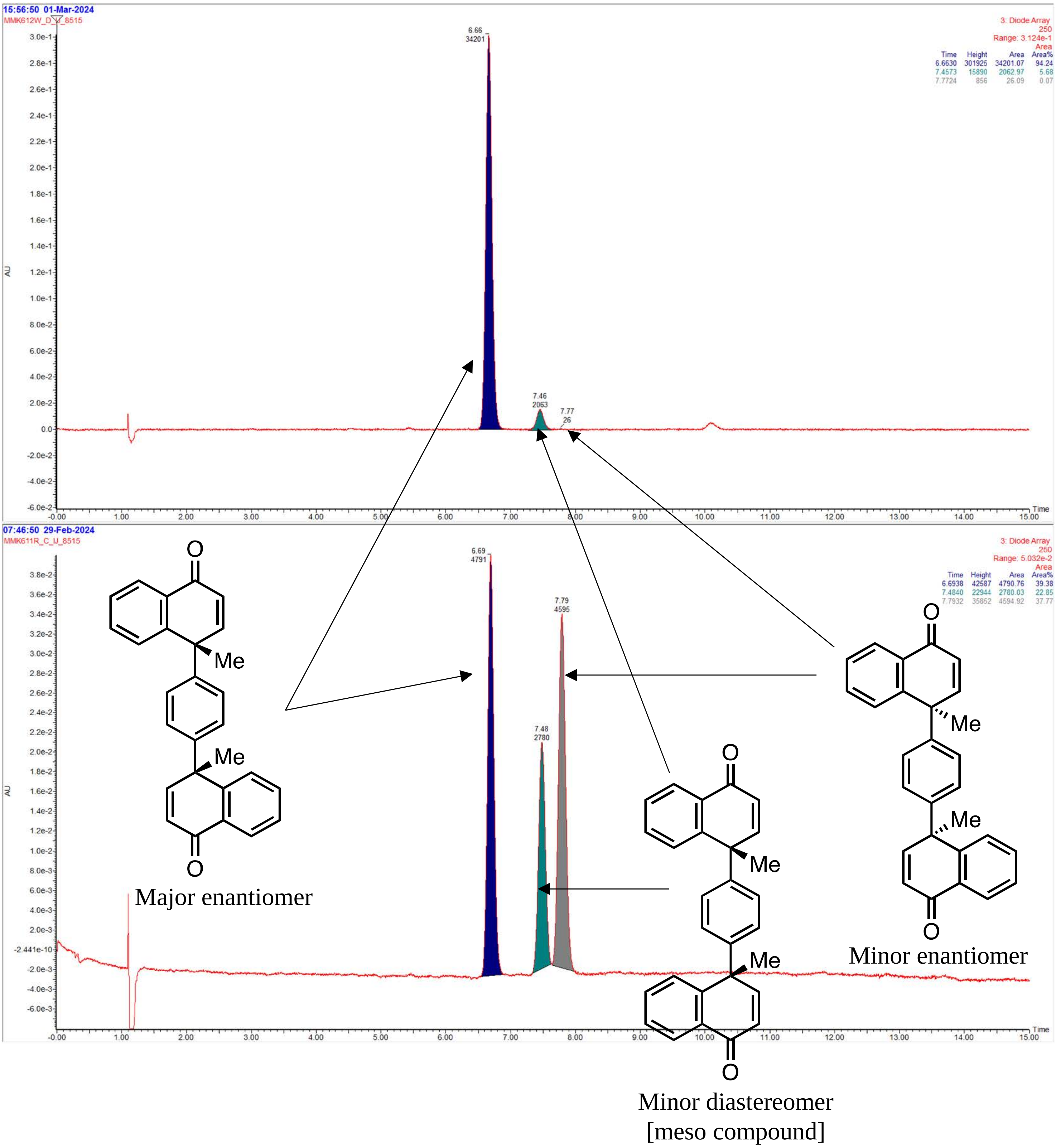

(4*R*,4'*R*)-4,4'-([1,1':4',1''-terphenyl]-4,4''-diyl)Bis(4-methylnaphthalen-1(4*H*)-one) (3b)

Chiral SFC Analysis: CHIRALPAK AD (CO_2 (A):0.1% NH_3 in MeOH (B), isocratic 50% B, 2.5 mL min^{-1}, 40 °C, 290 nm) indicated 99% *ee* and 13:1 dr, t_R = 2.76 (minor enantiomer), 3.31 (minor diastereomer [meso compound]), 3.70 (major enantiomer) minutes.

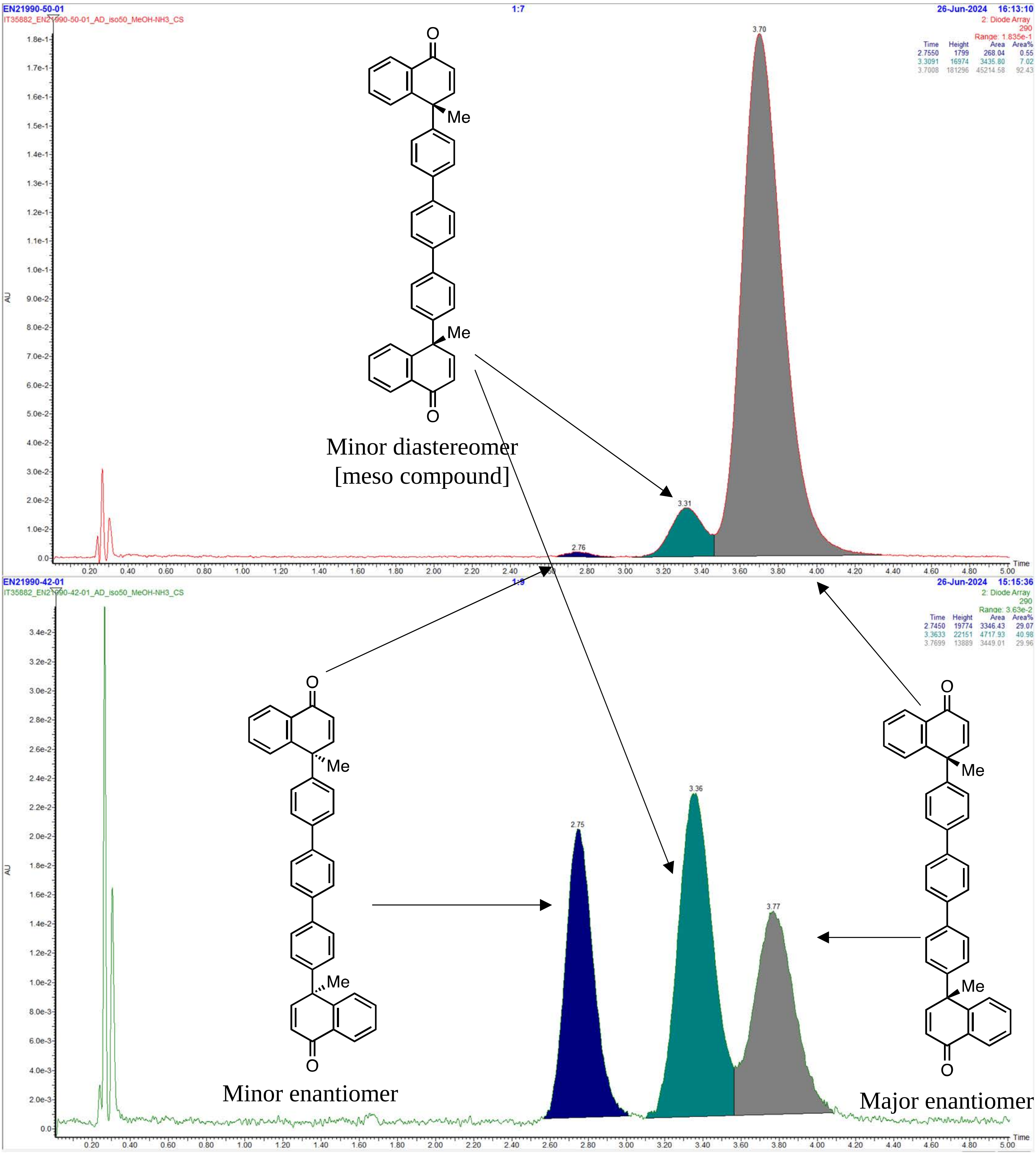

(4*R*,4'*R*)-4,4'-([1,1':3',1''-terphenyl]-4,4''-diyl)Bis(4-methylnaphthalen-1(4*H*)-one) (3c)

Chiral SFC Analysis: CHIRALPAK IF (CO_2 (A):0.1% DEA in IPA (B), isocratic 50% B, 2.5 mL min^{-1}, 40 °C, 254 nm) indicated 99% *ee* and 11:1 dr, t_R = 1.64 (minor enantiomer), 1.85 (minor diastereomer [meso compound]), 2.07 (major enantiomer) minutes.

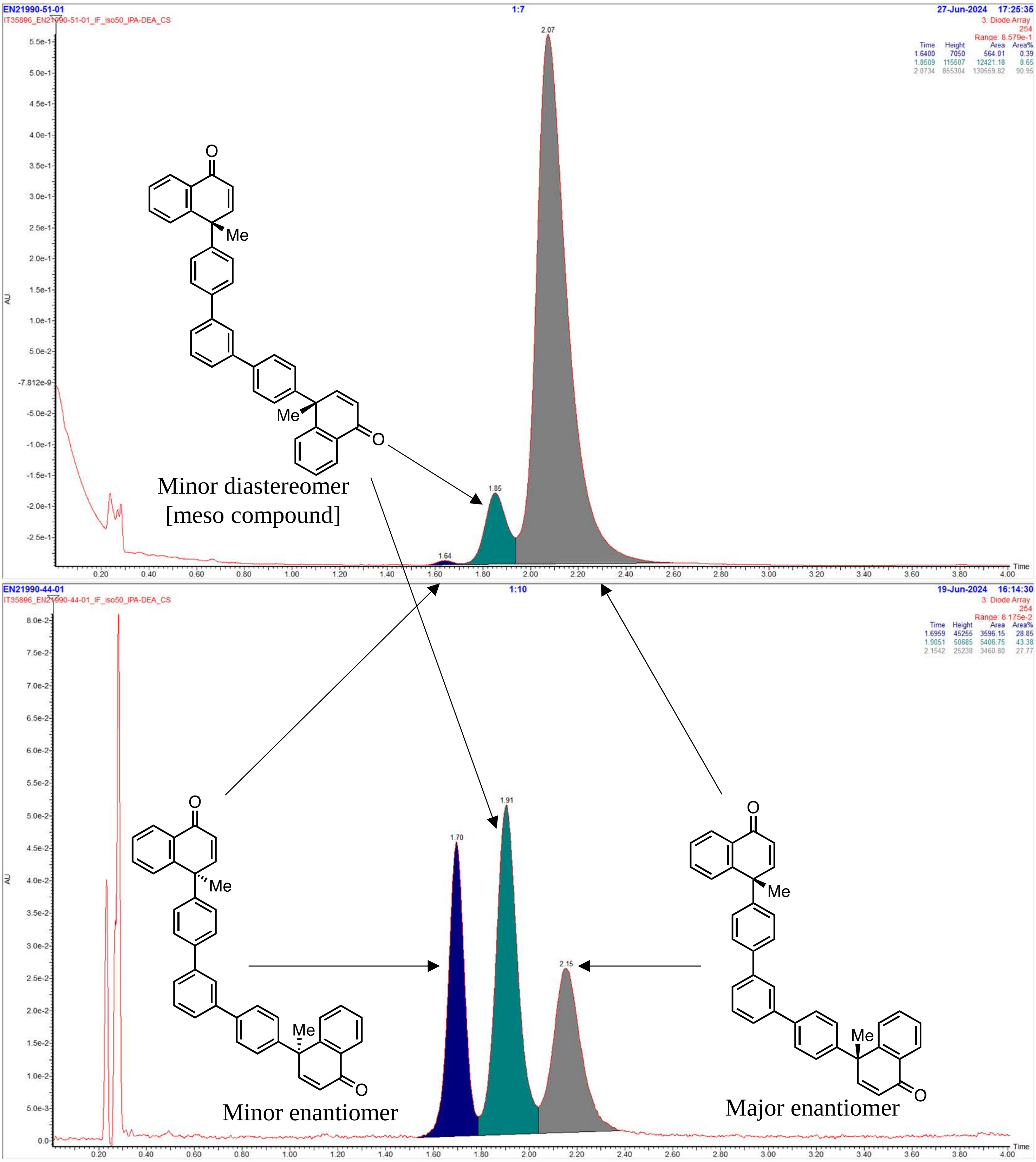

1-Methyl-1-phenylnaphthalen-2(1*H*)-one (S1)

Chiral SFC Analysis: CHIRALPAK IK (CO_2:MeOH, 95:05, 2.5 mL min^{-1}, 40 °C, 300 nm) indicated 15% *ee*, t_R = 9.19 (major), 13.80 (minor) minutes.

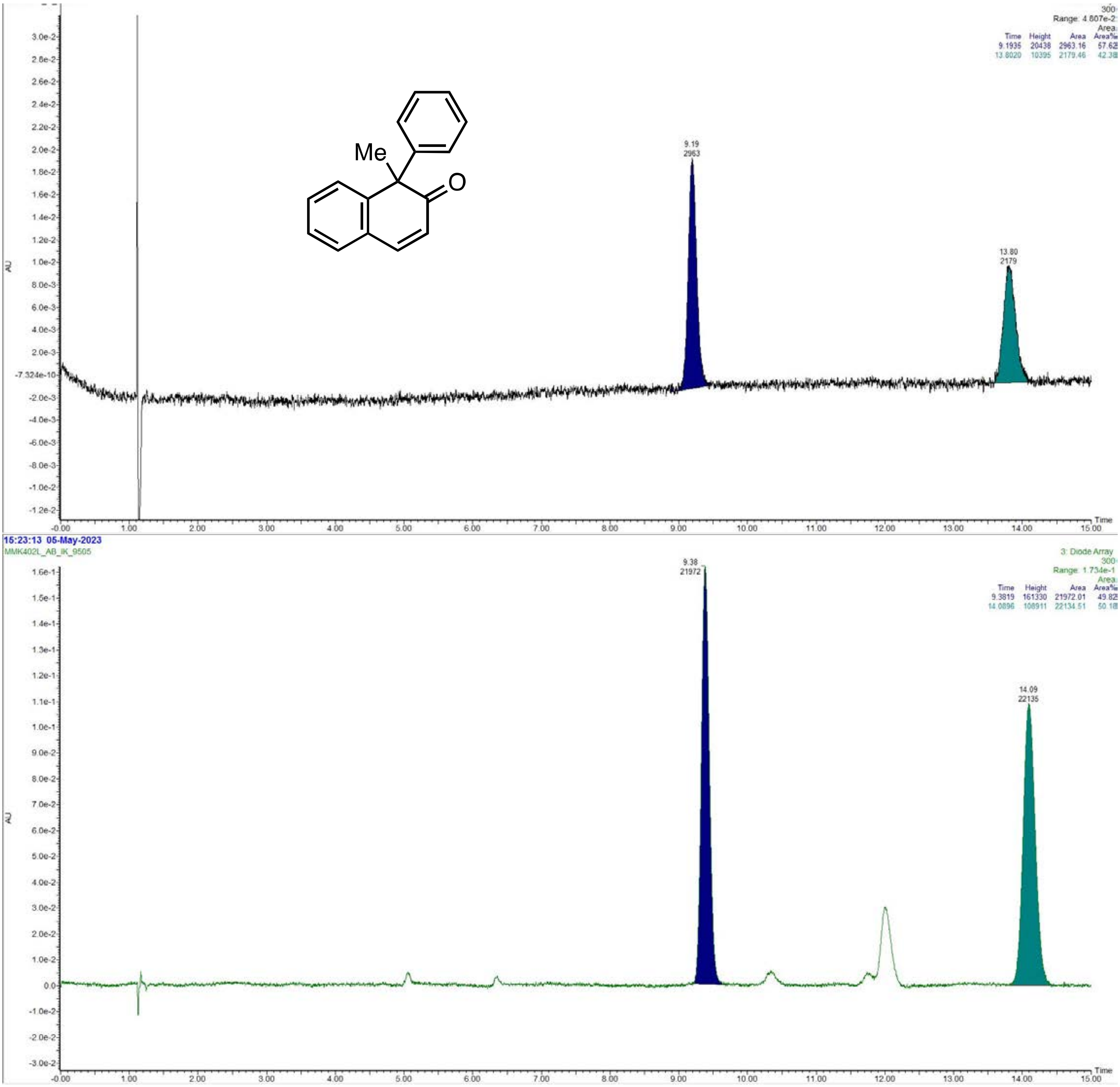

(3*S*,4*R*)-4-Methyl-4-phenyl-3-vinyl-3,4-dihydronaphthalen-1(2*H*)-one (4a)

Chiral SFC Analysis: CHIRALPAK IE (CO_2:MeOH, 90:10, 2.5 mL min^{-1}, 40 °C, 250 nm) indicated 98% *ee*, t_R = 3.72 (minor), 4.00 (major) minutes.

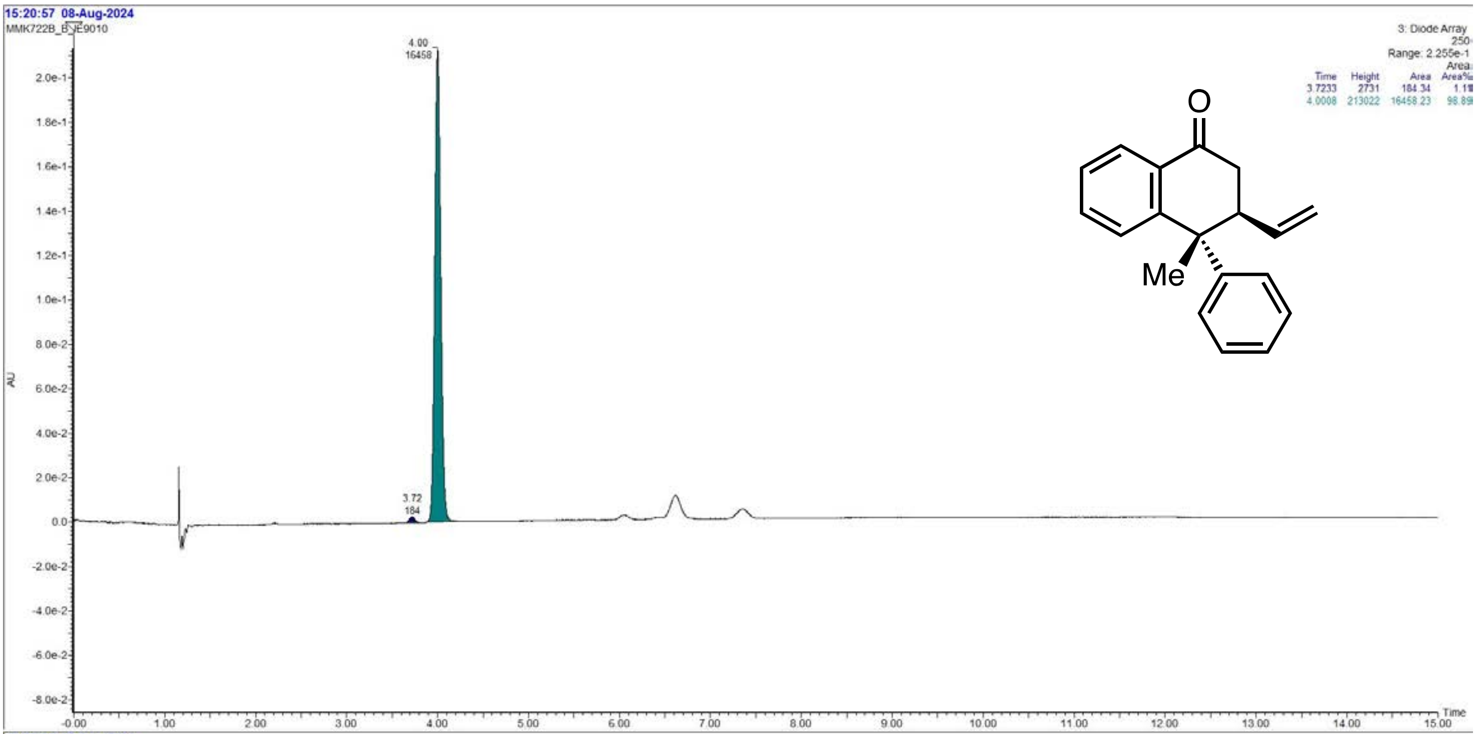

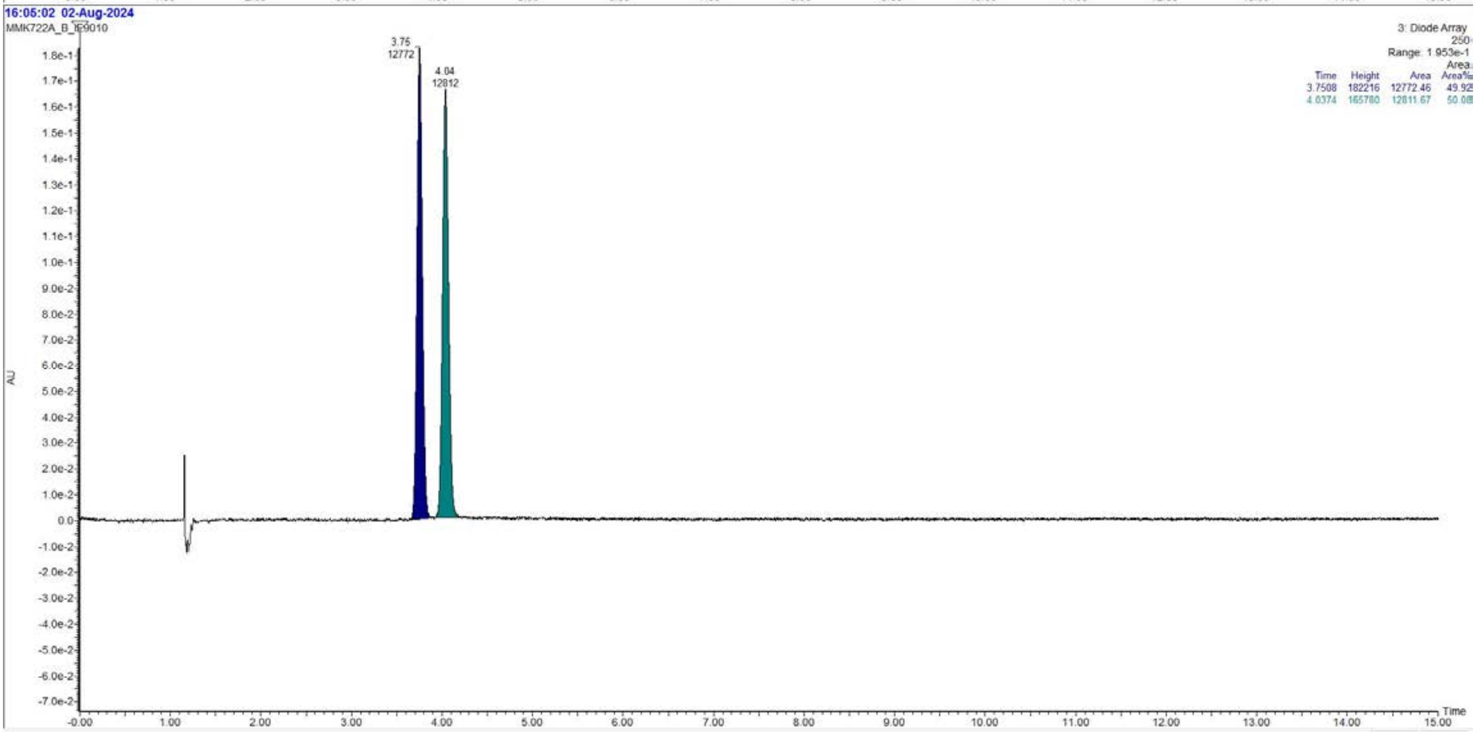

(1a*S*,7*R*,7a*S*)-7-Methyl-7-phenyl-7,7a-dihydronaphtho[2,3-*b*]oxiren-2(1a*H*)-one (4b)

Chiral SFC Analysis: CHIRALPAK IE (CO_2:MeOH, 94:06, 2.5 mL min^{-1}, 40 °C, 220 nm) indicated 97% *ee*, t_R = 6.52 (major), 7.06 (minor) minutes.

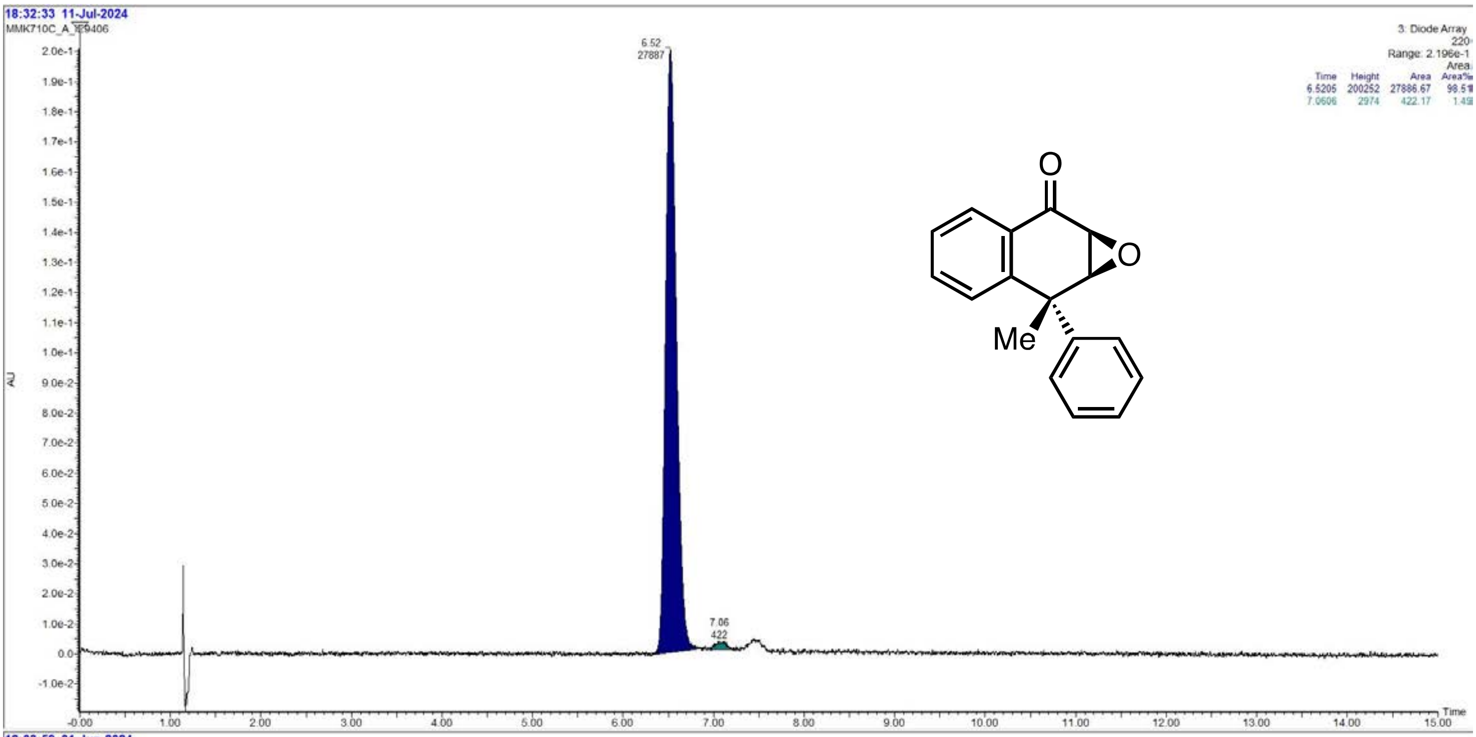

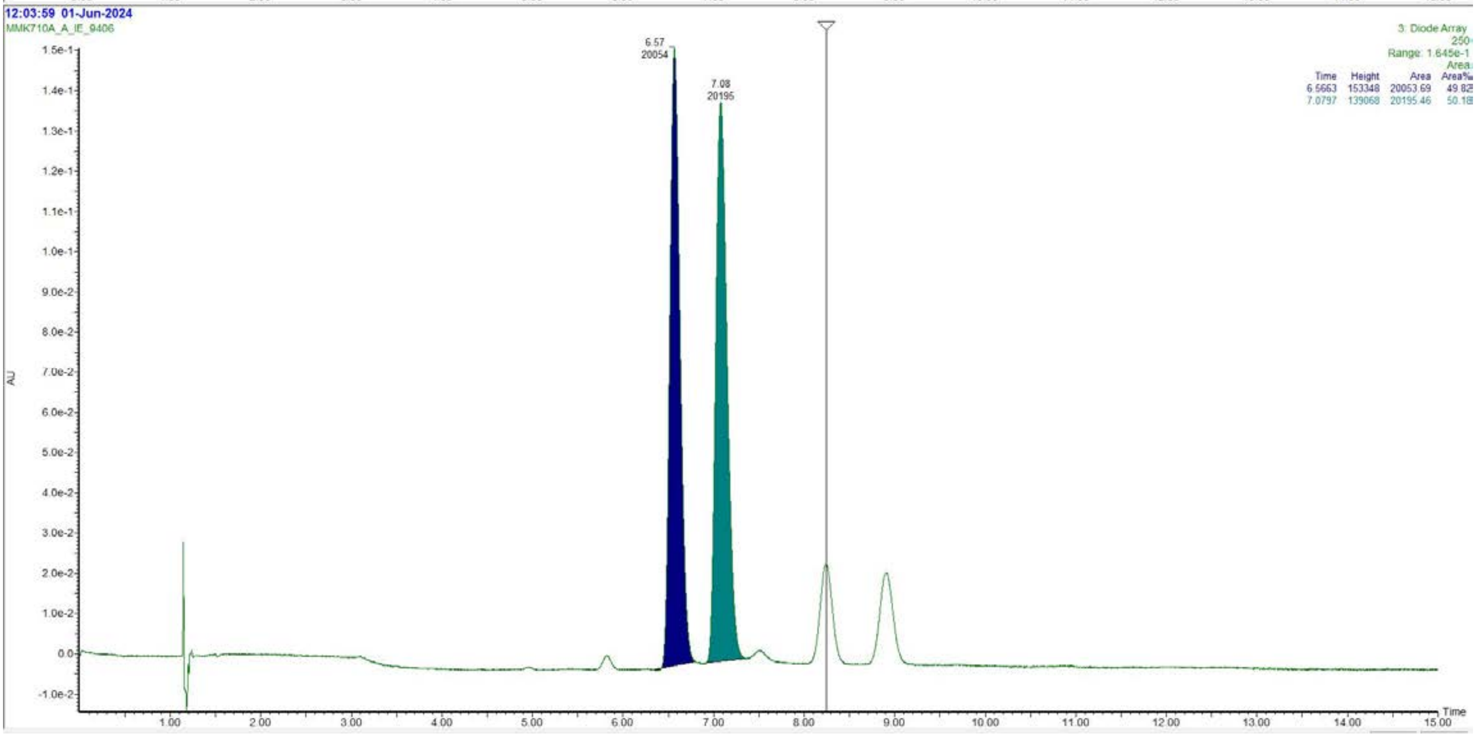

(*S*)-4-(3,4-dichlorophenyl)-4-Methylnaphthalen-1(4*H*)-one (5a) (0.1 mmol scale)

Chiral SFC Analysis: CHIRALPAK IE (CO_2:MeOH, 90:10, 2.5 mL min^{-1}, 40 °C, 250 nm) indicated 94% *ee*, t_R = 7.17 (major), 7.68 (minor) minutes.

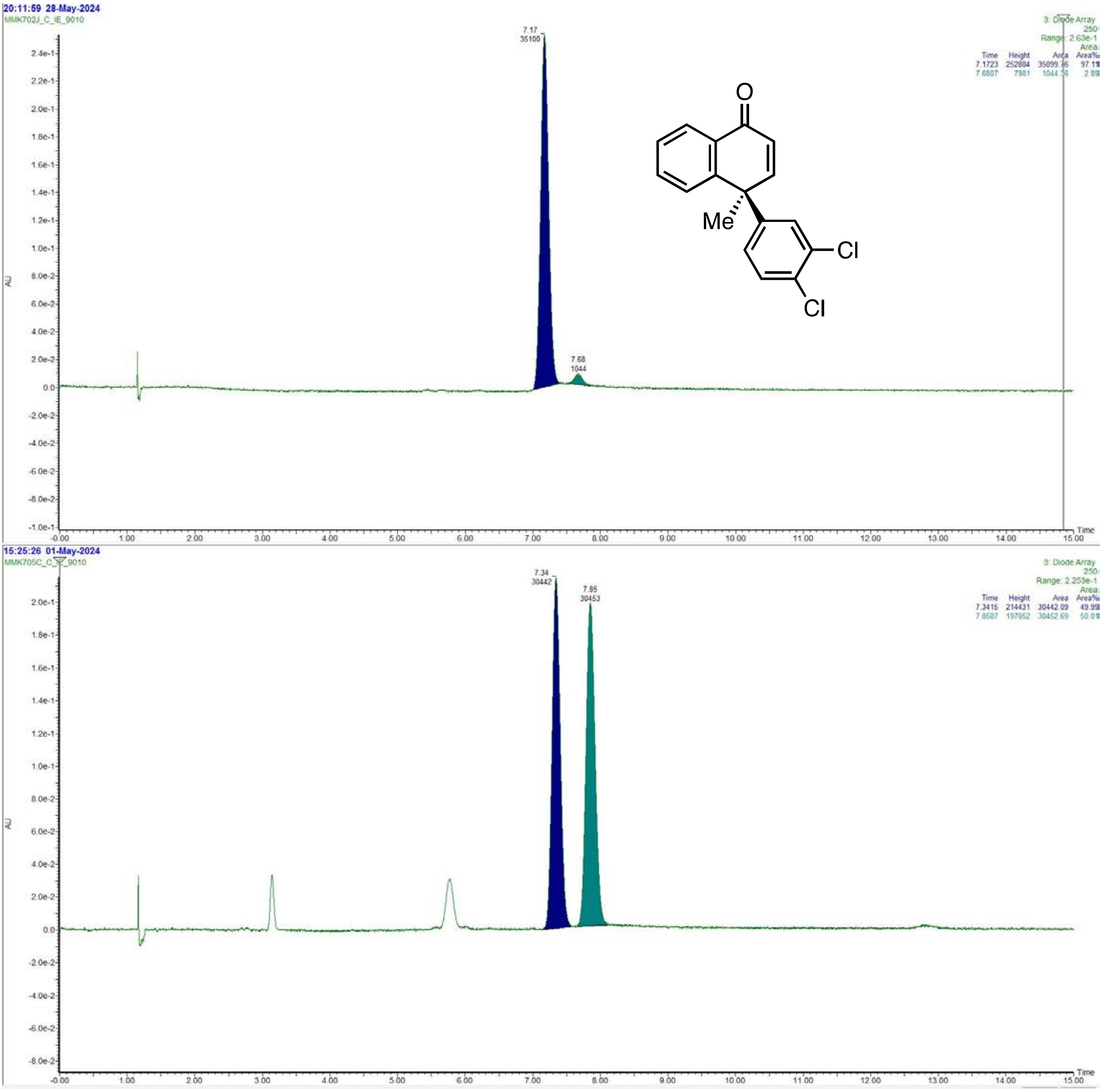

(*S*)-4-(3,4-dichlorophenyl)-4-Methylnaphthalen-1(4*H*)-one (5a) (2 mmol scale)

Chiral SFC Analysis: CHIRALPAK IE (CO_2:MeOH, 90:10, 2.5 mL min^{-1}, 40 °C, 250 nm) indicated 92% *ee*, t_R = 7.29 (major), 7.80 (minor) minutes.

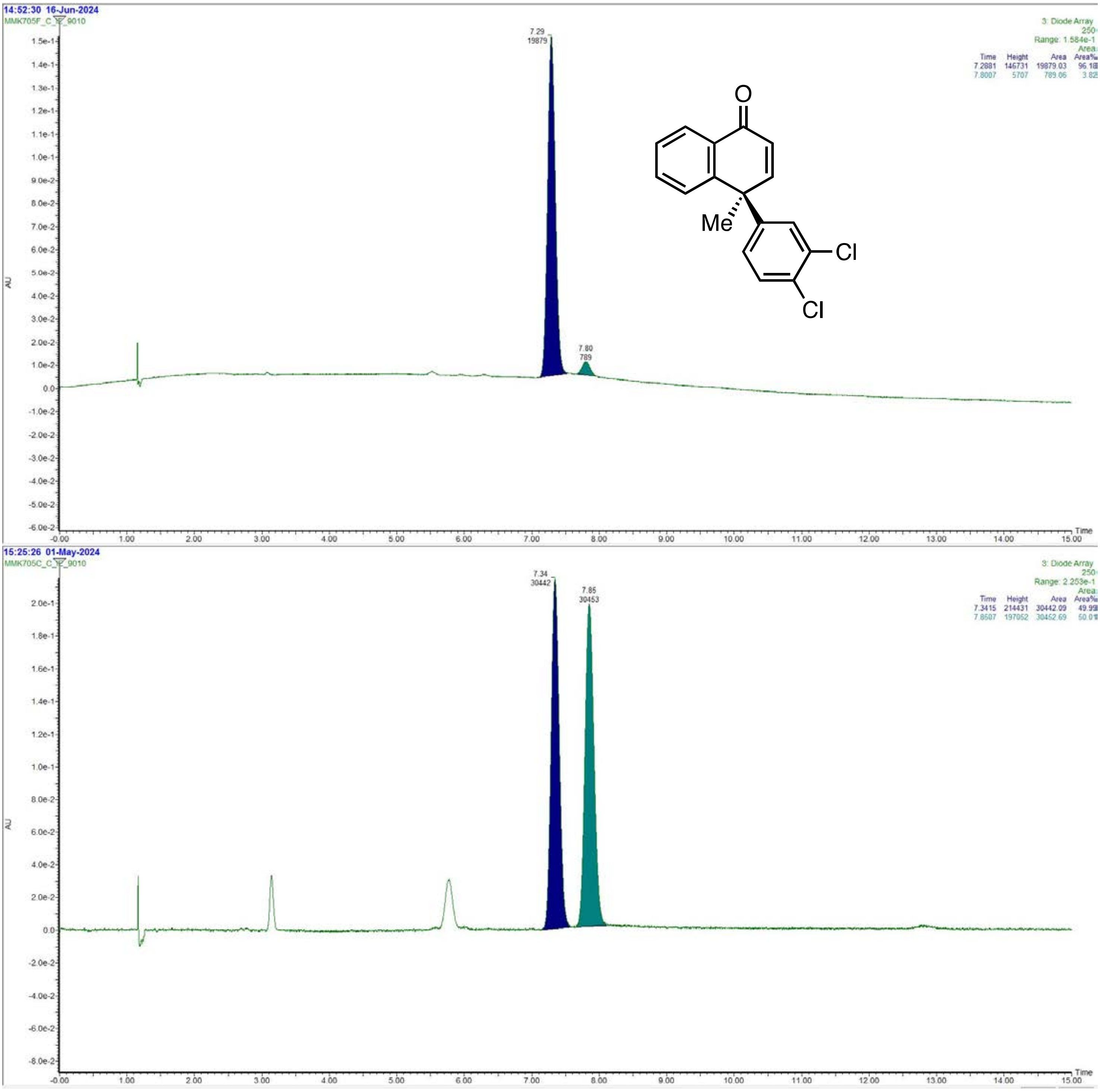

(*S*)-4-(3,4-dichlorophenyl)-4-Methyl-3,4-dihydronaphthalen-1(2*H*)-one (5b)

Chiral SFC Analysis: CHIRALPAK IE (CO_2:MeOH, 90:10, 2.5 mL min^{-1}, 40 °C, 220 nm) indicated 92% *ee*, t_R = 5.94 (major), 6.41 (minor) minutes.

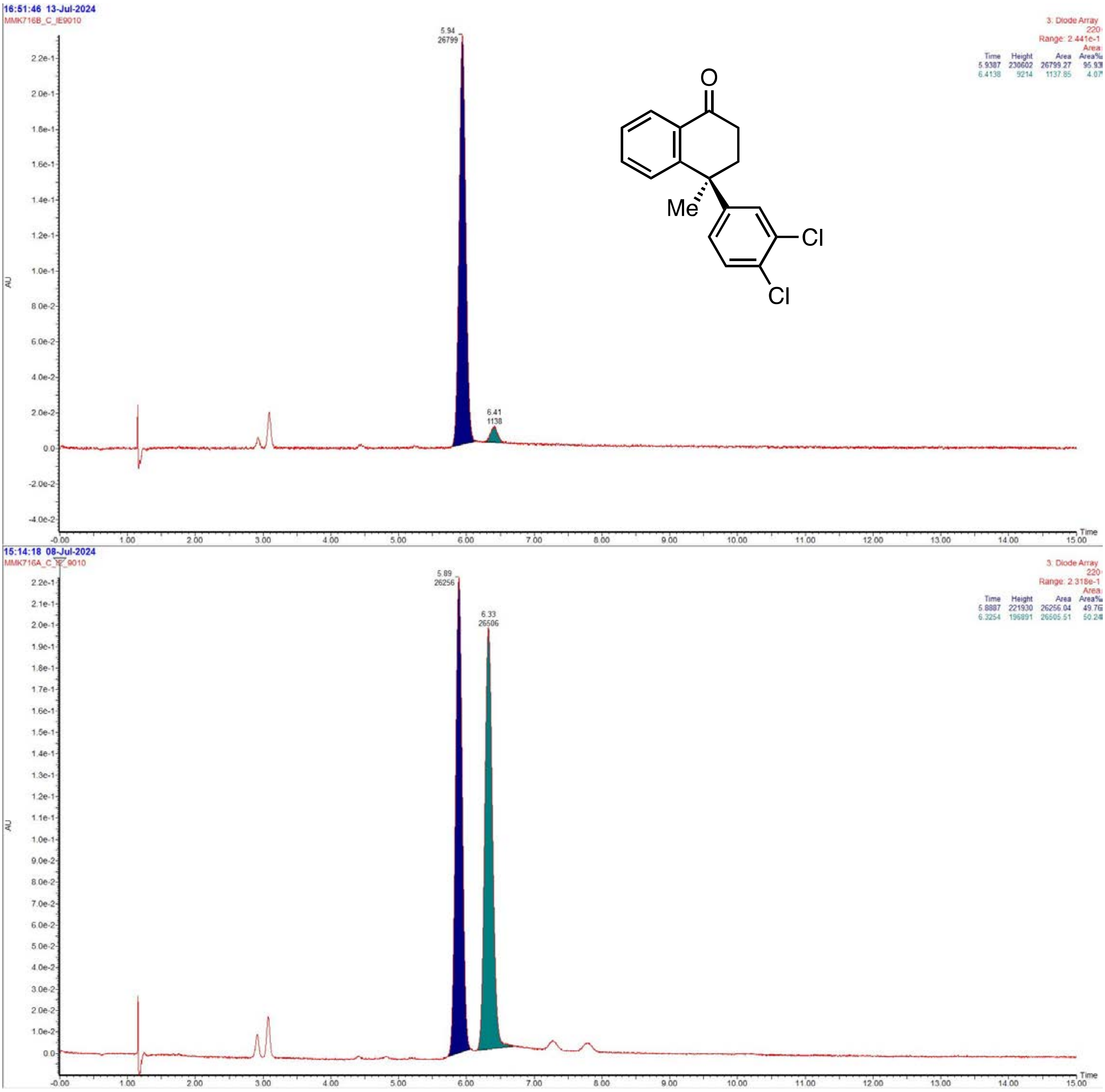

(1*S*,4*S*)-4-(3,4-dichlorophenyl)-4-Methyl-1,2,3,4-tetrahydronaphthalen-1-ol (5cb)

Chiral SFC Analysis: CHIRALPAK IE (CO_2:MeOH, 90:10, 2.5 mL min^{-1}, 40 °C, 220 nm) indicated 92% *ee*, t_R = 5.46 (major), 6.93 (minor) minutes.

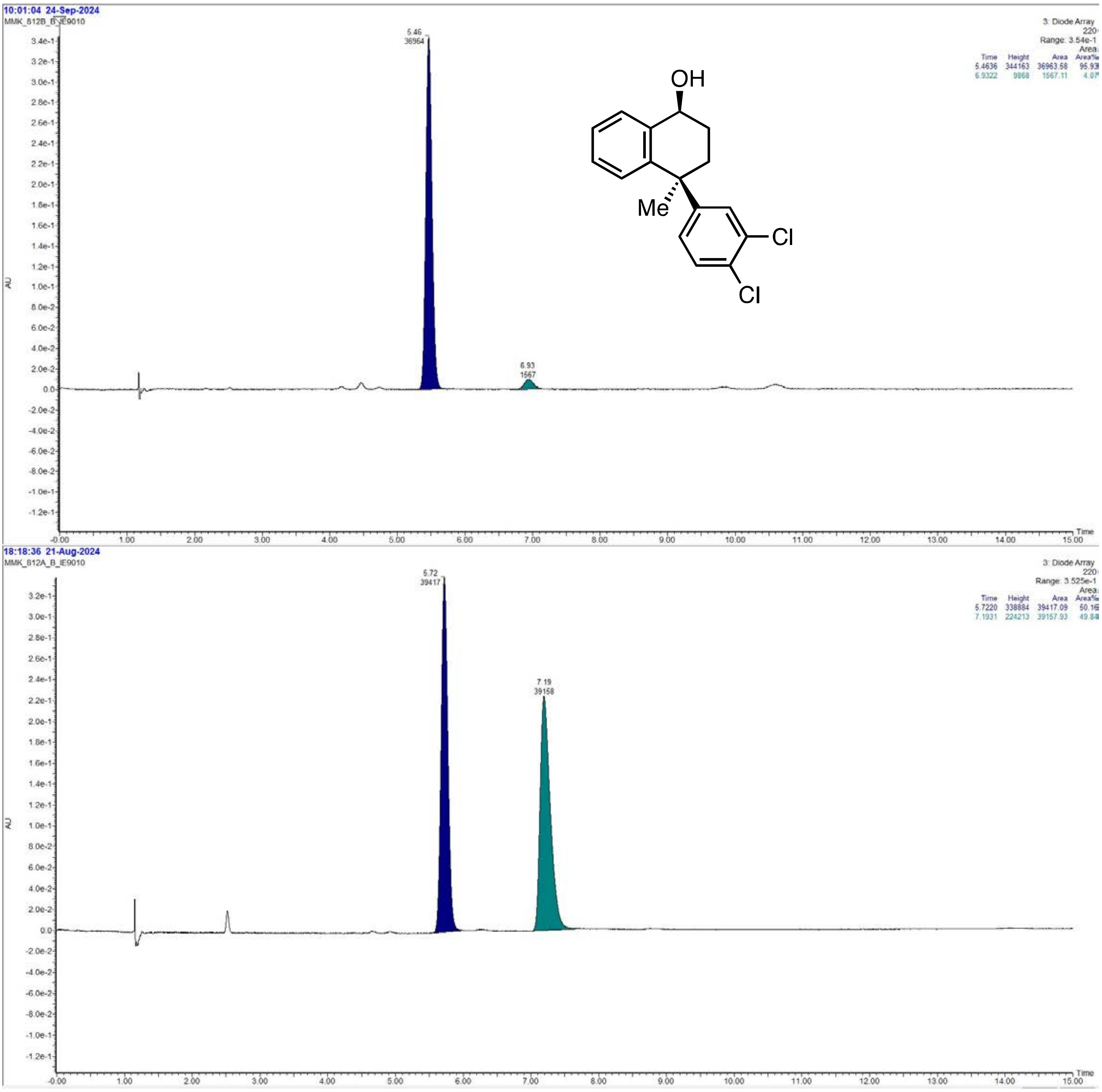

(1*R*,4*S*)-4-(3,4-dichlorophenyl)-4-Methyl-1,2,3,4-tetrahydronaphthalen-1-ol (5ca)

Chiral SFC Analysis: CHIRALPAK IE (CO_2:MeOH, 90:10, 2.5 mL min^{-1}, 40 °C, 220 nm) indicated 93% *ee*, t_R = 5.62 (major), 6.07 (minor) minutes.

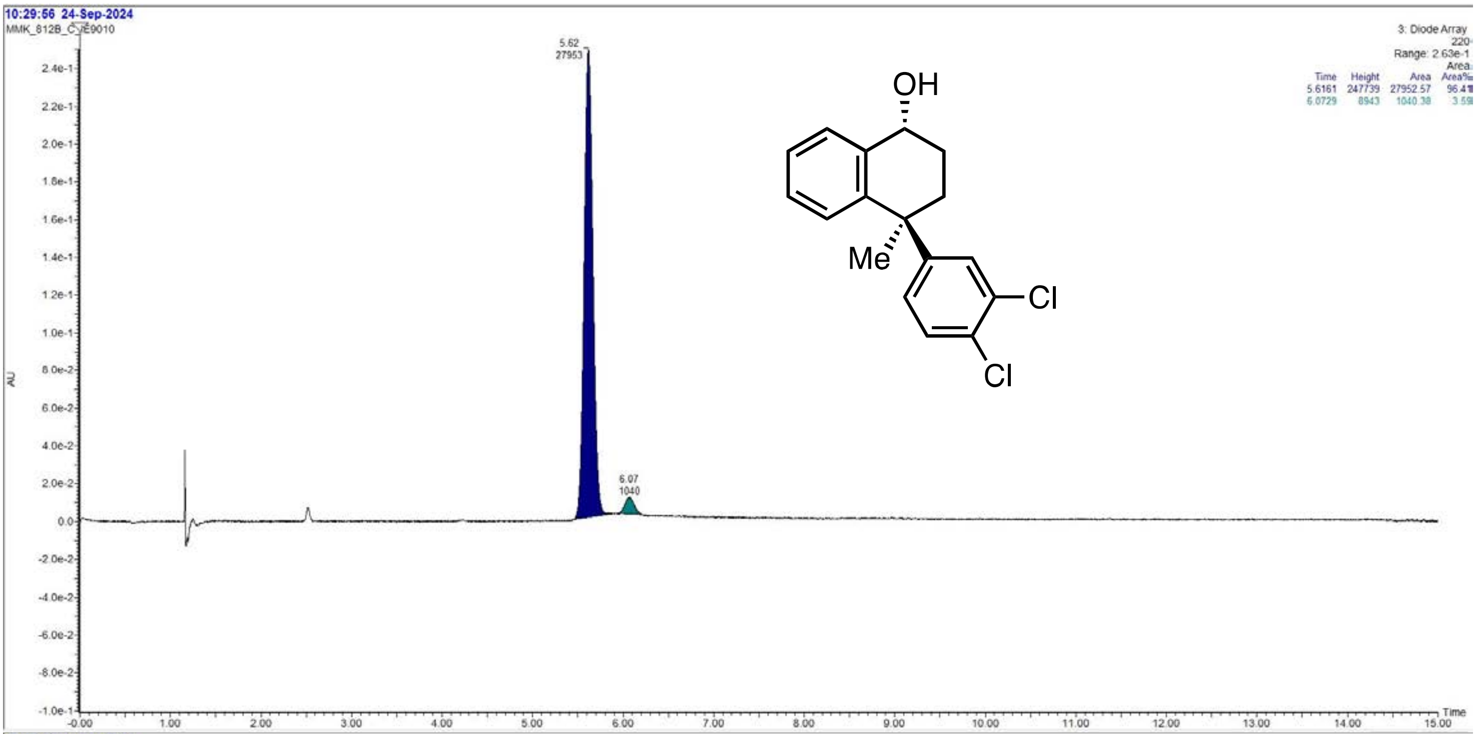

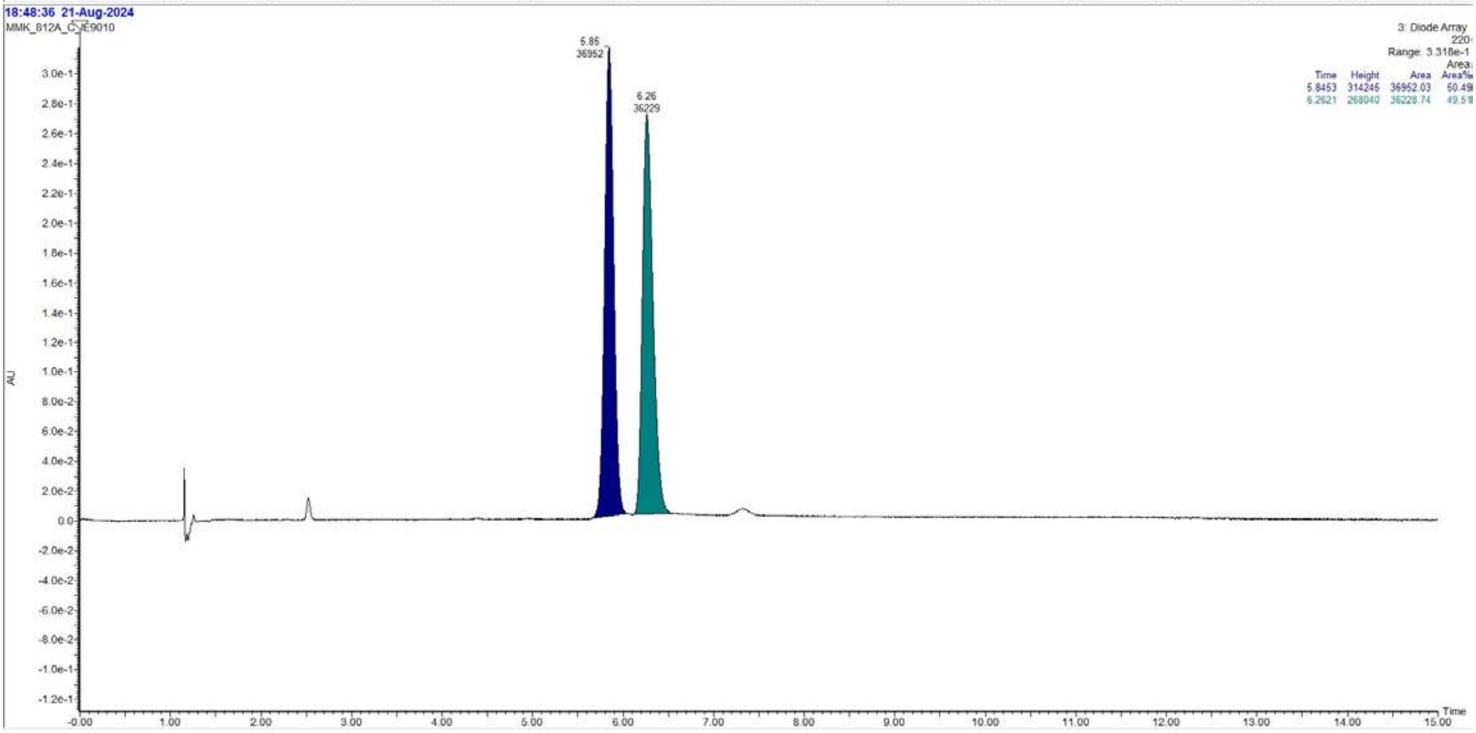

(1*S*,4*R*)-4-Azido-1-(3,4-dichlorophenyl)-1-methyl-1,2,3,4-tetrahydronaphthalene (S19b)

Chiral SFC Analysis: CHIRALPAK IJ (CO_2:MeOH, 95:05, 2.5 mL min^{-1}, 40 °C, 220 nm) indicated 91% *ee*, t_R = 5.09 (major), 6.25 (minor) minutes.

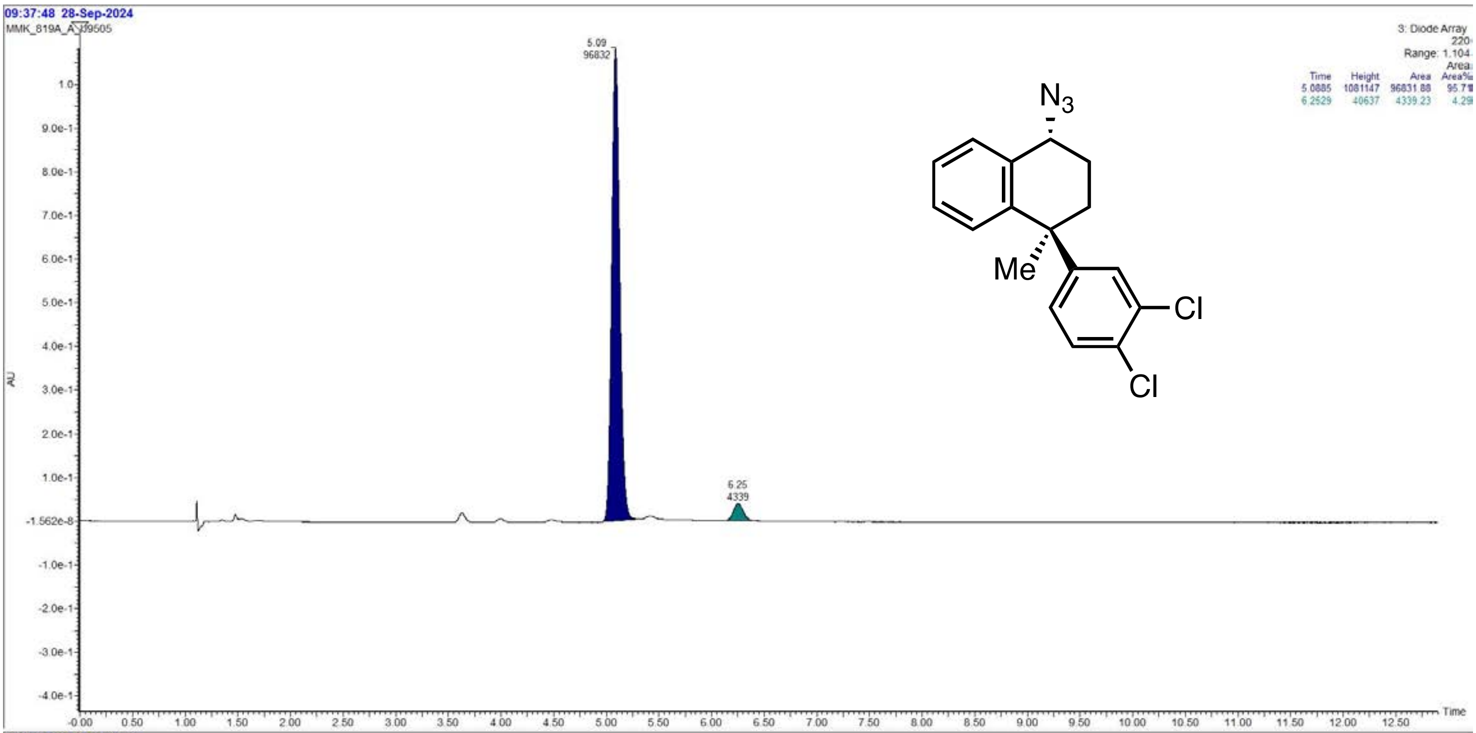

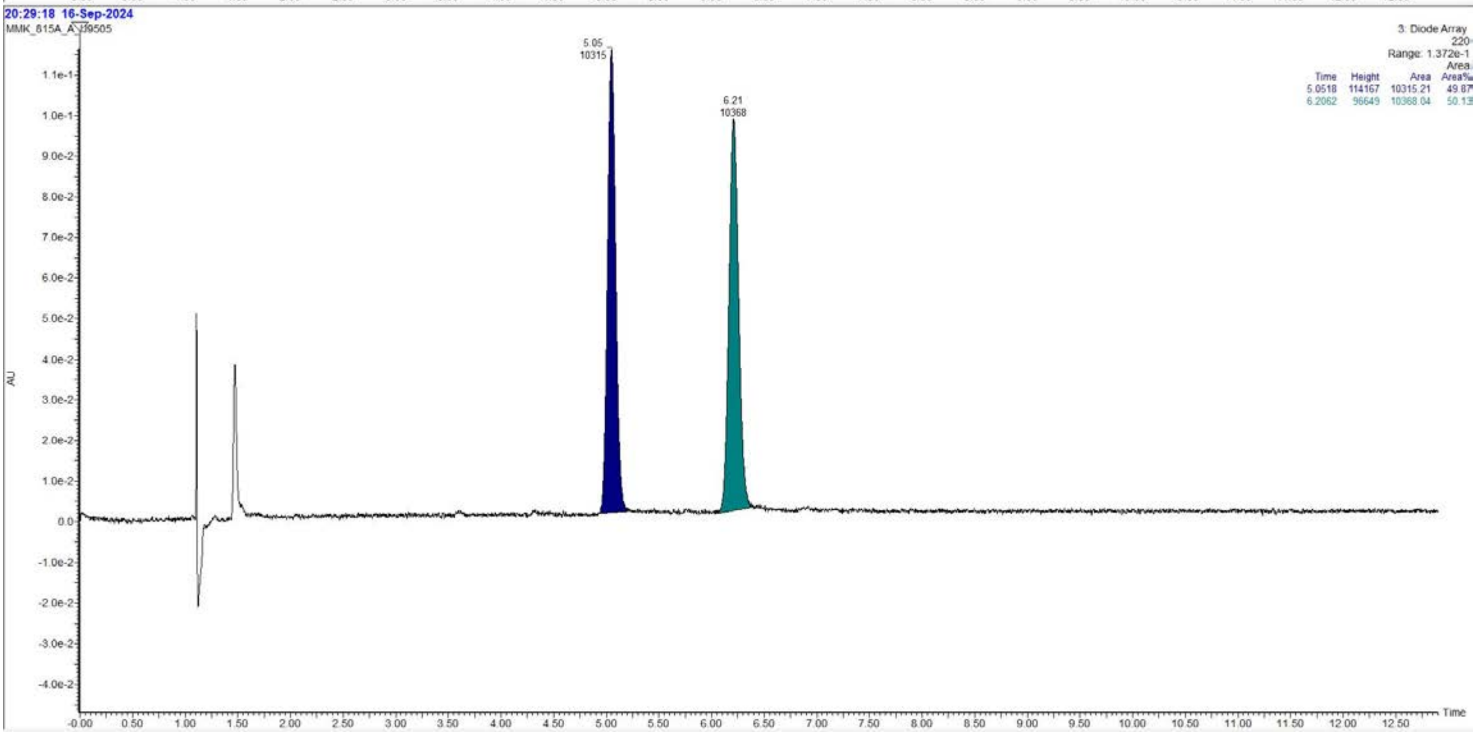

(1*S*,4*S*)-4-Azido-1-(3,4-dichlorophenyl)-1-methyl-1,2,3,4-tetrahydronaphthalene (S19a)

Chiral SFC Analysis: CHIRALPAK IK (CO_2:MeOH, 90:10, 1.25 mL min^{-1}, 40 °C, 230 nm) indicated 94% *ee*, t_R = 8.90 (major), 9.21 (minor) minutes.

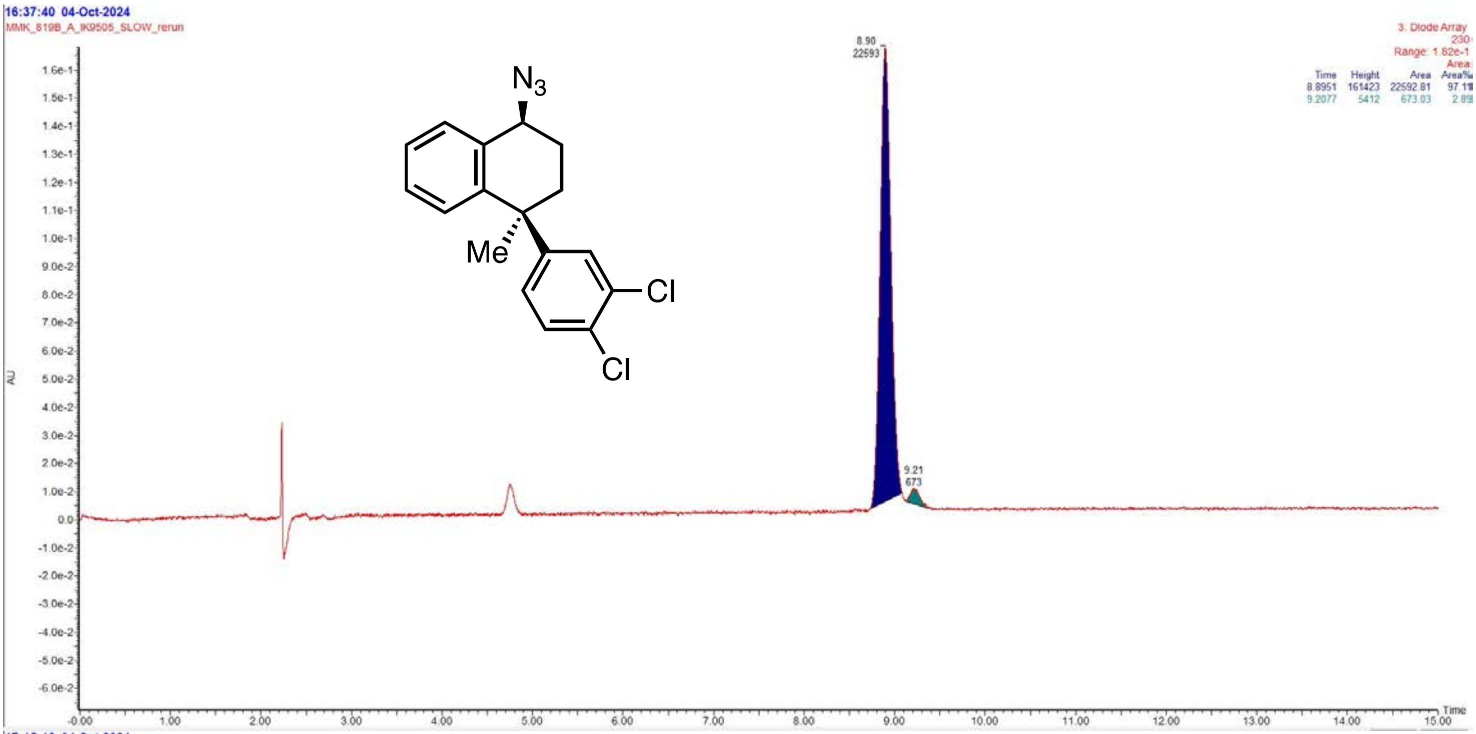

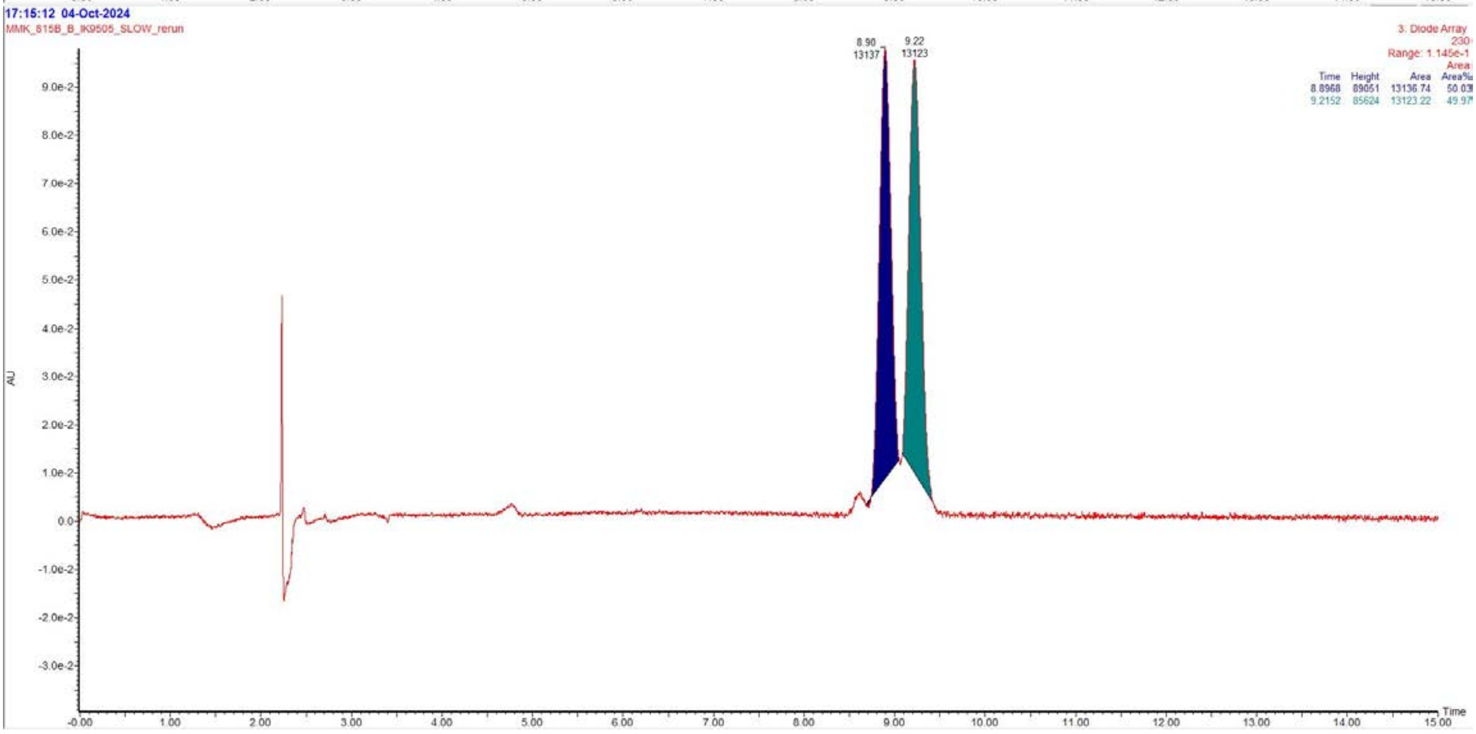

(*R*)-4-(3,4-bis(methoxymethoxy)phenyl)-6-(methoxymethoxy)-4,7-Dimethylnaphthalen-1(4*H*)-one (11)

Chiral SFC Analysis: CHIRALPAK IE (CO_2:MeOH, 90:10, 2.5 mL min^{-1}, 40 °C, 230 nm) indicated 84% *ee*, t_R = 10.95 (major), 11.90 (minor) minutes.

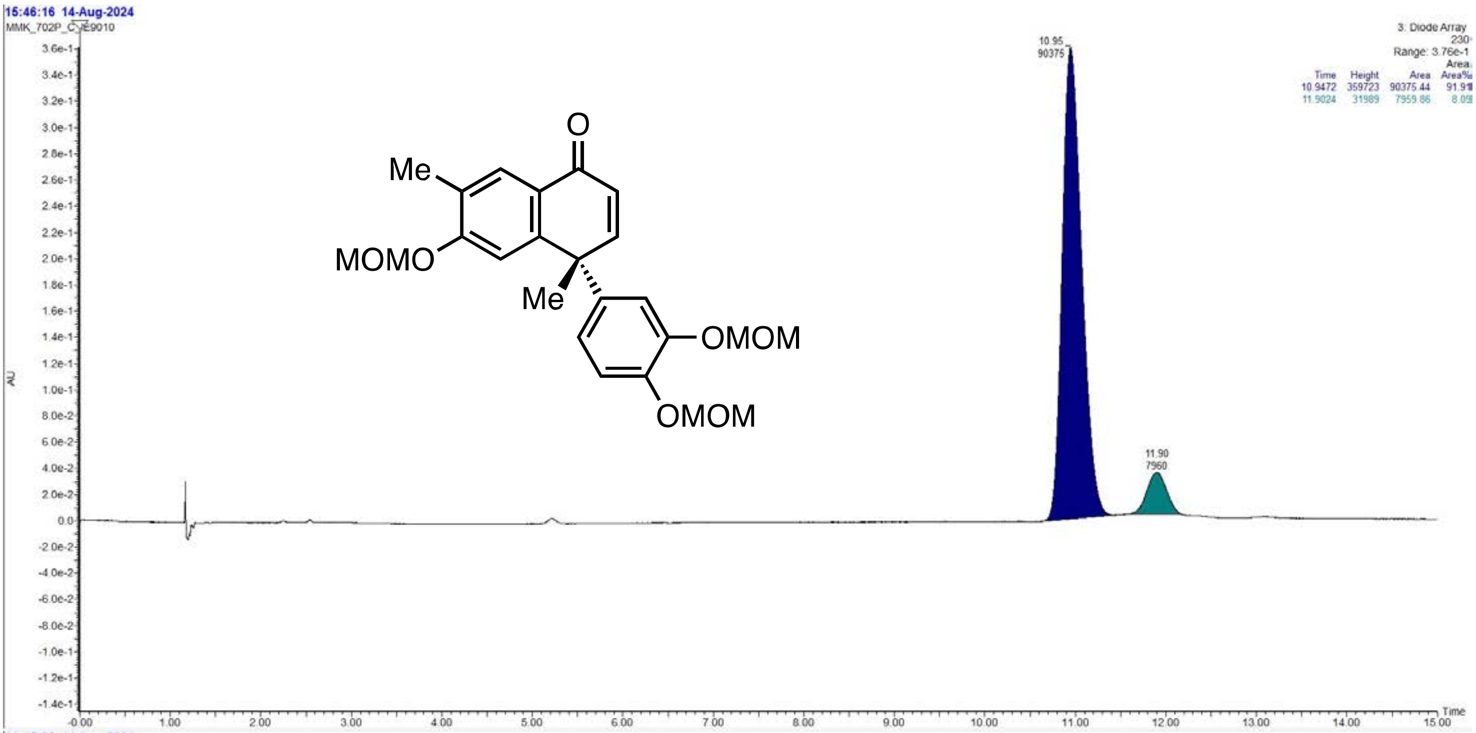

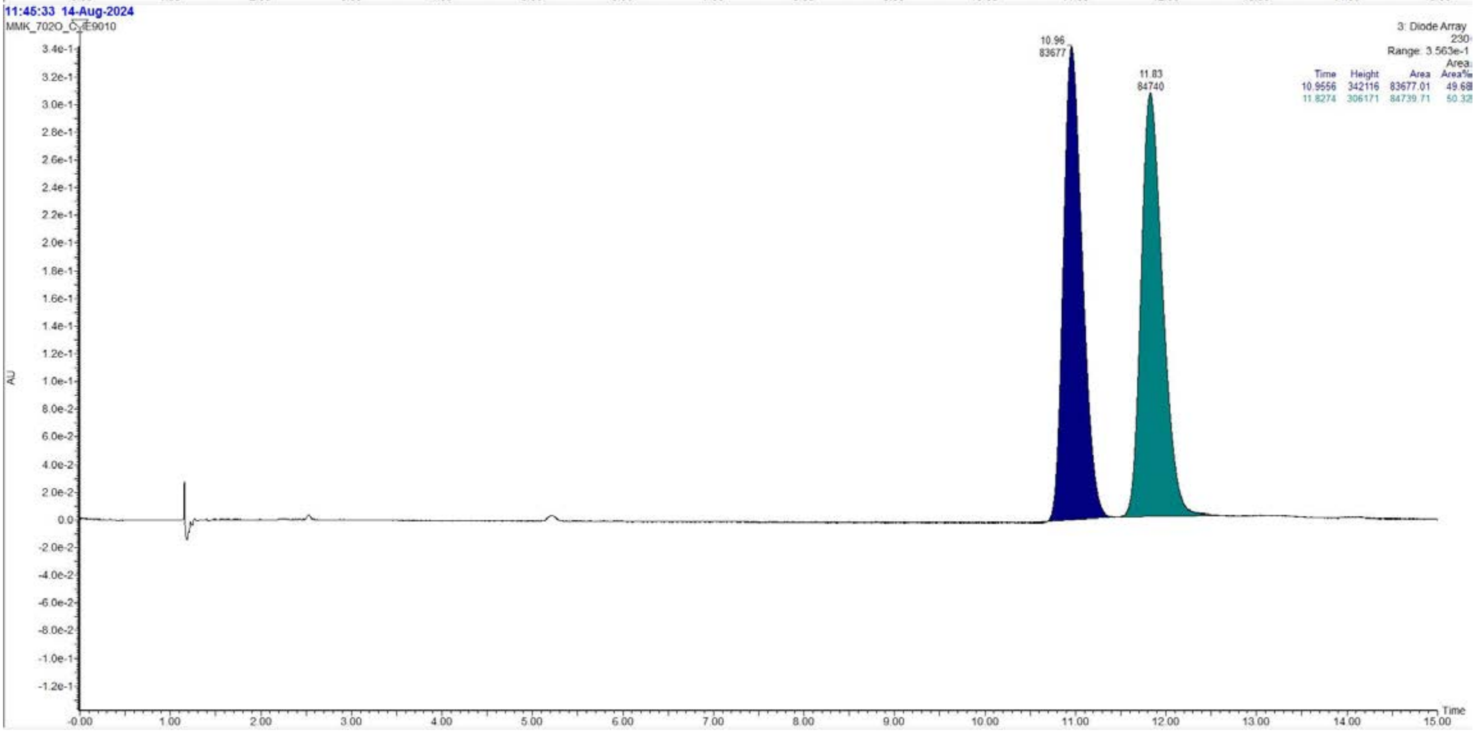

(*R*)-4-(3,4-bis(methoxymethoxy)phenyl)-6-(methoxymethoxy)-4,7-Dimethyl-3,4-dihydronaphthalen-1(2*H*)-one (7)

Chiral SFC Analysis: CHIRALPAK IE (CO_2:MeOH, 90:10, 2.5 mL min^{-1}, 40 °C, 270 nm) indicated 82% *ee*, t_R = 8.97 (major), 10.03 (minor) minutes.

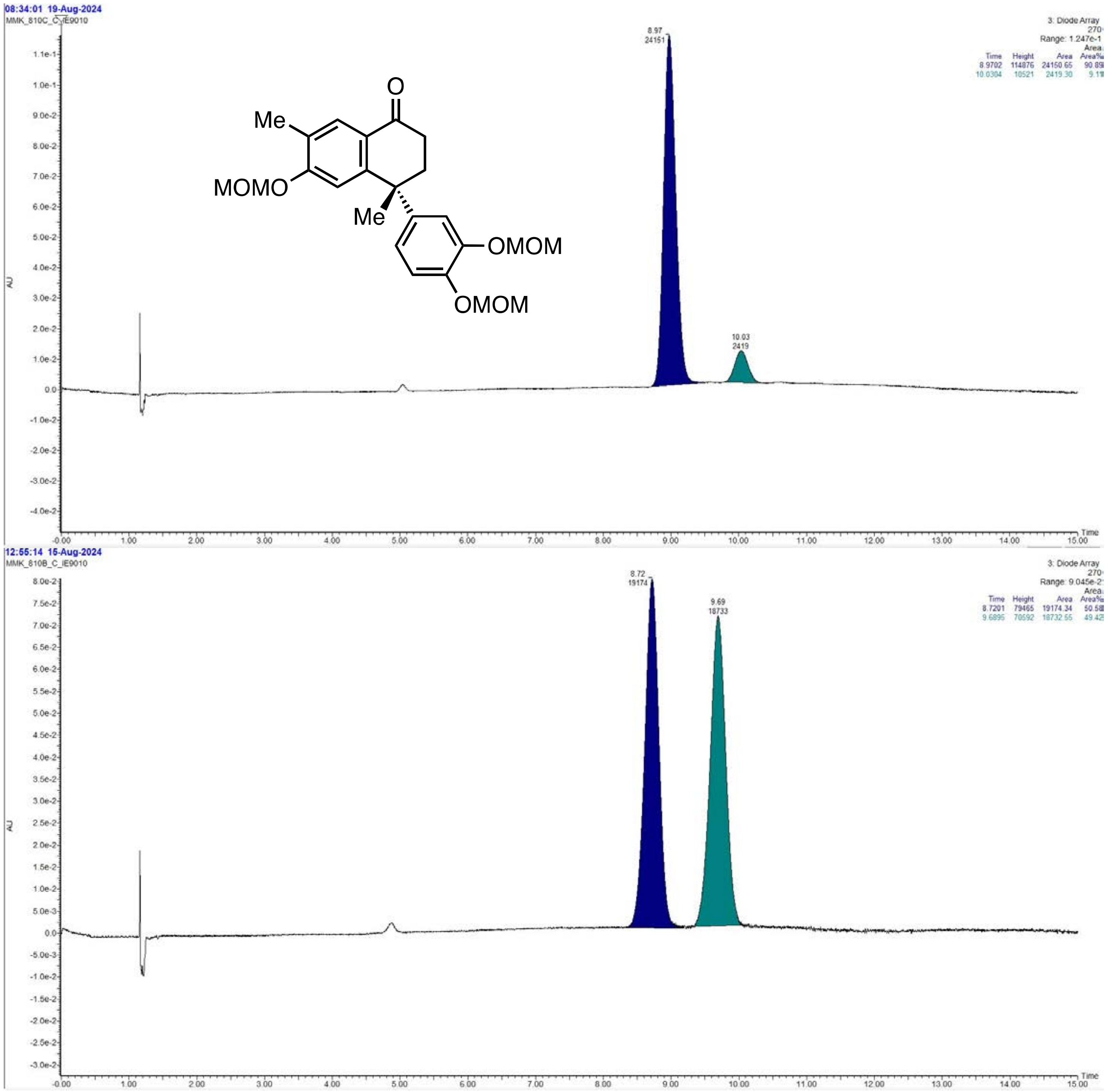

2,3-Dihydro-2'*H*-spiro[indene-1,1'-naphthalen]-2'-one (S25)

Chiral SFC Analysis: CHIRALPAK IK (CO_2:MeOH, 85:15, 2.5 mL min^{-1}, 40 °C, 300 nm) indicated 12% *ee*, t_R = 5.23 (major), 6.24 (minor) minutes.

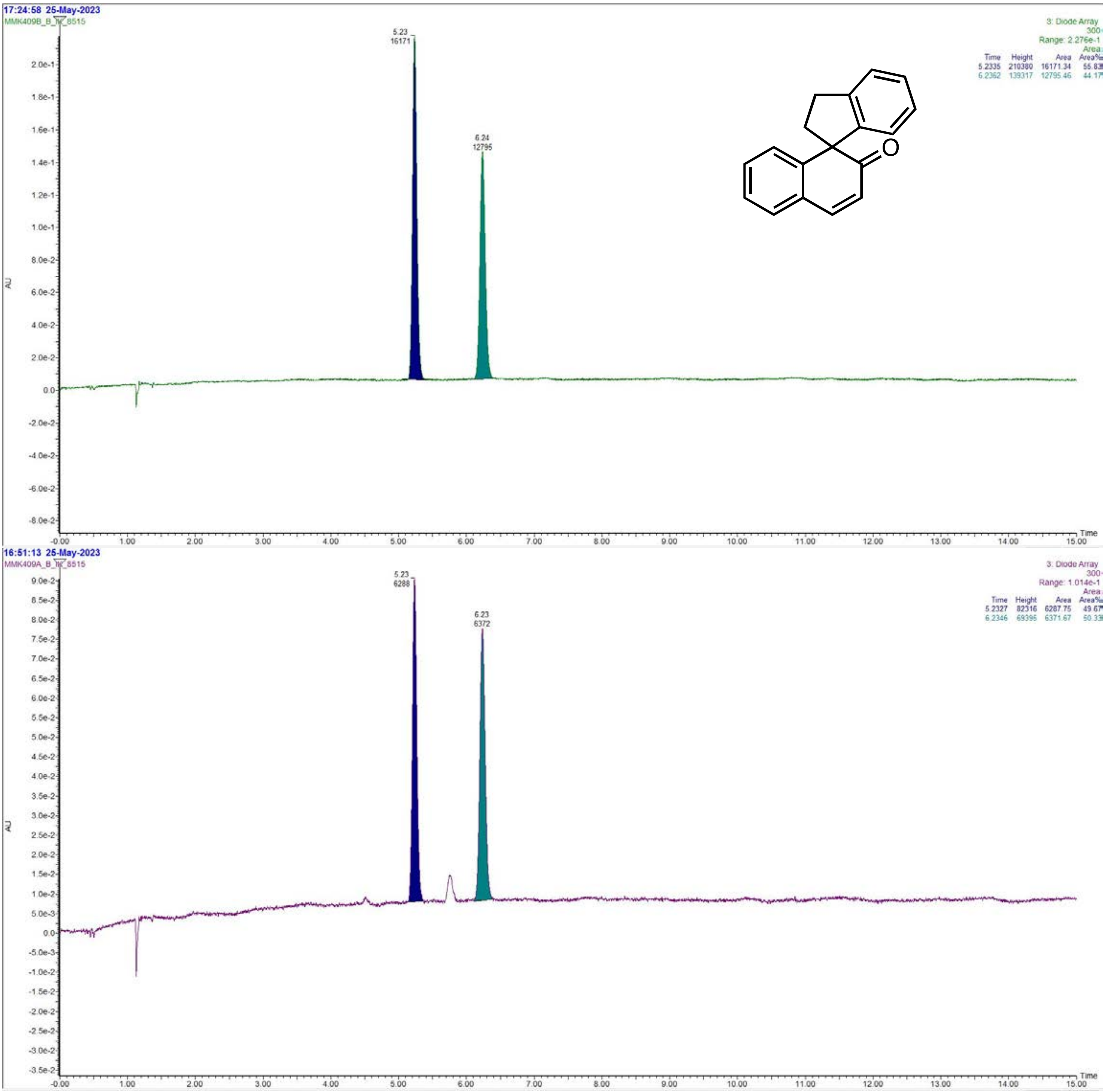

Supplement: Supplementary file 1 — ja4c14754_si_001.pdf [file ja4c14754_si_001.pdf]
